# Supplementary material for: Atroposelective hydroarylation of biaryl phosphines directed by phosphorus centres
Source: Nat Commun. 2023 Dec 21;14:8509. doi: 10.1038/s41467-023-44202-1 (PMC10739911; doi:10.1038/s41467-023-44202-1)
Supplement: Supplementary file 1 — Supplementary Information [file 41467_2023_44202_MOESM1_ESM.pdf]

Supplementary Information for

Atroposelective Hydroarylation of Biaryl Phosphines directed by phosphorus centres

Zexian Li[1,2,4], Minyan Wang[2,4], Youqing Yang[1], Yong Liang[2], Xiangyang Chen[3], Yue Zhao[2], K. N. Houk[3], and Zhuangzhi Shi[1,2]*

[1]Key Laboratory of Green and Precise Synthetic Chemistry and Applications, Ministry of Education, Huaibei Normal University, Huaibei 235000 (China)

[2]State Key Laboratory of Coordination Chemistry, Chemistry and Biomedicine Innovation Center (ChemBIC), School of Chemistry and Chemical Engineering, Nanjing University, Nanjing 210093 (China)

[3]Department of Chemistry and Biochemistry, University of California Los Angeles, CA 90095 (USA)

[4]These authors contributed equally to this work

*e-mail: shiz@nju.edu.cn

Table of Contents

1. Supplementary Notes 3

2. Supplementary Methods 4

2.1 General Procedure for Synthesis of Starting Materials 4
2.2 Optimization of the reaction conditions 19
2.3 Experimental Procedures and Characterization of Products 23
2.3.1 asymmetric C–H hydroarylation with alkynes 23
2.3.2 asymmetric C–H hydroarylation with alkenes 49
2.4 Mechanistic Studies 81
2.4.1 Isolation of iridium complex 15 81
2.4.2 Test of the reactivity of 15 and 3aa for C–H activation of 1a 82
2.4.3 Observation of H/D exchange during the reaction of d-1a with alkyne 2a. 82
2.4.4 Kinetic isotope effect (KIE) experiments 84
2.4.5 Nonlinear Effects 85
2.4.6 Computational details 86
2.5 Crystallographic Data 93
2.6 Copies of ^{1}H NMR, ^{13}C NMR, ^{19}F NMR and ^{31}P NMR Spectra 137
2.7 Copies HPLC Spectra 267

3. Supplementary References 326

1. Supplementary Notes

Unless otherwise mentioed, all reactions were performed under an argon atmosphere using flame-dried glasswares. All new compounds were fully characterized. NMR-spectra were recorded on Bruker ARX-400 MHz or a ARX-500 Associated. Chemical shifts (δ values) were reported in ppm with $CDCl_3$. Coupling constants were reported in Hz, and multiplicity was indicated as follows: s (singlet); d (doublet); t (triplet); q (quartet); hept (heptet); m (multiplet); dd (doublet of doublets); dt (doublet of triplets); dq (doublet of quartets); ddd (doublet of doublet of doublets). Mass spectra were conducted at Micromass Q-Tof instrument (ESI) and Agilent Technologies 5973N (EI). IR spectra were recorded on a FT-IR spectrometer. Optical rotations were measured on an automatic polarimeter with $[\alpha]_D^{25}$ values reported in degrees; concentration (*c*) is in g/100 mL. Chiral HPLC analyses were performed on an UltiMate 3000 liquid chromatography. All reactions were carried out in flame-dried reaction vessels (25 mL) with Teflon screw caps under argon. $[Ir(cod)Cl]_2$ was purchased from ALDRICH. **L2**- **L5** were purchased from DAICEL. Unless otherwise noted, materials were obtained from commercial suppliers and used without further purification.

2. Supplementary Methods

2.1 General Procedure for Synthesis of Starting Materials

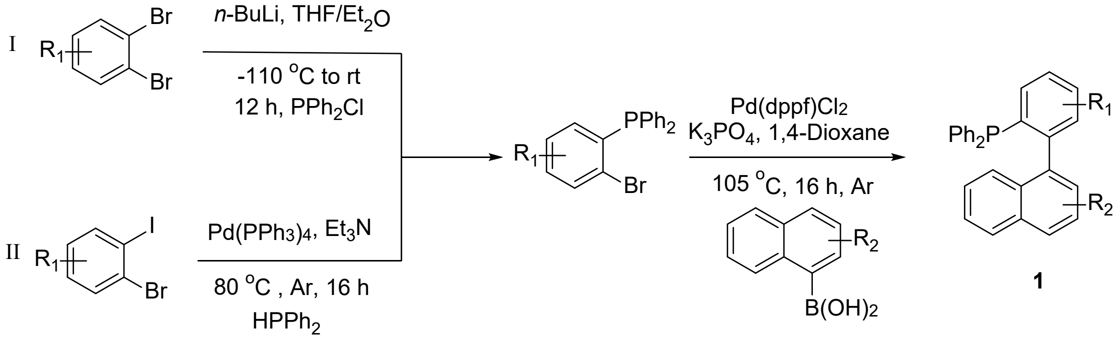

1

According to the literature[1-2], route **I**: to a solution of 1,2-dibromobenzene (12.9 g, 53.5 mmol) in Et_2O/THF (100 mL/100mL) at -110 ºC (with a coldbath compose of Et_2O and liquid N_2) was added dropwise a solution of *n*-BuLi (2.5 M in hexane, 20 mL, 50 mmol). The resulting mixture is stirring at -110 ºC for 50 minutes then followed by the addition of a solution of Ph_2PCl (9.2 mL, 11.0 g) in THF (5 ml). At this temperature, stirring for 1 h and then allowed to warm to rt 12 h. The resulting solution was cooled to -80 ºC and then added a saturated solution of NH_4Cl (80 mL). The resulting mixture was allowed to warm to rt and separated. The aqueous phase was extracted by Et_2O (3×50 mL) and the combined organic phases was dried with Na_2SO_4 and evaporated in vacuum. The crude product was recrystallized with methanol to give (2-bromophenyl)diphenylphosphane as a white solid. Route **II**: 2-iodobromobenzene (2.41 g, 8.53 mmol), diphenylphosphine (1.6 g, 8.53 mmol), triethylamine (10.1 g, 10.0 mmol), and a catalytic amount of $Pd(PPh_3)_4$ (51.3 mg, 0.045 mmol) were dissolved in 1.5 mL of toluene to give a clear, bright yellow solution. The solution was heated at 80 °C with stirring for 16 hours in a sealed tube, resulting in the precipitation of triethylammonium iodide. The resulting orange solution was dried in vacuo at 50 °C, extracted into diethyl ether (40 mL), and filtered through a pad of silica gel to give a clear, pale yellow solution, from which the volatiles were removed in vacuo to yield the phosphine as a creamy white powder

According to the literature[3], a two-necked flask was charged with Pd(dppf)Cl_2 (36.5 mg, 0.05 mmol), (2-bromophenyl)diphenylphosphine (1.7 g, 5.0 mmol), 1-naphthylboronic acid (2.58 g, 15 mmol), anhydrous K_3PO_4 (3.5 g, 20 mmol) and dry 1,4-dioxane (35 mL). The reaction mixture was heated at 105 °C with vigorous stirring for 16 h. After cooling to room temperature, the reaction mixture was diluted with water (100 mL) and extracted with Et_2O (3 × 50 mL). The combined organic extracts were dried over $MgSO_4$ and concentrated under reduced pressure. The crude material obtained was purified by flash column chromatography (PE/EtOAc = 200/1) to give pure **1** as a white solid.

(2-(Naphthalen-1-yl)phenyl)diphenylphosphane (1a)

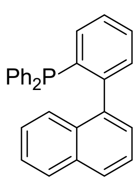

1a

Routes **I**, with 10.0 mmol scale, **1a** was prepared from commercially available 1,2-dibromobenzene and 1-naphthylboronic acid to obtain a white solid (2.37 g, 61 %, two steps). **^{1}H NMR (500 MHz, CDCl$_3$)** δ 7.83 (d, *J* = 8.1 Hz, 1H), 7.79 (d, *J* = 8.2 Hz, 1H), 7.46 – 7.38 (m, 3H), 7.37 – 7.31 (m, 2H), 7.31 – 7.25 (m, 5H), 7.23 – 7.18 (m, 3H), 7.18 – 7.13 (m, 3H), 7.12 – 7.08 (m, 2H), 7.08 – 7.03 (m, 1H); **^{13}C NMR (126 MHz, CDCl$_3$)** δ 146.2 (d, *J* = 31.2 Hz), 139.1 (d, *J* = 6.9 Hz), 137.8 (d, *J* = 12.7 Hz), 137.6 (d, *J* = 12.1 Hz), 137.4 (d, *J* = 12.1 Hz), 133.8, 133.7 (d, *J* = 5.2 Hz), 133.6 (d, *J* = 5.2 Hz), 133.3, 132.2, 130.8 (d, *J* = 5.2 Hz), 128.4 (d, *J* = 13.3 Hz), 128.3 (d, *J* = 6.4 Hz), 128.2 (d, *J* = 6.9 Hz), 128.0 (d, *J* = 8.1 Hz), 127.9 (d, *J* = 3.5 Hz), 127.7 (d, *J* = 11.6 Hz), 126.3, 125.6 (d, *J* = 19.7 Hz), 124.5; **^{31}P NMR (202 MHz, CDCl$_3$)** δ -14.23; **IR (film)**: 3810, 3718, 3661, 2438, 649 cm^{-1}; **HRMS m/z (ESI)**: calcd for $C_{28}H_{22}P$ $[M+H]^+$: 389.1454, found 289.1439.

(4-Methyl-2-(naphthalen-1-yl)phenyl)diphenylphosphane (1b)

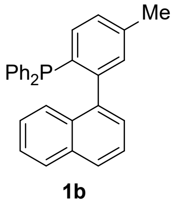

1b

Routes II, with 1.0 mmol scale, **1b** was prepared from commercially available 2-bromo-1-iodo-4-methylbenzene and 1-naphthylboronic acid to obtain a white solid (0.22 g, 54 %, two steps). **^{1}H NMR (500**

MHz, $CDCl_3$) δ 7.85 (d, *J* = 8.2 Hz, 1H), 7.81 (d, *J* = 8.2 Hz, 1H), 7.49 – 7.40 (m, 2H), 7.35 – 7.28 (m, 5H), 7.24 – 7.15 (m, 7H), 7.13 – 7.08 (m, 3H), 7.07 (d, *J* = 7.0 Hz, 1H), 2.40 (s, 3H); **^{13}C NMR (126 MHz, $CDCl_3$)** δ 146.4 (d, *J* = 31.8 Hz), 139.3 (d, *J* = 6.9 Hz), 138.5, 138.0 – 137.9 (m), 134.2 (d, *J* = 11.0 Hz), 134.0, 133.7 (d, *J* = 3.5 Hz), 133.5 (d, *J* = 4.0 Hz), 133.3, 132.3, 131.6 (d, *J* = 5.8 Hz), 128.6, 128.24 (d, *J* = 2.9 Hz), 128.20, 128.10 – 128.05 (m), 127.8 (d, *J* = 3.5 Hz), 127.7, 126.4, 125.6 (d, *J* = 16.8 Hz), 124.6, 21.2; **^{31}P NMR (202 MHz, $CDCl_3$)** δ -15.44; **IR (film):** 3911, 3894, 3791, 3661, 3621, 2347, 676 **cm^{-1}**; **HRMS m/z (ESI)**: calcd for $C_{29}H_{24}P$ $[M+H]^+$: 403.1610, found 403.1601.

(5-Methyl-2-(naphthalen-1-yl)phenyl)diphenylphosphane (1c)

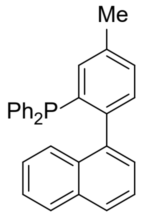

1c

Routes **II**, with 1.0 mmol scale, **1c** was prepared from commercially available 1-bromo-2-iodo-4-methylbenzene and 1-naphthylboronic acid to obtain a white solid (0.21 g, 51 %, two steps). **^{1}H NMR (500 MHz, $CDCl_3$)** δ 7.82 (d, *J* = 8.1 Hz, 1H), 7.78 (d, *J* = 8.2 Hz, 1H), 7.44 (d, *J* = 8.5 Hz, 1H), 7.40 (t, *J* = 7.5 Hz, 1H), 7.30 – 7.25 (m, 5H), 7.23 (s, 1H), 7.23 – 7.20 (m, 2H), 7.19 (d, *J* = 2.1 Hz, 1H), 7.18 – 7.13 (m, 3H), 7.10 (td, *J* = 7.5, 2.1 Hz, 2H), 7.05 (d, *J* = 6.9 Hz, 1H), 7.00 (d, *J* = 4.6 Hz, 1H), 2.31 (s, 3H); **^{13}C NMR (126 MHz, $CDCl_3$)** δ 143.4 (d, *J* = 31.2 Hz), 139.1 (d, *J* = 6.9 Hz), 137.7 – 137.4 (m), 137.2, 134.2, 133.8, 133.6 (d, *J* = 2.3 Hz), 133.3, 132.4, 130.7 (d, J = 5.8 Hz), 129.4, 128.3 – 128.0 (m), 127.6, 126.4, 125.5 (d, *J* = 15.0 Hz), 124.6, 21.4; **^{31}P NMR (202 MHz, $CDCl_3$)** δ -14.10; **IR (film):** 3913, 3894, 3791, 3661, 2347, 676 cm^{-1}; **HRMS m/z (ESI)**: calcd for $C_{29}H_{24}P$ $[M+H]^+$: 403.1610, found 403.1600.

(2-(4-Methylnaphthalen-1-yl)phenyl)diphenylphosphane (1d)

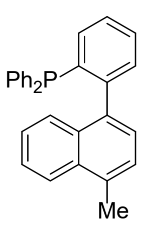

1d

Routes **I**, with 1.0 mmol scale, **1d** was prepared from commercially available 1,2-dibromobenzene and (4-methylnaphthalen-1-yl)boronic acid to obtain a white solid (0.21 g, 52 %, two steps). **^{1}H NMR (500 MHz, $CDCl_3$)** δ 8.02 – 7.93 (m, 1H), 7.49 – 7.42 (m, 2H), 7.38 (t, *J* = 7.4

Hz, 1H), 7.35 – 7.30 (m, 2H), 7.29 – 7.24 (m, 4H), 7.24 – 7.17 (m, 3H), 7.18 – 7.06 (m, 6H), 6.95 (t, *J* = 5.6 Hz, 1H), 2.66 (s, 3H); **^{13}C NMR (126 MHz, $CDCl_3$)** δ 146.6 (d, *J* = 31.8 Hz), 137.7 (d, *J* = 12.7 Hz), 137.6, 137.5 (d, *J* = 7.5 Hz), 133.9, 133.6 (d, *J* = 20.2 Hz), 132.3 (d, *J* = 13.9 Hz), 131.0 (d, *J* = 5.2 Hz), 128.4, 128.3, 128.24, 128.19, 128.13, 128.11, 128.05, 127.6, 126.9, 125.4, 125.3, 124.2, 19.6; **^{31}P NMR (202 MHz, $CDCl_3$)** δ -14.59; **IR (film)**: 3911, 3774, 3661, 2358, 750 cm^{-1}; **HRMS m/z (ESI)**: calcd for $C_{29}H_{24}P$ $[M+H]^+$: 403.1610, found 403.1599.

(4,5-Dimethyl-2-(naphthalen-1-yl)phenyl)diphenylphosphane (1e)

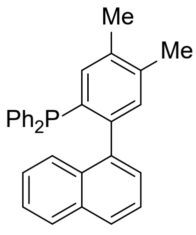

1e

Routes **I**, with 1.0 mmol scale, **1e** was prepared from commercially available 1,2-dibromo-4,5-dimethylbenzene and 1-naphthylboronic acid to obtain a white solid (0.24 g, 57 %, two steps). **^{1}H NMR (500 MHz, $CDCl_3$)** δ 7.82 (d, *J* = 8.1 Hz, 1H), 7.77 (d, *J* = 8.2 Hz, 1H), 7.48 (d, *J* = 8.4 Hz, 1H), 7.43 – 7.37 (m, 1H), 7.32 – 7.26 (m, 5H), 7.23 – 7.19 (m, 2H), 7.18 – 7.14 (m, 3H), 7.13 – 7.09 (m, 3H), 7.07 (dd, *J* = 7.0, 1.2 Hz, 1H), 6.96 (d, *J* = 3.8 Hz, 1H), 2.28 (s, 3H), 2.22 (s, 3H); **^{13}C NMR (126 MHz, $CDCl_3$)** δ 144.2, 144.0, 139.3 (d, *J* = 6.9 Hz), 138.1 – 138.0 (m), 137.3, 136.0, 134.9, 134.2 (d, *J* = 11.6 Hz), 133.6, 133.5, 133.3, 132.5, 132.1 (d, *J* = 6.4 Hz), 128.2 – 128.1 (m), 128.0 (d, *J* = 2.9 Hz), 127.9 (d, *J* = 3.5 Hz), 127.6, 126.5, 125.5 (d, *J* = 12.7 Hz), 124.6, 19.64, 19.59; **^{31}P NMR (202 MHz, $CDCl_3$)** δ -15.29. **IR (film)**: 3845, 3810, 3661, 3578, 2348, 678 cm^{-1}; **HRMS m/z (ESI)**: calcd for $C_{30}H_{26}P$ $[M+H]^+$: 417.1767, found 417.1754.

Diphenyl(2-(4-phenylnaphthalen-1-yl)phenyl)phosphane (1f)

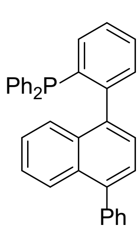

1f

Routes **I**, with 1.0 mmol scale, **1f** was prepared from commercially available 1,2-dibromobenzene and (4-phenylnaphthalen-1-yl)boronic acid to obtain a white solid (0.26 g, 57 %, two steps). **^{1}H NMR (400 MHz, $CDCl_3$)** δ 7.88 (d, *J* = 8.4 Hz, 1H), 7.53 – 7.42 (m, 6H), 7.42 – 7.38 (m, 1H), 7.38 – 7.31 (m, 3H), 7.31 – 7.25 (m, 4H), 7.22 (d, *J* = 2.2 Hz, 1H), 7.21 – 7.17 (m, 3H), 7.17 – 7.13 (m, 3H), 7.13 – 7.09 (m, 2H), 7.07 (d, *J* =

7.2 Hz, 1H); **^{13}C NMR (101 MHz, CDCl$_3$)** δ 146.3 (d, *J* = 30.9 Hz), 140.9, 139.9, 138.6 (d, *J* = 6.4 Hz), 138.0 (d, *J* = 12.7 Hz), 137.5 (d, *J* = 11.8 Hz), 137.4 (d, *J* = 12.3 Hz), 133.9 (d, *J* = 9.1 Hz), 133.7 (d, *J* = 9.1 Hz), 132.5, 131.5, 131.1 (d, *J* = 5.0 Hz), 130.2, 128.5 (d, *J* = 5.0 Hz), 128.3 – 128.1 (m), 127.7, 127.5 (d, *J* = 3.6 Hz), 127.1, 126.7, 126.1, 125.7 (d, *J* = 7.7 Hz), 125.5; **^{31}P NMR (162 MHz, CDCl$_3$)** δ -13.77; **IR (film)**: 3911, 3719, 3639, 2359 cm^{-1}; **HRMS m/z (ESI)**: calcd for $C_{34}H_{26}P$ $[M+H]^+$: 465.1767, found 465.1755.

(4-Methoxy-2-(naphthalen-1-yl)phenyl)diphenylphosphane (1g)

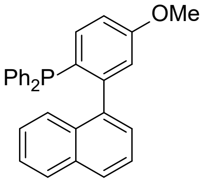

1g

Routes **II**, with 1.0 mmol scale, **1g** was prepared from commercially available 2-bromo-1-iodo-4-methoxybenzene and 1-naphthylboronic acid to obtain a white solid (0.20 g, 48 %, two steps). **^{1}H NMR (400 MHz, CDCl$_3$)** δ 7.81 (dd, *J* = 13.8, 8.2 Hz, 2H), 7.50 (d, *J* = 8.4 Hz, 1H), 7.40 (t, *J* = 7.5 Hz, 1H), 7.33 – 7.22 (m, 5H), 7.22 – 7.13 (m, 5H), 7.13 – 7.03 (m, 4H), 6.91 (d, *J* = 7.9 Hz, 2H), 3.74 (s, 3H); **^{13}C NMR (101 MHz, CDCl$_3$)** δ 159.8, 148.1 (d, *J* = 33.6 Hz), 139.2 (d, *J* = 7.3 Hz), 138.3 (d, *J* = 12.7 Hz), 135.6, 133.4 (d, *J* = 19.1 Hz), 133.2, 132.2, 128.5 (d, *J* = 10.0 Hz), 128.2 – 128.0 (m), 127.8, 127.6 (d, *J* = 3.6 Hz), 126.3, 125.7 (d, *J* = 17.7 Hz), 124.6, 115.9 (d, *J* = 5.9 Hz), 114.1, 55.2; **^{31}P NMR (162 MHz, CDCl$_3$)** δ -16.37; **IR (film)**: 3911, 3739, 3575, 2339, 670 cm^{-1}; **HRMS m/z (ESI)**: calcd for $C_{29}H_{24}OP$ $[M+H]^+$: 419.1559, found 419.1549.

(2-(4-Methoxynaphthalen-1-yl)phenyl)diphenylphosphane (1h)

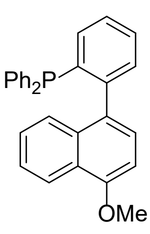

1h

Routes **I**, with 1.0 mmol scale, **1h** was prepared from commercially available 1,2-dibromobenzene and (4-methoxynaphthalen-1-yl)boronic acid to obtain a white solid (0.22 g, 53 %, two steps). **^{1}H NMR (400 MHz, CDCl$_3$)** δ 8.27 (dt, J = 8.4, 1.0 Hz, 1H), 7.41 – 7.34 (m, 3H), 7.33 – 7.25 (m, 6H), 7.23 – 7.16 (m, 3H), 7.16 – 7.12 (m, 3H), 7.12 – 7.10 (m, 1H), 7.10 – 7.06 (m, 1H), 6.97 (d, J = 7.8 Hz, 1H), 6.61 (d, J = 7.9 Hz, 1H), 3.92 (s, 3H); **^{13}C NMR (101 MHz, CDCl$_3$)** δ 155.0, 146.4 (d, *J* = 31.3 Hz), 138.2 (d, *J* =

12.3 Hz), 137.9 (d, J = 12.7 Hz), 137.6 (d, J = 12.3 Hz), 133.9, 133.8 (d, J = 2.7 Hz), 133.6 (d, J = 2.3 Hz), 133.1, 131.4, 131.3 (d, J = 5.0 Hz), 128.4 – 128.1 (m), 127.9 (d, J = 3.6 Hz), 127.5, 126.1 (d, J = 9.1 Hz), 125.3, 124.8, 121.9, 55.3; **^{31}P NMR (162 MHz, $CDCl_3$)** δ -14.32; **IR (film)**: 3991, 3845, 3639, 2359, 749 cm^{-1}; **HRMS m/z (ESI)**: calcd for $C_{29}H_{24}OP$ $[M+H]^+$: 419.1559, found 419.1546.

(6-(Naphthalen-1-yl)benzo[d][1,3]dioxol-5-yl)diphenylphosphane (1i)

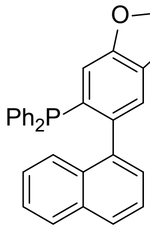

1i

Routes **I**, with 1.0 mmol scale, **1i** was prepared from commercially available 5,6-dibromobenzo[d][1,3]dioxole and 1-naphthylboronic acid to obtain a white solid (0.25 g, 57 %, two steps). **^{1}H NMR (500 MHz, $CDCl_3$)** δ 7.87 (d, J = 8.1 Hz, 1H), 7.83 (d, J = 8.4 Hz, 1H), 7.53 (d, J = 8.4 Hz, 1H), 7.49 – 7.42 (m, 1H), 7.38 – 7.29 (m, 5H), 7.25 – 7.19 (m, 5H), 7.15 – 7.08 (m, 2H), 7.07 (d, J = 7.0 Hz, 1H), 6.85 (d, J = 3.5 Hz, 1H), 6.69 (d, J = 2.6 Hz, 1H), 6.03 (s, 2H); **^{13}C NMR (126 MHz, $CDCl_3$)** δ 148.2, 147.4, 141.2 (d, J = 35.3 Hz), 139.0 (d, J = 8.1 Hz), 138.2 – 138.1 (m), 133.5 – 133.3 (m), 132.5, 130.2 (d, J = 12.1 Hz), 128.3 – 128.1 (m), 127.9, 126.3, 125.7 (d, J = 24.9 Hz), 124.6, 113.3, 111.2 (d, J = 6.9 Hz), 101.3; **^{31}P NMR (202 MHz, $CDCl_3$)** δ -14.39; **IR (film)**: 3861, 3810, 3662, 2348 cm^{-1}; **HRMS m/z (ESI)**: calcd for $C_{29}H_{22}O_2P$ $[M+H]^+$: 433.1352, found 433.1339.

(5-Fluoro-2-(naphthalen-1-yl)phenyl)diphenylphosphane (1j)

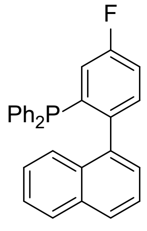

1j

Routes **II**, with 1.0 mmol scale, **1j** was prepared from commercially available 1-bromo-4-fluoro-2-iodobenzene and 1-naphthylboronic acid to obtain a white solid (0.16 g, 39 %, two steps). **^{1}H NMR (500 MHz, $CDCl_3$)** δ 7.84 (d, J = 8.1 Hz, 1H), 7.80 (d, J = 8.2 Hz, 1H), 7.43 (t, J = 7.5 Hz, 1H), 7.38 (d, J = 8.4 Hz, 1H), 7.36 – 7.26 (m, 6H), 7.24 – 7.16 (m, 5H), 7.13 (td, J = 8.4, 2.8 Hz, 1H), 7.10 – 7.06 (m, 2H), 7.02 (d, J = 6.9 Hz, 1H), 6.87 (dt, J = 9.3, 2.9 Hz, 1H); **^{13}C NMR (101 MHz, $CDCl_3$)** δ 162.2 (d, J_{CF}= 248.4 Hz), 141.8 (d, J = 29.5 Hz), 141.2 – 140.1 (m), 137.9 (d, J = 6.5 Hz), 136.9 – 136.4 (m), 133.7 (d, J = 20.0 Hz), 133.3, 132.5, 132.4, 132.4, 132.3, 128.6 (d, J = 10.9 Hz),

128.5 – 128.0 (m), 125.9 (d, J = 23.6 Hz), 125.1 (d, J = 107.6 Hz), 120.0 (d, J = 21.3 Hz), 115.6 (d, J = 21.3 Hz); **^{31}P NMR (162 MHz, CDCl$_3$)** δ -13.52; **^{19}F NMR (376 MHz, CDCl$_3$)** δ -114.11; **IR (film)**: 3911, 3719, 3576, 2352 cm^{-1}; **HRMS m/z (ESI)**: calcd for $C_{28}H_{21}FP$ [M+H]$^+$: 407.1359, found 407.1355.

(4,5-Difluoro-2-(naphthalen-1-yl)phenyl)diphenylphosphane (1k)

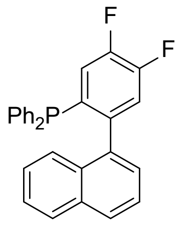

1k

Routes **I**, with 1.0 mmol scale, **1k** was prepared from commercially available 1,2-dibromo-4,5-difluorobenzene and 1-naphthylboronic acid to obtain a white solid (0.19 g, 45 %, two steps). **^{1}H NMR (500 MHz, CDCl$_3$)** δ 7.84 (dd, J = 14.6, 8.2 Hz, 2H), 7.48 – 7.44 (m, 1H), 7.40 (d, J = 8.1 Hz, 1H), 7.36 – 7.31 (m, 4H), 7.29 (dd, J = 8.2, 7.0 Hz, 2H), 7.25 – 7.20 (m, 3H), 7.20 – 7.15 (m, 3H), 7.10 – 7.05 (m, 2H), 7.01 (d, J = 6.6 Hz, 1H), 7.00 – 6.94 (m, 1H); **^{13}C NMR (126 MHz, CDCl$_3$)** δ 150.2 (dd, J_{CF} = 252.2, 12.6 Hz), 149.8 (dd, J_{CF} = 252.0, 12.6 Hz), 143.1 – 142.8 (m), 136.9 (d, J = 5.9 Hz), 136.7 (d, J = 11.9 Hz), 136.6 (d, J = 12.5 Hz), 135.5 – 135.4 (m), 133.3, 131.9, 128.8 (d, J = 9.8 Hz), 128.5 – 128.2 (m), 128.1 (d, J = 4.0 Hz), 126.1, 125.8 (d, J = 5.2 Hz), 124.5, 122.2 (d, J = 16.8 Hz), 119.9 (dd, J = 16.4, 5.0 Hz); **^{31}P NMR (202 MHz, CDCl$_3$)** δ -14.97; **^{19}F NMR (471 MHz, CDCl$_3$)** δ -137.43 (d, J = 21.7 Hz, 1F), -138.62 (d, J = 21.7 Hz, 1F); **IR (film)**: 3810, 3699, 3639, 2358, 668 cm^{-1}; **HRMS m/z (ESI)**: calcd for $C_{28}H_{20}F_2P$ [M+H]$^+$: 425.1265, found 425.1251.

(4-Chloro-2-(naphthalen-1-yl)phenyl)diphenylphosphane (1l)

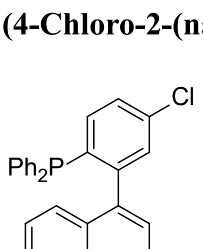

1l

Routes **II**, with 1.0 mmol scale, **1l** was prepared from commercially available 2-bromo-4-chloro-1-iodobenzene and 1-naphthylboronic acid to obtain a white solid (0.12 g, 29 %, two steps). **^{1}H NMR (500 MHz, CDCl$_3$)** δ 7.87 (dd, J = 15.1, 8.1 Hz, 2H), 7.51 – 7.43 (m, 2H), 7.41 – 7.29 (m, 7H), 7.25 – 7.18 (m, 5H), 7.16 (dd, J = 8.5, 3.3 Hz, 1H), 7.12 (t, J = 7.6 Hz, 2H), 7.06 (d, J = 7.0 Hz, 1H); **^{13}C NMR (126 MHz, CDCl$_3$)** δ 147.8 (d, J = 32.4 Hz), 137.7 (d, J = 6.9 Hz), 137.1 – 136.7 (m), 135.2, 134.7, 133.7 (d, J = 6.9 Hz), 133.5 (d, J = 6.9 Hz), 133.3, 131.9, 130.7 (d, J = 5.2 Hz), 128.6 –

127.90, 127.87 , 126.0, 125.7, 124.5; **^{31}P NMR (202 MHz, $CDCl_3$)** δ -15.46; **IR (film)**: 3911, 3758, 3639, 2358 cm^{-1}; **HRMS m/z (ESI)**: calcd for $C_{28}H_{21}ClP$ $[M+H]^+$: 423.1064, found 423.1071.

(2-(4-Chloronaphthalen-1-yl)phenyl)diphenylphosphane (1m)

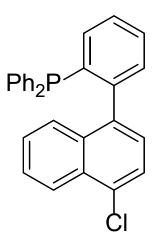

1m

Routes **I**, with 1.0 mmol scale, **1m** was prepared from commercially available 1,2-dibromobenzene and (4-chloronaphthalen-1-yl)boronic acid to obtain a white solid (0.13 g, 31 %, two steps). **^{1}H NMR (500 MHz, $CDCl_3$)** δ 8.37 (d, J = 9.3 Hz, 1H), 7.61 (t, J = 7.8 Hz, 1H), 7.51 (d, J = 7.8 Hz, 1H), 7.48 (d, J = 6.6 Hz, 1H), 7.46 (s, 1H), 7.45 – 7.41 (m, 2H), 7.40 – 7.35 (m, 4H), 7.30 – 7.23 (m, 6H), 7.18 (d, J = 5.2 Hz, 2H), 7.09 – 6.99 (m, 1H); **^{13}C NMR (126 MHz, $CDCl_3$)** δ 145.3 (d, J = 31.2 Hz), 138.4 (d, J = 6.9 Hz), 137.9 (d, J = 13.3 Hz), 137.2 (d, J = 11.6 Hz), 137.0 (d, J = 12.1 Hz), 133.8, 133.7 (d, J = 19.7 Hz), 133.4, 131.5, 130.9 (d, J = 5.2 Hz), 130.5, 128.5 (d, J = 5.8 Hz), 128.4, 128.3, 128.2 (d, J = 6.9 Hz), 128.0, 127.8 (d, J = 4.0 Hz), 126.8 (d, J = 21.4 Hz), 126.4, 124.7 (d, J = 53.8 Hz); **^{31}P NMR (162 MHz, $CDCl_3$)** δ -14.28; **IR (film)**: 3991, 3845, 3639, 2359, 750 cm^{-1}; **HRMS m/z (ESI)**: calcd for $C_{28}H_{21}ClP$ $[M+H]^+$: 423.1064, found 423.1049.

(2-(Naphthalen-1-yl)-4-(trifluoromethyl)phenyl)diphenylphosphane (1n)

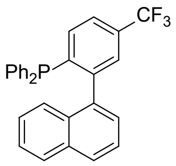

1n

Routes **II**, with 1.0 mmol scale, **1n** was prepared from commercially available 2-bromo-1-iodo-4-(trifluoromethyl)benzene and 1-naphthylboronic acid to obtain a white solid (0.16 g, 36 %, two steps). **^{1}H NMR (500 MHz, $CDCl_3$)** δ 7.87 (dd, J = 12.4, 8.1 Hz, 2H), 7.64 (d, J = 7.9 Hz, 2H), 7.48 (t, J = 7.2 Hz, 1H), 7.42 – 7.31 (m, 7H), 7.25 – 7.17 (m, 5H), 7.13 (t, J = 7.6 Hz, 2H), 7.07 (d, J = 7.0 Hz, 1H); **^{13}C NMR (126 MHz, $CDCl_3$)** δ 146.6 (d, J = 30.6 Hz), 143.5 (d, J = 17.3 Hz), 137.5 (d, J = 6.4 Hz), 136.4 (d, J = 12.1 Hz), 136.1 (d, J = 12.1 Hz), 134.0 – 133.7 (m), 133.3, 131.8, 130.5 (q, J_{CF3} = 32.4 Hz), 128.8 (d, J = 23.1 Hz), 128.5 (d, J = 7.5 Hz), 128.4 – 128.1 (m), 127.43 – 127.37 (m), 126.1, 125.8 (d, J = 4.0 Hz), 124.6, 124.2 (q, J_{CF3} = 4.0 Hz), 124.0 (q,

J_{CF3} = 272.2 Hz); **^{31}P NMR (202 MHz, CDCl$_3$)** δ -13.93; **^{19}F NMR (471 MHz, CDCl$_3$)** δ -62.58; **IR (film)**: 3889, 3758, 3638, 2358 cm^{-1}; **HRMS m/z (ESI)**: calcd for $C_{29}H_{21}F_3P$ [M+H]$^+$: 457.1327, found 457.1315.

(2-(Naphthalen-1-yl)-5-(trifluoromethyl)phenyl)diphenylphosphane (1o)

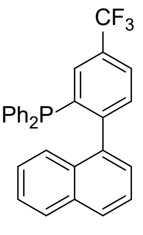

1o

Routes **II**, with 1.0 mmol scale, **1o** was prepared from commercially available 1-bromo-2-iodo-4-(trifluoromethyl)benzene and 1-naphthylboronic acid to obtain a white solid (0.19 g, 41 %, two steps). **^{1}H NMR (500 MHz, CDCl$_3$)** δ 7.87 (dd, J = 11.8, 8.0 Hz, 2H), 7.72 (d, J = 8.4 Hz, 1H), 7.52 – 7.42 (m, 3H), 7.41 – 7.29 (m, 6H), 7.25 – 7.15 (m, 5H), 7.12 (t, J = 7.7 Hz, 2H), 7.06 (d, J = 6.9 Hz, 1H); **^{13}C NMR (126 MHz, CDCl$_3$)** δ 149.7 (d, *J* = 30.1 Hz), 140.1 (d, *J* = 17.3 Hz), 137.6 (d, *J* = 6.4 Hz), 136.5 (d, *J* = 11.6 Hz), 136.0 (d, *J* = 12.1 Hz), 133.9 – 133.6 (m), 133.3, 131.7, 131.3 (d, *J* = 4.6 Hz), 130.1 (d, *J* = 4.0 Hz), 129.9 (q, J_{CF3} = 32.1 Hz), 128.9 – 128.2 (m), 127.9 (d, *J* = 3.5 Hz), 126.0, 125.8 (d, *J* = 8.1 Hz), 125.2 (q, J_{CF3} = 3.8 Hz), 124.1 (q, J_{CF3} = 272.8 Hz), 124.5; **^{31}P NMR (202 MHz, CDCl$_3$)** δ -13.68; **^{19}F NMR (471 MHz, CDCl$_3$)** δ -62.41; **IR (film)**: 3911, 3719, 3640, 2359, 750 cm^{-1}; **HRMS m/z (ESI)**: calcd for $C_{29}H_{21}F_3P$ [M+H]$^+$: 457.1327, found 457.1316.

(2-(1,2-Dihydroacenaphthylen-5-yl)phenyl)diphenylphosphane (1p)

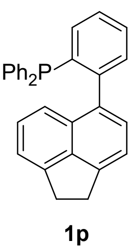

1p

Routes **I**, with 1.0 mmol scale, **1p** was prepared from commercially available 1,2-dibromobenzene and (1,2-dihydroacenaphthylen-5-yl)boronic acid to obtain a white solid (0.18 g, 44 %, two steps). **^{1}H NMR (500 MHz, CDCl$_3$)** δ 7.41 (m, 1H), 7.38 – 7.34 (m, 1H), 7.34 – 7.31 (m, 1H), 7.31 – 7.27 (m, 4H), 7.25 – 7.21 (m, 3H), 7.20 (d, *J* = 8.3 Hz, 2H), 7.18 – 7.15 (m, 3H), 7.13 – 7.08 (m, 3H), 7.02 (d, *J* = 7.0 Hz, 1H), 3.43 – 3.33 (m, 4H); **^{13}C NMR (126 MHz, CDCl$_3$)** δ 146.1 (d, *J* = 31.2 Hz), 145.8 (d, *J* = 24.3 Hz), 139.1, 137.9 (d, *J* = 3.5 Hz), 137.8 (d, *J* = 3.5 Hz), 137.5 (d, *J* = 12.1 Hz), 134.6 (d, *J* = 7.5 Hz), 134.1, 133.7 (d, *J* = 12.7 Hz), 133.6 (d, *J* = 13.3 Hz), 131.0 (d, *J* = 5.2 Hz), 130.5, 129.6 (d, *J* = 4.0 Hz), 128.5, 128.2 (d, *J* = 28.3

Hz), 128.1 (d, J = 27.2 Hz), 127.6 (d, J = 25.4 Hz), 121.2, 118.6 (d, J = 130.0 Hz), 30.5, 30.1; **^{31}P NMR (202 MHz, CDCl$_3$)** δ -14.46; **IR (film)**: 3911, 3682, 2359, 750 cm^{-1}; **HRMS m/z (ESI)**: calcd for $C_{30}H_{24}P$ [M+H]$^+$: 415.1610, found 415.1595.

(2-(Phenanthren-9-yl)phenyl)diphenylphosphane (1q)

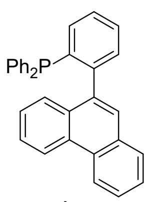

1q

Routes **I**, with 1.0 mmol scale, **1q** was prepared from commercially available 1,2-dibromobenzene and phenanthren-9-ylboronic acid to obtain a white solid (0.22 g, 51 %, two steps). **^{1}H NMR (500 MHz, CDCl$_3$)** δ 8.08 (d, J = 8.1 Hz, 1H), 7.44 – 7.38 (m, 2H), 7.36 (td, J = 7.3, 1.5 Hz, 1H), 7.33 – 7.26 (m, 4H), 7.26 – 7.21 (m, 4H), 7.21 – 7.16 (m, 3H), 7.15 – 7.11 (m, 2H), 7.11 – 7.04 (m, 4H), 6.99 – 6.88 (m, 2H); **^{13}C NMR (126 MHz, CDCl$_3$)** δ 159.3, 157.3, 145.4 (d, J = 30.6 Hz), 138.1 (d, J = 12.7 Hz), 137.3 (d, J = 12.1 Hz), 137.1 (d, J = 12.1 Hz), 135.1 – 135.0 (m), 133.8 (d, J = 3.5 Hz), 133.7, 133.6 (d, J = 3.5 Hz), 133.5 (d, J = 4.6 Hz), 131.0 (d, J = 5.2 Hz), 128.46 (d, J = 4.0 Hz), 128.32, 128.27, 128.1 (d, J = 6.9 Hz), 127.8, 127.7 – 127.6 (m), 126.6, 126.3, 125.8 (d, J = 2.3 Hz), 123.4 (d, J = 16.2 Hz), 120.5 (d, J = 5.2 Hz), 108.1 (d, J = 19.7 Hz); **^{31}P NMR (202 MHz, CDCl$_3$)** δ -13.98; **IR (film)**: 3911, 3774, 3639, 2359, 750 cm^{-1}; **HRMS m/z (ESI)**: calcd for $C_{32}H_{24}P$ [M+H]$^+$: 439.1610, found 439.1595.

Diphenyl(2-(pyren-1-yl)phenyl)phosphane (1r)

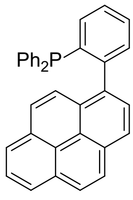

1r

Routes **I**, with 1.0 mmol scale, **1r** was prepared from commercially available 1,2-dibromobenzene and 1-pyreneboronic acid to obtain a white solid (0.19 g, 42 %, two steps). **^{1}H NMR (500 MHz, CDCl$_3$)** δ 8.55 – 8.06 (m, 4H), 8.05 – 7.90 (m, 3H), 7.90 – 7.81 (m, 1H), 7.75 – 7.60 (m, 2H), 7.59 – 7.37 (m, 4H), 7.37 – 7.24 (m, 4H), 7.22 – 7.03 (m, 5H); **^{13}C NMR (126 MHz, CDCl$_3$)** δ 146.7 (d, J = 30.6 Hz), 138.1 (d, J = 12.1 Hz), 137.4 – 136.8 (m), 133.9, 133.8, 133.7, 131.3 (d, J = 4.6 Hz), 131.2, 130.8 (d, J = 25.4 Hz), 129.1 (d, J = 20.8 Hz), 128.5, 128.4, 128.32, 128.29, 128.26, 128.2 (d, J = 3.5 Hz), 128.1, 127.8, 127.4, 127.2 (d, J = 20.2 Hz), 125.7 (d, J = 32.9 Hz), 124.9 (d, J = 13.9 Hz), 124.6 (d, J = 26.6 Hz), 123.7; **^{31}P NMR (202 MHz, CDCl$_3$)** δ -14.19; **IR (film)**: 3911, 3682,

3639, 2360, 751 cm^{-1}; **HRMS m/z (ESI)**: calcd for $C_{34}H_{24}P$ $[M+H]^+$: 463.1610, found 463.1597.

[1,2'-Binaphthalen]-3'-yldiphenylphosphane (1s)

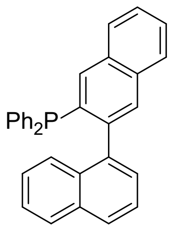

1s

Routes **I**, with 1.0 mmol scale, **1s** was prepared from commercially available 2,3-dibromonaphthalene and 1-naphthylboronic acid to obtain a white solid (0.26 g, 60 %, two steps). **^{1}H NMR (500 MHz, $CDCl_3$)** δ 7.90 – 7.81 (m, 4H), 7.76 (d, *J* = 8.1 Hz, 1H), 7.64 (d, *J* = 3.8 Hz, 1H), 7.55 (t, *J* = 7.4 Hz, 1H), 7.51 (t, *J* = 7.3 Hz, 1H), 7.45 (t, *J* = 8.4 Hz, 2H), 7.39 – 7.26 (m, 6H), 7.23 (t, *J* = 7.9 Hz, 4H), 7.16 (t, *J* = 7.7 Hz, 2H), 7.10 (d, *J* = 6.9 Hz, 1H); **^{13}C NMR (126 MHz, $CDCl_3$)** δ 142.4 (d, *J* = 29.5 Hz), 138.6 (d, *J* = 5.8 Hz), 137.2 (d, *J* = 11.6 Hz), 137.0 (d, *J* = 13.9 Hz), 136.8 (d, *J* = 12.1 Hz), 134.1 – 133.8 (m), 133.2 (d, *J* = 8.7 Hz), 132.63, 132.58, 129.5 (d, *J* = 5.2 Hz), 128.6, 128.39, 128.36, 128.3 (d, *J* = 7.5 Hz), 128.2 (d, *J* = 6.9 Hz), 128.0 (d, *J* = 3.5 Hz), 127.8, 127.6, 126.9, 126.5, 126.1, 125.6 (d, *J* = 20.2 Hz), 124.5; **^{31}P NMR (202 MHz, $CDCl_3$)** δ -13.07; **IR (film)**: 3911, 3682, 3639, 2358, 750 cm^{-1}; **HRMS m/z (ESI)**: calcd for $C_{32}H_{24}P$ $[M+H]^+$: 439.1610, found 439.1596.

4-(2-(Diphenylphosphanyl)phenyl)quinoline (1t)

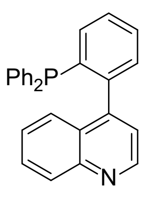

1t

Routes **I**, with 1.0 mmol scale, **1t** was prepared from commercially available 1,2-dibromobenzene and 4-quinolinyl boronic acid to obtain a white solid (0.13 g, 33 %, two steps). **^{1}H NMR (500 MHz, $CDCl_3$)** δ 8.75 (d, *J* = 4.5 Hz, 1H), 8.12 (d, *J* = 8.4 Hz, 1H), 7.66 (t, *J* = 7.6 Hz, 1H), 7.49 – 7.44 (m, 2H), 7.41 (t, *J* = 7.2 Hz, 1H), 7.37 (d, *J* = 7.5 Hz, 1H), 7.34 – 7.27 (m, 4H), 7.24 – 7.22 (m, 1H), 7.20 (t, *J* = 7.4 Hz, 5H), 7.13 – 7.08 (m, 2H), 7.00 (d, *J* = 4.4 Hz, 1H); **^{13}C NMR (126 MHz, $CDCl_3$)** δ 149.2, 148.2, 147.6 (d, *J* = 6.4 Hz), 143.3 (d, *J* = 30.6 Hz), 137.3 (d, *J* = 15.0 Hz), 136.8 (d, *J* = 11.6 Hz), 136.4 (d, *J* = 11.6 Hz), 133.84 , 133.79 , 133.7, 130.1 (d, *J* = 5.2 Hz), 129.3 (d, *J* = 51.4 Hz), 128.6 (d, *J* = 14.5 Hz), 128.5 (d, *J* = 4.0 Hz), 128.4, 128.2 (d, *J* = 6.9 Hz), 127.4, 126.2 (d, *J* = 7.5 Hz), 122.6 (d, *J* = 3.5 Hz); **^{31}P NMR (202 MHz, $CDCl_3$)** δ

-13.98; **IR (film)**: 3911, 3682, 3640, 2359, 750 cm^{-1}; **HRMS m/z (ESI)**: calcd for $C_{27}H_{21}NP$ [M+H]$^+$: 390.1406, found 390.1393.

(2-(Dibenzo[b,d]furan-1-yl)phenyl)diphenylphosphane (1u)

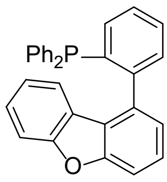

1u

Routes **I**, with 1.0 mmol scale, **1u** was prepared from commercially available 1,2-dibromobenzene and dibenzo[b,d]furan-1-ylboronic acid to obtain a white solid (0.16 g, 38 %, two steps). **^{1}H NMR (400 MHz, CDCl$_3$)** δ 7.52 – 7.43 (m, 3H), 7.43 – 7.37 (m, 2H), 7.37 – 7.32 (m, 1H), 7.30 – 7.27 (m, 3H), 7.25 – 7.20 (m, 1H), 7.20 – 7.14 (m, 3H), 7.09 – 7.04 (m, 2H), 7.03 – 6.99 (m, 4H), 6.93 (d, J = 7.5 Hz, 1H), 6.87 (d, J = 7.4 Hz, 1H); **^{13}C NMR (101 MHz, CDCl$_3$)** δ 156.0 (d, J = 36.3 Hz), 144.9 (d, J = 28.6 Hz), 137.4 (d, J = 14.1 Hz), 136.9 (d, J = 11.8 Hz), 136.7 (d, J = 11.8 Hz), 136.5 (d, J = 6.8 Hz), 134.0 (d, J = 13.2 Hz), 133.8 (d, J = 13.6 Hz), 133.6, 129.9 (d, J = 5.0 Hz), 128.8, 128.5, 128.3 (d, J = 6.8 Hz), 128.2 (d, J = 6.8 Hz), 128.0 (d, J = 6.8 Hz), 126.7, 126.1, 124.6 (d, J = 3.6 Hz), 122.2 (d, J = 2.7 Hz), 111.3, 110.5; **^{31}P NMR (162 MHz, CDCl$_3$)** δ -12.81; **IR (film)**: 3911, 3774, 3640, 2360, 750 cm^{-1}; **HRMS m/z (ESI)**: calcd for $C_{30}H_{22}OP$ [M+H]$^+$: 429.1403, found 429.1385.

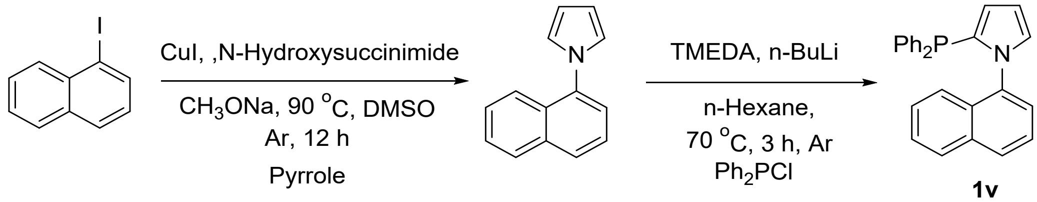

2-(Diphenylphosphanyl)-1-(naphthalen-1-yl)-1H-pyrrole (1v)

Step I:[4] To a solution of CH_3ONa (3.0 mmol) in DMSO (3 mL) was added CuI (19 mg, 0.1 mmol), and N-Hydroxysuccinimide (0.2 mmol). After stirring at room temperature for 30 minutes, a mixture of 1-iodonaphthalene (2.0 mmol) and pyrrole (2.0 mmol) in 2 mL DMSO were added to the flask. The flask was immersed in an oil bath, and the reaction mixture was stirred at the 70 °C for the corresponding reaction time. The mixture was cooled to room temperature, 10 mL water was added, and the resulting suspension was extracted with ethyl acetate (10 mL×4). The extraction was

concentrated, and purified by column chromatography on silica gel (PE/DCM = 10/1) to provide the desired product 1-(naphthalen-1-yl)-1H-pyrrole. Step II: In a three nacked 50 ml round bottom flask with reflux condenser 1-(naphthalen-1-yl)-1H-pyrrole (1 mmol) was dissolved in 2 ml of freshly distilled n-hexane under argon. TMEDA (1.5 mmol) was added followed by n-BuLi (1 mmol, 2.5 M in hexane) at room temperature. The reaction mixture was refluxed for 3 h to obtain a yellow suspension. A solution of the dichlorophenylphosphine (1 mmol in 0.5 ml hexane) was slowly added via syringe. The mixture was further refluxed for 1h. After cooling to room temperature, degassed water (5 ml) was added and the mixture was stirred to get a clear solution. The aqueous layer was extracted with hexane (2x 15 ml) and the combined organic layers were washed with degassed water (15 ml). The solution was dried over Na_2SO_4 and concentrated at 45 °C to get a (0.2 g, 53%) yellow solid which was recrystallized from methanol. **^{1}H NMR (400 MHz, CDCl$_3$)** δ 7.85 (d, *J* = 8.3 Hz, 2H), 7.46 (t, *J* = 7.5 Hz, 1H), 7.39 – 7.31 (m, 3H), 7.30 (d, *J* = 3.5 Hz, 5H), 7.24 (d, *J* = 7.0 Hz, 2H), 7.22 – 7.19 (m, 4H), 7.12 – 7.06 (m, 1H), 6.47 – 6.40 (m, 1H), 6.26 – 6.19 (m, 1H); **^{13}C NMR (101 MHz, CDCl$_3$)** δ 137.6 (d, *J* = 8.2 Hz), 137.4 (d, *J* = 6.4 Hz), 137.0 (d, *J* = 2.7 Hz), 133.8, 133.4 – 133.2 (m), 131.2 (d, *J* = 45.9 Hz), 128.8, 128.4, – 127.9 (m), 126.7 (d, *J* = 48.6 Hz), 126.0 (d, *J* = 3.2 Hz), 124.7, 123.3, 118.5, 109.5; **^{31}P NMR (162 MHz, CDCl$_3$)** δ -30.82; **IR (film)**: 3877, 3774, 3640, 2360, 2340, 750 cm^{-1}; **HRMS m/z (ESI)**: calcd for $C_{26}H_{21}NP$ [M+H]$^+$: 378.1406, found 378.1395.

Bis(4-fluorophenyl)(2-(naphthalen-1-yl)phenyl)phosphane (1w)

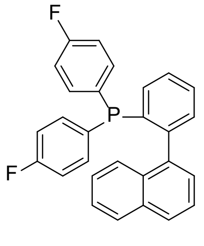

1w

Routes **I**, with 1.0 mmol scale, **1x** was prepared from commercially available 1,2-dibromobenzene, 1-naphthylboronic acid and chlorobis(4-fluorophenyl)phosphane to obtain a white solid (0.16 g, 38 %, two steps). **^{1}H NMR (400 MHz, CDCl$_3$)** δ 7.81 (dd, *J* = 11.4, 7.8 Hz, 2H), 7.48 – 7.38 (m, 2H), 7.36 (dd, *J* = 7.5, 1.7 Hz, 1H), 7.34 – 7.28 (m, 3H), 7.28 – 7.22 (m, 1H), 7.18 – 7.10 (m, 3H), 7.07 – 6.96 (m, 5H), 6.84 (t, *J* = 8.9 Hz, 2H); **^{13}C NMR (101 MHz, CDCl$_3$)** δ 163.2 (d, J_{CF} = 249.5 Hz), 163.1 (d,

J_{CF} = 250.5 Hz), 146.0 (d, J = 30.0 Hz), 138.8 (d, J = 6.4 Hz), 137.8 (d, J = 12.7 Hz), 135.7 (dd, J = 8.2, 2.7 Hz), 135.5 (dd, J = 7.7, 2.3 Hz), 133.3, 133.2, 132.9 (dd, J = 12.3, 3.6 Hz), 132.4 (dd, J = 12.5, 3.4 Hz), 132.1 (d, J = 1.1 Hz), 131.0 (d, J = 5.0 Hz), 128.7, 128.1– 127.8 (m), 126.2, 125.7 (d, J = 10.9 Hz), 124.6, 115.6 (dd, J = 22.2, 7.1 Hz), 115.4 (dd, J = 20.2, 8.1 Hz); **^{31}P NMR (162 MHz, CDCl$_3$)** δ -16.21 (t, J = 4.9 Hz, 1P); **^{19}F NMR (376 MHz, CDCl$_3$)** δ -112.54 (s, 1F) , -112.81(s, 1F); **IR (film)**: 3911, 3661, 3622, 2360, 1275 cm^{-1}; **HRMS m/z (ESI)**: calcd for $C_{28}H_{20}F_2P$ [M+H]$^+$: 425.1265, found 425.1249.

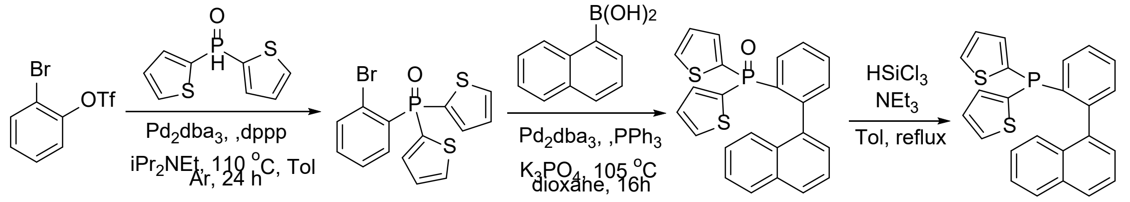

1x

(2-(Naphthalen-1-yl)phenyl)di(thiophen-2-yl)phosphane (1x)

According to the literature [5], Step 1: a dry Schlenk flask containing a magnetic rod was charged with the aryl bromide (2 mmol), di(thiophen-2-yl)phosphine oxide (1.1 equiv.), $Pd_2(dba)_3$ (2.5 mol%), dppp (6 mol%). The Schlenk flask was evacuated and backfilled with nitrogen thrice, then iPr_2NEt (1.5 equiv.) and dry toluene (6 mL) were injected. The reaction mixture was heated at 120 °C in an oil bath for 24 h. After cooling to room temperature, the mixture was filtrated through a short plug of celite and washed with dichloromethane. The filtrate was concentrated under vacuum, and the residue was purified by silica gel flash column chromatography with hexane/ethyl acetate (1:1) to afford the product (2-bromophenyl)di(thiophen-2-yl)phosphine oxide. Step 2: A two-necked flask was charged with (2-bromophenyl)di(thiophen-2-yl)phosphine oxide (0.35 g, 1 mmol) and 1-naphthylboronic acid (0.17 g, 1 mmol), together with $Pd_2(dba)_3$ (9.2 mg, 0.01 mmol), PPh_3 (10.5 mg, 0.04 mmol) and K_3PO_4 (0.484 g, 2 mmol) in 4 mL of dioxane

under a nitrogen atmosphere. The reaction mixture was stirred at 105 °C for 16 h and cooled to room temperature. The mixture was diluted with water (5mL) and extracted with $CHCl_3$ (3 × 5 mL). The combined organic extracts were washed with brine, dried over $MgSO_4$, and evaporated in vacuo. After flash chromatography (EtOAc/hexane = 2/1), (2-(naphthalen-1-yl)phenyl)di(thiophen-2-yl)phosphine oxide as a white solid was obtained. Step 3: A solution of (2-(naphthalen-1-yl)phenyl)di(thiophen-2-yl)phosphine oxide (0.29 g, 0.7 mmol) in toluene (4 mL) was frozen using an EtOH/liquid nitrogen bath, to which trichlorosilane (0.35 mL, 3.5 mmol) and triethylamine (0.53 mL, 3.8 mmol) were added. The mixture was stirred at 110 °C under nitrogen overnight. After cooling to room temperature, a saturated aqueous solution of $NaHCO_3$ (5 mL) was added, and the mixture was stirred for 5 min. The mixture was filtered through a pad of alumina and evaporated in vacuo to give the crude product. Purification by flash chromatography (PE/EA = 10/1) gave **1x** as a white solid (0.22 g, 27 %, three steps). **^{1}H NMR (500 MHz, $CDCl_3$)** δ 8.00 – 7.93 (m, 2H), 7.65 (dd, *J* = 5.0, 1.3 Hz, 1H), 7.64 – 7.60 (m, 1H), 7.59 – 7.52 (m, 4H), 7.52 – 7.48 (m, 1H), 7.47 (dd, *J* = 4.9, 1.2 Hz, 1H), 7.46 – 7.38 (m, 2H), 7.31 (d, J = 6.9 Hz, 1H), 7.24 (ddd, *J* = 6.0, 3.5, 1.2 Hz, 1H), 7.18 – 7.13 (m, 1H), 7.07 (ddd, *J* = 6.0, 3.5, 1.2 Hz, 1H), 6.97 – 6.92 (m, 1H); **^{13}C NMR (126 MHz, $CDCl_3$)** δ 144.8 (d, *J* = 30.1 Hz), 138.9 (d, *J* = 8.6 Hz), 138.6 – 138.4 (m), 137.7 (d, *J* = 25.4 Hz), 135.7 – 135.3 (m), 133.2, 132.0, 131.9 (d, *J* = 1.7 Hz), 131.5 (d, J = 30.6 Hz), 130.8 (d, *J* = 4.6 Hz), 128.7, 127.9 – 127.5, 126.1 (d, *J* = 1.2 Hz), 125.6, 125.5, 124.7; **^{31}P NMR (162 MHz, $CDCl_3$)** δ -42.17; **IR (film)**: 3911, 3758, 3576, 2360, 1277 cm^{-1}; **HRMS m/z (ESI)**: calcd for $C_{24}H_{18}PS_2$ $[M+H]^+$: 401.0582, found 401.0568.

2.2 Optimization of the reaction conditions

Supplementary Table 1. Optimization of reaction conditions for the hydroarylation of 2a. [a]

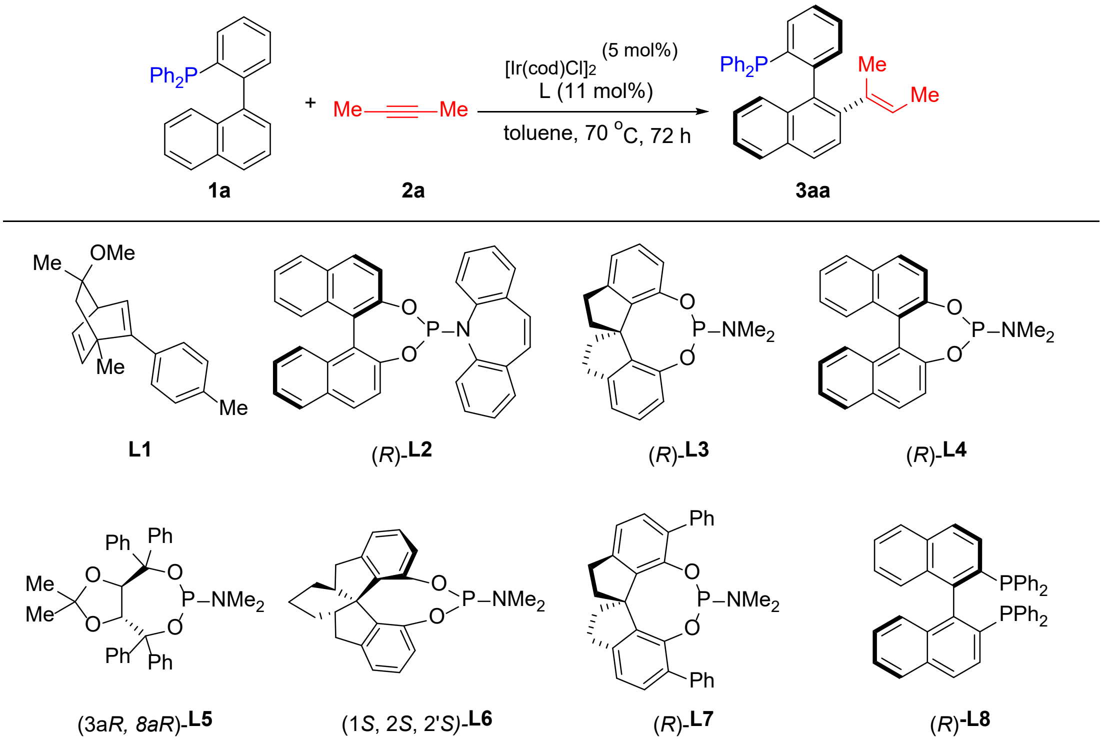

entry	L	yield of **3aa** (%)[b]	ee of **3aa** (%)[c]
1	L1	33	0
2	L2	79	-51
3	L3	82	97
4	L4	76	-66
5	L5	trace	0
6	L6	75	-97
7	L7	57	7
8	L8	77	0
9	-	42	0

[a] Reaction conditions: $[Ir(cod)Cl]_2$ (0.01 mmol) and L (0.022 mmol) in toluene (2.0 ml) at r.t. for 1h, then **1a** (0.2 mmol), **2a** (1.0 mmol) was added and stirring at 70 °C for 72h. [b] Isolated yield of **3aa**. [c] Determined by HPLC analysis.

Supplementary Table 2. Effect of catalyst for the hydroarylation of 2a.[a]

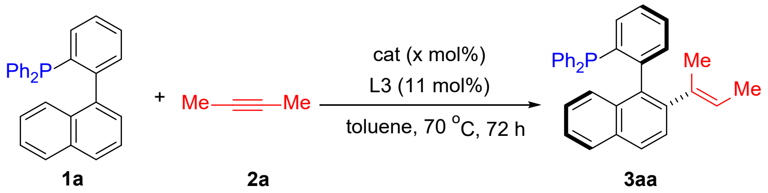

entry	cat (x mol%)	yield of **3aa** (%)[b]	ee of **3aa** (%)[c]
1	$[Ir(cod)Cl]_2$ (5 mol%)	82	97
2	$[Ir(coe)_2Cl]_2$ (5 mol%)	77	77
3	$[Ir(cod)OMe]_2$ (5 mol%)	9	15
4	$Ir(cod)BF_4$ (10 mol%)	23	61
5	$[Rh(cod)Cl]_2$ (5 mol%)	70	12
6	Pd(OAc) (5 mol%)	0	-
7	-	0	-

[a] Reaction conditions: catalyst (x mmol) and **L3** (0.022 mmol) in toluene (2.0 ml) at r.t. for 1h, then **1a** (0.2 mmol), **2a** (1.0 mmol) was added and stirring at 70 ºC for 72h. [b] Isolated yield of **3aa**. [c] Determined by HPLC analysis.

Supplementary Table 3. Effect of solvent for the hydroarylation of 2a. [a]

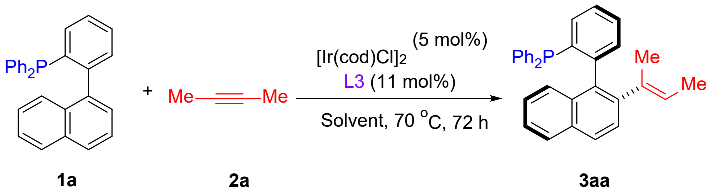

entry	Solvent	yield of **3aa** (%)[b]	ee of **3aa** (%)[c]
1	Toluene	82	97
2	THF	44	71
3	DCM	23	83
4	n-Hexane	N.R.	-
5	MeOH	N.R.	-
6	MeCN	N.R.	-
7	DMF	9	31

[a] Reaction conditions: $[Ir(cod)Cl]_2$ (0.01 mmol) and **L3** (0.022 mmol) in solvent (2.0 ml) at r.t. for 1h, then **1a** (0.2 mmol), **2a** (1.0 mmol) was added and stirring at 70 ºC for 72h. [b] Isolated yield of **3aa**. [c] Determined by HPLC analysis.

Supplementary Table 4. Optimization of reaction conditions for the hydroarylation of 4a. [a]

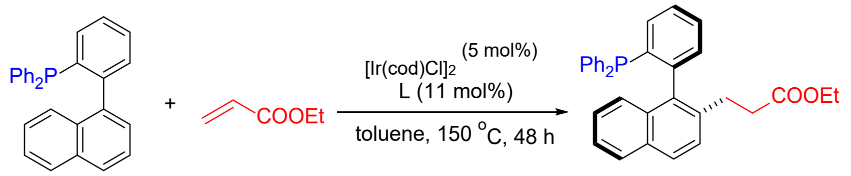

1a **4a** **5aa**

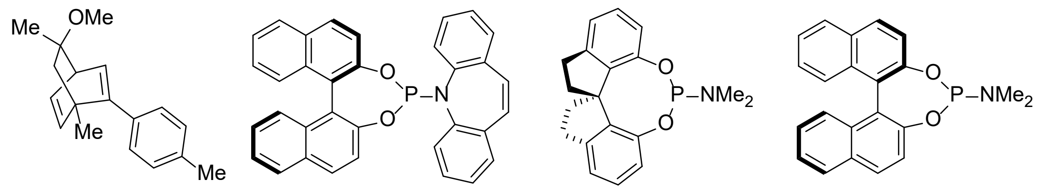

L1 (*R*)-**L2** (*R*)-**L3** (*R*)-**L4**

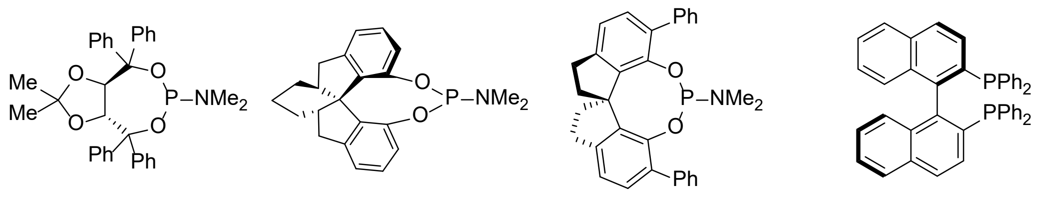

(3a*R*, 8a*R*)-**L5** (1*S*, 2*S*, 2'*S*)-**L6** (*R*)-**L7** (*R*)-**L8**

entry	L	yield of **5aa** (%)[b]	ee of **5aa** (%)[c]
1	L1	20	0
2	L2	69	15
3	L3	71	-78
4	L4	77	91
5	L5	4	3
6	L6	65	77
7	L7	50	27
8	L8	38	25

[a] Reaction conditions: $[Ir(cod)Cl]_2$ (0.01 mmol) and L (0.022 mmol) in toluene (2.0 ml) at r.t. for 1h, then **1a** (0.2 mmol), **4a** (1.0 mmol) was added and stirring at 150 °C for 48h. [b] Isolated yield of **5aa**. [c] Determined by HPLC analysis.

Supplementary Table 5. Effect of catalyst for the hydroarylation of 4a.[a]

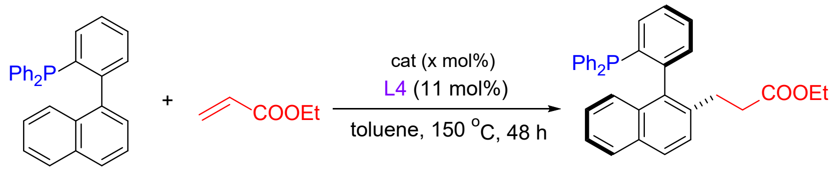

entry	cat (x mol%)	yield of **5aa** (%)[b]	ee of **5aa** (%)[c]
1	$[Ir(cod)Cl]_2$ (5 mol%)	77	91
2	$[Ir(coe)_2Cl]_2$ (5 mol%)	69	47
3	$[Ir(cod)OMe]_2$ (5 mol%)	15	9
4	$Ir(cod)BF_4$ (10 mol%)	37	35
5	$[Rh(cod)Cl]_2$ (5 mol%)	N.R.	-
6	$Pd(OAc)_2$ (10 mol%)	N.R.	-

[a] Reaction conditions: catalyst (x mmol) and **L4** (0.022 mmol) in toluene (2.0 ml) at r.t. for 1h, then **1a** (0.2 mmol), **4a** (1.0 mmol) was added and stirring at 150 °C for 48h. [b] Isolated yield of **5aa**. [c] Determined by HPLC analysis.

Supplementary Table 6. Effect of solvent for the hydroarylation of 4a. [a]

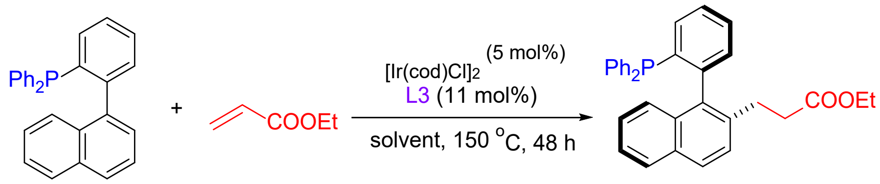

Entry	Solvent	yield of **5aa** (%)[b]	ee of **5aa** (%)[c]
1	Toluene	77	91
2	1,4-dioxane	36	70
3	DCE	44	59
4	n-Hexane	N.R.	-
5	EtOH	N.R.	-
6	MeCN	N.R.	-
7	DMF	N.R.	-

[a] Reaction conditions: $[Ir(cod)Cl]_2$ (0.01 mmol) and **L4** (0.022 mmol) in solvent (2.0 ml) at r.t. for 1h, then **1a** (0.2 mmol), **4a** (1.0 mmol) was added and stirring at 150 °C for 48h. [b] Isolated yield of **5aa**. [c] Determined by HPLC analysis.

2.3 Experimental Procedures and Characterization of Products

2.3.1 asymmetric C–H hydroarylation with alkynes

General Procedure A:

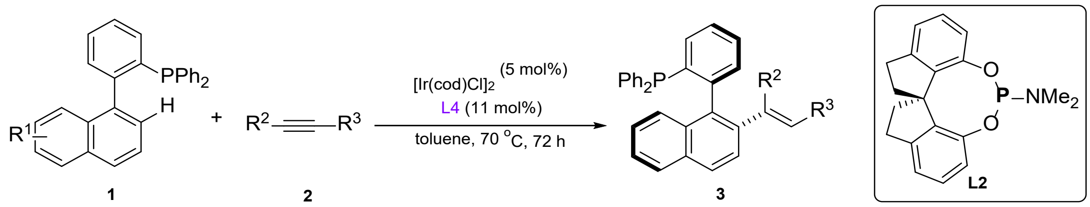

A 25.0 mL dry Schlenk tube with a stirring bar was added with $[Ir(cod)Cl]_2$ (3.4 mg, 0.01 mmol, 0.05 equiv) and **L3** (7.3 mg, 0.022 mmol, 0.11 equiv) in 2.0 ml toluene under Ar. The formed mixture was stirred at room temperature for 1h. Then **1** (0.2 mmol, 1 equiv) and **2** (1.0 mmol, 5 equiv) was added under argon, the resulting mixture was stirred at 70 ºC. After 72 h, the solvent was removed under vaccum. The crude product purified by column chromatography on silica gel (300-400 mesh, PE/DCM or PE/EtOAc as eluent) to afford the corresponding product **3**.

Synthetic route of Racemic **3**

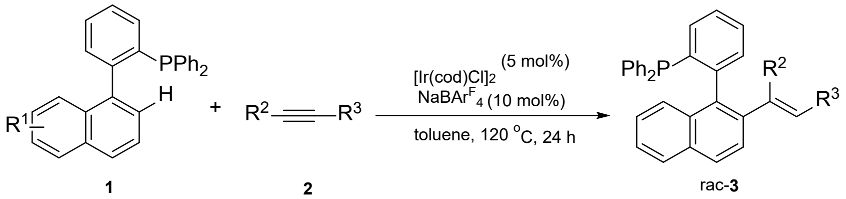

A 25.0 mL dry Schlenk tube with a stirring bar was added with $[Ir(cod)Cl]_2$ (3.4 mg, 0.01 mmol, 0.05 equiv) and $NaBAr^F_4$ (17.7 mg, 0.02 mmol, 0.11 equiv) in 2.0 ml toluene under Ar. The formed mixture was stirred at room temperature for 1h. Then **1** (0.2 mmol, 1 equiv) and **2** (1.0 mmol, 5 equiv) was added under argon, the resulting mixture was stirred at 120 ºC. After 24 h, the solvent was removed under vaccum. The crude product purified by column chromatography on silica gel (300-400 mesh, PE/DCM or PE/EtOAc as eluent) to afford the corresponding racemic product **3**.

(R,E)-(2-(2-(But-2-en-2-yl)naphthalen-1-yl)phenyl)diphenylphosphane (3aa)

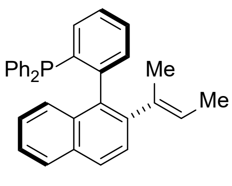

3aa

General Procedure **A**, a 25.0 mL dry Schlenk tube with a stirring bar was added with [Ir(cod)Cl]$_2$ (3.4 mg, 0.01 mmol, 0.05 equiv) and **L3** (7.3 mg, 0.022 mmol, 0.11 equiv) in 2.0 ml toluene under Ar. The formed mixture was stirred at room temperature for 1h. Then **1a** (77.6 mg, 0.2 mmol) and **2a** (2-butyne, 54.0 mg, 1.0 mmol) was added under argon, the resulting mixture was stirred at 70 °C. After 72 h, the solvent was removed under vaccum. The crude product purified by column chromatography on silica gel (PE/DCM = 10/1) to afford the corresponding product **3aa** as a white solid (89.6 mg, 82%). **^{1}H NMR (400 MHz, CDCl$_3$)** δ 7.81 (d, *J* = 8.5 Hz, 1H), 7.77 (d, *J* = 7.6 Hz, 1H), 7.41 – 7.32 (m, 4H), 7.32 – 7.27 (m, 2H), 7.24 (m, 3H), 7.17 – 7.11 (m, 3H), 7.10 – 7.05 (m, 2H), 7.02 – 6.92 (m, 4H), 5.37 (qd, *J* = 6.7, 1.6 Hz, 1H), 1.59 (s, 3H), 1.41 (d, *J* = 6.3 Hz, 3H); **^{13}C NMR (101 MHz, CDCl$_3$)** δ 146.4 (d, *J* = 35.0 Hz), 142.3 (d, *J* = 1.8 Hz), 138.3 (d, *J* = 13.2 Hz), 138.1 (d, *J* = 5.0 Hz), 137.9 (d, *J* = 2.7 Hz), 136.5, 136.0 (d, *J* = 7.3 Hz), 135.1 (d, *J* = 3.2 Hz), 133.8 (d, *J* = 21.3 Hz), 133.0 (d, *J* = 2.3 Hz), 132.9 (d, *J* = 18.2 Hz), 131.92, 131.85 (d, J = 6.4 Hz), 128.3 (d, *J* = 17.3 Hz), 128.1 (d, *J* = 5.4 Hz), 128.0 (d, *J* = 7.3 Hz), 127.6 (d, *J* = 5.0 Hz), 127.4 (d, *J* = 16.3 Hz), 127.0 (d, *J* = 36.8 Hz), 126.3 (d, *J* = 2.3 Hz), 125.3, 124.8, 17.7 (d, *J* = 2.3 Hz), 13.8; **^{31}P NMR (162 MHz, CDCl$_3$)** δ -15.66; **IR (film)**: 3670, 3601, 2360, 1735, 750 cm^{-1}; **HRMS m/z (ESI)**: calcd for $C_{32}H_{28}P$ [M+H]$^+$: 443.1923, found 443.1916.

The enantiomeric excess of **3aa** was determined by chiral HPLC analysis on Ciralpak IA column. Conditions: hexane/isopropanol = 99: 1 flow rate = 1.0 L/min, 25 °C, uv-vis detection at λ = 220 nm, 97% ee, t_{R1} = 8.4 min (minor), t_{R2} = 9.6 min (major); $[\alpha]_D^{25}$ = -9.58 (*c* = 1.9, CHCl$_3$). 99% ee of **3aa** can be obtained through one time recrystallization with MeOH.

(R,E)-(2-(2-(But-2-en-2-yl)naphthalen-1-yl)-4-methylphenyl)diphenylphosphane (3ba)

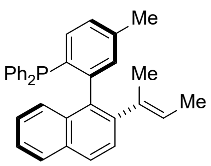

3ba

General Procedure **A**, a 25.0 mL dry Schlenk tube with a stirring bar was added with [Ir(cod)Cl]$_2$ (3.4 mg, 0.01 mmol, 0.05 equiv) and **L3** (7.3 mg, 0.022 mmol, 0.11 equiv) in 2.0 ml toluene under Ar. The formed mixture was stirred at room temperature for 1h. Then **1b** (80.4 mg, 0.2 mmol) and **2a** (54.0 mg, 1.0 mmol) was added under argon, the resulting mixture was stirred at 70 °C. After 72 h, the solvent was removed under vaccum. The crude product purified by column chromatography on silica gel (PE/DCM = 10/1) to afford the corresponding product **3ba** as a white solid (71.1 mg, 78%). **^{1}H NMR (500 MHz, CDCl$_3$)** δ 7.87 (d, J = 8.5 Hz, 1H), 7.84 (d, J = 8.1 Hz, 1H), 7.41 (d, J = 8.4 Hz, 1H), 7.37 – 7.28 (m, 5H), 7.25 – 7.17 (m, 5H), 7.15 (td, J = 7.2, 1.5 Hz, 2H), 7.11 – 7.01 (m, 4H), 5.41 (qd, J = 6.9, 1.6 Hz, 1H), 2.42 (s, 3H), 1.65 (s, 3H), 1.46 (d, J = 7.1 Hz, 3H); **^{13}C NMR (126 MHz, CDCl$_3$)** δ 146.5 (d, *J* = 35.5 Hz), 142.2, 138.6 (d, *J* = 13.1 Hz), 138.3, 136.5, 136.2 (d, *J* = 6.7 Hz), 135.2 (d, *J* = 2.9 Hz), 134.3 (d, *J* = 10.1 Hz), 133.6 (d, *J* = 20.8 Hz), 133.1, 132.8 (d, *J* = 17.8 Hz), 132.5 (d, *J* = 6.7 Hz), 131.9, 128.2 (d, *J* = 36.9 Hz), 128.01 , 127.97 , 127.91 , 127.51 , 127.46 , 127.1 (d, *J* = 40.1 Hz), 126.1, 125.0 (d, *J* = 59.7 Hz), 21.3, 17.8, 13.8; **^{31}P NMR (202 MHz, CDCl$_3$)** δ -16.80; **IR (film)**: 3861, 3700, 3601, 2360, 1276 cm^{-1}; **HRMS m/z (ESI)**: calcd for $C_{33}H_{30}P$ [M+H]$^+$: 457.2080, found 457.2066.

The enantiomeric excess of **3ba** was determined by chiral HPLC analysis on Ciralpak IA column. Conditions: hexane/isopropanol = 99: 1 flow rate = 0.5 L/min, 25 °C, uv-vis detection at λ = 220 nm, 97% ee, t_{R1} = 4.7 min (minor), t_{R2} = 5.0 min (major); $[\alpha]_D^{25}$ = -111.45 (*c* = 1.59, CHCl$_3$).

(R,E)-(2-(2-(But-2-en-2-yl)naphthalen-1-yl)-5-methylphenyl)diphenylphosphane (3ca)

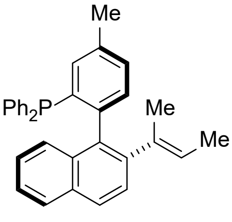

3ca

General Procedure **A**, a 25.0 mL dry Schlenk tube with a stirring bar was added with [Ir(cod)Cl]$_2$ (3.4 mg, 0.01 mmol, 0.05 equiv) and **L3** (7.3 mg, 0.022 mmol, 0.11 equiv) in 2.0 ml toluene under Ar. The formed mixture was stirred at room temperature for 1h.

Then **1c** (80.4 mg, 0.2 mmol) and **2a** (54.0 mg, 1.0 mmol) was added under argon, the resulting mixture was stirred at 70 °C. After 72 h, the solvent was removed under vaccum. The crude product purified by column chromatography on silica gel (PE/DCM = 10/1) to afford the corresponding product **3ca** as a white solid (82.1 mg, 90%). **^{1}H NMR (400 MHz, CDCl$_3$)** δ 7.81 (d, *J* = 8.4 Hz, 1H), 7.77 (d, *J* = 8.2 Hz, 1H), 7.35 (d, *J* = 8.4 Hz, 1H), 7.31 – 7.27 (m, 1H), 7.27 – 7.23 (m, 3H), 7.22 – 7.19 (m, 2H), 7.19 – 7.15 (m, 2H), 7.15 – 7.12 (m, 2H), 7.12 – 7.07 (m, 2H), 7.03 – 7.00 (m, 1H), 7.00 – 6.95 (m, 3H), 5.36 (qd, *J* = 6.8, 1.5 Hz, 1H), 2.33 (s, 3H), 1.59 (t, *J* = 1.3 Hz, 3H), 1.43 – 1.40 (m, 3H); **^{13}C NMR (101 MHz, CDCl$_3$)** δ 143.5 (d, *J* = 35.4 Hz), 142.5 (d, *J* = 1.8 Hz), 138.5 (d, *J* = 13.6 Hz), 138.3 (d, *J* = 14.1 Hz), 137.5 (d, *J* = 11.4 Hz), 136.8, 136.7, 136.1 (d, *J* = 6.8 Hz), 135.4 (d, *J* = 2.7 Hz), 133.8 (d, *J* = 21.3 Hz), 133.3 (d, *J* = 1.8 Hz), 132.9 (d, *J* = 18.2 Hz), 132.0, 131.7 (d, *J* = 6.8 Hz), 129.4, 128.1 (d, *J* = 5.9 Hz), 128.0, 127.9, 127.5 (d, *J* = 2.7 Hz), 127.4 (d, *J* = 17.3 Hz), 126.9, 126.1 (d, *J* = 2.3 Hz), 125.0 (d, *J* = 46.3 Hz), 21.4, 17.8 (d, *J* = 2.3 Hz), 13.8; **^{31}P NMR (202 MHz, CDCl$_3$)** δ -16.80; **IR (film)**: 3862, 3702, 3599, 2360, 1276 cm^{-1}; **HRMS m/z (ESI)**: calcd for $C_{33}H_{30}P$ $[M+H]^+$: 457.2080, found 457.2068.

The enantiomeric excess of **3ca** was determined by chiral HPLC analysis on Ciralpak IA column. Conditions: hexane/isopropanol = 99: 1 flow rate = 0.5 L/min, 25 °C, uv-vis detection at λ = 254 nm, 99% ee, t_{R1} = 5.3 min (minor), t_{R2} = 5.7 min (major); $[\alpha]_D^{25}$ = -102.22 (*c* = 1.26, CHCl$_3$).

(R,E)-(2-(2-(But-2-en-2-yl)-4-methylnaphthalen-1-yl)phenyl)diphenylphosphane (3da)

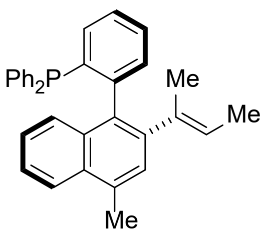

3da

General Procedure **A**, a 25.0 mL dry Schlenk tube with a stirring bar was added with $[Ir(cod)Cl]_2$ (3.4 mg, 0.01 mmol, 0.05 equiv) and **L3** (7.3 mg, 0.022 mmol, 0.11 equiv) in 2.0 ml toluene under Ar. The formed mixture was stirred at room temperature for 1h. Then **1d** (80.4 mg, 0.2 mmol) and **2a** (54.0 mg, 1.0 mmol) was added under argon, the resulting mixture was stirred at 70 °C. After 72 h, the solvent was removed under vaccum. The crude

product purified by column chromatography on silica gel (PE/DCM = 10/1) to afford the corresponding product **3da** as a white solid (71.1 mg, 78%). **^{1}H NMR (500 MHz, CDCl$_3$)** δ 8.01 (d, *J* = 8.4 Hz, 1H), 7.47 – 7.42 (m, 2H), 7.41 – 7.38 (m, 2H), 7.38 – 7.34 (m, 1H), 7.33 – 7.29 (m, 4H), 7.23 – 7.19 (m, 3H), 7.16 (td, *J* = 7.4, 6.9, 1.6 Hz, 2H), 7.10 – 7.02 (m, 4H), 5.46 – 5.38 (m, 1H), 2.79 (s, 3H), 1.65 (s, 3H), 1.46 (dd, *J* = 6.7, 1.2 Hz, 3H); **^{13}C NMR (126 MHz, CDCl$_3$)** δ 146.7 (d, *J* = 35.3 Hz), 141.8 (d, *J* = 1.7 Hz), 138.4 (d, *J* = 13.3 Hz), 138.2 (d, *J* = 13.9 Hz), 138.0 (d, *J* = 11.0 Hz), 136.5, 135.2 (d, *J* = 2.9 Hz), 134.4 (d, *J* = 6.9 Hz), 133.8, 133.6, 133.2 (d, *J* = 1.7 Hz), 132.9 (d, J = 17.9 Hz), 132.1 (d, *J* = 6.4 Hz), 131.1, 128.4, 128.13, 128.06, 128.0 (d, *J* = 2.3 Hz), 127.9, 127.5 (d, *J* = 13.9 Hz), 127.3, 126.0 (d, *J* = 2.3 Hz), 124.8 (d, *J* = 37.6 Hz), 123.7, 19.6, 17.7 (d, *J* = 1.5 Hz), 13.8; **^{31}P NMR (202 MHz, CDCl$_3$)** δ -15.99; **IR (film)**: 3054, 2987, 1753, 1735, 1440, 909, 730, 703 cm^{-1}; **HRMS m/z (ESI)**: calcd for $C_{33}H_{30}P$ [M+H]$^{+}$: 457.2080, found 457.2069.

The enantiomeric excess of **3da** was determined by chiral HPLC analysis on Ciralpak IA column. Conditions: hexane/isopropanol = 99: 1 flow rate = 0.5 L/min, 25 oC, uv-vis detection at λ = 220 nm, 99% ee, t_{R1} = 4.7 min (minor), t_{R2} = 5.0 min (major) ; $[\alpha]_D^{25}$ = -92.87 (*c* = 3.0, CHCl$_3$).

(R,E)-(2-(2-(But-2-en-2-yl)naphthalen-1-yl)-4,5-dimethylphenyl)diphenylphosphane (3ea)

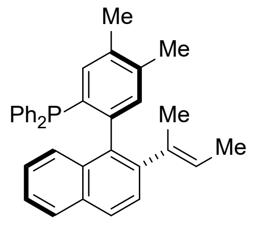

3ea

General Procedure **A**, a 25.0 mL dry Schlenk tube with a stirring bar was added with [Ir(cod)Cl]$_2$ (3.4 mg, 0.01 mmol, 0.05 equiv) and **L3** (7.3 mg, 0.022 mmol, 0.11 equiv) in 2.0 ml toluene under Ar. The formed mixture was stirred at room temperature for 1h. Then **1e** (83.2 mg, 0.2 mmol) and **2a** (54.0 mg, 1.0 mmol) was added under argon, the resulting mixture was stirred at 70 ºC. After 72 h, the solvent was removed under vaccum. The crude product purified by column chromatography on silica gel (PE/DCM = 10/1) to afford the corresponding product **3ea** as a white solid (58.3 mg, 62%). **^{1}H NMR (400 MHz, CDCl$_3$)** δ 7.79 (t, *J* = 8.1 Hz, 2H), 7.34 (d, *J* = 8.4 Hz, 1H), 7.30 (td, *J* = 5.1, 2.4 Hz, 1H), 7.30 – 7.20 (m,

3H), 7.20 – 7.11 (m, 2H), 7.12 (dt, *J* = 2.3, 1.4 Hz, 2H), 7.10 (q, *J* = 1.2 Hz, 1H), 7.11 – 7.05 (m, 1H), 7.07 – 7.00 (m, 2H), 7.04 – 6.95 (m, 2H), 5.34 (qd, *J* = 6.8, 1.5 Hz, 1H), 2.27 (s, 3H), 2.24 (s, 3H), 1.59 (t, *J* = 1.3 Hz, 3H), 1.39 (dd, *J* = 6.8, 1.2 Hz, 2H); **^{13}C NMR (101 MHz, $CDCl_3$)** δ 144.1 (d, *J* = 35.9 Hz), 142.4 (d, *J* = 2.3 Hz), 138.9 (d, *J* = 7.7 Hz), 138.7 (d, *J* = 6.4 Hz), 137.2, 136.7, 136.2 (d, *J* = 6.8 Hz), 136.1 (d, *J* = 3.2 Hz), 135.6, 135.4 (d, *J* = 3.2 Hz), 134.3 (d, *J* = 10.0 Hz), 133.8 (d, *J* = 20.9 Hz), 133.6 (d, *J* = 20.9 Hz), 133.3 (d, *J* = 2.3 Hz), 132.9 (d, *J* = 7.3 Hz), 132.8 (d, *J* = 17.7 Hz), 132.0, 128.0 (d, *J* = 2.5 Hz), 127.9 (d, *J* = 4.1 Hz), 127.5 (d, *J* = 10.4 Hz), 127.3 (d, *J* = 3.2 Hz), 127.0, 126.0 (d, *J* = 2.3 Hz), 125.0 (d, *J* = 47.2 Hz), 19.7 (d, *J* = 2.3 Hz), 17.8 (d, *J* = 1.8 Hz), 13.8; **^{31}P NMR (162 MHz, $CDCl_3$)** δ -16.69; **IR (film)**: 3725, 3600, 2360, 1276 cm^{-1}; **HRMS m/z (ESI)**: calcd for $C_{34}H_{32}P$ $[M+H]^+$: 471.2236, found 471.2226.

The enantiomeric excess of **3ea** was determined by chiral HPLC analysis on Ciralpak IA column. Conditions: hexane/isopropanol = 99: 1 flow rate = 0.7 L/min, 25 ºC, uv-vis detection at λ = 220 nm, 97% ee, t_{R1} = 5.3 min (minor), t_{R2} = 5.8 min (major) ; $[\alpha]_D^{25}$ = -93.61 (*c* = 0.49, $CHCl_3$).

(R,E)-(2-(2-(But-2-en-2-yl)-4-phenylnaphthalen-1-yl)phenyl)diphenylphosphane (3fa)

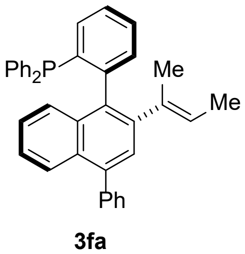

3fa

General Procedure **A**, a 25.0 mL dry Schlenk tube with a stirring bar was added with $[Ir(cod)Cl]_2$ (3.4 mg, 0.01 mmol, 0.05 equiv) and **L3** (7.3 mg, 0.022 mmol, 0.11 equiv) in 2.0 ml toluene under Ar. The formed mixture was stirred at room temperature for 1h. Then **1f** (92.8 mg, 0.2 mmol) and **2a** (54.0 mg, 1.0 mmol) was added under argon, the resulting mixture was stirred at 70 ºC. After 72 h, the solvent was removed under vaccum. The crude product purified by column chromatography on silica gel (PE/DCM = 10/1) to afford the corresponding product **3fa** as a white solid (70.4 mg, 68%). **^{1}H NMR (500 MHz, $CDCl_3$)** δ 7.95 (d, *J* = 8.2 Hz, 1H), 7.69 – 7.64 (m, 2H), 7.55 (t, *J* = 7.7 Hz, 2H), 7.47 (td, *J* = 6.1, 5.0,

1.9 Hz, 3H), 7.45 – 7.40 (m, 3H), 7.35 – 7.28 (m, 4H), 7.25 – 7.21 (m, 3H), 7.17 (td, *J* = 7.3, 6.9, 1.7 Hz, 2H), 7.13 – 7.05 (m, 4H), 5.48 (qd, *J* = 6.7, 1.5 Hz, 1H), 1.68 (s, 3H), 1.48 (d, *J* = 6.8 Hz, 3H); **^{13}C NMR (126 MHz, CDCl$_3$)** δ 146.6 (d, *J* = 35.0 Hz), 141.9 (d, *J* = 2.3 Hz), 141.0, 139.7, 138.3 (d, *J* = 12.7 Hz), 138.2 (d, *J* = 8.7 Hz), 138.1 (d, *J* = 6.4 Hz), 136.5, 135.7 (d, *J* = 6.4 Hz), 135.3 (d, *J* = 2.9 Hz), 133.9 (d, *J* = 21.4 Hz), 133.5 (d, *J* = 2.9 Hz), 133.0 (d, *J* = 18.5 Hz), 132.2 (d, *J* = 6.4 Hz), 130.4, 130.2, 128.5 (d, *J* = 7.5 Hz), 128.3, 128.2, 128.2, 128.1 (d, *J* = 6.9 Hz), 127.8, 127.5, 127.3, 127.2, 126.6 (d, *J* = 2.9 Hz), 125.7, 125.2, 125.0, 17.8 (d, *J* = 1.7 Hz), 13.9; **^{31}P NMR (202 MHz, CDCl$_3$)** δ -15.52; **IR (film)**: 3726, 3682, 2360, 1276 cm^{-1}; **HRMS m/z (ESI)**: calcd for $C_{38}H_{32}P$ [M+H]$^+$: 519.2236, found 519.2225; $[\alpha]_D^{25}$ = -46.83 (*c* = 2.32, $CHCl_3$).

The enantiomeric excess of **3fa** was determined by chiral HPLC analysis on Ciralpak IA column. Conditions: hexane/isopropanol = 99: 1 flow rate = 0.5 L/min, 25 °C, uv-vis detection at λ = 300 nm, 98% ee, t_{R1} = 5.3 min (minor), t_{R2} = 5.6 min (major).

(R,E)-(2-(2-(But-2-en-2-yl)naphthalen-1-yl)-4-methoxyphenyl)diphenylphosphane (3ga)

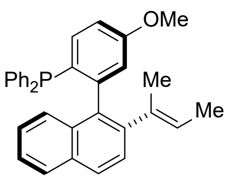

3ga

General Procedure **A**, a 25.0 mL dry Schlenk tube with a stirring bar was added with $[Ir(cod)Cl]_2$ (3.4 mg, 0.01 mmol, 0.05 equiv) and **L3** (7.3 mg, 0.022 mmol, 0.11 equiv) in 2.0 ml toluene under Ar. The formed mixture was stirred at room temperature for 1h. Then **1g** (83.6 mg, 0.2 mmol) and **2a** (54.0 mg, 1.0 mmol) was added under argon, the resulting mixture was stirred at 70 °C. After 72 h, the solvent was removed under vaccum. The crude product purified by column chromatography on silica gel (PE/DCM = 10/1) to afford the corresponding product **3ga** as a white solid (71.7 mg, 76%). **^{1}H NMR (400 MHz, CDCl$_3$)** δ 7.83 (d, *J* = 8.4 Hz, 1H), 7.80 (d, *J* = 8.2 Hz, 1H), 7.36 (d, *J* = 8.6 Hz, 1H), 7.34 – 7.28 (m, 2H), 7.26 – 7.22 (m, 3H), 7.17 – 7.09 (m, 5H), 7.05 (d, *J* = 3.3 Hz, 2H), 7.02 – 6.96 (m, 2H), 6.93 (dd, *J* = 8.6, 2.7 Hz, 1H), 6.86 (dd, *J* = 3.8, 2.7 Hz, 1H), 5.41 – 5.27 (m, 1H), 3.78 (s, 3H), 1.62 (s, 3H), 1.41 (dd, *J* = 6.7, 1.2 Hz, 3H); **^{13}C NMR (101 MHz, CDCl$_3$)** δ 159.7, 148.2 (d, *J*

= 37.2 Hz), 142.1 (d, J = 2.3 Hz), 139.0, 138.8, 138.7, 136.7 (d, J = 2.7 Hz), 136.4, 136.0 (d, J = 6.8 Hz), 133.5 (d, J = 20.9 Hz), 132.8 (d, J = 17.7 Hz), 131.9, 128.02 (d, J = 2.3 Hz), 127.98 (d, J = 1.4 Hz), 127.9, 127.6, 127.5 (d, J = 5.0 Hz), 127.2, 126.9, 126.1 (d, J = 1.8 Hz), 125.4, 124.9, 116.6 (d, J = 7.3 Hz), 114.1, 55.2, 17.8 (d, J = 1.8 Hz), 13.8; **^{31}P NMR (162 MHz, CDCl$_3$)** δ -17.81; **IR (film)**: 3828, 3560, 2360, 1276, cm^{-1}; **HRMS m/z (ESI)**: calcd for $C_{33}H_{30}OP$ [M+H]$^+$: 473.2029, found 473.2018.

The enantiomeric excess of **3ga** was determined by chiral HPLC analysis on Ciralpak IA column. Conditions: hexane/isopropanol = 99: 1 flow rate = 0.5 L/min, 25 ºC, uv-vis detection at λ = 220 nm, 94% ee, t_{R1} = 9.8 min (minor), t_{R2} = 13.8 min (major) ; $[\alpha]_D^{25}$ = -66.99 (c = 2.06, CHCl$_3$).

(R,E)-(2-(2-(But-2-en-2-yl)-4-methoxynaphthalen-1-yl)phenyl)diphenylphosphane (3ha)

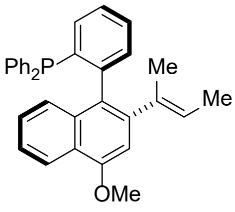

3ha

General Procedure **A**, a 25.0 mL dry Schlenk tube with a stirring bar was added with [Ir(cod)Cl]$_2$ (3.4 mg, 0.01 mmol, 0.05 equiv) and **L3** (7.3 mg, 0.022 mmol, 0.11 equiv) in 2.0 ml toluene under Ar. The formed mixture was stirred at room temperature for 1h. Then **1h** (83.6 mg, 0.2 mmol) and **2a** (54.0 mg, 1.0 mmol) was added under argon, the resulting mixture was stirred at 70 ºC. After 72 h, the solvent was removed under vaccum. The crude product purified by column chromatography on silica gel (PE/DCM = 10/1) to afford the corresponding product **3ha** as a white solid (67.0 mg, 71%). **^{1}H NMR (500 MHz, CDCl$_3$)** δ 8.29 (d, J = 8.3 Hz, 1H), 7.47 – 7.40 (m, 3H), 7.39 – 7.33 (m, 3H), 7.33 – 7.31 (m, 2H), 7.24 – 7.19 (m, 3H), 7.15 (td, J = 7.4, 1.6 Hz, 2H), 7.06 (qd, J = 8.2, 1.5 Hz, 3H), 6.94 (d, J = 8.2 Hz, 1H), 6.78 (s, 1H), 5.47 (qd, J = 6.7, 1.5 Hz, 1H), 4.08 (s, 3H), 1.68 (s, 3H), 1.50 – 1.47 (m, 3H); **^{13}C NMR (126 MHz, CDCl$_3$)** δ 154.6, 146.6 (d, J = 34.7 Hz), 142.3 (d, J = 2.3 Hz), 138.6 (d, J = 6.4 Hz), 138.5 (d, J = 4.0 Hz), 138.1 (d, J = 13.9 Hz), 137.0, 135.1 (d, J = 2.9 Hz), 133.8 (d, J = 20.8 Hz), 132.8 (d, J = 17.9 Hz), 132.4 (d, J = 6.4 Hz), 128.3 (d, J = 26.0 Hz), 128.03 (d, J = 5.5 Hz), 127.97 (d, J = 7.1 Hz), 127.5, 127.2, 126.6, 126.0 (d, J = 2.3 Hz), 125.8, 124.2, 121.4, 105.2, 55.3, 17.7 (d, J = 1.7 Hz), 13.7. 17.7

(d, J = 1.7 Hz); **^{31}P NMR (202 MHz, $CDCl_3$)** δ -15.76; **IR (film)**: 3788, 3662, 3576, 2360, 1276 cm^{-1}; **HRMS m/z (ESI)**: calcd for $C_{33}H_{30}OP$ [M+H]$^+$: 473.2029, found 473.2019.

The enantiomeric excess of **3ha** was determined by chiral HPLC analysis on Ciralpak IA column. Conditions: hexane/isopropanol = 99: 1 flow rate = 1.0 L/min, 25 °C, uv-vis detection at λ = 220 nm, 99% ee, t_{R1} = 5.3 min (minor), t_{R2} = 5.5 min (major) ; $[\alpha]_D^{25}$ = -55.29 (c = 2.89, $CHCl_3$).

(R,E)-(6-(2-(But-2-en-2-yl)naphthalen-1-yl)benzo[d][1,3]dioxol-5-yl)diphenylphosphane (3ia)

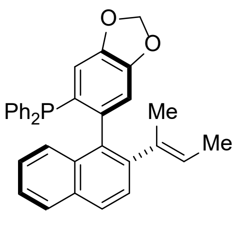

3ia

General Procedure **A**, a 25.0 mL dry Schlenk tube with a stirring bar was added with $[Ir(cod)Cl]_2$ (3.4 mg, 0.01 mmol, 0.05 equiv) and **L3** (7.3 mg, 0.022 mmol, 0.11 equiv) in 2.0 ml toluene under Ar. The formed mixture was stirred at room temperature for 1h. Then **1i** (86.2 mg, 0.2 mmol) and **2a** (54.0 mg, 1.0 mmol) was added under argon, the resulting mixture was stirred at 70 °C. After 72 h, the solvent was removed under vaccum. The crude product purified by column chromatography on silica gel (PE/DCM = 10/1) to afford the corresponding product **3ia** as a white solid (75.8 mg, 78%). **^{1}H NMR (400 MHz, $CDCl_3$)** δ 7.81 (t, J = 9.0 Hz, 2H), 7.35 (d, J = 8.4 Hz, 1H), 7.33 – 7.30 (m, 1H), 7.29 – 7.27 (m, 1H), 7.26 – 7.23 (m, 2H), 7.21 – 7.16 (m, 1H), 7.16 – 7.11 (m, 3H), 7.11 – 7.06 (m, 2H), 7.01 – 6.92 (m, 2H), 6.82 (d, J = 2.3 Hz, 1H), 6.79 (d, J = 3.7 Hz, 1H), 6.05 (d, J = 1.3 Hz, 1H), 6.01 (d, J = 1.3 Hz, 1H), 5.40 – 5.31 (m, 1H), 1.63 (t, J = 1.3 Hz, 3H), 1.44 (dd, J = 6.8, 1.2 Hz, 3H); **^{13}C NMR (101 MHz, $CDCl_3$)** δ 148.2, 147.1, 142.6 (d, J = 2.3 Hz), 141.2 (d, J = 38.1 Hz), 138.7, 138.6, 138.4, 136.5, 135.7 (d, J = 7.7 Hz), 133.5 (d, J = 20.9 Hz), 132.7 (d, J = 18.2 Hz), 132.0, 128.14 (d, J = 5.5 Hz), 128.06, 128.0, 127.64, 127.60, 127.1 (d, J = 48.6 Hz), 126.3 (d, J = 2.3 Hz), 125.2 (d, J = 53.1 Hz), 114.0 (d, J = 2.3 Hz), 111.9 (d, J = 7.3 Hz), 101.3, 17.8, 13.9; **^{31}P NMR (162 MHz, $CDCl_3$)** δ -15.41; **IR (film)**: 3810, 3601, 2361, 1276 cm^{-1}; **HRMS m/z (ESI)**: calcd for $C_{33}H_{28}O_2P$ [M+H]$^+$: 487.1821, found 487.1811.

The enantiomeric excess of **3ia** was determined by chiral HPLC analysis on Ciralpak IA column. Conditions: hexane/isopropanol = 99: 1 flow rate = 0.5 L/min, 25 °C, uv-vis detection at λ = 254 nm, 95% ee, t_{R1} = 11.8 min (minor), t_{R2} = 16.4 min (major) ; $[\alpha]_D^{25}$ = -120.00 (c = 0.21, $CHCl_3$).

(R,E)-(2-(2-(But-2-en-2-yl)naphthalen-1-yl)-5-fluorophenyl)diphenylphosphane (3ja)

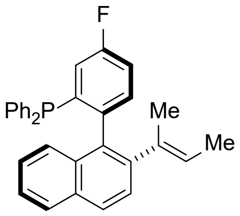

3ja

General Procedure **A**, a 25.0 mL dry Schlenk tube with a stirring bar was added with $[Ir(cod)Cl]_2$ (3.4 mg, 0.01 mmol, 0.05 equiv) and **L3** (7.3 mg, 0.022 mmol, 0.11 equiv) in 2.0 ml toluene under Ar. The formed mixture was stirred at room temperature for 1h. Then **1j** (80.4 mg, 0.2 mmol) and **2a** (54.0 mg, 1.0 mmol) was added under argon, the resulting mixture was stirred at 70 °C. After 72 h, the solvent was removed under vaccum. The crude product purified by column chromatography on silica gel (PE/DCM = 10/1) to afford the corresponding product **3ja** as a white solid (71.8 mg, 78%). **^{1}H NMR (400 MHz, $CDCl_3$)** δ 7.82 (d, J = 8.4 Hz, 1H), 7.77 (d, J = 8.2 Hz, 1H), 7.36 (d, J = 8.4 Hz, 1H), 7.31 – 7.23 (m, 5H), 7.20 – 7.14 (m, 1H), 7.14 – 7.09 (m, 4H), 7.08 – 7.02 (m, 2H), 7.01 – 6.91 (m, 3H), 6.87 (d, J = 8.4 Hz, 1H), 5.37 (qd, J = 6.8, 1.6 Hz, 1H), 1.61 (s, 3H), 1.46 (d, J = 6.1 Hz, 3H); **^{13}C NMR (101 MHz, CDCl3)** δ 161.9 (d, J = 248.4 Hz), 142.6, 142.0 (dd, J = 34.0, 2.9 Hz), 141.1 (dd, J = 15.6, 4.4 Hz), 137.5 (d, J = 13.6 Hz), 136.9 (d, J = 14.1 Hz), 136.4, 134.8 (d, J = 6.4 Hz), 133.9 (d, J = 21.3 Hz), 133.3 (t, J = 6.8 Hz), 132.8 (d, J = 18.6 Hz), 131.9, 128.6, 128.3 (d, J = 5.9 Hz), 128.1 (d, J = 7.7 Hz), 127.9 (d, J = 17.3 Hz), 127.4 (d, J = 33.6 Hz), 126.6, 126.4, 125.1 (d, J = 52.2 Hz), 120.9 (d, J = 22.3 Hz), 115.73 (d, J = 21.3 Hz), 17.8, 13.9; **^{31}P NMR (162 MHz, $CDCl_3$)** δ -15.01; **^{19}F NMR (376 MHz, $CDCl_3$)** δ -114.59; **IR (film)**: 3846, 3598, 2360, 1276 cm^{-1}; **HRMS m/z (ESI)**: calcd for $C_{32}H_{27}FP$ $[M+H]^+$: 461.1826, found 461.1816.

The enantiomeric excess of **3ja** was determined by chiral HPLC analysis on Ciralpak IA column. Conditions: hexane/isopropanol = 99: 1 flow rate = 0.5 L/min, 25 °C,

uv-vis detection at λ = 220 nm, 99% ee, t_{R1} = 5.6 min (minor), t_{R2} = 6.0 min (major) ; $[\alpha]_D^{25}$ = -118.36 (c = 1.1, $CHCl_3$).

(R,E)-(2-(2-(But-2-en-2-yl)naphthalen-1-yl)-4,5-difluorophenyl)diphenylphosphane (3ka)

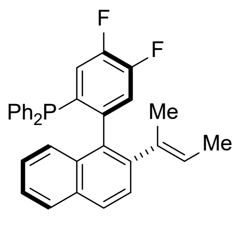

3ka

General Procedure **A**, a 25.0 mL dry Schlenk tube with a stirring bar was added with $[Ir(cod)Cl]_2$ (3.4 mg, 0.01 mmol, 0.05 equiv) and **L3** (7.3 mg, 0.022 mmol, 0.11 equiv) in 2.0 ml toluene under Ar. The formed mixture was stirred at room temperature for 1h. Then **1k** (84.8 mg, 0.2 mmol) and **2a** (54.0 mg, 1.0 mmol) was added under argon, the resulting mixture was stirred at 70 ºC. After 72 h, the solvent was removed under vaccum. The crude product purified by column chromatography on silica gel (PE/DCM = 10/1) to afford the corresponding product **3ka** as a white solid (54.5 mg, 57%). **^{1}H NMR (500 MHz, $CDCl_3$)** δ 7.85 (d, J = 8.4 Hz, 1H), 7.80 (d, J = 8.2 Hz, 1H), 7.37 (d, J = 8.4 Hz, 1H), 7.33 – 7.28 (m, 4H), 7.24 – 7.17 (m, 1H), 7.17 – 7.08 (m, 6H), 7.02 (td, J = 7.6, 6.9, 1.4 Hz, 1H), 6.94 (td, J = 8.0, 1.5 Hz, 2H), 6.89 (d, J = 8.5 Hz, 1H), 5.37 (qd, J = 6.8, 1.6 Hz, 1H), 1.66 (s, 3H), 1.49 (dd, J = 6.7, 1.2 Hz, 3H); **^{13}C NMR (126 MHz, CDCl3)** δ 150.9 (dd, J = 97.4, 12.4 Hz), 148.9 (dd, J = 96.2, 12.4 Hz), 143.3 (ddd, J = 36.3, 6.1, 4.2 Hz), 142.6 (d, J = 1.5 Hz), 137.4 (d, J = 13.3 Hz), 136.9 (d, J = 13.9 Hz), 136.0 , 135.7 - 135.5 (m), 133.8 (d, J = 21.4 Hz), 132.7 (d, J = 18.5 Hz), 131.9, 128.7, 128.4 (d, J = 5.8 Hz), 128.24 (d, J = 7.5 Hz), 128.15 (d, J = 7.5 Hz), 127.4 (d, J = 65.3 Hz), 126.7 (d, J = 2.9 Hz), 126.3, 125.6, 125.3, 123.1 (d, J = 16.2 Hz), 120.5 (dd, J = 15.9, 6.6 Hz), 17.9 (d, J = 2.3 Hz), 13.9; **^{31}P NMR (202 MHz, $CDCl_3$)** δ -16.29; **^{19}F NMR (471 MHz, $CDCl_3$)** δ -137.10 (d, J = 21.7 Hz, 1F), -138.76 (d, J = 21.7 Hz, 1F); **IR (film)**: 3846, 3600, 2360, 1277 cm^{-1}; **HRMS m/z (ESI)**: calcd for $C_{32}H_{26}F_2P$ $[M+H]^+$: 479.1735, found 479.1725.

The enantiomeric excess of **3ka** was determined by chiral HPLC analysis on Ciralpak IA column. Conditions: hexane/isopropanol = 99: 1 flow rate = 0.5 L/min, 25 ºC, uv-vis detection at λ = 220 nm, 97% ee, t_{R1} = 5.4 min (minor), t_{R2} = 5.9 min (major) ; $[\alpha]_D^{25}$ = -55.32 (c = 1.0, $CHCl_3$).

(R,E)-(2-(2-(But-2-en-2-yl)naphthalen-1-yl)-4-chlorophenyl)diphenylphosphane (3la)

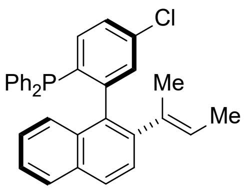

3la

General Procedure **A**, a 25.0 mL dry Schlenk tube with a stirring bar was added with [Ir(cod)Cl]2 (3.4 mg, 0.01 mmol, 0.05 equiv) and **L3** (7.3 mg, 0.022 mmol, 0.11 equiv) in 2.0 ml toluene under Ar. The formed mixture was stirred at room temperature for 1h. Then **1l** (84.4 mg, 0.2 mmol) and **2a** (54.0 mg, 1.0 mmol) was added under argon, the resulting mixture was stirred at 70 °C. After 72 h, the solvent was removed under vaccum. The crude product purified by column chromatography on silica gel (PE/DCM = 10/1) to afford the corresponding product **3la** as a white solid (64.9 mg, 65%). **^{1}H NMR (400 MHz, $CDCl_3$)** δ 7.83 (d, *J* = 8.3 Hz, 1H), 7.78 (d, *J* = 8.5 Hz, 1H), 7.40 – 7.29 (m, 2H), 7.32 – 7.22 (m, 3H), 7.21 – 7.12 (m, 1H), 7.16 – 7.05 (m, 3H), 7.09 – 6.98 (m, 2H), 6.95 (td, *J* = 7.7, 1.5 Hz, 3H), 5.36 (qd, *J* = 6.8, 1.5 Hz, 1H), 1.63 (t, *J* = 1.3 Hz, 3H), 1.43 (dd, *J* = 6.9, 1.3 Hz, 2H); **^{13}C NMR (101 MHz, CDCl3)** δ 164.0, 161.5, 149.1 (d, *J* = 7.7 Hz), 148.7 (d, *J* = 7.7 Hz), 142.2 (d, *J* = 2.3 Hz), 138.1 (d, *J* = 12.7 Hz), 137.8 (d, *J* = 13.6 Hz), 137.12 (d, *J* = 2.7 Hz), 137.06 (d, *J* = 2.3 Hz), 136.1, 133.6 (d, *J* = 20.9 Hz), 132.8 (d, *J* = 17.7 Hz), 131.9, 128.3, 128.2, 128.1 (d, *J* = 2.7 Hz), 128.0 (d, *J* = 7.7 Hz), 127.7 (d, *J* = 17.3 Hz), 127.1, 126.6 (d, *J* = 2.3 Hz), 126.5, 125.5, 125.0, 118.7 (d, *J* = 6.8 Hz), 118.5 (d, *J* = 6.8 Hz), 114.8 (d, *J* = 20.9 Hz), 17.8 (d, *J* = 2.3 Hz), 13.9; **^{31}P NMR (202 MHz, $CDCl_3$)** δ -17.55; **IR (film)**: 3725, 3601, 2360, 1276 cm^{-1}; **HRMS m/z (ESI)**: calcd for $C_{32}H_{27}ClP$ $[M+H]^+$: 477.1533, found 477.1524.

The enantiomeric excess of **3la** was determined by chiral HPLC analysis on Ciralpak IA column. Conditions: hexane/isopropanol = 99: 1 flow rate = 0.5 L/min, 25 °C, uv-vis detection at λ = 254 nm, 97% ee, t_{R1} = 5.6 min (minor), t_{R2} = 6.2 min (major) ; $[\alpha]_D^{25}$ = -96.00 (*c* = 3.05, $CHCl_3$).

(R,E)-(2-(2-(But-2-en-2-yl)-4-chloronaphthalen-1-yl)phenyl)diphenylphosphane (3ma)

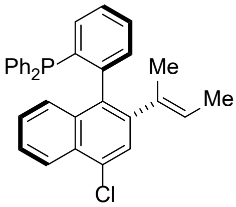

3ma

General Procedure **A**, a 25.0 mL dry Schlenk tube with a stirring bar was added with $[Ir(cod)Cl]_2$ (3.4 mg, 0.01 mmol, 0.05 equiv) and **L3** (7.3 mg, 0.022 mmol, 0.11 equiv) in 2.0 ml toluene under Ar. The formed mixture was stirred at room temperature for 1h. Then **1m** (84.4 mg, 0.2 mmol) and **2a** (54.0 mg, 1.0 mmol) was added under argon, the resulting mixture was stirred at 70 °C. After 72 h, the solvent was removed under vaccum. The crude product purified by column chromatography on silica gel (PE/DCM = 10/1) to afford the corresponding product **3ma** as a white solid (82.8 mg, 87%). **^{1}H NMR (500 MHz, CDCl$_3$)** δ 8.27 (d, J = 8.4 Hz, 1H), 7.55 (s, 1H), 7.47 – 7.37 (m, 4H), 7.33 – 7.29 (m, 4H), 7.23 – 7.11 (m, 5H), 7.09 – 7.05 (m, 1H), 7.04 – 6.97 (m, 3H), 5.45 (qd, J = 6.8, 1.5 Hz, 1H), 1.63 (s, 3H), 1.48 – 1.46 (m, 3H); **^{13}C NMR (126 MHz, CDCl$_3$)** δ 145.5 (d, J = 34.8 Hz), 142.5 (d, J = 1.7 Hz), 138.05, 138.04 (d, J = 24.3 Hz), 137.6 (d, J = 13.3 Hz), 135.6, 135.4 (d, J = 6.4 Hz), 135.1 (d, J = 2.9 Hz), 134.2 (d, J = 2.3 Hz), 133.8 (d, J = 21.4 Hz), 132.8 (d, J = 18.5 Hz), 131.9 (d, J = 6.4 Hz), 131.2, 129.2, 128.6, 128.3, 128.1 (d, J = 5.8 Hz), 128.0 (d, J = 7.5 Hz), 127.7 (d, J = 15.6 Hz), 127.4 (d, J = 22.5 Hz), 127.1 (d, J = 2.3 Hz), 125.9 (d, J = 9.8 Hz), 124.0, 17.5 (d, J = 2.3 Hz), 13.8; **^{31}P NMR (202 MHz, CDCl$_3$)** δ -15.72; **IR (film)**: 3726, 3639, 3600, 2360, 1277 cm^{-1}; **HRMS m/z (ESI)**: calcd for $C_{32}H_{27}ClP$ $[M+H]^+$: 477.1533, found 477.1525.

The enantiomeric excess of **3ma** was determined by chiral HPLC analysis on Ciralpak IA column. Conditions: hexane/isopropanol = 99.5: 0.5 flow rate = 0.5 L/min, 25 °C, uv-vis detection at λ = 254 nm, 97% ee, t_{R1} = 6.0 min (minor), t_{R2} = 6.7 min (major); $[\alpha]_D^{25}$ = -74.35 (c = 2.27, CHCl$_3$).

(R,E)-(2-(2-(But-2-en-2-yl)naphthalen-1-yl)-4-(trifluoromethyl)phenyl)diphenylphosphane (3na)

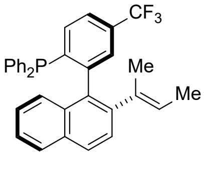

3na

General Procedure **A**, a 25.0 mL dry Schlenk tube with a stirring bar was added with $[Ir(cod)Cl]_2$ (3.4 mg, 0.01 mmol, 0.05 equiv) and **L3** (7.3 mg, 0.022 mmol, 0.11 equiv) in 2.0 ml toluene under

Ar. The formed mixture was stirred at room temperature for 1h. Then **1n** (91.2 mg, 0.2 mmol) and **2a** (54.0 mg, 1.0 mmol) was added under argon, the resulting mixture was stirred at 70 ºC. After 72 h, the solvent was removed under vaccum. The crude product purified by column chromatography on silica gel (PE/DCM = 10/1) to afford the corresponding product **3na** as a white solid (52.0 mg, 51%). **^{1}H NMR (500 MHz, $CDCl_3$)** δ 7.90 (d, *J* = 8.4 Hz, 1H), 7.83 (d, *J* = 8.3 Hz, 1H), 7.63 (d, *J* = 7.6 Hz, 2H), 7.52 (d, *J* = 7.4 Hz, 1H), 7.45 – 7.41 (m, 1H), 7.38 – 7.29 (m, 5H), 7.26 – 7.20 (m, 2H), 7.19 – 7.11 (m, 4H), 7.04 – 6.97 (m, 3H), 6.86 (d, *J* = 8.5 Hz, 1H), 5.41 (q, *J* = 6.8 Hz, 1H), 1.67 (s, 3H), 1.50 (d, *J* = 6.7 Hz, 3H); **^{13}C NMR (126 MHz, CDCl3)** δ 146.9 (d, *J* = 34.7 Hz), 143.5 (d, *J* = 15.6 Hz), 142.5, 137.3 (d, *J* = 13.3 Hz), 136.4 (d, *J* = 13.3 Hz), 136.0, 135.2 (d, *J* = 2.9 Hz), 134.5 (d, *J* = 6.4 Hz), 134.0 (d, *J* = 21.4 Hz), 132.9 (d, *J* = 18.5 Hz), 132.6 (d, *J* = 2.3 Hz), 131.9, 130.4 (q, *J* = 32.4 Hz), 128.7, 128.3 (d, *J* = 5.8 Hz), 128.24, 128.18, 128.1, 127.6, 127.1, 126.8 (d, *J* = 2.3 Hz), 126.3, 125.6, 125.0, 123.8 (q, *J* = 3.5 Hz), 17.8 (d, *J* = 2.3 Hz), 13.8; **^{31}P NMR (202 MHz, $CDCl_3$)** δ -15.87; **^{19}F NMR (471 MHz, $CDCl_3$)** δ -62.65; **IR (film)**: 3877, 3622, 2360, 1276 cm^{-1}; **HRMS m/z (ESI)**: calcd for $C_{33}H_{27}F_3P$ $[M+H]^+$: 511.1797, found 511.1787.

The enantiomeric excess of **3na** was determined by chiral HPLC analysis on Ciralpak IA column. Conditions: hexane/isopropanol = 99: 1 flow rate = 0.5 L/min, 25 ºC, uv-vis detection at λ = 220 nm, 98% ee, t_{R1} = 14.0 min (minor), t_{R2} = 15.3 min (major); $[\alpha]_D^{25}$ = -132.93 (*c* = 1.47, $CHCl_3$).

(R,E)-(2-(2-(But-2-en-2-yl)naphthalen-1-yl)-5-(trifluoromethyl)phenyl)diphenylphosphane (3oa)

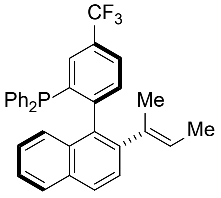

3oa

General Procedure **A**, a 25.0 mL dry Schlenk tube with a stirring bar was added with $[Ir(cod)Cl]_2$ (3.4 mg, 0.01 mmol, 0.05 equiv) and **L3** (7.3 mg, 0.022 mmol, 0.11 equiv) in 2.0 ml toluene under Ar. The formed mixture was stirred at room temperature for 1h. Then **1o** (91.2 mg, 0.2 mmol) and **2a** (54.0 mg, 1.0 mmol) was added under argon, the resulting mixture was stirred at 70 ºC. After 72 h, the solvent

was removed under vaccum. The crude product purified by column chromatography on silica gel (PE/DCM = 10/1) to afford the corresponding product **3oa** as a white solid (74.5 mg, 73%). **^{1}H NMR (500 MHz, $CDCl_3$)** δ 7.91 (d, *J* = 8.5 Hz, 1H), 7.85 (d, *J* = 8.1 Hz, 1H), 7.74 – 7.67 (m, 2H), 7.49 (dd, *J* = 7.8, 4.1 Hz, 1H), 7.45 (d, *J* = 8.4 Hz, 1H), 7.38 – 7.32 (m, 4H), 7.25 – 7.21 (m, 1H), 7.20 – 7.13 (m, 4H), 7.08 – 7.02 (m, 1H), 7.00 (td, *J* = 8.0, 1.5 Hz, 2H), 6.86 (d, *J* = 8.4 Hz, 1H), 5.44 (qd, *J* = 6.7, 1.5 Hz, 1H), 1.70 (t, *J* = 1.4 Hz, 3H), 1.52 (dd, *J* = 6.7, 1.4 Hz, 3H); **^{13}C NMR (126 MHz, CDCl3)** δ 150.2 (d, *J* = 34.1 Hz), 142.2 (d, *J* = 1.7 Hz), 140.2 (d, *J* = 16.2 Hz), 137.1 (d, *J* = 13.3 Hz), 136.5 (d, *J* = 13.9 Hz), 136.0, 134.6 (d, *J* = 6.4 Hz), 133.9 (d, *J* = 21.4 Hz), 132.7 (d, *J* = 17.9 Hz), 132.5 (d, *J* = 2.3 Hz), 132.6 (d, *J* = 5.2 Hz), 131.9, 131.3 (t, *J* = 3.2 Hz), 129.5 (q, *J* = 32.1 Hz), 128.7, 128.4 (d, *J* = 5.2 Hz), 128.2 (d, *J* = 7.5 Hz), 128.1, 127.4 (d, *J* = 60.7 Hz), 126.8 (d, *J* = 2.3 Hz), 126.3, 125.6, 125.01, 124.96, 124.1 (q, J = 272.6 Hz), 17.8 (d, *J* = 2.3 Hz), 13.9; **^{31}P NMR (202 MHz, $CDCl_3$)** δ -15.40; **^{19}F NMR (471 MHz, $CDCl_3$)** δ -62.30; **IR (film)**: 3828, 3601, 2360, 1276 cm^{-1}; **HRMS m/z (ESI)**: calcd for $C_{33}H_{27}F_3P$ $[M+H]^+$: 511.1797, found 511.1788.

The enantiomeric excess of **3oa** was determined by chiral HPLC analysis on Ciralpak IA column. Conditions: hexane/isopropanol = 99: 1 flow rate = 0.5 L/min, 25 °C, uv-vis detection at λ = 254 nm, 95% ee, t_{R1} = 8.9 min (minor), t_{R2} = 9.5 min (major); $[\alpha]_D^{25}$ = -84.11 (*c* = 1.95, $CHCl_3$).

(R,E)-(2-(4-(But-2-en-2-yl)-1,2-dihydroacenaphthylen-5-yl)phenyl)diphenylphosphane (3pa)

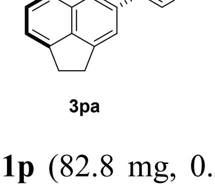

3pa

General Procedure **A**, a 25.0 mL dry Schlenk tube with a stirring bar was added with $[Ir(cod)Cl]_2$ (3.4 mg, 0.01 mmol, 0.05 equiv) and **L3** (7.3 mg, 0.022 mmol, 0.11 equiv) in 2.0 ml toluene under Ar. The formed mixture was stirred at room temperature for 1h. Then **1p** (82.8 mg, 0.2 mmol) and **2a** (54.0 mg, 1.0 mmol) was added under argon, the resulting mixture was stirred at 70 °C. After 72 h, the solvent was removed under vaccum. The crude product purified by column chromatography on silica gel

(PE/DCM = 10/1) to afford the corresponding product **3pa** as a white solid (71.1 mg, 76%). **^{1}H NMR (500 MHz, $CDCl_3$)** δ 7.46 – 7.41 (m, 2H), 7.40 – 7.34 (m, 1H), 7.34 – 7.30 (m, 3H), 7.25 – 7.21 (m, 4H), 7.19 (d, J = 6.1 Hz, 2H), 7.17 – 7.13 (m, 2H), 7.09 (t, J = 7.2 Hz, 2H), 6.80 (d, J = 8.2 Hz, 1H), 5.38 (q, J = 6.9 Hz, 1H), 3.47 (t, J = 9.1 Hz, 4H), 1.65 (s, 3H), 1.43 (d, J = 6.7 Hz, 3H); **^{13}C NMR (126 MHz, CDCl3)** δ 146.5 (d, J = 35.3 Hz), 145.3 (d, J = 14.4 Hz), 144.2 (d, J = 2.3 Hz), 138.7 (d, J = 14.4 Hz), 138.3 (d, J = 13.3 Hz), 137.7, 137.6 (d, J = 10.4 Hz), 136.9, 135.3 (d, J = 2.9 Hz), 133.5 (d, J = 20.8 Hz), 133.0 (d, J = 18.5 Hz), 132.0 (d, J = 6.4 Hz), 131.4 (d, J = 2.3 Hz), 128.5, 128.1, 128.00, 127.95 (d, J = 6.9 Hz), 127.6, 127.4, 127.1, 126.1 (d, J = 2.3 Hz), 121.8, 120.6, 118.5, 30.5, 30.2, 17.9, 13.8; **^{31}P NMR (202 MHz, $CDCl_3$)** δ -16.06; **IR (film)**: 3726, 3599, 2360, 1276 cm^{-1}; **HRMS m/z (ESI)**: calcd for $C_{34}H_{30}P$ $[M+H]^+$: 469.2080, found 469.2073.

The enantiomeric excess of **3pa** was determined by chiral HPLC analysis on Ciralpak IA column. Conditions: hexane/isopropanol = 99: 1 flow rate = 0.6 L/min, 25 °C, uv-vis detection at λ = 220 nm, 97% ee, t_{R1} = 7.8 min (minor), t_{R2} = 8.2 min (major); $[\alpha]_D^{25}$ = -21.82 (c = 0.50, $CHCl_3$).

(S,E)-(2-(10-(But-2-en-2-yl)phenanthren-9-yl)phenyl)diphenylphosphane (3qa)

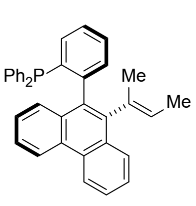

3qa

General Procedure **A**, a 25.0 mL dry Schlenk tube with a stirring bar was added with $[Ir(cod)Cl]_2$ (3.4 mg, 0.01 mmol, 0.05 equiv) and **L3** (7.3 mg, 0.022 mmol, 0.11 equiv) in 2.0 ml toluene under Ar. The formed mixture was stirred at room temperature for 1h. Then **1q** (87.6 mg, 0.2 mmol) and **2a** (54.0 mg, 1.0 mmol) was added under argon, the resulting mixture was stirred at 70 °C. After 72 h, the solvent was removed under vaccum. The crude product purified by column chromatography on silica gel (PE/DCM = 10/1) to afford the corresponding product **3qa** as a white solid (42.3 mg, 43%). **^{1}H NMR (500 MHz, $CDCl_3$)** δ 8.17 (d, J = 7.6 Hz, 1H), 8.13 – 8.08 (m, 3H), 8.08 – 8.04 (m, 1H), 7.96 (t, J = 7.6 Hz, 1H), 7.59 (d, J = 9.3 Hz, 1H), 7.52 – 7.43 (m, 4H), 7.34 – 7.28 (m, 3H), 7.24 – 7.15 (m, 4H), 7.07 (t, J = 7.6 Hz, 2H), 7.01 – 6.94 (m, 2H), 5.66 – 5.54 (m, 1H), 1.76 (s, 3H), 1.57 (dd, J = 6.7, 1.4 Hz, 3H); **^{13}C NMR (126

MHz, $CDCl_3$) δ 146.7 (d, *J* = 34.7 Hz), 143.5, 138.6 (d, *J* = 11.6 Hz), 138.4 (d, *J* = 13.3 Hz), 137.6 (d, *J* = 13.9 Hz), 137.2, 135.3 (d, *J* = 6.4 Hz), 135.0 (d, *J* = 2.9 Hz), 134.0 (d, *J* = 21.4 Hz), 132.9 (d, *J* = 17.9 Hz), 132.2 (d, *J* = 6.4 Hz), 131.3, 130.7 (d, *J* = 22.5 Hz), 130.0 (d, *J* = 2.9 Hz), 128.4 (d, *J* = 7.5 Hz), 128.2 (d, *J* = 5.2 Hz), 128.1 (d, *J* = 2.9 Hz), 127.7 (d, *J* = 8.7 Hz), 127.4 (d, *J* = 32.4 Hz), 127.0 (d, *J* = 2.9 Hz), 126.6, 126.1, 125.5 (d, *J* = 31.2 Hz), 124.8 (d, *J* = 19.7 Hz), 124.6, 123.4, 18.3 (d, *J* = 1.2 Hz), 14.1; **^{31}P NMR (202 MHz, $CDCl_3$)** δ -15.50; **IR (film)**: 3726, 3600, 2360, 1276 cm^{-1}; **HRMS m/z (ESI)**: calcd for $C_{36}H_{30}P$ $[M+H]^+$: 493.2080, found 493.2080. The enantiomeric excess of **3qa** was determined by chiral HPLC analysis on Ciralpak IA column. Conditions: hexane/isopropanol = 99: 1 flow rate = 0.5 L/min, 25 ºC, uv-vis detection at λ = 220 nm, 97% ee, t_{R1} = 6.8 min (minor), t_{R2} = 7.6 min (major); $[\alpha]_D^{25}$ = -78.80 (*c* = 1.5, $CHCl_3$).

(R,E)-(2-(2-(But-2-en-2-yl)pyren-1-yl)phenyl)diphenylphosphane (3ra)

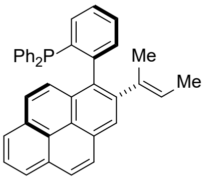

3ra

General Procedure **A**, a 25.0 mL dry Schlenk tube with a stirring bar was added with $[Ir(cod)Cl]_2$ (3.4 mg, 0.01 mmol, 0.05 equiv) and **L3** (7.3 mg, 0.022 mmol, 0.11 equiv) in 2.0 ml toluene under Ar. The formed mixture was stirred at room temperature for 1h. Then **1r** (92.4 mg, 0.2 mmol) and **2a** (54.0 mg, 1.0 mmol) was added under argon, the resulting mixture was stirred at 70 ºC. After 72 h, the solvent was removed under vaccum. The crude product purified by column chromatography on silica gel (PE/DCM = 10/1) to afford the corresponding product **3ra** as a white solid (60.9 mg, 59%). **^{1}H NMR (500 MHz, $CDCl_3$)** δ 8.21 – 8.17 (m, 2H), 8.16 – 8.09 (m, 3H), 8.00 (t, J = 7.6 Hz, 1H), 7.65 (d, J = 9.3 Hz, 1H), 7.58 – 7.55 (m, 1H), 7.54 – 7.50 (m, 3H), 7.38 – 7.33 (m, 3H), 7.31 – 7.27 (m, 2H), 7.25 – 7.20 (m, 2H), 7.12 (td, J = 7.6, 1.7 Hz, 2H), 7.05 (td, J = 7.9, 1.5 Hz, 2H), 5.68 (qd, J = 6.7, 1.5 Hz, 1H), 1.83 (s, 3H), 1.64 (dd, J = 6.8, 1.3 Hz, 3H).; **^{13}C NMR (101 MHz, $CDCl_3$)** δ 146.6 (d, *J* = 35.0 Hz), 143.5, 138.6 (d, *J* = 11.8 Hz), 138.4 (d, *J* = 13.2 Hz), 137.6 (d, *J* = 14.1 Hz), 137.1, 135.2 (d, *J* = 5.9 Hz), 134.9 (d, *J* = 2.7 Hz), 134.0 (d, *J* = 21.3 Hz), 132.8 (d, *J* = 18.2 Hz), 132.2 (d, *J* = 5.9 Hz), 131.2, 130.7 (d, *J* = 18.2 Hz), 130.0 (d, *J* = 2.7 Hz), 128.3

(d, J = 5.9 Hz), 128.1 (d, J = 6.4 Hz), 128.0 , 127.6 (d, J = 7.3 Hz), 127.5, 127.2, 126.9 (d, J = 2.7 Hz), 126.5, 126.0, 125.4 (d, J = 24.5 Hz), 124.7 (d, J = 15.9 Hz), 124.6, 123.3, 18.2, 14.0; **^{31}P NMR (202 MHz, CDCl$_3$)** δ -15.42; **IR (film)**: 3877, 3601, 2360, 1276 cm^{-1}; **HRMS m/z (ESI)**: calcd for $C_{38}H_{30}P$ [M+H]$^+$: 517.2080, found 517.2068.

The enantiomeric excess of **3ra** was determined by chiral HPLC analysis on Ciralpak IA column. Conditions: hexane/isopropanol = 99: 1 flow rate = 0.5 L/min, 25 °C, uv-vis detection at λ = 220 nm, 99% ee, t_{R1} = 6.8 min (minor), t_{R2} = 7.6 min (major); $[\alpha]_D^{25}$ = -141.39 (c = 0.72, CHCl$_3$).

(R,E)-(2-(But-2-en-2-yl)-[1,2'-binaphthalen]-3'-yl)diphenylphosphane (3sa)

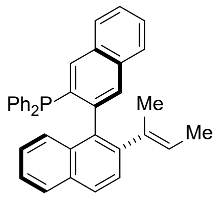

3sa

General Procedure **A**, a 25.0 mL dry Schlenk tube with a stirring bar was added with [Ir(cod)Cl]$_2$ (3.4 mg, 0.01 mmol, 0.05 equiv) and **L3** (7.3 mg, 0.022 mmol, 0.11 equiv) in 2.0 ml toluene under Ar. The formed mixture was stirred at room temperature for 1h. Then **1s** (87.6 mg, 0.2 mmol) and **2a** (54.0 mg, 1.0 mmol) was added under argon, the resulting mixture was stirred at 70 °C. After 72 h, the solvent was removed under vaccum. The crude product purified by column chromatography on silica gel (PE/DCM = 10/1) to afford the corresponding product **3sa** as a white solid (72.8 mg, 74%). **^{1}H NMR (500 MHz, CDCl$_3$)** δ 7.55 (d, J = 8.4 Hz, 1H), 7.50 – 7.42 (m, 5H), 7.35 (d, J = 8.4 Hz, 1H), 7.31 (t, J = 6.0 Hz, 3H), 7.28 (d, J = 7.9 Hz, 1H), 7.21 (td, J = 7.0, 2.2 Hz, 2H), 7.00 (t, J = 7.2 Hz, 1H), 6.94 – 6.83 (m, 5H), 6.24 (d, J = 7.8 Hz, 1H), 5.36 (q, J = 6.7 Hz, 1H), 1.63 (s, 3H), 1.41 (d, J = 6.9 Hz, 3H); **^{13}C NMR (126 MHz, CDCl$_3$)** δ 142.61 (d, J = 1.7 Hz), 142.58, 142.3, 138.3 (d, J = 13.3 Hz), 137.5 (d, J = 12.7 Hz), 137.2 (d, J = 13.9 Hz), 136.7, 135.6 (d, J = 5.8 Hz), 135.3 (d, J = 2.4 Hz), 134.1 (d, J = 22.0 Hz), 133.2 (d, J = 2.3 Hz), 133.1, 133.0 (d, J = 17.9 Hz), 132.5, 131.9, 130.2 (d, J = 6.4 Hz), 128.4, 128.2 (d, J = 5.2 Hz), 128.0, 127.8 (d, J = 42.2 Hz), 127.7 (d, J = 8.1 Hz), 127.3 (d, J = 13.9 Hz), 126.9 (d, J = 8.1 Hz), 126.1 (d, J = 2.9 Hz), 125.9, 125.2, 124.7, 18.0 (d, J = 2.3 Hz), 13.9; **^{31}P NMR (202 MHz, CDCl$_3$)** δ -14.67; **IR (film)**: 3726, 3622, 2360, 1276 cm^{-1}; **HRMS m/z**

(ESI): calcd for $C_{36}H_{30}P$ $[M+H]^+$: 493.2080, found 493.2072.

The enantiomeric excess of **3sa** was determined by chiral HPLC analysis on Ciralpak IA column. Conditions: hexane/isopropanol = 99: 1 flow rate = 1.0 L/min, 25 °C, uv-vis detection at λ = 220 nm, 99% ee, t_{R1} = 6.6 min (minor), t_{R2} = 7.8 min (major); $[\alpha]_D^{25}$ = -57.78 (c = 0.14, $CHCl_3$).

(S,E)-3-(But-2-en-2-yl)-4-(2-(diphenylphosphanyl)phenyl)quinoline (3ta)

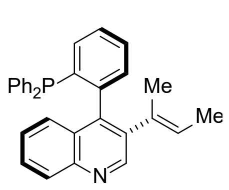

3ta

General Procedure **A**, a 25.0 mL dry Schlenk tube with a stirring bar was added with $[Ir(cod)Cl]_2$ (3.4 mg, 0.01 mmol, 0.05 equiv) and **L3** (7.3 mg, 0.022 mmol, 0.11 equiv) in 2.0 ml toluene under Ar. The formed mixture was stirred at room temperature for 1h. Then **1t** (77.8 mg, 0.2 mmol) and **2a** (54.0 mg, 1.0 mmol) was added under argon, the resulting mixture was stirred at 70 °C. After 72 h, the solvent was removed under vaccum. The crude product purified by column chromatography on silica gel (PE/DCM = 10/1) to afford the corresponding product **3ta** as a white solid (43.4 mg, 49%). **^{1}H NMR (500 MHz, $CDCl_3$)** δ 8.82 (s, 1H), 8.09 (d, J = 8.3 Hz, 1H), 7.54 (m, 1H), 7.48 – 7.42 (m, 1H), 7.40 (m, 2H), 7.29 (m, 4H), 7.21 – 7.16 (m, 1H), 7.16 – 7.06 (m, 5H), 7.01 – 6.94 (m, 3H), 5.50 (qd, J = 6.7, 1.5 Hz, 1H); **^{13}C NMR (126 MHz, $CDCl_3$)** δ 151.2, 146.6, 144.2 (d, J = 6.5 Hz), 143.6 (d, J = 34.7 Hz), 137.5 (d, J = 12.7 Hz), 137.3 (d, J = 13.9 Hz), 137.2 (d, J = 11.0 Hz), 137.1, 135.2 (d, J = 2.9 Hz), 133.7 (d, J = 20.8 Hz), 133.3, 132.8 (d, J = 18.5 Hz), 130.8 (d, J = 6.4 Hz), 128.9, 128.6, 128.4, 128.4 (d, J = 2.3 Hz), 128.23, 128.19, 128.14, 128.01 (d, J = 19.1 Hz), 127.7 (d, J = 1.7 Hz), 126.5, 125.9, 17.4 (d, J = 1.7 Hz), 14.0; **^{31}P NMR (202 MHz, $CDCl_3$)** δ -15.48; **IR (film)**: 3700, 3622, 2360, 1276 cm^{-1}; **HRMS m/z (ESI)**: calcd for $C_{31}H_{27}NP$ $[M+H]^+$: 444.1876, found 444.1864.

The enantiomeric excess of **3ta** was determined by chiral HPLC analysis on Ciralpak IA column. Conditions: hexane/isopropanol = 95: 5 flow rate = 1.0 L/min, 25 °C, uv-vis detection at λ = 220 nm, 97% ee, t_{R1} = 5.8 min (minor), t_{R2} = 6.2 min (major); $[\alpha]_D^{25}$ = -96.4 (c = 1.5, $CHCl_3$).

(R,E)-(2-(2-(But-2-en-2-yl)dibenzo[b,d]furan-1-yl)phenyl)diphenylphosphane (3ua)

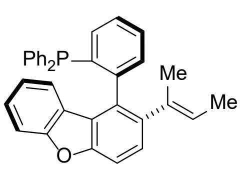

3ua

General Procedure **A**, a 25.0 mL dry Schlenk tube with a stirring bar was added with $[Ir(cod)Cl]_2$ (3.4 mg, 0.01 mmol, 0.05 equiv) and **L3** (7.3 mg, 0.022 mmol, 0.11 equiv) in 2.0 ml toluene under Ar. The formed mixture was stirred at room temperature for 1h. Then **1u** (85.6 mg, 0.2 mmol) and **2a** (54.0 mg, 1.0 mmol) was added under argon, the resulting mixture was stirred at 70 ºC. After 72 h, the solvent was removed under vaccum. The crude product purified by column chromatography on silica gel (PE/DCM = 10/1) to afford the corresponding product **3ua** as a white solid (81.0 mg, 84%). **^{1}H NMR (500 MHz, $CDCl_3$)** δ 7.55 (d, J = 8.4 Hz, 1H), 7.50 – 7.42 (m, 5H), 7.35 (d, J = 8.4 Hz, 1H), 7.31 (t, J = 6.0 Hz, 3H), 7.28 (d, J = 7.9 Hz, 1H), 7.21 (td, J = 7.0, 2.2 Hz, 2H), 7.00 (t, J = 7.2 Hz, 1H), 6.94 – 6.83 (m, 5H), 6.24 (d, J = 7.8 Hz, 1H), 5.36 (q, J = 6.7 Hz, 1H), 1.63 (s, 3H), 1.41 (d, J = 6.9 Hz, 3H); **^{13}C NMR (126 MHz, $CDCl_3$)** δ 156.4, 154.3, 145.7 (d, J = 34.7 Hz), 140.0 (d, J = 2.3 Hz), 137.7, 137.6, 137.4 (d, J = 12.7 Hz), 135.4, 135.1 (d, J = 2.9 Hz), 134.3 (d, J = 6.9 Hz), 133.7 (d, J = 21.4 Hz), 133.0 (d, J = 18.5 Hz), 130.8 (d, J = 5.8 Hz), 128.8, 128.1 (d, J = 5.2 Hz), 128.0 (d, J = 17.9 Hz), 127.8 (d, J = 3.4 Hz), 127.7, 126.6 (d, J = 2.3 Hz), 126.3, 124.5, 123.5, 122.0 (d, J = 38.1 Hz), 110.7 (d, J = 76.3 Hz), 18.1 (d, J = 2.3 Hz), 13.9; **^{31}P NMR (202 MHz, $CDCl_3$)** δ -15.33; **IR (film)**: 3700, 3499, 3055, 2360, 1276 cm^{-1}; **HRMS m/z (ESI)**: calcd for $C_{34}H_{28}OP$ $[M+H]^+$: 483.1872, found 483.1864.

The enantiomeric excess of **3ua** was determined by chiral HPLC analysis on Ciralpak IA column. Conditions: hexane/isopropanol = 99: 1 flow rate = 0.5 L/min, 25 ºC, uv-vis detection at λ = 220 nm, 98% ee, t_{R1} = 6.0 min (minor), t_{R2} = 6.5 min (major); $[\alpha]_D^{25}$ = -145.45 (c = 0.66, $CHCl_3$).

(R,E)-1-(2-(But-2-en-2-yl)naphthalen-1-yl)-2-(diphenylphosphanyl)-1H-pyrrole (3va)

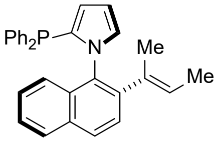

3va

General Procedure **A**, a 25.0 mL dry Schlenk tube with a stirring bar was added with $[Ir(cod)Cl]_2$ (3.4 mg, 0.01 mmol, 0.05 equiv) and **L3** (7.3 mg, 0.022 mmol, 0.11 equiv) in 2.0 ml toluene under Ar. The formed mixture was stirred at room temperature for 1h. Then **1v** (75.4 mg, 0.2 mmol) and **2a** (54.0 mg, 1.0 mmol) was added under argon, the resulting mixture was stirred at 70 °C. After 72 h, the solvent was removed under vaccum. The crude product purified by column chromatography on silica gel (PE/DCM = 10/1) to afford the corresponding product **3va** as a pale yellow solid (59.5 mg, 69%). **^{1}H NMR (500 MHz, $CDCl_3$)** δ 7.87 (dd, J = 18.8, 8.3 Hz, 2H), 7.47 – 7.41 (m, 2H), 7.39 – 7.35 (m, 2H), 7.32 (d, J = 7.0 Hz, 3H), 7.25 – 7.14 (m, 6H), 7.04 (s, 1H), 6.97 (d, J = 8.5 Hz, 1H), 6.46 (d, J = 26.2 Hz, 2H), 5.49 (q, J = 6.9 Hz, 1H), 1.59 (s, 3H), 1.44 (d, J = 6.9 Hz, 3H); **^{13}C NMR (126 MHz, $CDCl_3$)** δ 141.6, 138.8 (d, J = 6.9 Hz), 137.8 (d, J = 6.4 Hz), 134.2, 133.1 (d, J = 20.2 Hz), 132.9 (d, J = 19.1 Hz), 132.5, 132.3 (d, J = 2.3 Hz), 131.0 (d, J = 3.5 Hz), 128.5, 127.99 (d, J = 3.5 Hz), 127.96, 127.9 (d, J = 6.4 Hz), 127.3 (d, J = 26.5 Hz), 126.7 (d, J = 17.3 Hz), 125.7, 123.5, 118.9 (d, J = 3.5 Hz), 109.7, 16.4, 13.8; **^{31}P NMR (202 MHz, $CDCl_3$)** δ -33.62; **IR (film)**: 3699, 3576, 2360, 1276 cm^{-1}; **HRMS m/z (ESI)**: calcd for $C_{30}H_{27}NP$ $[M+H]^+$: 432.1876, found 432.1865.

The enantiomeric excess of **3va** was determined by chiral HPLC analysis on Ciralpak IA column. Conditions: hexane/isopropanol = 99: 1 flow rate = 0.8 L/min, 25 °C, uv-vis detection at λ = 220 nm, 96% ee, t_{R1} = 5.2 min (minor), t_{R2} = 5.9 min (major); $[\alpha]_D^{25}$ = -41.84 (c = 0.44, $CHCl_3$).

(R,E)-(2-(2-(But-2-en-2-yl)naphthalen-1-yl)phenyl)bis(4-fluorophenyl)phosphane (3wa)

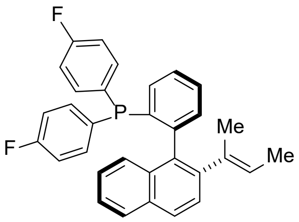

3wa

General Procedure **A**, a 25.0 mL dry Schlenk tube with a stirring bar was added with $[Ir(cod)Cl]_2$ (3.4 mg, 0.01 mmol, 0.05 equiv) and **L3** (7.3 mg, 0.022 mmol, 0.11 equiv) in 2.0 ml toluene under Ar. The formed mixture was stirred at

room temperature for 1h. Then **1w** (84.8 mg, 0.2 mmol) and **2a** (54.0 mg, 1.0 mmol) was added under argon, the resulting mixture was stirred at 70 °C. After 72 h, the solvent was removed under vaccum. The crude product purified by column chromatography on silica gel (PE/DCM = 10/1) to afford the corresponding product **3wa** as a white solid (49.7 mg, 52%). **^{1}H NMR (500 MHz, CDCl$_3$)** δ 7.85 (d, J = 8.4 Hz, 1H), 7.80 (d, J = 8.2 Hz, 1H), 7.45 (t, J = 7.4 Hz, 1H), 7.39 (t, J = 8.4 Hz, 2H), 7.36 – 7.28 (m, 3H), 7.14 – 7.05 (m, 2H), 7.04 – 6.97 (m, 3H), 6.92 (q, J = 7.4, 6.6 Hz, 2H), 6.87 (d, J = 8.5 Hz, 1H), 6.81 (t, J = 8.6 Hz, 2H), 5.41 (q, J = 6.8 Hz, 1H), 1.64 (s, 3H), 1.48 (d, J = 6.7 Hz, 3H); **^{13}C NMR (126 MHz, CDCl$_3$)** δ 163.9 (d, J = 48.6 Hz), 162.0 (d, J = 48.0 Hz), 146.2 (d, J = 34.1 Hz), 142.3 (d, J = 2.3 Hz), 137.9 (d, J = 11.6 Hz), 136.5, 135.8 (d, J = 8.1 Hz), 135.6 (d, J = 7.5 Hz), 134.6 (d, J = 7.5 Hz), 134.5 (d, J = 7.5 Hz), 134.3 (d, J = 2.9 Hz), 133.5 (dd, J = 13.6, 3.8 Hz), 133.0 (dd, J = 14.0, 3.0 Hz), 132.9 (d, J = 2.3 Hz), 132.0 (d, J = 6.4 Hz), 131.9, 128.6, 127.7, 127.6, 127.2, 126.6, 126.2 (d, J = 2.9 Hz), 125.1 (d, J = 54.9 Hz), 115.4 (dd, J = 16.5, 4.3 Hz), 115.3, 115.2 (d, J = 8.1 Hz), 17.7 (d, J = 2.3 Hz), 13.9; **^{19}F NMR (471 MHz, CDCl$_3$)** δ -112.90 (d, J = 4.3 Hz, 1F), -113.78 (d, J = 5.2 Hz, 1F); **^{31}P NMR (202 MHz, CDCl$_3$)** δ -17.63; **IR (film)**: 3887, 3561, 3071, 1276 cm^{-1}; **HRMS m/z (ESI)**: calcd for $C_{32}H_{26}F_2P$ $[M+H]^+$: 479.1735, found 479.1725.

The enantiomeric excess of **3wa** was determined by chiral HPLC analysis on Ciralpak IA column. Conditions: hexane/isopropanol = 99: 1 flow rate = 0.5 L/min, 25 °C, uv-vis detection at λ = 220 nm, 97% ee, t_{R1} = 6.0 min (minor), t_{R2} = 6.3 min (major); $[\alpha]_D^{25}$ = -92.94 (c = 1.1, CHCl$_3$).

(R,E)-(2-(2-(But-2-en-2-yl)naphthalen-1-yl)phenyl)di(thiophen-2-yl)phosphane (3xa)

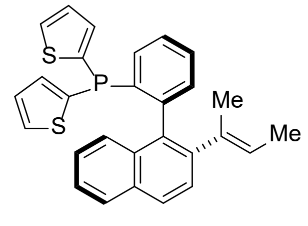

3xa

General Procedure **A**, a 25.0 mL dry Schlenk tube with a stirring bar was added with [Ir(cod)Cl]$_2$ (3.4 mg, 0.01 mmol, 0.05 equiv) and **L3** (7.3 mg, 0.022 mmol, 0.11 equiv) in 2.0 ml toluene under Ar. The formed mixture was stirred at

room temperature for 1h. Then **1x** (80.0 mg, 0.2 mmol) and **2a** (54.0 mg, 1.0 mmol) was added under argon, the resulting mixture was stirred at 70 °C. After 72 h, the solvent was removed under vaccum. The crude product purified by column chromatography on silica gel (PE/DCM = 10/1) to afford the corresponding product **3xa** as a white solid (67.2 mg, 74%). **^{1}H NMR (500 MHz, $CDCl_3$)** δ 7.87 (d, J = 8.4 Hz, 1H), 7.82 (d, J = 8.2 Hz, 1H), 7.73 (dt, J = 7.0, 3.9 Hz, 1H), 7.56 – 7.51 (m, 1H), 7.49 – 7.41 (m, 2H), 7.41 (d, J = 5.5 Hz, 1H), 7.36 – 7.29 (m, 1H), 7.32 – 7.25 (m, 1H), 7.11 – 7.05 (m, 2H), 7.08 – 7.01 (m, 1H), 7.00 (d, J = 8.4 Hz, 1H), 6.86 – 6.80 (m, 1H), 6.74 – 6.68 (m, 1H), 5.44 (qd, J = 6.8, 1.5 Hz, 1H), 1.65 (s, 3H), 1.50 (d, J = 6.7 Hz, 3H); **^{13}C NMR (126 MHz, $CDCl_3$)** δ 145.0 (d, J = 37.0 Hz), 142.4, 139.2 (d, J = 26.0 Hz), 138.8 (d, J = 17.3 Hz), 138.7 (d, J = 2.9 Hz), 136.5, 135.5 (d, J = 7.5 Hz), 135.1 (d, J = 27.7 Hz), 134.5 (d, J = 22.5 Hz), 133.6 (d, J = 2.9 Hz), 133.0 (d, J = 2.3 Hz), 131.8, 131.5 (d, J = 6.4 Hz), 131.2, 130.8 (d, J = 2.3 Hz), 128.9, 127.8 (d, J = 6.4 Hz), 127.7, 127.5 (d, J = 9.8 Hz), 127.4 (d, J = 8.1 Hz), 127.2, 126.4, 126.2 (d, J = 2.9 Hz), 125.4, 124.8, 17.7 (d, J = 2.3 Hz), 13.8; **^{31}P NMR (202 MHz, $CDCl_3$)** δ -41.90; **IR (film)**: 3877, 3600, 3100, 2360, 1276 cm^{-1}; **HRMS m/z (ESI)**: calcd for $C_{28}H_{24}PS_2$ $[M+H]^+$: 455.1052, found 455.1042.

The enantiomeric excess of **3xa** was determined by chiral HPLC analysis on Ciralpak IA column. Conditions: hexane/isopropanol = 98: 2 flow rate = 1.0 L/min, 25 °C, uv-vis detection at λ = 220 nm, 95% ee, t_{R1} = 4.3 min (minor), t_{R2} = 4.9 min (major); $[\alpha]_D^{25}$ = -80.75 (c = 0.8, $CHCl_3$).

(R,E)-(2-(2-(Hex-3-en-3-yl)naphthalen-1-yl)phenyl)diphenylphosphane (3ab)

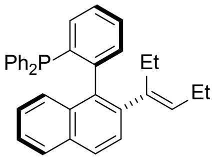

3ab

General Procedure **A**, a 25.0 mL dry Schlenk tube with a stirring bar was added with $[Ir(cod)Cl]_2$ (3.4 mg, 0.01 mmol, 0.05 equiv) and **L3** (7.3 mg, 0.022 mmol, 0.11 equiv) in 2.0 ml toluene under Ar. The formed mixture was stirred at room temperature for 1h. Then **1a** (77.6 mg, 0.2 mmol) and 3-hexyne **2b** (82.0 mg, 1.0 mmol) was added under argon, the resulting mixture was stirred at 75 °C. After 72 h, the solvent was removed under vaccum. The crude product purified by column chromatography on silica gel

(PE/DCM = 10/1) to afford the corresponding product **3ab** as a white solid (93.1 mg, 99%). **^{1}H NMR (500 MHz, CDCl$_3$)** δ 7.86 (d, *J* = 8.4 Hz, 1H), 7.81 (d, *J* = 7.9 Hz, 1H), 7.46 – 7.42 (m, 2H), 7.42 – 7.37 (m, 2H), 7.35 – 7.31 (m, 1H), 7.31 – 7.26 (m, 4H), 7.23 – 7.16 (m, 3H), 7.13 (td, *J* = 7.4, 1.6 Hz, 2H), 7.02 (td, *J* = 7.9, 1.5 Hz, 2H), 6.95 – 6.88 (m, 2H), 5.35 (t, *J* = 7.2 Hz, 1H), 2.21 – 2.11 (m, 1H), 2.07 – 1.96 (m, 2H), 1.87 – 1.76 (m, 1H), 0.86 (t, *J* = 7.6 Hz, 3H), 0.81 (t, *J* = 7.5 Hz, 3H); **^{13}C NMR (126 MHz, CDCl$_3$)** δ 146.3 (d, *J* = 35.3 Hz), 141.6, 140.6 (d, *J* = 1.7 Hz), 138.5 (d, *J* = 13.3 Hz), 138.3 (d, *J* = 12.1 Hz), 137.7 (d, *J* = 13.3 Hz), 136.3 (d, *J* = 6.9 Hz), 134.9 (d, *J* = 2.3 Hz), 134.0 (d, *J* = 20.8 Hz), 133.4 (d, *J* = 2.3 Hz), 133.0, 132.9, 132.8, 131.9, 131.5 (d, *J* = 6.4 Hz), 128.5, 128.4, 128.3, 128.1, 128.0, 127.9, 127.6, 127.4 (d, *J* = 10.4 Hz), 127.3, 126.9, 124.9 (d, *J* = 41.6 Hz), 24.2, 21.2, 14.2, 13.5; **^{31}P NMR (202 MHz, CDCl$_3$)** δ -15.48; **IR (film)**: 3877, 3599, 2360, 1276 cm^{-1}; **HRMS m/z (ESI)**: calcd for $C_{34}H_{32}P$ $[M+H]^+$: 471.2236, found 471.2225.

The enantiomeric excess of **3ab** was determined by chiral HPLC analysis on Ciralpak IA column. Conditions: hexane/isopropanol = 99: 1 flow rate = 0.5 L/min, 25 °C, uv-vis detection at λ = 254 nm, 91% ee, t_{R1} = 7.4 min (minor), t_{R2} = 8.0 min (major); $[\alpha]_D^{25}$ = -97.84 (*c* = 0.74, CHCl$_3$).

(R,E)-(2-(2-(Oct-4-en-4-yl)naphthalen-1-yl)phenyl)diphenylphosphane (3ac)

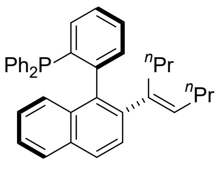

3ac

General Procedure **A**, a 25.0 mL dry Schlenk tube with a stirring bar was added with $[Ir(cod)Cl]_2$ (3.4 mg, 0.01 mmol, 0.05 equiv) and **L3** (7.3 mg, 0.022 mmol, 0.11 equiv) in 2.0 ml toluene under Ar. The formed mixture was stirred at room temperature for 1h. Then **1a** (77.6 mg, 0.2 mmol) and 4-octyne **2c** (110.2 mg, 1.0 mmol) was added under argon, the resulting mixture was stirred at 90 °C. After 72 h, the solvent was removed under vaccum. The crude product purified by column chromatography on silica gel (PE/DCM = 10/1) to afford the corresponding product **3ac** as a white solid (52.8 mg, 53%). **^{1}H NMR (500 MHz, CDCl$_3$)** δ 8.82 (s, 1H), 8.09 (d, J = 8.3 Hz, 1H), 7.54 (m, 1H), 7.48 – 7.42 (m, 1H), 7.40 (m, 2H), 7.29 (m, 4H), 7.21 – 7.16 (m, 1H), 7.16 – 7.06 (m, 5H), 7.01 – 6.94 (m, 3H), 5.50 (qd, J = 6.7, 1.5 Hz, 1H); **^{13}C NMR (126**

MHz, $CDCl_3$) δ 151.2, 146.6, 144.2 (d, *J* = 6.5 Hz), 143.6 (d, *J* = 34.7 Hz), 137.5 (d, *J* = 12.7 Hz), 137.3 (d, *J* = 13.9 Hz), 137.2 (d, *J* = 11.0 Hz), 137.1, 135.2 (d, *J* = 2.9 Hz), 133.7 (d, *J* = 20.8 Hz), 133.3, 132.8 (d, *J* = 18.5 Hz), 130.8 (d, *J* = 6.4 Hz), 128.9, 128.6, 128.4, 128.4 (d, *J* = 2.3 Hz), 128.2, 128.2, 128.13, 128.08, 127.9, 127.7 (d, *J* = 1.7 Hz), 126.5, 125.9, 17.4 (d, *J* = 1.7 Hz), 14.0; **^{31}P NMR (202 MHz, $CDCl_3$)** δ -15.48; **IR (film)**: 3700, 3622, 2360, 1276 cm^{-1}; **HRMS m/z (ESI)**: calcd for $C_{36}H_{36}P$ $[M+H]^+$: 499.2549, found 499.2539.

The enantiomeric excess of **3ac** was determined by chiral HPLC analysis on Ciralpak IA column. Conditions: hexane/isopropanol = 99.5: 0.5 flow rate = 0.8 L/min, 25 °C, uv-vis detection at λ = 220 nm, 85% ee, t_{R1} = 4.6 min (minor), t_{R2} = 4.8 min (major); $[\alpha]_D^{25}$ = -61.89 (*c* = 1.06, $CHCl_3$).

(R,E)-(2-(2-(2,9-Dimethyldec-5-en-5-yl)naphthalen-1-yl)phenyl)diphenylphosphane (3ad)

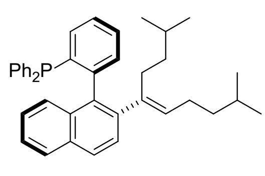

3ad

General Procedure **A**, a 25.0 mL dry Schlenk tube with a stirring bar was added with $[Ir(cod)Cl]_2$ (3.4 mg, 0.01 mmol, 0.05 equiv) and **L3** (7.3 mg, 0.022 mmol, 0.11 equiv) in 2.0 ml toluene under Ar. The formed mixture was stirred at room temperature for 1h. Then **1a** (77.6 mg, 0.2 mmol) and 2,9-dimethyldec-5-yne **2d** (166.1 mg, 1.0 mmol) was added under argon, the resulting mixture was stirred at 75 °C. After 72 h, the solvent was removed under vaccum. The crude product purified by column chromatography on silica gel (PE/DCM = 10/1) to afford the corresponding product **3ad** as a white solid (36.6 mg, 33%). **^{1}H NMR (500 MHz, $CDCl_3$)** δ 7.85 (d, *J* = 8.4 Hz, 1H), 7.82 (d, *J* = 8.1 Hz, 1H), 7.46 – 7.41 (m, 2H), 7.41 – 7.37 (m, 2H), 7.35 – 7.32 (m, 1H), 7.31 (d, *J* = 7.6 Hz, 1H), 7.28 (d, J = 3.4 Hz, 3H), 7.21 – 7.15 (m, 3H), 7.11 (t, *J* = 7.5 Hz, 2H), 7.01 – 6.94 (m, 3H), 6.88 (d, *J* = 8.4 Hz, 1H), 5.34 (t, *J* = 7.2 Hz, 1H), 2.21 – 2.10 (m, 1H), 2.05 – 1.96 (m, 1H), 1.97 – 1.88 (m, 1H), 1.77 – 1.68 (m, 1H), 1.46 – 1.38 (m, 2H), 1.16 (q, *J* = 7.2 Hz, 2H), 1.08 – 1.01 (m, 2H), 0.85 (t, *J* = 5.9 Hz, 6H), 0.80 (dd, *J* = 6.7, 2.0 Hz, 6H); **^{13}C NMR (126 MHz, $CDCl_3$)** δ 146.3 (d, *J* = 35.3 Hz), 141.0, 140.7, 138.6 (d, *J* = 13.9 Hz), 138.3 (d, *J* = 12.1 Hz),

137.9 (d, J = 13.9 Hz), 136.1 (d, J = 6.4 Hz), 135.0 (d, J = 2.9 Hz), 134.0 (d, J = 21.4 Hz), 133.0 (d, J = 2.2 Hz), 132.8 (d, J = 17.3 Hz), 132.5 (d, J = 2.9 Hz), 131.9, 131.5 (d, J = 6.4 Hz), 128.4 (d, J = 13.9 Hz), 128.2, 128.03, 127.98, 127.9, 127.6, 127.4 (d, J = 6.9 Hz), 127.3, 126.9, 124.9 (d, J = 48.6 Hz), 38.8, 37.9, 29.2, 28.1, 27.6, 26.0, 22.7, 22.6, 22.5, 22.3; **^{31}P NMR (202 MHz, $CDCl_3$)** δ -15.45; **IR (film)**: 3700, 3661, 2360, 1276 cm^{-1}; **HRMS m/z (ESI)**: calcd for $C_{40}H_{44}P$ $[M+H]^+$: 555.3175, found 555.3165.

The enantiomeric excess of **3ad** was determined by chiral HPLC analysis on Ciralpak IA column. Conditions: hexane/isopropanol = 99.9: 0.1 flow rate = 0.8 L/min, 25 °C, uv-vis detection at λ = 220 nm, 93% ee, t_{R1} = 5.8 min (minor), t_{R2} = 6.3 min (major); $[\alpha]_D^{25}$ = -52.25 (c = 1.11, $CHCl_3$).

(R,E)-Diphenyl(2-(2-(1-phenylprop-1-en-2-yl)naphthalen-1-yl)phenyl)phosphane e (3ae)

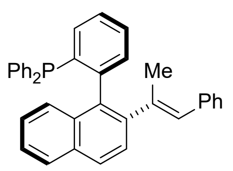

3ae

General Procedure **A**, a 25.0 mL dry Schlenk tube with a stirring bar was added with $[Ir(cod)Cl]_2$ (3.4 mg, 0.01 mmol, 0.05 equiv) and **L3** (7.3 mg, 0.022 mmol, 0.11 equiv) in 2.0 ml toluene under Ar. The formed mixture was stirred at room temperature for 1h. Then **1a** (77.6 mg, 0.2 mmol) and prop-1-yn-1-ylbenzene **2e** (116.2 mg, 1.0 mmol) was added under argon, the resulting mixture was stirred at 90 °C. After 72 h, the solvent was removed under vaccum. The crude product purified by column chromatography on silica gel (PE/DCM = 10/1) to afford the corresponding product **3ae** as a white solid (32.3 mg, 32%). **^{1}H NMR (500 MHz, $CDCl_3$)** δ 7.89 (d, J = 8.6 Hz, 1H), 7.80 (d, J = 8.4 Hz, 1H), 7.52 (d, J = 8.4 Hz, 1H), 7.46 – 7.42 (m, 1H), 7.41 – 7.38 (m, 1H), 7.38 – 7.33 (m, 2H), 7.33 – 7.29 (m, 1H), 7.29 – 7.21 (m, 3H), 7.20 – 7.15 (m, 2H), 7.14 – 7.07 (m, 3H), 7.06 – 7.02 (m, 4H), 7.02 – 7.00 (m, 2H), 6.98 (d, J = 1.3 Hz, 1H), 6.98 – 6.95 (m, 2H), 6.44 (s, 1H), 1.90 (d, J = 1.3 Hz, 3H); **^{13}C NMR (101 MHz, $CDCl_3$)** δ 146.0 (d, J = 34.5 Hz), 142.0, 138.5, 138.3 (d, J = 12.3 Hz), 138.1, 138.0 (d, J = 13.1 Hz), 137.4 (d, J = 13.2 Hz), 136.3, 135.0 (d, J = 2.3 Hz), 134.0 (d, J = 21.3 Hz), 132.9 (d, J = 18.2 Hz), 132.1, 132.0 (d, J = 6.4 Hz), 131.7 (d, J

= 2.7 Hz), 128.9, 128.4, 128.3, 128.1 (d, J = 1.3 Hz), 128.0 (d, J = 3.2 Hz), 127.9, 127.8, 127.6, 127.5 (d, J = 3.6 Hz), 127.1, 126.4 (d, J = 48.6 Hz), 125.2 (d, J = 31.3 Hz), 20.0 (d, J = 2.3 Hz); **^{31}P NMR (162 MHz, $CDCl_3$)** δ -15.29; **IR (film)**: 3622, 2360, 1276 cm^{-1}; **HRMS m/z (ESI)**: calcd for $C_{37}H_{30}P$ $[M+H]^+$: 505.2080, found 505.2070.

The enantiomeric excess of **3ae** was determined by chiral HPLC analysis on Ciralpak IA column. Conditions: hexane/isopropanol = 99: 1 flow rate = 0.5 L/min, 25 °C, uv-vis detection at λ = 220 nm, 89% ee, t_{R1} = 7.9 min (major), t_{R2} = 9.0 min (minor); $[\alpha]_D^{25}$ = -15.84 (c = 0.75, $CHCl_3$).

2.3.2 asymmetric C–H hydroarylation with alkenes

General Procedure B:

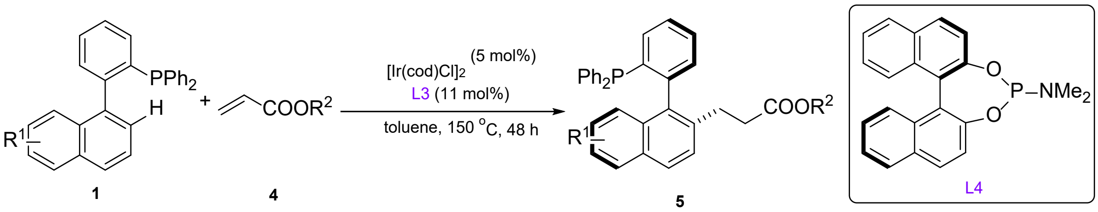

a 25.0 mL dry Schlenk tube with a stirring bar was added with $[Ir(cod)Cl]_2$ (3.4 mg, 0.01 mmol, 0.05 equiv) and **L4** (8.0 mg, 0.022 mmol, 0.11 equiv) in 2.0 ml toluene under Ar. The formed mixture was stirred at room temperature for 1h. Then **1** (0.2 mmol, 1 equiv) and **4** (1.0 mmol, 5 equiv) was added under argon, the resulting mixture was stirred at 150 °C. After 48 h, the solvent was removed under vaccum. The crude product purified by column chromatography on silica gel (300-400 mesh, PE/EtOAc as eluent) to afford the corresponding product **5**.

Synthetic route of Racemic **5**

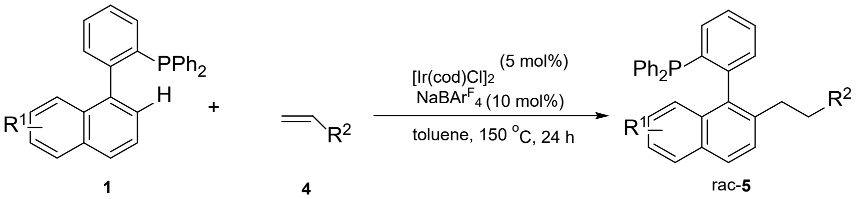

A 25.0 mL dry Schlenk tube with a stirring bar was added with $[Ir(cod)Cl]_2$ (3.4 mg, 0.01 mmol, 0.05 equiv) and $NaBAr^F_4$ (17.7 mg, 0.02 mmol, 0.11 equiv) in 2.0 ml

toluene under Ar. The formed mixture was stirred at room temperature for 1h. Then **1** (0.2 mmol, 1 equiv) and **4** (1.0 mmol, 5 equiv) was added under argon, the resulting mixture was stirred at 150 °C. After 24 h, the solvent was removed under vaccum. The crude product purified by column chromatography on silica gel (300-400 mesh, PE/DCM or PE/EtOAc as eluent) to afford the corresponding racemic product **5**.

Ethyl (R)-3-(1-(2-(diphenylphosphanyl)phenyl)naphthalen-2-yl)propanoate (5aa)

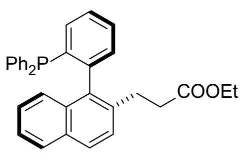

5aa

General Procedure **B**, a 25.0 mL dry Schlenk tube with a stirring bar was added with $[Ir(cod)Cl]_2$ (3.4 mg, 0.01 mmol, 0.05 equiv) and **L4** (8.0 mg, 0.022 mmol, 0.11 equiv) in 2.0 ml toluene under Ar. The formed mixture was stirred at room temperature for 1h. Then **1a** (77.6 mg, 0.2 mmol) and **4a** (ethyl acrylate, 100.0 mg, 1.0 mmol) was added under argon, the resulting mixture was stirred at 150 °C. After 48 h, the solvent was removed under vaccum. The crude product purified by column chromatography on silica gel (PE/EtOAc = 20/1) to afford the corresponding product **5aa** as a white solid (75.2 mg, 77%). **^{1}H NMR (400 MHz, $CDCl_3$)** δ 7.78 (d, J = 8.5 Hz, 1H), 7.74 (d, J = 7.4 Hz, 1H), 7.43 (td, J = 7.1, 2.1 Hz, 1H), 7.39 – 7.31 (m, 3H), 7.30 – 7.21 (m, 5H), 7.21 – 7.16 (m, 2H), 7.12 – 7.05 (m, 4H), 7.04 – 6.95 (m, 3H), 4.02 (q, J = 7.2 Hz, 2H), 2.67 – 2.50 (m, 2H), 2.49 – 2.28 (m, 2H), 1.15 (t, J = 7.2 Hz, 3H); **^{13}C NMR (101 MHz, $CDCl_3$)** δ 172.7, 145.0 (d, J = 33.2 Hz), 138.3 (d, J = 12.3 Hz), 137.3 (d, J = 6.8 Hz), 136.8 (d, J = 12.3 Hz), 136.0, 134.2, 133.7 (d, J = 3.2 Hz), 133.5 (d, J = 4.1 Hz), 132.9, 131.7, 130.7 (d, J = 5.9 Hz), 129.0, 128.3, 128.2, 128.0 (d, J = 3.2 Hz), 127.9, 127.6 (d, J = 27.7 Hz), 126.4, 125.5, 124.8, 60.1, 35.0, 28.8, 14.1; **^{31}P NMR (162 MHz, $CDCl_3$)** δ -15.39; **IR (film)**: 3911, 3758, 3756, 2360, 1277 cm^{-1}; **HRMS m/z (ESI)**: calcd for $C_{33}H_{30}O_2P$ $[M+H]^+$: 489.1978, found 489.1967;

The enantiomeric excess of **5aa** was determined by chiral HPLC analysis on Ciralpak IB column. Conditions: hexane/isopropanol = 99: 1 flow rate = 0.5 L/min, 25 °C, uv-vis detection at λ = 220 nm, 91% ee, t_{R1} = 13.4 min (major), t_{R2} = 14.5 min (minor); $[\alpha]_D^{25}$ = -27.08 (c = 16.3, $CHCl_3$).

Ethyl (R)-3-(1-(2-(diphenylphosphanyl)-5-methylphenyl)naphthalen-2-yl)propanoate (5ba)

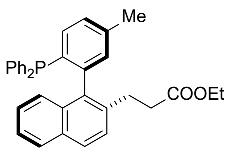

5ba

General Procedure **B**, a 25.0 mL dry Schlenk tube with a stirring bar was added with $[Ir(cod)Cl]_2$ (3.4 mg, 0.01 mmol, 0.05 equiv) and **L4** (8.0 mg, 0.022 mmol, 0.11 equiv) in 2.0 ml toluene under Ar. The formed mixture was stirred at room temperature for 1h. Then **1b** (80.4 mg, 0.2 mmol) and **4a** (100.0 mg, 1.0 mmol) was added under argon, the resulting mixture was stirred at 150 °C. After 48 h, the solvent was removed under vaccum. The crude product purified by column chromatography on silica gel (PE/EtOAc = 20/1) to afford the corresponding product **5ba** as a white solid (64.3 mg, 64%). **^{1}H NMR (500 MHz, $CDCl_3$)** δ 7.80 (d, J = 8.4 Hz, 1H), 7.76 (dd, J = 8.2, 1.7 Hz, 1H), 7.35 (d, J = 8.5 Hz, 1H), 7.33 – 7.04 (m, 13H), 7.03 – 6.98 (m, 3H), 4.09 (qd, J = 7.2, 1.1 Hz, 2H), 2.68 – 2.54 (m, 2H), 2.49 – 2.41 (m, 1H), 2.38 (s, 3H), 2.35 – 2.28 (m, 1H), 1.19 (t, J = 7.2 Hz, 3H); **^{13}C NMR (126 MHz, $CDCl_3$)** δ 173.1, 145.3 (d, J = 34.1 Hz), 139.3, 137.7 (d, J = 7.5 Hz), 137.6 – 137.3 (m), 136.0 (d, J = 2.3 Hz), 134.7 (d, J = 10.4 Hz), 134.5 (d, J = 2.3 Hz), 133.7, 133.5 (d, J = 2.3 Hz), 133.1 (d, J = 2.3 Hz), 131.9, 131.6 (d, J = 6.4 Hz), 128.9, 128.3 – 128.1 (m), 127.6, 126.6 (d, J = 24.3 Hz), 125.3 (d, J = 77.4 Hz), 60.3, 35.1, 28.9 (d, J = 2.3 Hz), 21.4, 14.2; **^{31}P NMR (202 MHz, $CDCl_3$)** δ -16.60; **IR (film)**: 3911, 3682, 2360, 750 cm^{-1}; **HRMS m/z (ESI)**: calcd for $C_{34}H_{32}O_2P$ $[M+H]^+$: 503.2134, found 503.2123.

The enantiomeric excess of **5ba** was determined by chiral HPLC analysis on Ciralpak IB column. Conditions: hexane/isopropanol = 99: 1 flow rate = 1.0 L/min, 25 °C, uv-vis detection at λ = 220 nm, 90% ee, t_{R1} = 9.8 min (major), t_{R2} = 11.0 min (minor); $[\alpha]_D^{25}$ = -13.54 (c = 1.64, $CHCl_3$).

Ethyl (R)-3-(1-(2-(diphenylphosphanyl)-4-methylphenyl)naphthalen-2-yl)propanoate (5ca)

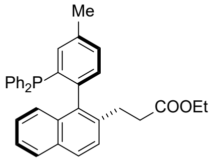
5ca

General Procedure **B**, a 25.0 mL dry Schlenk tube with a stirring bar was added with $[Ir(cod)Cl]_2$ (3.4 mg, 0.01 mmol, 0.05 equiv) and **L4** (8.0 mg, 0.022 mmol, 0.11 equiv) in 2.0 ml toluene under Ar. The formed mixture was stirred at room temperature for 1h. Then **1c** (80.4 mg, 0.2 mmol) and **4a** (100.0 mg, 1.0 mmol) was added under argon, the resulting mixture was stirred at 150 °C. After 48 h, the solvent was removed under vaccum. The crude product purified by column chromatography on silica gel (PE/EtOAc = 20/1) to afford the corresponding product **5ca** as a white solid (80.2 mg, 80%). **^{1}H NMR (500 MHz, CDCl$_3$)** δ 7.70 (d, J = 8.5 Hz, 1H), 7.66 (d, J = 7.2 Hz, 1H), 7.25 (d, J = 8.5 Hz, 1H), 7.22 – 7.19 (m, 1H), 7.19 – 7.15 (m, 4H), 7.11 – 7.07 (m, 2H), 7.05 (dd, J = 6.9, 2.6 Hz, 2H), 7.04 – 6.99 (m, 3H), 6.99 – 6.96 (m, 1H), 6.94 – 6.90 (m, 3H), 3.95 (q, J = 7.1 Hz, 2H), 2.58 – 2.50 (m, 1H), 2.50 – 2.43 (m, 1H), 2.39 – 2.31 (m, 1H), 2.28 – 2.20 (m, 4H), 1.08 (t, J = 7.2 Hz, 3H); **^{13}C NMR (126 MHz, CDCl$_3$)** δ 173.0, 142.2 (d, J = 33.5 Hz), 137.9 (d, J = 12.1 Hz), 137.5 (d, J = 7.5 Hz), 137.4, 137.2 (d, J = 3.5 Hz), 137.1 (d, J = 2.9 Hz), 136.3 (d, J = 2.3 Hz), 134.8 (d, J = 2.3 Hz), 133.8 (d, J = 1.6 Hz), 133.7 (d, J = 2.9 Hz), 133.3 (d, J = 2.9 Hz), 131.9, 130.8 (d, J = 6.4 Hz), 130.2, 128.24, 128.19, 128.1, 127.98 (d, J = 6.4 Hz), 127.93, 127.5, 126.5 (d, J = 14.5 Hz), 125.4, 124.8, 60.1, 35.1, 28.8 (d, J = 2.3 Hz), 21.4, 14.2; **^{31}P NMR (202 MHz, CDCl$_3$)** δ -15.22; **IR (film)**: 3911, 3699, 3575, 2360 cm^{-1}; **HRMS m/z (ESI)**: calcd for $C_{34}H_{32}O_2P$ [M+H]$^+$: 503.2134, found 503.2123.

The enantiomeric excess of **5ca** was determined by chiral HPLC analysis on Ciralpak IB column. Conditions: hexane/isopropanol = 99: 1 flow rate = 0.5 L/min, 25 °C, uv-vis detection at λ = 220 nm, 99% ee, t_{R1} = 6.5 min (minor), t_{R2} = 7.5 min (major) ; $[\alpha]_D^{25}$ = -17.27 (c = 3.0, $CHCl_3$).

Ethyl (R)-3-(1-(2-(diphenylphosphanyl)phenyl)-4-methylnaphthalen-2-yl)propanoate (5da)

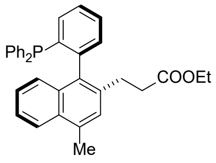
5da

General Procedure **B**, a 25.0 mL dry Schlenk tube with a stirring bar was added with $[Ir(cod)Cl]_2$ (3.4 mg, 0.01 mmol, 0.05 equiv)

and **L4** (8.0 mg, 0.022 mmol, 0.11 equiv) in 2.0 ml toluene under Ar. The formed mixture was stirred at room temperature for 1h. Then **1d** (80.4 mg, 0.2 mmol) and **4a** (200.0 mg, 2.0 mmol) was added under argon, the resulting mixture was stirred at 150 °C. After 48 h, the solvent was removed under vaccum. The crude product purified by column chromatography on silica gel (PE/EtOAc = 20/1) to afford the corresponding product **5da** as a white solid (66.3 mg, 66%). **^{1}H NMR (400 MHz, CDCl$_3$)** δ 7.9 (d, J = 8.4 Hz, 1H), 7.45 (t, J = 7.3 Hz, 1H), 7.40 (d, J = 7.5 Hz, 1H), 7.38 – 7.32 (m, 2H), 7.29 – 7.25 (m, 3H), 7.25 – 7.22 (m, 1H), 7.21 – 7.16 (m, 3H), 7.16 – 7.06 (m, 4H), 7.04 – 6.96 (m, 3H), 4.04 (q, J = 7.2 Hz, 2H), 2.71 (s, 3H), 2.58 – 2.48 (m, 2H), 2.48 – 2.39 (m, 1H), 2.37 – 2.27 (m, 1H), 1.18 (t, J = 7.2 Hz, 3H).; **^{13}C NMR (126 MHz, CDCl$_3$)** δ 173.0, 145.4 (d, J = 33.5 Hz), 138.4 (d, J = 11.6 Hz), 137.1 (d, J = 12.1 Hz), 137.0 (d, J = 12.6 Hz), 135.8 (d, J = 7.2 Hz), 135.6 (d, J = 1.7 Hz), 134.4 (d, J = 2.3 Hz), 134.2, 133.7 (d, J = 5.8 Hz), 133.6 (d, J = 6.4 Hz), 133.1 (d, J = 2.3 Hz), 131.1 (d, J = 6.0 Hz), 129.1, 128.3 (d, J = 2.9 Hz), 128.22 (d, J = 6.4 Hz), 1128.20, 128.0 (d, J = 6.9 Hz), 127.2 (d, J = 31.2 Hz), 125.2, 124.8, 123.8, 60.2, 35.2, 28.8, 19.6, 14.2; **^{31}P NMR (162 MHz, CDCl$_3$)** δ -15.72; **IR (film)**: 3810, 3662, 3576, 2360, 1276 cm^{-1}; **HRMS m/z (ESI)**: calcd for $C_{34}H_{32}O_2P$ [M+H]$^+$: 503.2134, found 503.2124.

The enantiomeric excess of **5da** was determined by chiral HPLC analysis on Ciralpak IC column. Conditions: hexane/isopropanol = 99: 1 flow rate = 0.5 L/min, 25 °C, uv-vis detection at λ = 220 nm, 93% ee, t_{R1} = 17.9 min (major), t_{R2} = 19.4 min (minor); $[\alpha]_D^{25}$ = -18.40 (c = 0.25, CHCl$_3$).

Ethyl (R)-3-(1-(2-(diphenylphosphanyl)-4,5-dimethylphenyl)naphthalen-2-yl)propanoate (5ea)

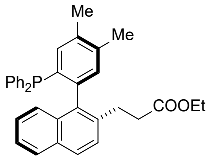

5ea

General Procedure **B**, a 25.0 mL dry Schlenk tube with a stirring bar was added with [Ir(cod)Cl]$_2$ (3.4 mg, 0.01 mmol, 0.05 equiv) and **L4** (8.0 mg, 0.022 mmol, 0.11 equiv) in 2.0 ml toluene under Ar. The formed mixture was stirred at room temperature for 1h. Then **1e** (83.2 mg, 0.2 mmol) and **4a** (100.0 mg, 1.0 mmol) was added under argon,

the resulting mixture was stirred at 150 °C. After 48 h, the solvent was removed under vaccum. The crude product purified by column chromatography on silica gel (PE/EtOAc = 20/1) to afford the corresponding product **5ea** as a white solid (57.8 mg, 56%). **^{1}H NMR (500 MHz, CDCl$_3$)** δ 7.80 (dd, *J* = 13.3, 8.3 Hz, 2H), 7.37 – 7.31 (m, 2H), 7.30 – 7.27 (m, 3H), 7.22 – 7.17 (m, 3H), 7.17 – 7.13 (m, 2H), 7.13 – 7.09 (m, 2H), 7.08 – 7.00 (m, 4H), 4.11 – 4.03 (m, 2H), 2.71 – 2.56 (m, 2H), 2.51 – 2.43 (m, 1H), 2.37 – 2.32 (m, 1H), 2.30 (s, 2H), 2.28 (s, 1H), 1.21 (td, *J* = 7.2, 2.0 Hz, 3H); **^{13}C NMR (126 MHz, CDCl$_3$)** δ 173.1, 142.8 (d, *J* = 34.1 Hz), 137.8 (d, *J* = 48.6 Hz), 137.7 (d, *J* = 5.2 Hz), 137.5 (d, *J* = 12.1 Hz), 136.14, 136.07 (d, *J* = 1.6 Hz), 135.4 (d, *J* = 2.9 Hz), 134.6 (d, *J* = 10.4 Hz), 133.6, 133.4, 133.2 (d, *J* = 2.3 Hz), 131.9 (d, *J* = 6.9 Hz), 131.8, 128.2 (d, *J* = 6.4 Hz), 128.1 (d, *J* = 4.0 Hz), 128.0 (d, *J* = 6.4 Hz), 127.7 (d, *J* = 37.6 Hz), 126.6 (d, *J* = 40.5 Hz), 125.4, 124.8, 60.2, 35.1, 28.8, 19.7 (d, *J* = 4.0 Hz), 14.2; **^{31}P NMR (202 MHz, CDCl$_3$)** δ -16.52; **IR (film)**: 3911, 3719, 3639, 2360, 1276 cm^{-1}; **HRMS m/z (ESI)**: calcd for $C_{35}H_{34}O_2P$ $[M+H]^+$: 517.2291, found 517.2280.

The enantiomeric excess of **5ea** was determined by chiral HPLC analysis on Ciralpak IA column. Conditions: hexane/isopropanol = 99: 1 flow rate = 0.5 L/min, 25 °C, uv-vis detection at λ = 220 nm, 88% ee, t_{R1} = 15.3 min (minor), t_{R2} = 22.8 min (major) ; $[\alpha]_D^{25}$ = -13.89 (*c* = 0.36, CHCl$_3$).

Ethyl (R)-3-(1-(2-(diphenylphosphanyl)phenyl)-4-phenylnaphthalen-2-yl)propanoate (5fa)

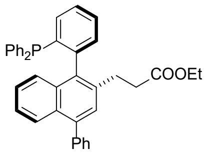

5fa

General Procedure **B**, a 25.0 mL dry Schlenk tube with a stirring bar was added with $[Ir(cod)Cl]_2$ (3.4 mg, 0.01 mmol, 0.05 equiv) and **L4** (8.0 mg, 0.022 mmol, 0.11 equiv) in 2.0 ml toluene under Ar. The formed mixture was stirred at room temperature for 1h. Then **1f** (92.8 mg, 0.2 mmol) and **4a** (100.0 mg, 1.0 mmol) was added under argon, the resulting mixture was stirred at 150 °C. After 48 h, the solvent was removed under vaccum. The crude product purified by column chromatography on silica gel

(PE/EtOAc = 20/1) to afford the corresponding product **5fa** as a white solid (62.0 mg, 55%). **^{1}H NMR (500 MHz, CDCl$_3$)** δ 7.85 (d, *J* = 8.4 Hz, 1H), 7.57 – 7.53 (m, 2H), 7.52 – 7.47 (m, 3H), 7.46 – 7.40 (m, 2H), 7.37 – 7.33 (m, 1H), 7.31 – 7.26 (m, 6H), 7.24 – 7.19 (m, 2H), 7.19 – 7.09 (m, 4H), 7.08 – 7.02 (m, 3H), 4.03 (q, *J* = 7.2 Hz, 2H), 2.68 – 2.60 (m, 1H), 2.60 – 2.52 (m, 1H), 2.50 – 2.42 (m, 1H), 2.39 – 2.31 (m, 1H), 1.17 (t, *J* = 7.2 Hz, 3H);**^{13}C NMR (126 MHz, CDCl$_3$)** δ 172.9, 145.0 (d, *J* = 32.9 Hz), 140.5 (d, *J* = 72.8 Hz), 138.4 (d, *J* = 12.1 Hz), 137.0, 136.91, 136.8 (d, *J* = 4.7 Hz), 136.7, 135.6 (d, *J* = 1.6 Hz), 134.3, 133.8 (d, *J* = 12.7 Hz), 133.7 (d, *J* = 13.3 Hz), 133.3 (d, *J* = 2.1 Hz), 131.0 (d, *J* = 5.8 Hz), 130.2, 130.0, 129.1, 128.4 (d, *J* = 4.0 Hz), 128.3 (d, *J* = 5.8 Hz), 128.2, 128.1 (d, *J* = 6.9 Hz), 127.9, 127.6, 127.2, 126.9, 125.7, 125.4, 125.0, 60.2, 35.1, 28.9 (d, *J* = 2.9 Hz), 14.2; **^{31}P NMR (202 MHz, CDCl$_3$)** δ -15.10; **IR (film)**: 3894, 3662, 3576, 2360, 1276 cm^{-1}; **HRMS m/z (ESI)**: calcd for $C_{39}H_{34}O_2P$ [M+H]$^+$: 565.2291, found 565.2277.

The enantiomeric excess of **5fa** was determined by chiral HPLC analysis on Ciralpak IB column. Conditions: hexane/isopropanol = 99: 1 flow rate = 1.0 L/min, 25 °C, uv-vis detection at λ = 220 nm, 93% ee, t_{R1} = 13.2 min (minor), t_{R2} = 15.6 min (major) ; $[\alpha]_D^{25}$ = -2.67 (*c* = 0.3, CHCl$_3$).

Ethyl (R)-3-(1-(2-(diphenylphosphanyl)-5-methoxyphenyl)naphthalen-2-yl)propanoate (5ga)

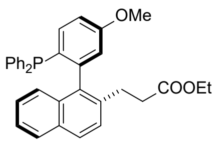

5ga

General Procedure **B**, a 25.0 mL dry Schlenk tube with a stirring bar was added with [Ir(cod)Cl]$_2$ (3.4 mg, 0.01 mmol, 0.05 equiv) and **L4** (8.0 mg, 0.022 mmol, 0.11 equiv) in 2.0 ml toluene under Ar. The formed mixture was stirred at room temperature for 1h. Then **1g** (83.6 mg, 0.2 mmol) and **4a** (100.0 mg, 1.0 mmol) was added under argon, the resulting mixture was stirred at 150 °C. After 48 h, the solvent was removed under vaccum. The crude product purified by column chromatography on silica gel (PE/EtOAc = 20/1) to afford the corresponding product **5ga** as a white solid (70.5 mg, 68%). **^{1}H NMR (500 MHz, CDCl$_3$)** δ 7.86 (d, *J* = 8.4 Hz, 1H), 7.82 (d, *J* = 8.1 Hz,

1H), 7.40 (d, J = 8.5 Hz, 1H), 7.38 – 7.34 (m, 1H), 7.33 – 7.28 (m, 4H), 7.25 – 7.19 (m, 3H), 7.19 – 7.14 (m, 3H), 7.12 – 7.09 (m, 1H), 7.08 – 7.04 (m, 2H), 7.00 (dd, J = 8.5, 2.7 Hz, 1H), 6.85 (dd, J = 3.7, 2.7 Hz, 1H), 4.09 (qd, J = 7.1, 1.1 Hz, 2H), 3.83 (s, 3H), 2.76 – 2.60 (m, 2H), 2.56 – 2.47 (m, 1H), 2.43 – 2.34 (m, 1H), 1.23 (t, J = 7.2 Hz, 3H); **^{13}C NMR (126 MHz, $CDCl_3$)** δ 172.9, 160.3, 146.9 (d, J = 36.4 Hz), 137.8 (d, J = 12.1 Hz), 137.6 (d, J = 12.7 Hz), 137.4 (d, J = 6.9 Hz), 136.0 (d, J = 2.9 Hz), 135.8 (d, J = 2.3 Hz), 133.5, 133.3 (d, J = 2.9 Hz), 132.9 (d, J = 2.3 Hz), 131.7, 128.9 (d, J = 9.2 Hz), 128.2 (d, J = 6.4 Hz), 128.1 (d, J = 2.3 Hz), 128.03 (d, J = 3.5 Hz), 127.96, 127.6, 126.5 (d, J = 21.4 Hz), 126.0, 125.0, 115.8 (d, J = 6.9 Hz), 114.2, 60.2, 55.2, 35.1, 28.7, 14.1; **^{31}P NMR (202 MHz, $CDCl_3$)** δ -17.63; **IR (film)**: 3845, 3576, 2360, 1276 cm^{-1}; **HRMS m/z (ESI)**: calcd for $C_{34}H_{32}O_3P$ $[M+H]^+$: 519.2084, found 519.2073.

The enantiomeric excess of **3ga** was determined by chiral HPLC analysis on Ciralpak IA column. Conditions: hexane/isopropanol = 99: 1 flow rate = 0.7 L/min, 25 °C, uv-vis detection at λ = 220 nm, 82% ee, t_{R1} = 14.1 min (minor), t_{R2} = 25.1 min (major) ; $[\alpha]_D^{25}$ = -20.00 (c = 3.1, $CHCl_3$).

Ethyl (R)-3-(1-(2-(diphenylphosphanyl)phenyl)-4-methoxynaphthalen-2-yl)propanoate (5ha)

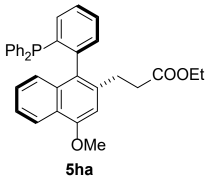

5ha

General Procedure **B**, a 25.0 mL dry Schlenk tube with a stirring bar was added with $[Ir(cod)Cl]_2$ (3.4 mg, 0.01 mmol, 0.05 equiv) and **L4** (8.0 mg, 0.022 mmol, 0.11 equiv) in 2.0 ml toluene under Ar. The formed mixture was stirred at room temperature for 1h. Then **1h** (83.6 mg, 0.2 mmol) and **4a** (100.0 mg, 1.0 mmol) was added under argon, the resulting mixture was stirred at 150 °C. After 48 h, the solvent was removed under vaccum. The crude product purified by column chromatography on silica gel (PE/EtOAc = 20/1) to afford the corresponding product **5ha** as a white solid (21.8 mg, 21%). **^{1}H NMR (500 MHz, $CDCl_3$)** δ 8.19 (d, J = 8.4 Hz, 1H), 7.45 (td, J = 7.4, 1.5 Hz, 1H), 7.39 (td, J = 7.6, 1.5 Hz, 1H), 7.34 – 7.30 (m, 2H), 7.30 – 7.26 (m, 4H), 7.23

(ddd, J = 7.5, 4.4, 1.7 Hz, 1H), 7.21 – 7.14 (m, 3H), 7.14 – 7.09 (m, 2H), 7.09 – 7.05 (m, 1H), 7.00 (tt, J = 7.3, 1.4 Hz, 2H), 6.89 (d, J = 8.4 Hz, 1H), 6.69 (s, 1H), 4.05 (q, J = 7.2 Hz, 2H), 4.02 (s, 3H), 2.65 – 2.52 (m, 2H), 2.48 – 2.41 (m, 1H), 2.40 – 2.32 (m, 1H), 1.19 (t, J = 7.1 Hz, 3H); **^{13}C NMR (126 MHz, CDCl$_3$)** δ 173.0, 155.2, 145.3 (d, J = 33.5 Hz), 138.9 (d, J = 11.0 Hz), 137.2 (d, J = 12.7 Hz), 137.0 (d, J = 12.7 Hz), 136.3 (d, J = 1.7 Hz), 134.3 (d, J = 2.3 Hz), 134.0 (d, J = 2.3 Hz), 133.8 (d, J = 12.7 Hz), 133.6 (d, J = 11.6 Hz), 131.5 (d, J = 5.8 Hz), 129.9 (d, J = 7.5 Hz), 129.1, 128.33, 128.27, 128.1 (d, J = 6.9 Hz), 127.7, 126.2 (d, J = 21.4 Hz), 124.3, 124.1, 121.5, 104.4, 60.8, 55.5, 35.3, 29.4 (d, J = 2.9 Hz), 14.2; **^{31}P NMR (202 MHz, CDCl$_3$)** δ -15.48; **IR (film)**: 3846, 3661, 3576, 2360, 1276 cm^{-1}; **HRMS m/z (ESI)**: calcd for $C_{34}H_{32}O_3P$ [M+H]$^+$: 519.2084, found 519.2073.

The enantiomeric excess of **5ha** was determined by chiral HPLC analysis on Ciralpak IA column. Conditions: hexane/isopropanol = 99: 1 flow rate = 1.0 L/min, 25 °C, uv-vis detection at λ = 220 nm, 90% ee, t_{R1} = 6.5 min (major), t_{R2} = 7.4 min (minor); $[\alpha]_D^{25}$ = -17.90 (c = 0.38, CHCl$_3$).

Ethyl (R)-3-(1-(6-(diphenylphosphanyl)benzo[d][1,3]dioxol-5-yl)naphthalen-2-yl)propanoate (5ia)

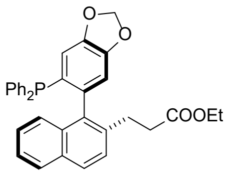

5ia

General Procedure **B**, a 25.0 mL dry Schlenk tube with a stirring bar was added with [Ir(cod)Cl]$_2$ (3.4 mg, 0.01 mmol, 0.05 equiv) and **L4** (8.0 mg, 0.022 mmol, 0.11 equiv) in 2.0 ml toluene under Ar. The formed mixture was stirred at room temperature for 1h. Then **1i** (86.2 mg, 0.2 mmol) and **4a** (100.0 mg, 1.0 mmol) was added under argon, the resulting mixture was stirred at 150 °C. After 48 h, the solvent was removed under vaccum. The crude product purified by column chromatography on silica gel (PE/EtOAc = 20/1) to afford the corresponding product **5ia** as a white solid (59.6 mg, 56%). **^{1}H NMR (500 MHz, CDCl$_3$)** δ^{1}H NMR (500 MHz, Chloroform-d) δ 7.79 (dd, J = 15.8, 8.2 Hz, 2H), 7.37 – 7.30 (m, 2H), 7.30 – 7.25 (m, 3H), 7.22 – 7.16 (m, 3H), 7.16 – 7.08 (m, 4H), 7.03 – 6.97 (m, 2H), 6.80 (d, J = 2.4 Hz, 1H), 6.72 (d, J = 3.5 Hz,

1H), 6.02 (dd, J = 10.1, 1.5 Hz, 2H), 4.06 (q, J = 7.1 Hz, 2H), 2.75 – 2.66 (m, 1H), 2.64 – 2.55 (m, 1H), 2.54 – 2.42 (m, 1H), 2.39 – 2.25 (m, 1H), 1.20 (t, J = 7.2 Hz, 3H); **^{13}C NMR (126 MHz, CDCl$_3$)** δ 172.9, 148.8, 147.5, 139.9 (d, J = 37.0 Hz), 137.7 (d, J = 12.7 Hz), 137.4 (d, J = 12.7 Hz), 137.2 (d, J = 8.1 Hz), 136.4, 133.5 (d, J = 19.7 Hz), 133.3 (d, J = 2.3 Hz), 131.9, 130.7 (d, J = 12.1 Hz), 128.4 (d, J = 6.4 Hz), 128.3 (d, J = 2.9 Hz), 128.2 (d, J = 7.5 Hz), 127.7, 126.5 (d, J = 15.6 Hz), 125.7, 125.1, 113.7, 111.1 (d, J = 6.9 Hz), 101.4, 60.3, 35.1, 28.8 (d, J = 2.9 Hz), 14.3; **^{31}P NMR (202 MHz, CDCl$_3$)** δ -15.49; **IR (film)**: 3911, 3576, 2360, 1276 cm^{-1}; **HRMS m/z (ESI)**: calcd for $C_{34}H_{30}O_4P$ $[M+H]^+$: 533.1876, found 533.1865.

The enantiomeric excess of **5ia** was determined by chiral HPLC analysis on Ciralpak IB column. Conditions: hexane/isopropanol = 99: 1 flow rate = 0.5 L/min, 25 °C, uv-vis detection at λ = 220 nm, 88% ee, t_{R1} = 20.4 min (major), t_{R2} = 23.6 min (minor) ; $[\alpha]_D^{25}$ = -1.55 (c = 0.33, CHCl$_3$).

Ethyl (R)-3-(1-(2-(diphenylphosphanyl)-4-fluorophenyl)naphthalen-2-yl)propanoate (5ja)

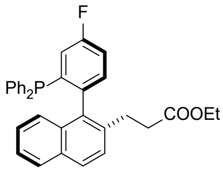

5ja

General Procedure **B**, a 25.0 mL dry Schlenk tube with a stirring bar was added with [Ir(cod)Cl]$_2$ (3.4 mg, 0.01 mmol, 0.05 equiv) and **L4** (8.0 mg, 0.022 mmol, 0.11 equiv) in 2.0 ml toluene under Ar. The formed mixture was stirred at room temperature for 1h. Then **1j** (81.2 mg, 0.2 mmol) and **4a** (100.0 mg, 1.0 mmol) was added under argon, the resulting mixture was stirred at 150 °C. After 48 h, the solvent was removed under vaccum. The crude product purified by column chromatography on silica gel (PE/EtOAc = 20/1) to afford the corresponding product **5ja** as a white solid (77.9 mg, 77%). **^{1}H NMR (500 MHz, CDCl$_3$)** δ 7.81 (d, J = 8.4 Hz, 1H), 7.75 (dd, J = 8.1, 1.4 Hz, 1H), 7.34 (d, J = 8.5 Hz, 1H), 7.32 – 7.26 (m, 4H), 7.21 – 7.15 (m, 5H), 7.13 – 7.06 (m, 3H), 7.03 – 7.00 (m, 1H), 7.00 – 6.96 (m, 2H), 6.93 (d, J = 8.1 Hz, 1H), 4.05 (q, J = 7.2 Hz, 2H), 2.63 – 2.55 (m, 1H), 2.55 – 2.48 (m, 1H), 2.47 – 2.40 (m, 1H), 2.38 – 2.30 (m, 1H), 1.19 (t, J = 7.1 Hz, 3H);**^{13}C NMR (126 MHz, CDCl$_3$)** δ 172.9,

163.3, 161.3, 141.7 (dd, *J* = 15.9, 4.3 Hz), 140.6 (dd, *J* = 31.8, 3.5 Hz), 136.5 (d, *J* = 1.7 Hz), 136.2 (d, *J* = 6.9 Hz), 136.1 (d, *J* = 12.1 Hz), 135.9 (d, *J* = 12.1 Hz), 133.8 (d, *J* = 20.8 Hz), 133.1 (d, *J* = 2.3 Hz), 132.5 (dd, *J* = 7.5, 5.2 Hz), 131.9, 128.7 (d, *J* = 11.0 Hz), 128.6 (d, *J* = 6.4 Hz), 128.4, 128.3 (d, *J* = 6.9 Hz), 127.1 (d, *J* = 149.1 Hz), 126.3, 125.4 (d, *J* = 89.6 Hz), 120.5 (d, *J* = 21.4 Hz), 116.3 (d, *J* = 21.4 Hz), 60.3, 35.1 (d, *J* = 1.7 Hz), 28.9 (d, *J* = 2.9 Hz), 14.3; **^{31}P NMR (202 MHz, $CDCl_3$)** δ -14.68; **^{19}F NMR (471 MHz, $CDCl_3$)** δ -113.92; **IR (film)**: 3911, 3576, 2360, 1276 cm^{-1}; **HRMS m/z (ESI)**: calcd for $C_{33}H_{29}FO_2P$ $[M+H]^+$: 507.1884, found 507.1883.
The enantiomeric excess of **5ja** was determined by chiral HPLC analysis on Ciralpak IB column. Conditions: hexane/isopropanol = 99: 1 flow rate = 1.0 L/min, 25 °C, uv-vis detection at λ = 220 nm, 92% ee, t_{R1} = 13.4 min (major), t_{R2} = 14.9 min (minor) ; $[\alpha]_D^{25}$ = -9.07 (*c* = 1.72, $CHCl_3$).

Ethyl (R)-3-(1-(2-(diphenylphosphanyl)-4,5-difluorophenyl)naphthalen-2-yl)propanoate (5ka)

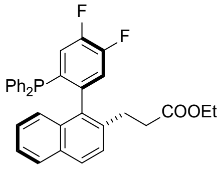

5ka

General Procedure **B**, a 25.0 mL dry Schlenk tube with a stirring bar was added with $[Ir(cod)Cl]_2$ (3.4 mg, 0.01 mmol, 0.05 equiv) and **L4** (8.0 mg, 0.022 mmol, 0.11 equiv) in 2.0 ml toluene under Ar. The formed mixture was stirred at room temperature for 1h. Then **1k** (84.8 mg, 0.2 mmol) and **4a** (100.0 mg, 1.0 mmol) was added under argon, the resulting mixture was stirred at 150 °C. After 48 h, the solvent was removed under vaccum. The crude product purified by column chromatography on silica gel (PE/EtOAc = 20/1) to afford the corresponding product **5ka** as a white solid (24.1 mg, 23%). **^{1}H NMR (500 MHz, $CDCl_3$)** δ 7.83 (d, *J* = 8.5 Hz, 1H), 7.78 (d, *J* = 8.1 Hz, 1H), 7.36 – 7.29 (m, 5H), 7.20 (t, *J* = 7.3 Hz, 1H), 7.17 – 7.11 (m, 5H), 7.11 – 7.05 (m, 2H), 7.00 – 6.90 (m, 3H), 4.07 (q, *J* = 7.2 Hz, 2H), 2.65 – 2.57 (m, 1H), 2.57 – 2.49 (m, 1H), 2.49 – 2.41 (m, 1H), 2.39 – 2.31 (m, 1H), 1.21 (t, *J* = 7.2 Hz, 3H); **^{13}C NMR (126 MHz, $CDCl_3$)** δ 172.7, 148.7 (d, *J* = 32.4 Hz), 140.7 (d, *J* = 16.8 Hz), 136.0 (d, *J* = 1.8 Hz), 135.9 (d, *J* = 6.9 Hz), 135.5 (dd, *J* = 23.4, 11.8 Hz), 133.8 (d, *J* = 4.0 Hz),

133.6 (d, J = 3.5 Hz), 132.4 (d, J = 2.3 Hz), 131.8, 131.3 (d, J = 5.2 Hz), 130.4 (q, J = 4.3 Hz), 128.9, 128.7 (d, J = 12.1 Hz), 128.6 (d, J = 6.9 Hz), 128.3 (d, J = 7.5 Hz), 127.7, 126.3, 125.9 (d, J = 12.1 Hz), 125.7 (q, J = 3.4 Hz), 125.1, 60.3, 35.0, 28.7 (d, J = 2.9 Hz), 14.1; **^{31}P NMR (202 MHz, $CDCl_3$)** δ -16.02; ^{19}F NMR (471 MHz, **$CDCl_3$**) δ -136.36 (d, J = 21.7 Hz, 1F), -138.21 (d, J = 21.7 Hz, 1F); **IR (film)**: 3911, 3576, 2360, 1267 cm^{-1}; **HRMS m/z (ESI)**: calcd for $C_{33}H_{28}F_2O_2P$ [M+H]$^+$: 525.1789, found 525.1780.

The enantiomeric excess of **5ka** was determined by chiral HPLC analysis on Ciralpak IB column. Conditions: hexane/isopropanol = 99: 1 flow rate = 1.0 L/min, 25 °C, uv-vis detection at λ = 220 nm, 93% ee, t_{R1} = 12.4 min (major), t_{R2} = 14.5 min (minor) ; $[\alpha]_D^{25}$ = -41.14 (c = 0.175, $CHCl_3$).

Ethyl (R)-3-(1-(5-chloro-2-(diphenylphosphanyl)phenyl)naphthalen-2-yl)propanoate (5la)

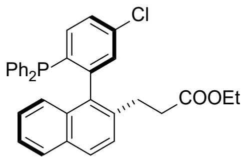

5la

General Procedure **B**, a 25.0 mL dry Schlenk tube with a stirring bar was added with $[Ir(cod)Cl]_2$ (3.4 mg, 0.01 mmol, 0.05 equiv) and **L4** (8.0 mg, 0.022 mmol, 0.11 equiv) in 2.0 ml toluene under Ar. The formed mixture was stirred at room temperature for 1h. Then **1l** (84.4 mg, 0.2 mmol) and **4a** (100.0 mg, 1.0 mmol) was added under argon, the resulting mixture was stirred at 150 °C. After 48 h, the solvent was removed under vaccum. The crude product purified by column chromatography on silica gel (PE/EtOAc = 20/1) to afford the corresponding product **5la** as a white solid (53.2 mg, 51%). **^{1}H NMR (500 MHz, $CDCl_3$)** δ 7.83 (d, J = 8.6 Hz, 1H), 7.78 (d, J = 7.3 Hz, 1H), 7.40 – 7.32 (m, 3H), 7.31 – 7.27 (m, 3H), 7.27 – 7.24 (m, 2H), 7.20 – 7.07 (m, 6H), 7.00 – 6.92 (m, 3H), 4.07 (q, J = 7.0, 0.9 Hz, 2H), 2.67 – 2.51 (m, 2H), 2.50 – 2.42 (m, 1H), 2.40 – 2.31 (m, 1H), 1.20 (t, J = 7.2 Hz, 3H); **^{13}C NMR (126 MHz, $CDCl_3$)** δ 172.8, 146.8 (d, J = 34.5 Hz), 137.4 (d, J = 13.2 Hz), 136.4 (d, J = 5.5 Hz), 136.3 (d, J = 5.4 Hz), 136.1 (d, J = 2.3 Hz), 135.9 (d, J = 6.8 Hz), 135.6 (d, J = 1.8 Hz), 135.4, 133.7 (d, J = 3.6 Hz), 133.5 (d, J = 2.7 Hz), 132.7 (d, J = 2.3 Hz),

131.8, 130.7 (d, J = 5.9 Hz), 128.6, 128.5 (d, J = 2.7 Hz), 128.3 (d, J = 19.5 Hz), 128.2 (d, J = 1.8 Hz), 127.7, 126.3 (d, J = 12.7 Hz), 125.8, 125.1, 60.3, 35.0 (d, J = 1.8 Hz), 28.8 (d, J = 2.3 Hz), 14.2; **^{31}P NMR (202 MHz, CDCl$_3$)** δ -16.63; **IR (film)**: 3910, 3576, 2360, 1267 cm^{-1}; **HRMS m/z (ESI)**: calcd for $C_{33}H_{29}ClO_2P$ [M+H]$^+$: 523.1588, found 523.1578.

The enantiomeric excess of **5la** was determined by chiral HPLC analysis on Ciralpak IC column. Conditions: hexane/isopropanol = 99: 1 flow rate = 0.5 L/min, 25 °C, uv-vis detection at λ = 220 nm, 90% ee, t_{R1} = 15.0 min (major), t_{R2} = 17.7 min (minor) ; $[\alpha]_D^{25}$ = -10.23 (c = 0.215, CHCl$_3$).

Ethyl (R)-3-(4-chloro-1-(2-(diphenylphosphanyl)phenyl)naphthalen-2-yl)propanoate (5ma)

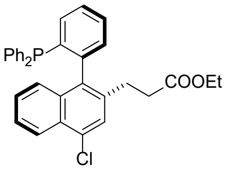

5ma

General Procedure **B**, a 25.0 mL dry Schlenk tube with a stirring bar was added with [Ir(cod)Cl]$_2$ (3.4 mg, 0.01 mmol, 0.05 equiv) and **L4** (8.0 mg, 0.022 mmol, 0.11 equiv) in 2.0 ml toluene under Ar. The formed mixture was stirred at room temperature for 1h. Then **1m** (84.4 mg, 0.2 mmol) and **4a** (100.0 mg, 1.0 mmol) was added under argon, the resulting mixture was stirred at 150 °C. After 48 h, the solvent was removed under vaccum. The crude product purified by column chromatography on silica gel (PE/EtOAc = 20/1) to afford the corresponding product **5ma** as a white solid (78.3 mg, 75%). **^{1}H NMR (500 MHz, CDCl$_3$)** δ 8.22 (d, J = 7.5 Hz, 1H), 7.50 – 7.46 (m, 2H), 7.43 (td, J = 7.6, 1.5 Hz, 2H), 7.34 (ddd, J = 7.8, 3.6, 1.6 Hz, 1H), 7.32 – 7.28 (m, 3H), 7.24 – 7.21 (m, 1H), 7.21 – 7.17 (m, 3H), 7.16 – 7.10 (m, 3H), 7.03 – 6.97 (m, 3H), 4.07 (q, J = 7.2 Hz, 2H), 2.64 – 2.56 (m, 1H), 2.56 – 2.49 (m, 1H), 2.49 – 2.40 (m, 1H), 2.39 – 2.31 (m, 1H), 1.21 (t, J = 7.1 Hz, 3H); **^{13}C NMR (126 MHz, CDCl$_3$)** δ 172.6, 144.2 (d, J = 32.9 Hz), 138.5 (d, J = 12.1 Hz), 136.9 (d, J = 6.9 Hz), 136.61 (d, J = 9.2 Hz), 136.55 (d, J = 1.8 Hz), 136.5, 134.3 (d, J = 1.7 Hz), 134.1 (d, J = 2.9 Hz), 133.8 (d, J = 4.6 Hz), 133.7 (d, J = 3.5 Hz), 131.8, 130.9 (d, J = 5.8 Hz), 129.21, 129.18, 128.5 (d, J = 6.4 Hz), 128.4 (d, J = 4.0 Hz), 128.1 (d, J = 2.9 Hz), 126.9 (d, J

= 31.2 Hz), 126.2 (d, *J* = 35.8 Hz), 124.1, 60.4, 34.9 (d, *J* = 2.3 Hz), 28.7(d, *J* = 2.9 Hz), 14.2; **^{31}P NMR (202 MHz, CDCl$_3$)** δ -15.50; **IR (film)**: 3911, 3699, 3576, 2360, 1584 cm^{-1}; **HRMS m/z (ESI)**: calcd for $C_{33}H_{29}ClO_2P$ [M+H]$^+$: 523.1588, found 523.1574.

The enantiomeric excess of **5ma** was determined by chiral HPLC analysis on Ciralpak IB column. Conditions: hexane/isopropanol = 99.5: 0.5 flow rate = 0.5 L/min, 25 °C, uv-vis detection at λ = 220 nm, 93% ee, t_{R1} = 13.5 min (major), t_{R2} = 14.3 min (minor); $[\alpha]_D^{25}$ = -33.81 (*c* = 1.68, CHCl$_3$).

Ethyl (R)-3-(1-(2-(diphenylphosphanyl)-5-(trifluoromethyl)phenyl)naphthalen-2-yl)propanoate (5na)

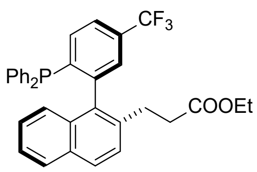

5na

General Procedure **B**, a 25.0 mL dry Schlenk tube with a stirring bar was added with [Ir(cod)Cl]$_2$ (3.4 mg, 0.01 mmol, 0.05 equiv) and **L4** (8.0 mg, 0.022 mmol, 0.11 equiv) in 2.0 ml toluene under Ar. The formed mixture was stirred at room temperature for 1h. Then **1n** (91.2 mg, 0.2 mmol) and **4a** (100.0 mg, 1.0 mmol) was added under argon, the resulting mixture was stirred at 150 °C. After 48 h, the solvent was removed under vaccum. The crude product purified by column chromatography on silica gel (PE/EtOAc = 20/1) to afford the corresponding product **5na** as a white solid (47.8 mg, 43%). **^{1}H NMR (500 MHz, CDCl$_3$)** δ 7.84 (d, *J* = 8.5 Hz, 1H), 7.78 (dd, *J* = 8.2, 1.5 Hz, 1H), 7.64 (dd, *J* = 8.2, 2.2 Hz, 1H), 7.50 (dd, *J* = 3.8, 2.1 Hz, 1H), 7.43 (dd, *J* = 8.2, 3.0 Hz, 1H), 7.35 (d, 1H), 7.33 – 7.28 (m, 4H), 7.20 – 7.16 (m, 2H), 7.16 – 7.07 (m, 4H), 7.00 – 6.94 (m, 2H), 6.85 (d, *J* = 8.4 Hz, 1H), 4.05 (qd, *J* = 7.1, 1.3 Hz, 2H), 2.59 – 2.50 (m, 2H), 2.50 – 2.42 (m, 1H), 2.42 – 2.33 (m, 1H), 1.18 (t, *J* = 7.1 Hz, 3H) ; **^{13}C NMR (126 MHz, CDCl$_3$)** δ 172.7, 145.6 (d, *J* = 32.9 Hz), 144.1 (d, *J* = 16.8 Hz), 136.3 (d, *J* = 2.3 Hz), 135.8, 135.7, 135.6, 135.5, 135.4, 134.4, 133.9 (d, *J* = 4.6 Hz), 133.8 (d, *J* = 3.5 Hz), 132.6 (d, *J* = 1.7 Hz), 131.9, 131.1 (q, *J* = 32.4 Hz), 128.9, 128.8, 128.6 (d, *J* = 6.4 Hz), 128.3 (d, *J* = 7.5 Hz), 127.7, 127.4 (dq, *J* = 7.6, 3.6 Hz), 126.4, 126.0, 125.9, 125.1, 124.5 (q, *J* = 3.4 Hz), 60.3, 35.0 (d, *J* = 1.7

Hz), 28.8 (d, J = 2.3 Hz), 14.1; **^{31}P NMR (202 MHz, $CDCl_3$)** δ -15.19; **^{19}F NMR (471 MHz, $CDCl_3$)** δ -62.59; **IR (film)**: 3699, 3576, 2360, 1276 cm^{-1}; **HRMS m/z (ESI)**: calcd for $C_{34}H_{29}F_3O_2P$ $[M+H]^+$: 557.1852, found 557.1841.

The enantiomeric excess of **5na** was determined by chiral HPLC analysis on Ciralpak IC column. Conditions: hexane/isopropanol = 99: 1 flow rate = 0.5 L/min, 25 °C, uv-vis detection at λ = 220 nm, 90% ee, t_{R1} = 11.7 min (major), t_{R2} = 12.7 min (minor) ; $[\alpha]_D^{25}$ = -53.44 (c = 0.64, $CHCl_3$).

Ethyl (R)-3-(1-(2-(diphenylphosphanyl)-4-(trifluoromethyl)phenyl)naphthalen-2-yl)propanoate (5oa)

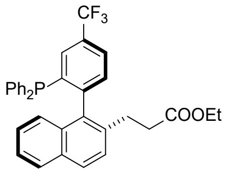

5oa

General Procedure **B**, a 25.0 mL dry Schlenk tube with a stirring bar was added with $[Ir(cod)Cl]_2$ (3.4 mg, 0.01 mmol, 0.05 equiv) and **L4** (8.0 mg, 0.022 mmol, 0.11 equiv) in 2.0 ml toluene under Ar. The formed mixture was stirred at room temperature for 1h. Then **1o** (91.2 mg, 0.2 mmol) and **4a** (100.0 mg, 1.0 mmol) was added under argon, the resulting mixture was stirred at 150 °C. After 48 h, the solvent was removed under vaccum. The crude product purified by column chromatography on silica gel (PE/EtOAc = 20/1) to afford the corresponding product **5oa** as a white solid (53.4 mg, 48%). **^{1}H NMR (500 MHz, $CDCl_3$)** δ 7.84 (d, J = 8.6 Hz, 1H), 7.77 (d, J = 7.9 Hz, 1H), 7.71 (dd, J = 7.9, 2.2 Hz, 1H), 7.57 (s, 1H), 7.39 – 7.34 (m, 2H), 7.34 – 7.27 (m, 4H), 7.20 – 7.14 (m, 3H), 7.13 – 7.06 (m, 3H), 6.96 (t, J = 7.4 Hz, 2H), 6.85 (d, J = 8.5 Hz, 1H), 4.05 (q, J = 7.2 Hz, 2H), 2.59 – 2.49 (m, 2H), 2.48 – 2.40 (m, 1H), 2.40 – 2.31 (m, 1H), 1.18 (t, J = 7.2 Hz, 3H); **^{13}C NMR (126 MHz, $CDCl_3$)** δ 172.7, 148.8 (d, J = 32.4 Hz), 140.8 (d, J = 16.8 Hz), 136.0 (d, J = 2.3 Hz), 135.9 (d, J = 6.9 Hz), 135.6 (d, J = 12.1 Hz), 135.4 (d, J = 12.1 Hz), 133.9 (d, J = 3.5 Hz), 133.7 (d, J = 2.3 Hz), 132.5 (d, J = 2.3 Hz), 131.9, 131.4 (d, J = 5.2 Hz), 130.4 (q, J = 4.7, 4.2 Hz), 130.11 (d, J = 32.2 Hz), 128.9 (d, J = 11.6 Hz), 128.7, 128.6 (d, J = 6.9 Hz), 128.3 (d, J = 7.5 Hz), 127.7, 126.4, 126.0 (d, J = 12.1 Hz), 125.8 (q, J = 3.5 Hz), 125.2, 60.4, 35.0, 28.8 (d, J = 2.9 Hz), 14.2; **^{31}P NMR (202 MHz, $CDCl_3$)** δ -14.84; **^{19}F NMR**

(471 MHz, $CDCl_3$) δ -108.22; **IR (film)**: 3911, 3699, 3576, 2360, 1276 cm^{-1}; **HRMS m/z (ESI)**: calcd for $C_{34}H_{29}F_3O_2P$ $[M+H]^+$: 557.1852, found 557.1840.

The enantiomeric excess of **5oa** was determined by chiral HPLC analysis on Ciralpak IA column. Conditions: hexane/isopropanol = 99: 1 flow rate = 0.5 L/min, 25 °C, uv-vis detection at λ = 220 nm, 99% ee, t_{R1} = 14.4 min (major), t_{R2} = 17.4 min (minor); $[\alpha]_D^{25}$ = -8.591 (c = 1.49, $CHCl_3$).

Ethyl (R)-3-(5-(2-(diphenylphosphanyl)phenyl)-1,2-dihydroacenaphthylen-4-yl)propanoate (5pa)

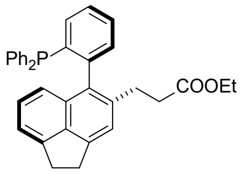

5pa

General Procedure **B**, a 25.0 mL dry Schlenk tube with a stirring bar was added with $[Ir(cod)Cl]_2$ (3.4 mg, 0.01 mmol, 0.05 equiv) and **L4** (8.0 mg, 0.022 mmol, 0.11 equiv) in 2.0 ml toluene under Ar. The formed mixture was stirred at room temperature for 1h. Then **1p** (82.8 mg, 0.2 mmol) and **4a** (100.0 mg, 1.0 mmol) was added under argon, the resulting mixture was stirred at 150 °C. After 48 h, the solvent was removed under vaccum. The crude product purified by column chromatography on silica gel (PE/EtOAc = 20/1) to afford the corresponding product **5pa** as a white solid (62.7 mg, 61%). **^{1}H NMR (500 MHz, $CDCl_3$)** δ 7.44 (td, J = 7.5, 1.7 Hz, 1H), 7.37 (td, J = 7.6, 1.5 Hz, 1H), 7.34 – 7.30 (m, 1H), 7.27 (dd, J = 4.4, 2.3 Hz, 3H), 7.25 – 7.20 (m, 2H), 7.20 – 7.16 (m, 2H), 7.16 – 7.11 (m, 5H), 7.04 (td, J = 7.8, 1.7 Hz, 2H), 6.73 (dd, J = 6.6, 2.4 Hz, 1H), 4.03 (q, J = 7.1 Hz, 2H), 3.46 – 3.29 (m, 4H), 2.66 – 2.58 (m, 1H), 2.58 – 2.50 (m, 1H), 2.45 – 2.37 (m, 1H), 2.34 – 2.25 (m, 1H), 1.18 (t, J = 7.2 Hz, 3H); **^{13}C NMR (126 MHz, $CDCl_3$)** δ 173.0, 145.7 (d, J = 66.5 Hz), 145.1 (d, J = 33.5 Hz), 138.4 (d, J = 11.0 Hz), 138.0 (d, J = 2.9 Hz), 137.8, 137.6 (d, J = 12.7 Hz), 137.2, 134.5 (d, J = 2.9 Hz), 133.8 (d, J = 19.7 Hz), 133.6 (d, J = 20.2 Hz), 133.2 (d, J = 7.5 Hz), 131.4 (d, J = 2.3 Hz), 131.1 (d, J = 5.8 Hz), 129.2, 128.4, 128.3 (d, J = 17.9 Hz), 128.1 (d, J = 6.9 Hz), 127.7 (d, J = 2.3 Hz), 121.5, 120.1, 118.7, 60.2, 35.6, 30.6, 30.2, 29.3(d, J = 2.3 Hz), 14.3; **^{31}P NMR (202 MHz, $CDCl_3$)** δ -15.59; **IR (film)**: 3846, 3662, 3576, 2360, 1276 cm^{-1}; **HRMS m/z (ESI)**: calcd for $C_{35}H_{32}O_2P$ $[M+H]^+$:

515.2134, found 515.2123.

The enantiomeric excess of **5pa** was determined by chiral HPLC analysis on Ciralpak IB column. Conditions: hexane/isopropanol = 99: 1 flow rate = 1.0 L/min, 25 ºC, uv-vis detection at λ = 220 nm, 93% ee, t_{R1} = 13.8 min (major), t_{R2} = 15.1 min (minor); $[\alpha]_D^{25}$ = -12.01 (c = 1.92, $CHCl_3$).

Ethyl (R)-3-(10-(2-(diphenylphosphanyl)phenyl)phenanthren-9-yl)propanoate (5qa)

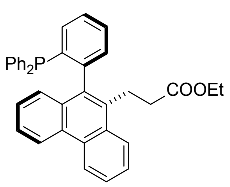

5qa

General Procedure **B**, a 25.0 mL dry Schlenk tube with a stirring bar was added with $[Ir(cod)Cl]_2$ (3.4 mg, 0.01 mmol, 0.05 equiv) and **L4** (8.0 mg, 0.022 mmol, 0.11 equiv) in 2.0 ml toluene under Ar. The formed mixture was stirred at room temperature for 1h. Then **1q** (87.6 mg, 0.2 mmol) and **4a** (100.0 mg, 1.0 mmol) was added under argon, the resulting mixture was stirred at 150 ºC. After 48 h, the solvent was removed under vaccum. The crude product purified by column chromatography on silica gel (PE/EtOAc = 20/1) to afford the corresponding product **5qa** as a white solid (54.9 mg, 51%). **^{1}H NMR (500 MHz, $CDCl_3$)** δ 8.03 (d, J = 7.6 Hz, 1H), 7.47 (td, J = 7.5, 1.7 Hz, 1H), 7.41 (td, J = 7.6, 1.6 Hz, 1H), 7.39 – 7.35 (m, 1H), 7.34 – 7.26 (m, 5H), 7.25 – 7.15 (m, 5H), 7.15 – 7.10 (m, 3H), 7.04 (d, J = 11.6 Hz, 1H), 7.02 – 6.97 (m, 2H), 6.95 (d, J = 8.5 Hz, 1H), 4.06 (q, J = 7.2 Hz, 2H), 2.63 – 2.56 (m, 1H), 2.54 – 2.47 (m, 1H), 2.46 – 2.39 (m, 1H), 2.36 – 2.29 (m, 1H), 1.20 (t, J = 7.1 Hz, 3H).;**^{13}C NMR (126 MHz, $CDCl_3$)** δ 172.7, 159.5, 157.5, 144.3 (d, J = 32.9 Hz), 138.8 (d, J = 11.6 Hz), 136.7 (d, J = 4.6 Hz), 136.6 (d, J = 4.6 Hz), 134.2, 133.8 (d, J = 1.4 Hz), 133.6 (d, J = .2 Hz), 131.1 (d, J = 5.8 Hz), 129.1, 128.44, 128.38, 128.36, 128.3, 128.12, 128.07, 128.0, 126.5, 126.4 (d, J = 3.5 Hz), 125.2 (d, J = 1.2 Hz), 122.1 (d, J = 16.8 Hz), 120.1 (d, J = 5.2 Hz), 109.8 (d, J = 19.7 Hz), 60.3, 34.8, 28.8, 14.2; **^{31}P NMR (202 MHz, $CDCl_3$)** δ -15.33; **IR (film)**: 3894, 3661, 3576, 2360, 1276 cm^{-1}; **HRMS m/z (ESI)**: calcd for $C_{37}H_{32}O_2P$ $[M+H]^+$: 539.2134, found 539.2140.

The enantiomeric excess of **5qa** was determined by chiral HPLC analysis on Ciralpak IA column. Conditions: hexane/isopropanol = 99: 1 flow rate = 0.5 L/min, 25 ºC,

uv-vis detection at λ = 254 nm, 90% ee, t_{R1} = 11.2 min (major), t_{R2} = 11.9 min (minor) ; $[\alpha]_D^{25}$ = -13.56 (c = 0.3, $CHCl_3$).

Ethyl (R)-3-(1-(2-(diphenylphosphanyl)phenyl)pyren-2-yl)propanoate (5ra)

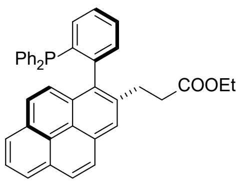

5ra

General Procedure B, a 25.0 mL dry Schlenk tube with a stirring bar was added with $[Ir(cod)Cl]_2$ (3.4 mg, 0.01 mmol, 0.05 equiv) and **L4** (8.0 mg, 0.022 mmol, 0.11 equiv) in 2.0 ml toluene under Ar. The formed mixture was stirred at room temperature for 1h. Then **1r** (92.4 mg, 0.2 mmol) and **4a** (200.0 mg, 2.0 mmol) was added under argon, the resulting mixture was stirred at 150 °C. After 48 h, the solvent was removed under vaccum. The crude product purified by column chromatography on silica gel (PE/EtOAc = 20/1) to afford the corresponding product **5ra** as a white solid (65.2 mg, 58%). **^{1}H NMR (500 MHz, $CDCl_3$)** δ 8.16 (d, J = 7.6 Hz, 1H), 8.11 – 8.02 (m, 4H), 7.95 (t, J = 7.5 Hz, 1H), 7.63 (d, J = 9.2 Hz, 1H), 7.54 (t, J = 7.4 Hz, 1H), 7.49 (t, J = 7.6 Hz, 1H), 7.41 (dd, J = 7.7, 3.6 Hz, 1H), 7.38 – 7.34 (m, 1H), 7.32 – 7.27 (m, 3H), 7.22 – 7.16 (m, 3H), 7.16 – 7.11 (m, 1H), 7.08 – 7.01 (m, 2H), 6.96 (t, J = 7.7 Hz, 2H), 4.09 (q, J = 7.1 Hz, 2H), 2.99 – 2.90 (m, 2H), 2.70 – 2.61 (m, 1H), 2.58 – 2.49 (m, 1H), 1.21 (t, J = 7.1 Hz, 3H);**^{13}C NMR (126 MHz, $CDCl_3$)** δ 173.0 , 145.3 (d, J = 32.9 Hz), 138.8 (d, J = 12.1 Hz), 136.9 (d, J = 12.1 Hz), 136.7 (d, J = 1.4 Hz), 136.4, 136.32, 136.26, 134.2 (d, J = 1.7 Hz), 133.9 (d, J = 20.8 Hz), 133.6 (d, J = 19.7 Hz), 131.2 (d, J = 5.8 Hz), 131.0 (d, J = 19.1 Hz), 130.5, 130.1 (d, J = 2.3 Hz), 129.1, 128.33 , 128.32 , 128.28 , 128.1 (d, J = 6.9 Hz), 128.0, 127.3 (d, J = 17.3 Hz), 126.8, 125.7 (d, J = 17.9 Hz), 124.8 (d, J = 12.7 Hz), 124.6, 124.4, 123.1, 60.3, 35.4, 29.3 (d, J = 2.9 Hz), 14.2; **^{31}P NMR (202 MHz, $CDCl_3$)** δ -15.27; **IR (film)**: 3910, 3576, 2360, 1276 cm^{-1}; **HRMS m/z (ESI)**: calcd for $C_{39}H_{32}O_2P$ $[M+H]^+$: 563.2134, found 563.2120.

The enantiomeric excess of **5ra** was determined by chiral HPLC analysis on Ciralpak IB column. Conditions: hexane/isopropanol = 99: 1 flow rate = 1.0 L/min, 25 °C, uv-vis detection at λ = 220 nm, 86% ee, t_{R1} = 19.9 min (minor), t_{R2} = 27.1 min (major); $[\alpha]_D^{25}$ = -12.19 (c = 0.53, $CHCl_3$).

Ethyl (R)-3-(3'-(diphenylphosphanyl)-[1,2'-binaphthalen]-2-yl)propanoate (5sa)

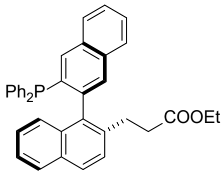

5sa

General Procedure **B**, a 25.0 mL dry Schlenk tube with a stirring bar was added with $[Ir(cod)Cl]_2$ (3.4 mg, 0.01 mmol, 0.05 equiv) and **L4** (8.0 mg, 0.022 mmol, 0.11 equiv) in 2.0 ml toluene under Ar. The formed mixture was stirred at room temperature for 1h. Then **1s** (87.6 mg, 0.2 mmol) and **4a** (100.0 mg, 1.0 mmol) was added under argon, the resulting mixture was stirred at 150 °C. After 48 h, the solvent was removed under vaccum. The crude product purified by column chromatography on silica gel (PE/EtOAc = 20/1) to afford the corresponding product **5sa** as a white solid (83.9 mg, 78%). **^{1}H NMR (500 MHz, $CDCl_3$)** δ 7.82 (t, *J* = 9.2 Hz, 1H), 7.79 – 7.73 (m, 3H), 7.71 (d, *J* = 4.1 Hz, 1H), 7.55 – 7.45 (m, 2H), 7.37 (d, *J* = 8.4 Hz, 1H), 7.34 – 7.25 (m, 4H), 7.25 – 7.18 (m, 2H), 7.15 (t, *J* = 7.2 Hz, 1H), 7.09 (t, *J* = 7.6 Hz, 2H), 7.05 – 6.97 (m, 3H), 6.95 (d, *J* = 8.4 Hz, 1H), 4.01 (q, *J* = 7.1 Hz, 2H), 2.65 – 2.51 (m, 2H), 2.50 – 2.42 (m, 1H), 2.41 – 2.33 (m, 1H), 1.14 (t, *J* = 7.2 Hz, 3H); **^{13}C NMR (126 MHz, $CDCl_3$)** δ 172.9, 141.2 (d, *J* = 32.4 Hz), 137.5 (d, *J* = 13.3 Hz), 136.8 (d, *J* = 6.4 Hz), 136.6 (d, *J* = 1.8 Hz), 136.5 (d, *J* = 11.6 Hz), 136.3 (d, *J* = 11.6 Hz), 134.4, 134.0 (d, *J* = 10.4 Hz), 133.9 (d, *J* = 11.6 Hz), 133.4, 133.3 (d, *J* = 2.9 Hz), 132.7, 131.8, 129.5 (d, *J* = 5.8 Hz), 128.5, 128.4, 128.3 (d, *J* = 9.2 Hz), 128.1 (d, *J* = 3.5 Hz), 128.0, 127.6 (d, *J* = 24.9 Hz), 127.0, 126.6, 126.3 (d, *J* = 29.5 Hz), 125.5, 124.8, 60.2, 35.1, 28.9 (d, *J* = 2.9 Hz), 14.1; **^{31}P NMR (202 MHz, $CDCl_3$)** δ -14.28; **IR (film)**: 3699, 3576, 2360, 1276 cm^{-1}; **HRMS m/z (ESI)**: calcd for $C_{37}H_{32}O_2P$ $[M+H]^+$: 539.2134, found 539.2123.

The enantiomeric excess of **5sa** was determined by chiral HPLC analysis on Ciralpak IA column. Conditions: hexane/isopropanol = 99: 1 flow rate = 1.0 L/min, 25 oC, uv-vis detection at λ = 220 nm, 91% ee, t_{R1} = 8.7 min (minor), t_{R2} = 10.2 min (major); $[\alpha]_D^{25}$ = 36.11 (*c* = 0.57, $CHCl_3$).

Ethyl (S)-3-(4-(2-(diphenylphosphanyl)phenyl)quinolin-3-yl)propanoate (5ta)

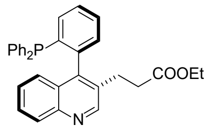

5ta

General Procedure **B**, a 25.0 mL dry Schlenk tube with a stirring bar was added with [Ir(cod)Cl]$_2$ (3.4 mg, 0.01 mmol, 0.05 equiv) and **L4** (8.0 mg, 0.022 mmol, 0.11 equiv) in 2.0 ml toluene under Ar. The formed mixture was stirred at room temperature for 1h. Then **1t** (77.8 mg, 0.2 mmol) and **4a** (100.0 mg, 1.0 mmol) was added under argon, the resulting mixture was stirred at 150 °C. After 48 h, the solvent was removed under vaccum. The crude product purified by column chromatography on silica gel (PE/EtOAc = 20/1) to afford the corresponding product **5ta** as a white solid (46.9 mg, 48%). **^{1}H NMR (500 MHz, CDCl$_3$)** δ 8.82 (s, 1H), 8.05 (d, *J* = 8.4 Hz, 1H), 7.54 (ddd, *J* = 8.4, 6.8, 1.4 Hz, 1H), 7.50 (td, *J* = 7.5, 1.6 Hz, 1H), 7.45 (td, *J* = 7.5, 1.5 Hz, 1H), 7.34 (ddd, *J* = 7.6, 3.5, 1.5 Hz, 1H), 7.32 – 7.27 (m, 3H), 7.22 – 7.16 (m, 4H), 7.15 – 7.11 (m, 3H), 7.02 – 6.94 (m, 3H), 4.06 (q, *J* = 7.1 Hz, 2H), 2.71 – 2.65 (m, 2H), 2.52 – 2.45 (m, 1H), 2.42 – 2.34 (m, 1H), 1.20 (t, *J* = 7.2 Hz, 3H); **^{13}C NMR (126 MHz, CDCl$_3$)** δ 172.4, 151.5, 146.6, 145.9 (d, *J* = 6.4 Hz), 142.1 (d, *J* = 32.9 Hz), 137.7 (d, *J* = 14.4 Hz), 136.2 (d, *J* = 11.6 Hz), 135.9 (d, *J* = 11.6 Hz), 134.3, 133.9, 133.8, 133.6, 131.0, 130.0 (d, *J* = 6.4 Hz), 129.2, 129.1, 128.6 (d, *J* = 2.3 Hz), 128.64 (d, *J* = 5.2 Hz), 128.56 (d, J = 5.2 Hz), 128.2 (d, *J* = 6.9 Hz), 127.7 (d, *J* = 2.9 Hz), 126.2 (d, *J* = 11.6 Hz), 60.5, 34.7, 26.4 (d, *J* = 2.9 Hz), 14.2; **^{31}P NMR (202 MHz, CDCl$_3$)** δ -15.15; **IR (film)**: 3911, 3577, 2360, 1276 cm^{-1}; **HRMS m/z (ESI)**: calcd for $C_{32}H_{29}NO_2P$ [M+H]$^+$: 490.1930, found 490.1919.

The enantiomeric excess of **5ta** was determined by chiral HPLC analysis on Ciralpak IB column. Conditions: hexane/isopropanol = 90: 10 flow rate = 1.0 L/min, 25 °C, uv-vis detection at λ = 220 nm, 91% ee, t_{R1} = 6.2 min (major), t_{R2} = 6.8 min (minor); $[\alpha]_D^{25}$ = -9.94 (*c* = 0.79, CHCl$_3$).

Ethyl (R)-3-(1-(2-(diphenylphosphanyl)phenyl)dibenzo[b,d]furan-2-yl)propanoate (5ua)

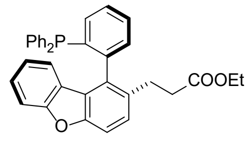

5ua

General Procedure **B**, a 25.0 mL dry Schlenk tube with a stirring bar was added with [Ir(cod)Cl]$_2$ (3.4 mg, 0.01 mmol, 0.05 equiv)

and **L4** (8.0 mg, 0.022 mmol, 0.11 equiv) in 2.0 ml toluene under Ar. The formed mixture was stirred at room temperature for 1h. Then **1u** (85.6 mg, 0.2 mmol) and **4a** (200.0 mg, 2.0 mmol) was added under argon, the resulting mixture was stirred at 150 °C. After 48 h, the solvent was removed under vaccum. The crude product purified by column chromatography on silica gel (PE/EtOAc = 20/1) to afford the corresponding product **5ua** as a white solid (60.2 mg, 57%). **^{1}H NMR (500 MHz, $CDCl_3$)** δ 7.53 – 7.47 (m, 2H), 7.47 – 7.41 (m, 2H), 7.36 – 7.31 (m, 2H), 7.31 – 7.26 (m, 5H), 7.24 – 7.19 (m, 2H), 7.01 – 6.96 (m, 1H), 6.95 – 6.87 (m, 5H), 6.35 – 6.29 (m, 1H), 4.05 (q, *J* = 7.2 Hz, 2H), 2.71 – 2.63 (m, 1H), 2.60 – 2.52 (m, 1H), 2.48 – 2.40 (m, 1H), 2.34 – 2.25 (m, 1H), 1.18 (t, *J* = 7.2 Hz, 3H); **^{13}C NMR (126 MHz, $CDCl_3$)** δ 172.9, 156.3, 154.3, 144.0 (d, *J* = 32.4 Hz), 138.1(d, *J* = 13.3 Hz), 136.3 (d, *J* = 11.6 Hz), 136.1 (d, *J* = 11.6 Hz), 135.4 (d, *J* = 7.5 Hz), 134.0, 133.9, 133.7, 133.1, 129.9 (d, *J* = 5.2 Hz), 129.5, 128.5 (d, *J* = 13.9 Hz), 128.4 (d, *J* = 2.9 Hz), 128.2, 127.9 (d, *J* = 6.9 Hz), 127.4, 126.5, 124.4, 123.6 (d, *J* = 2.9 Hz), 122.1(d, *J* = 2.9 Hz), 111.0 (d, *J* = 28.9 Hz), 60.2, 35.6, 27.6 (d, *J* = 3.5 Hz), 14.2; **^{31}P NMR (202 MHz, $CDCl_3$)** δ -14.16; **IR (film)**: 3599, 3361, 2360, 1276 cm^{-1}; **HRMS m/z (ESI)**: calcd for $C_{35}H_{30}O_3P$ $[M+H]^+$: 529.1927, found 529.1917.

The enantiomeric excess of **5ua** was determined by chiral HPLC analysis on Ciralpak IB column. Conditions: hexane/isopropanol = 99.5: 0.5 flow rate = 0.5 L/min, 25 °C, uv-vis detection at λ = 220 nm, 90% ee, t_{R1} = 22.8 min (major), t_{R2} = 26.1 min (minor); $[\alpha]_D^{25}$ = -44.16 (*c* = 2.12, $CHCl_3$).

Ethyl (R)-3-(1-(2-(bis(4-fluorophenyl)phosphanyl)phenyl)naphthalen-2-yl)propanoate (5wa)

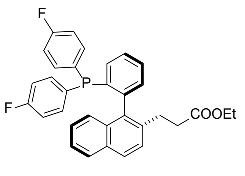

5wa

General Procedure **B**, a 25.0 mL dry Schlenk tube with a stirring bar was added with $[Ir(cod)Cl]_2$ (3.4 mg, 0.01 mmol, 0.05 equiv) and **L4** (8.0 mg, 0.022 mmol, 0.11 equiv) in 2.0 ml toluene under Ar. The formed mixture was stirred at room temperature for 1h. Then **1w** (84.8 mg, 0.2 mmol) and **4a** (100.0 mg, 1.0 mmol) was added under argon,

the resulting mixture was stirred at 150 °C. After 48 h, the solvent was removed under vaccum. The crude product purified by column chromatography on silica gel (PE/EtOAc = 20/1) to afford the corresponding product **5wa** as a white solid (42.0 mg, 40%). **^{1}H NMR (500 MHz, $CDCl_3$)** δ 7.82 (d, J = 8.5 Hz, 1H), 7.77 (d, J = 7.6 Hz, 1H), 7.49 (td, J = 7.5, 1.4 Hz, 1H), 7.43 (td, J = 7.6, 1.6 Hz, 1H), 7.37 (d, J = 8.4 Hz, 1H), 7.34 – 7.30 (m, 1H), 7.26 – 7.22 (m, 2H), 7.18 – 7.12 (m, 2H), 7.10 – 7.05 (m, 1H), 7.00 (t, J = 8.4 Hz, 2H), 6.96 – 6.90 (m, 2H), 6.88 (d, J = 8.4 Hz, 1H), 6.81 (t, J = 8.8 Hz, 2H), 4.11 – 4.01 (m, 2H), 2.68 – 2.55 (m, 2H), 2.52 – 2.43 (m, 1H), 2.40 – 2.32 (m, 1H), 1.20 (t, J = 7.2 Hz, 3H); **^{13}C NMR (126 MHz, $CDCl_3$)** δ 172.8, 163.1 (d, J = 248.5 Hz), 144.8 (d, J = 32.9 Hz), 138.2 (d, J = 12.1 Hz), 137.0 (d, J = 7.5 Hz), 136.0 (d, J = 1.6 Hz), 135.7 (d, J = 8.1 Hz), 135.5 (d, J = 8.0 Hz), 135.4 (d, J = 7.9 Hz), 133.6, 132.9 (d, J = 2.0 Hz), 132.1 (dd, J = 12.3, 3.4 Hz), 131.9 (dd, J = 12.3, 3.4 Hz), 131.8, 131.0 (d, J = 5.6 Hz), 129.3, 128.1 (d, J = 31.2 Hz), 127.6, 126.4 (d, J = 23.1 Hz), 125.3 (d, J = 76.9 Hz), 115.6 (dd, J = 20.8, 7.5 Hz), 115.3 (dd, J = 21.1, 7.8 Hz), 60.3 , 35.1, 28.9 (d, J = 2.9 Hz), 14.1; **^{31}P NMR (202 MHz, $CDCl_3$)** δ -17.50; **^{19}F NMR (471 MHz, $CDCl_3$)** δ -112.66 (d, J = 4.3 Hz), -112.79 (d, J = 4.3 Hz); **IR (film)**: 3699, 3622, 2360, 1276 cm^{-1}; **HRMS m/z (ESI)**: calcd for $C_{33}H_{28}F_2O_2P$ $[M+H]^+$: 525.1789, found 525.1777.

The enantiomeric excess of **5wa** was determined by chiral HPLC analysis on Ciralpak IB column. Conditions: hexane/isopropanol = 99: 1 flow rate = 1.0 L/min, 25 °C, uv-vis detection at λ = 220 nm, 95% ee, t_{R1} = 8.6 min (minor), t_{R2} = 9.3 min (major); $[\alpha]_D^{25}$ = 24.11 (c = 0.38, $CHCl_3$).

Ethyl (R)-3-(1-(2-(di(thiophen-2-yl)phosphanyl)phenyl)naphthalen-2-yl)propanoate (5xa)

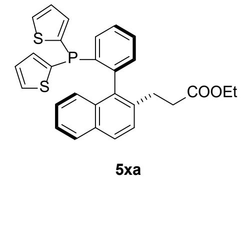

5xa

General Procedure **B**, a 25.0 mL dry Schlenk tube with a stirring bar was added with $[Ir(cod)Cl]_2$ (3.4 mg, 0.01 mmol, 0.05 equiv) and **L4** (8.0 mg, 0.022 mmol, 0.11 equiv) in 2.0 ml toluene under Ar. The formed mixture was stirred at room temperature for 1h.

Then **1x** (80.0 mg, 0.2 mmol) and **4a** (100.0 mg, 1.0 mmol) was added under argon, the resulting mixture was stirred at 150 °C. After 48 h, the solvent was removed under vaccum. The crude product purified by column chromatography on silica gel (PE/EtOAc = 20/1) to afford the corresponding product **5xa** as a white solid (37.1 mg, 37%). **^{1}H NMR (500 MHz, CDCl$_3$)** δ 7.84 (d, *J* = 8.4 Hz, 1H), 7.79 (d, *J* = 8.1 Hz, 1H), 7.63 – 7.58 (m, 1H), 7.55 (d, *J* = 5.0 Hz, 1H), 7.51 – 7.44 (m, 2H), 7.42 – 7.37 (m, 2H), 7.36 – 7.31 (m, 1H), 7.25 – 7.20 (m, 1H), 7.14 – 7.08 (m, 2H), 7.08 – 7.04 (m, 1H), 6.98 (d, *J* = 8.5 Hz, 1H), 6.83 – 6.79 (m, 1H), 6.76 (dd, *J* = 6.5, 3.1 Hz, 1H), 4.06 (q, *J* = 7.1 Hz, 2H), 2.66 (t, *J* = 8.1 Hz, 2H), 2.51 – 2.35 (m, 2H), 1.19 (t, *J* = 7.1 Hz, 3H); **^{13}C NMR (126 MHz, CDCl$_3$)** δ 172.9, 143.7 (d, *J* = 34.1 Hz), 139.0 (d, *J* = 7.5 Hz), 137.8 (d, *J* = 24.9 Hz), 137.6 (d, *J* = 25.4 Hz), 136.8 (d, *J* = 6.9 Hz), 136.3, 135.6 (d, *J* = 21.4 Hz), 135.4 (d, *J* = 22.0 Hz), 133.0 (d, *J* = 2.9 Hz), 132.8, 131.8, 131.5 (d, *J* = 37.0 Hz), 130.7 (d, *J* = 5.8 Hz), 129.4, 128.2 (d, *J* = 33.5 Hz), 127.9 (d, *J* = 7.5 Hz), 127.6, 127.5, 126.6, 126.2, 125.6, 124.9, 60.2, 35.2 , 28.8 (d, *J* = 2.9 Hz), 14.2; **^{31}P NMR (202 MHz, CDCl$_3$)** δ -43.41; **^{19}F NMR (471 MHz, CDCl$_3$)** δ -112.66 (d, *J* = 4.3 Hz), -112.79 (d, *J* = 4.3 Hz); **IR (film)**: 3724, 3601, 2360, 1521 cm^{-1}; **HRMS m/z (ESI)**: calcd for $C_{29}H_{26}O_2PS_2$ $[M+H]^+$: 501.1106, found 501.1095.

The enantiomeric excess of **5xa** was determined by chiral HPLC analysis on Ciralpak IB column. Conditions: hexane/isopropanol = 99: 1 flow rate = 0.5 L/min, 25 °C, uv-vis detection at λ = 220 nm, 87% ee, t_{R1} = 18.0 min (major), t_{R2} = 19.8 min (minor); $[\alpha]_D^{25}$ = -19.20 (*c* = 0.37, CHCl$_3$).

Methyl (R)-3-(1-(2-(diphenylphosphanyl)phenyl)naphthalen-2-yl)propanoate (5ab)

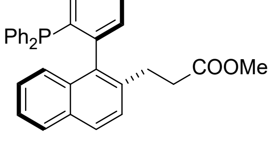

5ab

General Procedure **B**, a 25.0 mL dry Schlenk tube with a stirring bar was added with $[Ir(cod)Cl]_2$ (3.4 mg, 0.01 mmol, 0.05 equiv) and **L4** (8.0 mg, 0.022 mmol, 0.11 equiv) in 2.0 ml toluene under Ar. The formed mixture was stirred at room temperature for 1h. Then **1a** (77.6 mg, 0.2 mmol) and methyl acrylate **4b** (86.0 mg, 1.0 mmol) was added under argon, the resulting mixture was stirred at 150 °C. After

48 h, the solvent was removed under vaccum. The crude product purified by column chromatography on silica gel (PE/EtOAc = 20/1) to afford the corresponding product **5ab** as a white solid (72.6 mg, 77%). **^{1}H NMR (400 MHz, CDCl$_3$)** δ 7.81 (d, *J* = 8.5 Hz, 1H), 7.76 (d, *J* = 8.2 Hz, 1H), 7.46 (td, *J* = 7.3, 1.6 Hz, 1H), 7.40 (td, *J* = 7.5, 1.6 Hz, 1H), 7.32 (td, *J* = 8.7, 1.7 Hz, 3H), 7.30 – 7.25 (m, 3H), 7.25 – 7.21 (m, 1H), 7.21 – 7.16 (m, 2H), 7.15 – 7.12 (m, 1H), 7.12 – 7.07 (m, 2H), 7.03 – 6.94 (m, 3H), 3.58 (s, 3H), 2.66 – 2.58 (m, 1H), 2.57 – 2.49 (m, 1H), 2.48 – 2.41 (m, 1H), 2.38 – 2.28 (m, 1H); **^{13}C NMR (101 MHz, CDCl$_3$)** δ 173.3, 145.0 (d, *J* = 33.2 Hz), 138.3 (d, *J* = 12.3 Hz), 137.4 (d, *J* = 6.8 Hz), 136.9 (d, *J* = 8.2 Hz), 136.8 (d, *J* = 8.6 Hz), 135.9, 134.3, 133.7 (d, *J* = 5.9 Hz), 133.5 (d, *J* = 6.4 Hz), 133.0, 131.8, 130.8 (d, *J* = 5.9 Hz), 129.1, 128.3, 128.2 (d, *J* = 2.3 Hz), 128.1 (d, J = 6.8 Hz), 128.0, 127.7 (d, *J* = 28.6 Hz), 126.4 (d, *J* = 10.0 Hz), 125.2 (d, *J* = 64.9 Hz), 51.4, 34.8, 28.8; **^{31}P NMR (162 MHz, CDCl$_3$)** δ -15.45; **IR (film)**: 3911, 3577, 3099, 2360, 1276 cm^{-1}; **HRMS m/z (ESI)**: calcd for $C_{32}H_{28}O_2P$ [M+H]$^+$: 475.1821, found 475.1810.

The enantiomeric excess of **5ab** was determined by chiral HPLC analysis on Ciralpak IB column. Conditions: hexane/isopropanol = 99: 1 flow rate = 0.5 L/min, 25 oC, uv-vis detection at λ = 220 nm, 82% ee, t_{R1} = 14.9 min (major), t_{R2} = 16.5 min (minor); $[\alpha]_D^{25}$ = -15.05 (*c* = 3.23, CHCl$_3$).

Butyl (R)-3-(1-(2-(diphenylphosphanyl)phenyl)naphthalen-2-yl)propanoate (5ac)

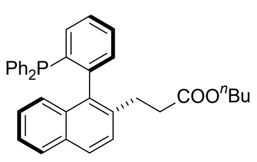

5ac

General Procedure **B**, a 25.0 mL dry Schlenk tube with a stirring bar was added with [Ir(cod)Cl]$_2$ (3.4 mg, 0.01 mmol, 0.05 equiv) and **L4** (8.0 mg, 0.022 mmol, 0.11 equiv) in 2.0 ml toluene under Ar. The formed mixture was stirred at room temperature for 1h. Then **1a** (77.6 mg, 0.2 mmol) and butyl acrylate **4c** (128.2 mg, 1.0 mmol) was added under argon, the resulting mixture was stirred at 150 °C. After 48 h, the solvent was removed under vaccum. The crude product purified by column chromatography on silica gel (PE/EtOAc = 20/1) to afford the corresponding product **5ac** as a white solid (61.1 mg, 59%). **^{1}H NMR (500 MHz, CDCl$_3$)** δ 7.83 (d, *J* = 8.5 Hz, 1H), 7.78 (d, *J* = 7.9 Hz, 1H), 7.48 (td, *J* = 7.5, 1.6 Hz, 1H), 7.42 (td, *J* = 7.6, 1.7

Hz, 1H), 7.38 – 7.32 (m, 3H), 7.31 – 7.28 (m, 3H), 7.26 – 7.23 (m, 1H), 7.21 – 7.16 (m, 3H), 7.16 – 7.11 (m, 2H), 7.11 – 7.06 (m, 1H), 7.04 – 6.96 (m, 3H), 4.04 – 3.97 (m, 2H), 2.65 – 2.52 (m, 2H), 2.50 – 2.41 (m, 1H), 2.40 – 2.32 (m, 1H), 1.57 – 1.49 (m, 2H), 1.33 – 1.28 (m, 2H), 0.89 (t, J = 7.4 Hz, 3H); **^{13}C NMR (126 MHz, $CDCl_3$)** δ 173.0, 145.0 (d, J = 33.5 Hz), 138.4 (d, J = 12.1 Hz), 137.4 (d, J = 7.5 Hz), 136.9 (d, J = 12.7 Hz), 136.1 (d, J = 2.3 Hz), 134.2 (d, J = 2.3 Hz), 133.8 (d, J = 2.9 Hz), 133.6 (d, J = 2.9 Hz), 133.0 (d, J = 2.3 Hz), 131.8, 130.8 (d, J = 5.8 Hz), 129.1, 128.31 (d, J = 1.5 Hz), 128.25 (d, J = 1.5 Hz), 128.1 (d, J = 5.2 Hz), 128.0, 127.8, 127.5, 126.5 (d, J = 2.9 Hz), 125.5, 124.9, 64.2, 35.2, 30.6, 28.9 (d, J = 2.9 Hz), 19.1, 13.7; **^{31}P NMR (202 MHz, $CDCl_3$)** δ -15.40; **IR (film)**: 3720, 3577, 2360, 1276 cm^{-1}; **HRMS m/z (ESI)**: calcd for $C_{35}H_{34}O_2P$ $[M+H]^+$: 517.2291, found 517.2297.

The enantiomeric excess of **5ac** was determined by chiral HPLC analysis on Ciralpak IB column. Conditions: hexane/isopropanol = 99: 1 flow rate = 0.5 L/min, 25 °C, uv-vis detection at λ = 220 nm, 92% ee, t_{R1} = 11.7 min (major), t_{R2} = 12.5 min (minor); $[\alpha]_D^{25}$ = -18.55 (c = 0.69, $CHCl_3$).

2,2,2-Trifluoroethyl (R)-3-(1-(2-(diphenylphosphanyl)phenyl)naphthalen-2-yl)propanoate (5ad)

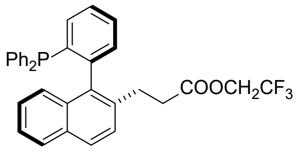

5ad

General Procedure B, a 25.0 mL dry Schlenk tube with a stirring bar was added with $[Ir(cod)Cl]_2$ (3.4 mg, 0.01 mmol, 0.05 equiv) and **L4** (8.0 mg, 0.022 mmol, 0.11 equiv) in 2.0 ml toluene under Ar. The formed mixture was stirred at room temperature for 1h. Then **1a** (77.6 mg, 0.2 mmol) and 2,2,2-trifluoroethyl acrylate **4d** (154.1 mg, 1.0 mmol) was added under argon, the resulting mixture was stirred at 150 °C. After 48 h, the solvent was removed under vaccum. The crude product purified by column chromatography on silica gel (PE/EtOAc = 20/1) to afford the corresponding product **5ad** as a white solid (55.3 mg, 51%). **^{1}H NMR (500 MHz, $CDCl_3$)** δ 7.81 (d, J = 8.5 Hz, 1H), 7.77 (d, J = 8.1 Hz, 1H), 7.49 – 7.44 (m, 1H), 7.40 (td, J = 7.6, 1.7 Hz, 1H), 7.36 – 7.30 (m, 3H), 7.29 – 7.25 (m, 3H), 7.24 – 7.21 (m, 1H), 7.19 – 7.14 (m, 3H), 7.13 – 7.08 (m, 3H), 7.02 – 6.97 (m, 3H), 4.36 (qd, J = 8.5,

1.4 Hz, 2H), 2.69 – 2.60 (m, 1H), 2.59 – 2.49 (m, 2H), 2.45 – 2.36 (m, 1H); **^{13}C NMR (126 MHz, CDCl$_3$)** δ 171.2, 144.8 (d, *J* = 32.9 Hz), 138.4 (d, *J* = 12.1 Hz), 137.5 (d, *J* = 7.5 Hz), 136.8 (d, *J* = 12.1 Hz), 136.6 (d, *J* = 12.7 Hz), 135.3 (d, *J* = 2.9 Hz), 134.3 (d, *J* = 2.3 Hz), 133.8 (d, *J* = 15.6 Hz), 133.6 (d, *J* = 16.8 Hz), 133.0 (d, *J* = 2.3 Hz), 131.9, 130.7 (d, *J* = 5.8 Hz), 129.1, 128.4 (d, *J* = 6.9 Hz), 128.32, 128.27, 128.1 (d, *J* = 6.9 Hz), 127.9, 127.6, 126.5, 126.2, 125.7, 125.1, 122.9 (q, *J* = 276.9 Hz), 60.2 (q, *J* = 36.4 Hz), 34.3, 28.5 (d, *J* = 2.3 Hz); **^{31}P NMR (202 MHz, CDCl$_3$)** δ -15.45; **^{19}F NMR (471 MHz, CDCl$_3$)** δ -73.66; **IR (film)**: 3846, 3601, 2360, 1276 cm^{-1}; **HRMS m/z (ESI)**: calcd for $C_{33}H_{27}F_3O_2P$ $[M+H]^+$: 543.1695, found 543.1684.

The enantiomeric excess of **3ad** was determined by chiral HPLC analysis on Ciralpak IB column. Conditions: hexane/isopropanol = 99: 1 flow rate = 0.5 L/min, 25 °C, uv-vis detection at λ = 220 nm, 93% ee, t_{R1} = 9.6 min (major), t_{R2} = 10.2 min (minor); $[\alpha]_D^{25}$ = -22.88 (*c* = 1.11, $CHCl_3$).

Ethyl (R)-3-(2'-(diphenylphosphanyl)-[1,1'-binaphthalen]-2-yl)propanoate (5ya)

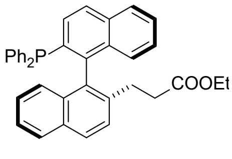

5ya

General Procedure **B**, a 25.0 mL dry Schlenk tube with a stirring bar was added with $[Ir(cod)Cl]_2$ (3.4 mg, 0.01 mmol, 0.05 equiv) and **L4** (8.0 mg, 0.022 mmol, 0.11 equiv) in 2.0 ml toluene under Ar. The formed mixture was stirred at room temperature for 1h. Then racmic racemic **1y** [6] (87.6 mg, 0.2 mmol) and **4a** (100.0 mg, 1.0 mmol) was added under argon, the resulting mixture was stirred at 150 °C. After 72 h, the solvent was removed under vaccum. The crude starting material and product purified by column chromatography on silica gel (PE/EtOAc = 20/1) to afford the corresponding product **5ya** as a white solid (46.3 mg, 43%) and (S)-**1y** (45.6 mg, 52%). **^{1}H NMR (400 MHz, CDCl$_3$)** δ 7.93 (d, *J* = 8.4 Hz, 1H), 7.91 – 7.86 (m, 2H), 7.83 (d, *J* = 8.1 Hz, 1H), 7.50 – 7.43 (m, 3H), 7.33 – 7.27 (m, 4H), 7.26 – 7.21 (m, 3H), 7.20 – 7.15 (m, 1H), 7.15 – 7.09 (m, 3H), 7.04 – 6.98 (m, 2H), 6.98 – 6.93 (m, 1H), 6.76 (dd, *J* = 8.5, 0.9 Hz, 1H), 3.96 (qd, *J* = 7.1, 1.0 Hz, 2H), 2.63 – 2.44 (m, 2H), 2.38 – 2.20 (m, 2H), 1.12 (t, *J* = 7.2 Hz, 3H); **^{13}C NMR (101 MHz, CDCl$_3$)** δ 172.9, 144.1 (d, *J* = 34.5 Hz), 137.4 (d, *J* = 12.7 Hz), 137.1 (d, *J* = 2.3 Hz), 135.4 (d, *J* = 11.4 Hz), 135.3

(d, J = 8.6 Hz), 133.70, 133.67, 133.5 (d, J = 4.1 Hz), 133.4, 133.3 (d, J = 2.3 Hz), 133.1 (d, J = 7.7 Hz), 132.0, 130.3 (d, J = 2.3 Hz), 128.4 (d, J = 5.5 Hz), 128.3 (d, J = 9.1 Hz), 128.2 (d, J = 8.2 Hz), 128.04, 128.00 (d, J = 2.3 Hz), 127.7, 126.9, 126.7 (d, J = 2.7 Hz), 126.6, 126.5 (d, J = 13.2 Hz), 125.4 (d, J = 72.7 Hz), 60.1, 34.5, 28.8, 14.1; **^{31}P NMR (162 MHz, $CDCl_3$)** δ -15.02; **IR (film)**: 3910, 3661, 3012, 2360, 1276 cm^{-1}; **HRMS m/z (ESI)**: calcd for $C_{37}H_{32}O_2P$ $[M+H]^+$: 539.2134, found 539.2122.

The enantiomeric excess of **1ya** was determined by chiral HPLC analysis on Ciralpak IB column. Conditions: hexane/isopropanol = 99: 1 flow rate = 0.5 L/min, 25 oC, uv-vis detection at λ = 220 nm, 93% ee, t_{R1} = 7.9 min (major), t_{R2} = 9.0 min (minor); $[\alpha]_D^{25}$ = -20.98 (c = 0.31, $CHCl_3$).

The enantiomeric excess of **1y** was determined by chiral HPLC analysis on Ciralpak AD-H column. Conditions: hexane/isopropanol = 99.5: 0.5 flow rate = 0.5 L/min, 25 oC, uv-vis detection at λ = 220 nm, 94% ee, t_{R1} = 14.3 min (major), t_{R2} = 16.6 min (minor).

Kinetic Resolution Studies

The selectivity factor (s) for the kinetic resolution was calculated according to the following equations derived by Kagan and Fiaud.[7]

$$s = \frac{\ln\{1 - c[1 + \text{e.e.}(\text{product})\}}{\ln\{1 - c[1 - \text{e.e.}(\text{product})\}}$$

Where

$$c = \frac{\text{e.e.}(\text{substrate})}{\text{e.e.}(\text{substrate}) + \text{e.e.}(\text{product})}$$

and e.e. is the enantiomeric excess of a specific molecule.

The s values for the KR reactions of (rac)-**1y** is as follows.

$$c = \frac{\text{e.e.}((\text{S}) - \mathbf{1y})}{\text{e.e.}((\text{S}) - \mathbf{1y}) + \text{e.e.}((\text{R}) - \mathbf{3ya})} = \frac{94\%}{94\% + 93\%} = 0.503$$

$$s = \frac{\ln\{1 - c[1 + \text{e.e.}((\text{R}) - \mathbf{5ya})\}}{\ln\{1 - c[1 - \text{e.e.}((\text{R}) - \mathbf{5ya})\}} = \frac{\ln\{1 - 0.503 * [1 + 93\%\}}{\ln\{1 - 0.503 * [1 + 93\%\}} = 99$$

Applications of the developed chiral phosphines in asymmetric catalysis

With the rapidly constructed chiral ligands in hand, we next selected some representative products including **3aa**, **5aa** and **5ya**, with different steric and electronic properties as a ligand library and tested it in transition metal-catalyzed asymmetric cross-coupling reactions. In order to reach the standard of commercialization, 99% ee of **5ya, 5aa** and **5ya** conveniently obtained through one time recrystallization with MeOH.

Construction of chiral ligand library

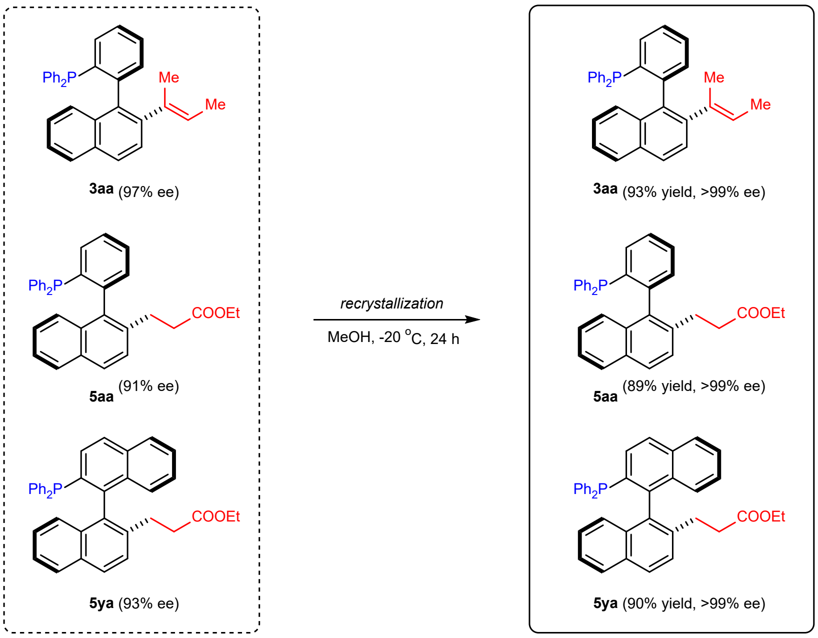

Rhodium-catalyzed asymmetric arylation reaction

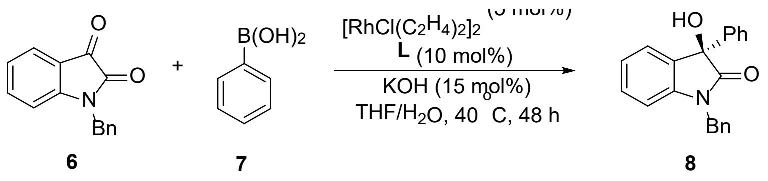

3aa	5aa	5ya
91% yield, 93% ee	12% yield, 88% ee	26% yield, 50% ee

(R)-1-Benzyl-3-hydroxy-3-phenylindolin-2-one (8)

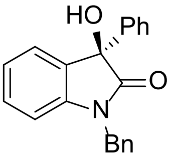

8

A solution of $[RhCl(C_2H_4)_2]_2$ (1.9 mg, 0.01 mmol Rh) and **3aa** (0.02 mmol) in THF (1.0 mL) was stirred for 10 min at room temperature. KOH (0.10 mL, 0.03 mmol; 0.3 M aqueous), isatin **6** (0.20 mmol), and $PhB(OH)_2$ **7** (0.40 mmol) were added successively with additional THF (1.0 mL). The resulting mixture was stirred for 48 h at 40 °C, and this was directly passed through a pad of silica gel with Et_2O. After removing the solvent under vacuum, the residue was purified by column chromatographyon silica gel (PE / EA = 3:1) affording 57.3 mg (91% yield) of **8** which consistent with the literature[8] as a white solid. **^{1}H NMR (400 MHz, $CDCl_3$)** δ 7.42 (dd, J = 8.1, 1.4 Hz, 2H), 7.38 – 7.27 (m, 9H), 7.23 (td, J = 7.8, 1.2 Hz, 1H), 7.05 (t, J = 7.9 Hz, 1H), 6.79 (d, J = 7.9 Hz,

1H), 5.05 (d, J = 15.7 Hz, 1H), 4.84 (d, J = 15.7 Hz, 1H), 3.29 (s, 1H); **^{13}C NMR (126 MHz, $CDCl_3$)** δ 177.6, 142.7, 140.1, 135.4, 131.5, 129.8, 128.9, 128.7, 128.4, 127.8, 127.3, 125.2, 125.0 , 123.6, 109.8, 96.0, 44.1.

The enantiomeric excess of **8** was determined by chiral HPLC analysis on Ciralpak AS-H column. Conditions: hexane/isopropanol = 80: 20 flow rate = 1.0 L/min, 25 °C, uv-vis detection at λ = 254 nm, 93% ee, t_{R1} = 23.2 min (major), t_{R2} = 39.7 min (minor). The absolute configuration was determined by contrasting with the previous study. [8]

Palladium-catalyzed asymmetric allylic alkylation reaction

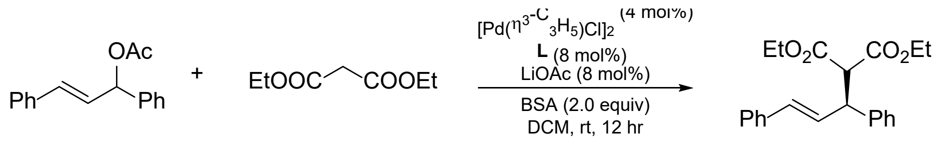

9 10 11

3aa	5aa	5ya
95% yield, 42% ee	93% yield, 92% ee	90% yield, 69% ee

Diethyl (S,E)-2-(1,3-diphenylallyl)malonate (11)

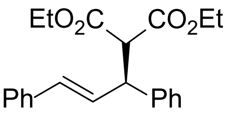

11

To a mixture of chiral ligand **5aa** (7.8 mg, 0.016 mmol), $[Pd(\eta3\text{-}C_3H_5)Cl]_2$ (2.9 mg, 0.008 mmol) and LiOAc (1.1 mg, 0.016 mmol) in DCM (1 mL) were added N,O-bis(trimethylsilyl)acetamide (BSA, 122.1 mg, 0.6 mmol) and allylic ester **9** (50.4 mg, 0.2 mmol) at room temperature under argon atmosphere. After 30 min, malonate **10** (96.0 mg, 0.6 mmol) was added. After 12 h, the reaction mixture was diluted with diethyl ether and water. The organic layer was washed with brine and dried over Na_2SO_4. The filtrate was concentrated and purified by column chromatography to afford **11** (65.5 mg, 93% yield). **^{1}H NMR (500 MHz, $CDCl_3$)** δ 7.36 – 7.32 (m, 6H), 7.32 – 7.27 (m, 2H), 7.27 – 7.20 (m, 2H), 6.51 (d, J = 15.7 Hz, 1H), 6.38 (dd, J = 15.7, 8.6 Hz, 1H), 4.33 – 4.27 (m, 1H), 4.21 (q, J = 7.2 Hz, 2H), 4.01 (qd, J = 7.1, 2.4 Hz, 2H), 3.96 (d, J = 11.0 Hz, 1H), 1.24 (t, J = 7.1 Hz, 3H), 1.04 (t, J = 7.1 Hz, 3H); **^{13}C NMR (126 MHz, $CDCl_3$)** δ 167.8, 167.4, 140.3, 136.8, 131.6, 129.3, 128.6, 128.4, 128.0, 127.5, 127.1, 126.3, 61.5, 61.3, 57.7, 49.2, 14.1, 13.7.

The enantiomeric excess of **11** was determined by chiral HPLC analysis on Ciralpak IA column. Conditions: hexane/isopropanol = 95: 5 flow rate = 1.0 L/min, 25 °C, uv-vis detection at λ = 254 nm, 92% ee, t_{R1} = 9.5 min (minor), t_{R2} = 11.9 min (major). The absolute configuration was determined by contrasting with the previous study. [9]

Palladium-catalyzed enantioselective Suzuki-Miyaura cross-coupling reaction

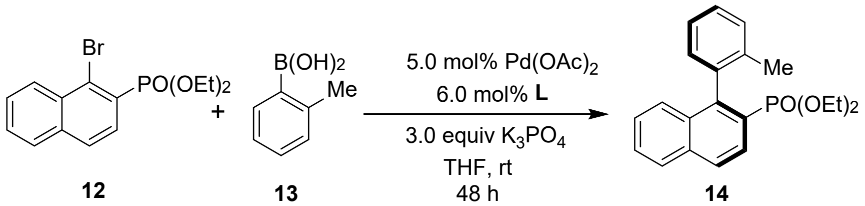

12 **13** **14**

**3aa**	**5aa**	**5ya**
65% yield, 77% ee	73% yield, 82% ee	80% yield, 93% ee

Diethyl (S)-(1-(o-tolyl)naphthalen-2-yl)phosphonate (14)

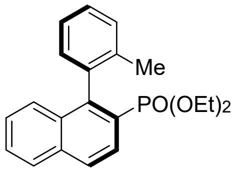

14

A flame-dried resealable Schlenk tube was charged with the aryl halide **12** (34.2 mg, 0.1 mmol, 1.0 equiv), arylboronic acid **13** (40.5 mg, 0.3 mmol, 3.0 equiv), and K_3PO_4 (63.6 mg, 0.3 mmol, 3.0 equiv). The Schlenk tube was capped with a rubber septum, and twice evacuated and backfilled with argon. A solution of $Pd(OAc)_2$ (1.1 mg, 0.005 mmol, 0.05 equiv) and phosphine **5ya** (**5ya**/Pd = 1.2) in 1/2 of the total amount of THF (0.4 mL total per mmol aryl bromide) was sonicated for about 30 seconds and injected into the Schlenk tube followed by addition of the rest of toluene. The septum was replaced with a teflon screwcap. The Schlenk tube was sealed, and the mixture was stirred at the room temperature for 48h. The reaction mixture was then cooled to room temperature, diluted with ethyl acetate, filtered, and concentrated. The crude material was purified by column chromatographyon silica gel affording 28.3 mg (80 % yield) of **14** which consistent with the literature[10] as colorless oil. **^{1}H NMR (400 MHz, $CDCl_3$)** δ 8.09 (dd, J = 12.1, 8.6 Hz, 1H), 7.95 – 7.87 (m, 2H), 7.58 – 7.51 (m, 1H), 7.41 – 7.33 (m, 2H), 7.33 – 7.26 (m, 3H), 7.24 – 7.18 (m, 1H), 3.97 – 3.73 (m, 4H), 1.92 (s, 3H), 1.18 (dt, J = 11.4, 7.1 Hz, 6H); **^{13}C NMR (101 MHz, $CDCl_3$)** δ 144.9 (d, J = 10.4 Hz), 137.8 (d, J = 5.1 Hz), 137.6, 135.0 (d, J = 2.7 Hz), 132.4 (d, J = 16.3

Hz), 130.7, 129.3, 128.3 (d, J = 10.3 Hz), 128.0 (d, J = 2.0 Hz), 127.8, 127.3 (d, J = 14.5 Hz), 127.1, 126.7 (d, J = 1.5 Hz), 125.8, 124.9, 123.9, 61.8 (d, J = 6.4 Hz), 61.7 (d, J = 5.9 Hz), 20.0, 16.3 (d, J = 6.6 Hz), 16.1 (d, J = 6.7 Hz); **^{31}P NMR (162 MHz, $CDCl_3$)** δ 17.9.

The enantiomeric excess of **14** was determined by chiral HPLC analysis on Ciralpak OD-H column. Conditions: hexane/isopropanol = 90: 10 flow rate = 0.5 L/min, 25 ºC, uv-vis detection at λ = 254 nm, 93% ee, t_{R1} = 11.2 min (minor), t_{R2} = 12.3 min (major). The absolute configuration was determined by contrasting with the previous study. [10]

2.4 Mechanistic Studies

2.4.1 Isolation of iridium complex 15

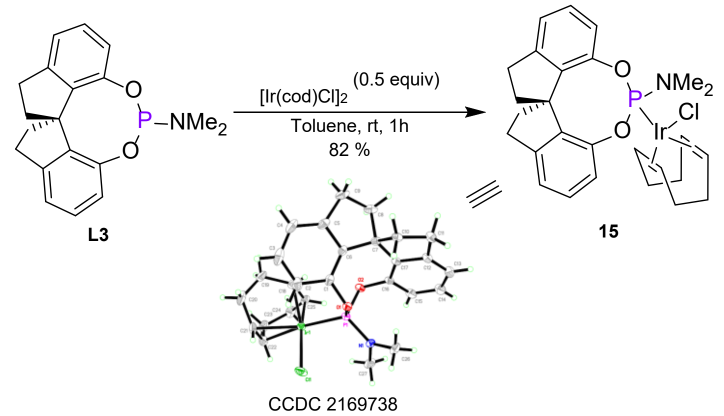

A 10.0 mL Schlenk tube with a stirring bar was added with **L3** (0.1 mmol, 32.5 mg), $[Ir(cod)Cl]_2$ (0.05 mol, 3), TMEDA (0.2 mmol, 33.6 mg) and Toluene (1.0 mL) under argon. The resulting mixture was stirred at rt under Ar for 1 h. Then the mixture was allowed to concentrate in vacuo affording the mixture. The prue product **15** was purified by column chromatographyon silica gel (PE/ETOAc = 5/1) affording 56.7 mg (82 %) as orange crystal. **^{1}H NMR (500 MHz, $CDCl_3$)** δ 7.73 (d, J = 8.1 Hz, 1H), 7.27 (t, J = 7.6 Hz, 1H), 7.17 (t, J = 7.7 Hz, 1H), 7.13 (d, J = 7.5 Hz, 1H), 7.08 (d, J = 7.3 Hz, 1H), 6.66 (d, J = 7.9 Hz, 1H), 5.42 – 5.27 (m, 2H), 3.43 – 3.32 (m, 1H), 3.17 – 3.02 (m, 2H), 2.85 (ddd, J = 16.0, 10.6, 7.9 Hz, 2H), 2.59 (d, J = 10.1 Hz, 6H), 2.48 – 2.40 (m, 1H), 2.32 – 2.16 (m, 4H), 2.10 – 1.98 (m, 2H), 1.98 – 1.90 (m, 2H), 1.90 – 1.81 (m, 2H), 1.80 – 1.70 (m, 1H), 1.70 – 1.57 (m, 2H), 1.52 – 1.41 (m, 2H), 1.36 – 1.23 (m, 1H), 1.18 – 0.94 (m, 1H), 0.93 – 0.83 (m, 1H); **^{13}C NMR (126 MHz, $CDCl_3$)** δ 146.6 (d, J = 1.4 Hz), 145.9 (d, J = 2.9 Hz), 145.8 (d, J = 13.3 Hz), 145.3 (d, J = 2.3 Hz), 140.7 (d, J = 3.5 Hz), 140.5 (d, J = 2.9 Hz), 128.3 (d, J = 2.3 Hz), 127.8 (d, J = 2.9 Hz), 124.4 (d, J = 4.0 Hz), 121.6 (d, J = 2.9 Hz), 121.4 (d, J = 2.3 Hz), 121.0 (d, J = 2.9 Hz), 101.4 (dd, J = 18.8, 13.6 Hz), 58.8 , 53.5 (d, J = 2.3 Hz), 53.0 (d, J = 2.3 Hz), 38.3 (d, J = 32.9 Hz), 37.3 (d, J = 10.4 Hz), 33.3 (dd, J = 73.1, 3.8 Hz), 30.6 (d, J = 17.9 Hz), 28.9 (dd, J = 64.4, 3.2 Hz); **^{31}P NMR (202 MHz, $CDCl_3$)** δ 94.15.

2.4.2 Test of the reactivity of 15 and 3aa for C–H activation of 1a

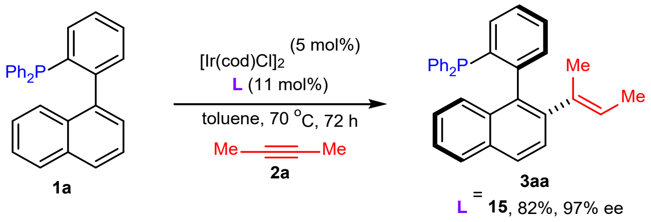

General Procedure **A**, a 25.0 mL dry Schlenk tube with a stirring bar was added with **15** (15.2 mg, 0.022 mmol, 0.11 equiv) in 2.0 ml toluene under Ar. Then **1a** (77.6 mg, 0.2 mmol) and **2a** (2-butyne, 54.0 mg, 1.0 mmol) was added under argon, the resulting mixture was stirred at 70 °C. After 72 h, the solvent was removed under vaccum. The crude product purified by column chromatography on silica gel (PE/DCM = 10/1) to afford the corresponding product **3aa** (89.6 mg, 82%, 97% ee).

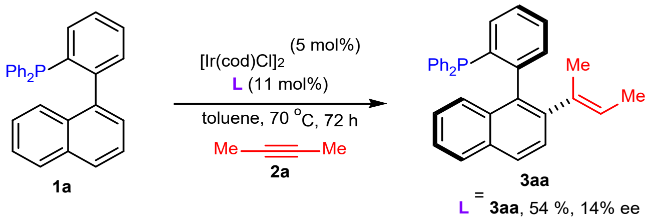

General Procedure **A**, a 25.0 mL dry Schlenk tube with a stirring bar was added with $[Ir(cod)Cl]_2$ (3.4 mg, 0.01 mmol, 0.05 equiv) and **3aa** (9.7 mg, 0.022 mmol, 0.11 equiv) in 2.0 ml toluene under Ar. The formed mixture was stirred at room temperature for 1h. Then **1a** (77.6 mg, 0.2 mmol) and **2a** (2-butyne, 54.0 mg, 1.0 mmol) was added under argon, the resulting mixture was stirred at 70 °C. After 72 h, the solvent was removed under vaccum. The crude product purified by column chromatography on silica gel (PE/DCM = 10/1) to afford the corresponding product **3aa** as a white solid (23.9 mg, 54%, 14% ee).

2.4.3 Observation of H/D exchange during the reaction of d-1a with alkyne 2a.

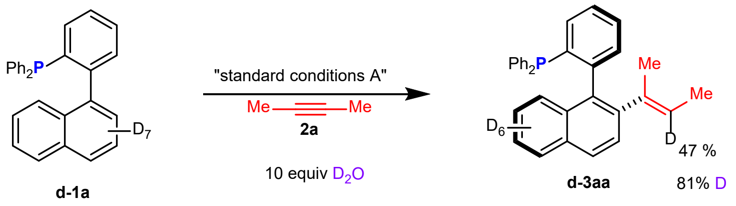

General Procedure A, a 25.0 mL dry Schlenk tube with a stirring bar was added with $[Ir(cod)Cl]_2$ (6.7 mg, 0.01 mmol, 0.05 equiv) and **L3** (7.3 mg, 0.022 mmol, 0.11 equiv) in 2.0 ml toluene under Ar. The formed mixture was stirred at room temperature for 1h. Then **d-1a** (79.0 mg, 0.2 mmol) and **2a** (54.0 mg, 1.0 mmol) was added under argon, the resulting mixture was stirred at 70 °C. After 72 h, the solvent was removed under vaccum. The crude product purified by column chromatography on silica gel (PE/DCM = 10/1) to afford the corresponding product **d-3aa** as a white solid (71.8 mg, 80%, 97% ee).

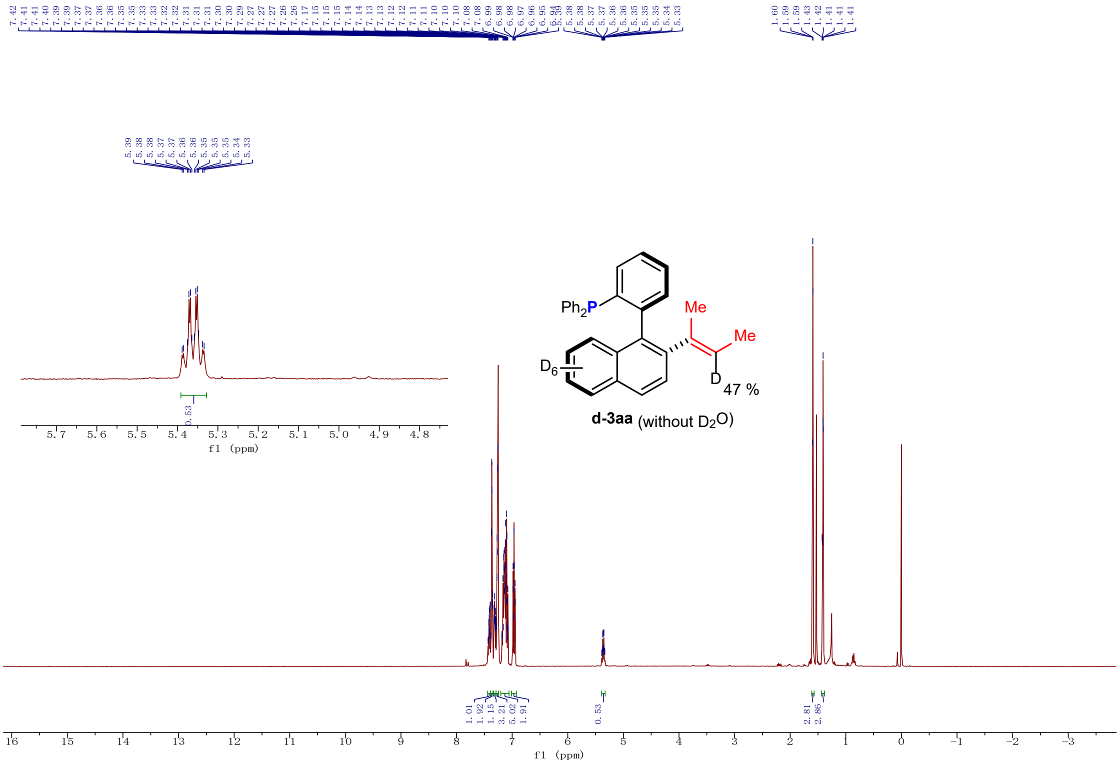

Supplementary Fig. 1. ^{1}H NMR spectra (400 MHz, $CDCl_3$, 25 °C) of **d-3aa**

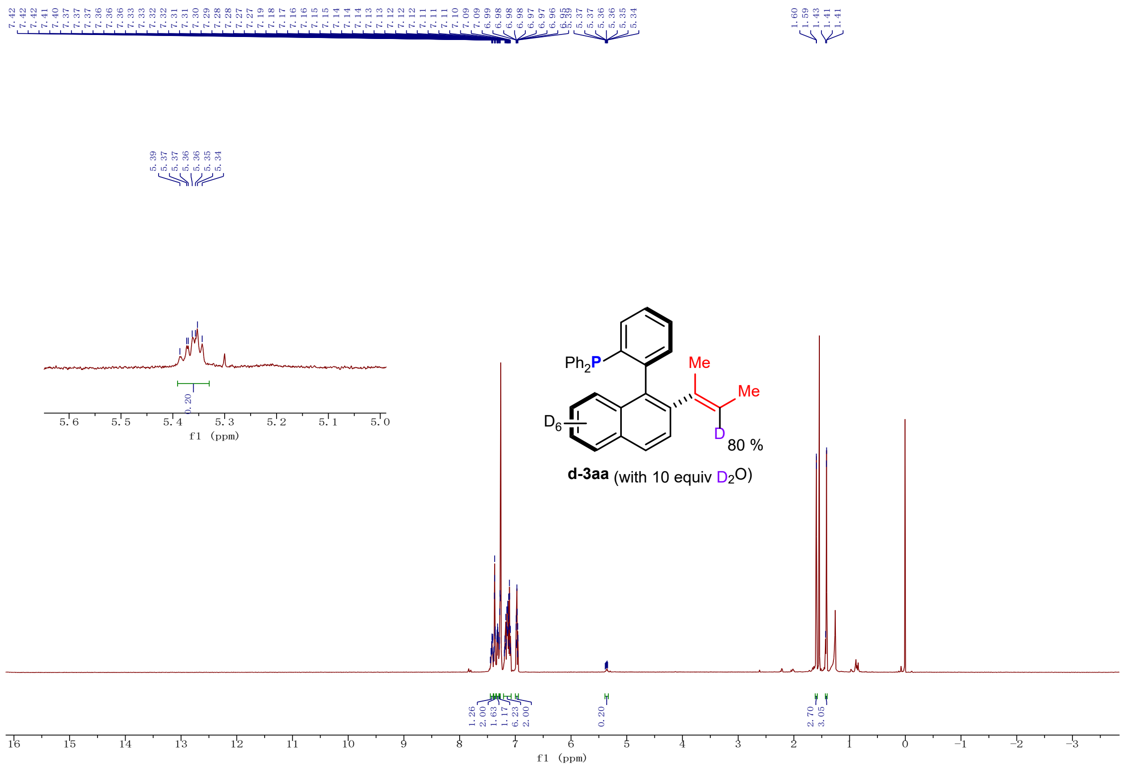

Supplementary Fig. 2. ^{1}H NMR spectra (400 MHz, CDCl₃, 25 °C) of **d-3aa**

2.4.4 Kinetic isotope effect (KIE) experiments

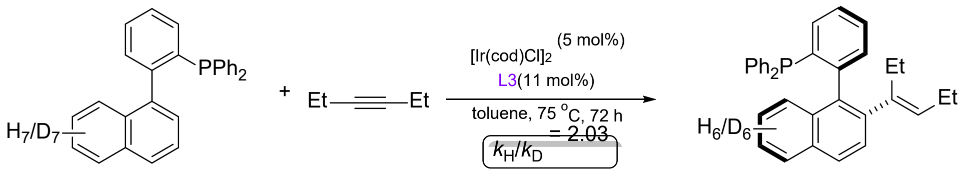

1a/d-1a = 1/1 **2b** **3ab/d-3ab**

General Procedure **A**, a 25.0 mL dry Schlenk tube with a stirring bar was added with [Ir(cod)Cl]₂ (1.7 mg, 0.005 mmol, 0.05 equiv) and **L3** (3.6 mg, 0.011 mmol, 0.11 equiv) in 1.0 ml toluene under Ar. The formed mixture was stirred at room temperature for 1h. Then **1a** (97.0 mg, 0.25 mmol), **d-1a** (98.8 mg, 0.25 mmol) and 3-hexyne **2b** (8.2 mg, 0.1 mmol) was added under argon, the resulting mixture was stirred at 75 °C. After 72 h, the solvent was removed under vaccum. The crude product purified by column chromatography on silica gel (PE/DCM = 10/1) to afford the corresponding product **3ab/d-3ab** mixture. The ratio of the two products was determined by the integration of signals in ^{1}H NMR resulting in a KIE-value of 2.03.

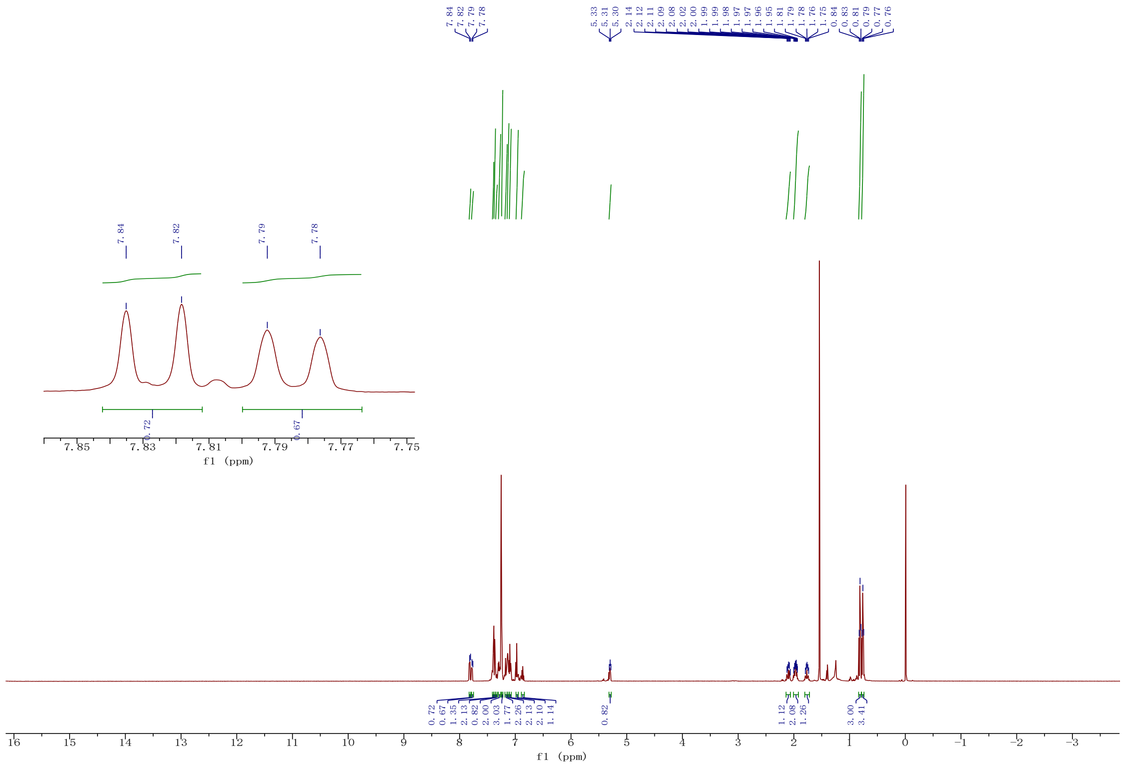

Supplementary Fig. 3. ^{1}H NMR spectra (400 MHz, $CDCl_3$, 25 °C) of **3ab/d-3ab**

2.4.5 Nonlinear Effects

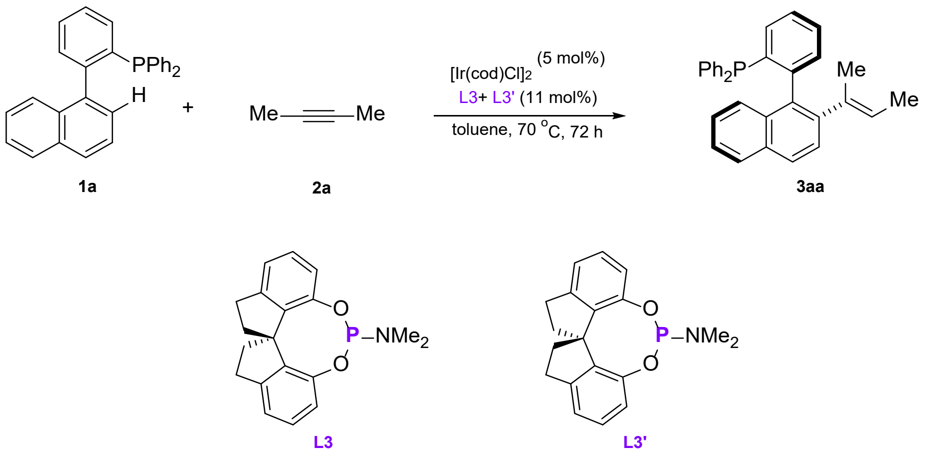

General Procedure **A**, a 25.0 mL dry Schlenk tube with a stirring bar was added with $[Ir(cod)Cl]_2$ (3.4 mg, 0.01 mmol, 0.05 equiv) and **L3** + **L3'** (7.3 mg, 0.022 mmol, 0.11 equiv) in 2.0 ml toluene under Ar. The formed mixture was stirred at room temperature for 1h. Then **1a** (77.6 mg, 0.2 mmol) and **2a** (2-butyne, 54.0 mg, 1.0

mmol) was added under argon, the resulting mixture was stirred at 70 °C. After 72 h, the solvent was removed under vaccum. The crude product purified by column chromatography on silica gel (PE/DCM = 10/1) to afford the corresponding product **3aa** as a white solid. The enantiomeric excess of **3aa** was determined by chiral HPLC analysis.

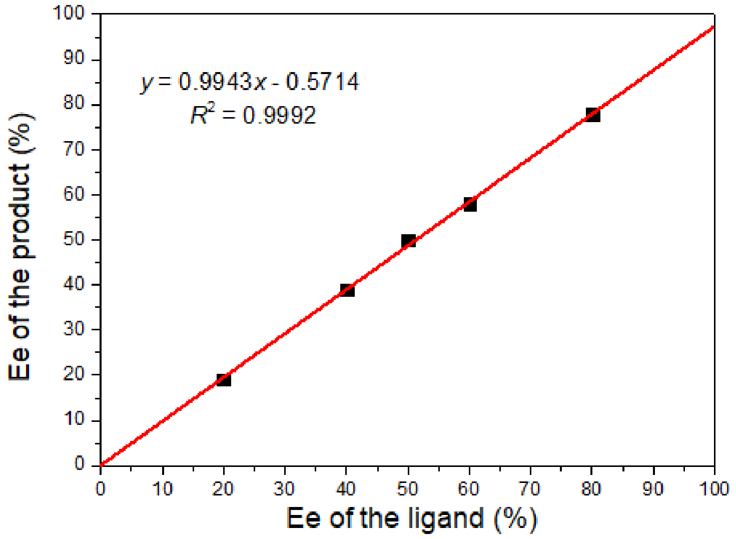

Supplementary Fig. 4. Nonlinear effect in the Ir/**L3**-catalyzed Alkyne hydroaromatic reaction of **1a**.

A linear effect was observed, indicating that the monomeric iridium complex with **L3** as the chiral ligand is the possible active species in asymmetric reaction.

2.4.6 Computational details

All DFT calculations were performed using the Gaussian 09 package[11]. Geometry of all the intermediates and transition structures were optimized at the B3LYP-D3BJ[12] level of theory with a mixed basis set of LANL2DZ[13] for Ir and 6-31G(d)[14] for other atoms in toluene solvent with the CPCM[15] solvation model. Vibrational frequencies were calculated at the same level to obtain the thermal correction to free energies and to confirm that the optimized structures are either local minimums or transition states. The keywords int=ultrafine (defining the integration grids), freq=noraman (skipping the extra steps required to compute the Raman

intensities), and iop(1/8=N) (setting the maximum size for an optimization step to 0.01N Bohr or radians) were used for all calculations. Solvation single-point energy were calculated in toluene with CPCM solvation model based on the above optimized geometries at the ωb97xd/6-311+G(d,p)[16]-SDD[17] level of theory with keyword. We added detailed input keywords and output information for all of the structures to improve reproducibility and accessibility. The frontier molecular orbitals analysis of some key intermediates was performed using HF/6-31G(d)-LANL2DZ method. All energies are reported here in kcal/mol and the calculated optimized structures are illustrated by CYLview.[18]

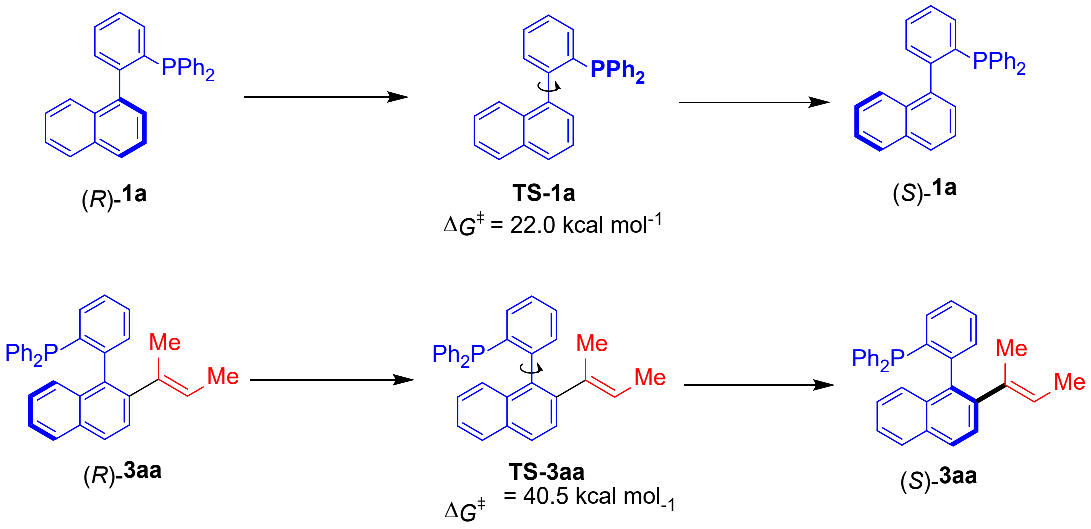

Supplementary Fig. 5. The energy barriers for the interconversion of two enantiomers.

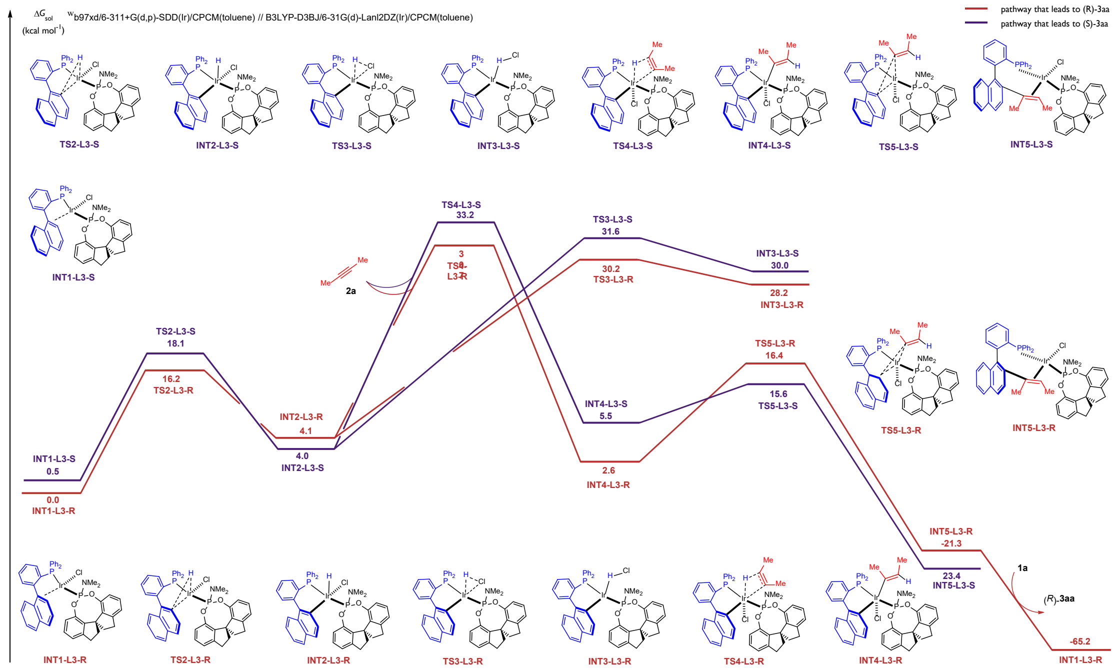

Supplementary Fig. 6. DFT-calculated energy profile for the enantioselective hydroarylation of 1a and alkyne 2a under the catalysis of Ir and chiral phosphine L3.

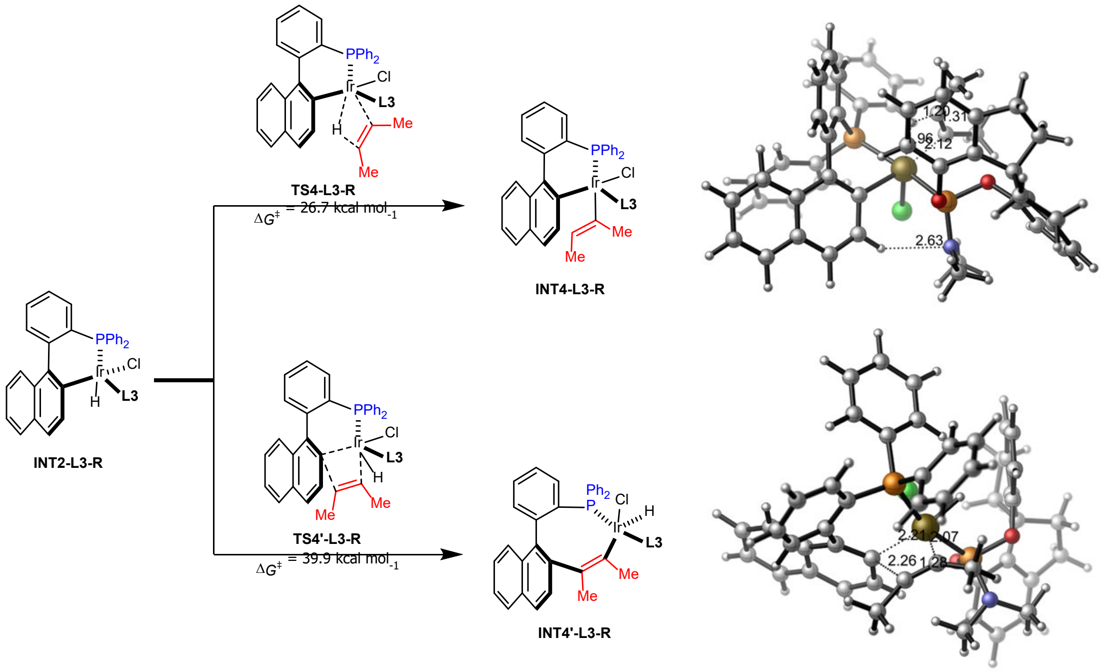

Supplementary Fig. 7. DFT-calculated free energies for the two competitive pathways of alkyne insertion into Ir–H and Ir–C bond.

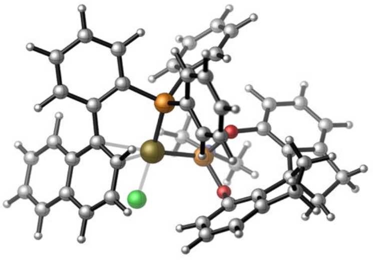

INT1-L3-R

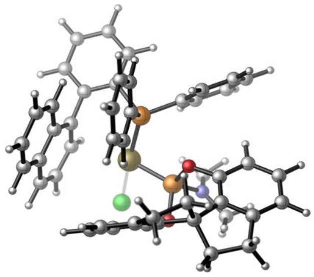

INT1-L3-S

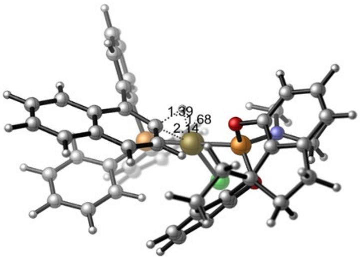

TS2-L3-R

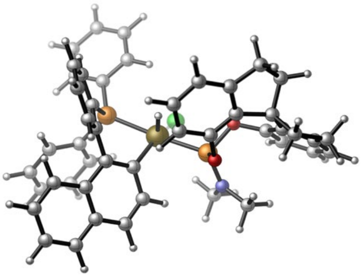

INT2-L3-R

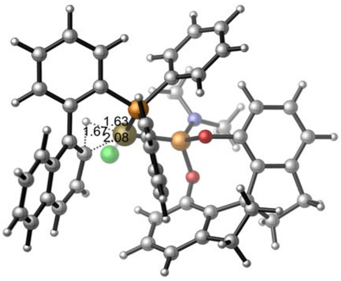

TS2-L3-S

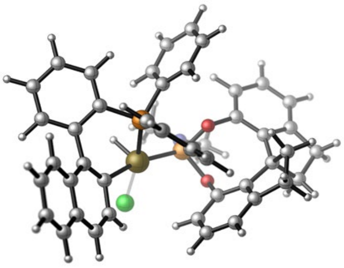

INT2-L3-S

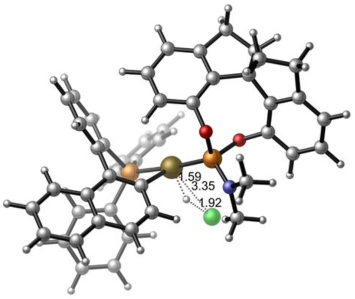

TS3-L3-R

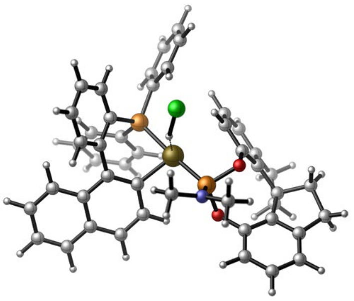

INT3-L3-R

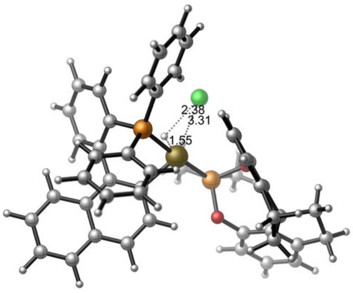

TS3-L3-S

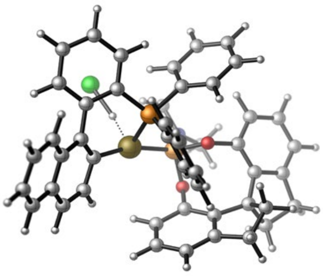

INT3-L3-S

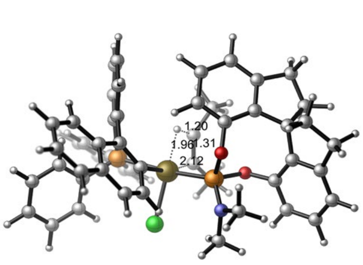

TS4-L3-R

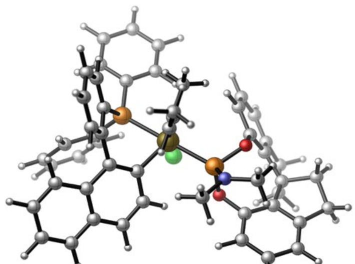

INT4-L3-R

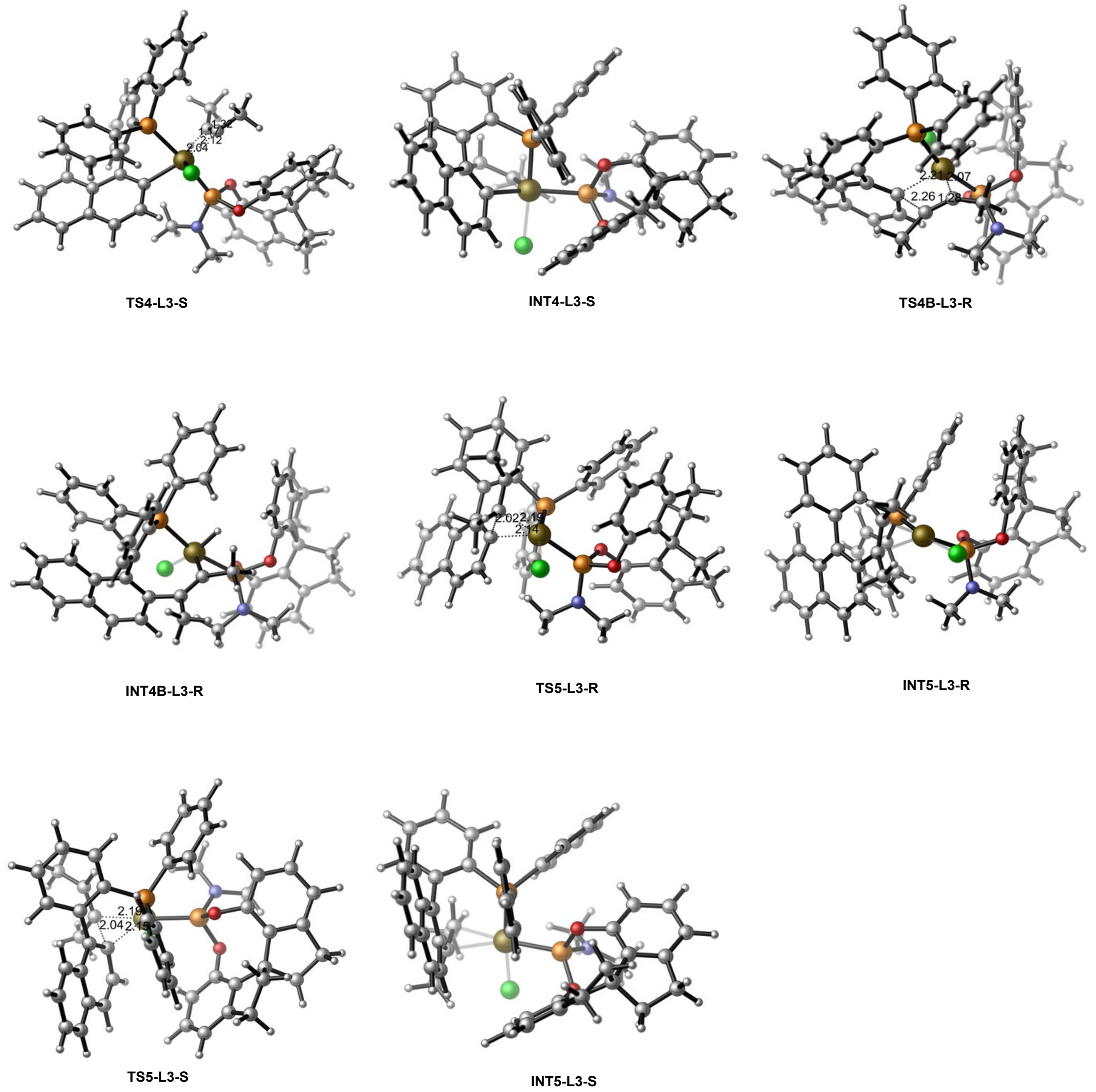

Supplementary Fig. 8. The calculated optimized structures of the key intermediates and transition states. Distances are in Å.

Supplementary Table 7. The calculated energies of stationary points (in Hartree/Particle) and vibrational frequencies of transitions states (in cm^{-1}).

Structure	E_{ele}[1]	E_0[2]	E[3]	H[4]	G[5]
**INT1-L3-R**	-3268.281437	-3267.517666	-3267.470921	-3267.469976	-3267.596791
**INT1-L3-S**	-3268.281758	-3267.517578	-3267.470944	-3267.469999	-3267.595937
**TS2-L3-R**	-3268.248752	-3267.490143	-3267.443395	-3267.442451	-3267.570958
**INT2-L3-R**	-3268.268841	-3267.508132	-3267.461087	-3267.460143	-3267.590293
**TS2-L3-S**	-3268.247977	-3267.48861	-3267.442125	-3267.441181	-3267.567881

**INT2-L3-S**	-3268.271031	-3267.509994	-3267.463189	-3267.462245	-3267.590389
**2a**	-155.9643993	-155.8797953	-155.8740553	-155.8731103	-155.9067703
**TS3-L3-R**	-3268.224113	-3267.465145	-3267.418485	-3267.417541	-3267.54874
**INT3-L3-R**	-3268.22749	-3267.468636	-3267.421171	-3267.420227	-3267.551923
**TS3-L3-S**	-3268.224022	-3267.464773	-3267.417921	-3267.416976	-3267.546443
**INT3-L3-S**	-3268.227128	-3267.467265	-3267.420633	-3267.419689	-3267.546752
**TS4-L3-R**	-3424.216769	-3423.368508	-3423.316371	-3423.315427	-3423.454581
**INT4-L3-R**	-3424.266823	-3423.414847	-3423.363258	-3423.362314	-3423.499433
**TS4-L3-S**	-3424.21351	-3423.364975	-3423.312989	-3423.312045	-3423.45062
**INT4-L3-S**	-3424.265716	-3423.412359	-3423.36125	-3423.360306	-3423.494823
**TS4B-L3-R**	-3424.195976	-3423.348194	-3423.296431	-3423.295487	-3423.433488
**INT4B-L3-R**	-3424.282504	-3423.431258	-3423.379261	-3423.378317	-3423.517374
**TS5-L3-R**	-3424.244645	-3423.393463	-3423.34168	-3423.340736	-3423.477493
**INT5-L3-R**	-3424.310609	-3423.455553	-3423.404333	-3423.403389	-3423.537556
**TS5-L3-S**	-3424.246827	-3423.395393	-3423.343651	-3423.342707	-3423.47876
**INT5-L3-S**	-3424.314939	-3423.459551	-3423.408439	-3423.407495	-3423.540851
**1a**	-1421.423595	-1421.020788	-1420.997666	-1420.996722	-1421.076407
(*R*)-**3aa**	-1577.517965	-1577.026075	-1576.997336	-1576.996392	-1577.087138
(*S*)-**3aa**	-1577.516719	-1577.024461	-1576.995947	-1576.995003	-1577.084856

Note: [1]The electronic energies calculated by ωb97xd in toluene solvent. [2]The sum of electronic and zero-point energies in toluene solvent. [3]The sum of electronic and thermal energies in toluene solvent. [4]The sum of electronic and thermal ethalpies in toluene solvent. [5]The sum of electronic and thermal free energies in toluene solvent.

Furthermore, three DFT functionals (B3LYP-D3BJ, M06, and ωb97x-D) with SDD for Ir and 6-311+G(d,p) for other atoms was further used to compute the solvation single-point energies in toluene with CPCM continuum model. All of the energies are calculated as the sum of the solution-phase free energy and the corresponding thermal correction obtained in gas phase. The absolute (in Hartree) single-point energies and

relative (in kcal mol^{-1}) Gibbs free energies in toluene with different DFT functionals were shown as follows:

structure	G_{corr}	$E_{B3LYP-D3BJ}$	$\Delta G_{B3LYP-D3BJ}$	E_{M06}	ΔG_{M06}	$E_{\omega b97xd}$	$\Delta G_{\omega b97xd}$
**INT1A-R**	0.684646	-3269.263476	0.0	-3267.321030	0.0	-3268.281437	0.0
**INT1A-S**	0.685821	-3269.264195	-0.1	-3267.320236	0.8	-3268.281758	0.5
**TS2A-R**	0.677794	-3269.231246	15.9	-3267.288953	15.8	-3268.248752	16.2
**INT2A-R**	0.678548	-3269.252669	3.0	-3267.300199	9.2	-3268.268841	4.1
**TS2A-S**	0.680096	-3269.232837	16.0	-3267.285379	19.1	-3268.247977	18.1
**INT2A-S**	0.680642	-3269.256038	1.8	-3267.301772	9.2	-3268.271031	4.0
**TS3A-R**	0.675373	-3269.212478	26.2	-3267.257176	34.3	-3268.224113	30.2
**INT3A-R**	0.675567	-3269.216703	23.7	-3267.262091	31.3	-3268.227490	28.2
**TS3A-S**	0.677579	-3269.212429	27.2	-3267.257480	35.0	-3268.224022	31.6
**INT3A-S**	0.680376	-3269.217688	25.7	-3267.261057	34.6	-3268.227128	31.4
**TS4A-R**	0.762188	-3425.273030	30.1	-3423.172342	38.0	-3424.216769	30.7
**INT4A-R**	0.767390	-3425.325659	0.3	-3423.220487	11.0	-3424.266823	2.6
**TS4A-S**	0.762890	-3425.270138	32.0	-3423.168350	40.5	-3424.213510	33.2
**INT4A-S**	0.770893	-3425.326087	1.9	-3423.219206	13.6	-3424.265716	5.5
**TS5A-R**	0.767152	-3425.303832	13.9	-3423.205327	20.4	-3424.244645	16.4
**INT5A-R**	0.773053	-3425.364097	-20.2	-3423.266876	-14.5	-3424.310609	-21.3
**TS5A-S**	0.768067	-3425.305750	12.9	-3423.206947	19.6	-3424.246827	15.6
**INT5A-S**	0.774088	-3425.367967	-22.4	-3423.270868	-16.8	-3424.314939	-23.4

The calculated results indicated the solvation single-point energies of key transition states using ωb97x-D functional is well agreement with the experimental observed enantioselectivity. We have added this table in the revised Supplementary Information.

2.5 Crystallographic Data

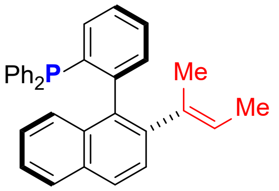

3aa

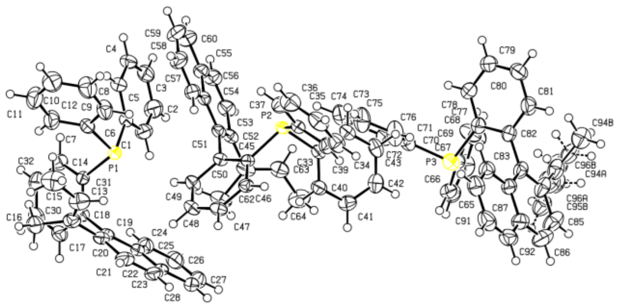

CCDC 2169735

Supplementary Table 8. Crystal data and structure refinement for B.

Identification code	B
Empirical formula	$C_{32}H_{27}P$
Formula weight	442.50
Temperature/K	189.99
Crystal system	orthorhombic
Space group	$P2_12_12_1$
a/Å	10.3923(6)
b/Å	12.6490(7)
c/Å	55.632(3)
α/°	90
β/°	90
γ/°	90
Volume/Å^3	7313.0(7)
Z	12
ρ_{calc}g/cm^3	1.206
μ/mm^{-1}	0.720
F(000)	2808.0
Crystal size/mm^3	0.12 × 0.1 × 0.1
Radiation	GaKα (λ = 1.34139)
2Θ range for data collection/°	5.528 to 121.448
Index ranges	$-13 \leq h \leq 13$, $-8 \leq k \leq 16$, $-71 \leq l \leq 67$
Reflections collected	56089
Independent reflections	16539 [R_{int} = 0.0414, R_{sigma} = 0.0465]
Data/restraints/parameters	16539/88/937

Goodness-of-fit on F^2	1.030
Final R indexes [I>=2σ (I)]	R_1 = 0.0439, wR_2 = 0.1065
Final R indexes [all data]	R_1 = 0.0597, wR_2 = 0.1164
Largest diff. peak/hole / e Å^{-3}	0.78/-0.56
Flack parameter	0.016(7)

Supplementary Table 9. Fractional Atomic Coordinates (×10^4) and Equivalent Isotropic Displacement Parameters (Å^2×10^3) for B. U_{eq} is defined as 1/3 of of the trace of the orthogonalised U_{IJ} tensor.

Atom	*x*	*y*	*z*	U(eq)
P2	6703.3(7)	6213.9(5)	4241.8(2)	28.50(15)
P1	2885.2(7)	1238.6(5)	4158.0(2)	32.85(16)
P3	7838.7(8)	8075.0(6)	2625.7(2)	39.18(19)
C48	7446(3)	2723(2)	4452.1(5)	35.8(7)
C38	7680(3)	6916.9(19)	4465.4(5)	30.0(6)
C39	7676(3)	6411.6(18)	3972.6(5)	28.7(6)
C33	8918(3)	7299(2)	4425.7(6)	38.2(7)
C49	6347(3)	3007(2)	4327.2(5)	34.9(6)
C14	1383(3)	-539(2)	4022.6(6)	38.6(7)
C50	6133(3)	4054.7(19)	4260.1(5)	27.9(6)
C18	3675(3)	-813(2)	4028.4(5)	29.1(6)
C51	4879(3)	4340.2(19)	4142.7(5)	29.9(6)
C45	7045(3)	4827.4(19)	4321.2(5)	26.7(5)
C13	2615(3)	-146(2)	4067.9(5)	29.9(6)
C5	554(3)	2374(2)	4060.8(6)	40.1(7)
C46	8142(3)	4524(2)	4448.4(5)	30.7(6)
C56	3863(3)	4700(2)	4297.6(5)	32.3(6)
C52	4676(3)	4258(2)	3898.8(5)	34.0(6)
C78	6350(3)	9894(3)	2740.3(5)	38.7(7)
C40	8591(3)	5712(2)	3882.7(5)	36.8(7)
C44	7479(3)	7358(2)	3847.3(5)	35.0(6)
C70	7175(3)	7886(2)	2323.9(5)	37.3(7)
C19	5037(3)	-453.3(19)	4062.5(5)	29.6(6)
C20	5595(3)	-396(2)	4286.8(5)	33.3(6)
C61	5656(3)	3890(2)	3722.1(5)	37.4(7)
C17	3459(3)	-1848(2)	3950.2(6)	41.0(7)
C7	2118(3)	1337(2)	4455.8(5)	35.9(6)

Supplementary Table 9. Fractional Atomic Coordinates (×10[4]) and Equivalent Isotropic Displacement Parameters (Å[2]×10[3]) for B. U_{eq} is defined as 1/3 of of the trace of the orthogonalised U_{IJ} tensor.

Atom	*x*	*y*	*z*	U(eq)
C47	8344(3)	3486(2)	4513.7(5)	34.7(6)
C77	7564(3)	9491(2)	2686.6(5)	34.0(6)
C37	7117(4)	7098(2)	4689.8(6)	42.5(7)
C55	2647(3)	4958(2)	4196.4(6)	40.4(7)
C79	6176(4)	10949(3)	2799.2(6)	43.8(8)
C63	6110(3)	4571(3)	3560.4(6)	43.1(8)
C29	4878(3)	-594(3)	4513.2(6)	40.3(7)
C83	9968(3)	9802(2)	2642.8(6)	38.5(7)
C24	5750(3)	-175(2)	3853.5(5)	33.3(6)
C30	4126(4)	-1603(3)	4542.4(6)	50.5(9)
C4	-332(3)	2830(2)	3905.9(7)	45.6(8)
C82	8625(3)	10169(2)	2690.7(5)	35.4(6)
C42	9058(3)	6878(3)	3554.8(6)	42.0(7)
C15	1189(3)	-1573(3)	3948.2(6)	44.2(8)
C41	9266(3)	5945(3)	3674.9(6)	42.2(8)
C16	2225(4)	-2224(2)	3911.7(6)	45.9(8)
C88	10728(3)	9465(2)	2843.6(6)	38.0(7)
C53	3443(3)	4511(2)	3804.0(6)	42.6(7)
C89	10258(3)	9452(2)	3079.7(6)	43.1(8)
C87	12020(3)	9121(2)	2802.4(7)	46.8(8)
C57	4024(3)	4812(2)	4548.1(6)	39.2(7)
C76	5569(3)	6881(3)	2723.5(6)	45.8(8)
C35	9010(4)	7992(2)	4827.1(6)	49.5(9)
C80	7225(4)	11614(3)	2806.7(6)	48.7(8)
C25	5193(4)	-181(2)	3619.2(6)	41.5(7)
C43	8164(3)	7587(2)	3643.2(6)	41.5(8)
C65	7563(3)	6986(2)	2201.6(6)	42.3(7)
C74	4772(4)	6543(3)	3120.5(7)	52.2(9)
C54	2469(3)	4840(3)	3948.4(7)	47.6(8)
C23	7054(3)	141(2)	3875.0(6)	40.1(7)
C3	-154(4)	2783(3)	3663.4(7)	51.6(9)
C6	1637(3)	1859(2)	3970.8(6)	35.2(6)
C69	6311(4)	8557(3)	2210.7(6)	53.8(9)
C71	6568(3)	7491(2)	2811.7(6)	39.3(7)

Supplementary Table 9. Fractional Atomic Coordinates (×10^4) and Equivalent Isotropic Displacement Parameters (Å^2×10^3) for B. U_{eq} is defined as 1/3 of of the trace of the orthogonalised U_{IJ} tensor.

**Atom**	***x***	***y***	***z***	**U(eq)**
C21	6919(3)	-110(2)	4302.5(6)	42.9(8)
C58	3036(4)	5167(3)	4690.5(7)	51.0(9)
C34	9573(4)	7831(2)	4605.4(7)	47.5(8)
C36	7777(4)	7627(3)	4868.4(6)	52.6(9)
C75	4681(4)	6416(3)	2877.0(7)	53.6(9)
C81	8428(4)	11234(3)	2753.4(6)	47.7(8)
C22	7611(3)	161(2)	4105.3(7)	47.2(8)
C90	10994(4)	9129(3)	3269.4(7)	52.4(9)
C84	10471(4)	9798(3)	2412.8(6)	48.7(8)
C12	1265(4)	625(3)	4556.4(7)	52.2(9)
C62	5977(4)	2729(3)	3726.6(7)	54.5(9)
C2	922(4)	2275(3)	3570.0(7)	56.9(10)
C1	1796(4)	1828(3)	3722.7(6)	49.4(9)
C91	12281(4)	8785(3)	3229.1(8)	59.4(10)
C60	1660(3)	5330(3)	4347.2(8)	55.5(9)
C66	7119(4)	6760(3)	1972.9(6)	48.3(8)
C8	2415(4)	2239(2)	4588.3(6)	49.5(9)
C72	6639(4)	7611(3)	3059.9(6)	58.6(10)
C68	5871(5)	8336(3)	1980.7(7)	65.8(12)
C26	5890(4)	100(3)	3422.1(7)	54.9(10)
C9	1842(4)	2430(3)	4808.4(7)	60.1(11)
C31	4928(4)	142(3)	4685.2(6)	51.9(9)
C73	5755(4)	7159(3)	3212.7(7)	61.9(11)
C64	7014(4)	4350(3)	3356.8(6)	54.8(9)
C92	12764(4)	8785(3)	3001.2(8)	56.8(9)
C10	997(4)	1717(3)	4904.9(7)	62.1(11)
C11	713(5)	818(3)	4778.1(7)	65.8(12)
C27	7179(5)	409(3)	3443.1(8)	60.3(11)
C93B	9675(6)	10193(5)	2200.6(10)	49.8(11)
C59	1843(4)	5434(3)	4587.4(8)	59.7(10)
C28	7755(4)	429(2)	3664.3(8)	56.0(10)
C86	12498(4)	9135(3)	2565.7(8)	60.8(11)
C67	6286(4)	7444(3)	1862.2(6)	55.8(10)
C32	4255(5)	112(4)	4924.0(6)	70.6(13)

Supplementary Table 9. Fractional Atomic Coordinates (×10[4]) and Equivalent Isotropic Displacement Parameters (Å^2×10^3) for B. U_{eq} is defined as 1/3 of of the trace of the orthogonalised U_{IJ} tensor.

**Atom**	*x*	*y*	*z*	**U(eq)**
C85	11758(4)	9461(3)	2380.5(8)	61.5(11)
C94B	9289(6)	11365(5)	2181.3(10)	57.0(13)
C95B	9344(6)	9531(5)	2027.0(8)	50.9(11)
C96B	8511(7)	9820(5)	1800.0(10)	61.0(13)
C94A	9402(15)	10756(15)	2151(3)	53(2)
C93A	9938(17)	9807(13)	2183(3)	54(2)
C95A	9979(16)	8979(13)	2017(2)	55(2)
C96A	9075(18)	9480(14)	1828(3)	61(3)

Supplementary Table 10. Anisotropic Displacement Parameters (Å^2×10^3) for B. The Anisotropic displacement factor exponent takes the form: $-2\pi^2[h^2a^{*2}U_{11}+2hka^*b^*U_{12}+\ldots]$.

**Atom**	**U_{11}**	**U_{22}**	**U_{33}**	**U_{23}**	**U_{13}**	**U_{12}**
P2	26.1(3)	23.2(3)	36.2(4)	-1.4(3)	-1.0(3)	2.4(3)
P1	28.0(4)	26.6(3)	44.0(4)	3.2(3)	2.9(3)	1.0(3)
P3	35.7(4)	36.7(4)	45.2(4)	-0.7(3)	-9.5(4)	-1.7(3)
C48	43.6(18)	25.9(12)	38.0(15)	2.8(11)	-3.7(14)	7.2(12)
C38	32.1(15)	23.5(11)	34.6(14)	-0.4(10)	0.9(12)	2.8(11)
C39	29.8(15)	23.5(11)	32.7(14)	0.2(10)	-6.1(12)	-0.1(10)
C33	38.0(17)	33.8(14)	42.7(17)	-3.2(12)	0.8(14)	-6.5(13)
C49	38.7(17)	26.4(13)	39.7(16)	0.2(11)	-6.4(13)	0.2(12)
C14	30.0(16)	40.7(15)	45.3(18)	2.9(13)	-4.1(14)	-2.8(13)
C50	28.0(14)	25.9(12)	29.7(14)	-1.6(10)	-2.5(12)	3.2(10)
C18	28.1(15)	27.4(12)	31.8(14)	4.3(10)	0.6(12)	0.9(10)
C51	28.6(14)	21.3(11)	39.7(15)	-1.5(10)	-4.6(12)	0.1(10)
C45	25.9(13)	25.7(11)	28.4(13)	-1.4(10)	1.5(11)	3.3(10)
C13	25.9(14)	29.8(12)	34.0(14)	4.3(11)	1.4(12)	-1.1(11)
C5	37.9(17)	32.9(14)	49.6(18)	1.9(13)	4.3(15)	7.2(13)
C46	26.7(15)	31.2(12)	34.2(15)	-2.4(11)	-2.8(12)	1.2(11)
C56	28.1(15)	23.3(12)	45.4(17)	1.4(11)	-0.4(13)	-2.1(10)
C52	36.5(17)	23.6(12)	41.7(16)	-2.9(11)	-9.2(13)	2.0(11)
C78	33.6(17)	47.8(16)	34.7(15)	5.1(13)	-0.6(13)	0.2(13)
C40	40.5(18)	33.3(14)	36.6(16)	2.6(12)	2.3(14)	6.8(12)
C44	38.2(17)	27.2(12)	39.4(16)	-0.1(11)	-8.8(14)	2.1(12)

Supplementary Table 10. Anisotropic Displacement Parameters (Å^2×10^3) for B. The Anisotropic displacement factor exponent takes the form: $-2\pi^2[h^2a^{*2}U_{11}+2hka^*b^*U_{12}+\ldots]$.

Atom	U_{11}	U_{22}	U_{33}	U_{23}	U_{13}	U_{12}
C70	36.3(16)	35.7(14)	39.9(16)	-0.9(12)	2.7(14)	-2.3(13)
C19	29.7(15)	21.7(11)	37.4(15)	-0.8(10)	0.3(12)	6.2(10)
C20	29.3(15)	31.3(13)	39.1(16)	-2.9(11)	-2.6(13)	7.4(11)
C61	39.5(17)	35.3(14)	37.4(16)	-7.2(12)	-14.0(14)	7.0(13)
C17	41.9(18)	30.9(14)	50.0(19)	-3.5(13)	0.0(15)	-1.3(13)
C7	36.1(16)	28.6(13)	43.0(16)	2.9(11)	-1.2(14)	2.0(12)
C47	35.8(16)	35.7(14)	32.4(15)	-1.9(11)	-7.7(13)	9.1(12)
C77	35.4(17)	37.6(14)	29.1(14)	3.5(11)	-3.4(12)	-2.5(12)
C37	43.5(19)	44.4(16)	39.6(17)	-6.5(13)	2.1(15)	-0.1(14)
C55	27.0(16)	32.0(13)	62(2)	-0.3(13)	-2.0(15)	-0.6(12)
C79	46(2)	48.8(17)	36.8(17)	6.6(13)	3.8(15)	8.0(15)
C63	48(2)	43.0(16)	38.5(17)	-2.2(13)	-7.0(15)	7.3(15)
C29	35.8(18)	47.1(17)	38.2(17)	6.0(13)	-3.7(14)	11.0(14)
C83	36.7(17)	38.3(15)	40.6(17)	-5.9(13)	6.3(14)	-10.6(13)
C24	37.3(17)	21.9(11)	40.5(16)	-0.2(11)	9.3(13)	6.6(11)
C30	56(2)	49.8(18)	46.0(19)	12.9(15)	6.3(18)	7.9(17)
C4	35.3(18)	36.0(15)	66(2)	5.1(15)	0.5(16)	9.8(13)
C82	36.5(17)	39.2(14)	30.5(14)	1.7(12)	0.6(12)	-3.6(12)
C42	49(2)	46.8(16)	30.4(15)	2.1(13)	-2.5(14)	-10.2(15)
C15	39.1(18)	47.7(17)	45.9(19)	1.6(14)	-4.4(15)	-13.4(15)
C41	41.2(19)	47.3(17)	38.1(17)	-4.3(13)	3.2(15)	5.4(14)
C16	54(2)	34.3(14)	49.3(19)	-4.5(13)	-5.8(17)	-11.1(15)
C88	34.0(17)	34.0(14)	46.0(18)	-4.7(12)	2.3(14)	-5.5(12)
C53	38.4(18)	39.7(15)	49.7(19)	0.4(13)	-14.0(16)	1.7(14)
C89	40.5(19)	43.2(16)	45.5(19)	1.8(14)	-1.5(15)	-3.4(14)
C87	35.1(18)	39.5(15)	66(2)	-9.7(15)	0.6(17)	-3.2(13)
C57	35.0(17)	32.5(14)	50.1(18)	-5.8(13)	3.0(15)	-4.0(12)
C76	48(2)	45.5(17)	43.5(18)	-9.4(14)	-2.6(16)	-6.9(15)
C35	65(2)	37.7(16)	46.4(19)	-8.6(14)	-19.0(18)	0.6(16)
C80	58(2)	37.9(15)	50(2)	1.3(14)	3.6(18)	7.1(16)
C25	54(2)	33.1(14)	37.8(17)	0.2(12)	8.5(15)	4.5(14)
C43	55(2)	33.3(14)	36.4(16)	8.1(12)	-8.4(15)	-4.0(14)
C65	38.1(18)	41.2(15)	47.8(18)	-2.7(13)	8.3(15)	2.4(13)
C74	56(2)	42.4(17)	58(2)	15.5(15)	-2.2(18)	-9.4(16)
C54	34.1(18)	45.1(16)	63(2)	1.3(15)	-14.5(16)	3.4(14)

Supplementary Table 10. Anisotropic Displacement Parameters (Å^2×10^3) for B. The Anisotropic displacement factor exponent takes the form: $-2\pi^2[h^2a^{*2}U_{11}+2hka^*b^*U_{12}+\ldots]$.

Atom	U_{11}	U_{22}	U_{33}	U_{23}	U_{13}	U_{12}
C23	34.3(17)	24.5(12)	61(2)	-4.3(12)	15.3(15)	4.6(12)
C3	45(2)	47.8(18)	62(2)	21.3(16)	-3.2(18)	8.4(16)
C6	32.8(15)	26.9(12)	46.0(17)	7.4(11)	5.8(14)	4.2(12)
C69	70(3)	44.7(18)	47(2)	-3.0(15)	-13.4(18)	13.8(17)
C71	42.1(18)	33.6(14)	42.3(17)	4.6(12)	-12.4(15)	-2.6(13)
C21	32.0(17)	42.4(16)	54(2)	-8.0(14)	-6.5(15)	7.0(13)
C58	50(2)	48.6(18)	54(2)	-8.5(15)	13.7(18)	-4.2(16)
C34	50(2)	37.0(15)	56(2)	-1.4(14)	-12.8(17)	-8.2(15)
C36	69(3)	51.9(18)	36.4(17)	-10.0(14)	0.3(17)	6.5(18)
C75	54(2)	41.3(18)	65(2)	-4.0(16)	-0.4(19)	-13.7(16)
C81	52(2)	38.6(15)	53(2)	-0.6(14)	6.7(17)	-9.0(16)
C22	26.4(16)	38.6(15)	77(2)	-7.9(15)	4.1(17)	4.1(13)
C90	58(2)	50.0(18)	49(2)	8.0(15)	-7.3(18)	-5.6(17)
C84	45(2)	57.7(18)	43.5(16)	-8.6(14)	8.2(14)	-19.7(15)
C12	61(2)	40.0(16)	56(2)	-3.1(15)	13.5(19)	-9.8(16)
C62	67(3)	38.6(16)	58(2)	-9.1(15)	-5.0(19)	16.4(17)
C2	57(2)	67(2)	47(2)	19.9(18)	8.1(18)	11.9(19)
C1	45(2)	55.0(19)	47.9(19)	15.1(16)	12.1(16)	14.6(16)
C91	52(2)	47.6(18)	79(3)	14.1(19)	-20(2)	-2.1(18)
C60	31.1(18)	56(2)	80(3)	5.8(19)	5.3(19)	4.1(16)
C66	56(2)	43.7(16)	45.1(18)	-8.5(14)	13.0(17)	-5.8(16)
C8	64(2)	37.8(15)	46.3(19)	-2.3(13)	3.5(17)	-10.1(16)
C72	66(3)	65(2)	45(2)	15.1(17)	-21.3(19)	-26(2)
C68	87(3)	62(2)	48(2)	2.3(17)	-23(2)	14(2)
C26	78(3)	43.0(17)	43.2(19)	6.1(14)	19.2(19)	12.3(18)
C9	84(3)	46.6(18)	50(2)	-7.4(16)	6(2)	-8.2(19)
C31	52(2)	64(2)	39.3(18)	-0.9(16)	-5.3(16)	12.3(18)
C73	71(3)	69(2)	45(2)	23.0(18)	-15.3(19)	-18(2)
C64	53(2)	71(2)	40.6(19)	-7.1(16)	-2.1(17)	8.4(19)
C92	40(2)	43.0(17)	88(3)	2.1(19)	-3(2)	1.9(17)
C10	80(3)	59(2)	47(2)	-4.9(17)	15(2)	1(2)
C11	80(3)	58(2)	59(2)	-1.5(18)	27(2)	-16(2)
C27	79(3)	40.0(17)	62(2)	11.6(16)	38(2)	11.6(18)
C93B	54(2)	56(2)	39.3(16)	-4.7(15)	7.5(15)	-14.8(18)
C59	39(2)	64(2)	76(3)	-6(2)	24(2)	0.4(17)

Supplementary Table 10. Anisotropic Displacement Parameters (Å^2×10^3) for B. The Anisotropic displacement factor exponent takes the form: -2π^2[h^{2}a*^{2}U$_{11}$+2hka*b*U$_{12}$+…].

Atom	U_{11}	U_{22}	U_{33}	U_{23}	U_{13}	U_{12}
C28	50(2)	30.4(15)	88(3)	4.9(16)	34(2)	6.3(15)
C86	41(2)	67(2)	74(3)	-22(2)	15(2)	-3.9(18)
C67	72(3)	57(2)	38.6(18)	-4.0(16)	-0.8(18)	-12.6(19)
C32	87(3)	92(3)	33.0(18)	-2.5(19)	-3(2)	28(3)
C85	51(2)	79(3)	54(2)	-22(2)	17(2)	-14(2)
C94B	59(3)	60(3)	52(2)	-8.7(19)	-3(2)	-11(2)
C95B	56(2)	58(2)	38.8(16)	-3.0(15)	6.6(15)	-12.8(18)
C96B	63(3)	72(3)	48(2)	0(2)	0(2)	-7(2)
C94A	49(5)	66(6)	44(4)	-9(3)	11(3)	-11(4)
C93A	51(5)	66(5)	45(3)	-9(3)	8(3)	-10(4)
C95A	52(5)	67(5)	45(3)	-9(3)	8(3)	-9(4)
C96A	58(5)	72(6)	51(3)	-8(4)	4(3)	-8(4)

Supplementary Table 11. Bond Lengths for B.

Atom	Atom	Length/Å	Atom	Atom	Length/Å
P2	C38	1.835(3)	C83	C88	1.433(5)
P2	C39	1.824(3)	C83	C84	1.382(4)
P2	C45	1.843(3)	C24	C25	1.426(4)
P1	C13	1.843(3)	C24	C23	1.417(4)
P1	C7	1.843(3)	C4	C3	1.363(5)
P1	C6	1.839(3)	C82	C81	1.406(4)
P3	C70	1.831(3)	C42	C41	1.373(4)
P3	C77	1.844(3)	C42	C43	1.382(5)
P3	C71	1.833(3)	C15	C16	1.370(5)
C48	C49	1.385(4)	C88	C89	1.402(5)
C48	C47	1.385(4)	C88	C87	1.430(5)
C38	C33	1.392(4)	C53	C54	1.358(5)
C38	C37	1.398(4)	C89	C90	1.366(5)
C39	C40	1.392(4)	C87	C92	1.414(5)
C39	C44	1.400(4)	C87	C86	1.408(5)
C33	C34	1.384(4)	C57	C58	1.373(5)
C49	C50	1.395(4)	C76	C71	1.384(4)
C14	C13	1.397(4)	C76	C75	1.389(5)
C14	C15	1.386(4)	C35	C34	1.380(5)

Supplementary Table 11. Bond Lengths for B.

Atom	Atom	Length/Å	Atom	Atom	Length/Å
C50	C51	1.502(4)	C35	C36	1.381(6)
C50	C45	1.403(4)	C80	C81	1.373(5)
C18	C13	1.405(4)	C25	C26	1.361(5)
C18	C19	1.498(4)	C65	C66	1.384(5)
C18	C17	1.398(4)	C74	C75	1.367(5)
C51	C56	1.437(4)	C74	C73	1.383(5)
C51	C52	1.377(4)	C23	C22	1.406(5)
C45	C46	1.396(4)	C23	C28	1.428(5)
C5	C4	1.387(5)	C3	C2	1.391(5)
C5	C6	1.393(4)	C6	C1	1.391(4)
C46	C47	1.379(4)	C69	C68	1.387(5)
C56	C55	1.421(4)	C71	C72	1.391(5)
C56	C57	1.411(4)	C21	C22	1.356(5)
C52	C61	1.490(4)	C58	C59	1.407(5)
C52	C53	1.422(4)	C90	C91	1.424(6)
C78	C77	1.394(4)	C84	C93B	1.526(7)
C78	C79	1.386(4)	C84	C85	1.415(6)
C40	C41	1.384(4)	C84	C93A	1.391(15)
C44	C43	1.371(4)	C12	C11	1.383(5)
C70	C65	1.386(4)	C2	C1	1.365(5)
C70	C69	1.386(5)	C91	C92	1.364(6)
C19	C20	1.378(4)	C60	C59	1.356(6)
C19	C24	1.423(4)	C66	C67	1.369(5)
C20	C29	1.484(4)	C8	C9	1.382(5)
C20	C21	1.426(4)	C72	C73	1.376(5)
C61	C63	1.332(5)	C68	C67	1.376(5)
C61	C62	1.506(4)	C26	C27	1.401(6)
C17	C16	1.385(5)	C9	C10	1.369(6)
C7	C12	1.383(4)	C31	C32	1.502(5)
C7	C8	1.393(4)	C10	C11	1.370(5)
C77	C82	1.397(4)	C27	C28	1.369(6)
C37	C36	1.380(5)	C93B	C94B	1.539(8)
C55	C54	1.400(5)	C93B	C95B	1.323(7)
C55	C60	1.406(5)	C86	C85	1.351(6)
C79	C80	1.377(5)	C95B	C96B	1.574(8)
C63	C64	1.498(5)	C94A	C93A	1.335(18)

Supplementary Table 11. Bond Lengths for B.

Atom	Atom	Length/Å	Atom	Atom	Length/Å
C29	C30	1.506(5)	C93A	C95A	1.398(17)
C29	C31	1.336(5)	C95A	C96A	1.546(18)
C83	C82	1.495(4)			

Supplementary Table 12. Bond Angles for B.

Atom	Atom	Atom	Angle/°	Atom	Atom	Atom	Angle/°
C38	P2	C45	101.08(12)	C3	C4	C5	120.5(3)
C39	P2	C38	100.58(13)	C77	C82	C83	122.9(3)
C39	P2	C45	102.75(12)	C77	C82	C81	118.5(3)
C7	P1	C13	104.06(12)	C81	C82	C83	118.5(3)
C6	P1	C13	98.27(13)	C41	C42	C43	119.4(3)
C6	P1	C7	100.08(14)	C16	C15	C14	119.8(3)
C70	P3	C77	103.69(13)	C42	C41	C40	120.6(3)
C70	P3	C71	101.17(14)	C15	C16	C17	119.9(3)
C71	P3	C77	100.14(14)	C89	C88	C83	122.8(3)
C49	C48	C47	120.0(2)	C89	C88	C87	118.3(3)
C33	C38	P2	124.9(2)	C87	C88	C83	118.9(3)
C33	C38	C37	118.1(3)	C54	C53	C52	121.4(3)
C37	C38	P2	116.9(2)	C90	C89	C88	122.1(3)
C40	C39	P2	125.9(2)	C92	C87	C88	118.6(3)
C40	C39	C44	117.7(3)	C86	C87	C88	118.5(4)
C44	C39	P2	116.4(2)	C86	C87	C92	122.9(4)
C34	C33	C38	120.6(3)	C58	C57	C56	120.9(3)
C48	C49	C50	120.8(3)	C71	C76	C75	121.1(3)
C15	C14	C13	121.5(3)	C34	C35	C36	119.5(3)
C49	C50	C51	118.9(2)	C81	C80	C79	120.0(3)
C49	C50	C45	119.3(3)	C26	C25	C24	121.2(4)
C45	C50	C51	121.6(2)	C44	C43	C42	120.4(3)
C13	C18	C19	122.6(2)	C66	C65	C70	121.6(3)
C17	C18	C13	119.0(3)	C75	C74	C73	119.0(4)
C17	C18	C19	118.4(3)	C53	C54	C55	121.1(3)
C56	C51	C50	116.9(3)	C24	C23	C28	119.4(3)
C52	C51	C50	122.9(3)	C22	C23	C24	118.4(3)
C52	C51	C56	120.2(3)	C22	C23	C28	122.2(3)
C50	C45	P2	118.3(2)	C4	C3	C2	119.9(3)

Supplementary Table 12. Bond Angles for B.

Atom	Atom	Atom	Angle/°	Atom	Atom	Atom	Angle/°
C46	C45	P2	122.7(2)	C5	C6	P1	124.4(2)
C46	C45	C50	118.9(2)	C1	C6	P1	117.8(2)
C14	C13	P1	121.8(2)	C1	C6	C5	117.8(3)
C14	C13	C18	118.5(3)	C70	C69	C68	120.6(3)
C18	C13	P1	119.6(2)	C76	C71	P3	124.4(3)
C4	C5	C6	120.5(3)	C76	C71	C72	116.9(3)
C47	C46	C45	121.3(3)	C72	C71	P3	118.6(3)
C55	C56	C51	119.2(3)	C22	C21	C20	121.8(3)
C57	C56	C51	122.5(3)	C57	C58	C59	120.2(4)
C57	C56	C55	118.3(3)	C35	C34	C33	120.6(3)
C51	C52	C61	124.7(3)	C37	C36	C35	120.2(3)
C51	C52	C53	119.1(3)	C74	C75	C76	120.9(3)
C53	C52	C61	116.2(3)	C80	C81	C82	121.5(3)
C79	C78	C77	121.4(3)	C21	C22	C23	120.9(3)
C41	C40	C39	120.8(3)	C89	C90	C91	119.7(4)
C43	C44	C39	121.1(3)	C83	C84	C93B	120.7(4)
C65	C70	P3	116.6(2)	C83	C84	C85	118.4(4)
C69	C70	P3	125.5(2)	C83	C84	C93A	134.3(8)
C69	C70	C65	117.9(3)	C85	C84	C93B	120.9(4)
C20	C19	C18	121.9(3)	C93A	C84	C85	105.2(8)
C20	C19	C24	120.5(3)	C11	C12	C7	120.8(3)
C24	C19	C18	117.6(3)	C1	C2	C3	119.6(4)
C19	C20	C29	123.3(3)	C2	C1	C6	121.8(3)
C19	C20	C21	118.3(3)	C92	C91	C90	119.5(4)
C21	C20	C29	118.4(3)	C59	C60	C55	121.2(4)
C52	C61	C62	116.4(3)	C67	C66	C65	119.6(3)
C63	C61	C52	119.1(3)	C9	C8	C7	121.1(3)
C63	C61	C62	124.2(3)	C73	C72	C71	122.2(3)
C16	C17	C18	121.2(3)	C67	C68	C69	120.2(4)
C12	C7	P1	126.7(2)	C25	C26	C27	121.0(4)
C12	C7	C8	117.5(3)	C10	C9	C8	120.6(3)
C8	C7	P1	115.8(2)	C29	C31	C32	126.7(4)
C46	C47	C48	119.7(3)	C72	C73	C74	119.9(4)
C78	C77	P3	122.3(2)	C91	C92	C87	121.7(4)
C78	C77	C82	119.1(3)	C9	C10	C11	118.9(4)
C82	C77	P3	118.5(2)	C10	C11	C12	121.1(4)

Supplementary Table 12. Bond Angles for B.

Atom	Atom	Atom	Angle/°	Atom	Atom	Atom	Angle/°
C36	C37	C38	121.0(3)	C28	C27	C26	119.9(3)
C54	C55	C56	119.0(3)	C84	C93B	C94B	120.7(4)
C54	C55	C60	121.8(3)	C95B	C93B	C84	119.9(5)
C60	C55	C56	119.3(3)	C95B	C93B	C94B	119.4(5)
C80	C79	C78	119.5(3)	C60	C59	C58	120.1(3)
C61	C63	C64	127.8(3)	C27	C28	C23	120.7(4)
C20	C29	C30	119.7(3)	C85	C86	C87	121.1(4)
C31	C29	C20	118.1(3)	C66	C67	C68	120.0(3)
C31	C29	C30	122.2(3)	C86	C85	C84	122.2(4)
C88	C83	C82	117.9(3)	C93B	C95B	C96B	125.6(6)
C84	C83	C82	121.3(3)	C84	C93A	C95A	126.1(14)
C84	C83	C88	120.8(3)	C94A	C93A	C84	107.2(12)
C19	C24	C25	122.3(3)	C94A	C93A	C95A	126.7(14)
C23	C24	C19	119.9(3)	C93A	C95A	C96A	97.2(14)
C23	C24	C25	117.8(3)				

Supplementary Table. 13 Torsion Angles for B.

A	B	C	D	Angle/°	A	B	C	D	Angle/°
P2	C38	C33	C34	179.3(2)	C77	P3	C70	C65	162.0(2)
P2	C38	C37	C36	-179.5(3)	C77	P3	C70	C69	-18.9(3)
P2	C39	C40	C41	179.5(2)	C77	P3	C71	C76	118.8(3)
P2	C39	C44	C43	179.7(2)	C77	P3	C71	C72	-65.1(3)
P2	C45	C46	C47	177.3(2)	C77	C78	C79	C80	-0.5(5)
P1	C7	C12	C11	-177.1(3)	C77	C82	C81	C80	-0.9(5)
P1	C7	C8	C9	176.3(3)	C37	C38	C33	C34	0.8(4)
P1	C6	C1	C2	-179.5(3)	C55	C56	C57	C58	-0.4(4)
P3	C70	C65	C66	179.7(3)	C55	C60	C59	C58	-0.1(6)
P3	C70	C69	C68	179.9(3)	C79	C78	C77	P3	176.2(2)
P3	C77	C82	C83	1.3(4)	C79	C78	C77	C82	-0.5(4)
P3	C77	C82	C81	-175.7(2)	C79	C80	C81	C82	-0.1(5)
P3	C71	C72	C73	-177.1(3)	C29	C20	C21	C22	-174.9(3)
C48	C49	C50	C51	-175.1(3)	C83	C82	C81	C80	-178.0(3)
C48	C49	C50	C45	-0.2(4)	C83	C88	C89	C90	180.0(3)
C38	P2	C39	C40	94.7(3)	C83	C88	C87	C92	-179.7(3)
C38	P2	C39	C44	-84.9(2)	C83	C88	C87	C86	0.5(4)

Supplementary Table. 13 Torsion Angles for B.

A	B	C	D	Angle/°	A	B	C	D	Angle/°
C38	P2	C45	C50	155.2(2)	C83	C84	C93B	C94B	66.3(6)
C38	P2	C45	C46	-21.9(3)	C83	C84	C93B	C95B	-115.8(5)
C38	C33	C34	C35	0.0(5)	C83	C84	C85	C86	0.7(6)
C38	C37	C36	C35	0.3(5)	C83	C84	C93A	C94A	69.1(16)
C39	P2	C38	C33	-9.6(3)	C83	C84	C93A	C95A	-111.8(16)
C39	P2	C38	C37	168.9(2)	C24	C19	C20	C29	175.1(3)
C39	P2	C45	C50	-101.1(2)	C24	C19	C20	C21	-3.5(4)
C39	P2	C45	C46	81.7(2)	C24	C25	C26	C27	-0.2(5)
C39	C40	C41	C42	0.8(5)	C24	C23	C22	C21	-0.2(4)
C39	C44	C43	C42	0.8(5)	C24	C23	C28	C27	0.4(4)
C33	C38	C37	C36	-0.9(4)	C30	C29	C31	C32	-1.5(6)
C49	C48	C47	C46	-0.5(5)	C4	C5	C6	P1	179.3(2)
C49	C50	C51	C56	92.8(3)	C4	C5	C6	C1	0.5(5)
C49	C50	C51	C52	-86.4(3)	C4	C3	C2	C1	-0.2(6)
C49	C50	C45	P2	-177.4(2)	C82	C83	C88	C89	1.0(4)
C49	C50	C45	C46	-0.1(4)	C82	C83	C88	C87	-179.4(3)
C14	C15	C16	C17	0.2(5)	C82	C83	C84	C93B	1.2(5)
C50	C51	C56	C55	-178.6(2)	C82	C83	C84	C85	178.7(3)
C50	C51	C56	C57	1.6(4)	C82	C83	C84	C93A	-20.4(11)
C50	C51	C52	C61	-0.5(4)	C15	C14	C13	P1	178.1(2)
C50	C51	C52	C53	177.8(2)	C15	C14	C13	C18	2.1(5)
C50	C45	C46	C47	0.2(4)	C41	C42	C43	C44	-0.8(5)
C18	C19	C20	C29	-5.3(4)	C88	C83	C82	C77	-90.9(4)
C18	C19	C20	C21	176.1(2)	C88	C83	C82	C81	86.1(4)
C18	C19	C24	C25	3.0(4)	C88	C83	C84	C93B	-177.9(4)
C18	C19	C24	C23	-178.2(2)	C88	C83	C84	C85	-0.4(5)
C18	C17	C16	C15	0.6(5)	C88	C83	C84	C93A	160.5(10)
C51	C50	C45	P2	-2.7(4)	C88	C89	C90	C91	-0.4(5)
C51	C50	C45	C46	174.6(3)	C88	C87	C92	C91	-0.1(5)
C51	C56	C55	C54	0.9(4)	C88	C87	C86	C85	-0.3(5)
C51	C56	C55	C60	-178.8(3)	C53	C52	C61	C63	68.2(4)
C51	C56	C57	C58	179.4(3)	C53	C52	C61	C62	-106.5(3)
C51	C52	C61	C63	-113.4(3)	C89	C88	C87	C92	-0.1(4)
C51	C52	C61	C62	71.8(4)	C89	C88	C87	C86	-179.8(3)
C51	C52	C53	C54	0.6(4)	C89	C90	C91	C92	0.1(5)
C45	P2	C38	C33	95.7(2)	C87	C88	C89	C90	0.4(5)

Supplementary Table. 13 Torsion Angles for B.

A	B	C	D	Angle/˚	A	B	C	D	Angle/˚
C45	P2	C38	C37	-85.7(2)	C87	C86	C85	C84	-0.4(6)
C45	P2	C39	C40	-9.3(3)	C57	C56	C55	C54	-179.3(3)
C45	P2	C39	C44	171.0(2)	C57	C56	C55	C60	1.0(4)
C45	C50	C51	C56	-81.9(3)	C57	C58	C59	C60	0.8(6)
C45	C50	C51	C52	98.9(3)	C76	C71	C72	C73	-0.7(6)
C45	C46	C47	C48	0.1(4)	C25	C24	C23	C22	179.3(3)
C13	P1	C7	C12	-14.9(3)	C25	C24	C23	C28	-0.5(4)
C13	P1	C7	C8	166.8(3)	C25	C26	C27	C28	0.1(5)
C13	P1	C6	C5	114.5(3)	C43	C42	C41	C40	0.0(5)
C13	P1	C6	C1	-66.8(3)	C65	C70	C69	C68	-1.1(6)
C13	C14	C15	C16	-1.7(5)	C65	C66	C67	C68	-1.6(6)
C13	C18	C19	C20	79.7(3)	C54	C55	C60	C59	179.6(3)
C13	C18	C19	C24	-100.6(3)	C23	C24	C25	C26	0.4(4)
C13	C18	C17	C16	-0.1(5)	C3	C2	C1	C6	0.5(6)
C5	C4	C3	C2	0.0(5)	C6	P1	C13	C14	-36.9(3)
C5	C6	C1	C2	-0.6(5)	C6	P1	C13	C18	139.0(2)
C56	C51	C52	C61	-179.7(3)	C6	P1	C7	C12	86.4(3)
C56	C51	C52	C53	-1.4(4)	C6	P1	C7	C8	-91.9(3)
C56	C55	C54	C53	-1.7(5)	C6	C5	C4	C3	-0.1(5)
C56	C55	C60	C59	-0.8(5)	C69	C70	C65	C66	0.6(5)
C56	C57	C58	C59	-0.5(5)	C69	C68	C67	C66	1.2(7)
C52	C51	C56	C55	0.6(4)	C71	P3	C70	C65	-94.5(3)
C52	C51	C56	C57	-179.2(3)	C71	P3	C70	C69	84.5(3)
C52	C61	C63	C64	-175.0(3)	C71	P3	C77	C78	-30.0(3)
C52	C53	C54	C55	0.9(5)	C71	P3	C77	C82	146.6(2)
C78	C77	C82	C83	178.1(3)	C71	C76	C75	C74	-0.2(5)
C78	C77	C82	C81	1.1(4)	C71	C72	C73	C74	1.4(6)
C78	C79	C80	C81	0.8(5)	C21	C20	C29	C30	-128.3(3)
C40	C39	C44	C43	0.0(4)	C21	C20	C29	C31	51.6(4)
C44	C39	C40	C41	-0.8(4)	C34	C35	C36	C37	0.5(5)
C70	P3	C77	C78	74.2(3)	C36	C35	C34	C33	-0.6(5)
C70	P3	C77	C82	-109.1(2)	C75	C76	C71	P3	176.3(3)
C70	P3	C71	C76	12.5(3)	C75	C76	C71	C72	0.1(5)
C70	P3	C71	C72	-171.4(3)	C75	C74	C73	C72	-1.4(6)
C70	C65	C66	C67	0.7(5)	C22	C23	C28	C27	-179.4(3)
C70	C69	C68	C67	0.2(7)	C90	C91	C92	C87	0.1(5)

Supplementary Table. 13 Torsion Angles for B.

A	B	C	D	Angle/°	A	B	C	D	Angle/°
C19	C18	C13	P1	1.5(4)	C84	C83	C82	C77	90.0(4)
C19	C18	C13	C14	177.6(3)	C84	C83	C82	C81	-93.0(4)
C19	C18	C17	C16	-179.0(3)	C84	C83	C88	C89	-179.8(3)
C19	C20	C29	C30	53.1(4)	C84	C83	C88	C87	-0.2(4)
C19	C20	C29	C31	-127.0(3)	C84	C93B	C95B	C96B	179.0(5)
C19	C20	C21	C22	3.8(4)	C84	C93A	C95A	C96A	172.9(15)
C19	C24	C25	C26	179.2(3)	C12	C7	C8	C9	-2.1(5)
C19	C24	C23	C22	0.5(4)	C62	C61	C63	C64	-0.6(6)
C19	C24	C23	C28	-179.4(2)	C60	C55	C54	C53	178.0(3)
C20	C19	C24	C25	-177.3(3)	C8	C7	C12	C11	1.2(6)
C20	C19	C24	C23	1.5(4)	C8	C9	C10	C11	-1.0(7)
C20	C29	C31	C32	178.5(3)	C26	C27	C28	C23	-0.2(5)
C20	C21	C22	C23	-1.9(4)	C9	C10	C11	C12	0.1(7)
C61	C52	C53	C54	179.1(3)	C73	C74	C75	C76	0.9(6)
C17	C18	C13	P1	-177.3(2)	C92	C87	C86	C85	-180.0(4)
C17	C18	C13	C14	-1.2(4)	C93B	C84	C85	C86	178.2(4)
C17	C18	C19	C20	-101.4(3)	C28	C23	C22	C21	179.6(3)
C17	C18	C19	C24	78.2(3)	C86	C87	C92	C91	179.6(3)
C7	P1	C13	C14	65.7(3)	C85	C84	C93B	C94B	-111.1(5)
C7	P1	C13	C18	-118.3(2)	C85	C84	C93B	C95B	66.7(6)
C7	P1	C6	C5	8.5(3)	C85	C84	C93A	C94A	-128.3(12)
C7	P1	C6	C1	-172.7(3)	C85	C84	C93A	C95A	50.8(18)
C7	C12	C11	C10	-0.2(7)	C94B	C93B	C95B	C96B	-3.1(9)
C7	C8	C9	C10	2.0(6)	C94A	C93A	C95A	C96A	-8(2)
C47	C48	C49	C50	0.6(5)	C93A	C84	C85	C86	-165.2(8)

Supplementary Table 14. Hydrogen Atom Coordinates (Å×10[4]) and Isotropic Displacement Parameters (Å[2]×10[3]) for B.

Atom	*x*	*y*	*z*	U(eq)
H48	7584.91	2005.47	4495.61	43
H33	9317.58	7193.46	4273.99	46
H49	5730.46	2481.35	4286.69	42
H14	660.36	-87.74	4043.38	46
H5	422	2412.66	4229.57	48
H46	8761.79	5043.43	4490.79	37

Supplementary Table 14. Hydrogen Atom Coordinates (Å×10[4]) and Isotropic Displacement Parameters (Å[2]×10[3]) for B.

Atom	*x*	*y*	*z*	U(eq)
H78	5625.35	9435.7	2736.5	46
H40	8754.42	5068.01	3964.91	44
H44	6860.46	7849.83	3904.77	42
H17	4172.44	-2302.07	3922.85	49
H47	9096.45	3294.25	4600.46	42
H37	6268.12	6852.85	4719.9	51
H79	5340.51	11211.05	2834.06	53
H63	5827.37	5281.8	3576.09	52
H30A	3218.19	-1471.83	4505.03	76
H30B	4202.8	-1853.08	4708.52	76
H30C	4466.82	-2140.6	4432.76	76
H4	-1067.36	3178.11	3969.22	55
H42	9522.94	7034.03	3412.21	50
H15	340.47	-1829.33	3922.51	53
H41	9880.24	5455.54	3614.84	51
H16	2096	-2931.66	3860.11	55
H53	3297.82	4447.62	3636.04	51
H89	9398.86	9675.91	3108.77	52
H57	4827.79	4638.89	4619.28	47
H76	5489.04	6779.53	2554.95	55
H35	9466.4	8351.69	4950.38	59
H80	7114.57	12336.06	2848.88	58
H25	4319.35	-385.71	3600.34	50
H43	8023.95	8238.13	3562.12	50
H65	8147.93	6512.77	2276.73	51
H74	4168.41	6214.58	3224.8	63
H54	1651.98	4993.21	3879.98	57
H3	-764.99	3097.2	3558.28	62
H69	6017.65	9172.81	2291.42	65
H21	7327.82	-110.32	4455.23	52
H58	3157.6	5233.41	4859.03	61
H34	10417.59	8087.64	4575.98	57
H36	7381.39	7740.26	5020.24	63
H75	4001.48	6002.76	2812.06	64
H81	9144.14	11700.26	2759.02	57

Supplementary Table 14. Hydrogen Atom Coordinates (Å×10^4) and Isotropic Displacement Parameters (Å^2×10^3) for B.

Atom	*x*	*y*	*z*	U(eq)
H22	8485.08	368.48	4122.96	57
H90	10648.65	9133.18	3427.54	63
H12	1055.27	-5.4	4472.08	63
H62A	6604.36	2591	3854.31	82
H62B	6343.16	2521.25	3571.26	82
H62C	5193.2	2320	3757.03	82
H2	1048.94	2238.37	3401.1	68
H1	2532.02	1487.15	3657.61	59
H91	12798.4	8557.83	3359.98	71
H60	850.24	5511.16	4279.55	67
H66	7389.21	6134.76	1893.06	58
H8	3020.28	2731.84	4526.4	59
H72	7320.03	8018.38	3126.12	70
H68	5281.76	8802.99	1904.77	79
H26	5495.68	86.55	3268.07	66
H9	2037.25	3061.54	4893.35	72
H31	5443.04	746.65	4653.21	62
H73	5820.6	7269.11	3381.16	74
H64A	7880.57	4590.93	3399.81	82
H64B	6722.2	4725.72	3212.77	82
H64C	7032.08	3588.25	3324.63	82
H92	13622.37	8554.13	2975.36	68
H10	614.09	1843.05	5057.23	74
H11	125.94	319.76	4843.75	79
H27	7653.25	604.59	3304.01	72
H59	1164.66	5688.25	4686.2	72
H28	8630.71	635.9	3677.75	67
H86	13355.83	8910.93	2535.67	73
H67	5995.41	7302.83	1703.53	67
H32A	4780.15	477.49	5044.4	106
H32B	4128.84	-625.19	4973.06	106
H32C	3417.91	462.62	4910.3	106
H85	12112.98	9464.37	2223.13	74
H94A	10049.83	11809.71	2206.27	86
H94B	8642.5	11528.35	2303.97	86

Supplementary Table 14. Hydrogen Atom Coordinates (Å×10[4]) and Isotropic Displacement Parameters (Å[2]×10[3]) for B.

Atom	*x*	*y*	*z*	U(eq)
H94C	8930.66	11502.44	2021.45	86
H95B	9635.02	8821.73	2040.49	61
H96A	7653.13	9505.56	1816.04	91
H96B	8928.62	9543.04	1655.08	91
H96C	8432.01	10589.69	1787.41	91
H94D	10068.01	11303.31	2160.1	80
H94E	8758.61	10880.1	2276.98	80
H94F	8985.88	10781.35	1993.36	80
H95A	10419.07	8320.99	2019.55	65
H96D	8314.02	9770.78	1908.47	91
H96E	8807.97	8939.78	1712.16	91
H96F	9530.18	10048.56	1743.7	91

Supplementary Table 15. Atomic Occupancy for B.

Atom	*Occupancy*	Atom	*Occupancy*	Atom	*Occupancy*
C93B	0.728(9)	C94B	0.728(9)	H94A	0.728(9)
H94B	0.728(9)	H94C	0.728(9)	C95B	0.728(9)
H95B	0.728(9)	C96B	0.728(9)	H96A	0.728(9)
H96B	0.728(9)	H96C	0.728(9)	C94A	0.272(9)
H94D	0.272(9)	H94E	0.272(9)	H94F	0.272(9)
C93A	0.272(9)	C95A	0.272(9)	H95A	0.272(9)
C96A	0.272(9)	H96D	0.272(9)	H96E	0.272(9)
H96F	0.272(9)				

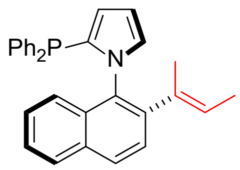

3va

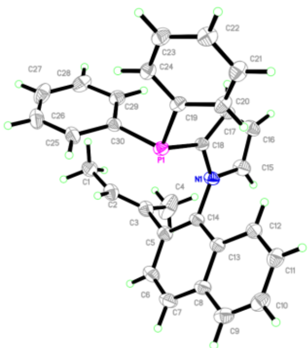

CCDC 2169737

Supplementary Table 16. Crystal data and structure refinement for 20200610_szz_lzx5831_0m_a.

Identification code	20200610_szz_lzx5831_0m_a	
Empirical formula	C30 H26 N P	
Formula weight	431.49	
Temperature	193(2) K	
Wavelength	0.71073 Å	
Crystal system	Orthorhombic	
Space group	$P2_12_12_1$	
Unit cell dimensions	a = 10.1349(5) Å	□= 90°.
	b = 12.6841(6) Å	□= 90°.
	c = 18.3343(7) Å	□ = 90°.
Volume	2356.91(18) Å^3	
Z	4	
Density (calculated)	1.216 Mg/m^3	
Absorption coefficient	0.134 mm^{-1}	
F(000)	912	
Crystal size	0.120 x 0.110 x 0.080 mm^3	
Theta range for data collection	2.296 to 27.490°.	
Index ranges	-13<=h<=10, -16<=k<=16, -23<=l<=23	
Reflections collected	22372	
Independent reflections	5388 [R(int) = 0.0422]	
Completeness to theta = 25.242°	99.7 %	
Refinement method	Full-matrix least-squares on F^2	
Data / restraints / parameters	5388 / 0 / 291	
Goodness-of-fit on F^2	1.027	
Final R indices [I>2sigma(I)]	R1 = 0.0367, wR2 = 0.0958	
R indices (all data)	R1 = 0.0448, wR2 = 0.1019	
Absolute structure parameter	-0.11(4)	

Extinction coefficient	n/a
Largest diff. peak and hole	0.161 and -0.243 e.Å^{-3}

Supplementary Table 17. Atomic coordinates (x 10^4) and equivalent isotropic displacement parameters (Å^{2}x 10^3)

for 20200610_szz_lzx5831_0m_a. U(eq) is defined as one third of the trace of the orthogonalized U^{ij} tensor.

	x	y	z	U(eq)
C(1)	3179(3)	7447(2)	307(2)	54(1)
C(2)	3975(3)	7217(2)	975(1)	48(1)
C(3)	4538(3)	7903(2)	1411(1)	45(1)
C(4)	4497(5)	9071(3)	1292(2)	88(1)
C(5)	5325(3)	7534(2)	2058(1)	42(1)
C(6)	6634(3)	7159(2)	1955(2)	51(1)
C(7)	7395(3)	6826(2)	2526(2)	51(1)
C(8)	6911(3)	6856(2)	3249(2)	42(1)
C(9)	7682(3)	6532(2)	3853(2)	51(1)
C(10)	7187(3)	6583(2)	4544(2)	53(1)
C(11)	5905(3)	6965(2)	4664(2)	50(1)
C(12)	5130(3)	7276(2)	4095(1)	43(1)
C(13)	5610(2)	7234(2)	3368(1)	37(1)
C(14)	4845(3)	7558(2)	2760(1)	37(1)
C(15)	3167(3)	8927(2)	3050(2)	49(1)
C(16)	1876(3)	8940(2)	3254(2)	48(1)
C(17)	1418(3)	7896(2)	3239(1)	40(1)
C(18)	2447(2)	7253(2)	3026(1)	33(1)
C(19)	1351(2)	5346(2)	3541(1)	36(1)
C(20)	1400(3)	5646(2)	4275(1)	42(1)
C(21)	535(3)	5221(2)	4777(1)	49(1)
C(22)	-388(3)	4483(2)	4564(2)	47(1)
C(23)	-449(3)	4176(2)	3837(2)	48(1)
C(24)	421(3)	4605(2)	3332(1)	41(1)
C(25)	2212(3)	4660(2)	1672(1)	46(1)
C(26)	1671(3)	4412(2)	999(2)	56(1)
C(27)	810(4)	5093(3)	671(2)	62(1)

C(28)	438(3)	6010(2)	1019(2)	54(1)
C(29)	948(3)	6258(2)	1698(1)	42(1)
C(30)	1847(2)	5590(2)	2029(1)	36(1)
N(1)	3520(2)	7904(2)	2903(1)	37(1)
P(1)	2623(1)	5843(1)	2913(1)	35(1)

Supplementary Table 18.. Bond lengths [Å] and angles [°] for 20200610_szz_lzx5831_0m_a.

C(1)-C(2)	1.494(4)	C(14)-N(1)	1.436(3)
C(1)-H(1A)	0.9800	C(15)-C(16)	1.362(4)
C(1)-H(1B)	0.9800	C(15)-N(1)	1.372(3)
C(1)-H(1C)	0.9800	C(15)-H(15)	0.9500
C(2)-C(3)	1.313(4)	C(16)-C(17)	1.402(4)
C(2)-H(2)	0.9500	C(16)-H(16)	0.9500
C(3)-C(4)	1.498(4)	C(17)-C(18)	1.380(3)
C(3)-C(5)	1.505(4)	C(17)-H(17)	0.9500
C(4)-H(4A)	0.9800	C(18)-N(1)	1.384(3)
C(4)-H(4B)	0.9800	C(18)-P(1)	1.809(2)
C(4)-H(4C)	0.9800	C(19)-C(24)	1.386(3)
C(5)-C(14)	1.377(3)	C(19)-C(20)	1.399(3)
C(5)-C(6)	1.422(4)	C(19)-P(1)	1.839(3)
C(6)-C(7)	1.366(4)	C(20)-C(21)	1.381(4)
C(6)-H(6)	0.9500	C(20)-H(20)	0.9500
C(7)-C(8)	1.415(4)	C(21)-C(22)	1.380(4)
C(7)-H(7)	0.9500	C(21)-H(21)	0.9500
C(8)-C(9)	1.416(4)	C(22)-C(23)	1.389(4)
C(8)-C(13)	1.420(3)	C(22)-H(22)	0.9500
C(9)-C(10)	1.363(4)	C(23)-C(24)	1.390(4)
C(9)-H(9)	0.9500	C(23)-H(23)	0.9500
C(10)-C(11)	1.404(4)	C(24)-H(24)	0.9500
C(10)-H(10)	0.9500	C(25)-C(26)	1.386(4)
C(11)-C(12)	1.365(4)	C(25)-C(30)	1.399(3)
C(11)-H(11)	0.9500	C(25)-H(25)	0.9500
C(12)-C(13)	1.420(3)	C(26)-C(27)	1.366(5)
C(12)-H(12)	0.9500	C(26)-H(26)	0.9500
C(13)-C(14)	1.418(4)	C(27)-C(28)	1.379(5)

C(27)-H(27)	0.9500	C(10)-C(9)-C(8)	120.6(3)
C(28)-C(29)	1.385(4)	C(10)-C(9)-H(9)	119.7
C(28)-H(28)	0.9500	C(8)-C(9)-H(9)	119.7
C(29)-C(30)	1.384(3)	C(9)-C(10)-C(11)	120.2(3)
C(29)-H(29)	0.9500	C(9)-C(10)-H(10)	119.9
C(30)-P(1)	1.830(2)	C(11)-C(10)-H(10)	119.9
C(2)-C(1)-H(1A)	109.5	C(12)-C(11)-C(10)	120.8(3)
C(2)-C(1)-H(1B)	109.5	C(12)-C(11)-H(11)	119.6
H(1A)-C(1)-H(1B)	109.5	C(10)-C(11)-H(11)	119.6
C(2)-C(1)-H(1C)	109.5	C(11)-C(12)-C(13)	120.7(3)
H(1A)-C(1)-H(1C)	109.5	C(11)-C(12)-H(12)	119.6
H(1B)-C(1)-H(1C)	109.5	C(13)-C(12)-H(12)	119.6
C(3)-C(2)-C(1)	127.2(3)	C(14)-C(13)-C(12)	122.7(2)
C(3)-C(2)-H(2)	116.4	C(14)-C(13)-C(8)	119.0(2)
C(1)-C(2)-H(2)	116.4	C(12)-C(13)-C(8)	118.3(2)
C(2)-C(3)-C(4)	123.7(3)	C(5)-C(14)-C(13)	122.3(2)
C(2)-C(3)-C(5)	120.3(2)	C(5)-C(14)-N(1)	120.5(2)
C(4)-C(3)-C(5)	115.9(2)	C(13)-C(14)-N(1)	117.2(2)
C(3)-C(4)-H(4A)	109.5	C(16)-C(15)-N(1)	108.4(2)
C(3)-C(4)-H(4B)	109.5	C(16)-C(15)-H(15)	125.8
H(4A)-C(4)-H(4B)	109.5	N(1)-C(15)-H(15)	125.8
C(3)-C(4)-H(4C)	109.5	C(15)-C(16)-C(17)	107.5(2)
H(4A)-C(4)-H(4C)	109.5	C(15)-C(16)-H(16)	126.2
H(4B)-C(4)-H(4C)	109.5	C(17)-C(16)-H(16)	126.2
C(14)-C(5)-C(6)	117.5(2)	C(18)-C(17)-C(16)	108.3(2)
C(14)-C(5)-C(3)	122.9(2)	C(18)-C(17)-H(17)	125.9
C(6)-C(5)-C(3)	119.6(2)	C(16)-C(17)-H(17)	125.9
C(7)-C(6)-C(5)	121.9(3)	C(17)-C(18)-N(1)	106.69(19)
C(7)-C(6)-H(6)	119.1	C(17)-C(18)-P(1)	133.70(19)
C(5)-C(6)-H(6)	119.1	N(1)-C(18)-P(1)	119.60(17)
C(6)-C(7)-C(8)	120.9(3)	C(24)-C(19)-C(20)	118.4(2)
C(6)-C(7)-H(7)	119.5	C(24)-C(19)-P(1)	122.39(19)
C(8)-C(7)-H(7)	119.5	C(20)-C(19)-P(1)	118.93(19)
C(7)-C(8)-C(9)	122.3(2)	C(21)-C(20)-C(19)	120.8(2)
C(7)-C(8)-C(13)	118.3(2)	C(21)-C(20)-H(20)	119.6
C(9)-C(8)-C(13)	119.4(3)	C(19)-C(20)-H(20)	119.6

C(20)-C(21)-C(22)	120.5(2)	C(26)-C(27)-C(28)	120.3(3)
C(20)-C(21)-H(21)	119.8	C(26)-C(27)-H(27)	119.9
C(22)-C(21)-H(21)	119.8	C(28)-C(27)-H(27)	119.9
C(21)-C(22)-C(23)	119.4(3)	C(27)-C(28)-C(29)	120.3(3)
C(21)-C(22)-H(22)	120.3	C(27)-C(28)-H(28)	119.8
C(23)-C(22)-H(22)	120.3	C(29)-C(28)-H(28)	119.8
C(24)-C(23)-C(22)	120.1(3)	C(28)-C(29)-C(30)	120.1(2)
C(24)-C(23)-H(23)	119.9	C(28)-C(29)-H(29)	120.0
C(22)-C(23)-H(23)	119.9	C(30)-C(29)-H(29)	120.0
C(19)-C(24)-C(23)	120.8(2)	C(29)-C(30)-C(25)	119.0(2)
C(19)-C(24)-H(24)	119.6	C(29)-C(30)-P(1)	124.31(18)
C(23)-C(24)-H(24)	119.6	C(25)-C(30)-P(1)	116.70(19)
C(26)-C(25)-C(30)	120.2(3)	C(15)-N(1)-C(18)	109.1(2)
C(26)-C(25)-H(25)	119.9	C(15)-N(1)-C(14)	124.6(2)
C(30)-C(25)-H(25)	119.9	C(18)-N(1)-C(14)	125.56(18)
C(27)-C(26)-C(25)	120.0(3)	C(18)-P(1)-C(30)	103.45(10)
C(27)-C(26)-H(26)	120.0	C(18)-P(1)-C(19)	101.43(11)
C(25)-C(26)-H(26)	120.0	C(30)-P(1)-C(19)	101.15(11)

Symmetry transformations used to generate equivalent atoms:

Supplementary Table 19.. Anisotropic displacement parameters (Å^{2}x 10^3) for 20200610_szz_lzx5831_0m_a. The anisotropic displacement factor exponent takes the form: -2□^{2}[h^2 a*^{2}U^{11} + ... + 2 h k a* b* U^{12}]

	U^{11}	U^{22}	U^{33}	U^{23}	U^{13}	U^{12}
C(1)	53(2)	62(2)	47(1)	-1(1)	10(1)	-5(2)
C(2)	55(2)	46(1)	42(1)	2(1)	9(1)	-2(1)
C(3)	49(2)	42(1)	44(1)	2(1)	9(1)	-4(1)
C(4)	136(4)	46(2)	82(2)	10(2)	-41(2)	-11(2)
C(5)	41(1)	37(1)	47(1)	0(1)	8(1)	-2(1)
C(6)	48(2)	55(2)	51(2)	-3(1)	14(1)	4(1)
C(7)	38(1)	50(2)	66(2)	-3(1)	9(1)	4(1)
C(8)	35(1)	32(1)	60(2)	-2(1)	2(1)	-3(1)
C(9)	42(2)	38(1)	75(2)	4(1)	-6(1)	-2(1)

C(10)	56(2)	44(1)	60(2)	4(1)	-14(1)	-7(1)
C(11)	59(2)	45(1)	47(1)	0(1)	-2(1)	-12(1)
C(12)	41(1)	38(1)	48(1)	-3(1)	5(1)	-6(1)
C(13)	35(1)	29(1)	48(1)	-2(1)	5(1)	-4(1)
C(14)	32(1)	30(1)	49(1)	-2(1)	6(1)	-3(1)
C(15)	49(2)	29(1)	69(2)	1(1)	8(1)	0(1)
C(16)	48(2)	36(1)	60(2)	0(1)	8(1)	11(1)
C(17)	35(1)	39(1)	47(1)	1(1)	5(1)	3(1)
C(18)	31(1)	32(1)	36(1)	0(1)	1(1)	-1(1)
C(19)	36(1)	30(1)	41(1)	5(1)	-7(1)	1(1)
C(20)	46(2)	41(1)	40(1)	5(1)	-12(1)	-8(1)
C(21)	61(2)	50(2)	37(1)	8(1)	-7(1)	-6(1)
C(22)	52(2)	42(1)	48(1)	12(1)	0(1)	-6(1)
C(23)	48(2)	37(1)	58(2)	1(1)	-4(1)	-9(1)
C(24)	44(2)	36(1)	43(1)	-2(1)	-4(1)	-3(1)
C(25)	50(2)	39(1)	49(1)	-6(1)	12(1)	0(1)
C(26)	76(2)	46(2)	48(2)	-12(1)	20(2)	-15(2)
C(27)	84(2)	63(2)	39(1)	0(1)	1(2)	-35(2)
C(28)	55(2)	58(2)	49(1)	13(1)	-11(1)	-14(2)
C(29)	41(1)	40(1)	45(1)	3(1)	-2(1)	-3(1)
C(30)	34(1)	33(1)	40(1)	-1(1)	6(1)	-2(1)
N(1)	32(1)	30(1)	49(1)	1(1)	5(1)	1(1)
P(1)	31(1)	30(1)	44(1)	0(1)	-3(1)	3(1)

Supplementary Table 20.. Hydrogen coordinates (x 10^4) and isotropic displacement parameters (Å^{2}x 10 ^{3})
for 20200610_szz_lzx5831_0m_a.

	x	y	z	U(eq)
H(1A)	2260	7240	390	81
H(1B)	3537	7049	-106	81
H(1C)	3220	8203	200	81
H(2)	4085	6494	1097	57
H(4A)	3926	9230	875	132

H(4B)	5391	9330	1194	132
H(4C)	4149	9417	1729	132
H(6)	6990	7140	1476	61
H(7)	8261	6571	2435	62
H(9)	8552	6275	3776	62
H(10)	7711	6359	4944	64
H(11)	5573	7007	5148	60
H(12)	4261	7524	4186	51
H(15)	3729	9524	3015	59
H(16)	1376	9545	3384	57
H(17)	549	7671	3355	48
H(20)	2037	6148	4430	51
H(21)	575	5439	5272	59
H(22)	-977	4187	4911	57
H(23)	-1086	3672	3686	57
H(24)	378	4386	2837	49
H(25)	2832	4197	1891	55
H(26)	1899	3769	765	68
H(27)	465	4933	202	74
H(28)	-171	6474	791	65
H(29)	681	6887	1938	51

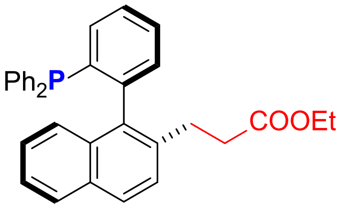

5aa

≡

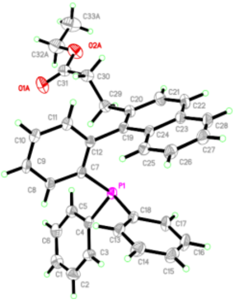

CCDC 2169736

Supplementary Table 21. Crystal data and structure refinement for 20191112LI_SZZ_LZX5_38_1_0m_a.

Identification code	20191112LI_SZZ_LZX5_38_1_0m_a
Empirical formula	C33 H29 O2 P
Formula weight	488.53

Temperature	190(2) K	
Wavelength	1.34138 Å	
Crystal system	Monoclinic	
Space group	C2	
Unit cell dimensions	a = 25.549(3) Å	□= 90°.
	b = 6.9723(8) Å	□= 122.969(4)°.
	c = 17.796(2) Å	□ = 90°.
Volume	2659.6(5) $Å^3$	
Z	4	
Density (calculated)	1.220 Mg/m^3	
Absorption coefficient	0.729 mm^{-1}	
F(000)	1032	
Crystal size	0.080 x 0.060 x 0.050 mm^3	
Theta range for data collection	2.575 to 51.243°.	
Index ranges	-29<=h<=29, -8<=k<=8, -20<=l<=20	
Reflections collected	16203	
Independent reflections	4345 [R(int) = 0.0582]	
Completeness to theta = 51.243°	99.6 %	
Refinement method	Full-matrix least-squares on F^2	
Data / restraints / parameters	4345 / 56 / 365	
Goodness-of-fit on F^2	1.042	
Final R indices [I>2sigma(I)]	R1 = 0.0392, wR2 = 0.0979	
R indices (all data)	R1 = 0.0431, wR2 = 0.1008	
Absolute structure parameter	0.05(4)	
Extinction coefficient	n/a	
Largest diff. peak and hole	0.277 and -0.289 $e.Å^{-3}$	

Supplementary Table 22. Atomic coordinates (x 10^4) and equivalent isotropic displacement parameters ($Å^2$x 10^3) for 20191112LI_SZZ_LZX5_38_1_0m_a. U(eq) is defined as one third of the trace of the

orthogonalized U^{ij} tensor.

	x	y	z	U(eq)
P(1)	8214(1)	5054(1)	7870(1)	36(1)
C(1)	9670(2)	8052(6)	7501(3)	54(1)
C(2)	9867(2)	6641(6)	8141(3)	58(1)
C(3)	9447(2)	5660(5)	8256(2)	46(1)
C(4)	8815(2)	6083(5)	7729(2)	37(1)
C(5)	8624(2)	7520(5)	7087(2)	46(1)
C(6)	9047(2)	8479(6)	6966(3)	53(1)
C(7)	7791(1)	3454(5)	6892(2)	33(1)
C(8)	8010(2)	2940(5)	6355(2)	39(1)
C(9)	7664(2)	1743(5)	5618(2)	44(1)
C(10)	7103(2)	1044(5)	5420(2)	44(1)
C(11)	6876(2)	1531(5)	5948(2)	39(1)
C(12)	7211(2)	2743(4)	6678(2)	33(1)
C(13)	8843(2)	1526(5)	8664(2)	46(1)
C(14)	9155(2)	264(7)	9371(2)	57(1)
C(15)	9275(2)	768(6)	10203(3)	59(1)
C(16)	9089(2)	2515(7)	10320(2)	64(1)
C(17)	8772(2)	3767(6)	9611(2)	51(1)
C(18)	8648(2)	3301(5)	8773(2)	37(1)
C(19)	6942(1)	3266(5)	7217(2)	34(1)
C(20)	6613(1)	4960(5)	7045(2)	38(1)
C(21)	6363(2)	5434(5)	7561(2)	48(1)
C(22)	6436(2)	4245(6)	8223(2)	50(1)
C(23)	6753(2)	2496(5)	8397(2)	41(1)
C(24)	7018(2)	1992(5)	7900(2)	35(1)
C(25)	7338(2)	215(6)	8093(2)	41(1)
C(26)	7393(2)	-979(6)	8739(2)	50(1)
C(27)	7127(2)	-493(6)	9221(2)	56(1)
C(28)	6820(2)	1200(6)	9065(2)	50(1)
C(29)	6495(2)	6292(5)	6306(2)	42(1)

C(30)	5894(2)	5764(5)	5425(2)	45(1)
C(31)	5755(2)	7130(5)	4689(2)	44(1)
O(1A)	6046(3)	7208(8)	4356(3)	66(1)
O(2A)	5330(2)	8394(7)	4552(3)	68(1)
C(32A)	5180(3)	9843(11)	3875(5)	76(2)
C(33A)	4673(4)	11018(14)	3788(7)	98(3)
O(1B)	6080(6)	8350(20)	4671(10)	104(4)
O(2B)	5182(5)	6777(15)	3887(7)	69(2)
C(32B)	4982(7)	8320(30)	3185(11)	72(3)
C(33B)	4660(8)	9910(30)	3328(12)	74(3)

Supplementary Table 23. Bond lengths [Å] and angles [°] for 20191112LI_SZZ_LZX5_38_1_0m_a.

P(1)-C(4)	1.831(3)	C(11)-C(12)	1.388(4)
P(1)-C(18)	1.835(3)	C(11)-H(11)	0.9500
P(1)-C(7)	1.843(3)	C(12)-C(19)	1.498(4)
C(1)-C(6)	1.370(6)	C(13)-C(14)	1.379(5)
C(1)-C(2)	1.375(6)	C(13)-C(18)	1.387(5)
C(1)-H(1)	0.9500	C(13)-H(13)	0.9500
C(2)-C(3)	1.379(5)	C(14)-C(15)	1.384(6)
C(2)-H(2)	0.9500	C(14)-H(14)	0.9500
C(3)-C(4)	1.388(5)	C(15)-C(16)	1.364(6)
C(3)-H(3)	0.9500	C(15)-H(15)	0.9500
C(4)-C(5)	1.393(5)	C(16)-C(17)	1.377(5)
C(5)-C(6)	1.385(5)	C(16)-H(16)	0.9500
C(5)-H(5)	0.9500	C(17)-C(18)	1.384(4)
C(6)-H(6)	0.9500	C(17)-H(17)	0.9500
C(7)-C(8)	1.393(4)	C(19)-C(20)	1.384(5)
C(7)-C(12)	1.404(4)	C(19)-C(24)	1.431(4)
C(8)-C(9)	1.392(5)	C(20)-C(21)	1.414(5)
C(8)-H(8)	0.9500	C(20)-C(29)	1.500(5)
C(9)-C(10)	1.366(5)	C(21)-C(22)	1.367(5)
C(9)-H(9)	0.9500	C(21)-H(21)	0.9500
C(10)-C(11)	1.388(5)	C(22)-C(23)	1.402(6)
C(10)-H(10)	0.9500	C(22)-H(22)	0.9500

C(23)-C(24)	1.419(5)	C(18)-P(1)-C(7)	100.83(15)
C(23)-C(28)	1.428(5)	C(6)-C(1)-C(2)	119.6(4)
C(24)-C(25)	1.420(5)	C(6)-C(1)-H(1)	120.2
C(25)-C(26)	1.363(5)	C(2)-C(1)-H(1)	120.2
C(25)-H(25)	0.9500	C(1)-C(2)-C(3)	121.0(4)
C(26)-C(27)	1.395(6)	C(1)-C(2)-H(2)	119.5
C(26)-H(26)	0.9500	C(3)-C(2)-H(2)	119.5
C(27)-C(28)	1.360(6)	C(2)-C(3)-C(4)	120.4(4)
C(27)-H(27)	0.9500	C(2)-C(3)-H(3)	119.8
C(28)-H(28)	0.9500	C(4)-C(3)-H(3)	119.8
C(29)-C(30)	1.526(5)	C(3)-C(4)-C(5)	117.9(3)
C(29)-H(29A)	0.9900	C(3)-C(4)-P(1)	125.3(3)
C(29)-H(29B)	0.9900	C(5)-C(4)-P(1)	116.6(3)
C(30)-C(31)	1.497(5)	C(6)-C(5)-C(4)	121.3(4)
C(30)-H(30A)	0.9900	C(6)-C(5)-H(5)	119.4
C(30)-H(30B)	0.9900	C(4)-C(5)-H(5)	119.4
C(31)-O(1A)	1.177(6)	C(1)-C(6)-C(5)	119.8(4)
C(31)-O(1B)	1.200(3)	C(1)-C(6)-H(6)	120.1
C(31)-O(2A)	1.314(5)	C(5)-C(6)-H(6)	120.1
C(31)-O(2B)	1.402(11)	C(8)-C(7)-C(12)	118.8(3)
O(2A)-C(32A)	1.455(7)	C(8)-C(7)-P(1)	123.7(2)
C(32A)-C(33A)	1.466(11)	C(12)-C(7)-P(1)	117.4(2)
C(32A)-H(32B)	0.9900	C(9)-C(8)-C(7)	120.9(3)
C(32A)-H(32A)	0.9900	C(9)-C(8)-H(8)	119.5
C(33A)-H(33A)	0.9800	C(7)-C(8)-H(8)	119.5
C(33A)-H(33B)	0.9800	C(10)-C(9)-C(8)	119.8(3)
C(33A)-H(33C)	0.9800	C(10)-C(9)-H(9)	120.1
O(2B)-C(32B)	1.509(15)	C(8)-C(9)-H(9)	120.1
C(32B)-C(33B)	1.49(2)	C(9)-C(10)-C(11)	120.3(3)
C(32B)-H(32C)	0.9900	C(9)-C(10)-H(10)	119.8
C(32B)-H(32D)	0.9900	C(11)-C(10)-H(10)	119.8
C(33B)-H(33D)	0.9800	C(12)-C(11)-C(10)	120.7(3)
C(33B)-H(33E)	0.9800	C(12)-C(11)-H(11)	119.6
C(33B)-H(33F)	0.9800	C(10)-C(11)-H(11)	119.6
C(4)-P(1)-C(18)	103.27(15)	C(11)-C(12)-C(7)	119.4(3)
C(4)-P(1)-C(7)	102.28(14)	C(11)-C(12)-C(19)	118.9(3)

C(7)-C(12)-C(19)	121.7(3)	C(23)-C(24)-C(19)	119.0(3)
C(14)-C(13)-C(18)	121.0(3)	C(26)-C(25)-C(24)	121.1(3)
C(14)-C(13)-H(13)	119.5	C(26)-C(25)-H(25)	119.4
C(18)-C(13)-H(13)	119.5	C(24)-C(25)-H(25)	119.4
C(13)-C(14)-C(15)	119.8(4)	C(25)-C(26)-C(27)	120.6(4)
C(13)-C(14)-H(14)	120.1	C(25)-C(26)-H(26)	119.7
C(15)-C(14)-H(14)	120.1	C(27)-C(26)-H(26)	119.7
C(16)-C(15)-C(14)	119.8(4)	C(28)-C(27)-C(26)	120.4(3)
C(16)-C(15)-H(15)	120.1	C(28)-C(27)-H(27)	119.8
C(14)-C(15)-H(15)	120.1	C(26)-C(27)-H(27)	119.8
C(15)-C(16)-C(17)	120.4(3)	C(27)-C(28)-C(23)	120.9(3)
C(15)-C(16)-H(16)	119.8	C(27)-C(28)-H(28)	119.6
C(17)-C(16)-H(16)	119.8	C(23)-C(28)-H(28)	119.6
C(16)-C(17)-C(18)	120.9(4)	C(20)-C(29)-C(30)	111.2(3)
C(16)-C(17)-H(17)	119.5	C(20)-C(29)-H(29A)	109.4
C(18)-C(17)-H(17)	119.5	C(30)-C(29)-H(29A)	109.4
C(17)-C(18)-C(13)	118.1(3)	C(20)-C(29)-H(29B)	109.4
C(17)-C(18)-P(1)	117.5(3)	C(30)-C(29)-H(29B)	109.4
C(13)-C(18)-P(1)	124.3(2)	H(29A)-C(29)-H(29B)	108.0
C(20)-C(19)-C(24)	120.2(3)	C(31)-C(30)-C(29)	111.9(3)
C(20)-C(19)-C(12)	120.0(3)	C(31)-C(30)-H(30A)	109.2
C(24)-C(19)-C(12)	119.7(3)	C(29)-C(30)-H(30A)	109.2
C(19)-C(20)-C(21)	119.3(3)	C(31)-C(30)-H(30B)	109.2
C(19)-C(20)-C(29)	122.1(3)	C(29)-C(30)-H(30B)	109.2
C(21)-C(20)-C(29)	118.6(3)	H(30A)-C(30)-H(30B)	107.9
C(22)-C(21)-C(20)	121.3(4)	O(1A)-C(31)-O(2A)	124.1(4)
C(22)-C(21)-H(21)	119.4	O(1B)-C(31)-O(2B)	116.8(8)
C(20)-C(21)-H(21)	119.4	O(1A)-C(31)-C(30)	123.7(4)
C(21)-C(22)-C(23)	120.7(3)	O(1B)-C(31)-C(30)	129.4(7)
C(21)-C(22)-H(22)	119.7	O(2A)-C(31)-C(30)	111.6(3)
C(23)-C(22)-H(22)	119.7	O(2B)-C(31)-C(30)	113.4(5)
C(22)-C(23)-C(24)	119.4(3)	C(31)-O(2A)-C(32A)	116.0(4)
C(22)-C(23)-C(28)	121.9(3)	O(2A)-C(32A)-C(33A)	107.2(6)
C(24)-C(23)-C(28)	118.7(3)	O(2A)-C(32A)-H(32B)	110.3
C(25)-C(24)-C(23)	118.3(3)	C(33A)-C(32A)-H(32B)	110.3
C(25)-C(24)-C(19)	122.7(3)	O(2A)-C(32A)-H(32A)	110.3

C(33A)-C(32A)-H(32A)	110.3	O(2B)-C(32B)-H(32C)	109.4
H(32B)-C(32A)-H(32A)	108.5	C(33B)-C(32B)-H(32D)	109.4
C(32A)-C(33A)-H(33A)	109.5	O(2B)-C(32B)-H(32D)	109.4
C(32A)-C(33A)-H(33B)	109.5	H(32C)-C(32B)-H(32D)	108.0
H(33A)-C(33A)-H(33B)	109.5	C(32B)-C(33B)-H(33D)	109.5
C(32A)-C(33A)-H(33C)	109.5	C(32B)-C(33B)-H(33E)	109.5
H(33A)-C(33A)-H(33C)	109.5	H(33D)-C(33B)-H(33E)	109.5
H(33B)-C(33A)-H(33C)	109.5	C(32B)-C(33B)-H(33F)	109.5
C(31)-O(2B)-C(32B)	114.4(10)	H(33D)-C(33B)-H(33F)	109.5
C(33B)-C(32B)-O(2B)	111.1(12)	H(33E)-C(33B)-H(33F)	109.5
C(33B)-C(32B)-H(32C)	109.4		

Symmetry transformations used to generate equivalent atoms:

Supplementary Table 24. Anisotropic displacement parameters ($Å^2$x 10^3) for 20191112LI_SZZ_LZX5_38_1_0m_a. The anisotropic displacement factor exponent takes the form: $-2\square^2[h^2 a^{*2}U^{11} + ... + 2 h k a^* b^* U^{12}]$

	U^{11}	U^{22}	U^{33}	U^{23}	U^{13}	U^{12}
P(1)	41(1)	37(1)	31(1)	-4(1)	20(1)	0(1)
C(1)	65(3)	45(2)	66(2)	-13(2)	43(2)	-17(2)
C(2)	47(2)	62(3)	65(2)	-8(2)	30(2)	-7(2)
C(3)	42(2)	46(2)	45(2)	-2(2)	21(2)	-2(2)
C(4)	48(2)	32(2)	32(2)	-7(1)	22(2)	-4(2)
C(5)	51(2)	44(2)	45(2)	1(2)	26(2)	5(2)
C(6)	72(3)	44(2)	54(2)	1(2)	41(2)	-4(2)
C(7)	42(2)	32(2)	26(1)	4(1)	18(1)	5(1)
C(8)	44(2)	43(2)	32(2)	4(1)	23(2)	4(2)
C(9)	59(2)	47(2)	30(2)	0(2)	27(2)	7(2)
C(10)	53(2)	44(2)	31(2)	-4(2)	21(2)	-1(2)
C(11)	48(2)	36(2)	33(2)	-2(1)	21(2)	0(2)

C(12)	40(2)	32(2)	28(2)	5(1)	18(1)	5(1)
C(13)	54(2)	46(2)	32(2)	-4(2)	18(2)	6(2)
C(14)	61(2)	50(2)	50(2)	5(2)	24(2)	9(2)
C(15)	61(2)	64(3)	42(2)	18(2)	22(2)	6(2)
C(16)	81(3)	79(3)	33(2)	4(2)	33(2)	3(2)
C(17)	68(2)	57(2)	36(2)	-1(2)	33(2)	4(2)
C(18)	40(2)	41(2)	31(2)	-2(1)	20(1)	-6(2)
C(19)	37(2)	35(2)	28(2)	-4(1)	17(1)	-5(1)
C(20)	44(2)	33(2)	41(2)	-1(2)	25(1)	-4(2)
C(21)	59(2)	40(2)	55(2)	-4(2)	38(2)	1(2)
C(22)	64(2)	52(2)	49(2)	-9(2)	41(2)	-8(2)
C(23)	45(2)	47(2)	33(2)	-5(2)	23(2)	-11(2)
C(24)	37(2)	38(2)	28(1)	-2(1)	17(1)	-8(1)
C(25)	44(2)	42(2)	33(2)	-1(2)	19(1)	-3(2)
C(26)	55(2)	47(2)	44(2)	12(2)	24(2)	1(2)
C(27)	60(2)	64(3)	41(2)	15(2)	26(2)	-4(2)
C(28)	57(2)	63(3)	37(2)	-1(2)	30(2)	-12(2)
C(29)	46(2)	35(2)	48(2)	2(2)	28(2)	4(2)
C(30)	44(2)	38(2)	52(2)	7(2)	25(2)	2(2)
C(31)	42(2)	39(2)	48(2)	3(2)	23(2)	2(2)
O(1A)	87(3)	63(3)	76(3)	18(3)	64(3)	21(3)
O(2A)	68(2)	66(2)	83(3)	34(2)	48(2)	28(2)
C(32A)	71(3)	70(3)	82(3)	40(3)	38(3)	23(3)
C(33A)	76(4)	76(5)	127(6)	42(5)	46(4)	20(4)
O(1B)	105(7)	87(8)	81(7)	30(7)	25(6)	-15(8)
O(2B)	62(4)	75(4)	71(4)	38(3)	37(3)	11(3)
C(32B)	67(4)	77(4)	74(4)	41(4)	40(4)	14(4)
C(33B)	70(5)	74(6)	84(6)	44(5)	47(5)	13(6)

Supplementary Table 25. Hydrogen coordinates (x 10^4) and isotropic displacement parameters (Å^{2}x 10 ^{3})

for 20191112LI_SZZ_LZX5_38_1_0m_a.

	x	y	z	U(eq)

H(1)	9964	8727	7431	65
H(2)	10298	6339	8508	70
H(3)	9591	4687	8700	55
H(5)	8194	7848	6724	55
H(6)	8906	9431	6514	64
H(8)	8402	3412	6494	46
H(9)	7817	1413	5253	53
H(10)	6867	220	4920	53
H(11)	6487	1030	5807	47
H(13)	8761	1175	8093	56
H(14)	9287	-947	9287	68
H(15)	9487	-100	10691	71
H(16)	9179	2870	10894	76
H(17)	8637	4967	9699	61
H(21)	6140	6602	7446	57
H(22)	6271	4608	8568	59
H(25)	7518	-148	7766	49
H(26)	7614	-2152	8862	60
H(27)	7160	-1350	9660	67
H(28)	6647	1524	9405	60
H(29A)	6850	6231	6229	50
H(29B)	6463	7624	6470	50
H(30A)	5933	4450	5249	54
H(30B)	5542	5769	5511	54
H(32B)	5045	9227	3296	91
H(32A)	5550	10649	4059	91
H(33A)	4808	11589	4369	147
H(33B)	4306	10212	3587	147
H(33C)	4568	12039	3349	147
H(32C)	5353	8821	3209	87
H(32D)	4698	7754	2584	87
H(33D)	4954	10556	3896	110
H(33E)	4310	9402	3346	110

H(33F)	4503	10837	2835	110

Supplementary Table 26. Torsion angles [°] for 20191112LI_SZZ_LZX5_38_1_0m_a.

C(6)-C(1)-C(2)-C(3)	-0.6(6)
C(1)-C(2)-C(3)-C(4)	-0.2(6)
C(2)-C(3)-C(4)-C(5)	0.0(5)
C(2)-C(3)-C(4)-P(1)	-174.3(3)
C(18)-P(1)-C(4)-C(3)	-4.3(3)
C(7)-P(1)-C(4)-C(3)	-108.7(3)
C(18)-P(1)-C(4)-C(5)	-178.7(2)
C(7)-P(1)-C(4)-C(5)	76.9(3)
C(3)-C(4)-C(5)-C(6)	1.0(5)
P(1)-C(4)-C(5)-C(6)	175.8(3)
C(2)-C(1)-C(6)-C(5)	1.5(6)
C(4)-C(5)-C(6)-C(1)	-1.7(5)
C(4)-P(1)-C(7)-C(8)	12.9(3)
C(18)-P(1)-C(7)-C(8)	-93.4(3)
C(4)-P(1)-C(7)-C(12)	-166.3(2)
C(18)-P(1)-C(7)-C(12)	87.4(3)
C(12)-C(7)-C(8)-C(9)	0.0(5)
P(1)-C(7)-C(8)-C(9)	-179.2(3)
C(7)-C(8)-C(9)-C(10)	-0.7(5)
C(8)-C(9)-C(10)-C(11)	0.4(5)
C(9)-C(10)-C(11)-C(12)	0.5(5)
C(10)-C(11)-C(12)-C(7)	-1.1(5)
C(10)-C(11)-C(12)-C(19)	178.7(3)
C(8)-C(7)-C(12)-C(11)	0.8(4)
P(1)-C(7)-C(12)-C(11)	-179.9(2)
C(8)-C(7)-C(12)-C(19)	-178.9(3)
P(1)-C(7)-C(12)-C(19)	0.4(4)
C(18)-C(13)-C(14)-C(15)	0.0(6)
C(13)-C(14)-C(15)-C(16)	-0.5(6)
C(14)-C(15)-C(16)-C(17)	1.2(7)
C(15)-C(16)-C(17)-C(18)	-1.4(6)
C(16)-C(17)-C(18)-C(13)	0.9(6)

C(16)-C(17)-C(18)-P(1)	179.3(3)
C(14)-C(13)-C(18)-C(17)	-0.2(5)
C(14)-C(13)-C(18)-P(1)	-178.5(3)
C(4)-P(1)-C(18)-C(17)	105.6(3)
C(7)-P(1)-C(18)-C(17)	-148.9(3)
C(4)-P(1)-C(18)-C(13)	-76.2(3)
C(7)-P(1)-C(18)-C(13)	29.3(3)
C(11)-C(12)-C(19)-C(20)	-96.0(4)
C(7)-C(12)-C(19)-C(20)	83.8(4)
C(11)-C(12)-C(19)-C(24)	83.0(4)
C(7)-C(12)-C(19)-C(24)	-97.3(4)
C(24)-C(19)-C(20)-C(21)	1.2(5)
C(12)-C(19)-C(20)-C(21)	-179.8(3)
C(24)-C(19)-C(20)-C(29)	-176.9(3)
C(12)-C(19)-C(20)-C(29)	2.0(5)
C(19)-C(20)-C(21)-C(22)	-0.5(5)
C(29)-C(20)-C(21)-C(22)	177.7(3)
C(20)-C(21)-C(22)-C(23)	-1.2(6)
C(21)-C(22)-C(23)-C(24)	2.2(5)
C(21)-C(22)-C(23)-C(28)	-178.0(4)
C(22)-C(23)-C(24)-C(25)	179.7(3)
C(28)-C(23)-C(24)-C(25)	-0.1(5)
C(22)-C(23)-C(24)-C(19)	-1.4(5)
C(28)-C(23)-C(24)-C(19)	178.7(3)
C(20)-C(19)-C(24)-C(25)	178.5(3)
C(12)-C(19)-C(24)-C(25)	-0.4(5)
C(20)-C(19)-C(24)-C(23)	-0.2(4)
C(12)-C(19)-C(24)-C(23)	-179.2(3)
C(23)-C(24)-C(25)-C(26)	-0.1(5)
C(19)-C(24)-C(25)-C(26)	-178.9(3)
C(24)-C(25)-C(26)-C(27)	0.9(5)
C(25)-C(26)-C(27)-C(28)	-1.4(6)
C(26)-C(27)-C(28)-C(23)	1.1(6)
C(22)-C(23)-C(28)-C(27)	179.8(4)
C(24)-C(23)-C(28)-C(27)	-0.4(5)
C(19)-C(20)-C(29)-C(30)	88.5(4)

C(21)-C(20)-C(29)-C(30)	-89.6(4)
C(20)-C(29)-C(30)-C(31)	177.6(3)
C(29)-C(30)-C(31)-O(1A)	70.6(6)
C(29)-C(30)-C(31)-O(1B)	11.3(15)
C(29)-C(30)-C(31)-O(2A)	-100.6(4)
C(29)-C(30)-C(31)-O(2B)	-175.4(6)
O(1A)-C(31)-O(2A)-C(32A)	6.3(8)
C(30)-C(31)-O(2A)-C(32A)	177.5(5)
C(31)-O(2A)-C(32A)-C(33A)	176.5(6)
O(1B)-C(31)-O(2B)-C(32B)	-15.3(16)
C(30)-C(31)-O(2B)-C(32B)	170.5(8)
C(31)-O(2B)-C(32B)-C(33B)	-84.2(14)

Symmetry transformations used to generate equivalent atoms:

Supplementary Table 27. Hydrogen bonds for 20191112LI_SZZ_LZX5_38_1_0m_a [Å and °].

_

D-H...A	d(D-H)	d(H...A)	d(D...A)	<(DHA)

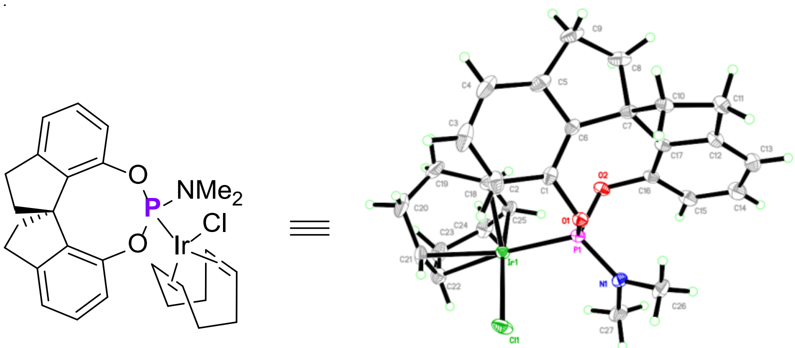

15

CCDC 2169738

Supplementary Table 28. Crystal data and structure refinement for 20200115LI_SZZ_LZX5_84_1_0m_a.

Identification code	20200115LI_SZZ_LZX5_84_1_0m_a
Empirical formula	C27 H32 Cl Ir N O2 P
Formula weight	661.15
Temperature	296(2) K
Wavelength	0.71073 Å

Crystal system	Orthorhombic	
Space group	$P2_12_12_1$	
Unit cell dimensions	a = 10.7740(11) Å	□= 90°.
	b = 13.7709(13) Å	□= 90°.
	c = 17.0575(17) Å	□ = 90°.
Volume	2530.8(4) $Å^3$	
Z	4	
Density (calculated)	1.735 Mg/m^3	
Absorption coefficient	5.469 mm^{-1}	
F(000)	1304	
Crystal size	0.120 x 0.110 x 0.080 mm^3	
Theta range for data collection	2.236 to 28.288°.	
Index ranges	-14<=h<=14, -18<=k<=16, -22<=l<=22	
Reflections collected	24227	
Independent reflections	6250 [R(int) = 0.0572]	
Completeness to theta = 25.242°	99.6 %	
Absorption correction	Semi-empirical from equivalents	
Max. and min. transmission	0.2627 and 0.1390	
Refinement method	Full-matrix least-squares on F^2	
Data / restraints / parameters	6250 / 0 / 301	
Goodness-of-fit on F^2	1.045	
Final R indices [I>2sigma(I)]	R1 = 0.0266, wR2 = 0.0637	
R indices (all data)	R1 = 0.0283, wR2 = 0.0649	
Absolute structure parameter	0.053(9)	
Extinction coefficient	n/a	
Largest diff. peak and hole	0.746 and -1.453 e.$Å^{-3}$	

Supplementary Table 29. Atomic coordinates (x 10^4) and equivalent isotropic displacement parameters ($Å^2$x 10^3)
for 20200115LI_SZZ_LZX5_84_1_0m_a. U(eq) is defined as one third of the trace of the orthogonalized U^{ij} tensor.

	x	y	z	U(eq)
Ir(1)	6311(1)	6037(1)	4598(1)	24(1)
P(1)	5848(1)	4893(1)	5485(1)	23(1)

Cl(1)	8078(2)	5122(1)	4280(1)	43(1)
O(1)	5395(4)	3868(3)	5110(2)	24(1)
O(2)	4661(4)	5188(3)	6033(2)	26(1)
C(25)	5219(6)	7144(4)	5099(4)	34(1)
C(1)	4343(5)	3920(4)	4633(3)	29(1)
N(1)	6964(5)	4485(4)	6049(3)	31(1)
C(16)	4351(5)	4516(4)	6634(3)	26(1)
C(21)	6384(7)	6663(5)	3402(3)	39(1)
C(17)	3576(5)	3748(4)	6481(3)	26(1)
C(18)	4496(6)	6624(5)	4553(4)	39(1)
C(2)	4503(7)	4098(5)	3828(4)	40(2)
C(22)	6994(6)	7308(4)	3888(4)	35(1)
C(27)	7905(7)	5155(5)	6322(5)	45(2)
C(7)	2795(5)	3501(4)	5772(4)	27(1)
C(15)	4911(6)	4610(5)	7362(4)	37(2)
C(8)	1474(5)	3931(5)	5806(4)	40(1)
C(6)	3180(6)	3802(4)	4956(3)	27(1)
C(13)	4007(7)	3114(5)	7782(4)	41(2)
C(12)	3448(6)	3019(4)	7052(4)	32(1)
C(3)	3450(9)	4180(5)	3368(4)	56(2)
C(10)	2720(6)	2378(4)	5851(4)	30(1)
C(14)	4722(7)	3914(6)	7932(4)	45(2)
C(5)	2138(6)	3928(5)	4480(4)	39(1)
C(19)	4165(7)	7024(6)	3744(5)	53(2)
C(24)	5782(8)	8130(5)	4976(4)	45(2)
C(23)	6422(8)	8250(4)	4183(4)	44(2)
C(26)	7105(7)	3478(5)	6315(4)	42(2)
C(20)	5089(9)	6773(6)	3108(4)	55(2)
C(4)	2268(8)	4125(5)	3690(5)	52(2)
C(11)	2688(6)	2200(5)	6730(4)	36(1)
C(9)	971(6)	3840(5)	4962(5)	46(2)

Supplementary Table 30. Bond lengths [Å] and angles [°] for 20200115LI_SZZ_LZX5_84_1_0m_a.

Ir(1)-C(25)	2.107(6)	Ir(1)-C(18)	2.118(6)

Ir(1)-C(21)	2.215(6)	C(15)-H(15)	0.9300
Ir(1)-P(1)	2.2407(14)	C(8)-C(9)	1.544(11)
Ir(1)-C(22)	2.252(6)	C(8)-H(8A)	0.9700
Ir(1)-Cl(1)	2.3461(15)	C(8)-H(8B)	0.9700
P(1)-O(1)	1.625(4)	C(6)-C(5)	1.396(8)
P(1)-O(2)	1.635(4)	C(13)-C(14)	1.369(10)
P(1)-N(1)	1.641(5)	C(13)-C(12)	1.390(9)
O(1)-C(1)	1.398(7)	C(13)-H(13)	0.9300
O(2)-C(16)	1.421(7)	C(12)-C(11)	1.498(8)
C(25)-C(18)	1.410(9)	C(3)-C(4)	1.389(12)
C(25)-C(24)	1.502(10)	C(3)-H(3)	0.9300
C(25)-H(25)	0.9300	C(10)-C(11)	1.521(9)
C(1)-C(6)	1.378(9)	C(10)-H(10A)	0.9700
C(1)-C(2)	1.404(8)	C(10)-H(10B)	0.9700
N(1)-C(27)	1.447(8)	C(14)-H(14)	0.9300
N(1)-C(26)	1.467(8)	C(5)-C(4)	1.383(10)
C(16)-C(17)	1.373(7)	C(5)-C(9)	1.507(11)
C(16)-C(15)	1.386(9)	C(19)-C(20)	1.512(12)
C(21)-C(22)	1.382(9)	C(19)-H(19A)	0.9700
C(21)-C(20)	1.492(11)	C(19)-H(19B)	0.9700
C(21)-H(21)	0.9300	C(24)-C(23)	1.527(11)
C(17)-C(12)	1.404(8)	C(24)-H(24A)	0.9700
C(17)-C(7)	1.512(8)	C(24)-H(24B)	0.9700
C(18)-C(19)	1.527(10)	C(23)-H(23A)	0.9700
C(18)-H(18)	0.9300	C(23)-H(23B)	0.9700
C(2)-C(3)	1.385(11)	C(26)-H(26A)	0.9600
C(2)-H(2)	0.9300	C(26)-H(26B)	0.9600
C(22)-C(23)	1.521(9)	C(26)-H(26C)	0.9600
C(22)-H(22)	0.9300	C(20)-H(20A)	0.9700
C(27)-H(27A)	0.9600	C(20)-H(20B)	0.9700
C(27)-H(27B)	0.9600	C(4)-H(4)	0.9300
C(27)-H(27C)	0.9600	C(11)-H(11A)	0.9700
C(7)-C(6)	1.509(8)	C(11)-H(11B)	0.9700
C(7)-C(8)	1.542(8)	C(9)-H(9A)	0.9700
C(7)-C(10)	1.556(8)	C(9)-H(9B)	0.9700
C(15)-C(14)	1.381(10)		

C(25)-Ir(1)-C(18)	39.0(3)	C(17)-C(16)-O(2)	120.5(5)
C(25)-Ir(1)-C(21)	96.4(2)	C(15)-C(16)-O(2)	118.9(5)
C(18)-Ir(1)-C(21)	81.4(3)	C(22)-C(21)-C(20)	125.6(7)
C(25)-Ir(1)-P(1)	96.31(19)	C(22)-C(21)-Ir(1)	73.5(4)
C(18)-Ir(1)-P(1)	95.0(2)	C(20)-C(21)-Ir(1)	108.4(5)
C(21)-Ir(1)-P(1)	154.59(19)	C(22)-C(21)-H(21)	117.2
C(25)-Ir(1)-C(22)	80.7(3)	C(20)-C(21)-H(21)	117.2
C(18)-Ir(1)-C(22)	89.2(3)	Ir(1)-C(21)-H(21)	88.0
C(21)-Ir(1)-C(22)	36.0(2)	C(16)-C(17)-C(12)	118.6(5)
P(1)-Ir(1)-C(22)	169.18(18)	C(16)-C(17)-C(7)	131.6(5)
C(25)-Ir(1)-Cl(1)	159.25(19)	C(12)-C(17)-C(7)	109.9(5)
C(18)-Ir(1)-Cl(1)	161.1(2)	C(25)-C(18)-C(19)	122.9(6)
C(21)-Ir(1)-Cl(1)	88.1(2)	C(25)-C(18)-Ir(1)	70.1(3)
P(1)-Ir(1)-Cl(1)	87.66(5)	C(19)-C(18)-Ir(1)	112.7(5)
C(22)-Ir(1)-Cl(1)	91.62(17)	C(25)-C(18)-H(18)	118.5
O(1)-P(1)-O(2)	101.9(2)	C(19)-C(18)-H(18)	118.5
O(1)-P(1)-N(1)	98.8(2)	Ir(1)-C(18)-H(18)	87.3
O(2)-P(1)-N(1)	108.8(3)	C(3)-C(2)-C(1)	117.9(7)
O(1)-P(1)-Ir(1)	114.31(16)	C(3)-C(2)-H(2)	121.1
O(2)-P(1)-Ir(1)	112.71(16)	C(1)-C(2)-H(2)	121.1
N(1)-P(1)-Ir(1)	118.31(19)	C(21)-C(22)-C(23)	123.6(6)
C(1)-O(1)-P(1)	115.3(3)	C(21)-C(22)-Ir(1)	70.5(3)
C(16)-O(2)-P(1)	115.8(3)	C(23)-C(22)-Ir(1)	110.6(4)
C(18)-C(25)-C(24)	126.1(6)	C(21)-C(22)-H(22)	118.2
C(18)-C(25)-Ir(1)	70.9(3)	C(23)-C(22)-H(22)	118.2
C(24)-C(25)-Ir(1)	111.8(5)	Ir(1)-C(22)-H(22)	88.9
C(18)-C(25)-H(25)	116.9	N(1)-C(27)-H(27A)	109.5
C(24)-C(25)-H(25)	116.9	N(1)-C(27)-H(27B)	109.5
Ir(1)-C(25)-H(25)	87.2	H(27A)-C(27)-H(27B)	109.5
C(6)-C(1)-O(1)	119.9(5)	N(1)-C(27)-H(27C)	109.5
C(6)-C(1)-C(2)	121.6(6)	H(27A)-C(27)-H(27C)	109.5
O(1)-C(1)-C(2)	118.6(5)	H(27B)-C(27)-H(27C)	109.5
C(27)-N(1)-C(26)	115.6(5)	C(6)-C(7)-C(17)	121.5(5)
C(27)-N(1)-P(1)	118.9(4)	C(6)-C(7)-C(8)	100.6(5)
C(26)-N(1)-P(1)	125.6(4)	C(17)-C(7)-C(8)	113.4(5)
C(17)-C(16)-C(15)	120.5(6)	C(6)-C(7)-C(10)	111.5(5)

C(17)-C(7)-C(10)	100.5(5)	C(20)-C(19)-H(19A)	108.7
C(8)-C(7)-C(10)	109.3(5)	C(18)-C(19)-H(19A)	108.7
C(14)-C(15)-C(16)	120.2(6)	C(20)-C(19)-H(19B)	108.7
C(14)-C(15)-H(15)	119.9	C(18)-C(19)-H(19B)	108.7
C(16)-C(15)-H(15)	119.9	H(19A)-C(19)-H(19B)	107.6
C(7)-C(8)-C(9)	104.9(5)	C(25)-C(24)-C(23)	113.8(6)
C(7)-C(8)-H(8A)	110.8	C(25)-C(24)-H(24A)	108.8
C(9)-C(8)-H(8A)	110.8	C(23)-C(24)-H(24A)	108.8
C(7)-C(8)-H(8B)	110.8	C(25)-C(24)-H(24B)	108.8
C(9)-C(8)-H(8B)	110.8	C(23)-C(24)-H(24B)	108.8
H(8A)-C(8)-H(8B)	108.8	H(24A)-C(24)-H(24B)	107.7
C(1)-C(6)-C(5)	118.9(5)	C(22)-C(23)-C(24)	112.6(5)
C(1)-C(6)-C(7)	130.6(5)	C(22)-C(23)-H(23A)	109.1
C(5)-C(6)-C(7)	110.4(5)	C(24)-C(23)-H(23A)	109.1
C(14)-C(13)-C(12)	119.1(6)	C(22)-C(23)-H(23B)	109.1
C(14)-C(13)-H(13)	120.4	C(24)-C(23)-H(23B)	109.1
C(12)-C(13)-H(13)	120.4	H(23A)-C(23)-H(23B)	107.8
C(13)-C(12)-C(17)	120.8(6)	N(1)-C(26)-H(26A)	109.5
C(13)-C(12)-C(11)	129.4(6)	N(1)-C(26)-H(26B)	109.5
C(17)-C(12)-C(11)	109.8(5)	H(26A)-C(26)-H(26B)	109.5
C(2)-C(3)-C(4)	121.5(7)	N(1)-C(26)-H(26C)	109.5
C(2)-C(3)-H(3)	119.3	H(26A)-C(26)-H(26C)	109.5
C(4)-C(3)-H(3)	119.3	H(26B)-C(26)-H(26C)	109.5
C(11)-C(10)-C(7)	104.3(5)	C(21)-C(20)-C(19)	113.4(6)
C(11)-C(10)-H(10A)	110.9	C(21)-C(20)-H(20A)	108.9
C(7)-C(10)-H(10A)	110.9	C(19)-C(20)-H(20A)	108.9
C(11)-C(10)-H(10B)	110.9	C(21)-C(20)-H(20B)	108.9
C(7)-C(10)-H(10B)	110.9	C(19)-C(20)-H(20B)	108.9
H(10A)-C(10)-H(10B)	108.9	H(20A)-C(20)-H(20B)	107.7
C(13)-C(14)-C(15)	120.6(6)	C(5)-C(4)-C(3)	119.3(7)
C(13)-C(14)-H(14)	119.7	C(5)-C(4)-H(4)	120.3
C(15)-C(14)-H(14)	119.7	C(3)-C(4)-H(4)	120.3
C(4)-C(5)-C(6)	120.6(7)	C(12)-C(11)-C(10)	103.1(5)
C(4)-C(5)-C(9)	129.2(6)	C(12)-C(11)-H(11A)	111.1
C(6)-C(5)-C(9)	110.2(6)	C(10)-C(11)-H(11A)	111.1
C(20)-C(19)-C(18)	114.4(6)	C(12)-C(11)-H(11B)	111.1

C(10)-C(11)-H(11B)	111.1	C(8)-C(9)-H(9A)	111.4
H(11A)-C(11)-H(11B)	109.1	C(5)-C(9)-H(9B)	111.4
C(5)-C(9)-C(8)	102.0(5)	C(8)-C(9)-H(9B)	111.4
C(5)-C(9)-H(9A)	111.4	H(9A)-C(9)-H(9B)	109.2

Symmetry transformations used to generate equivalent atoms:

Supplementary Table 31. Anisotropic displacement parameters ($Å^2 x 10^3$) for 20200115LI_SZZ_LZX5_84_1_0m_a. The anisotropic displacement factor exponent takes the form: -2□^{2}[$h^2 a^{*2}U^{11}$ + ... + 2 h k a* b* U^{12}]

	U^{11}	U^{22}	U^{33}	U^{23}	U^{13}	U^{12}
Ir(1)	23(1)	23(1)	26(1)	0(1)	2(1)	2(1)
P(1)	21(1)	24(1)	25(1)	-2(1)	1(1)	-1(1)
Cl(1)	31(1)	32(1)	66(1)	9(1)	20(1)	8(1)
O(1)	24(2)	26(2)	24(2)	-3(2)	0(2)	-2(2)
O(2)	25(2)	24(2)	30(2)	-6(2)	6(2)	-1(2)
C(25)	39(3)	31(3)	32(3)	0(2)	-3(3)	17(3)
C(1)	40(3)	23(2)	24(3)	-4(3)	-8(2)	-2(2)
N(1)	25(2)	39(3)	29(3)	4(2)	-7(2)	-5(2)
C(16)	22(3)	33(3)	25(3)	-7(2)	5(2)	-2(2)
C(21)	55(4)	38(3)	26(3)	10(2)	3(3)	5(3)
C(17)	23(3)	28(3)	28(3)	-4(2)	4(2)	-1(2)
C(18)	31(3)	40(3)	44(4)	1(3)	-3(3)	13(2)
C(2)	57(4)	38(4)	25(3)	-3(3)	-2(3)	-8(3)
C(22)	39(3)	29(3)	38(4)	6(2)	1(3)	2(2)
C(27)	35(4)	51(4)	50(5)	2(3)	-20(3)	-11(3)
C(7)	23(3)	24(3)	35(3)	-4(2)	-4(2)	-2(2)
C(15)	33(3)	47(4)	30(3)	-13(3)	5(3)	-11(3)
C(8)	24(3)	30(3)	65(4)	-8(3)	-1(3)	1(3)
C(6)	30(3)	22(3)	28(3)	-1(2)	-8(2)	0(2)
C(13)	43(4)	52(4)	29(3)	4(3)	5(3)	1(3)
C(12)	31(3)	34(3)	32(3)	0(2)	6(2)	-3(2)
C(3)	93(7)	43(4)	33(4)	4(3)	-23(4)	-2(4)
C(10)	24(3)	27(3)	37(3)	-4(2)	1(2)	-5(2)
C(14)	42(4)	68(5)	24(3)	-8(3)	0(3)	-11(4)

C(5)	37(3)	31(3)	48(4)	-3(3)	-19(3)	-4(3)
C(19)	43(4)	61(5)	54(5)	15(4)	-19(4)	11(3)
C(24)	60(5)	34(3)	41(4)	-3(3)	-3(3)	14(3)
C(23)	58(5)	28(3)	46(4)	7(3)	-6(4)	8(3)
C(26)	47(4)	46(4)	32(4)	2(3)	-13(3)	11(3)
C(20)	77(6)	58(5)	31(4)	8(3)	-23(4)	-8(4)
C(4)	62(5)	42(4)	53(5)	11(3)	-33(4)	-4(3)
C(11)	37(3)	32(3)	37(4)	1(3)	5(3)	-6(3)
C(9)	30(3)	38(4)	70(5)	2(3)	-17(3)	6(3)

Supplementary Table 32. Hydrogen coordinates (x 10^4) and isotropic displacement parameters (Å^{2}x 10 ^{3})
for 20200115LI_SZZ_LZX5_84_1_0m_a.

	x	y	z	U(eq)
H(25)	5361	6848	5581	41
H(21)	6819	6112	3246	47
H(18)	4207	6011	4691	46
H(2)	5292	4160	3612	48
H(22)	7799	7159	4044	42
H(27A)	7762	5304	6864	68
H(27B)	7867	5741	6018	68
H(27C)	8709	4864	6264	68
H(15)	5414	5143	7466	44
H(8A)	1497	4605	5969	48
H(8B)	960	3568	6170	48
H(13)	3896	2640	8164	50
H(3)	3537	4274	2831	67
H(10A)	1976	2129	5601	36
H(10B)	3439	2070	5615	36
H(14)	5084	3990	8423	54
H(19A)	3358	6774	3594	63
H(19B)	4099	7725	3779	63
H(24A)	6384	8250	5388	54

H(24B)	5135	8617	5023	54
H(23A)	5820	8479	3803	53
H(23B)	7067	8738	4229	53
H(26A)	7826	3196	6074	63
H(26B)	6383	3111	6170	63
H(26C)	7200	3466	6875	63
H(20A)	4835	6171	2859	66
H(20B)	5073	7279	2712	66
H(4)	1572	4220	3377	63
H(11A)	1845	2221	6929	43
H(11B)	3054	1577	6861	43
H(9A)	569	3218	4879	55
H(9B)	390	4357	4841	55

2.6 Copies of ^{1}H NMR, ^{13}C NMR, ^{19}F NMR and ^{31}P NMR Spectra

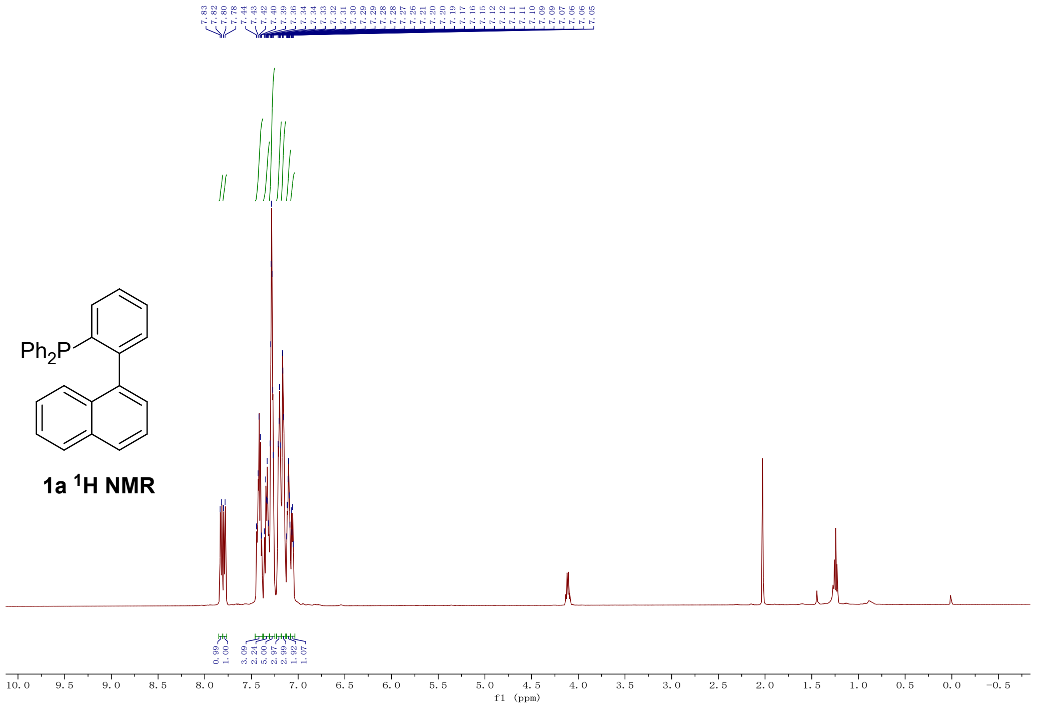

Supplementary Fig. 9. ^{1}H NMR spectra (500 MHz, $CDCl_3$, 25 °C) of **1a**

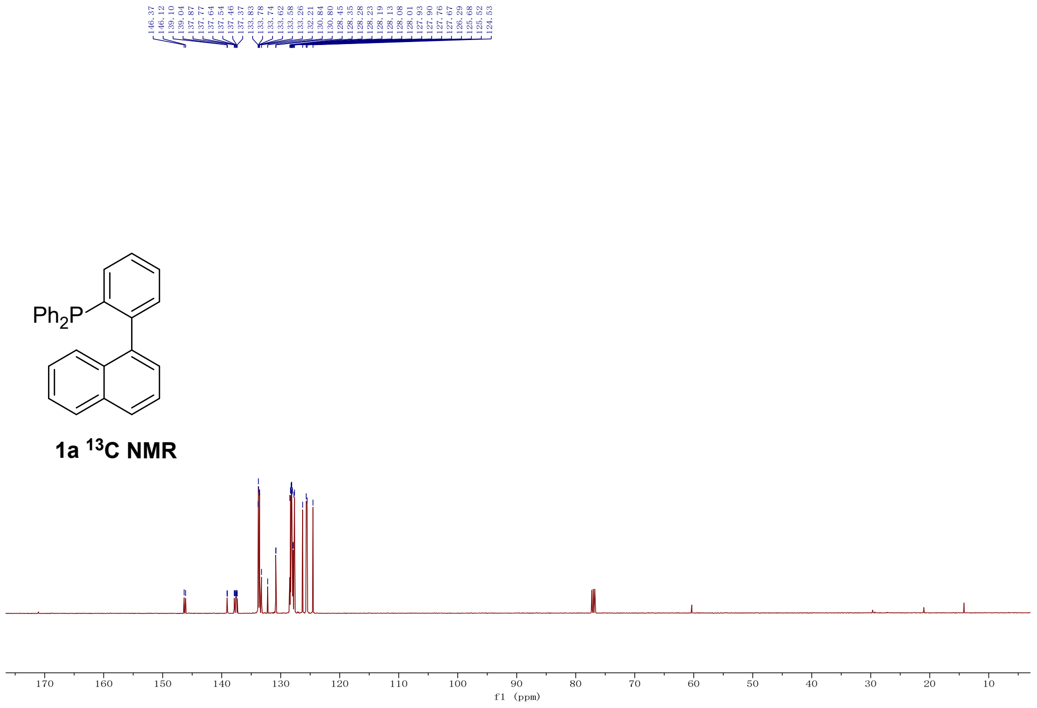

Supplementary Fig. 10. ^{13}C NMR spectra (126 MHz, $CDCl_3$, 25 °C) of **1a**

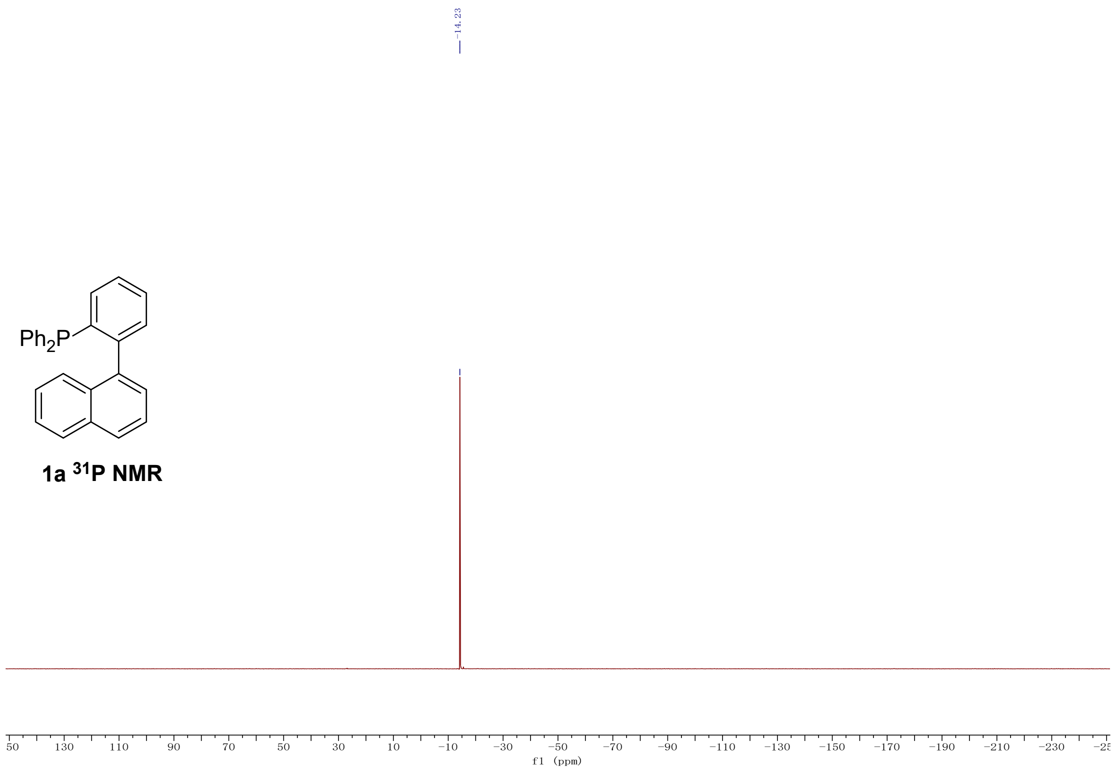

Supplementary Fig. 11. ^{31}P NMR spectra (202 MHz, $CDCl_3$, 25 °C) of **1a**

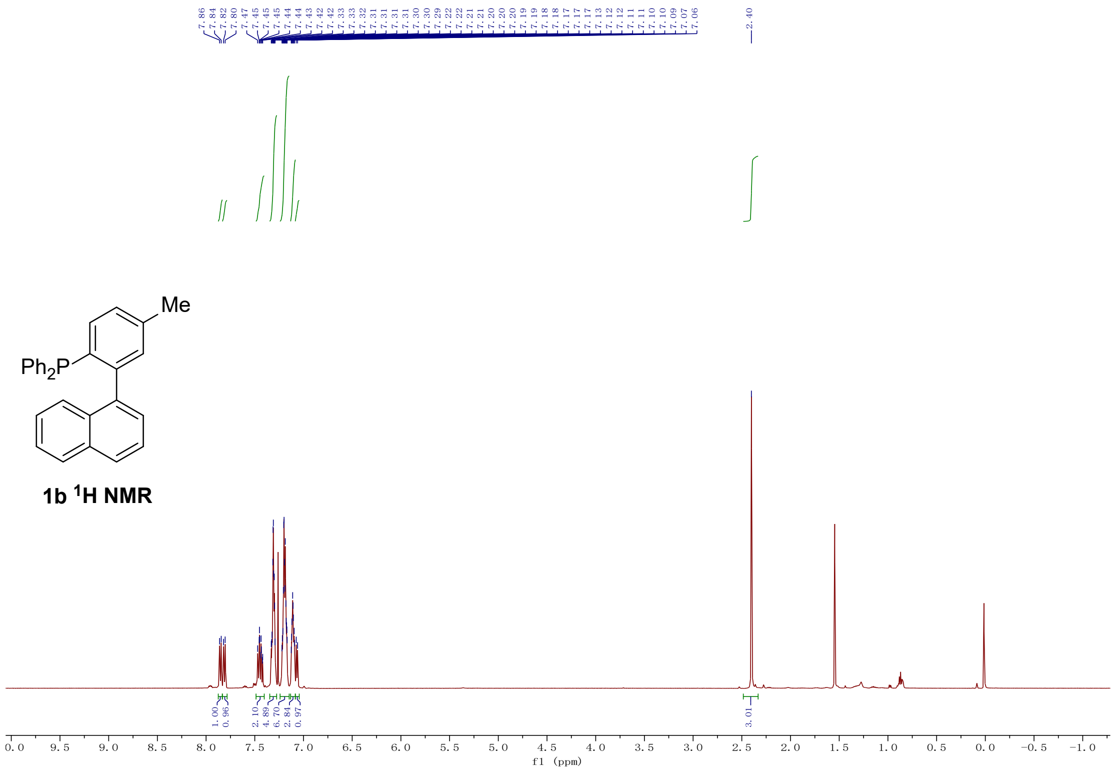

Supplementary Fig. 12. ^{1}H NMR spectra (500 MHz, $CDCl_3$, 25 °C) of **1b**

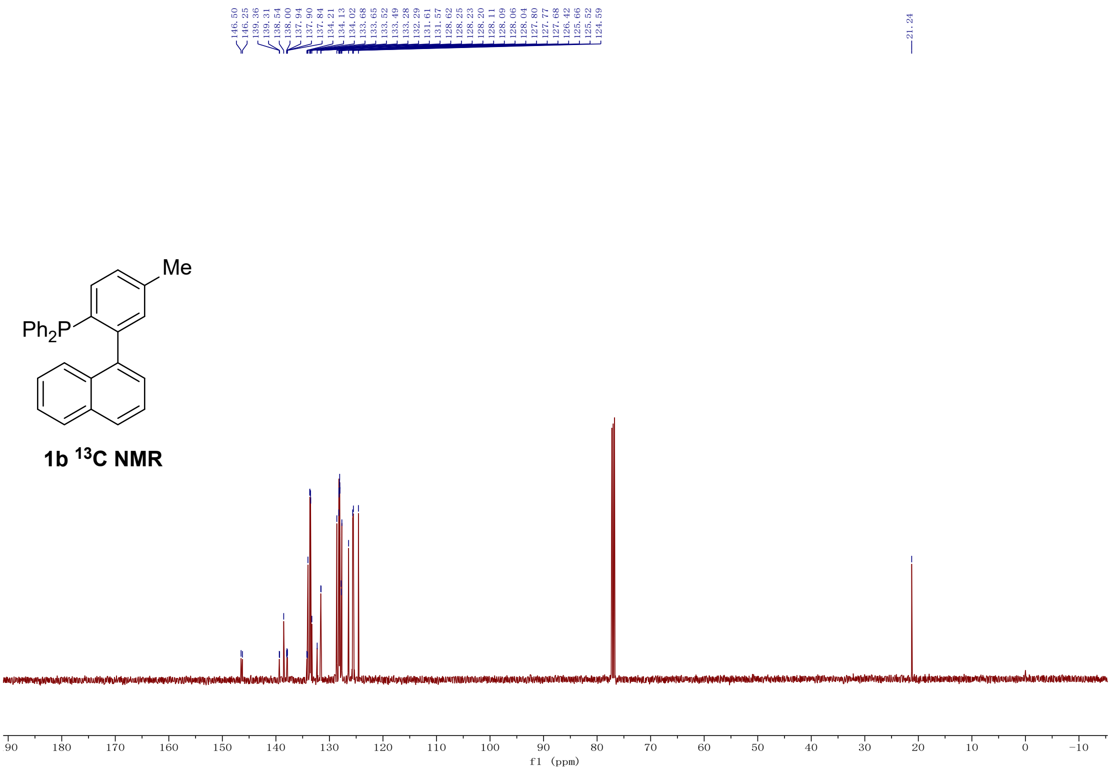

Supplementary Fig. 13. ^{13}C NMR spectra (126 MHz, $CDCl_3$, 25 ºC) of **1b**

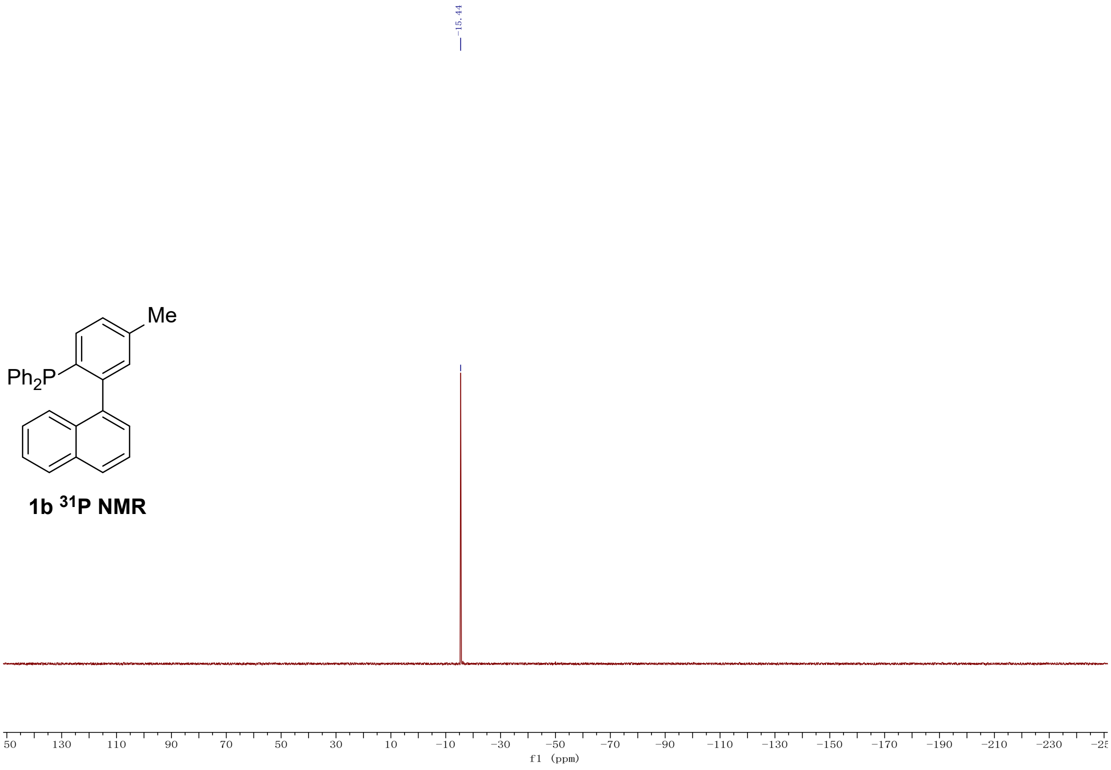

Supplementary Fig. 14. ^{31}P NMR spectra (202 MHz, $CDCl_3$, 25 ºC) of **1b**

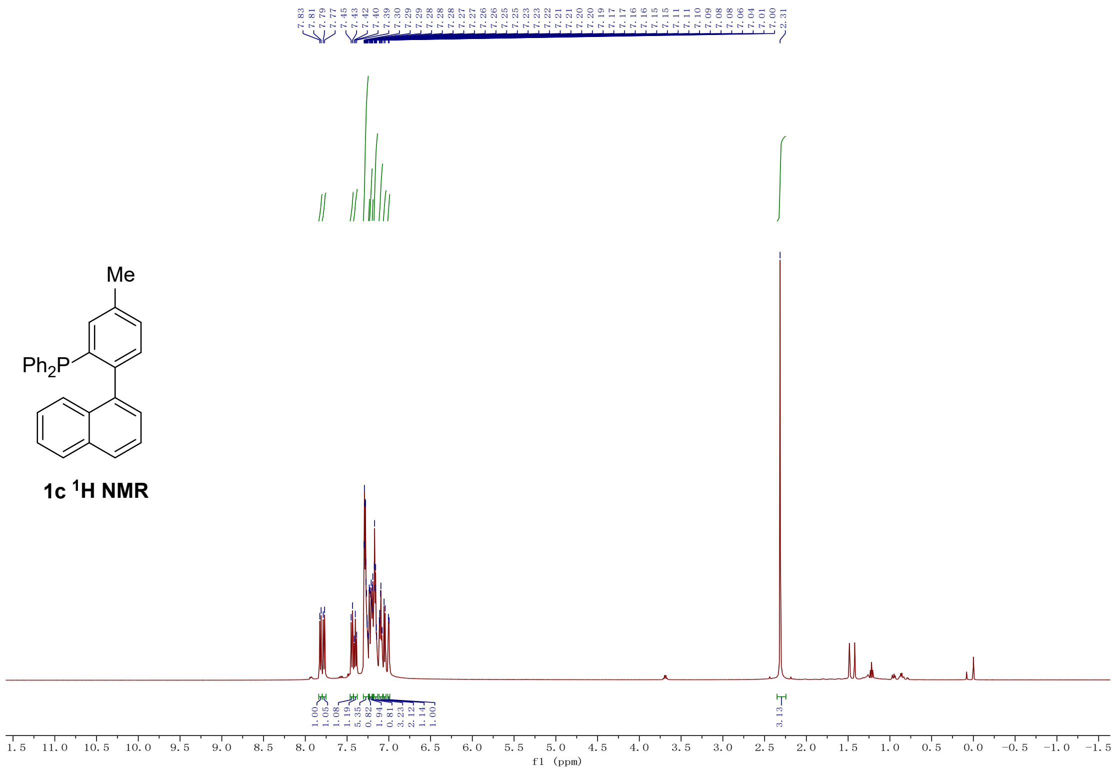

Supplementary Fig. 15. ^{1}H NMR spectra (500 MHz, $CDCl_3$, 25 ºC) of **1c**

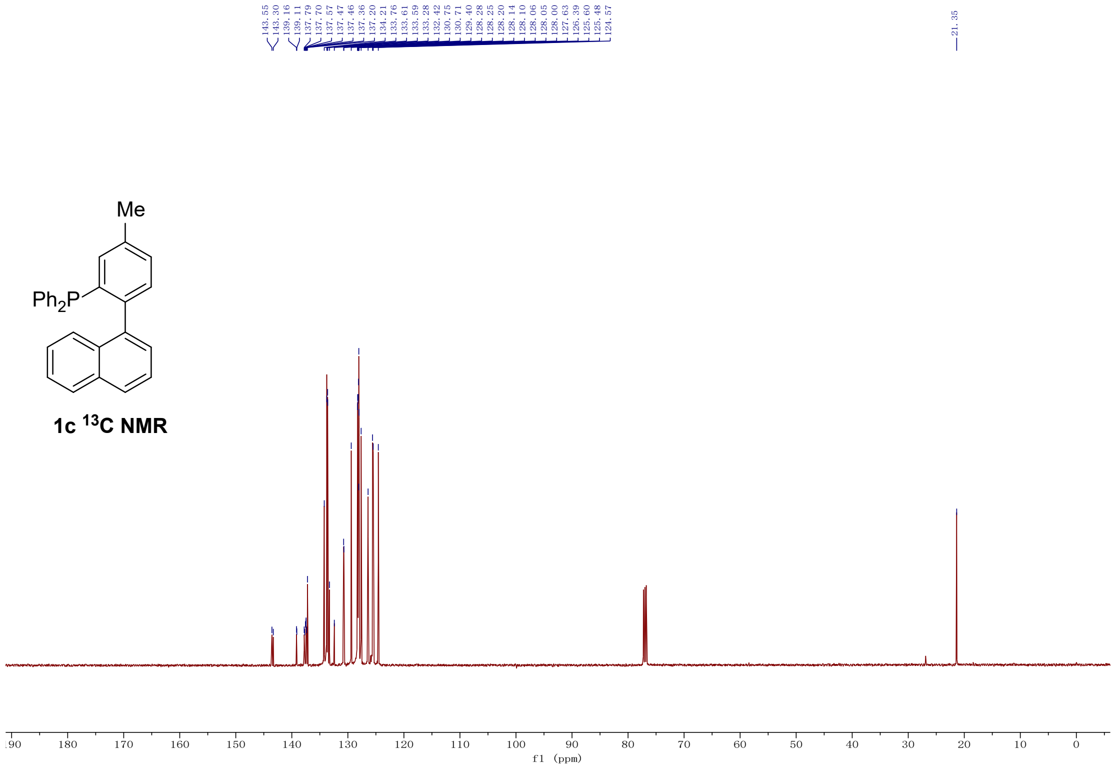

Supplementary Fig. 16. ^{13}C NMR spectra (126 MHz, $CDCl_3$, 25 ºC) of **1c**

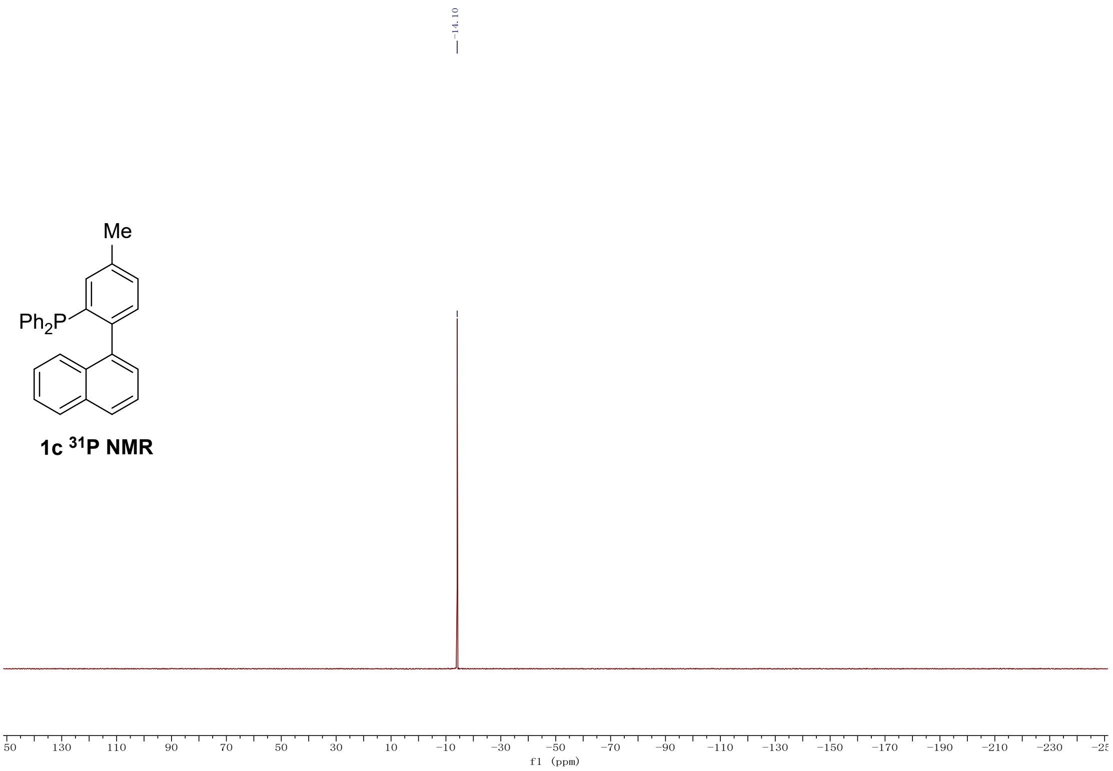

Supplementary Fig. 17. ^{31}P NMR spectra (202 MHz, CDCl$_3$, 25 ºC) of **1c**

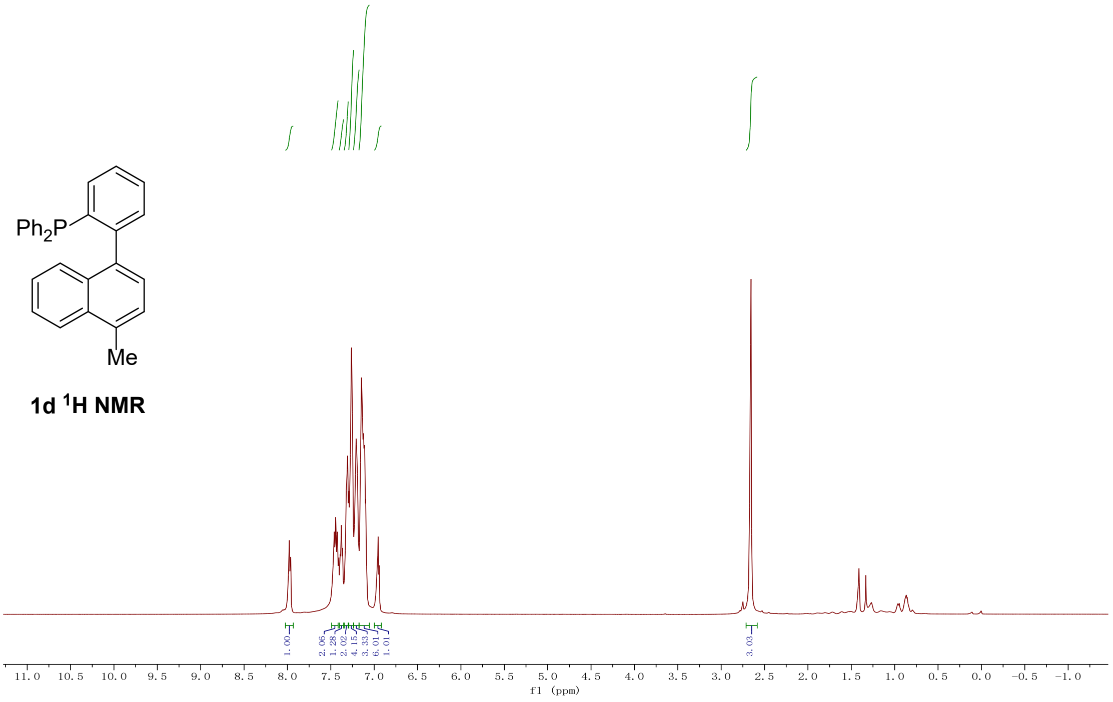

Supplementary Fig. 18. ^{1}H NMR spectra (500 MHz, CDCl$_3$, 25 ºC) of **1d**

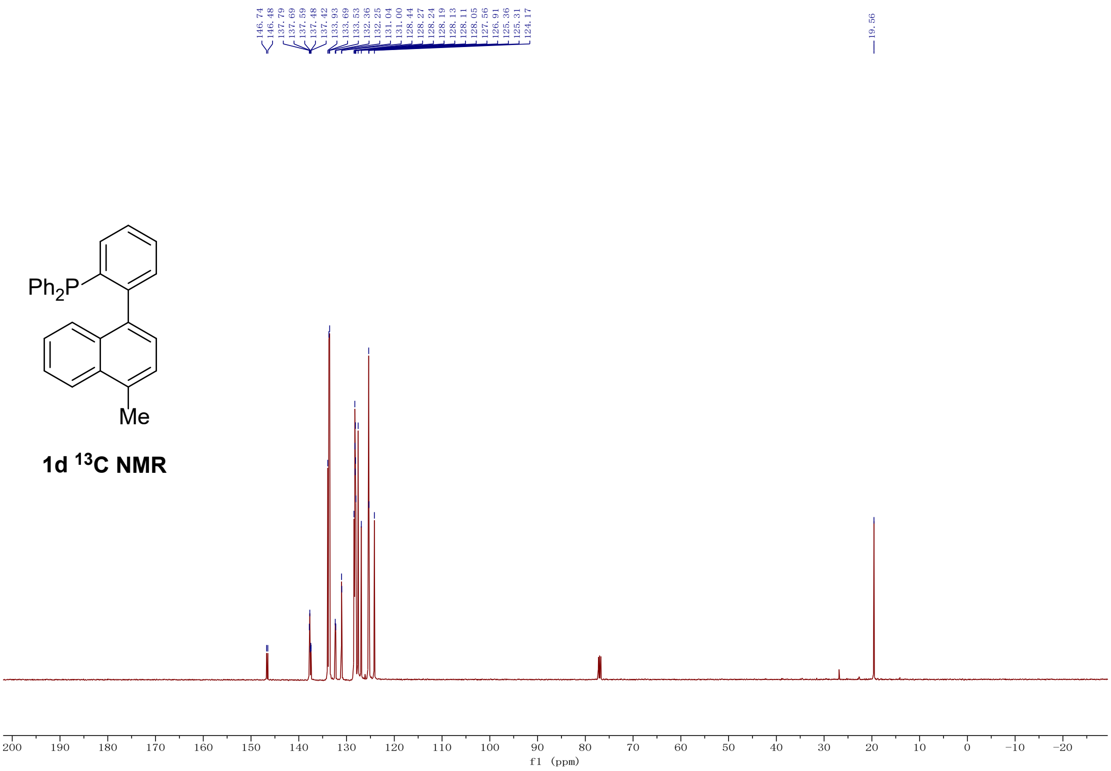

Supplementary Fig. 19. ^{13}C NMR spectra (126 MHz, $CDCl_3$, 25 °C) of **1d**

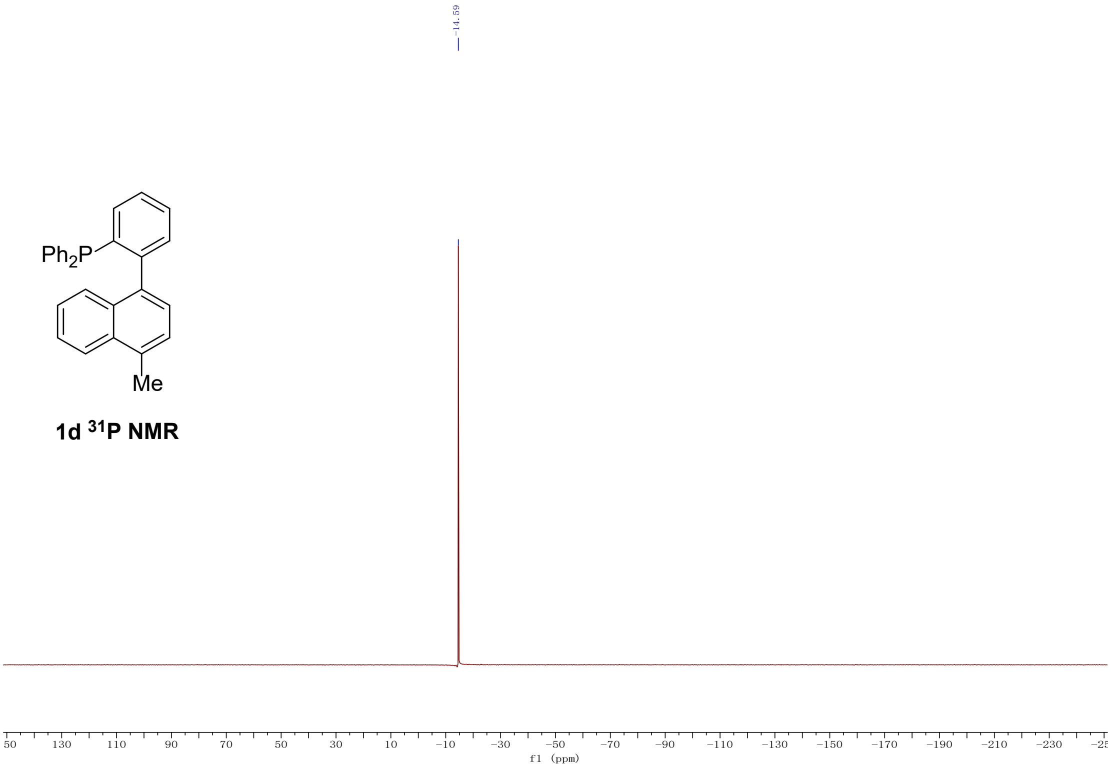

Supplementary Fig. 20. ^{31}P NMR spectra (202 MHz, $CDCl_3$, 25 °C) of **1d**

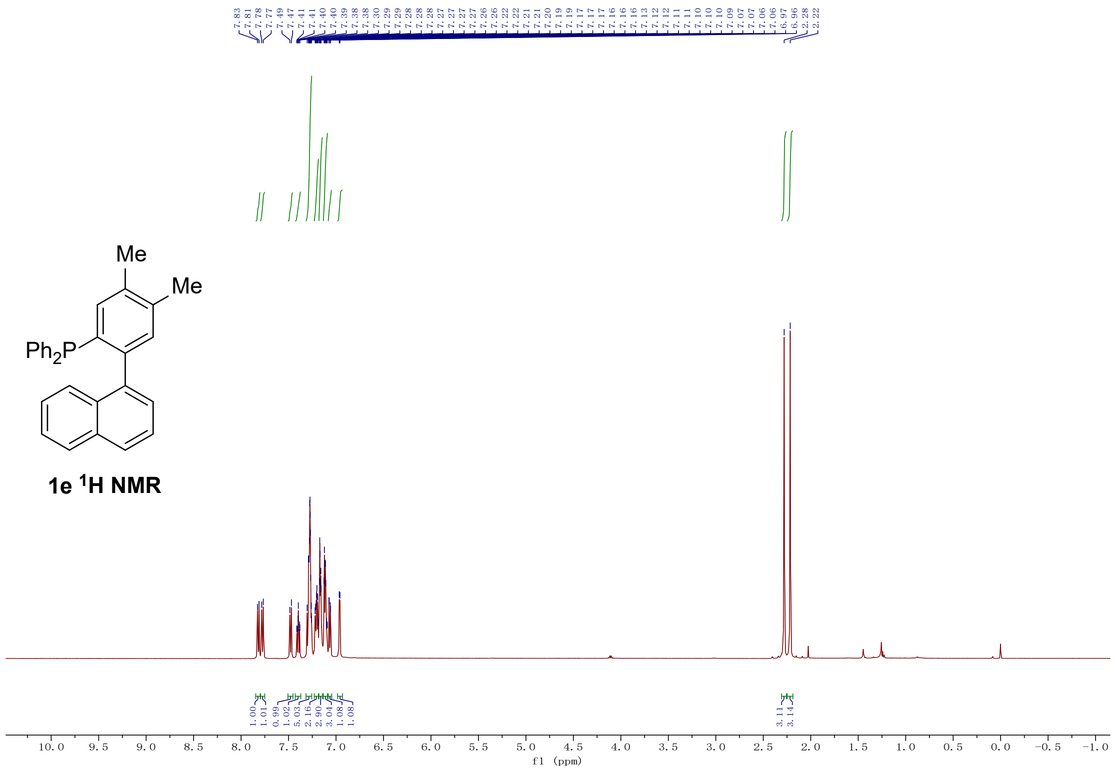

Supplementary Fig. 21. ^{1}H NMR spectra (500 MHz, $CDCl_3$, 25 ºC) of **1e**

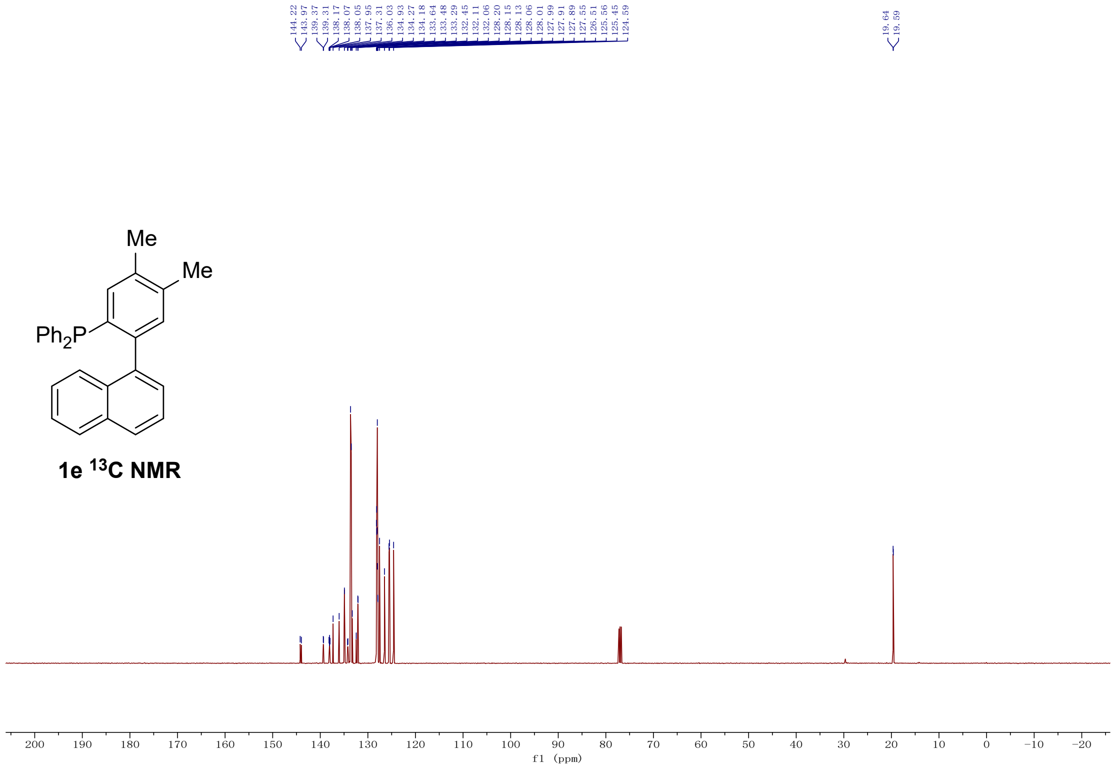

Supplementary Fig. 22. ^{13}C NMR spectra (126 MHz, $CDCl_3$, 25 ºC) of **1e**

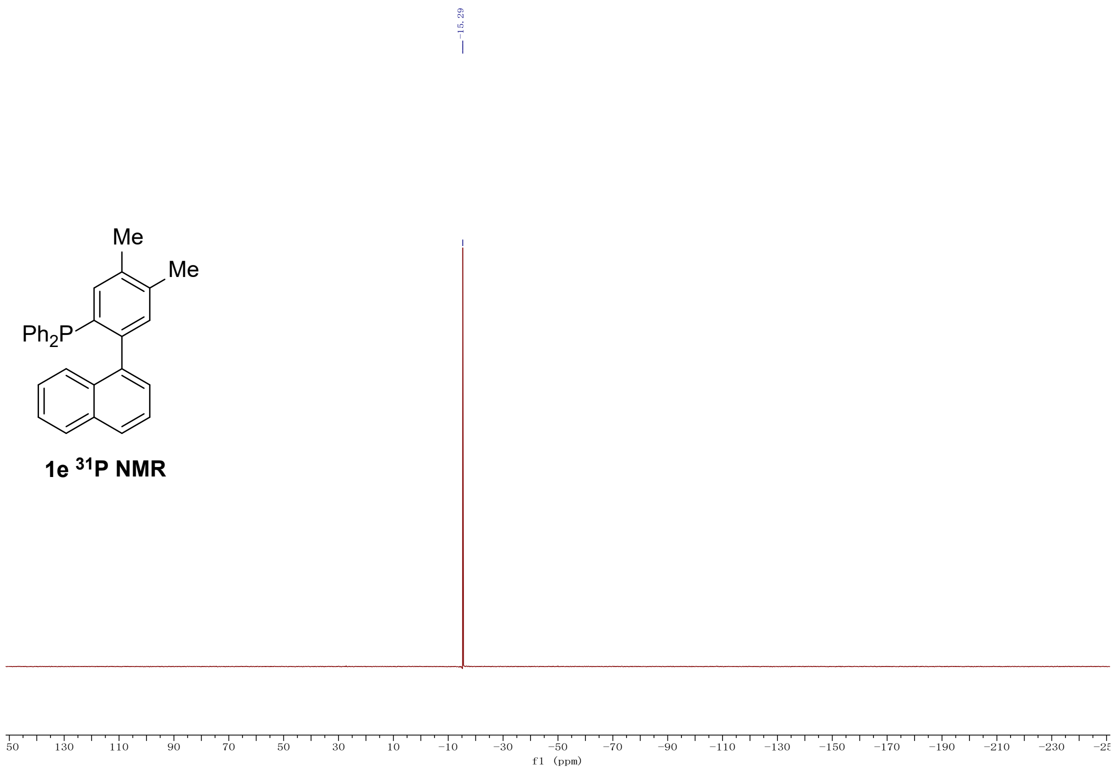

Supplementary Fig. 23. ^{31}P NMR spectra (202 MHz, $CDCl_3$, 25 °C) of **1e**

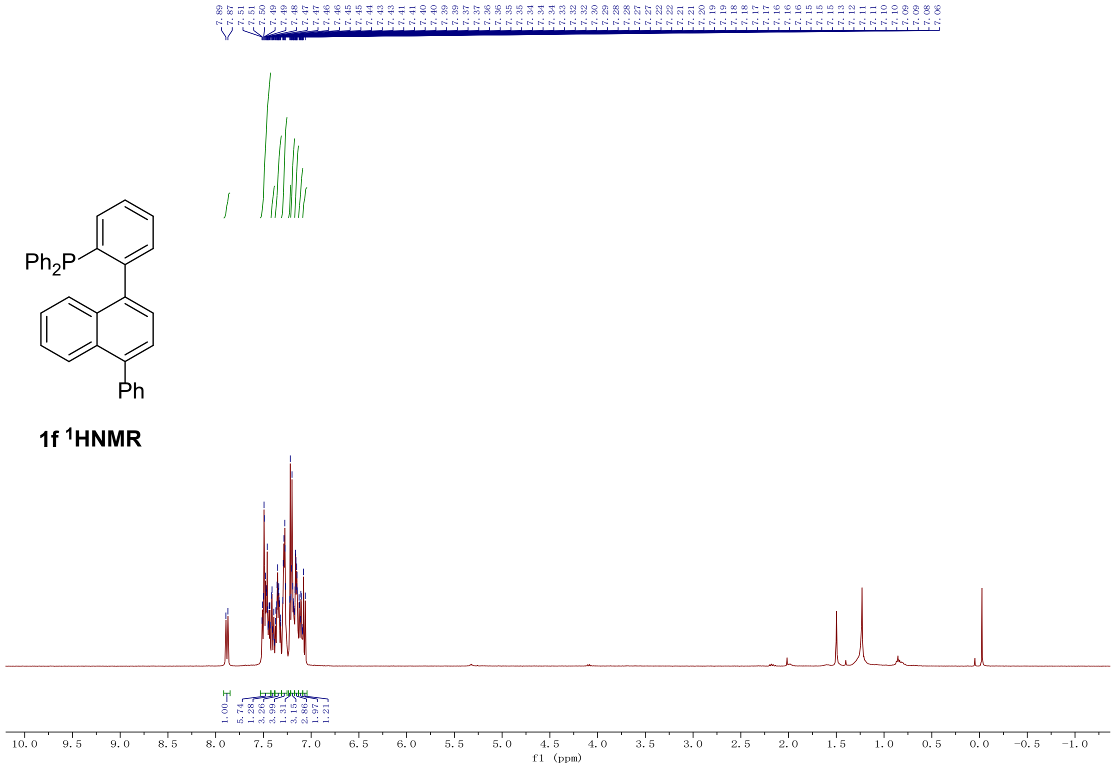

Supplementary Fig. 24. ^{1}H NMR spectra (400 MHz, $CDCl_3$, 25 °C) of **1f**

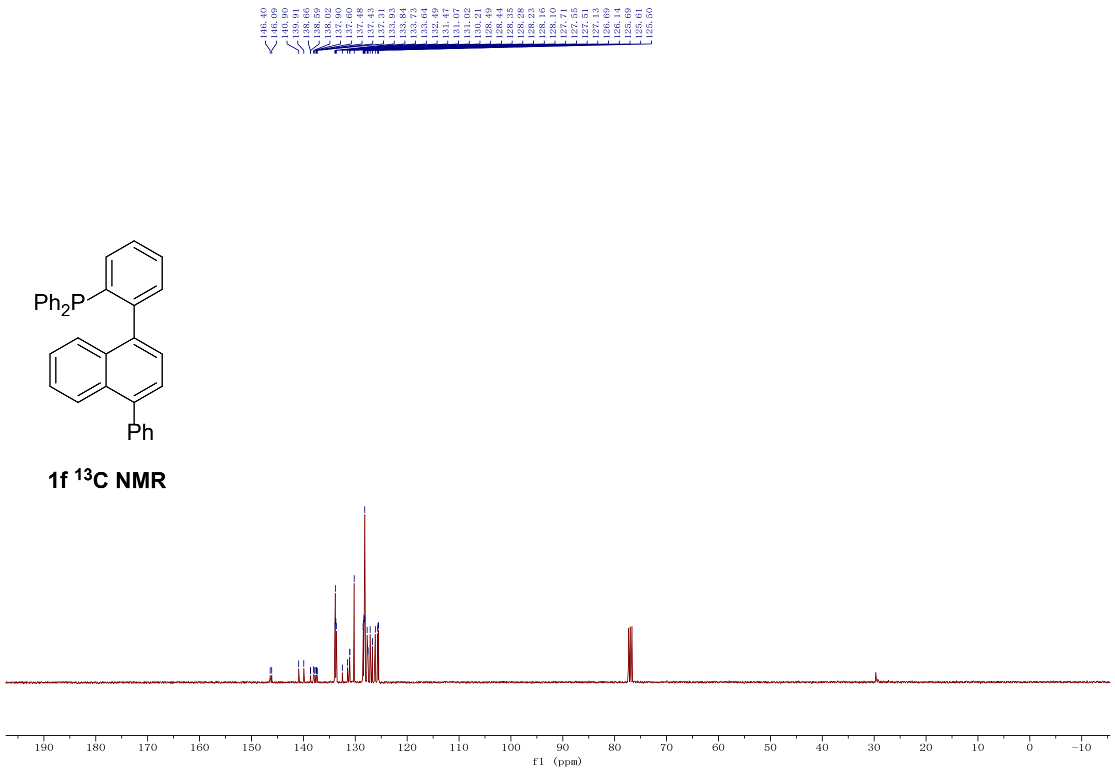

Supplementary Fig. 25. ^{13}C NMR spectra (101 MHz, $CDCl_3$, 25 °C) of **1f**

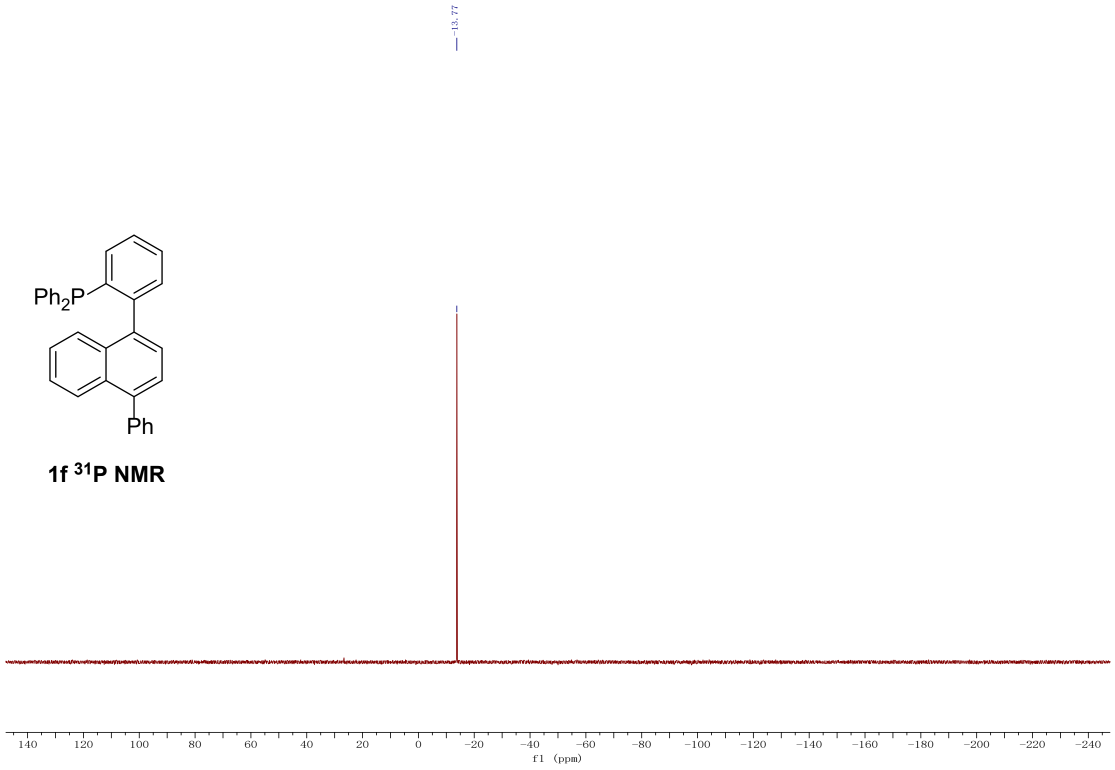

Supplementary Fig. 26. ^{31}P NMR spectra (162 MHz, $CDCl_3$, 25 °C) of **1f**

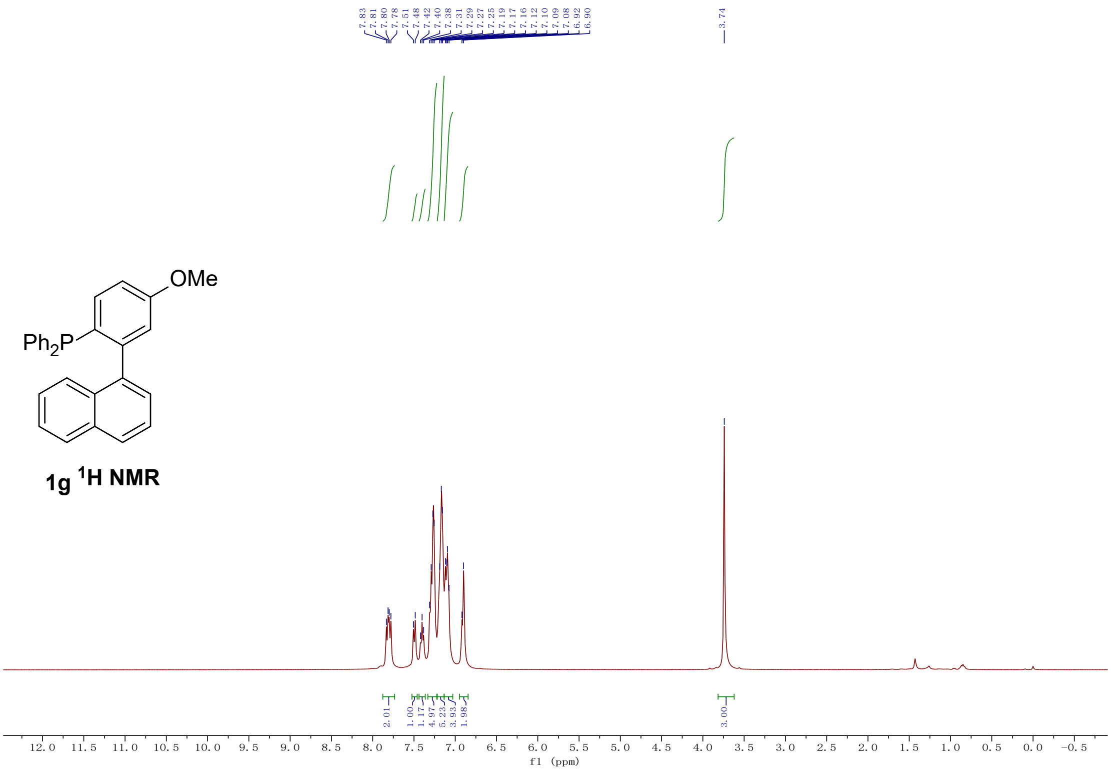

Supplementary Fig. 27. ^{1}H NMR spectra (400 MHz, $CDCl_3$, 25 ºC) of **1g**

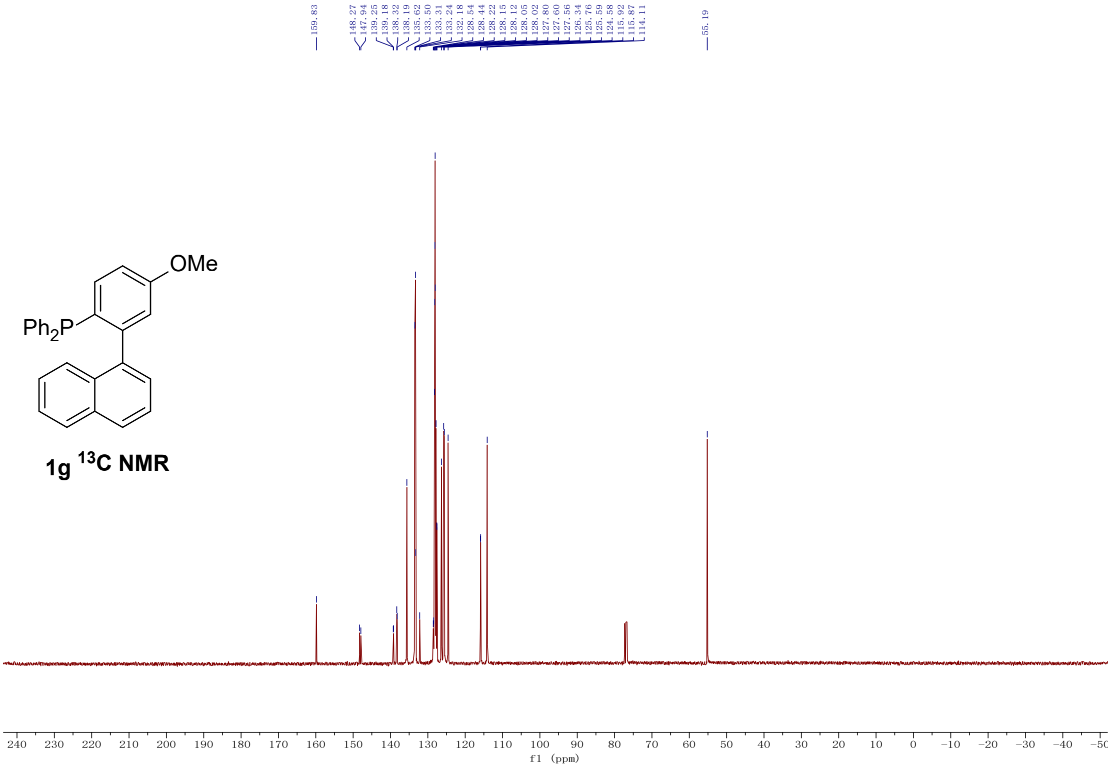

Supplementary Fig. 28. ^{13}C NMR spectra (101 MHz, $CDCl_3$, 25 ºC) of **1g**

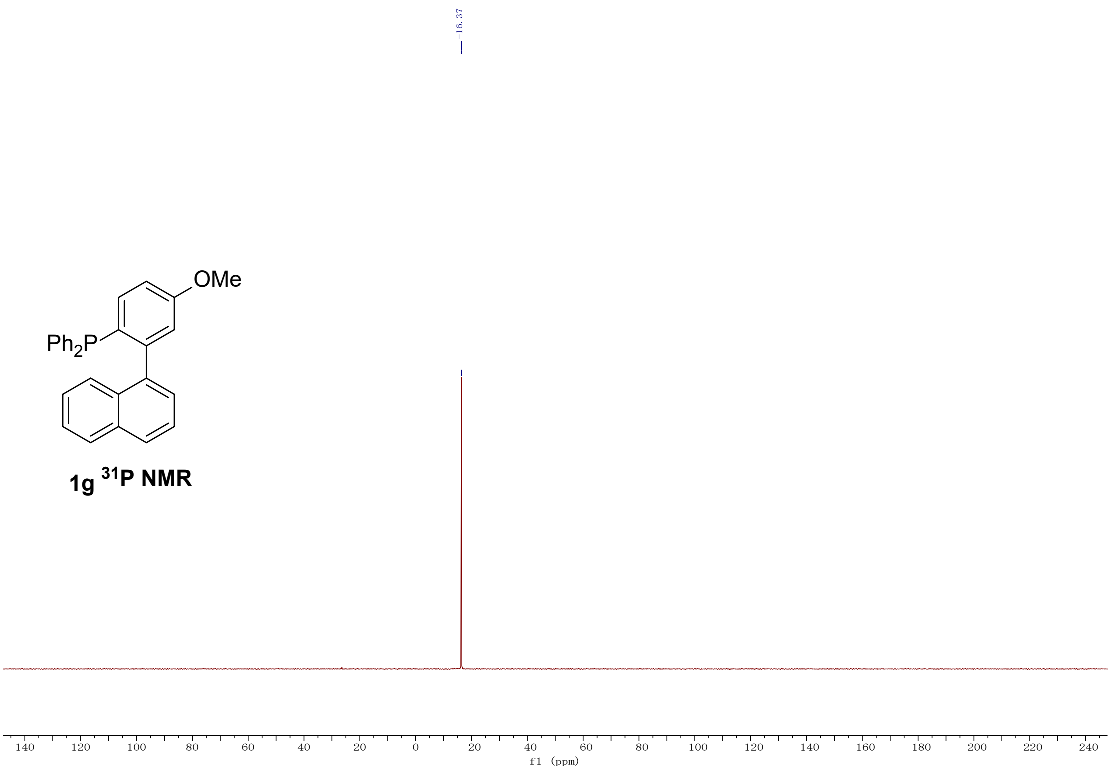

Supplementary Fig. 29. ^{31}P NMR spectra (162 MHz, $CDCl_3$, 25 ºC) of **1g**

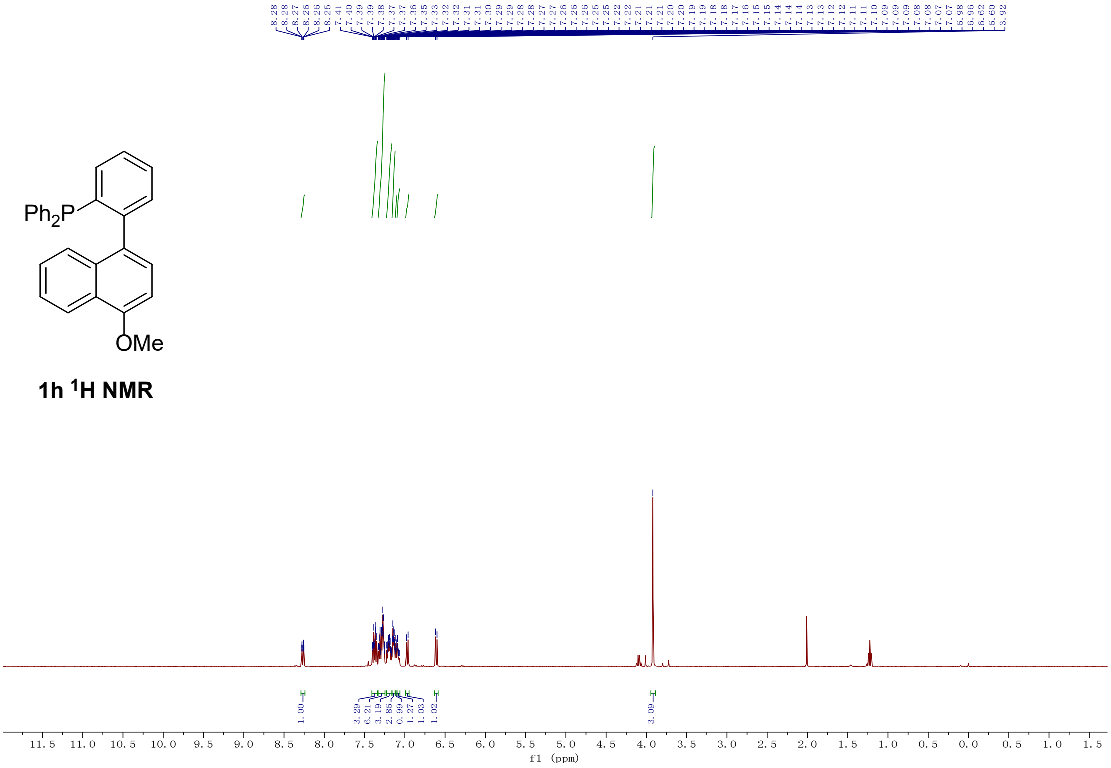

Supplementary Fig. 30. ^{1}H NMR spectra (400 MHz, $CDCl_3$, 25 ºC) of **1h**

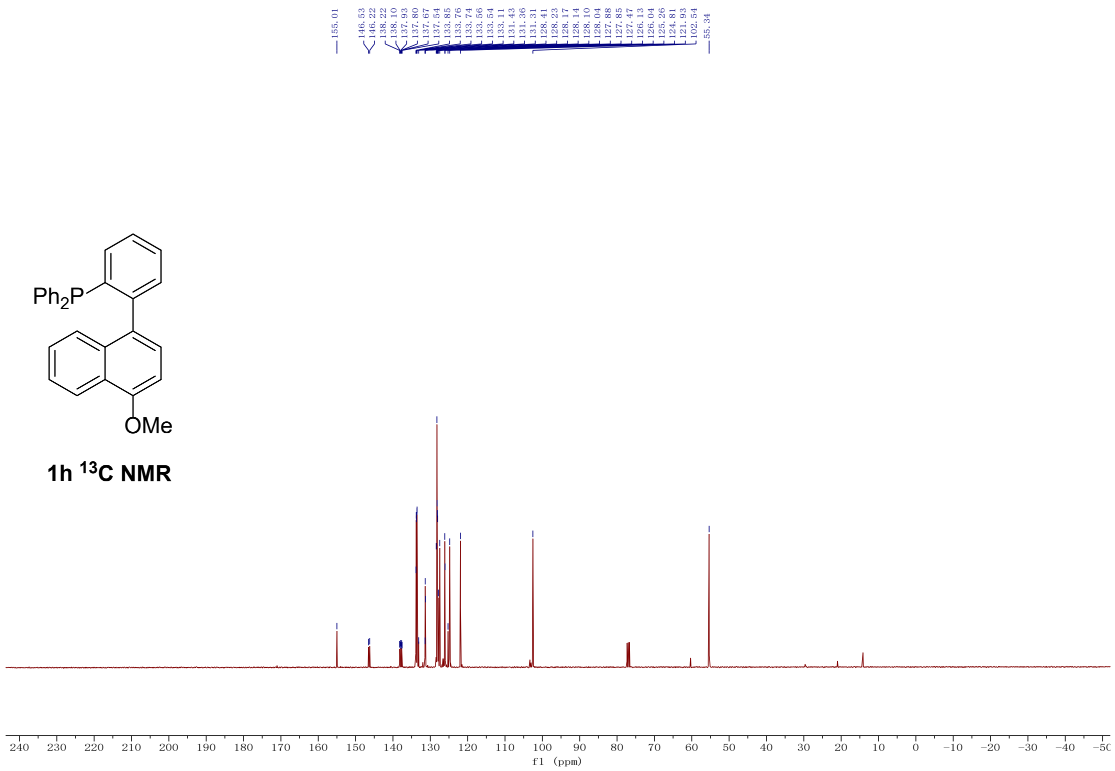

Supplementary Fig. 31. ^{13}C NMR spectra (101 MHz, $CDCl_3$, 25 ºC) of **1h**

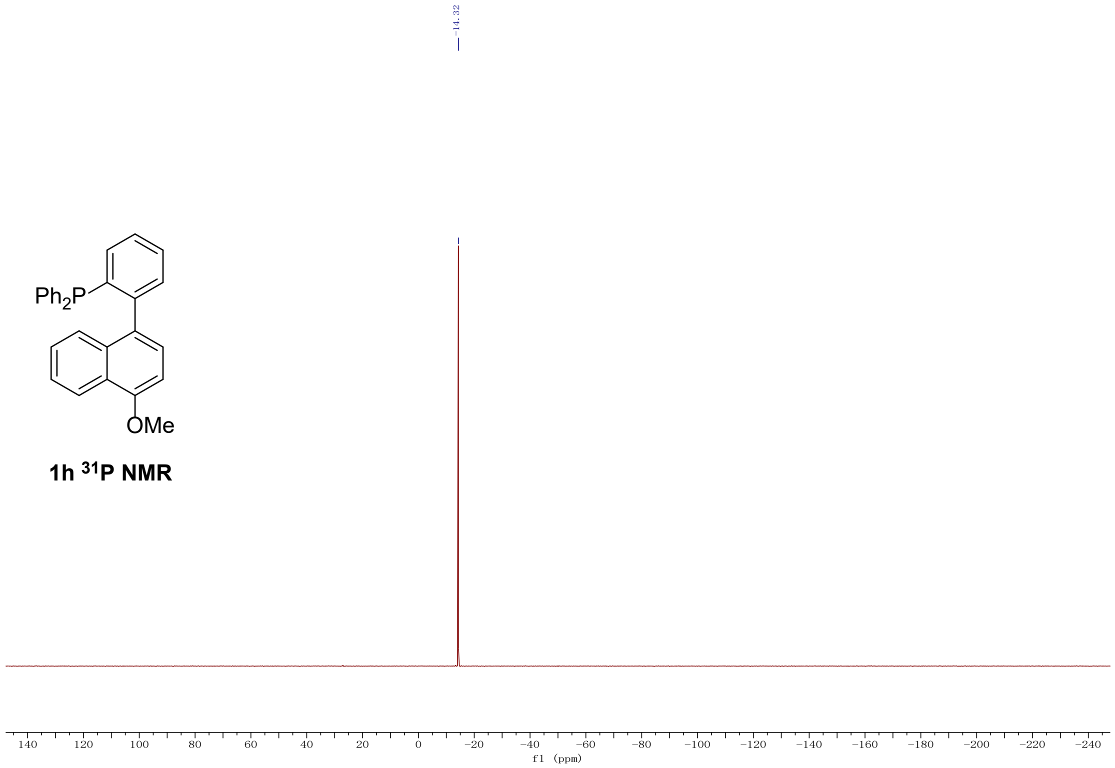

Supplementary Fig. 32. ^{31}P NMR spectra (162 MHz, $CDCl_3$, 25 ºC) of **1h**

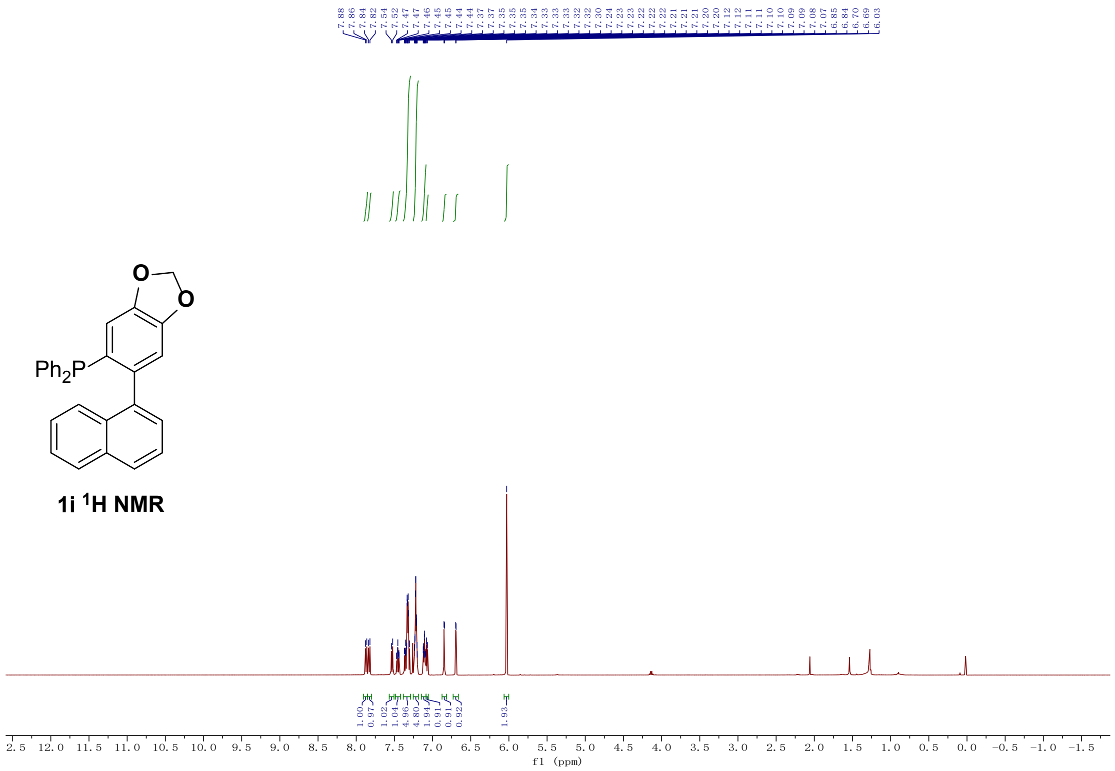

Supplementary Fig. 33. ^{1}H NMR spectra (400 MHz, $CDCl_3$, 25 ºC) of **1i**

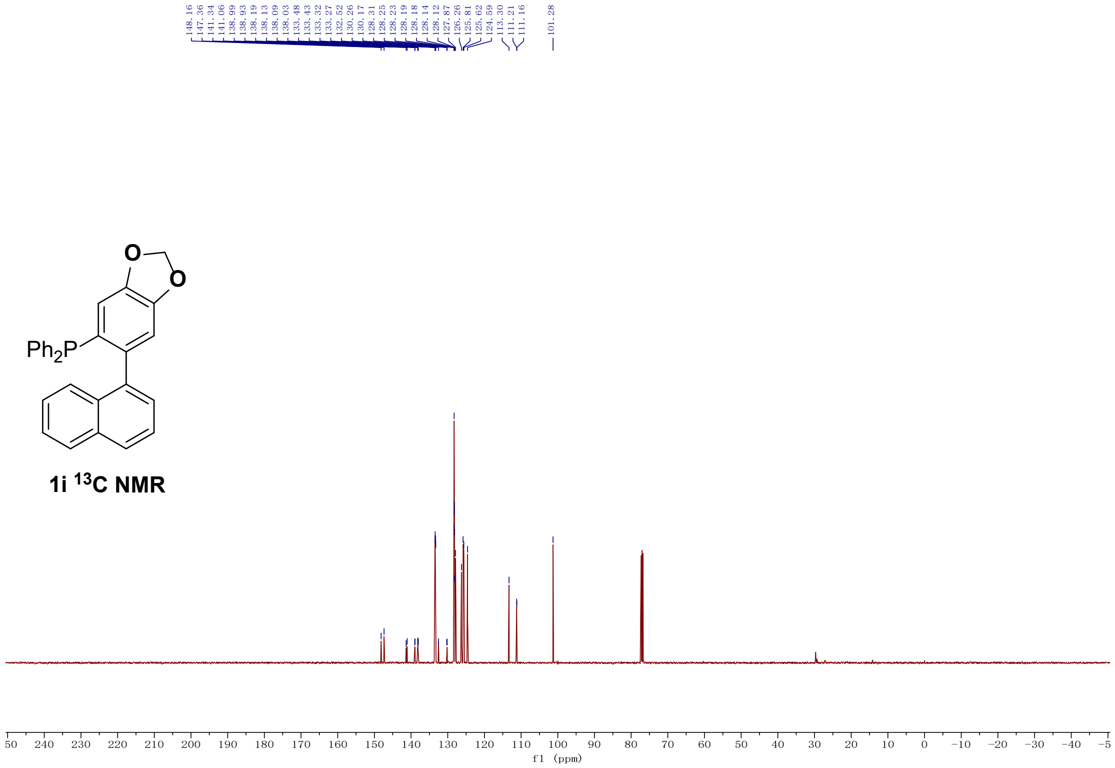

Supplementary Fig. 34. ^{13}C NMR spectra (101 MHz, $CDCl_3$, 25 ºC) of **1i**

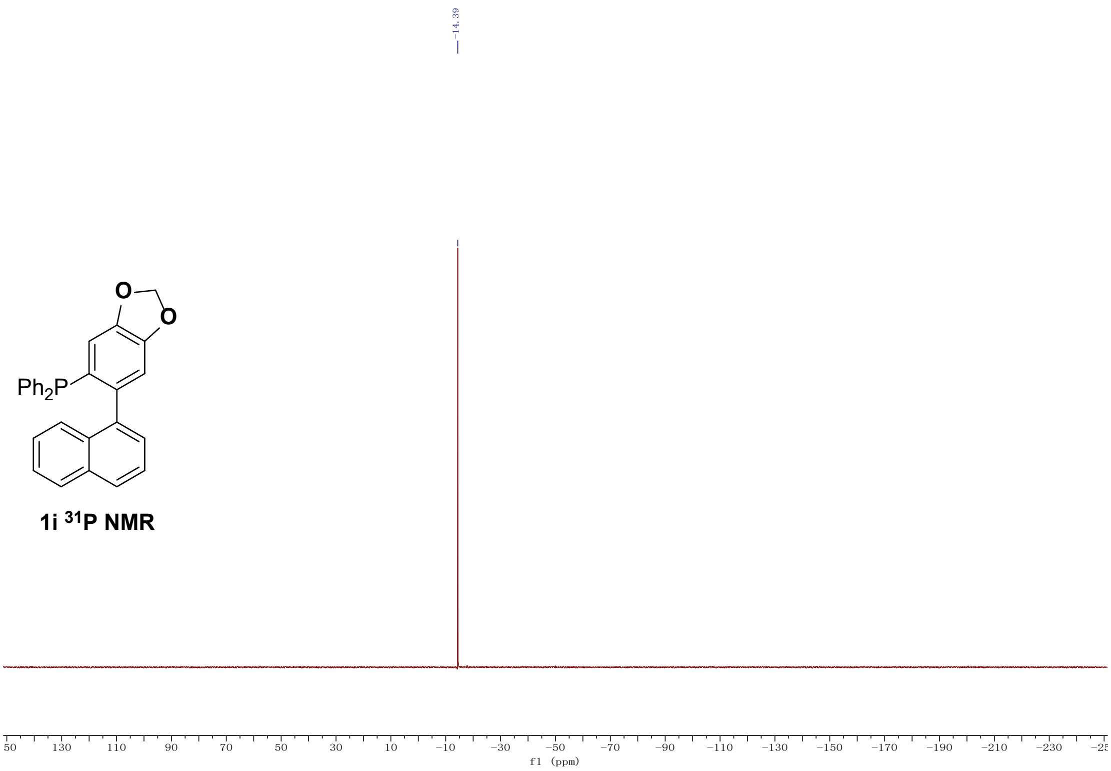

Supplementary Fig. 35. ^{31}P NMR spectra (162 MHz, $CDCl_3$, 25 ºC) of **1i**

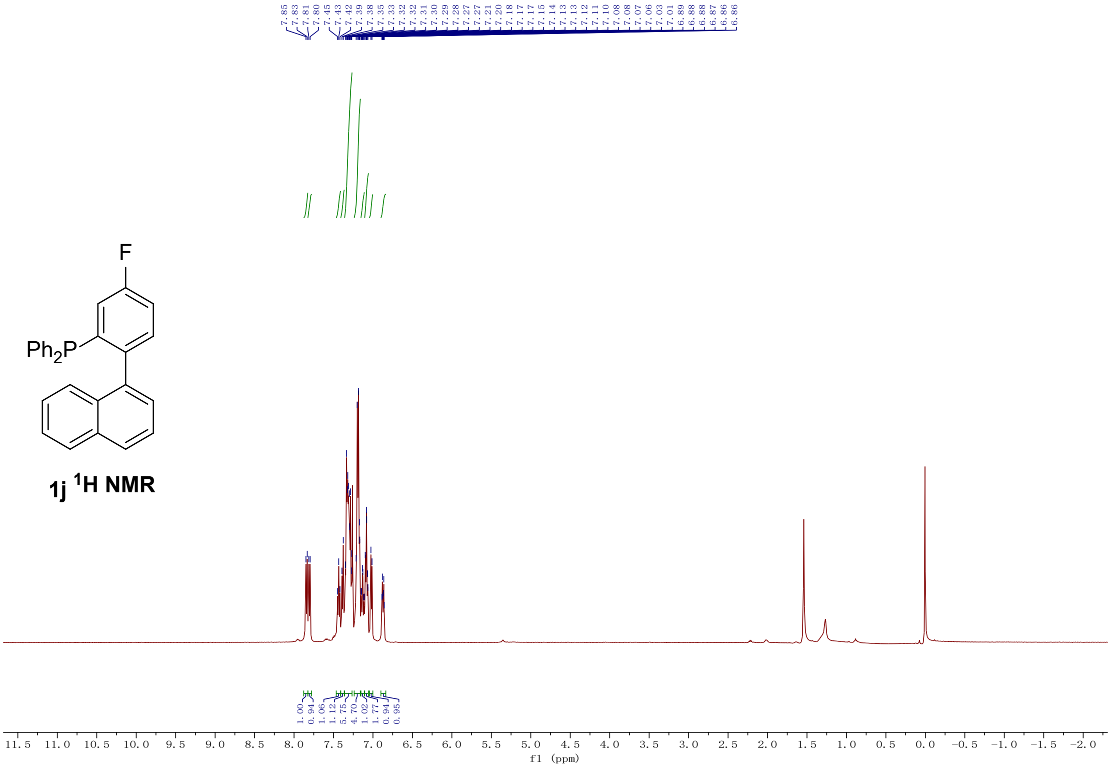

Supplementary Fig. 36. ^{1}H NMR spectra (500 MHz, $CDCl_3$, 25 ºC) of **1j**

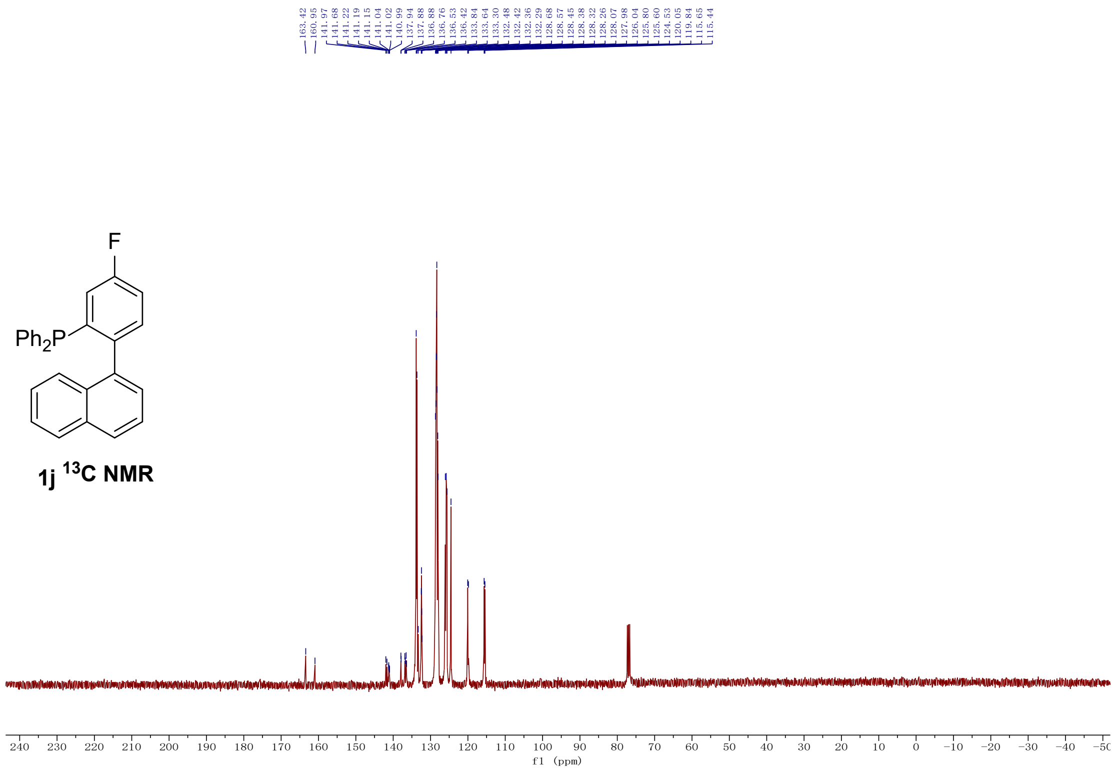

Supplementary Fig. 37. ^{13}C NMR spectra (101 MHz, $CDCl_3$, 25 ºC) of **1j**

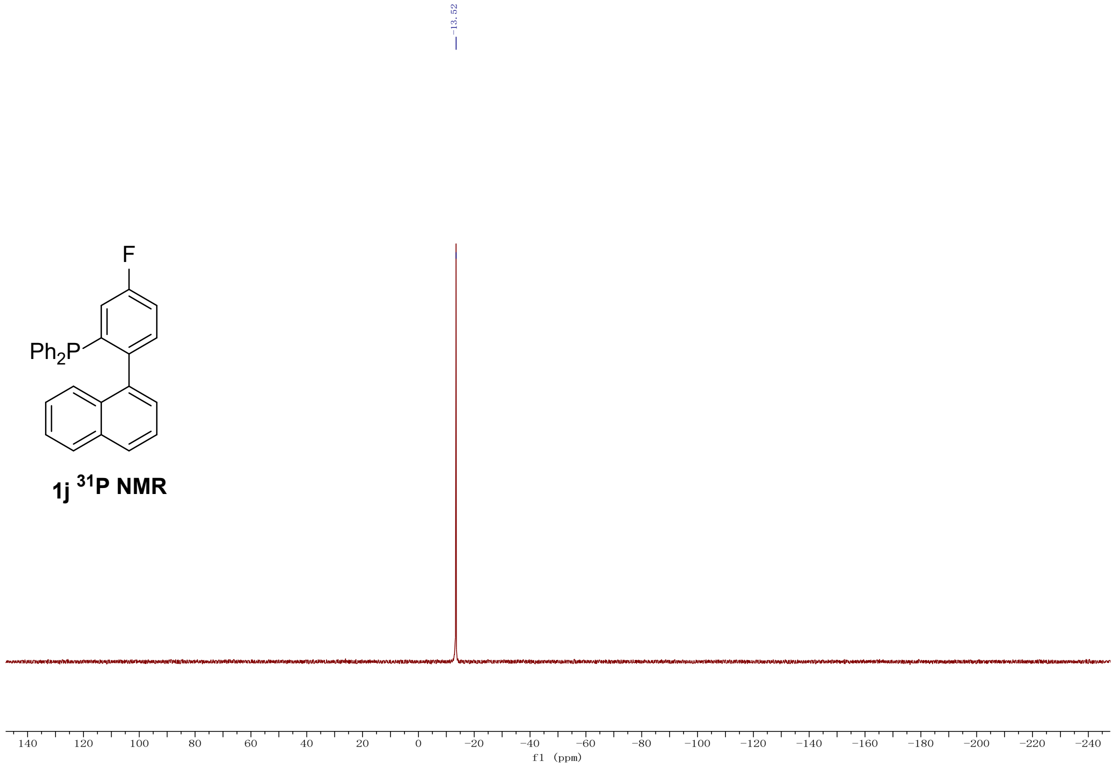

Supplementary Fig. 38. ^{31}P NMR spectra (162 MHz, $CDCl_3$, 25 ºC) of **1j**

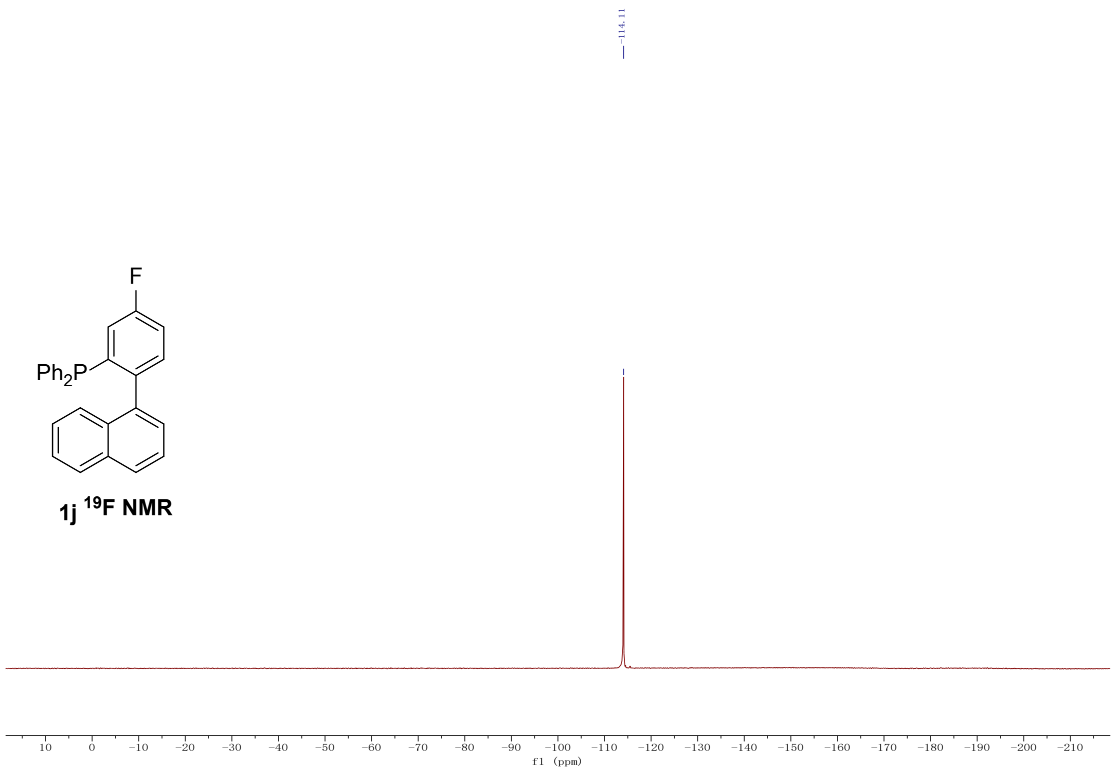

Supplementary Fig. 39. ^{19}F NMR spectra (376 MHz, $CDCl_3$, 25 °C) of **1j**

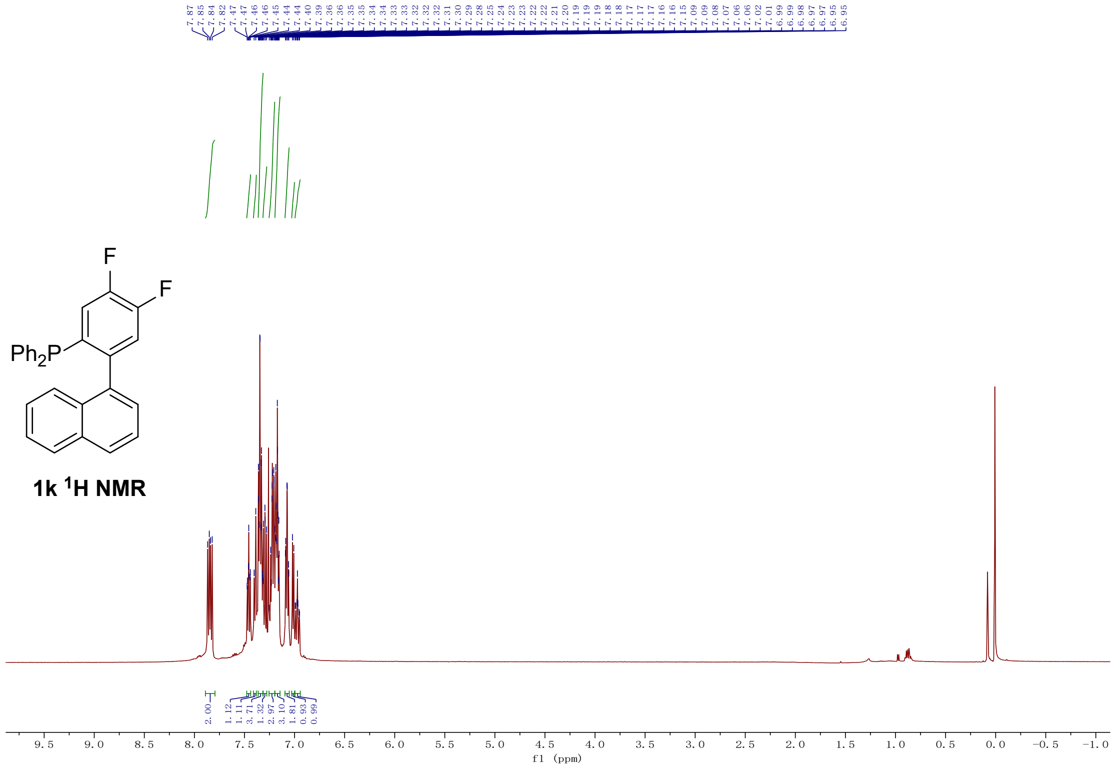

Supplementary Fig. 40. ^{1}H NMR spectra (500 MHz, $CDCl_3$, 25 °C) of **1k**

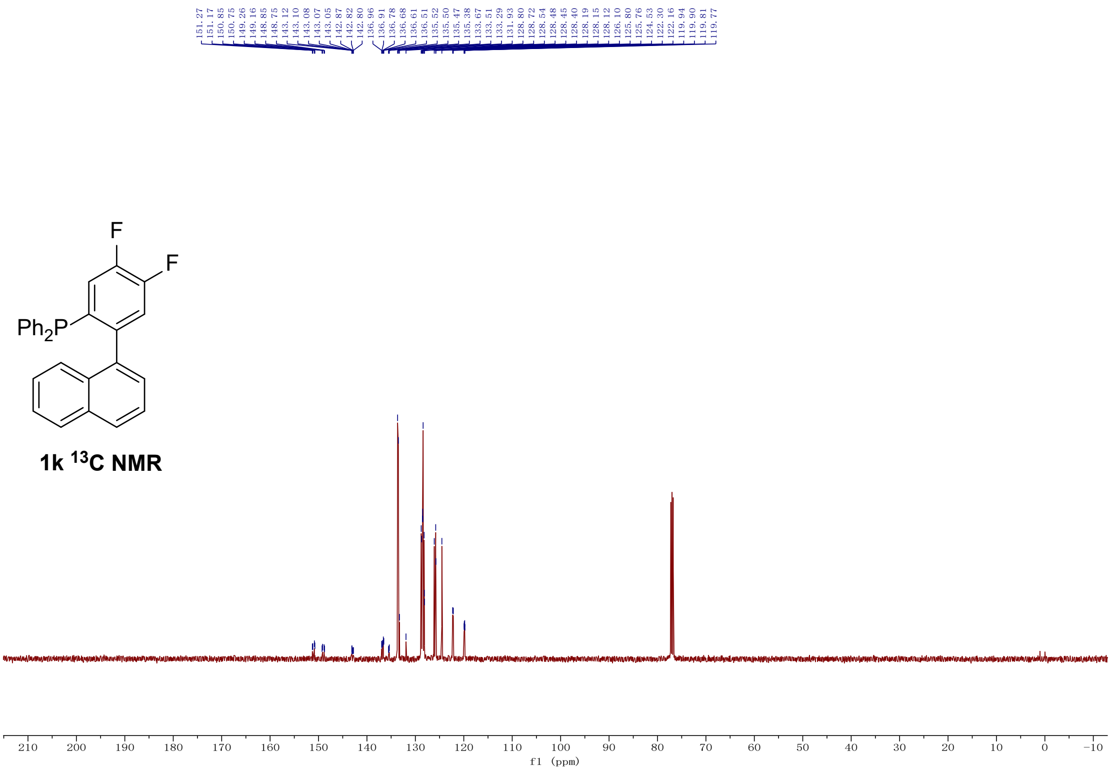

Supplementary Fig. 41. ^{13}C NMR spectra (126 MHz, CDCl3, 25 ºC) of **1k**

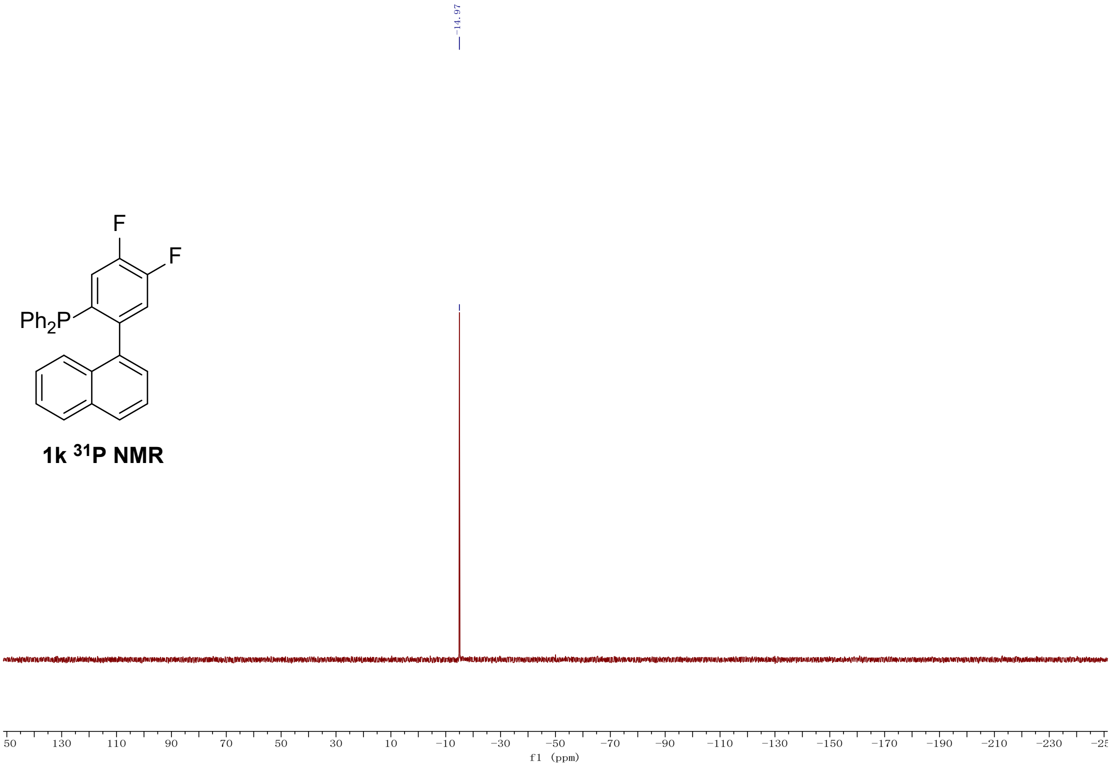

Supplementary Fig. 42. ^{31}P NMR spectra (202 MHz, CDCl3, 25 ºC) of **1k**

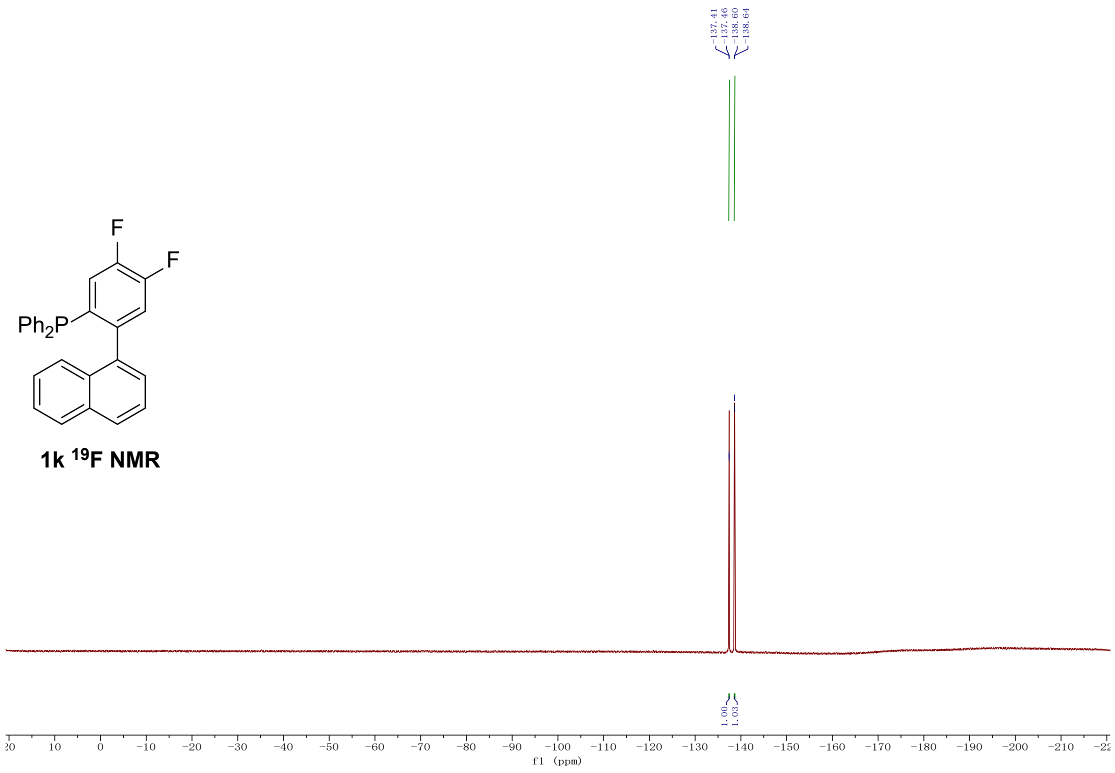

Supplementary Fig. 43. ^{19}F NMR spectra (471 MHz, $CDCl_3$, 25 ºC) of **1k**

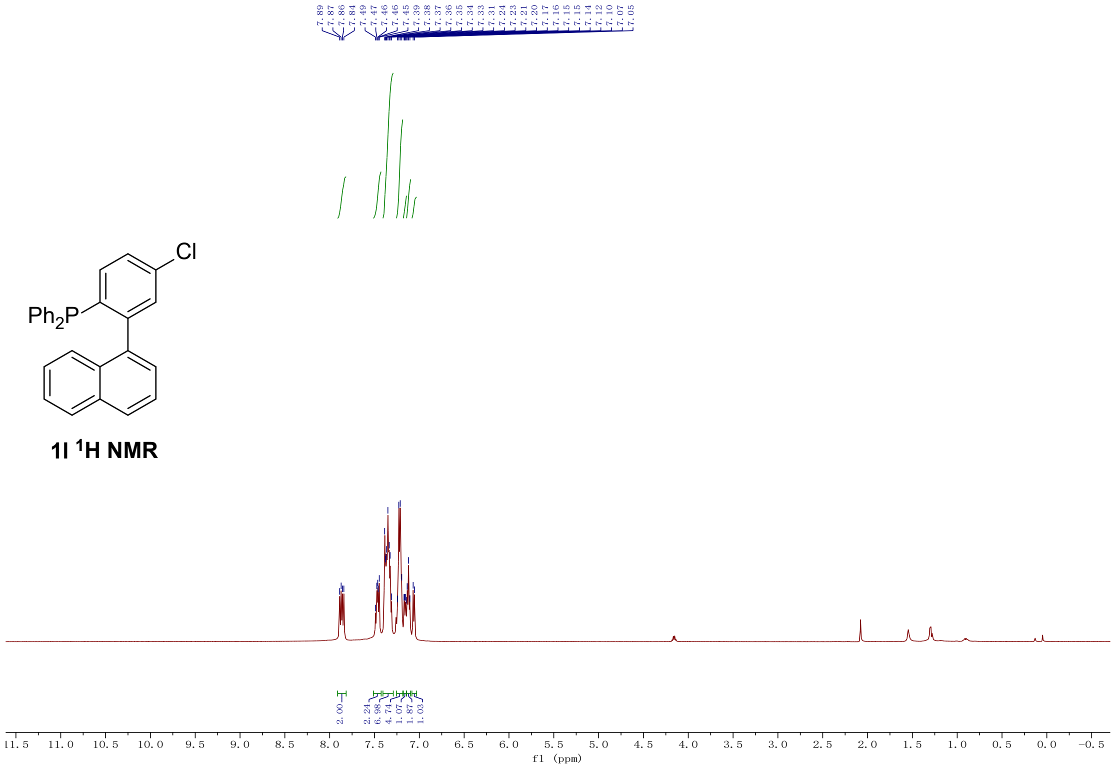

Supplementary Fig. 44. ^{1}H NMR spectra (500 MHz, $CDCl_3$, 25 ºC) of **1l**

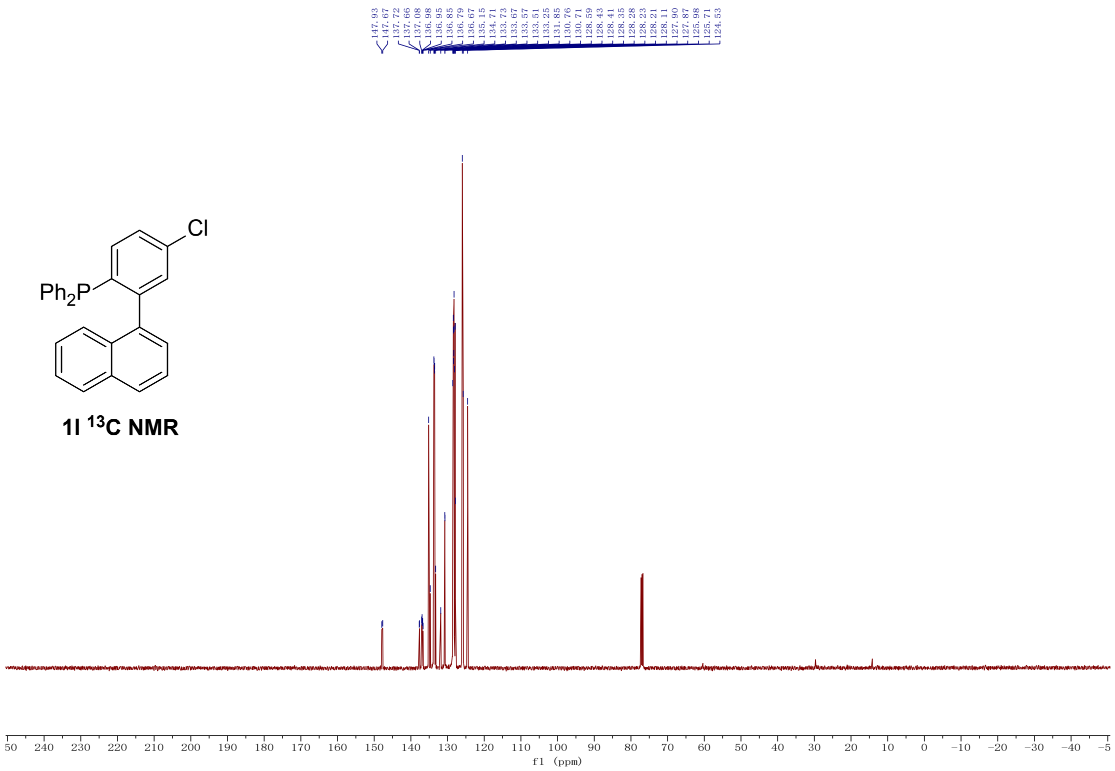

Supplementary Fig. 45. ^{13}C NMR spectra (126 MHz, CDCl$_3$, 25 °C) of **1l**

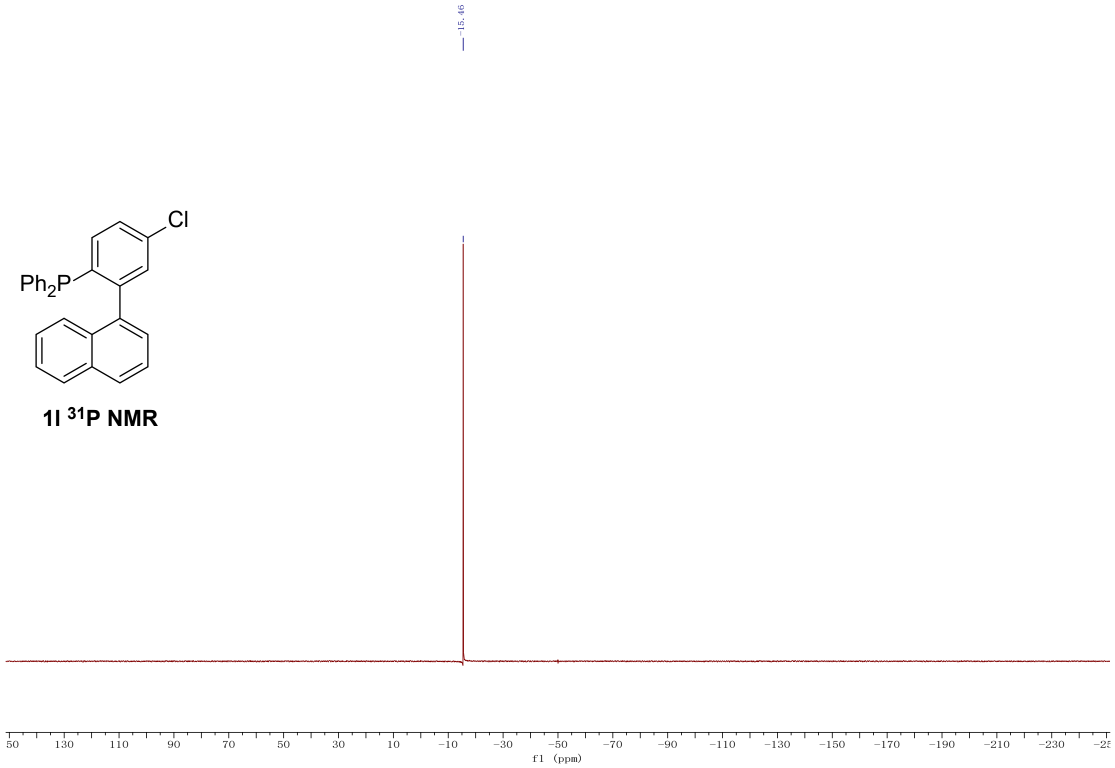

Supplementary Fig. 46. ^{31}P NMR spectra (202 MHz, CDCl$_3$, 25 °C) of **1l**

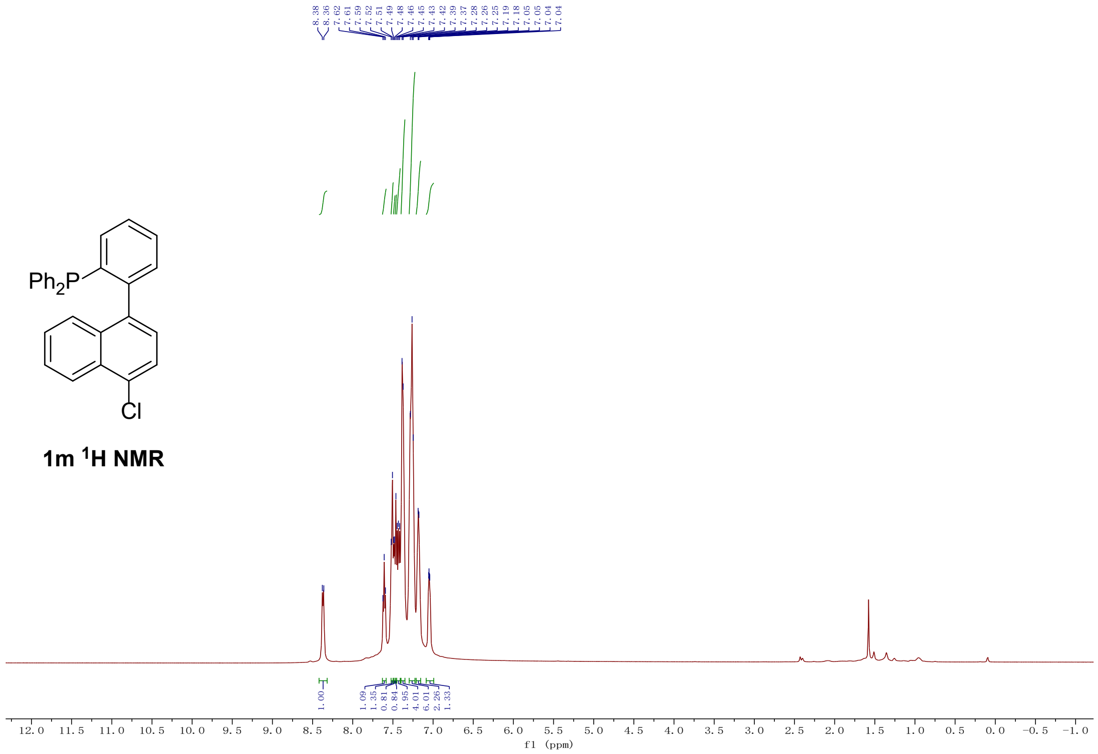

Supplementary Fig. 47. ^{1}H NMR spectra (500 MHz, $CDCl_3$, 25 ºC) of **1m**

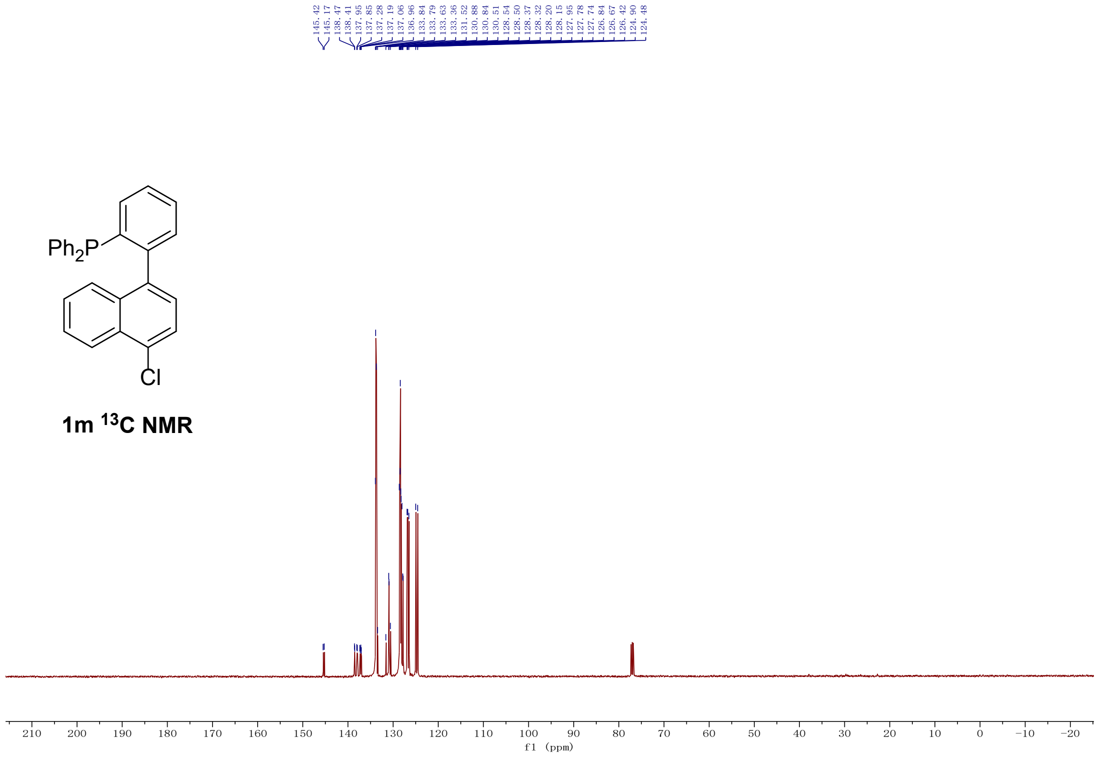

Supplementary Fig. 48. ^{13}C NMR spectra (126 MHz, $CDCl_3$, 25 ºC) of **1m**

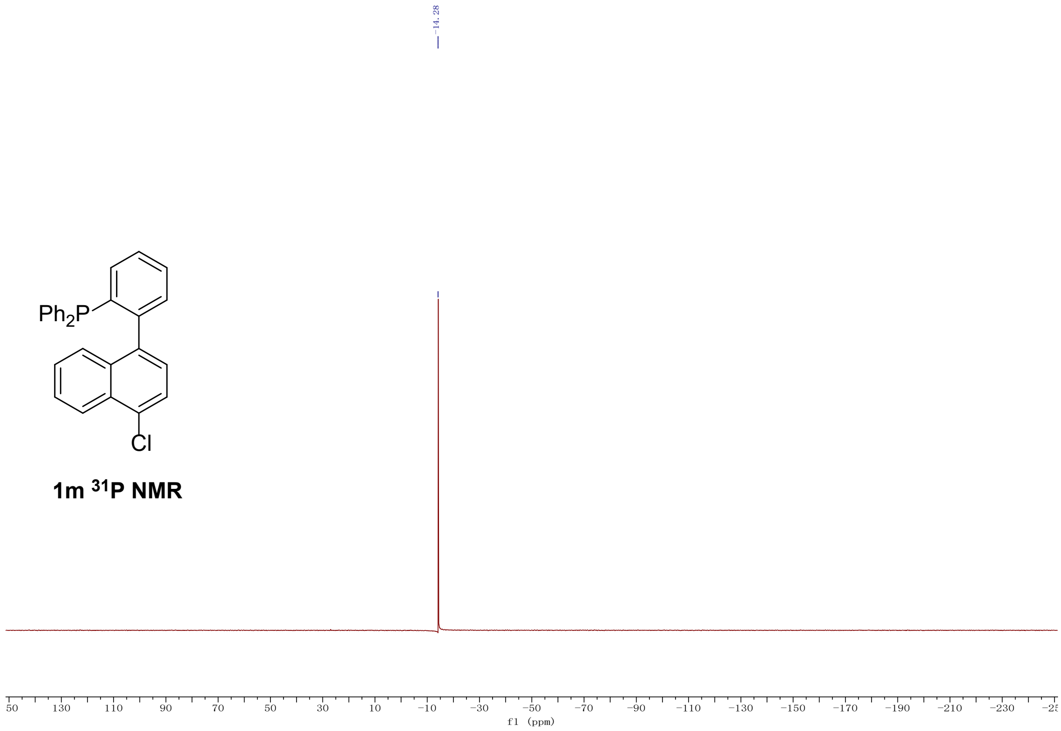

Supplementary Fig. 49. ^{31}P NMR spectra (162 MHz, $CDCl_3$, 25 ºC) of **1m**

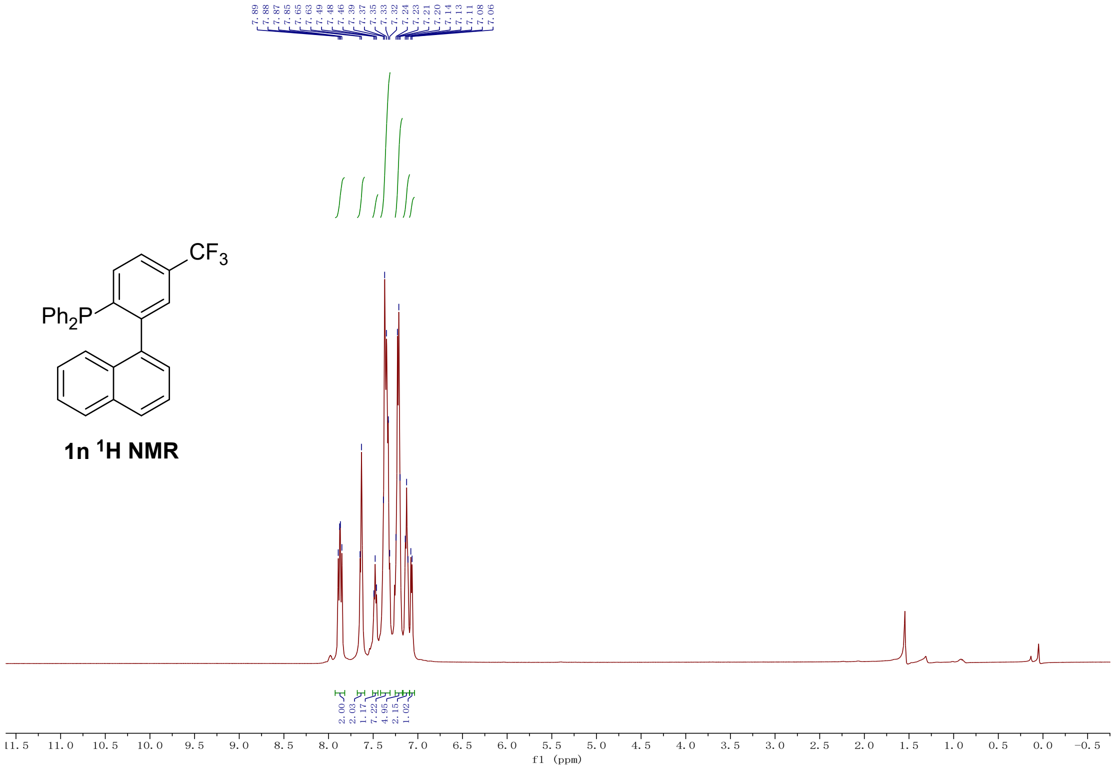

Supplementary Fig. 50. ^{1}H NMR spectra (500 MHz, $CDCl_3$, 25 ºC) of **1n**

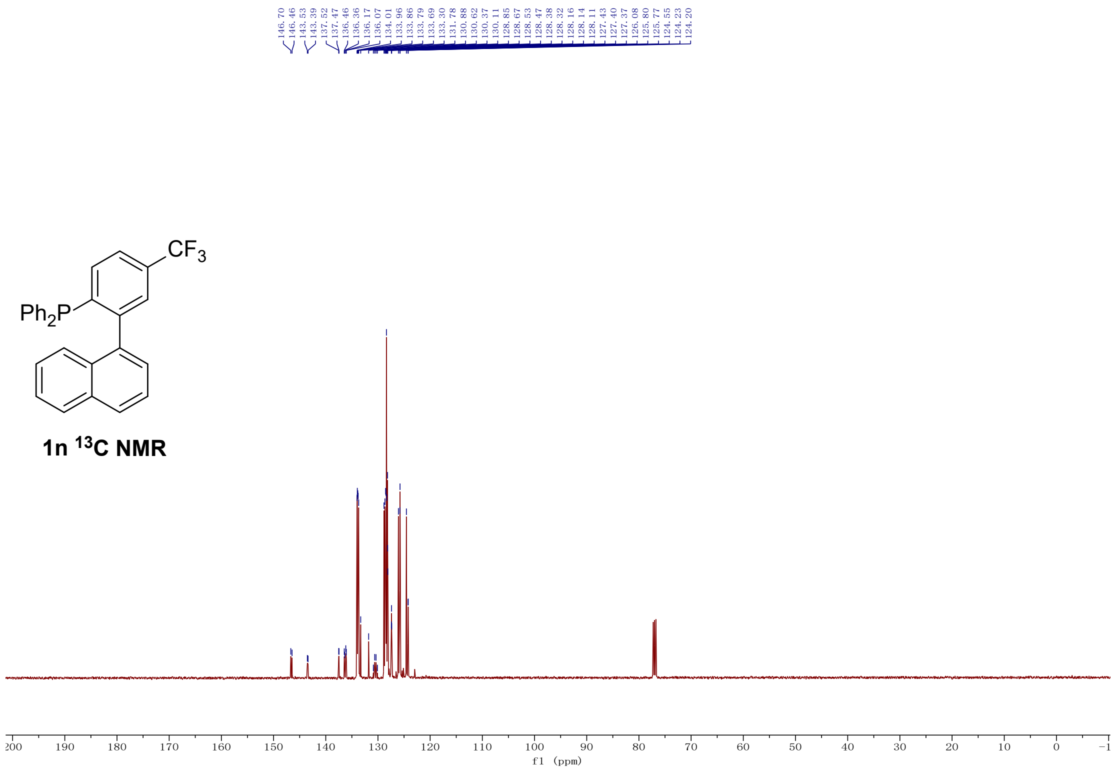

Supplementary Fig. 51. ^{13}C NMR spectra (126 MHz, $CDCl_3$, 25 °C) of **1n**

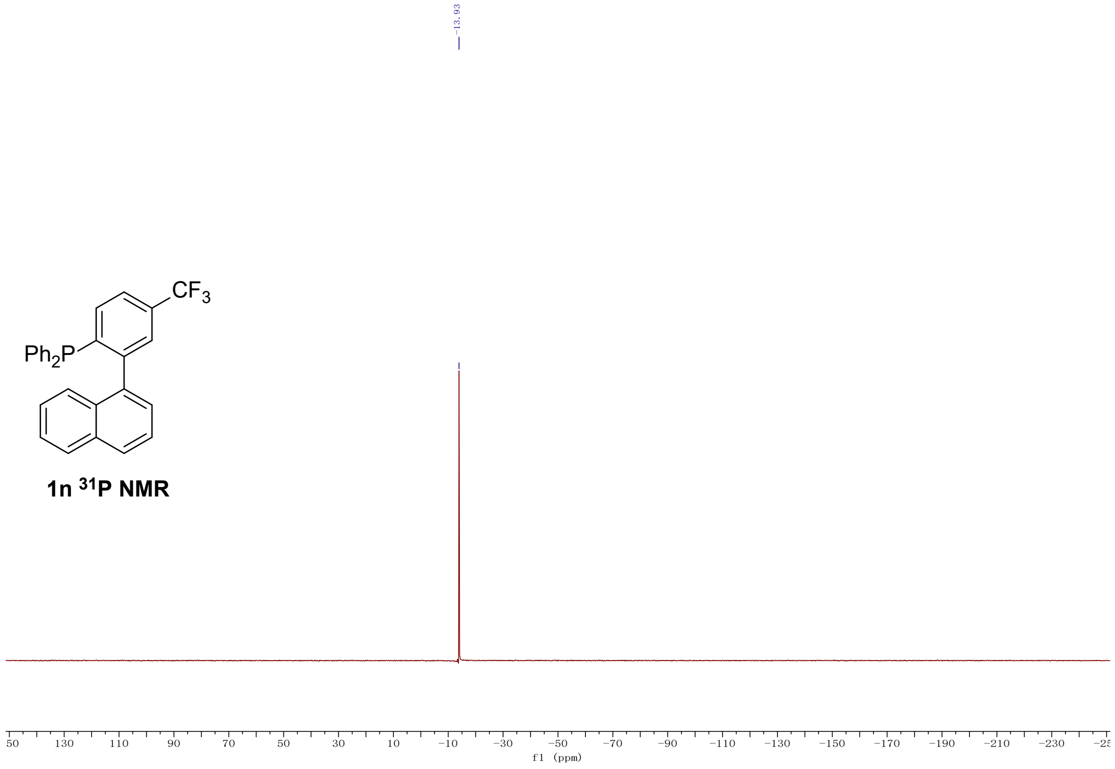

Supplementary Fig. 52. ^{31}P NMR spectra (202 MHz, $CDCl_3$, 25 °C) of **1n**

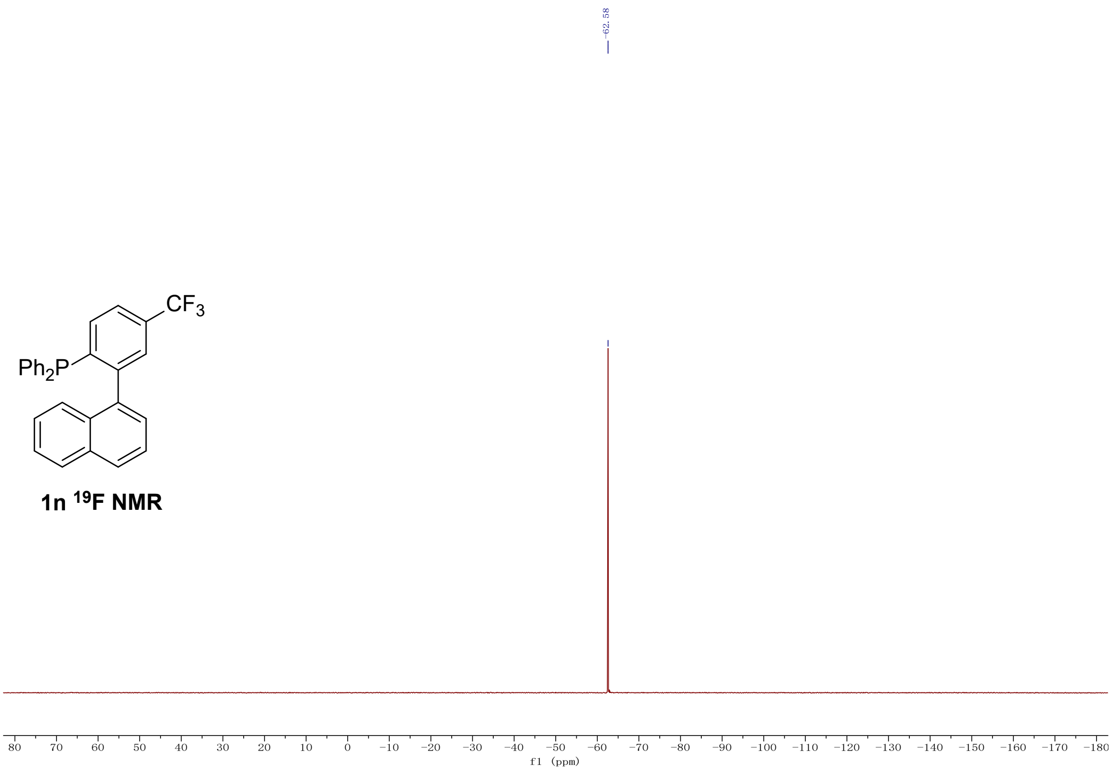

Supplementary Fig. 53. ^{19}F NMR spectra (471 MHz, $CDCl_3$, 25 ºC) of **1n**

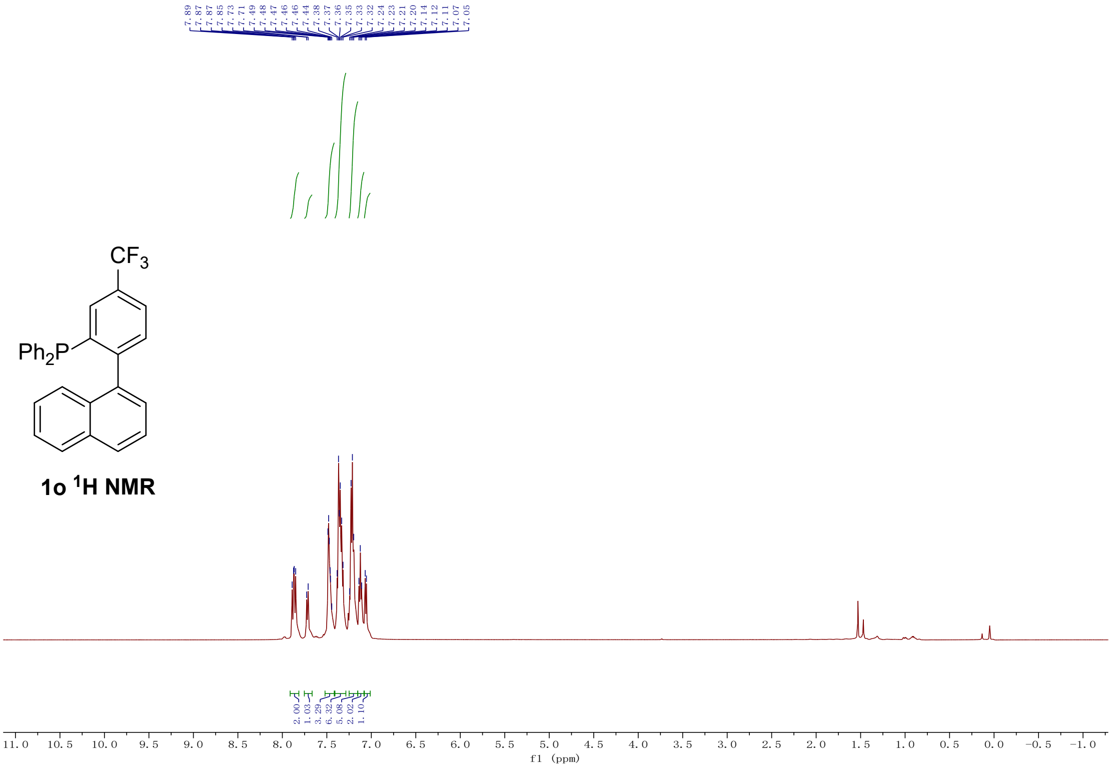

Supplementary Fig. 54. ^{1}H NMR spectra (500 MHz, $CDCl_3$, 25 ºC) of **1o**

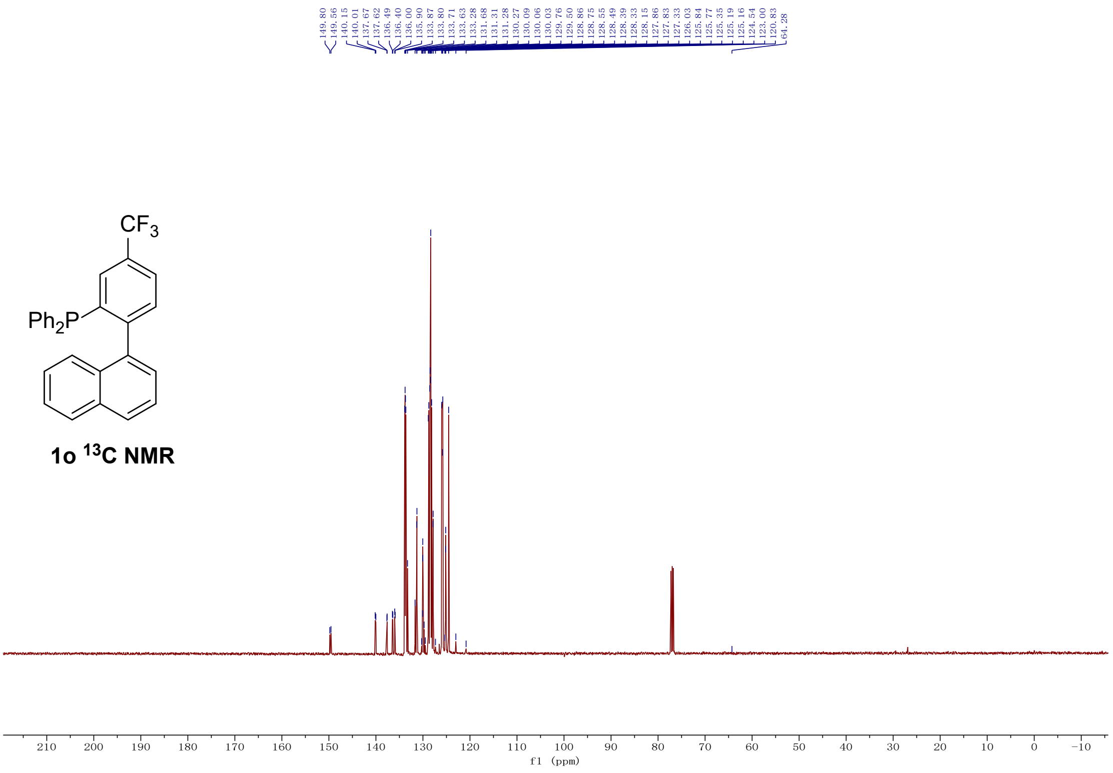

Supplementary Fig. 55. ^{13}C NMR spectra (126 MHz, $CDCl_3$, 25 ºC) of **1o**

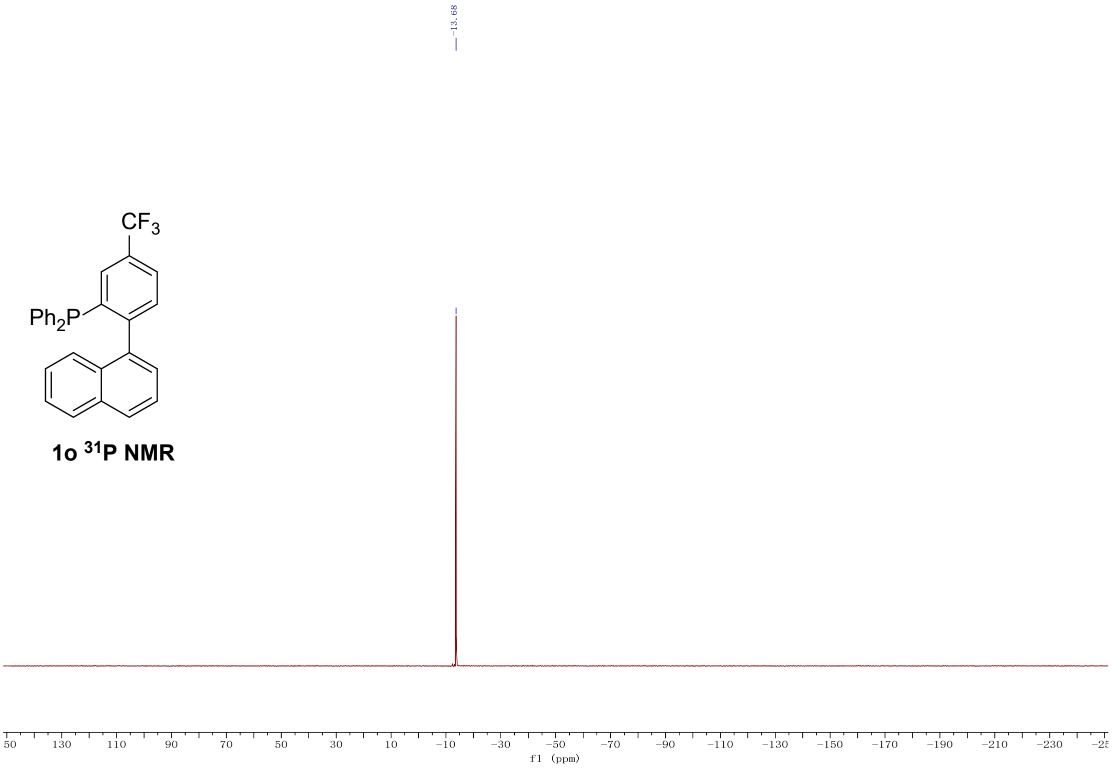

Supplementary Fig. 56. ^{31}P NMR spectra (202 MHz, $CDCl_3$, 25 ºC) of **1o**

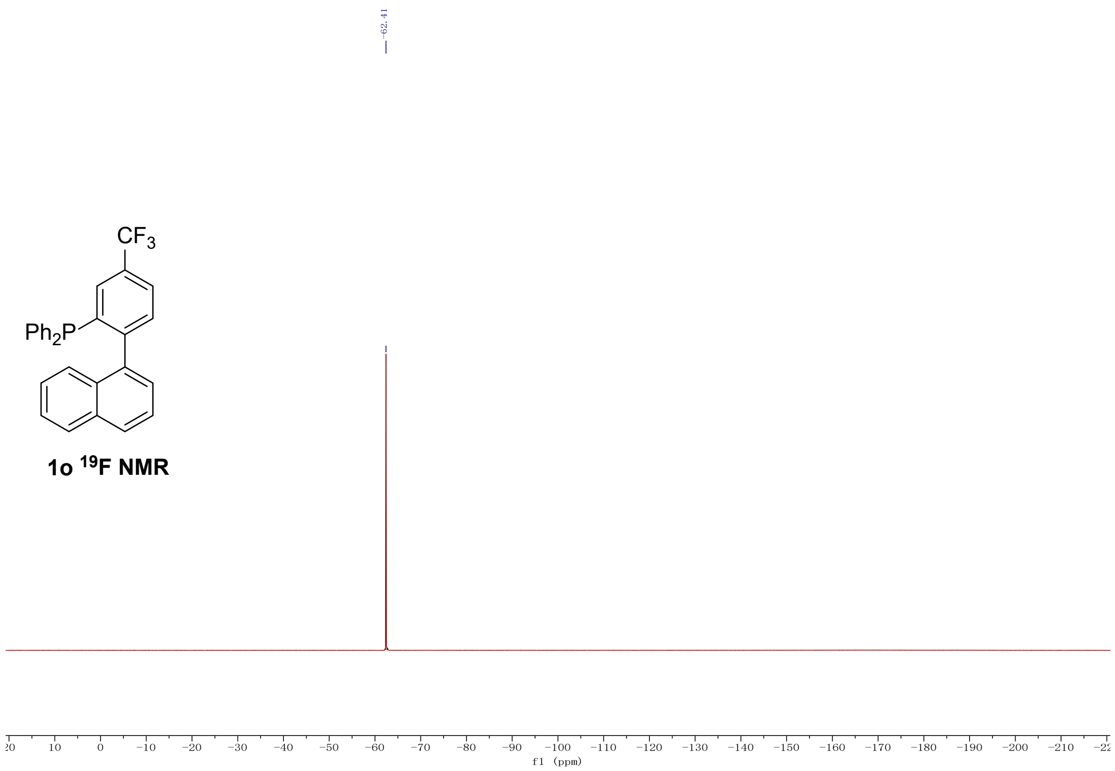

Supplementary Fig. 57. ^{19}F NMR spectra (471 MHz, $CDCl_3$, 25 ºC) of **1o**

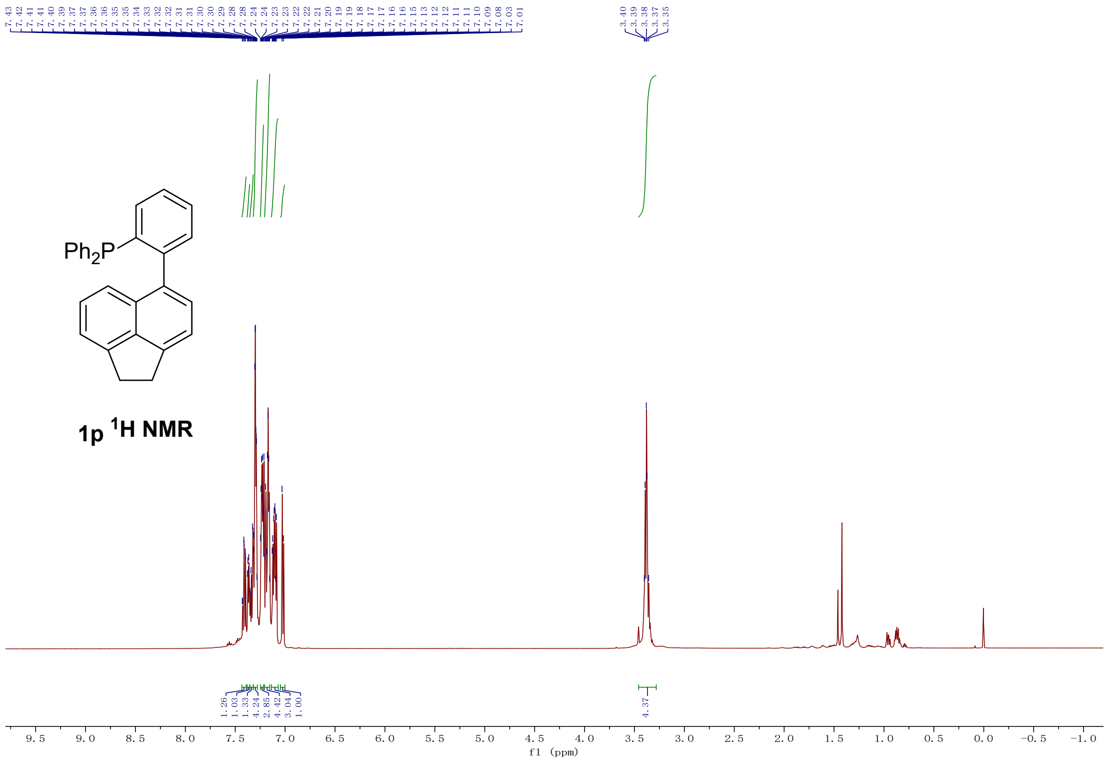

Supplementary Fig. 58. ^{1}H NMR spectra (500 MHz, $CDCl_3$, 25 ºC) of **1p**

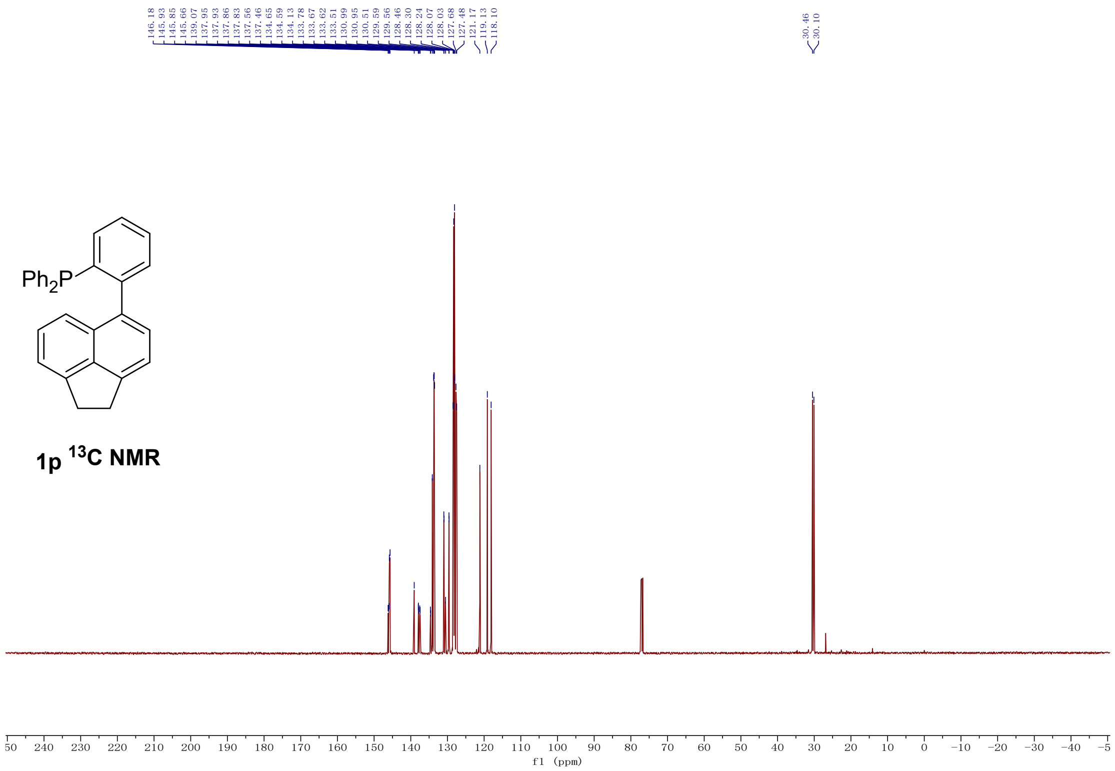

Supplementary Fig. 59. ^{13}C NMR spectra (126 MHz, $CDCl_3$, 25 °C) of **1p**

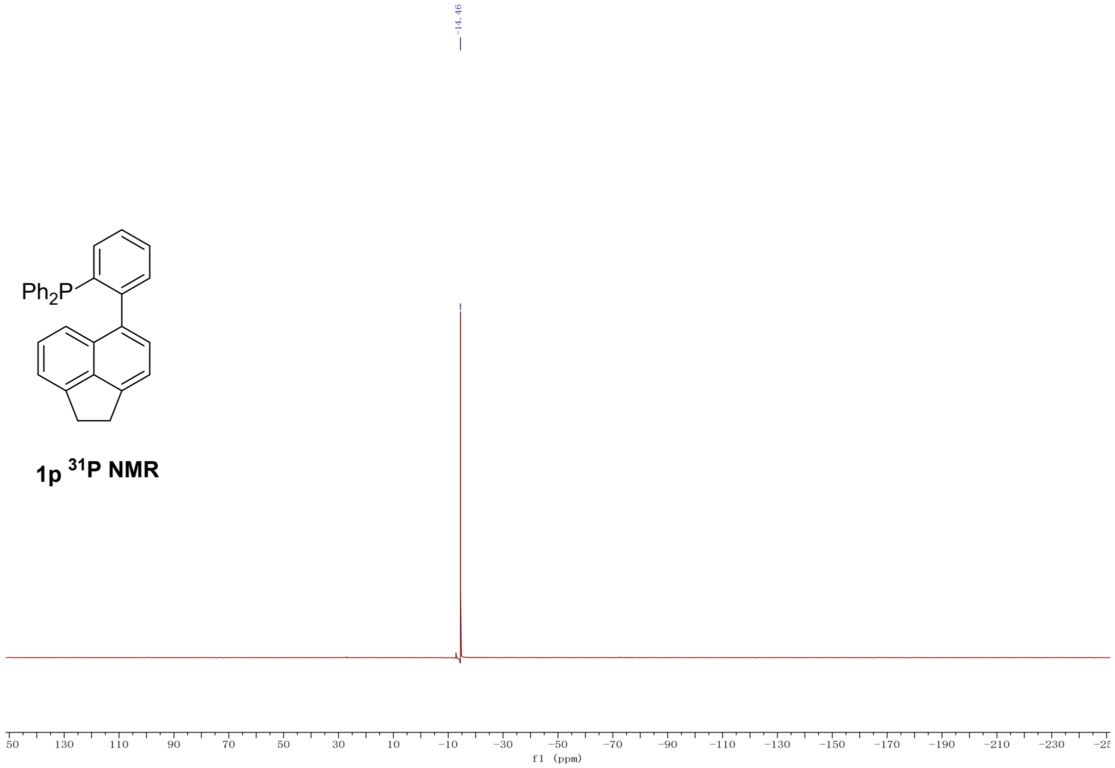

Supplementary Fig. 60. ^{31}P NMR spectra (202 MHz, $CDCl_3$, 25 °C) of **1p**

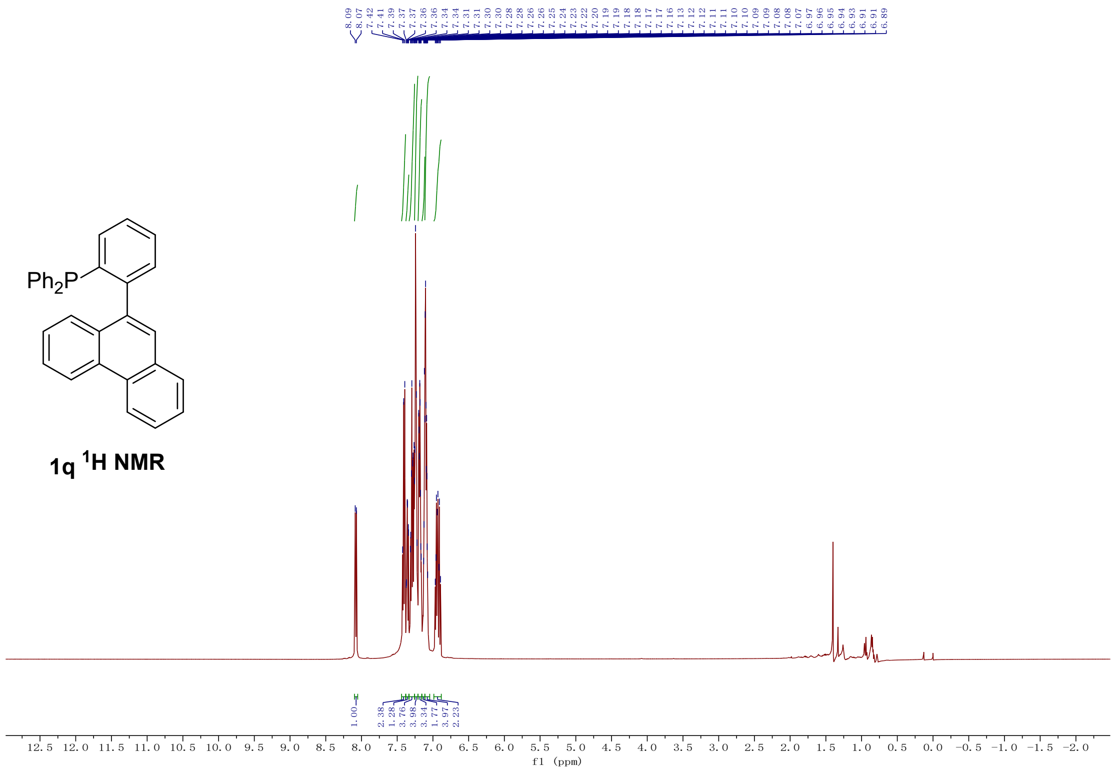

Supplementary Fig. 61. ^{1}H NMR spectra (500 MHz, $CDCl_3$, 25 ºC) of **1q**

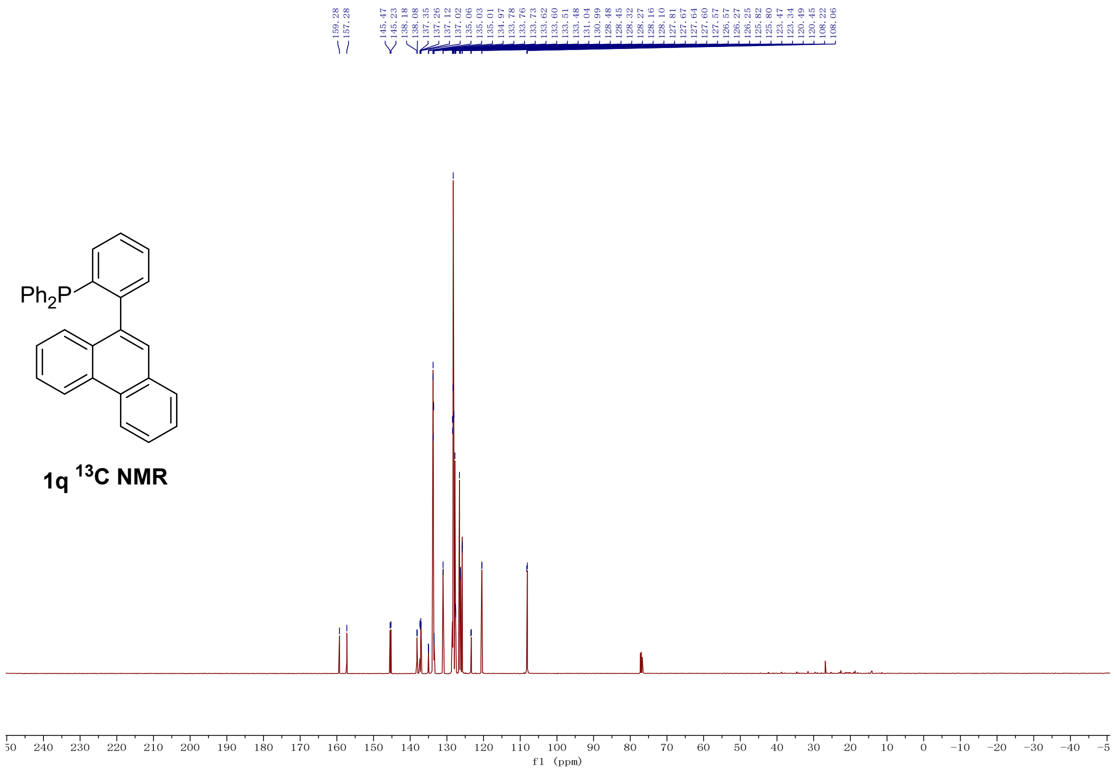

Supplementary Fig. 62. ^{13}C NMR spectra (126 MHz, $CDCl_3$, 25 ºC) of **1q**

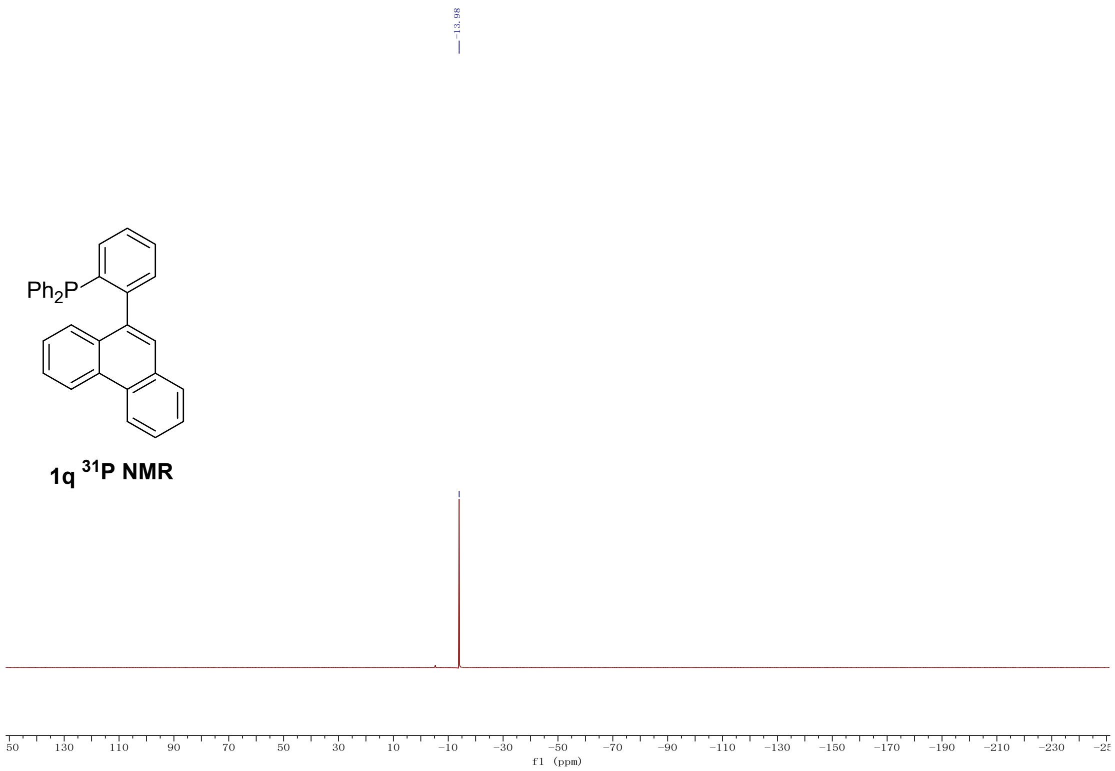

Supplementary Fig. 63. ^{31}P NMR spectra (202 MHz, $CDCl_3$, 25 ºC) of **1q**

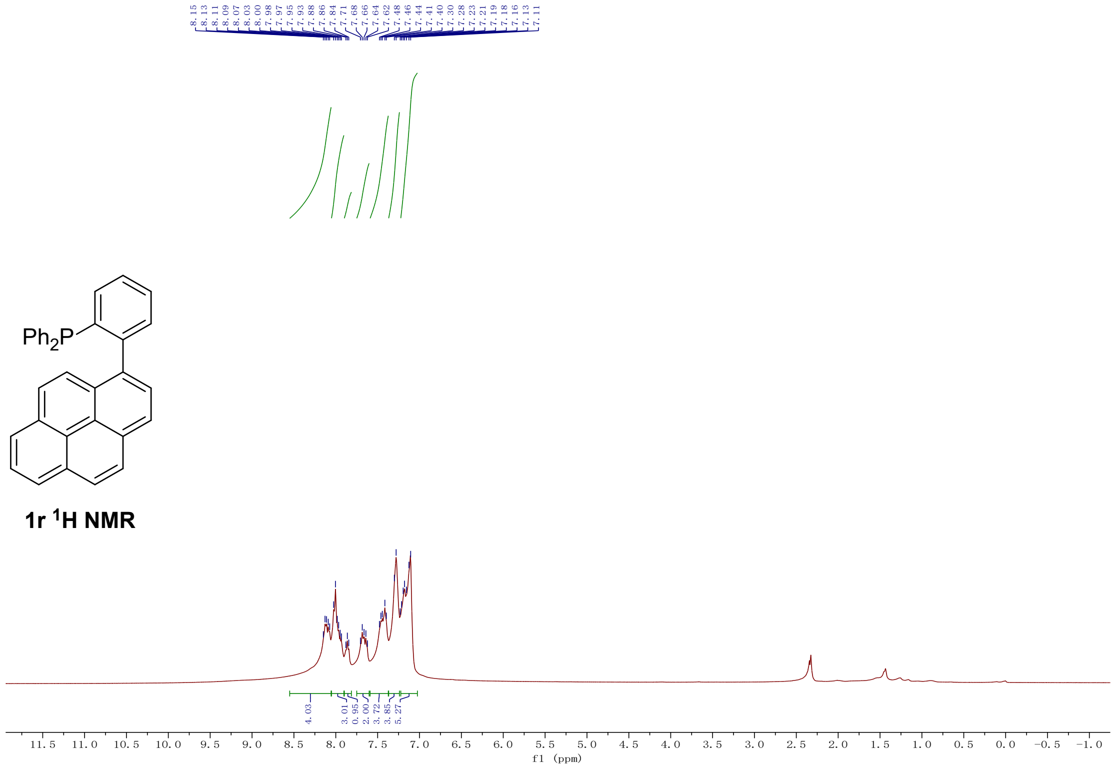

Supplementary Fig. 64. ^{1}H NMR spectra (500 MHz, $CDCl_3$, 25 ºC) of **1r**

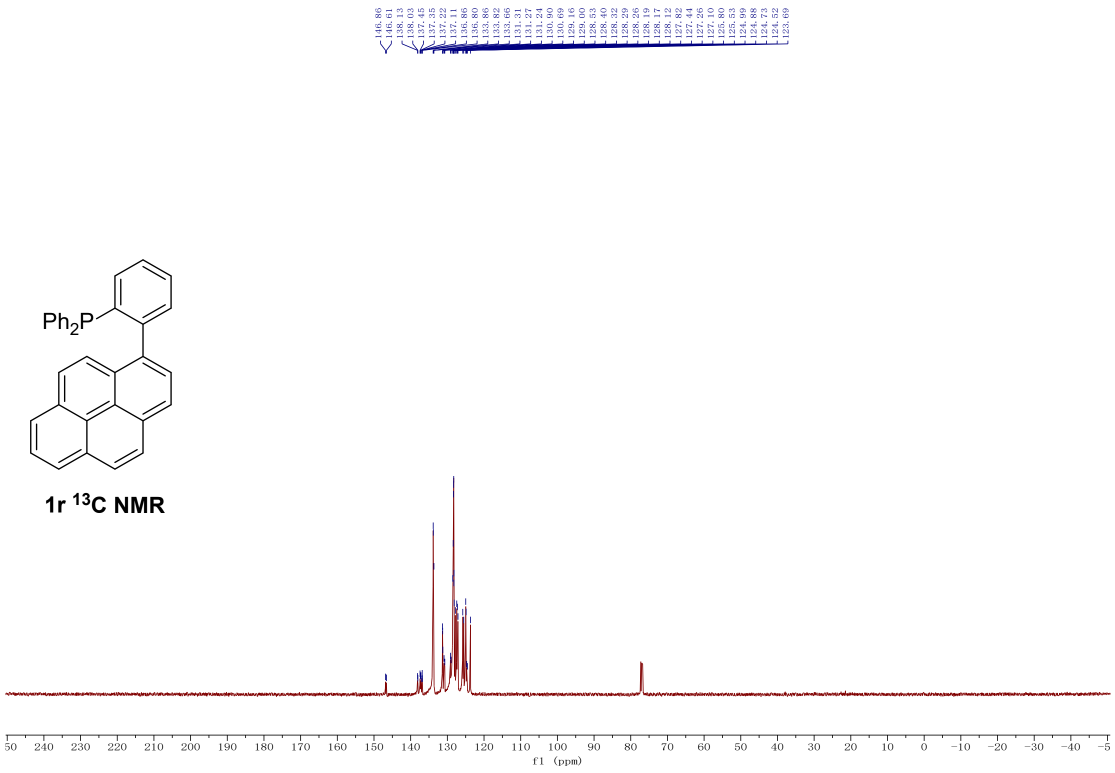

Supplementary Fig. 65. ^{13}C NMR spectra (126 MHz, CDCl₃, 25 ºC) of **1r**

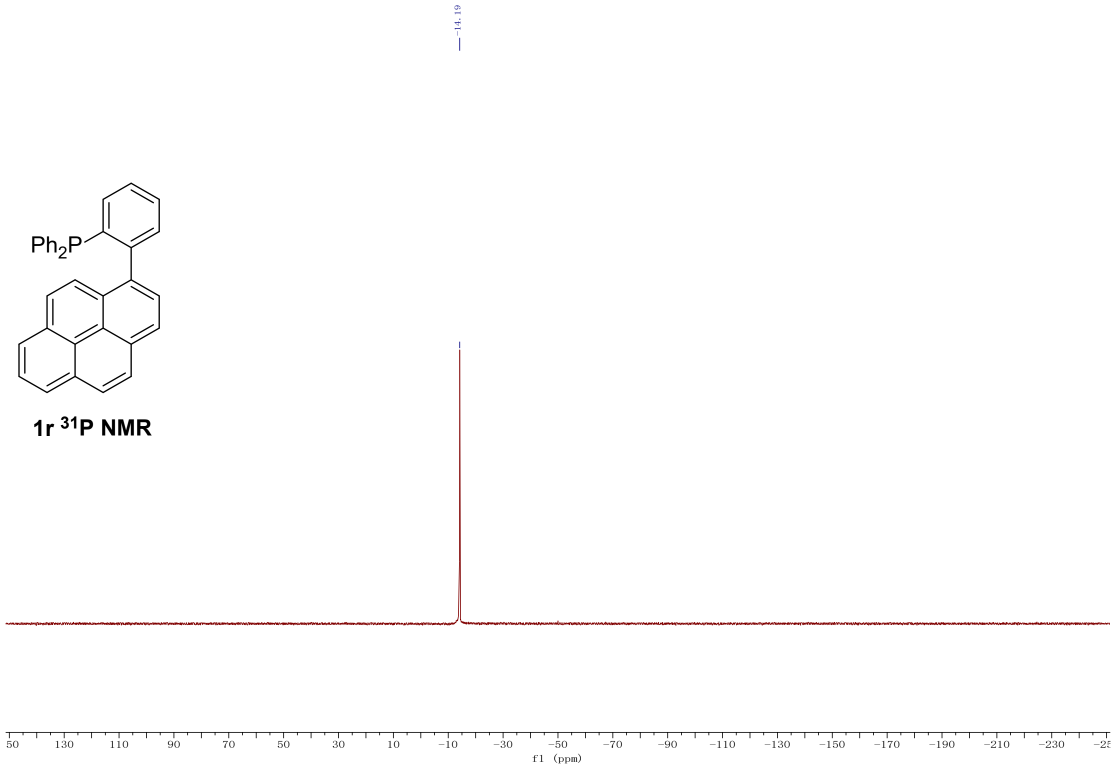

Supplementary Fig. 66. ^{31}P NMR spectra (202 MHz, CDCl₃, 25 ºC) of **1r**

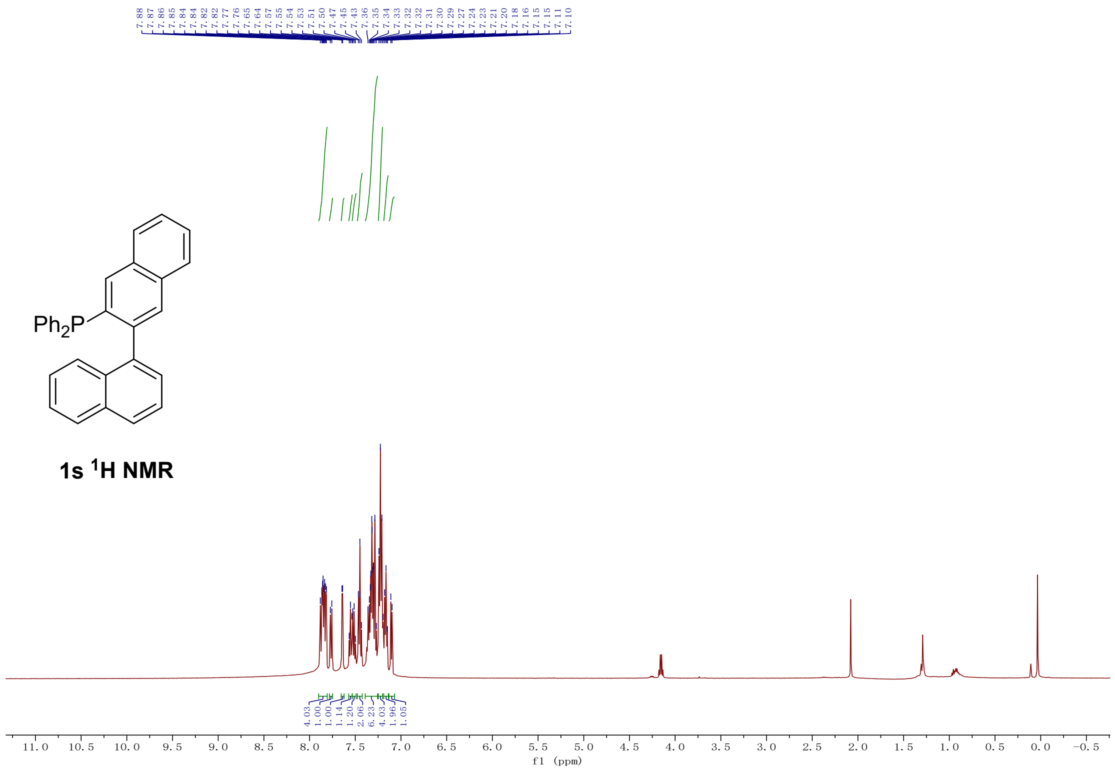

Supplementary Fig. 67. ^{1}H NMR spectra (500 MHz, $CDCl_3$, 25 °C) of **1s**

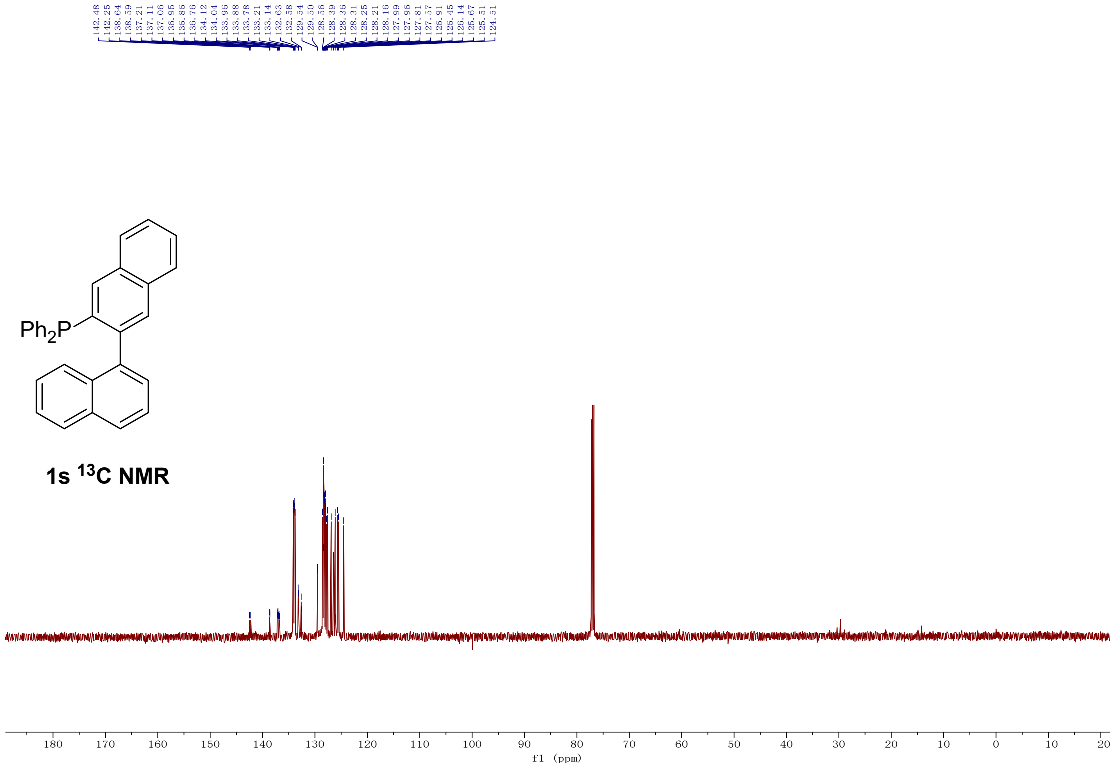

Supplementary Fig. 68. ^{13}C NMR spectra (126 MHz, $CDCl_3$, 25 °C) of **1s**

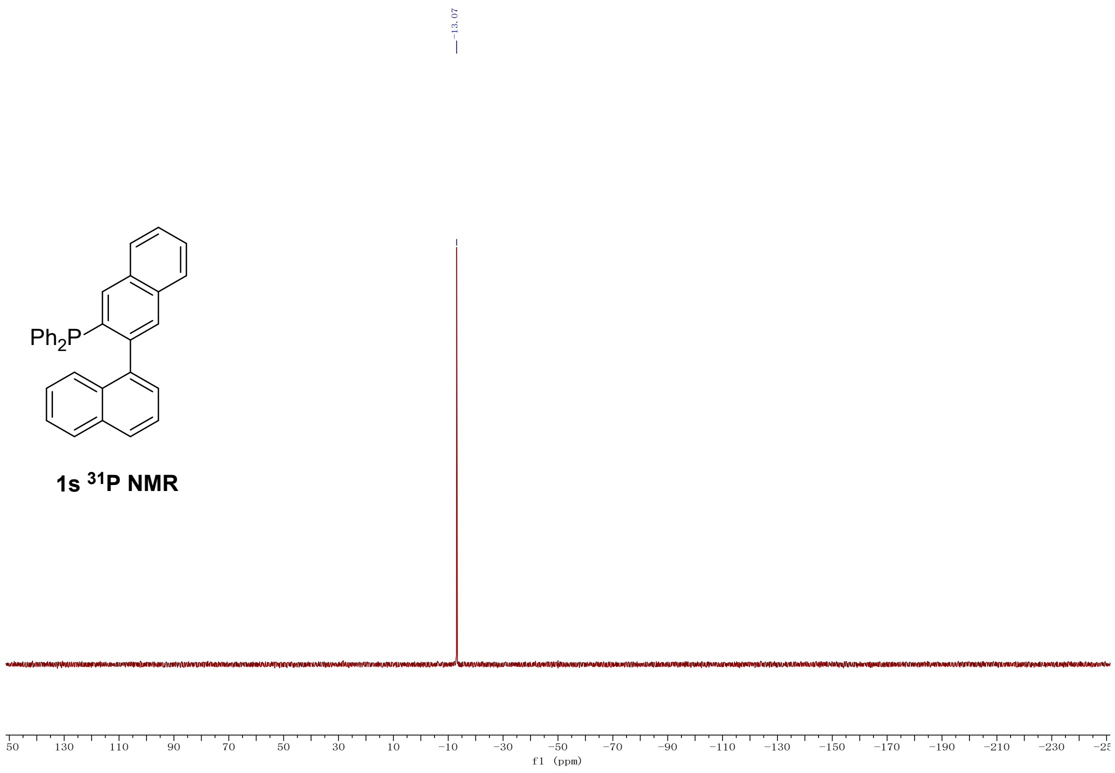

Supplementary Fig. 69. ^{31}P NMR spectra (202 MHz, $CDCl_3$, 25 °C) of **1s**

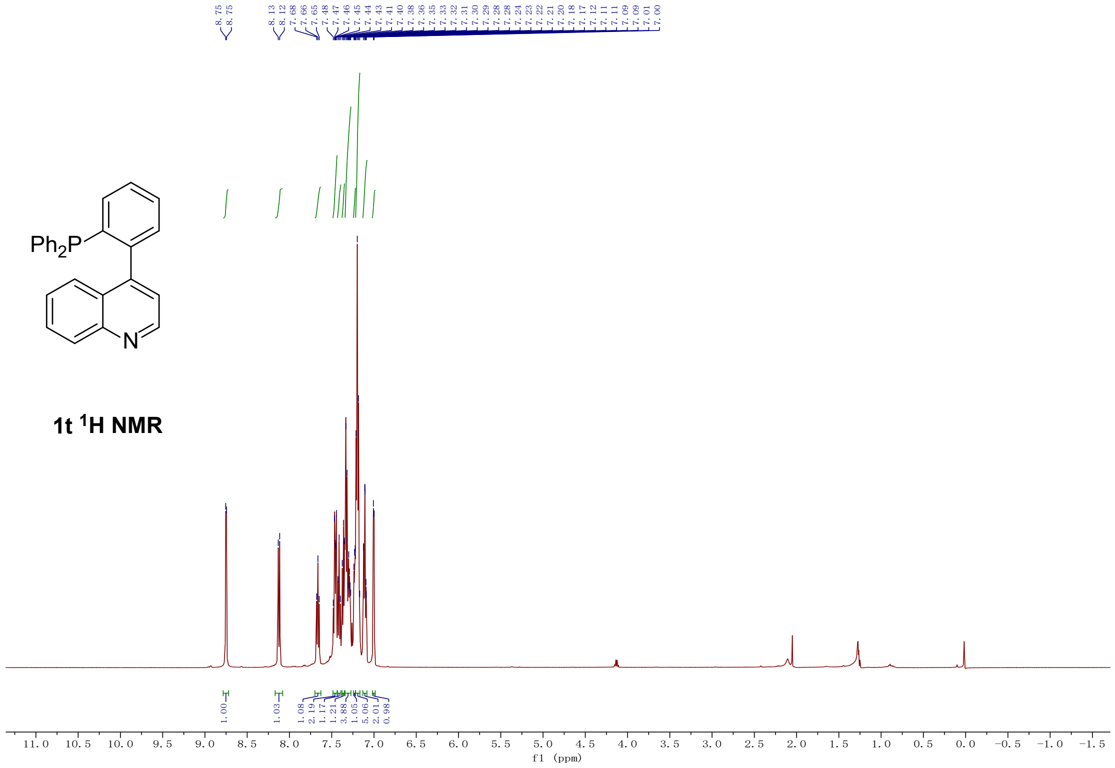

Supplementary Fig. 70. ^{1}H NMR spectra (500 MHz, $CDCl_3$, 25 °C) of **1t**

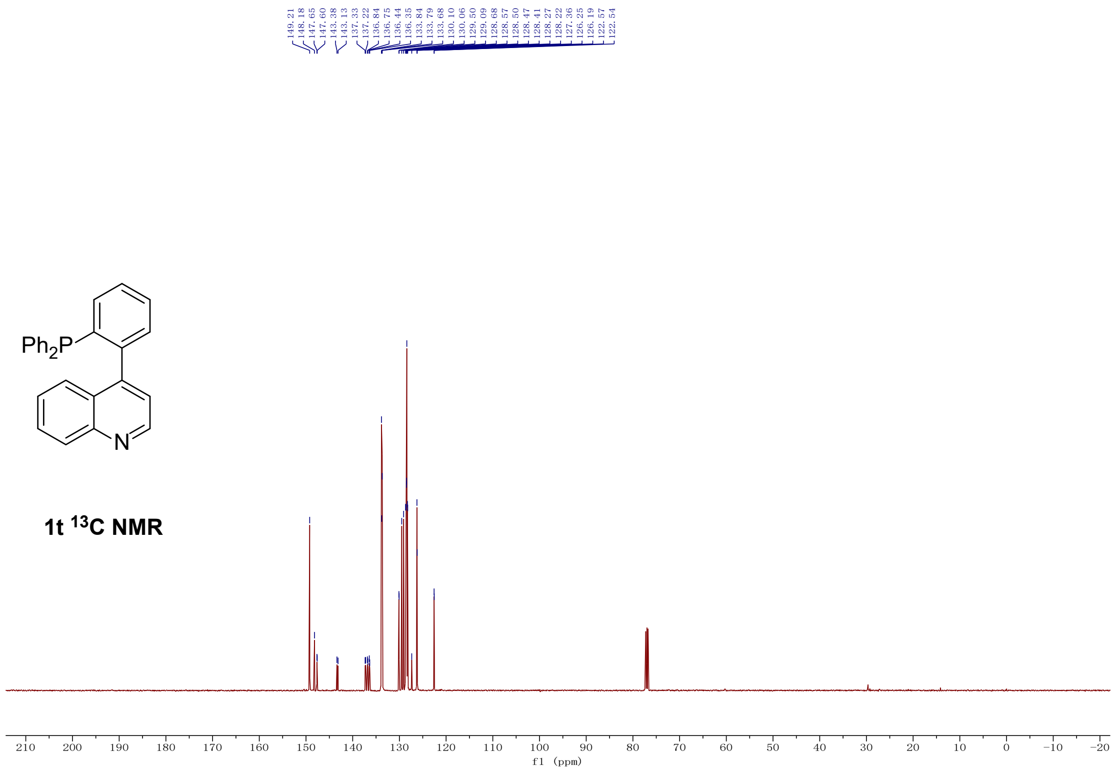

Supplementary Fig. 71. ^{13}C NMR spectra (126 MHz, $CDCl_3$, 25 ºC) of **1t**

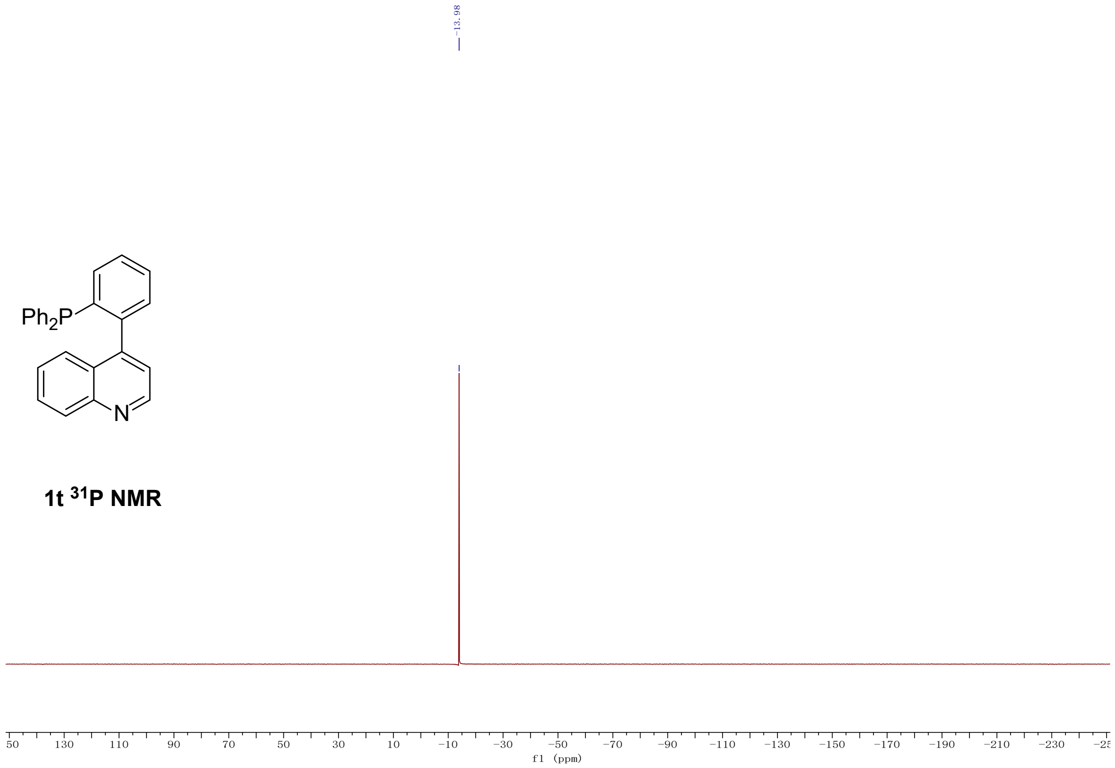

Supplementary Fig. 72. ^{31}P NMR spectra (202 MHz, $CDCl_3$, 25 ºC) of **1t**

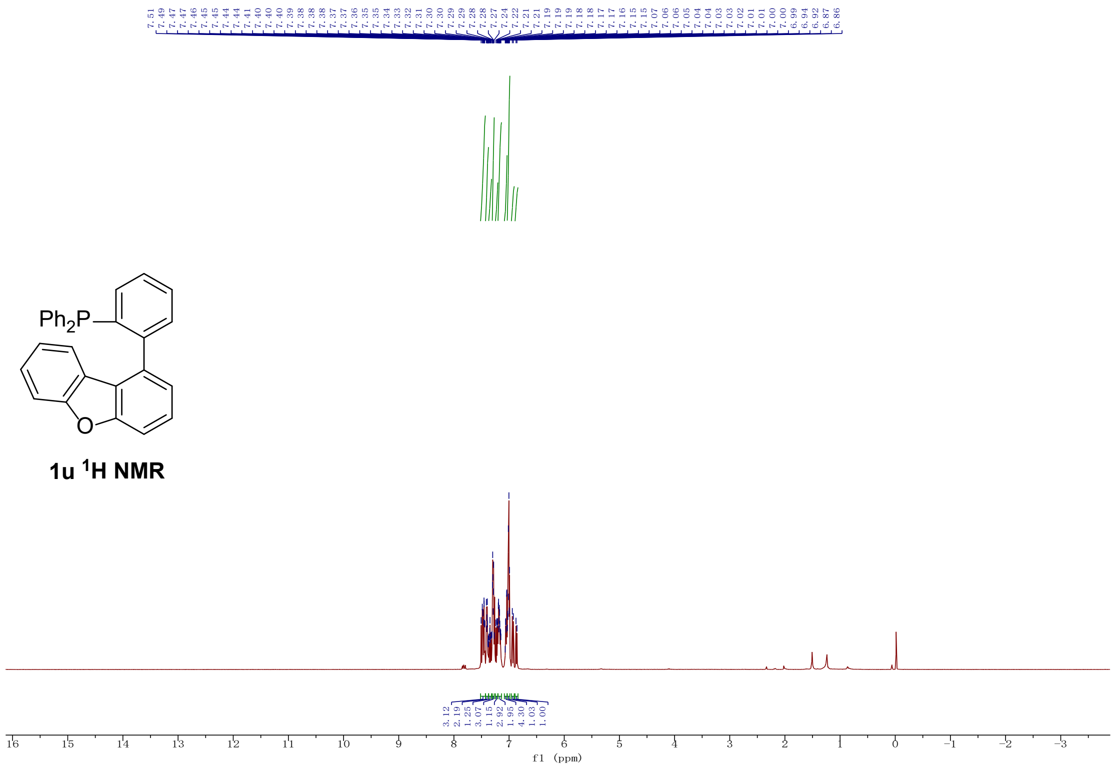

Supplementary Fig. 73. ^{1}H NMR spectra (400 MHz, CDCl3, 25 ºC) of **1u**

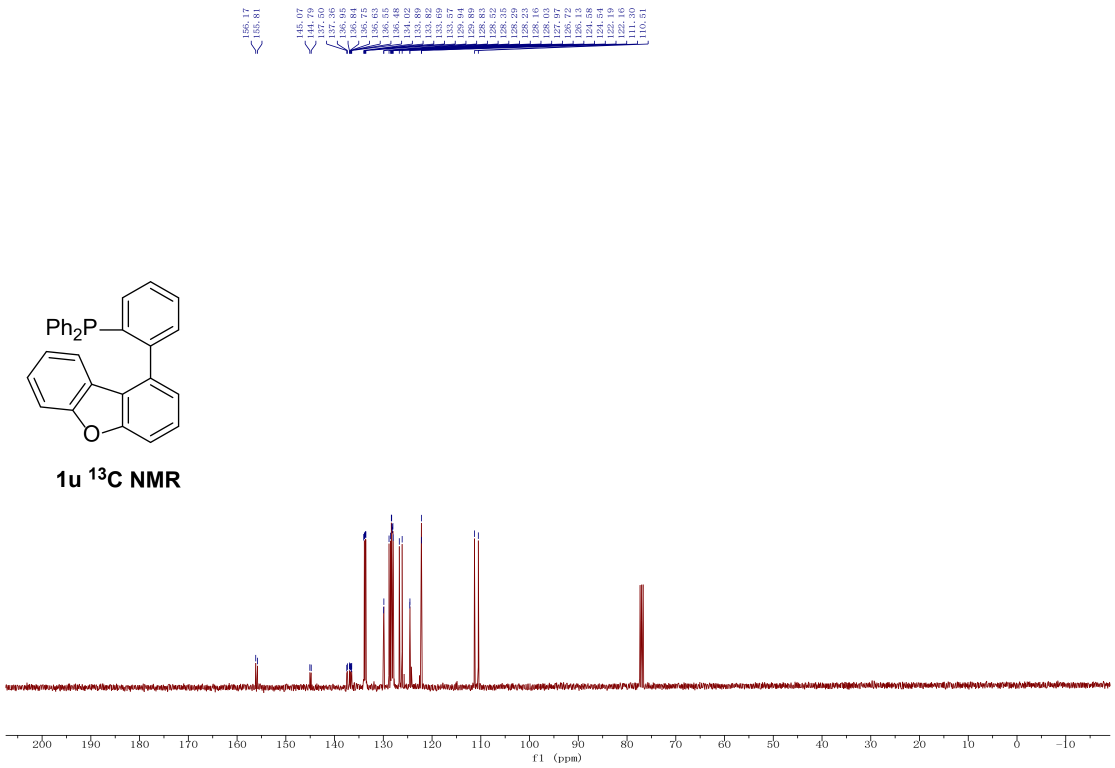

Supplementary Fig. 74. ^{13}C NMR spectra (101 MHz, CDCl3, 25 ºC) of **1u**

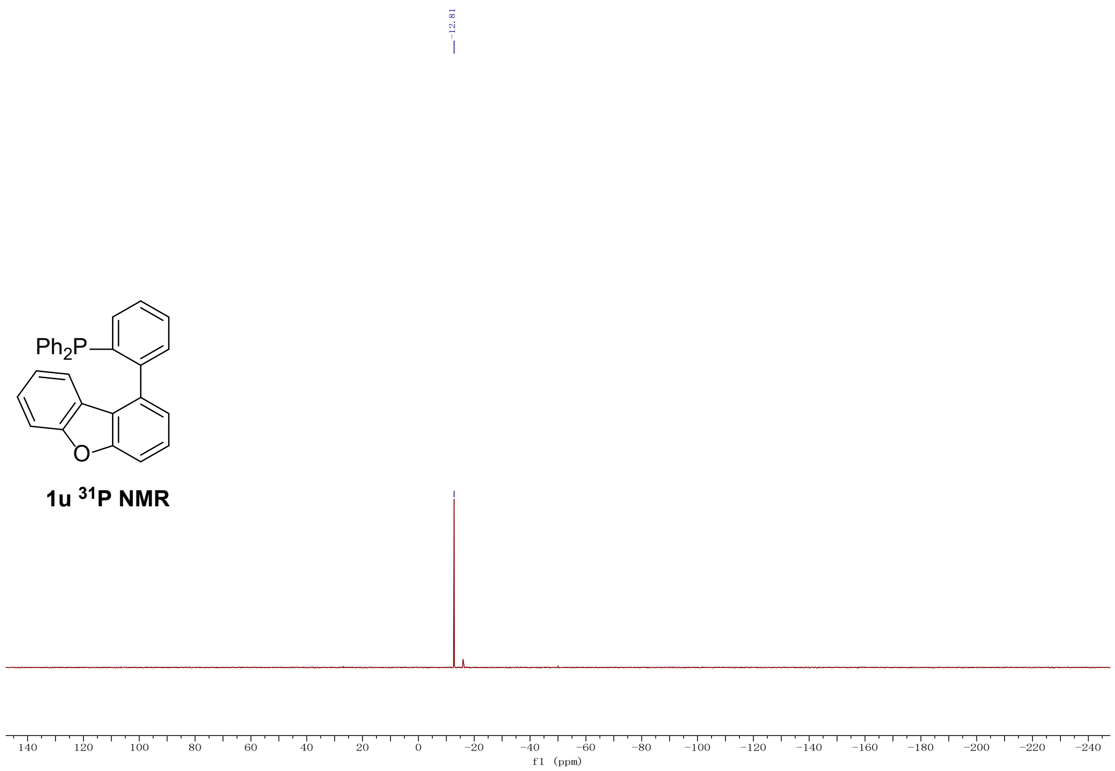

Supplementary Fig. 75 ^{31}P NMR spectra (162 MHz, $CDCl_3$, 25 ºC) of **1u**

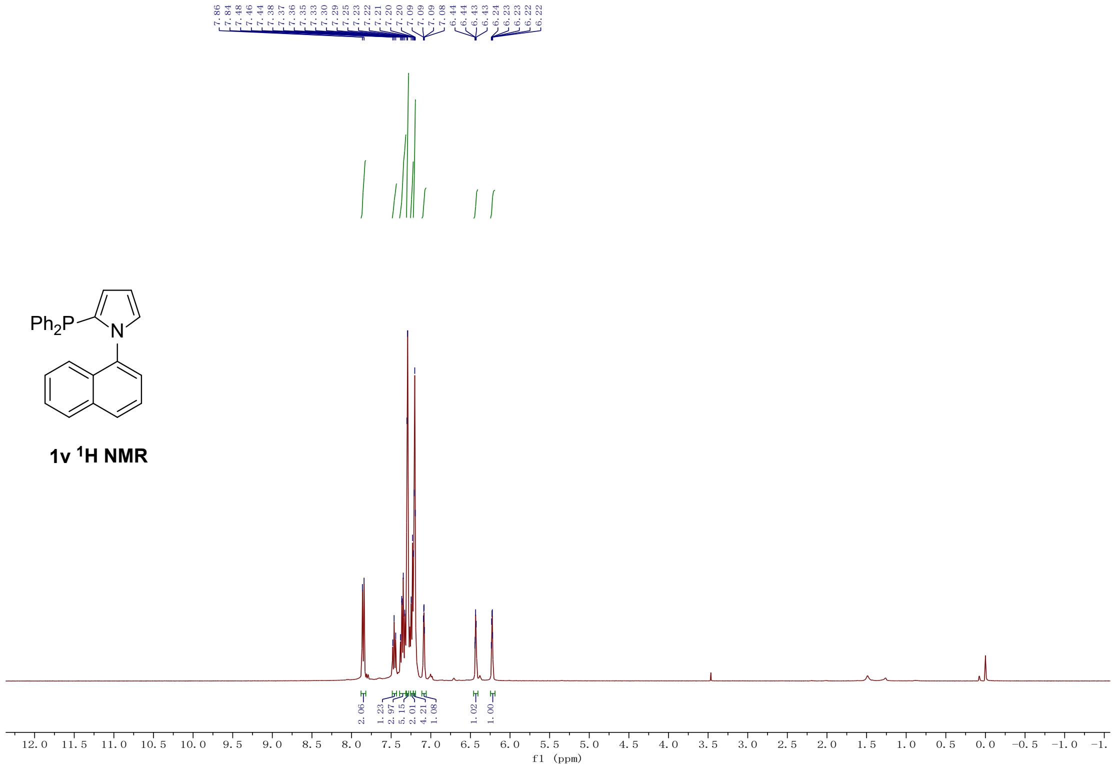

Supplementary Fig. 76. ^{1}H NMR spectra (400 MHz, $CDCl_3$, 25 ºC) of **1v**

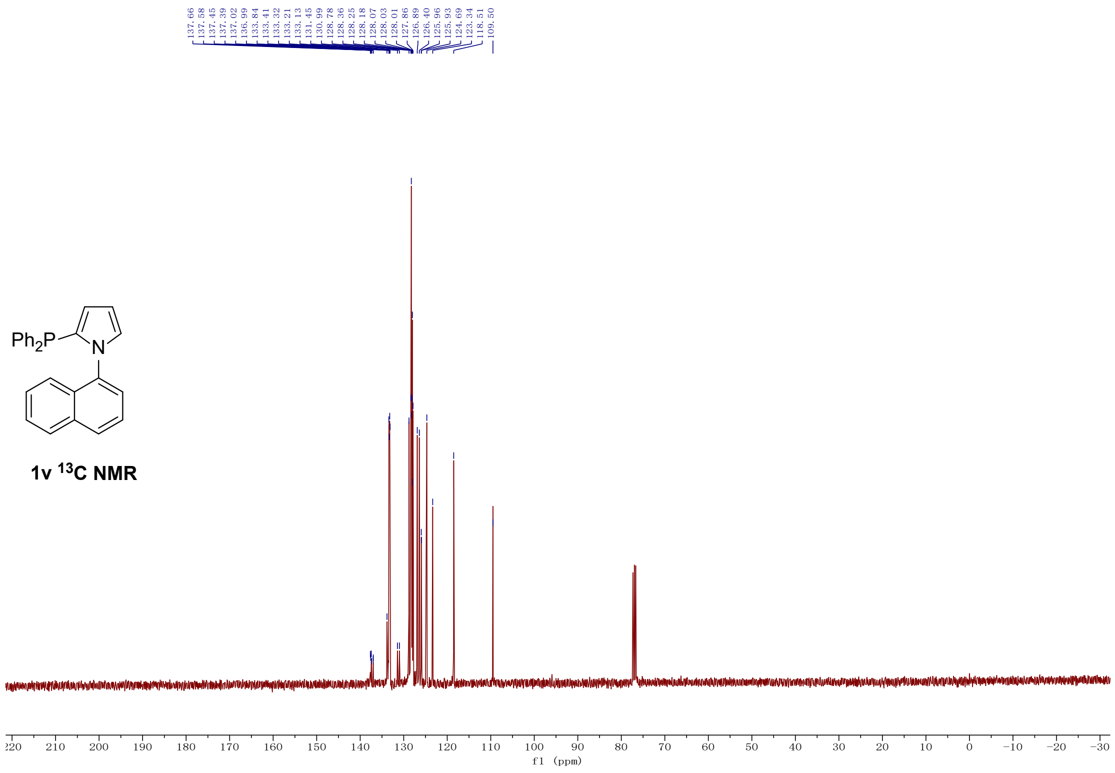

Supplementary Fig. 77. ^{13}C NMR spectra (101 MHz, $CDCl_3$, 25 °C) of **1v**

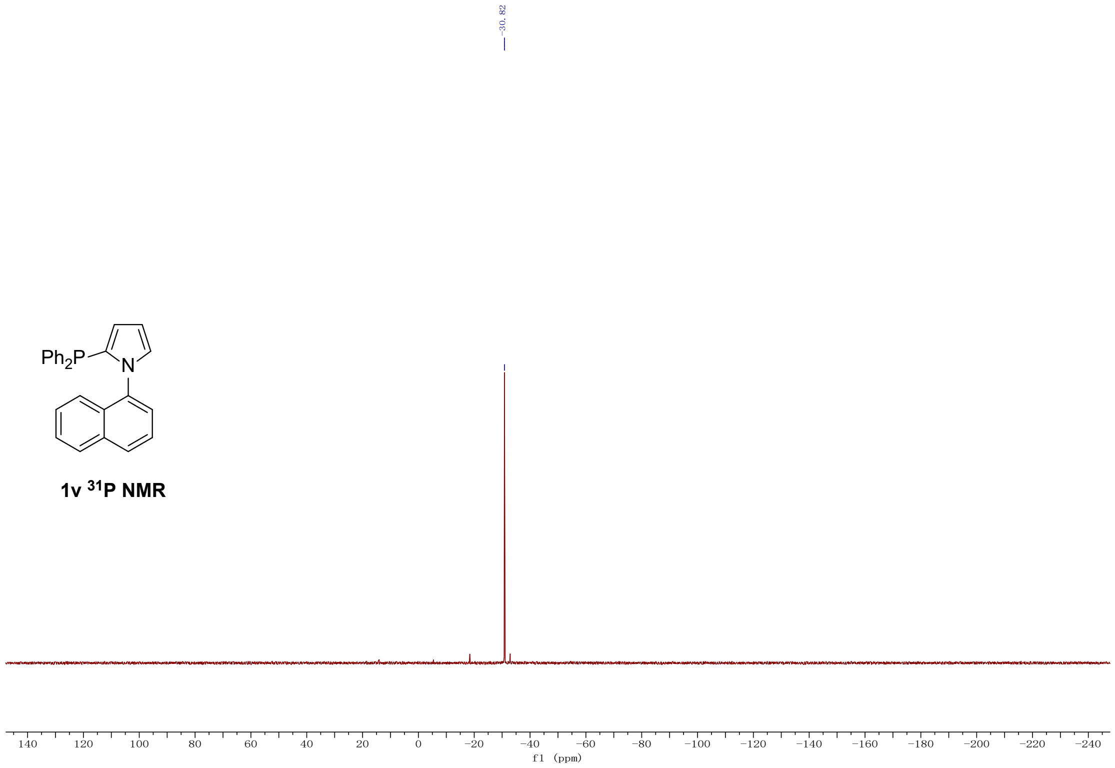

Supplementary Fig. 78 ^{31}P NMR spectra (162 MHz, $CDCl_3$, 25 °C) of **1v**

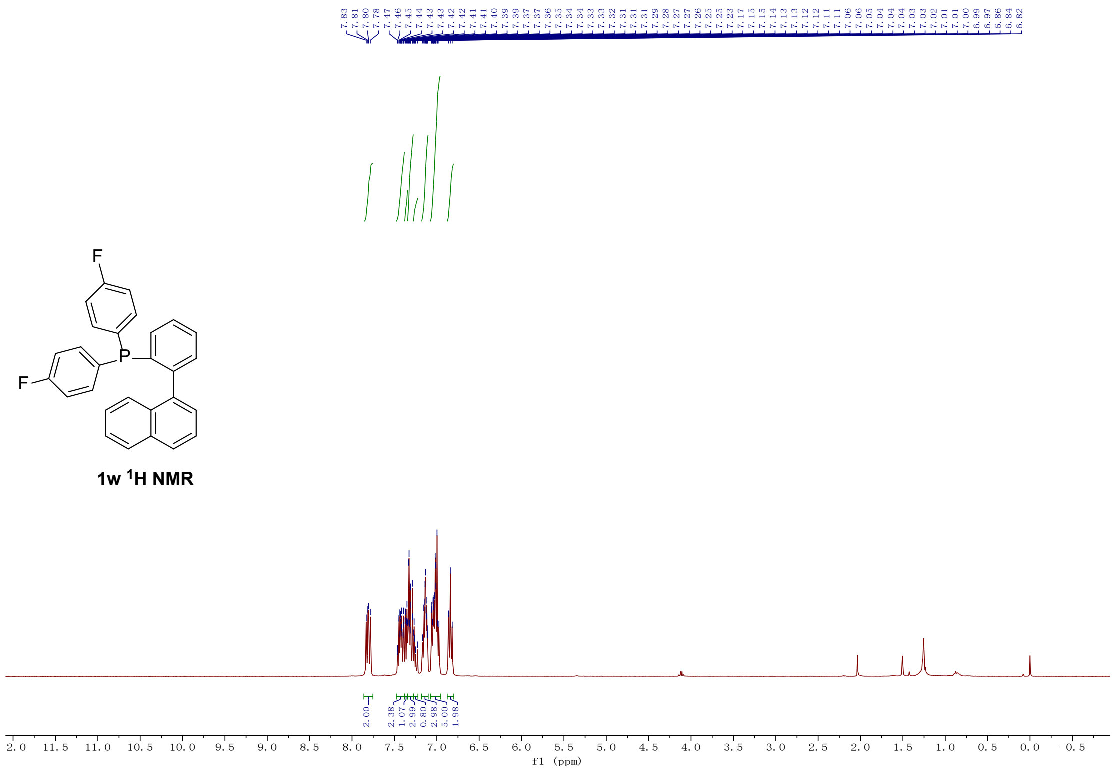

Supplementary Fig. 79. ^{1}H NMR spectra (400 MHz, $CDCl_3$, 25 ºC) of **1w**

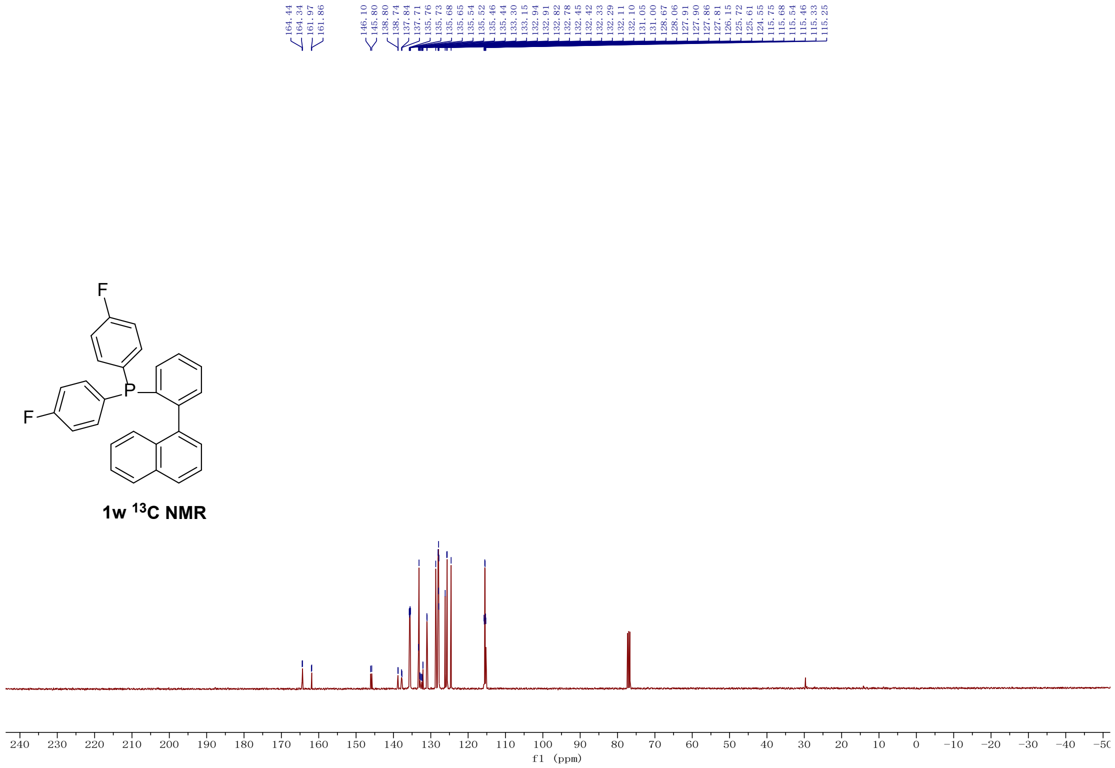

Supplementary Fig. 80. ^{13}C NMR spectra (101 MHz, $CDCl_3$, 25 ºC) of **1w**

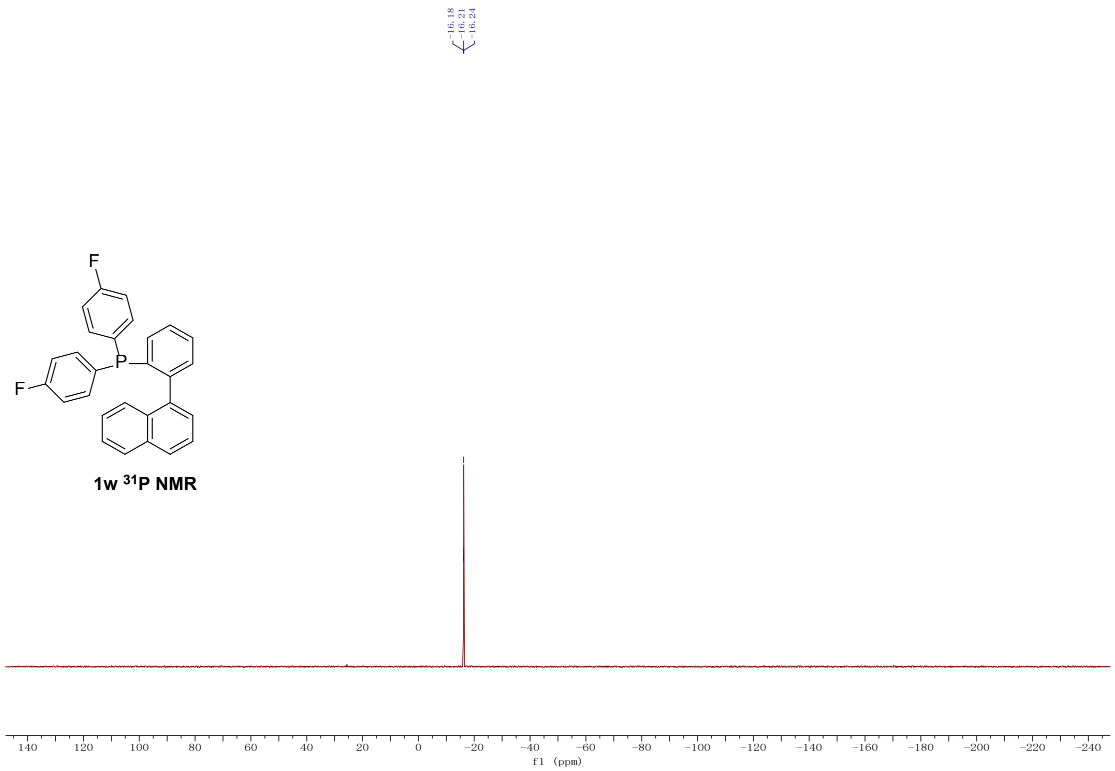

Supplementary Fig. 81 ^{31}P NMR spectra (162 MHz, $CDCl_3$, 25 ºC) of **1w**

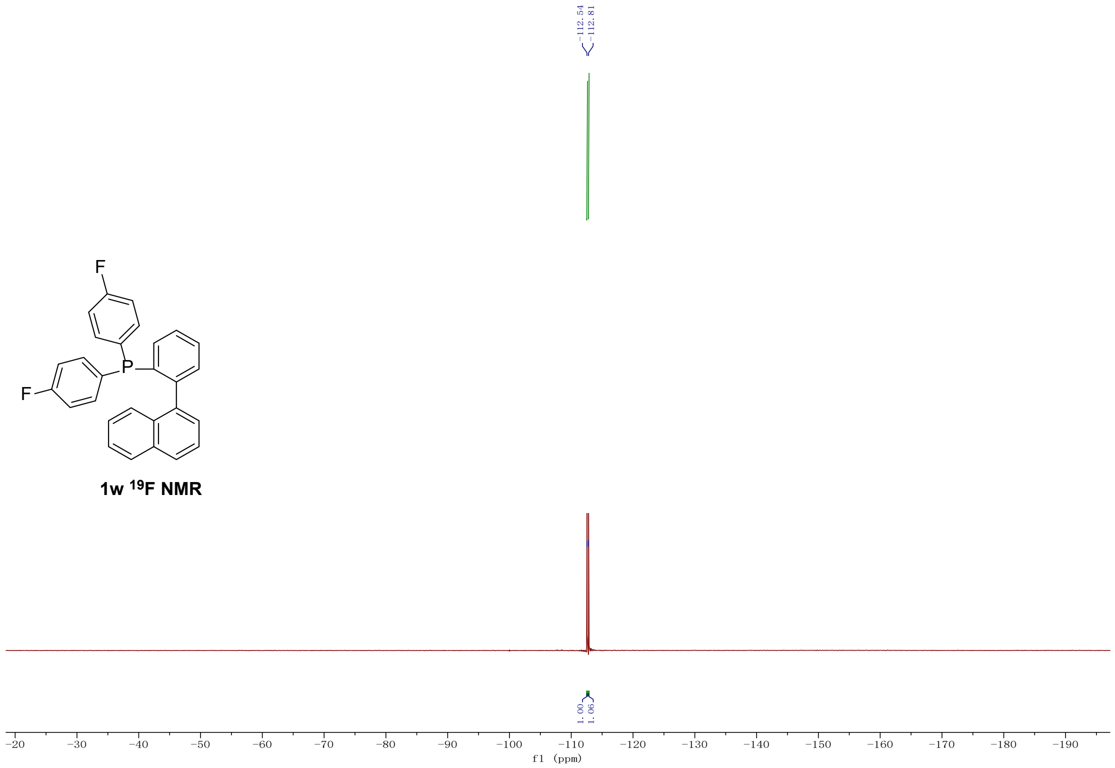

Supplementary Fig. 82 ^{19}F NMR spectra (376 MHz, $CDCl_3$, 25 ºC) of **1w**

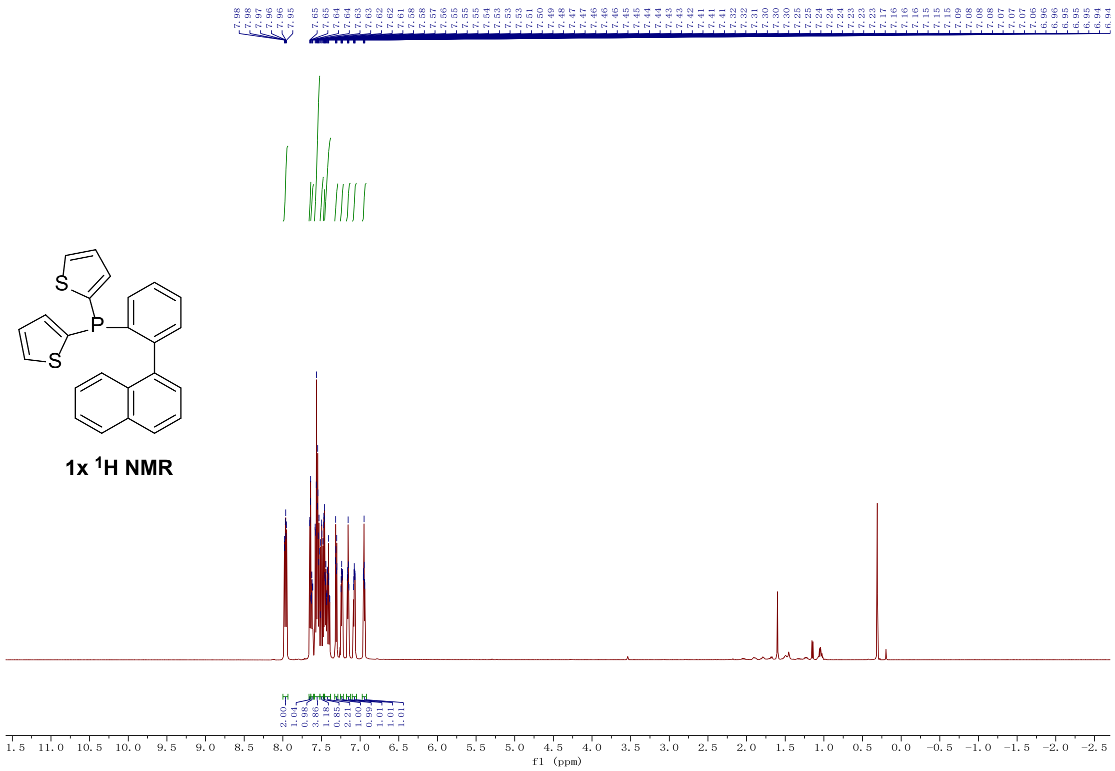

Supplementary Fig. 83. ^{1}H NMR spectra (500 MHz, $CDCl_3$, 25 °C) of **1x**

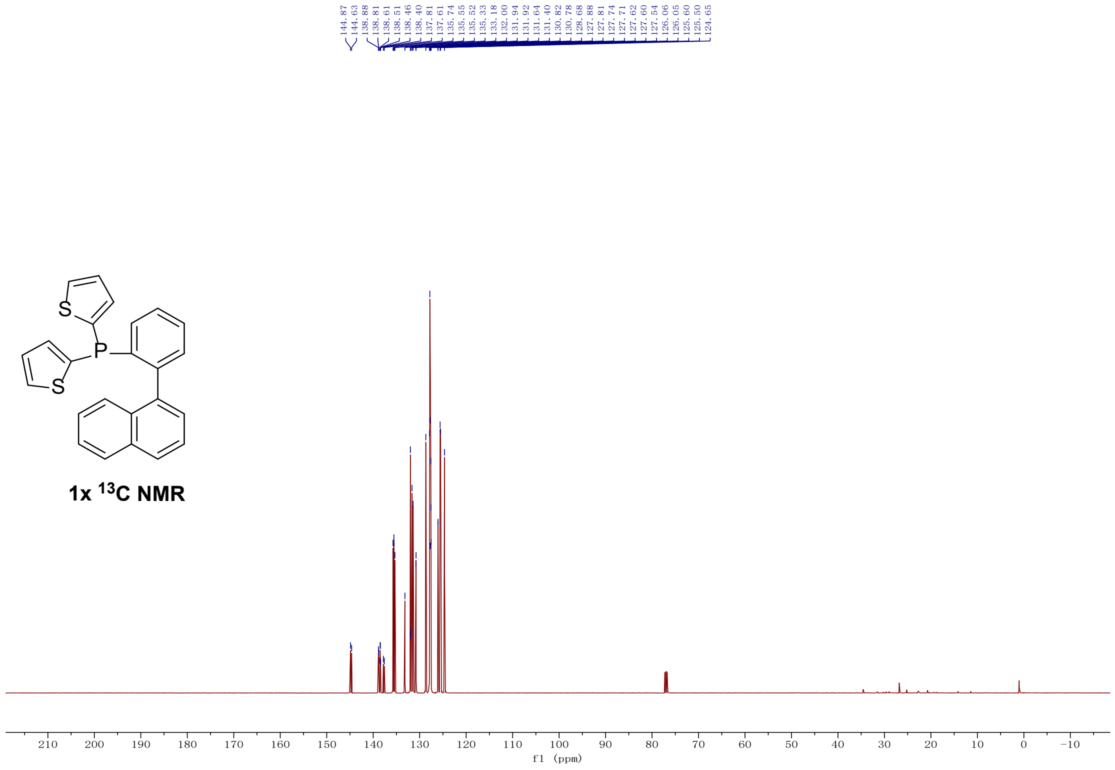

Supplementary Fig. 84. ^{13}C NMR spectra (126 MHz, $CDCl_3$, 25 °C) of **1x**

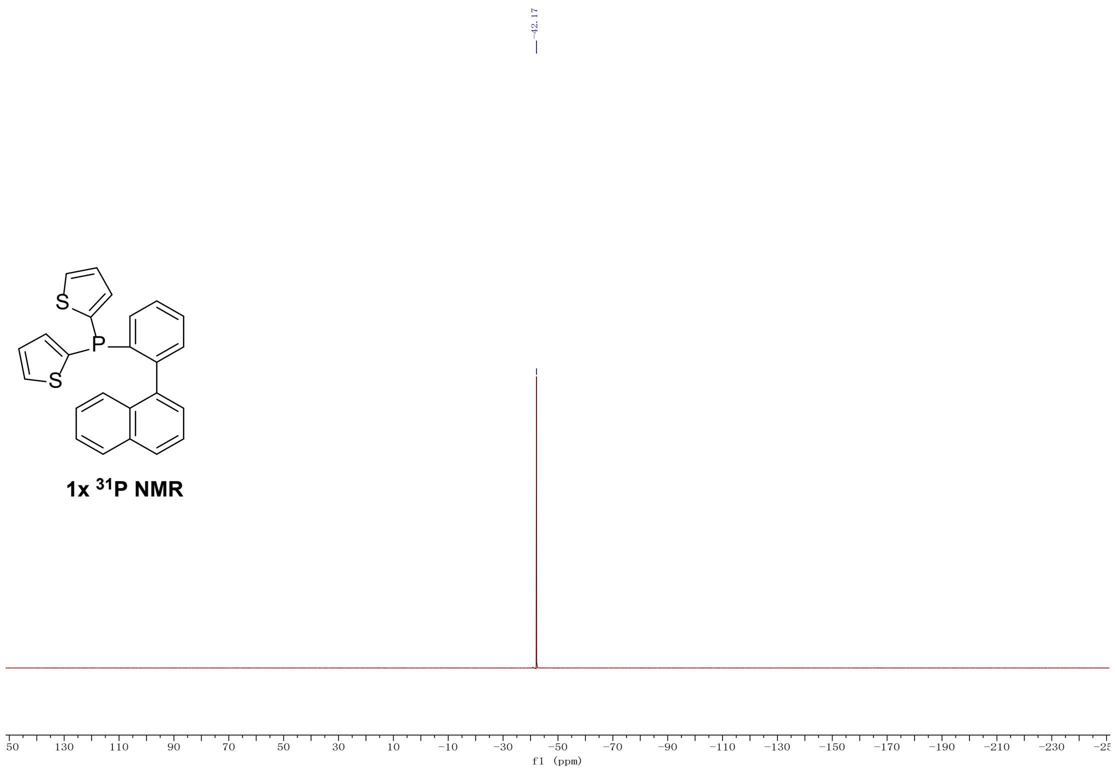

Supplementary Fig. 85 ^{31}P NMR spectra (162 MHz, $CDCl_3$, 25 ºC) of **1x**

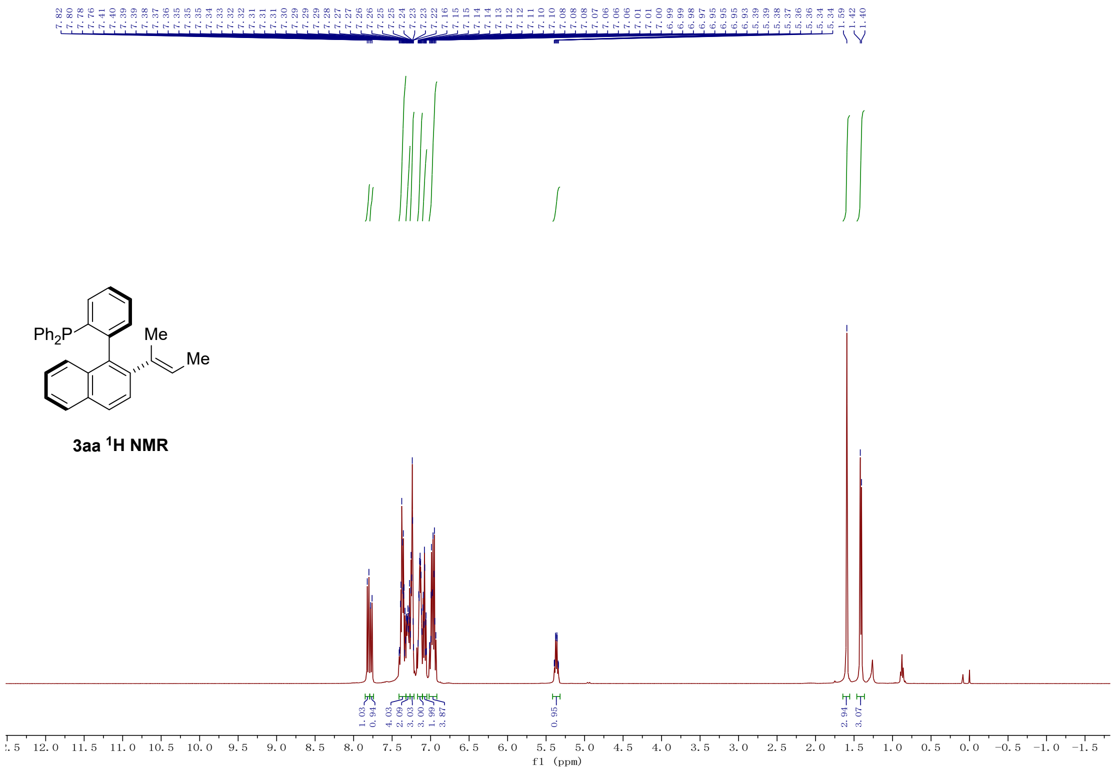

Supplementary Fig. 86. ^{1}H NMR spectra (400 MHz, $CDCl_3$, 25 ºC) of **3aa**

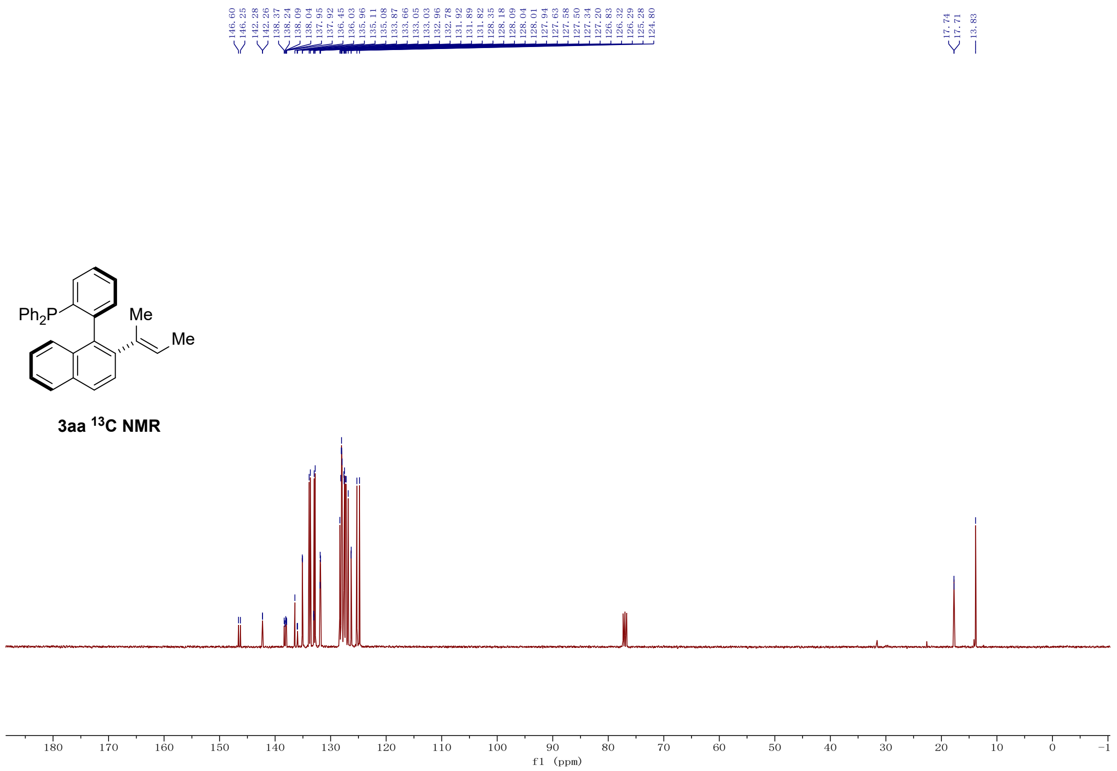

Supplementary Fig. 87. ^{13}C NMR spectra (101 MHz, CDCl3, 25 °C) of **3aa**

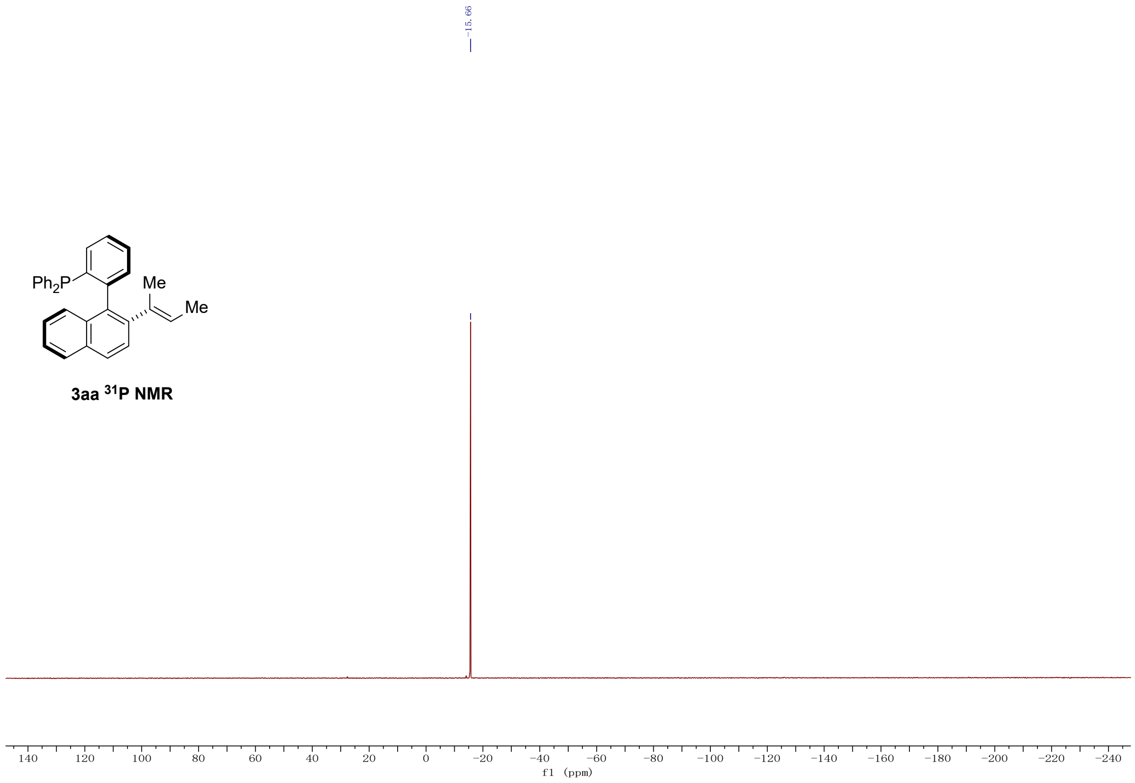

Supplementary Fig. 88 ^{31}P NMR spectra (162 MHz, CDCl3, 25 °C) of **3aa**

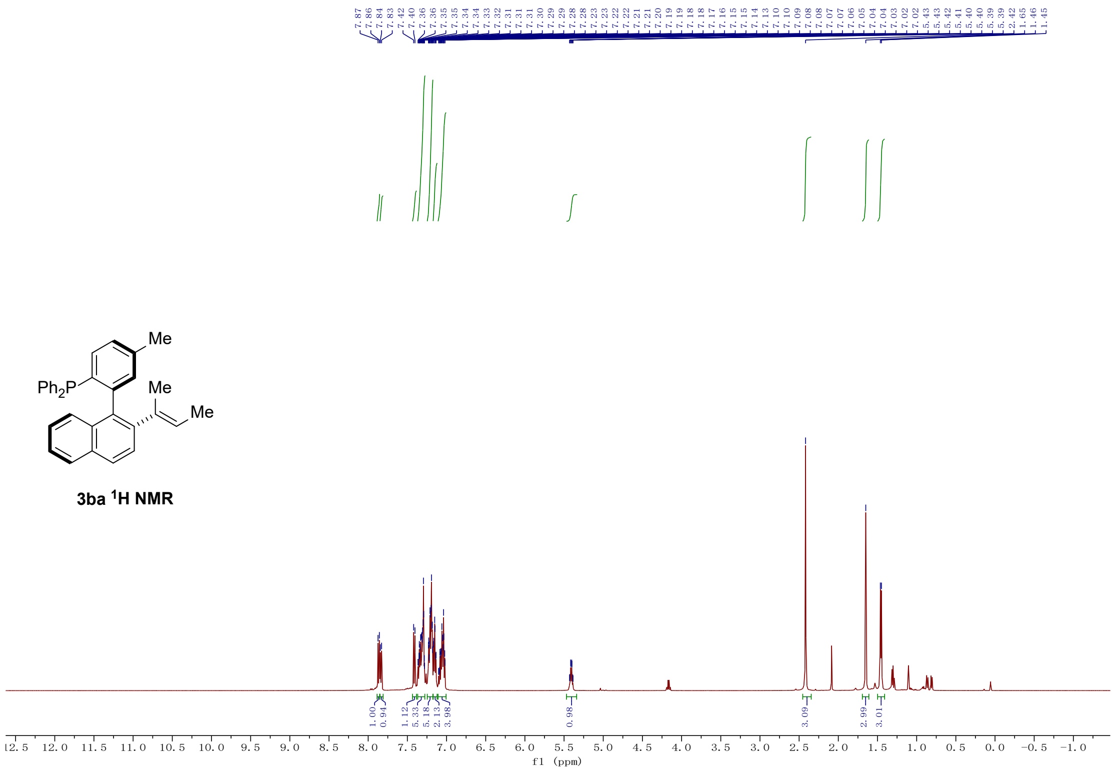

Supplementary Fig. 89. ^{1}H NMR spectra (500 MHz, $CDCl_3$, 25 ºC) of **3ba**

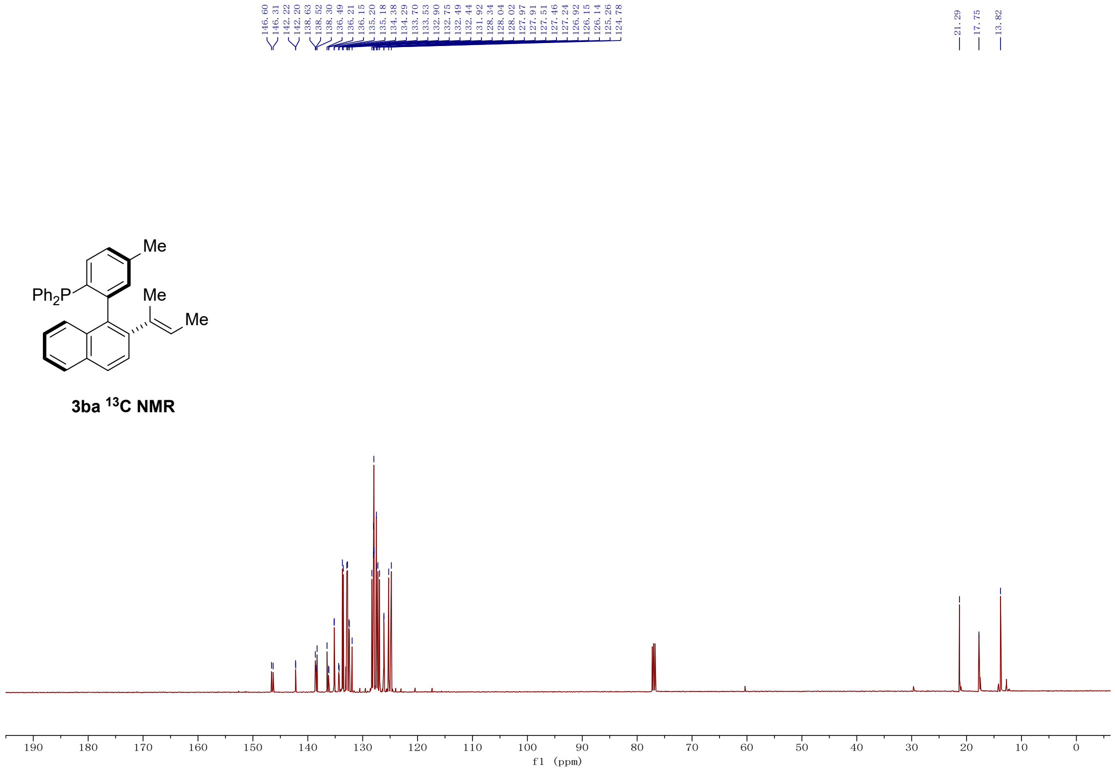

Supplementary Fig. 90. ^{13}C NMR spectra (126 MHz, $CDCl_3$, 25 ºC) of **3ba**

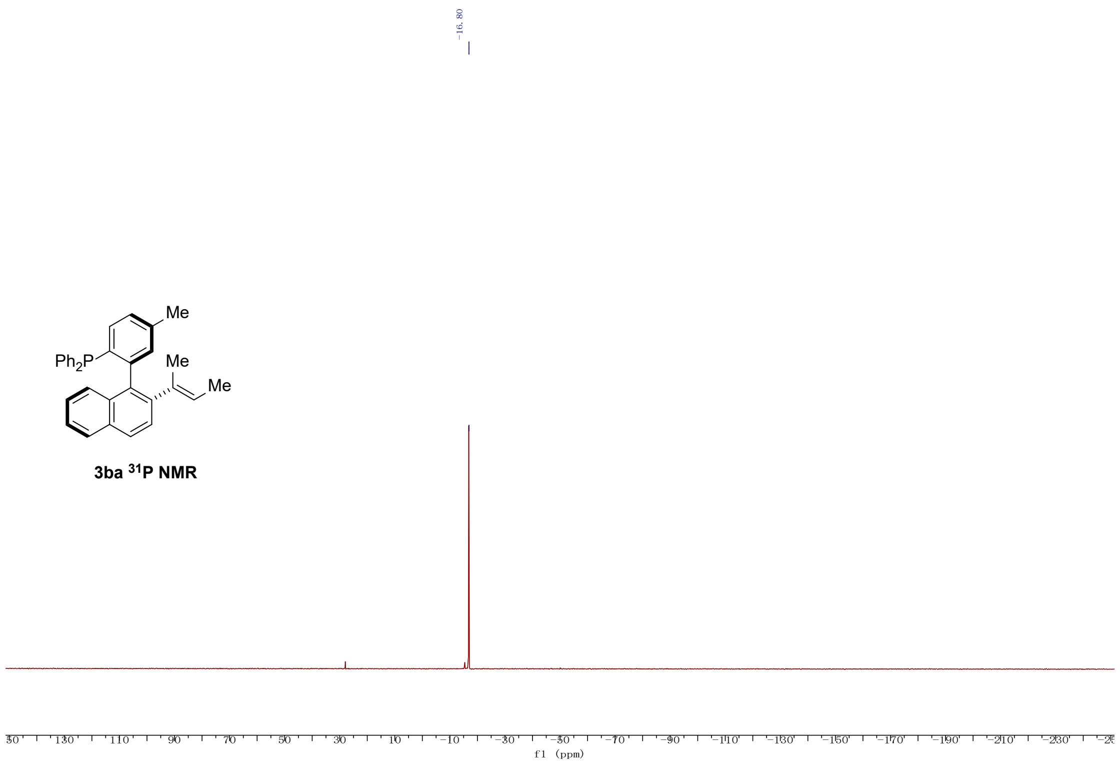

Supplementary Fig. 91 ^{31}P NMR spectra (202 MHz, CDCl$_3$, 25 °C) of **3ba**

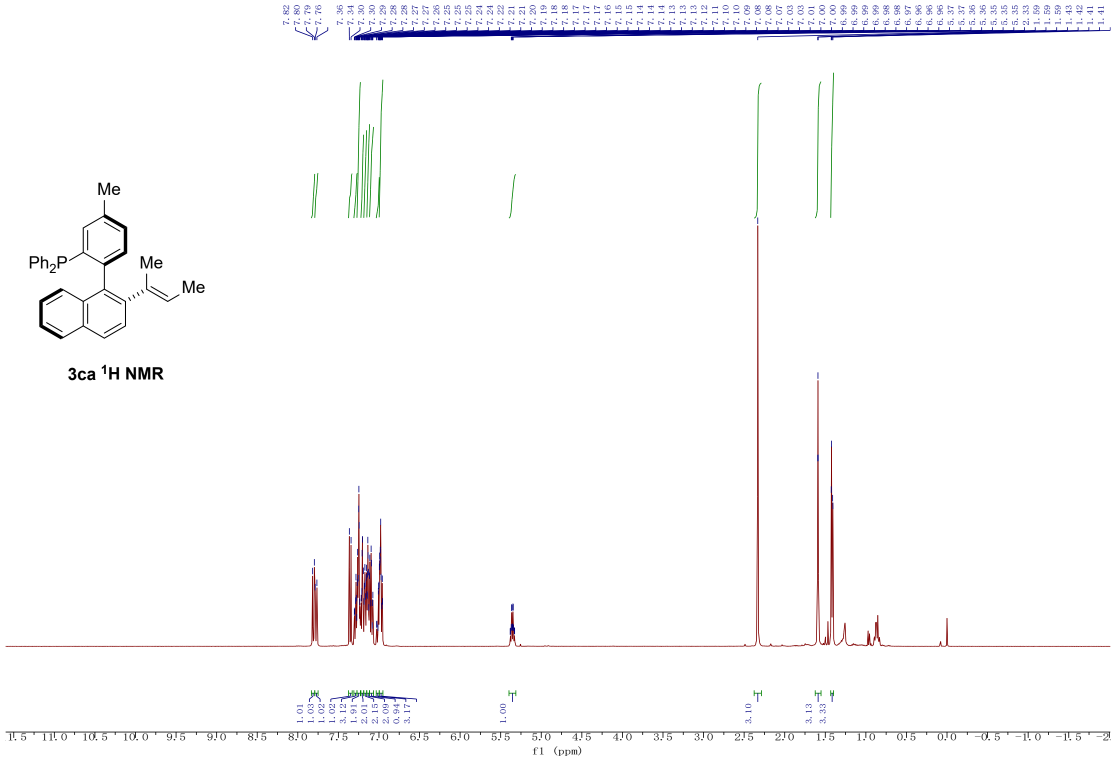

Supplementary Fig. 92. ^{1}H NMR spectra (400 MHz, CDCl$_3$, 25 °C) of **3ca**

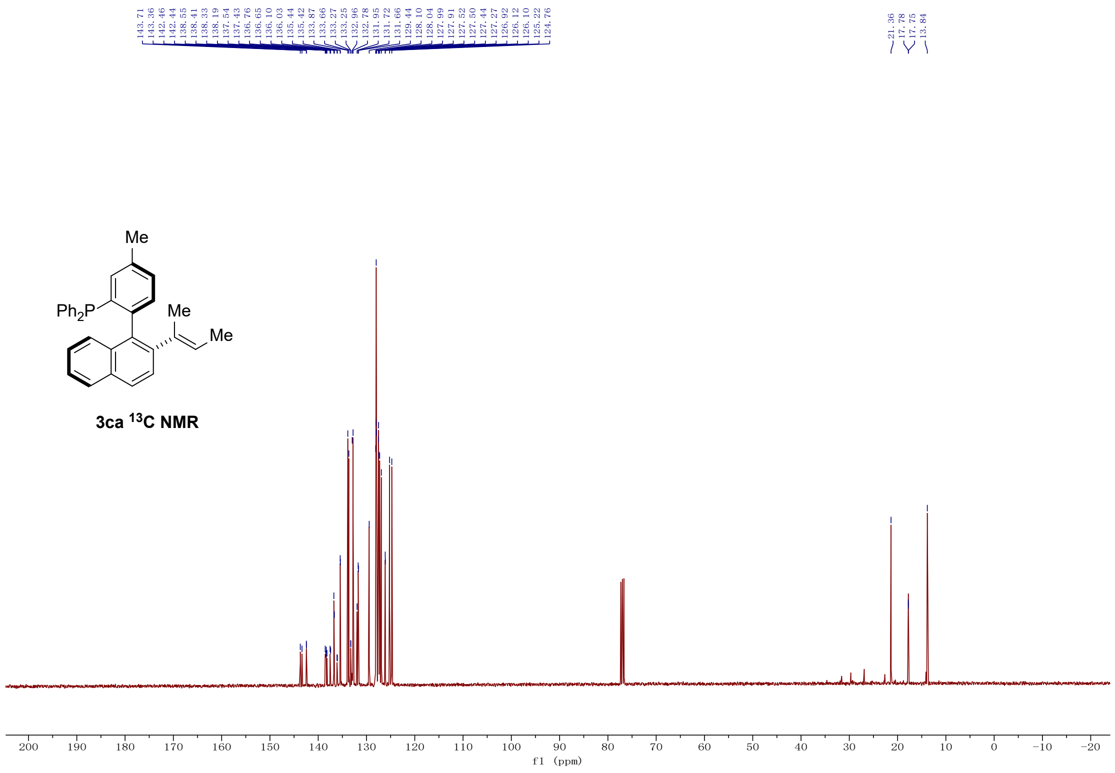

Supplementary Fig. 93. ^{13}C NMR spectra (101 MHz, $CDCl_3$, 25 °C) of **3ca**

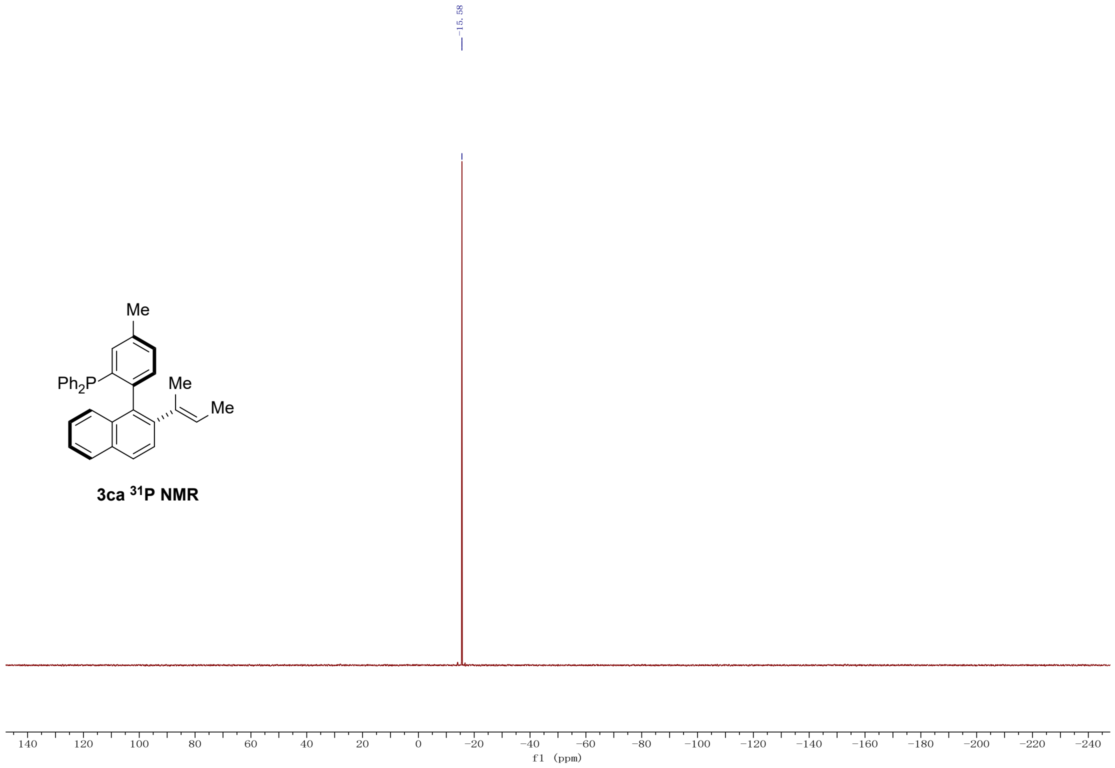

Supplementary Fig. 94. ^{31}P NMR spectra (202 MHz, $CDCl_3$, 25 °C) of **3ca**

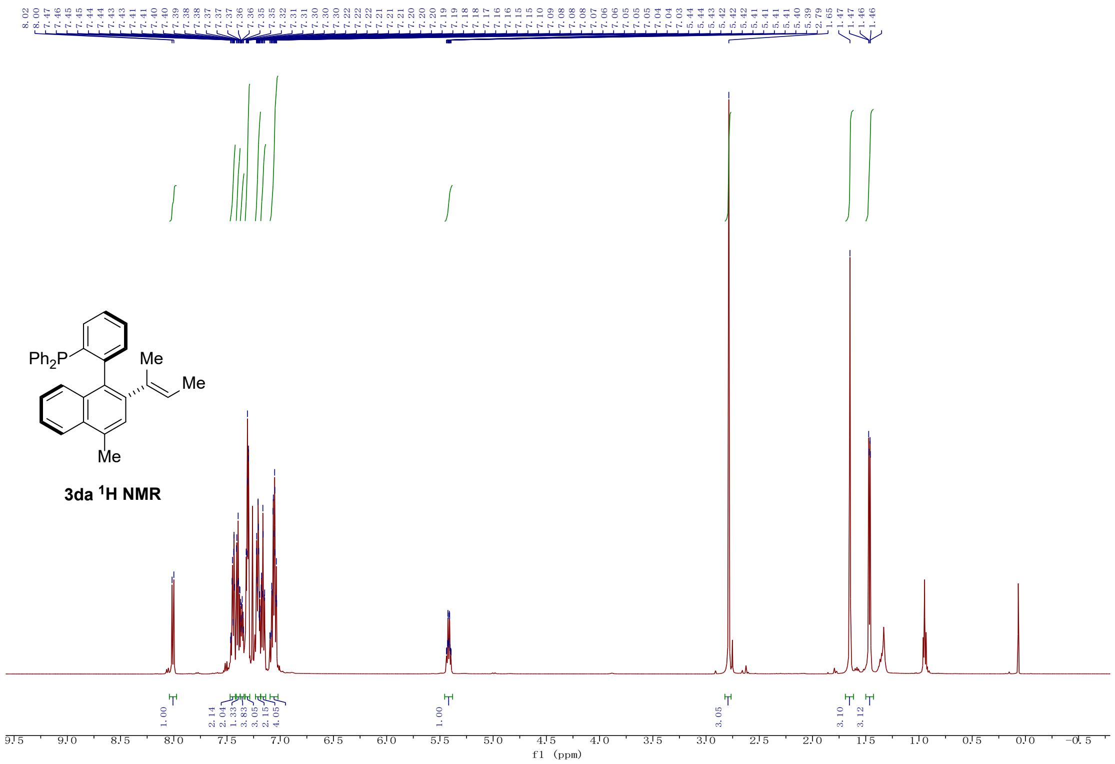

Supplementary Fig. 95. ^{1}H NMR spectra (500 MHz, $CDCl_3$, 25 °C) of **3da**

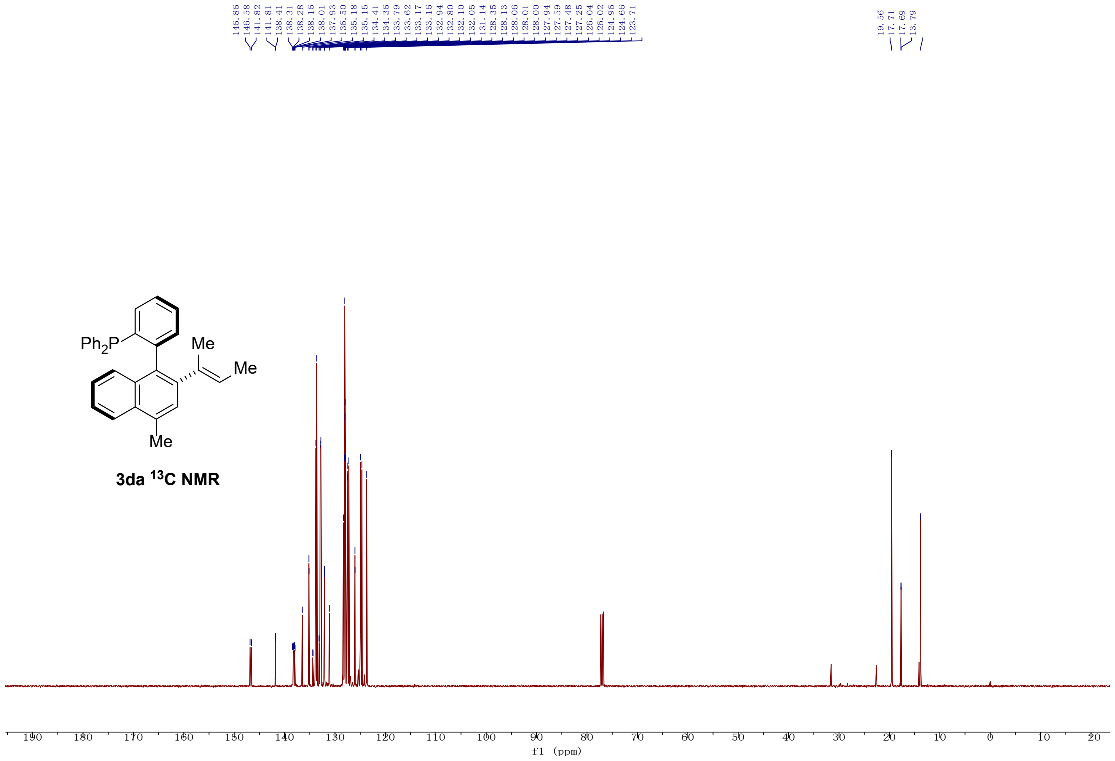

Supplementary Fig. 96. ^{13}C NMR spectra (126 MHz, $CDCl_3$, 25 °C) of **3da**

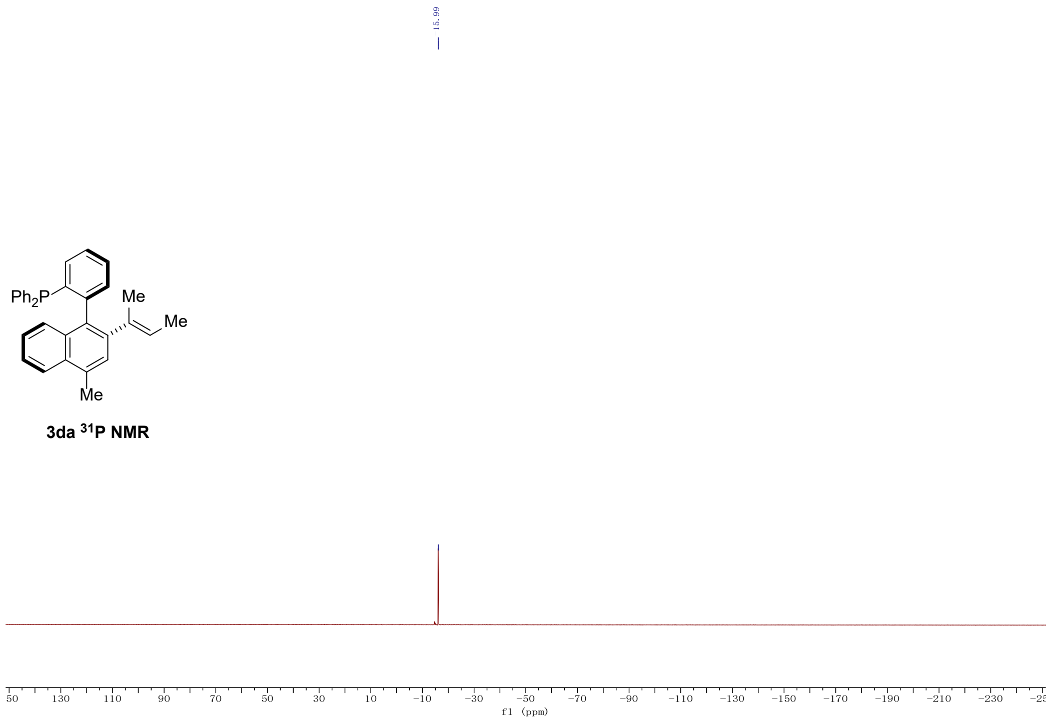

Supplementary Fig. 97. ^{31}P NMR spectra (202 MHz, $CDCl_3$, 25 °C) of **3da**

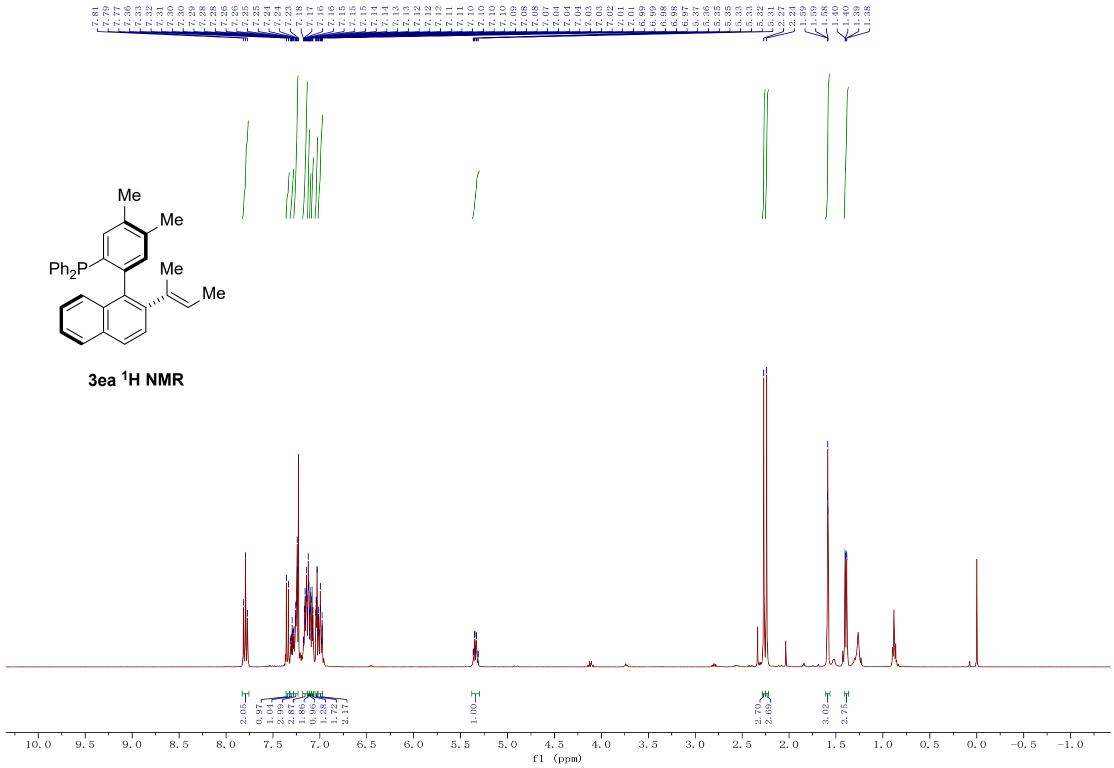

Supplementary Fig. 98. ^{1}H NMR spectra (400 MHz, $CDCl_3$, 25 °C) of **3ea**

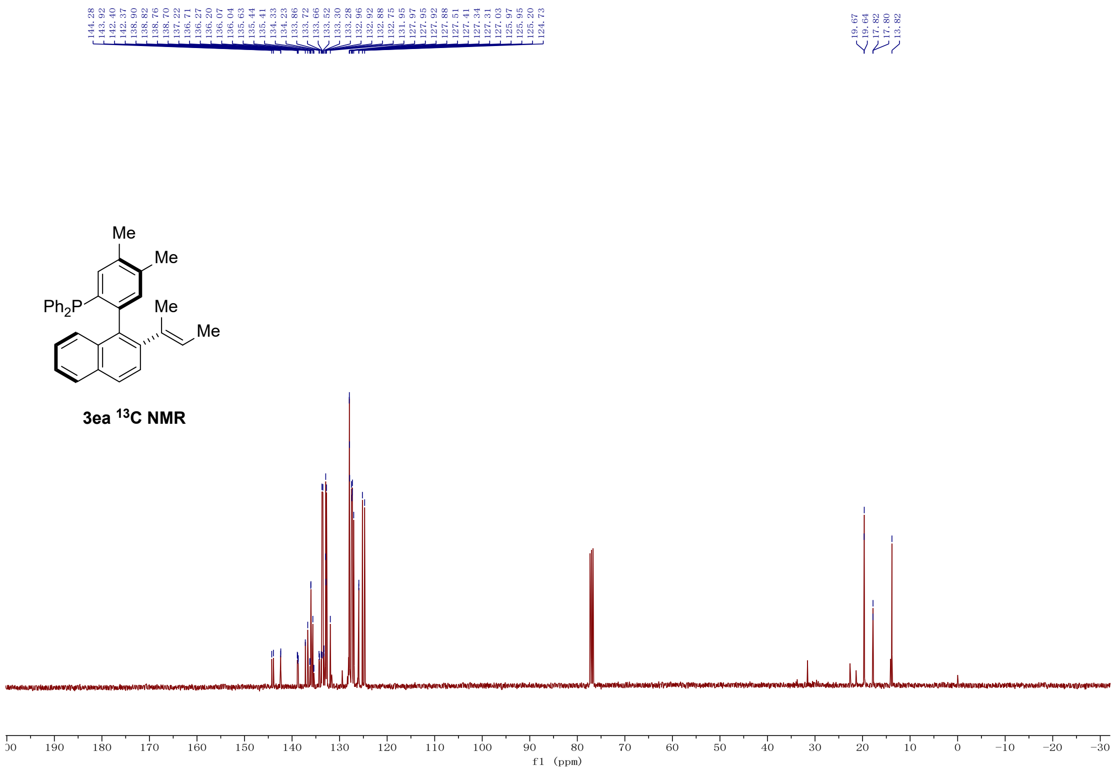

Supplementary Fig. 99. ^{13}C NMR spectra (101 MHz, $CDCl_3$, 25 ºC) of **3ea**

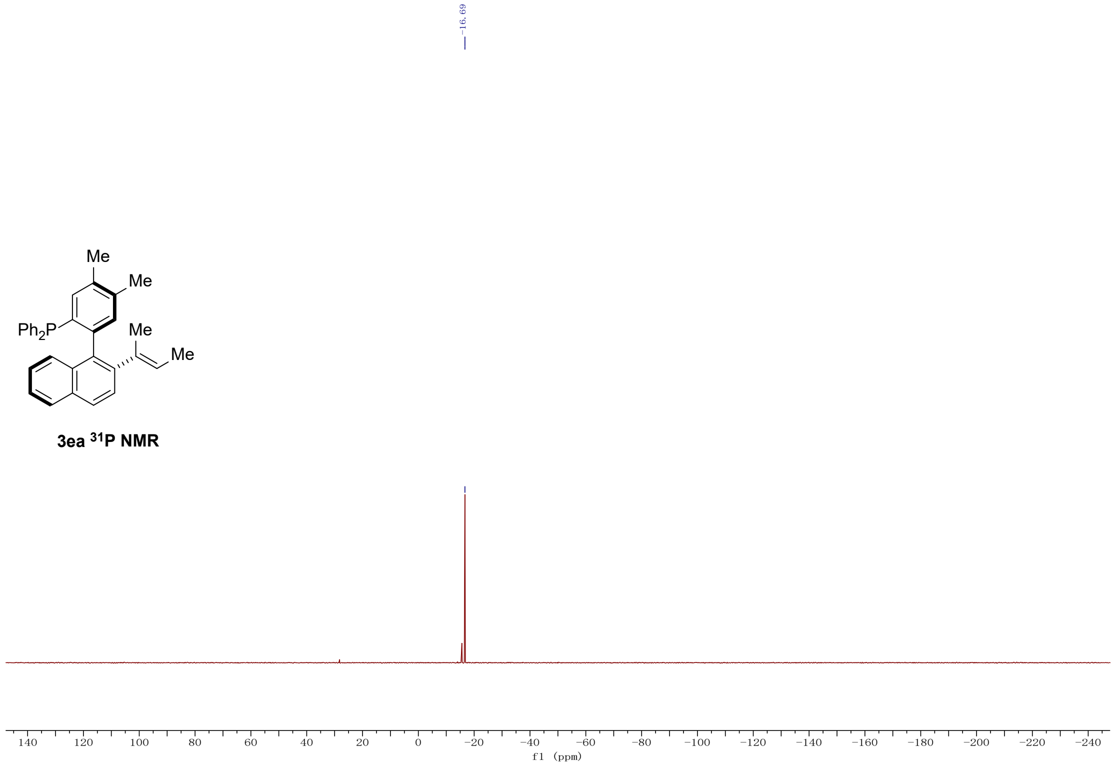

Supplementary Fig. 100. ^{31}P NMR spectra (162 MHz, $CDCl_3$, 25 ºC) of **3ea**

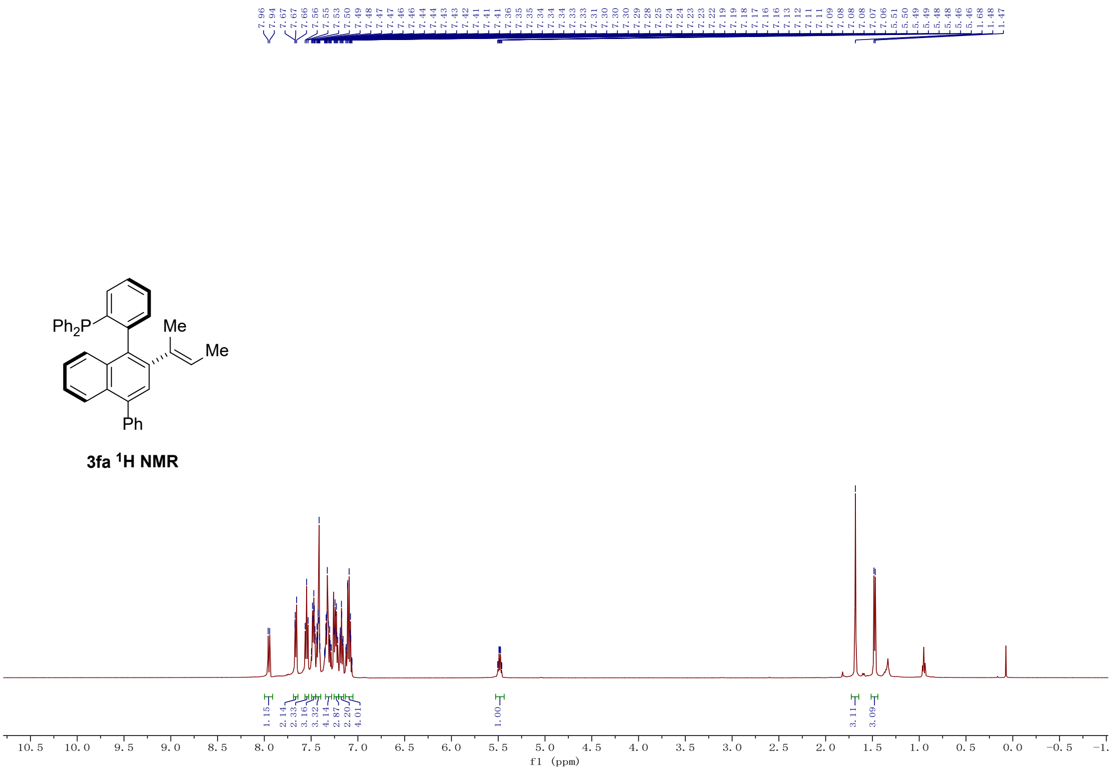

Supplementary Fig. 101. ^{1}H NMR spectra (500 MHz, CDCl3, 25 ºC) of **3fa**

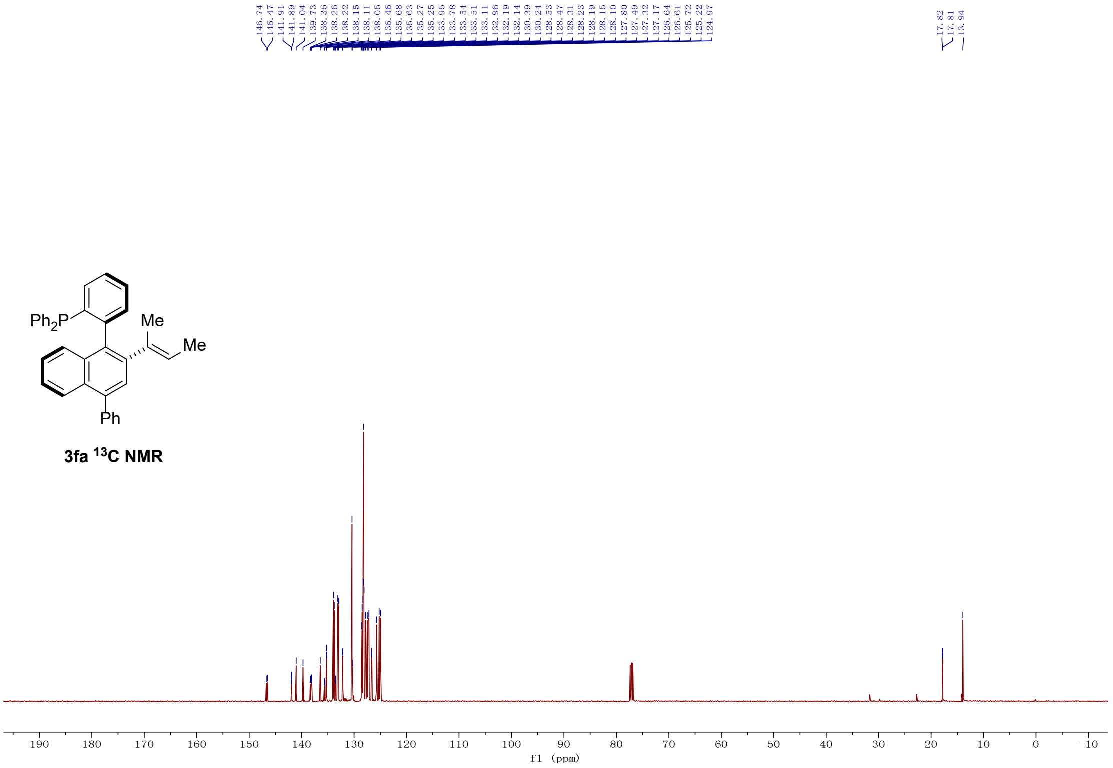

Supplementary Fig. 102. ^{13}C NMR spectra (126 MHz, CDCl3, 25 ºC) of **3fa**

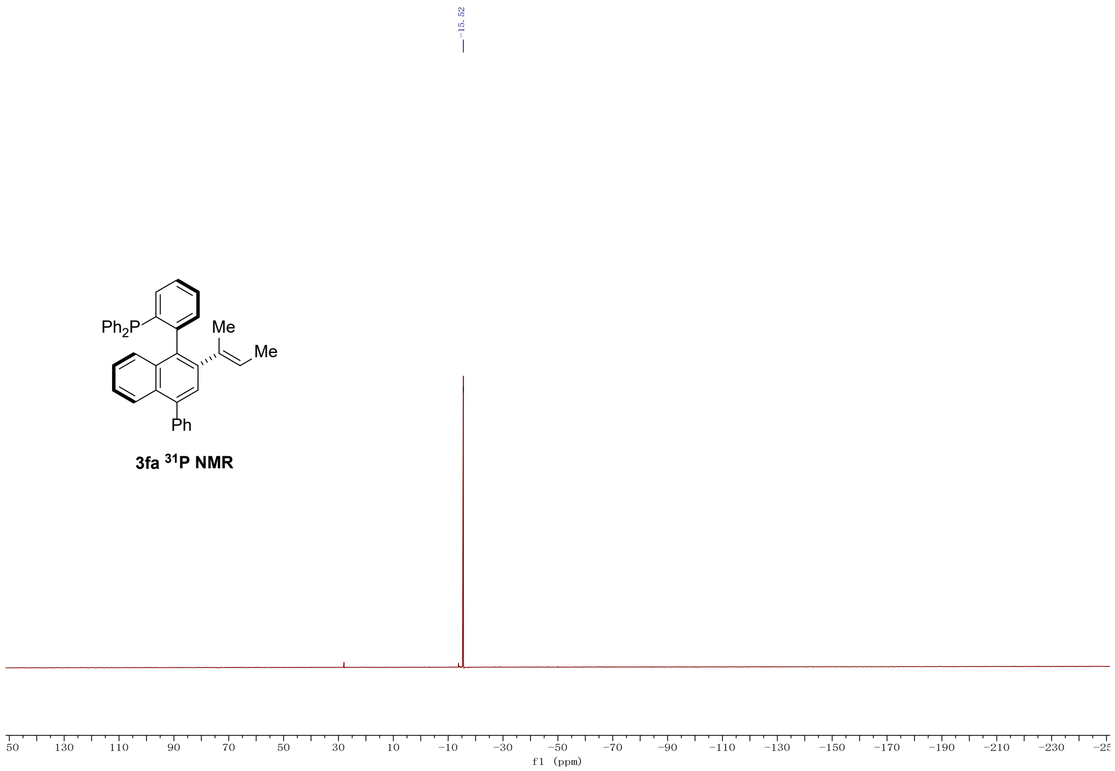

Supplementary Fig. 103. ^{31}P NMR spectra (202 MHz, $CDCl_3$, 25 ºC) of **3fa**

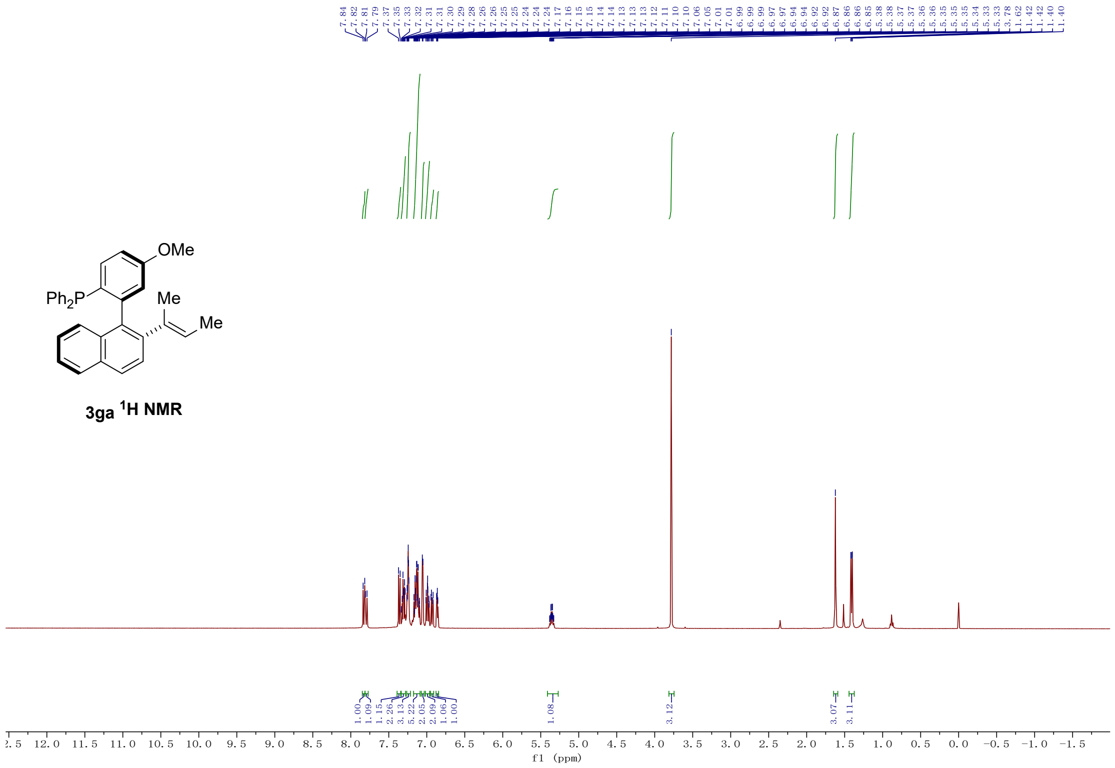

Supplementary Fig. 104. ^{1}H NMR spectra (400 MHz, $CDCl_3$, 25 ºC) of **3ga**

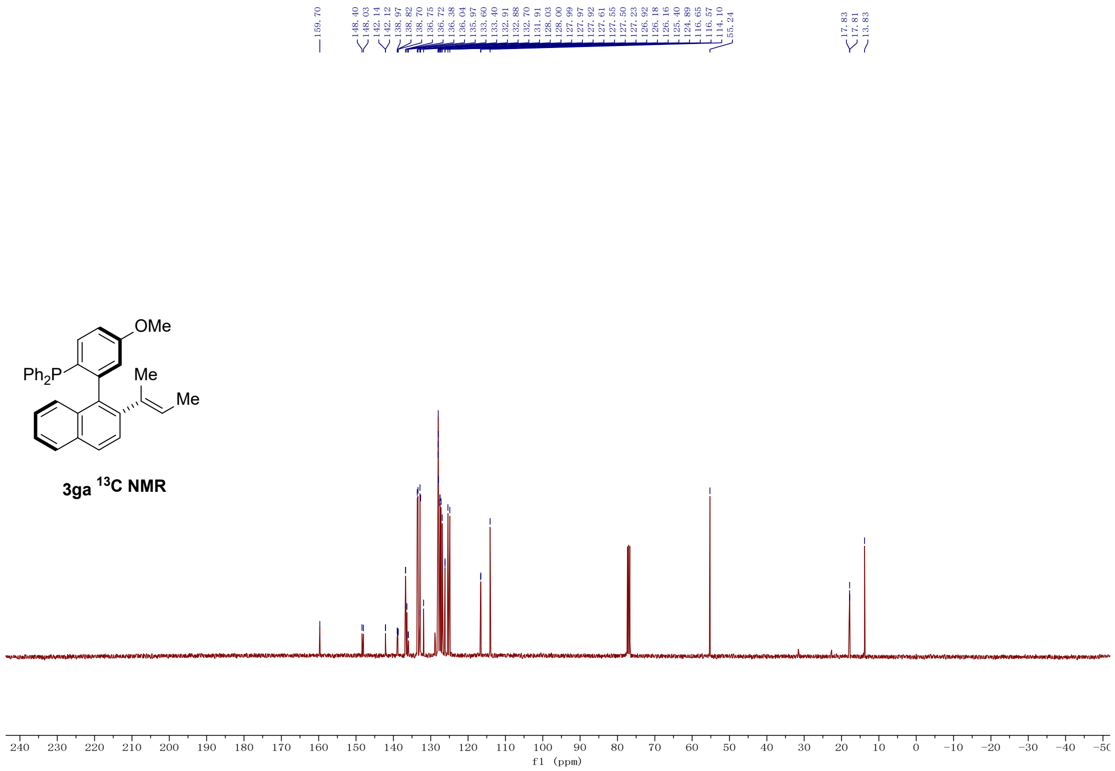

Supplementary Fig. 105. ^{13}C NMR spectra (101 MHz, $CDCl_3$, 25 ºC) of **3ga**

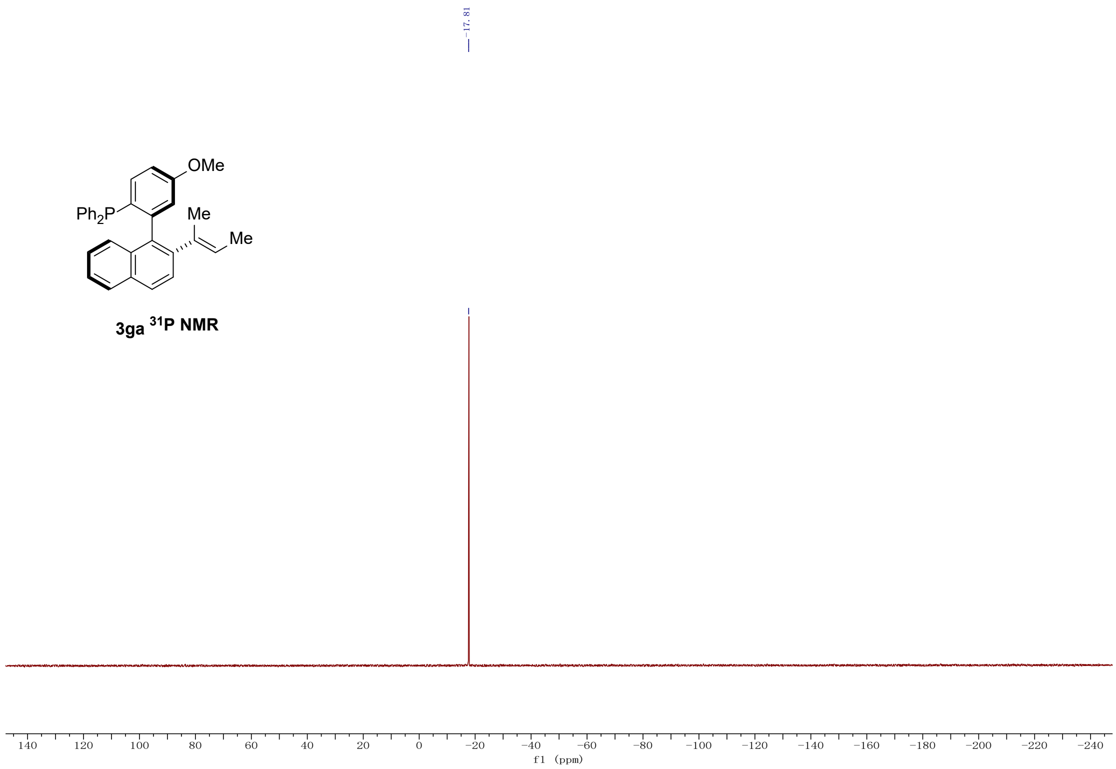

Supplementary Fig. 106. ^{31}P NMR spectra (162 MHz, $CDCl_3$, 25 ºC) of **3ga**

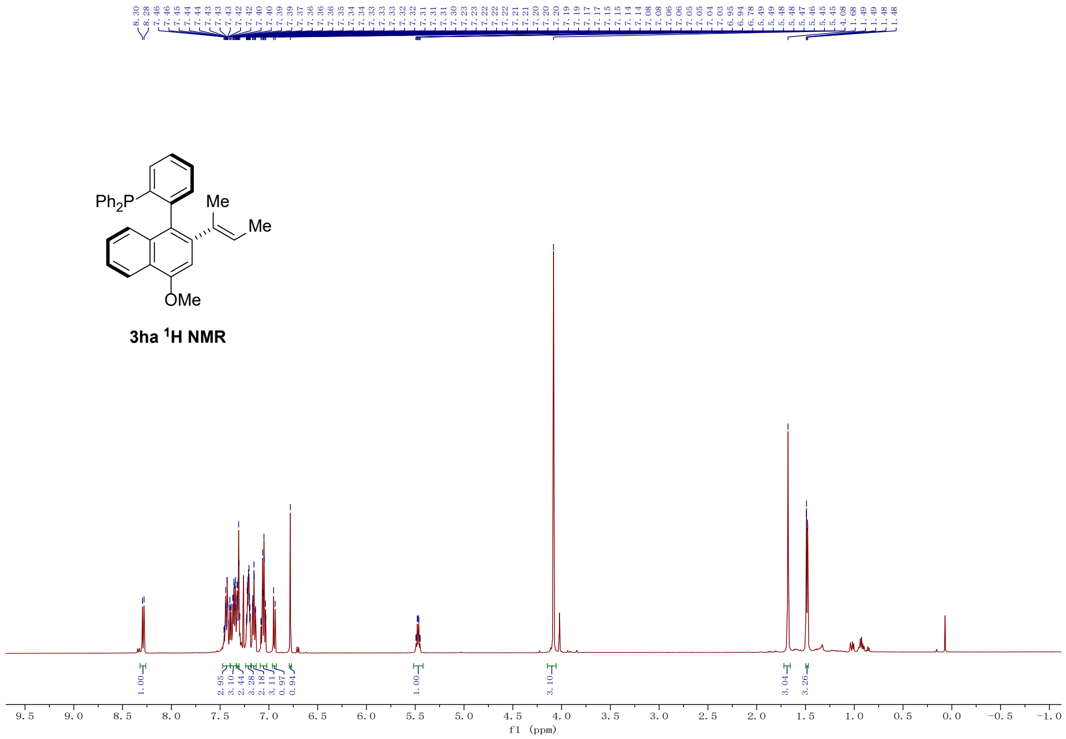

Supplementary Fig. 107. ^{1}H NMR spectra (500 MHz, $CDCl_3$, 25 ºC) of **3ha**

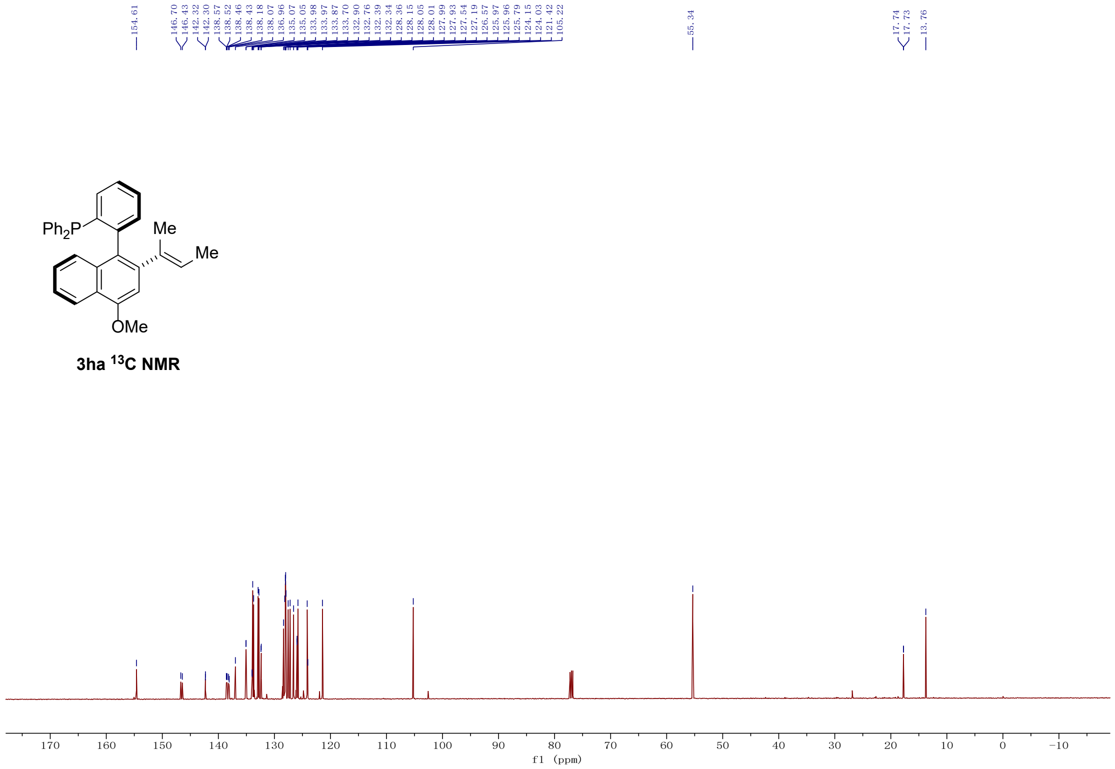

Supplementary Fig. 108. ^{13}C NMR spectra (126 MHz, $CDCl_3$, 25 ºC) of **3ha**

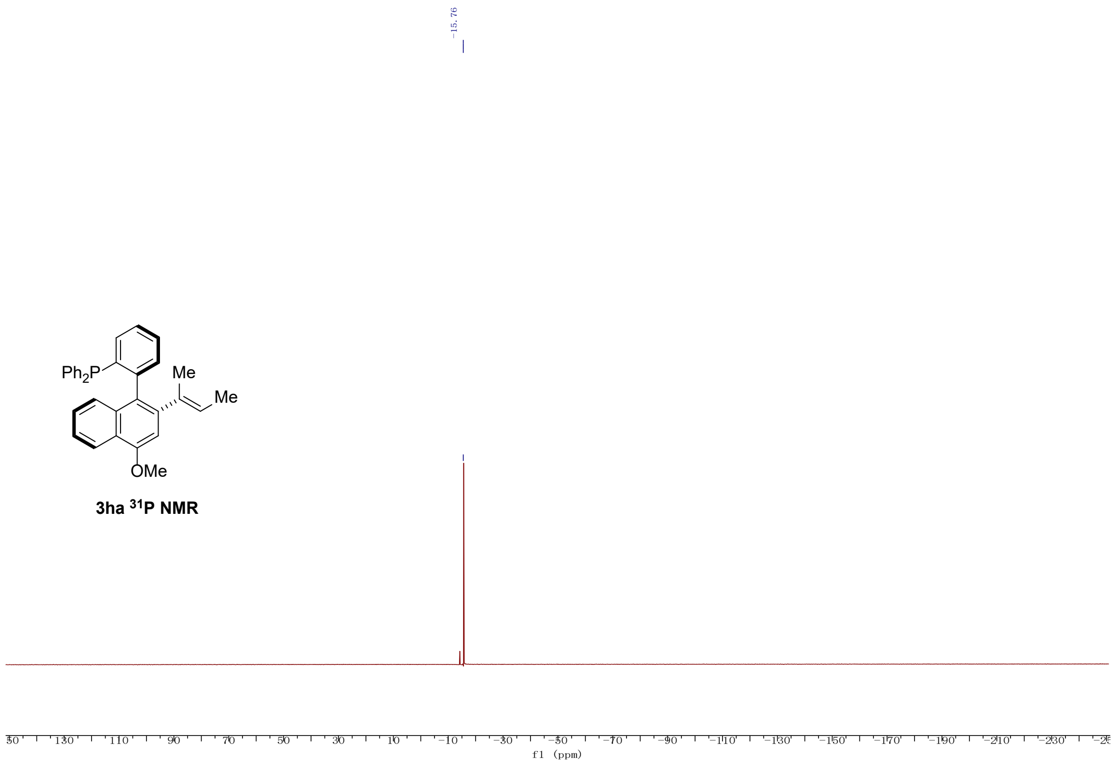

Supplementary Fig. 109. ^{31}P NMR spectra (202 MHz, $CDCl_3$, 25 ºC) of **3ha**

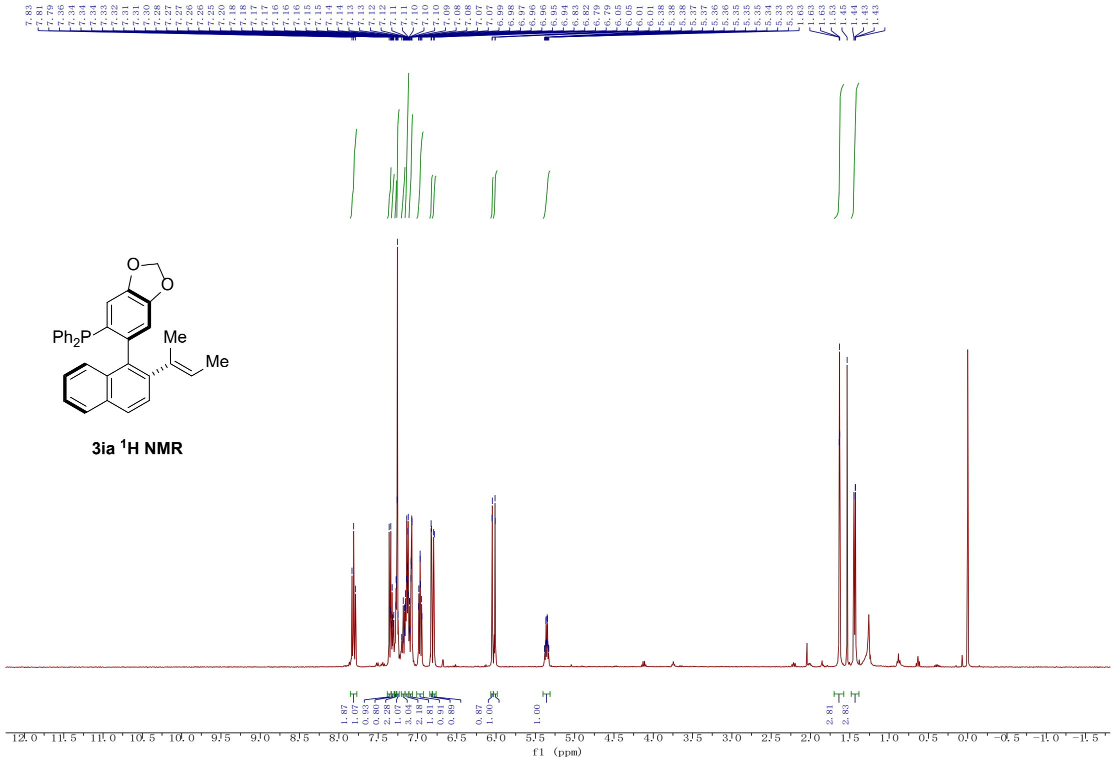

Supplementary Fig. 110. ^{1}H NMR spectra (400 MHz, $CDCl_3$, 25 ºC) of **3ia**

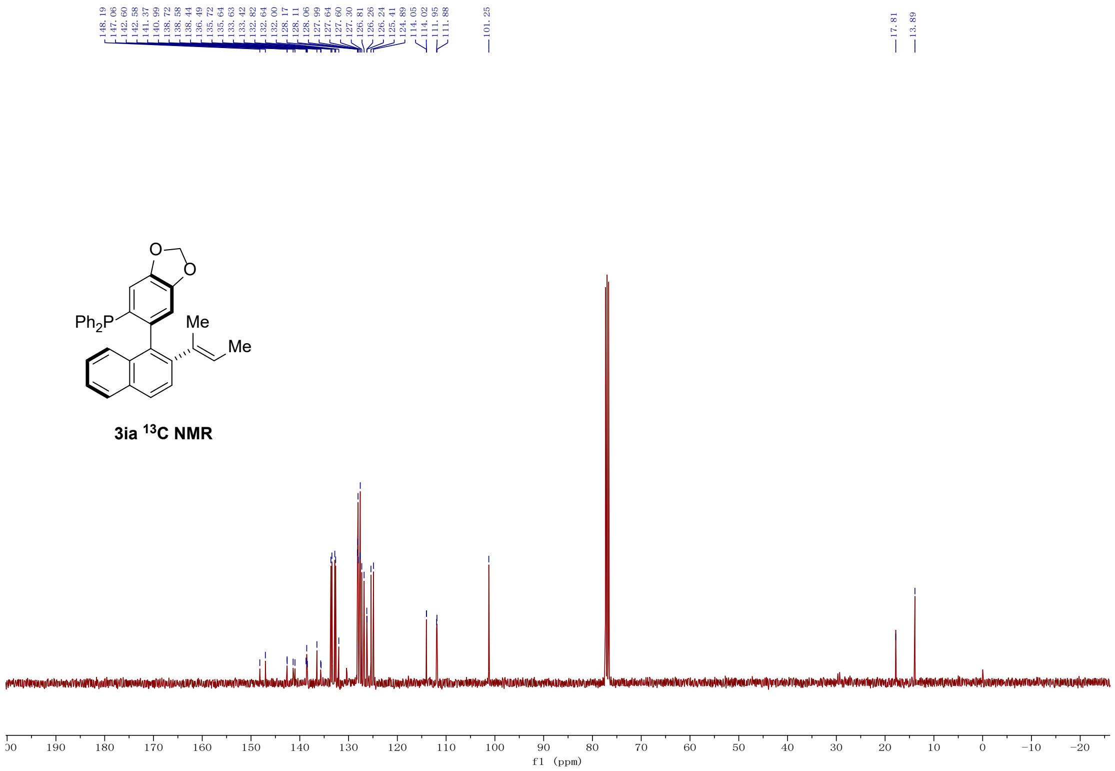

Supplementary Fig. 111. ^{13}C NMR spectra (101 MHz, $CDCl_3$, 25 °C) of **3ia**

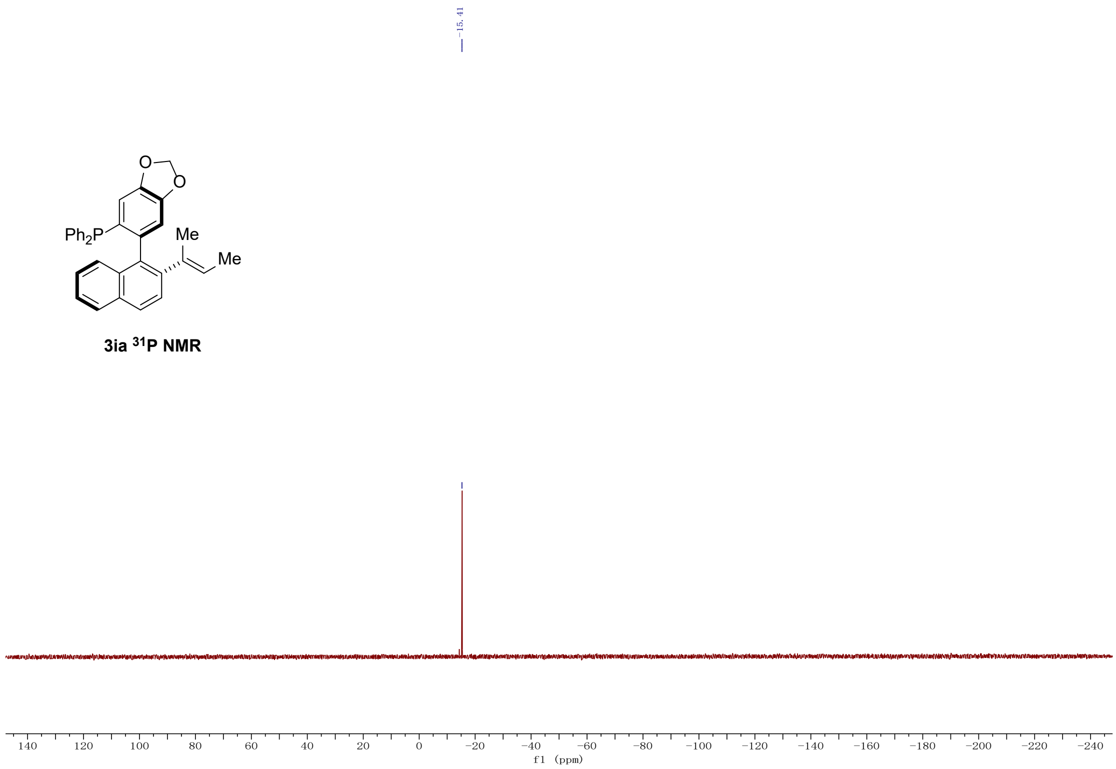

Supplementary Fig. 112. ^{31}P NMR spectra (162 MHz, $CDCl_3$, 25 °C) of **3ia**

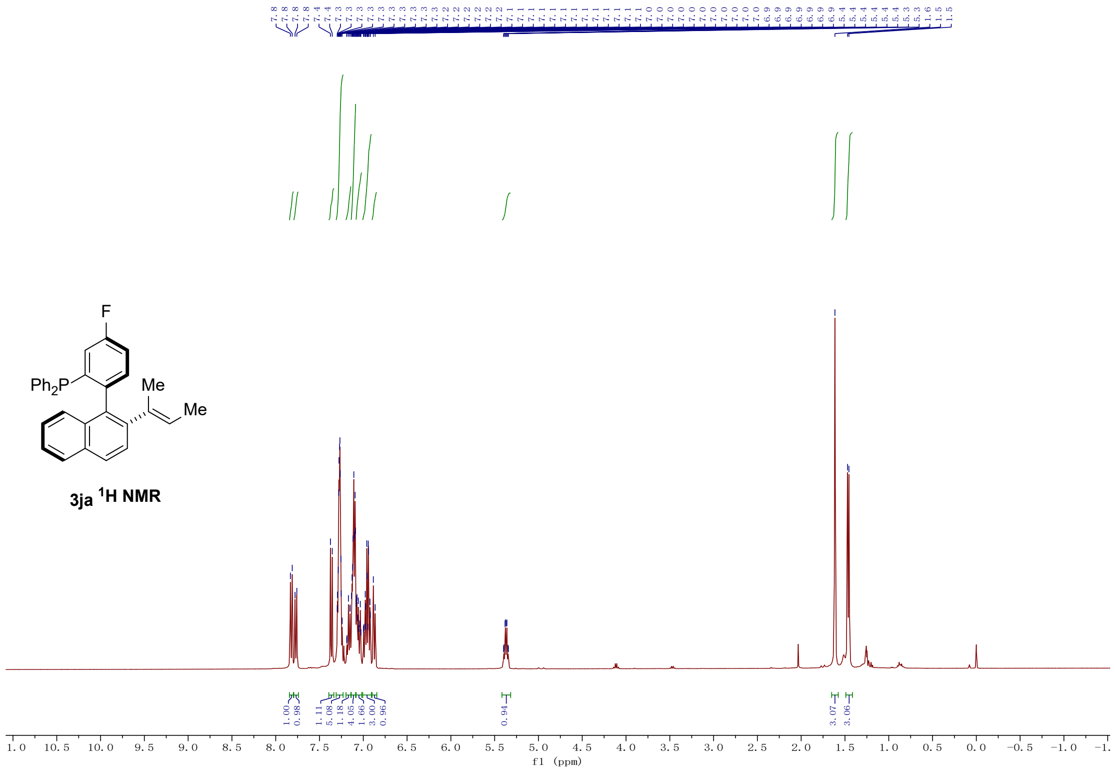

Supplementary Fig. 113. ^{1}H NMR spectra (400 MHz, $CDCl_3$, 25 ºC) of **3ja**

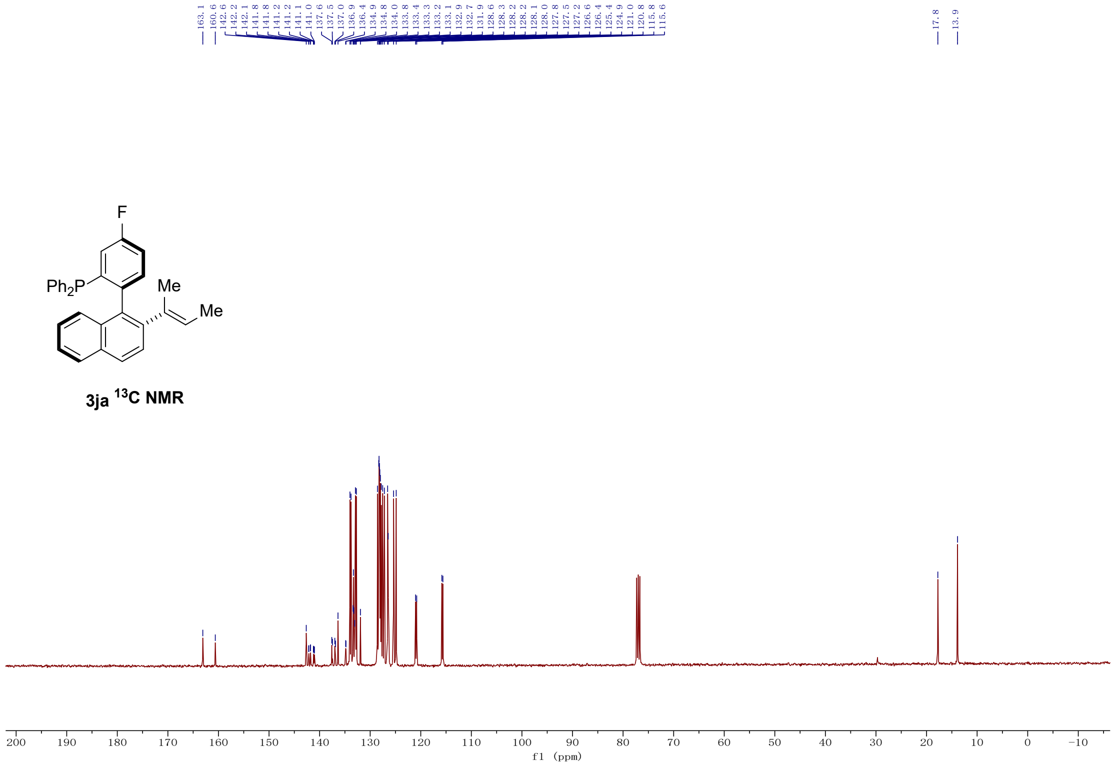

Supplementary Fig. 114. ^{13}C NMR spectra (101 MHz, $CDCl_3$, 25 ºC) of **3ja**

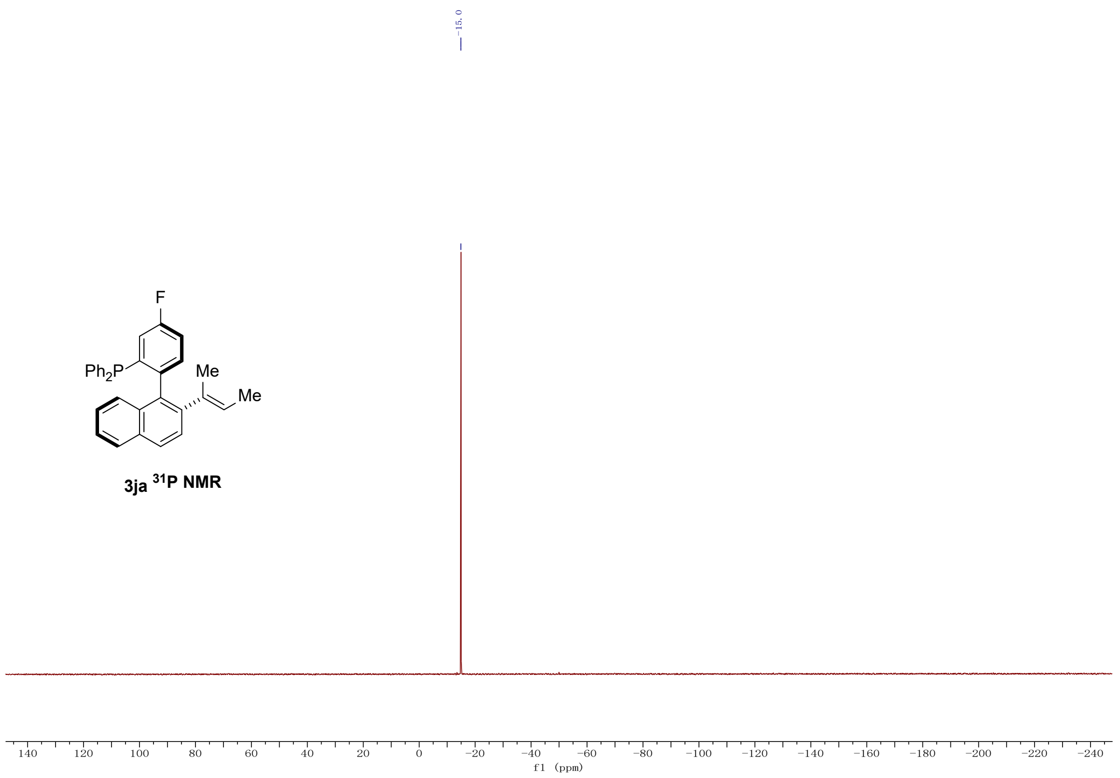

Supplementary Fig. 115. ^{31}P NMR spectra (162 MHz, $CDCl_3$, 25 °C) of **3ja**

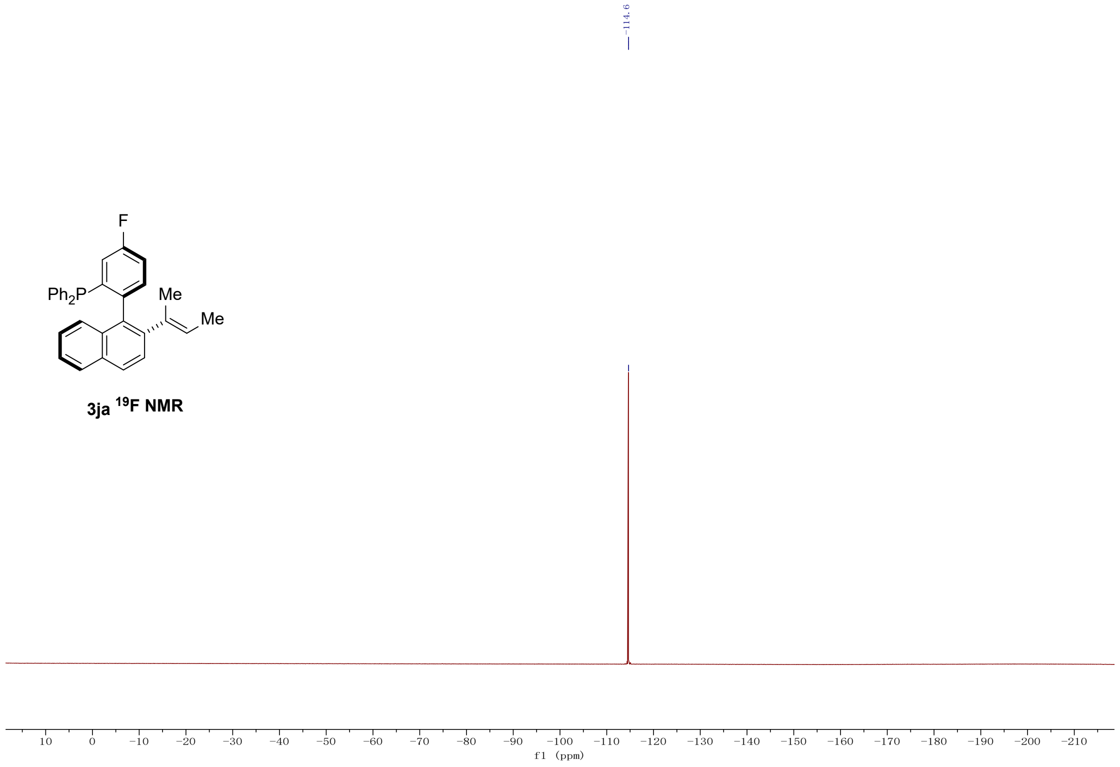

Supplementary Fig. 116. ^{19}F NMR spectra (376 MHz, $CDCl_3$, 25 °C) of **3ja**

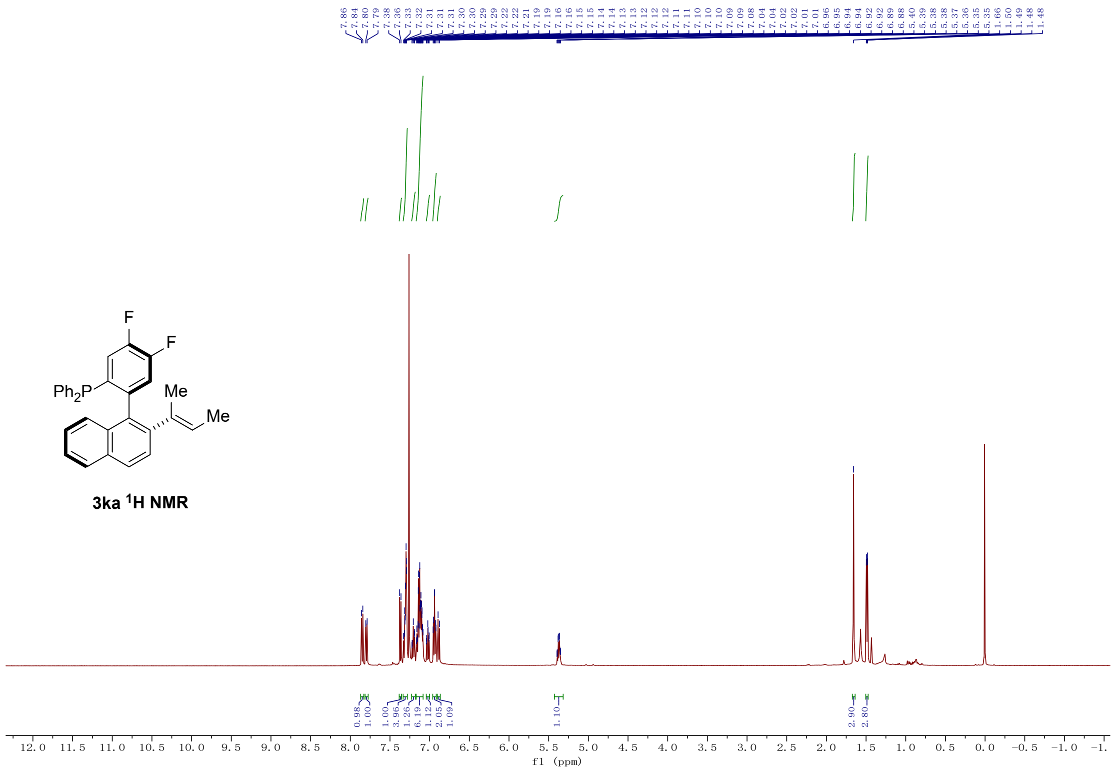

Supplementary Fig. 117. ^{1}H NMR spectra (500 MHz, CDCl3, 25 ºC) of **3ka**

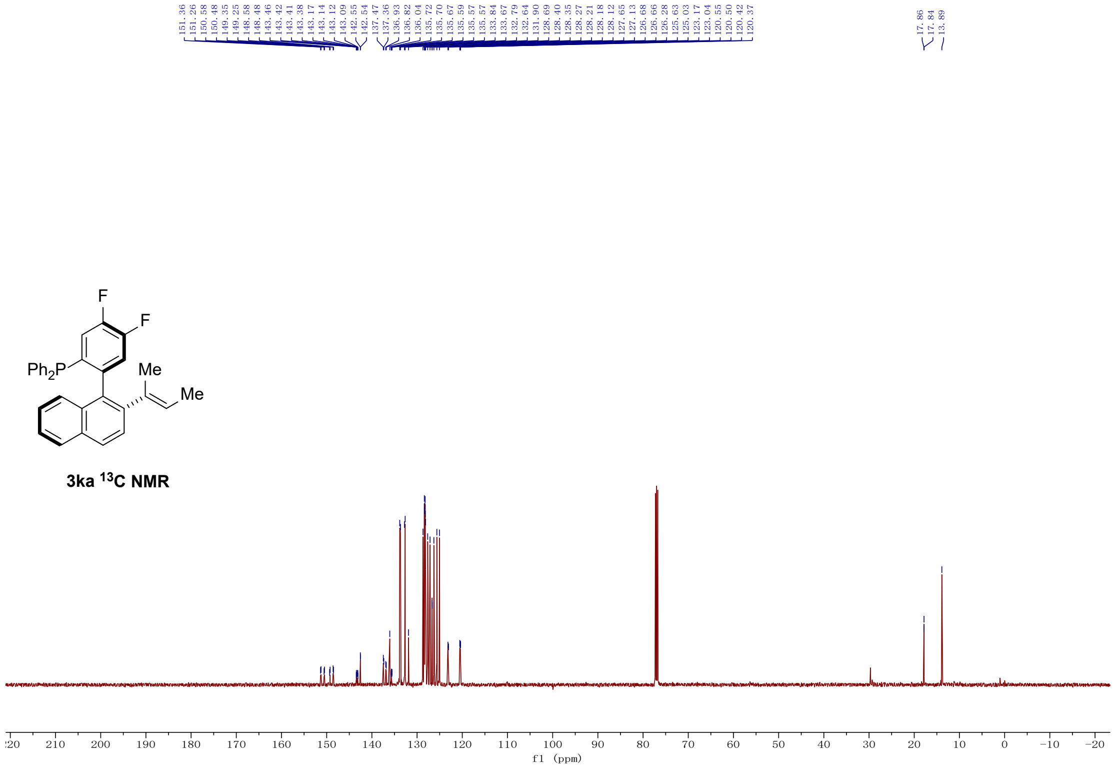

Supplementary Fig. 118. ^{13}C NMR spectra (126 MHz, CDCl3, 25 ºC) of **3ka**

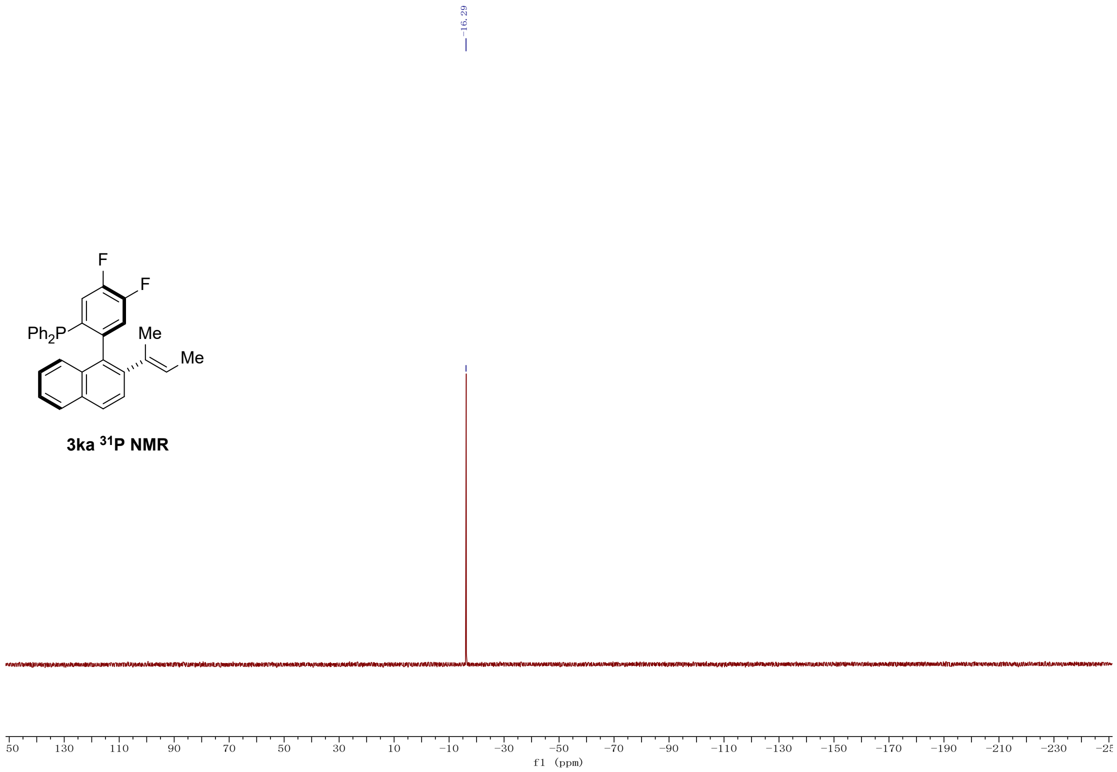

Supplementary Fig. 119. ^{31}P NMR spectra (202 MHz, $CDCl_3$, 25 °C) of **3ka**

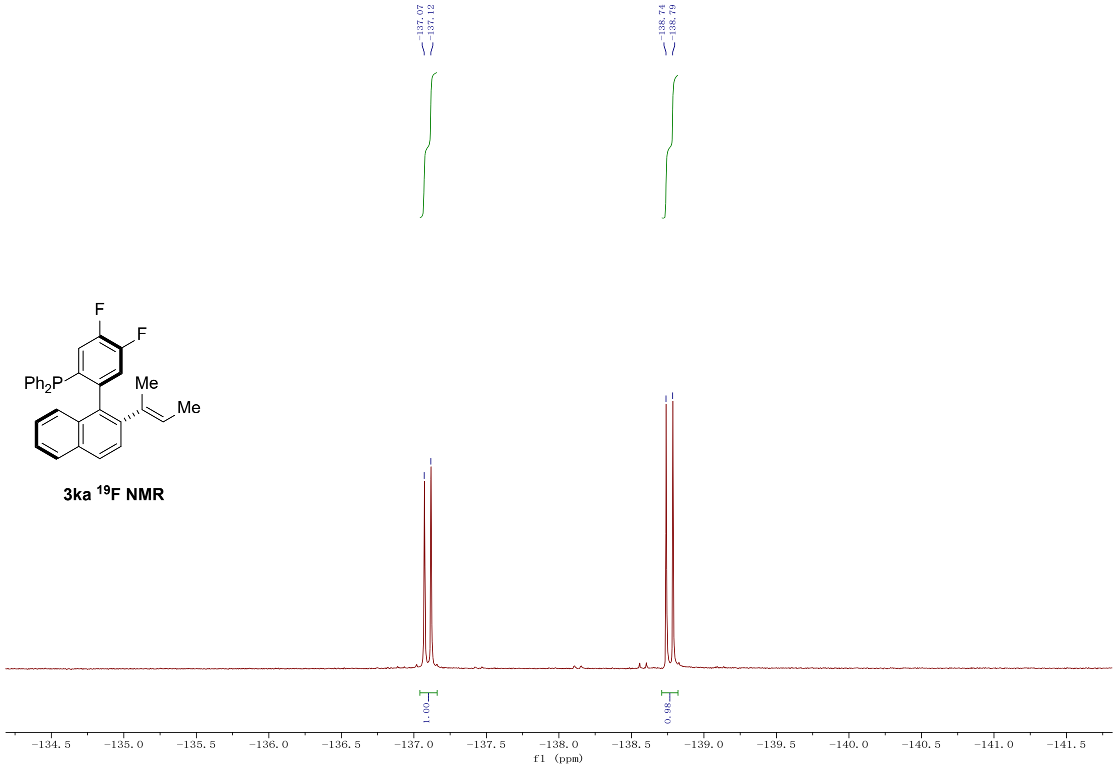

Supplementary Fig. 120. ^{19}F NMR spectra (471 MHz, $CDCl_3$, 25 °C) of **3ka**

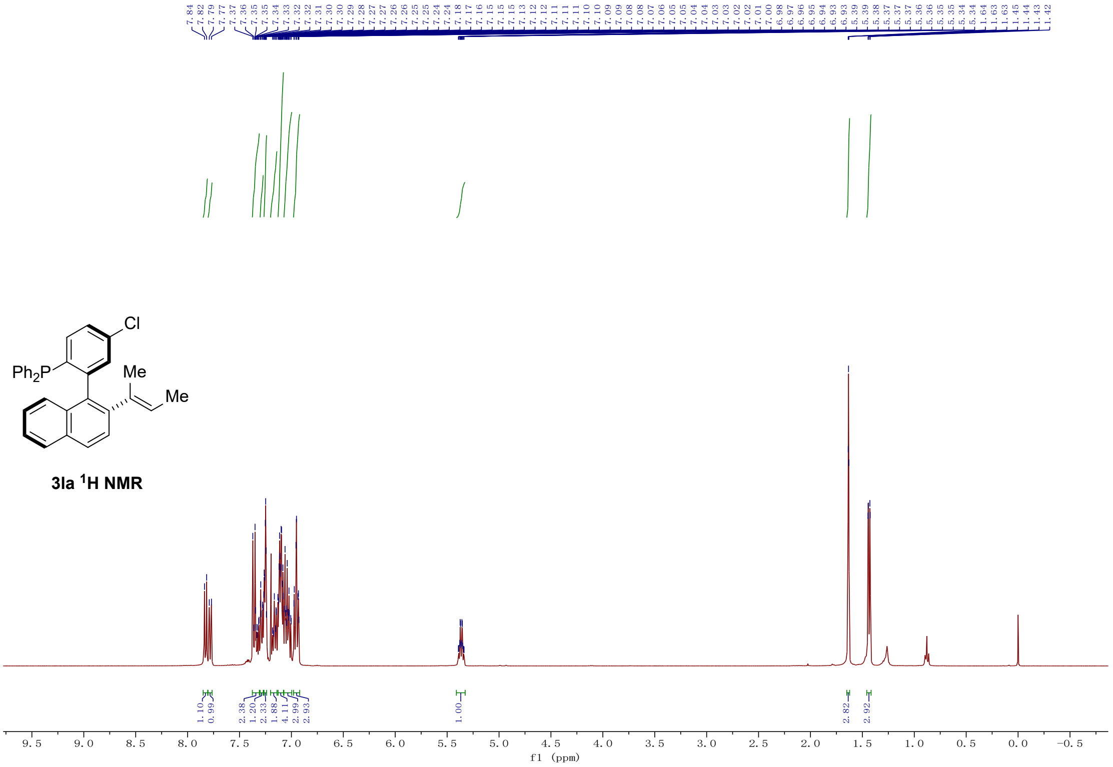

Supplementary Fig. 121. ^{1}H NMR spectra (400 MHz, $CDCl_3$, 25 °C) of **3la**

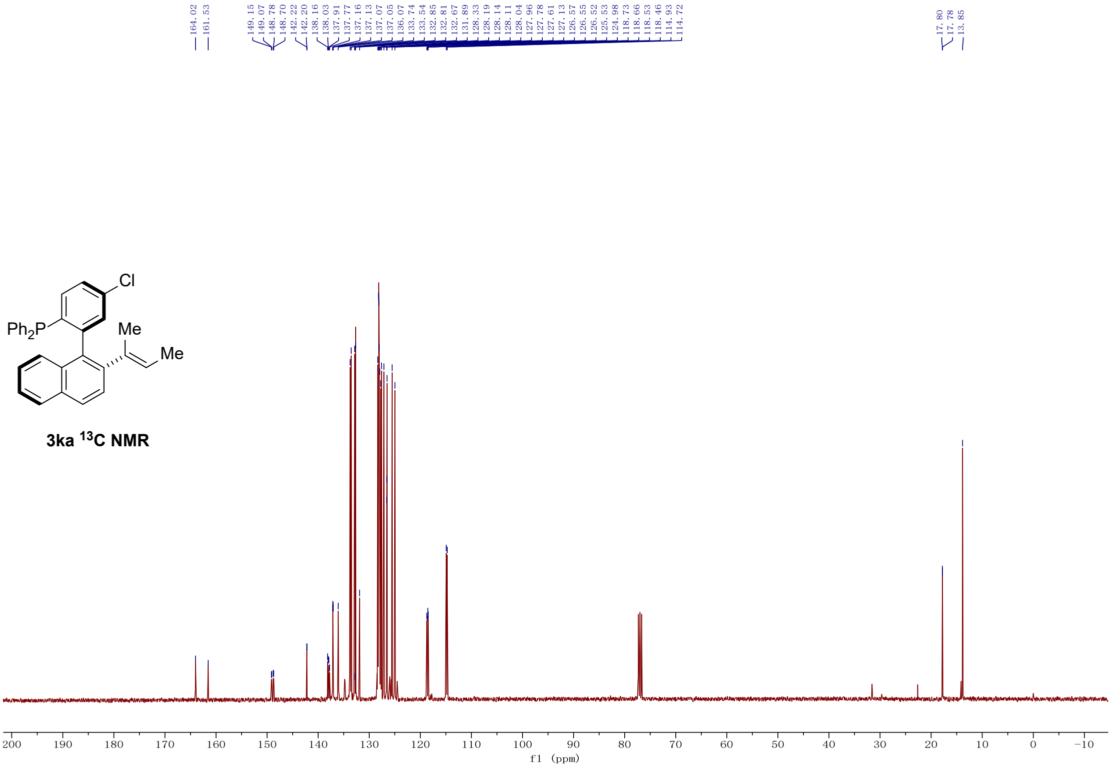

Supplementary Fig. 122. ^{13}C NMR spectra (101 MHz, $CDCl_3$, 25 °C) of **3la**

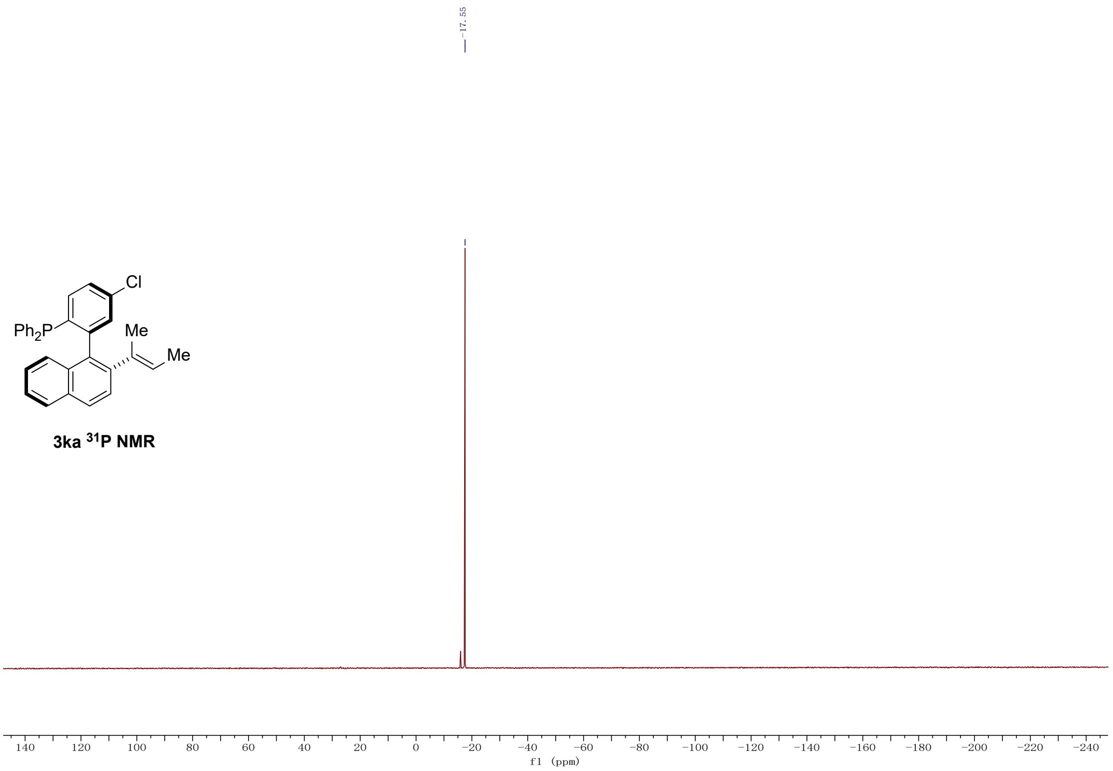

Supplementary Fig. 123. ^{31}P NMR spectra (162 MHz, $CDCl_3$, 25 °C) of **3la**

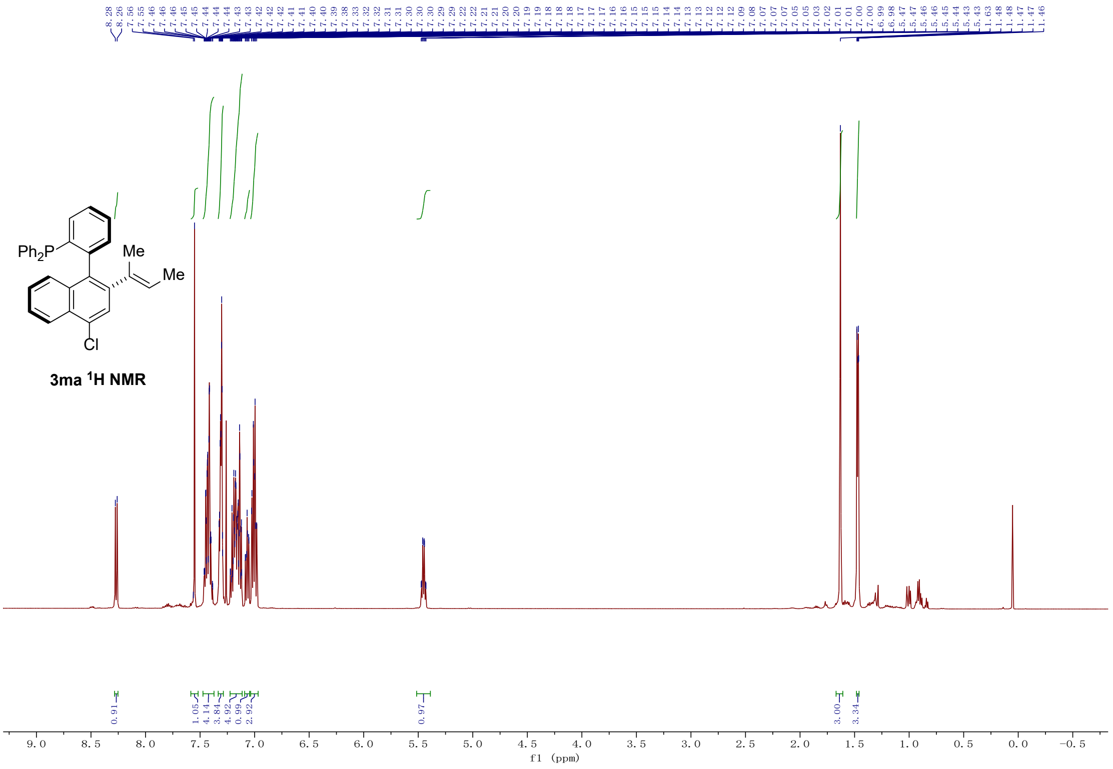

Supplementary Fig. 124. ^{1}H NMR spectra (500 MHz, $CDCl_3$, 25 °C) of **3ma**

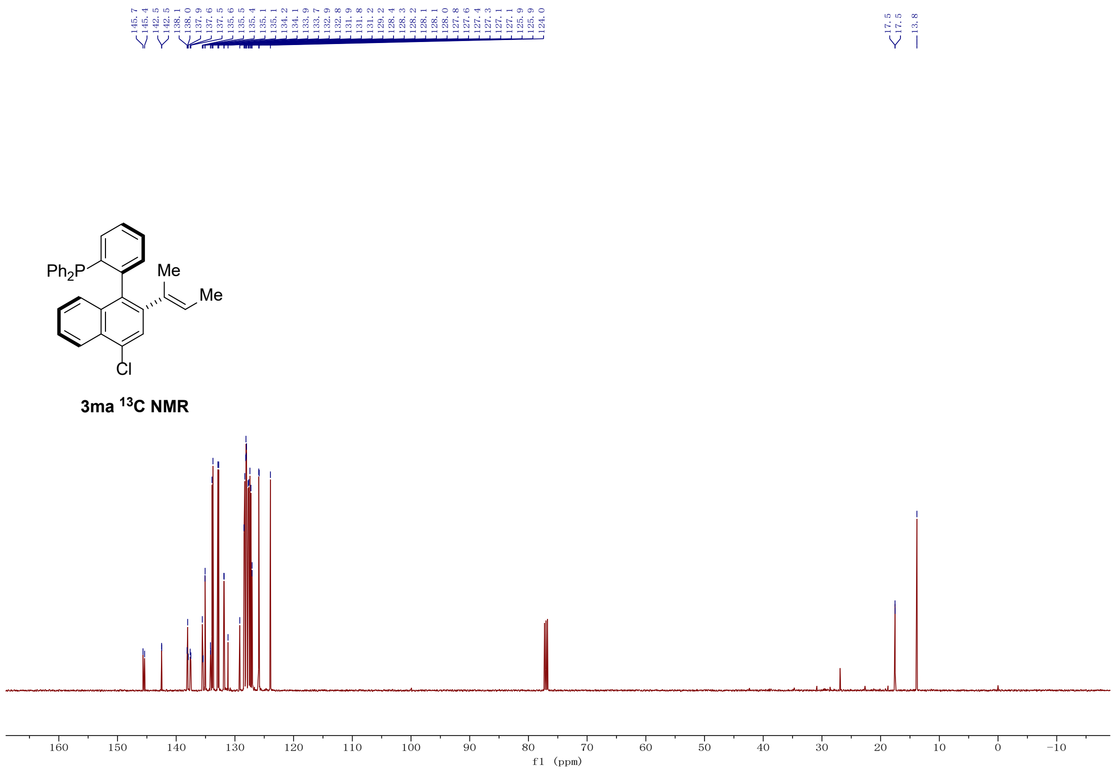

Supplementary Fig. 125. ^{13}C NMR spectra (126 MHz, $CDCl_3$, 25 ºC) of **3ma**

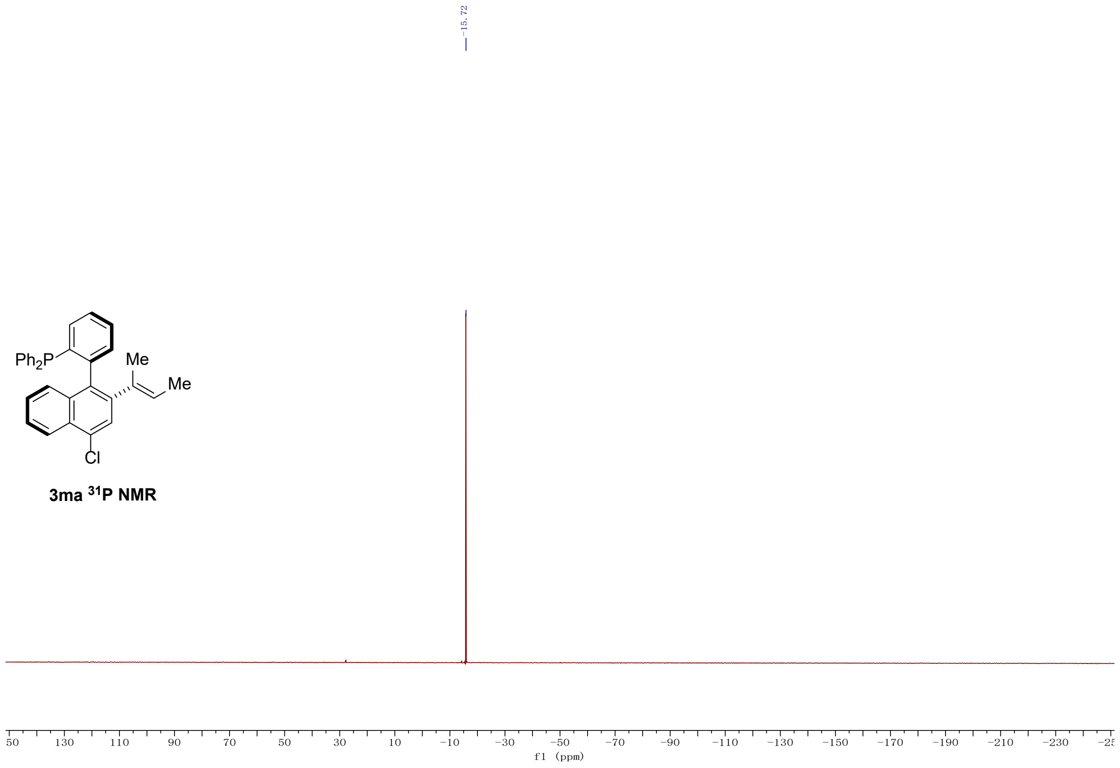

Supplementary Fig. 126. ^{31}P NMR spectra (202 MHz, $CDCl_3$, 25 ºC) of **3ma**

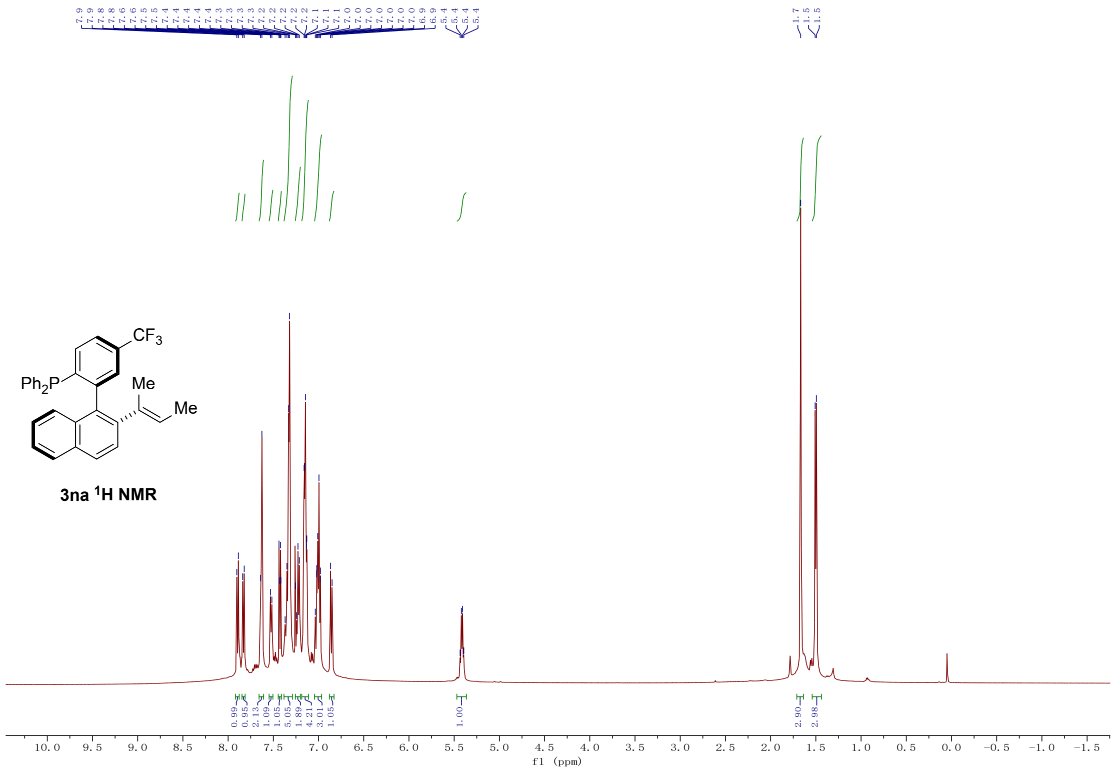

Supplementary Fig. 127. ^{1}H NMR spectra (500 MHz, $CDCl_3$, 25 °C) of **3na**

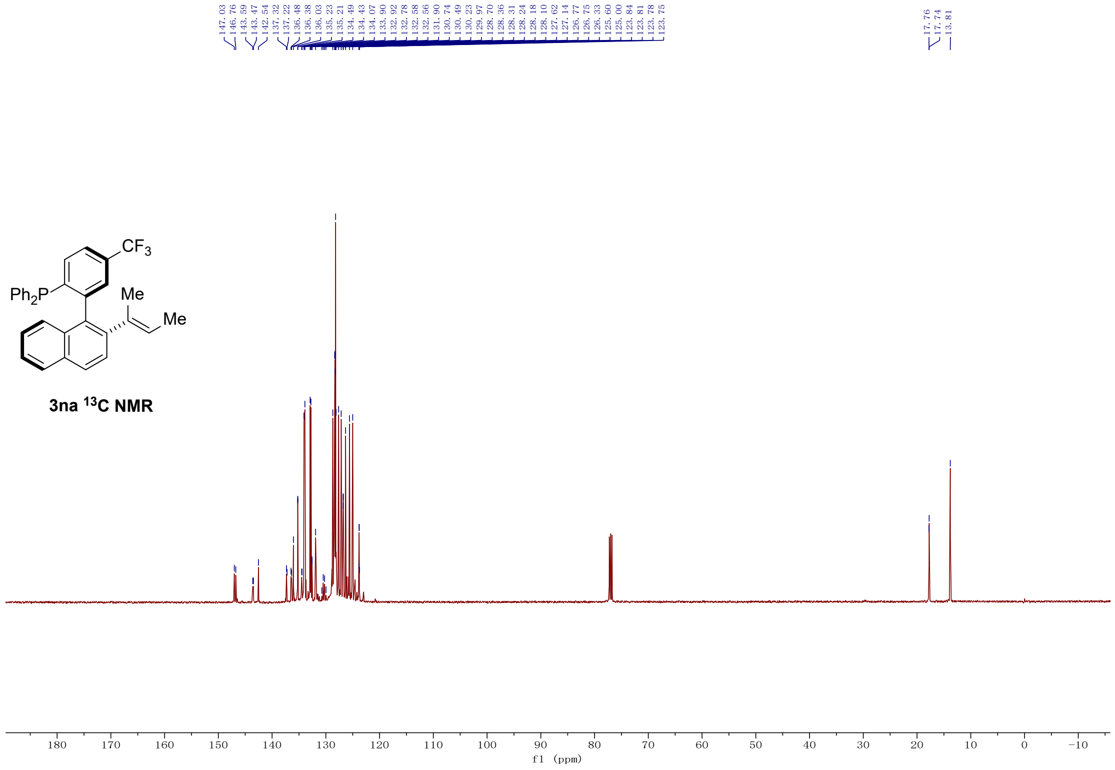

Supplementary Fig. 128. ^{13}C NMR spectra (126 MHz, $CDCl_3$, 25 °C) of **3na**

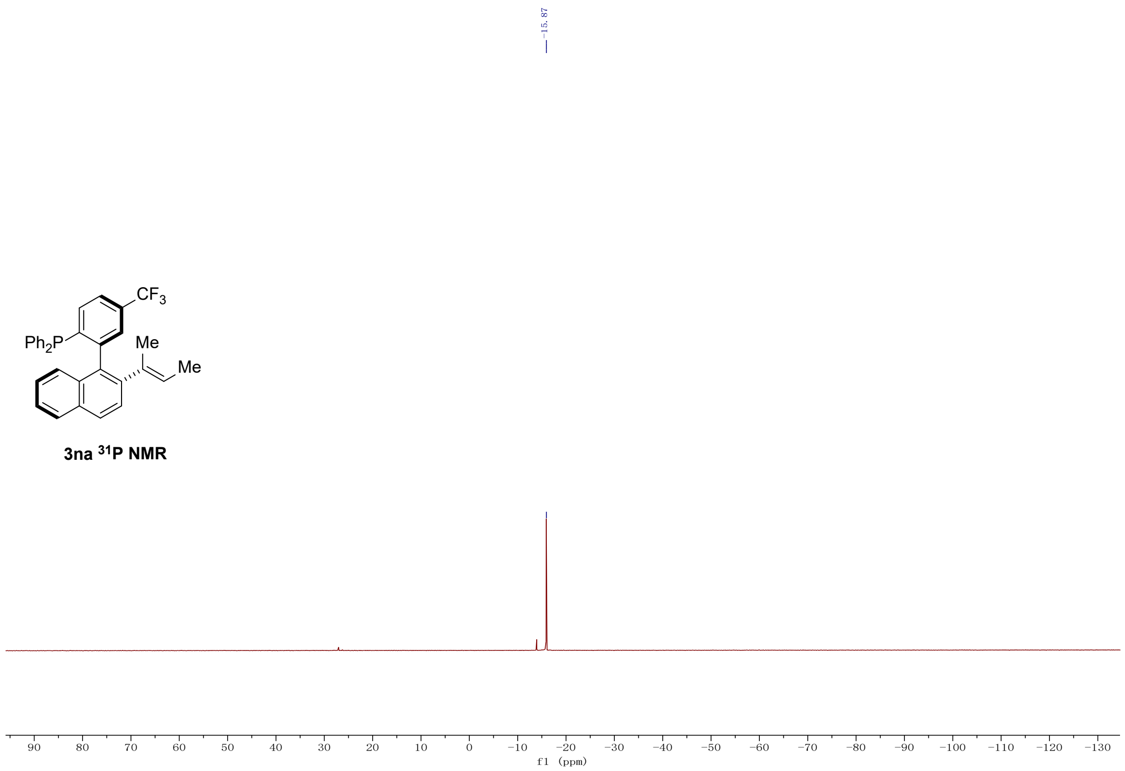

Supplementary Fig. 129. ^{31}P NMR spectra (202 MHz, $CDCl_3$, 25 °C) of **3na**

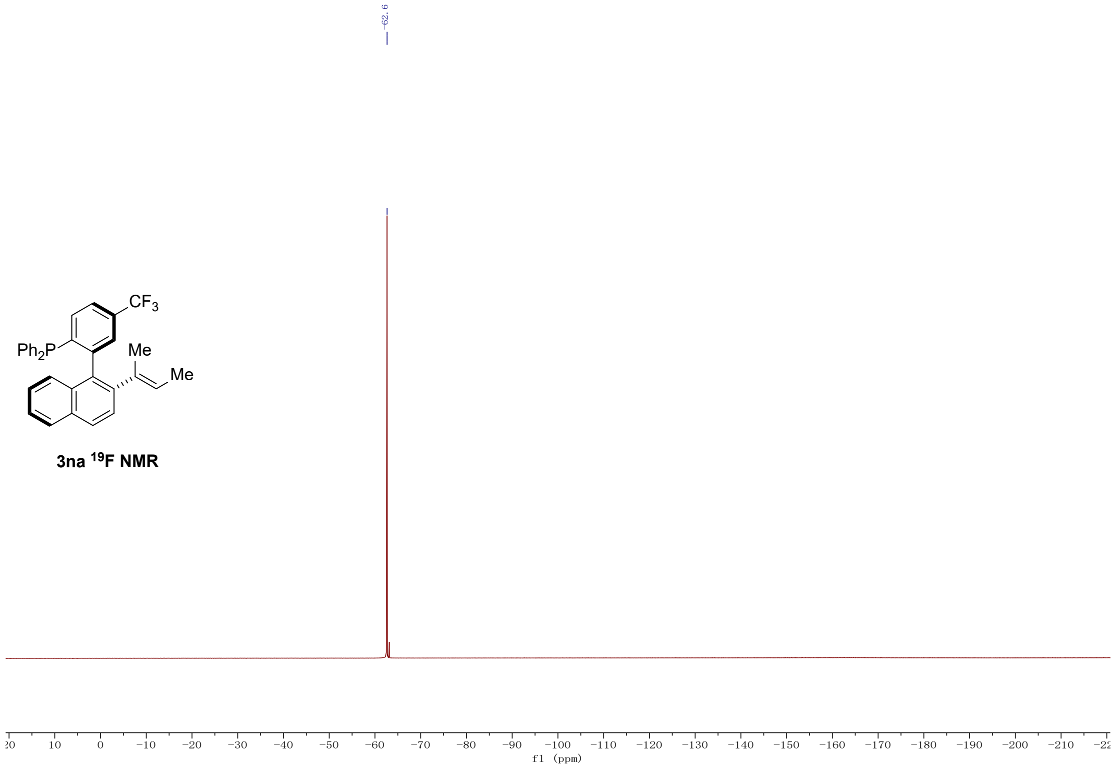

Supplementary Fig. 130. ^{19}F NMR spectra (471 MHz, $CDCl_3$, 25 °C) of **3na**

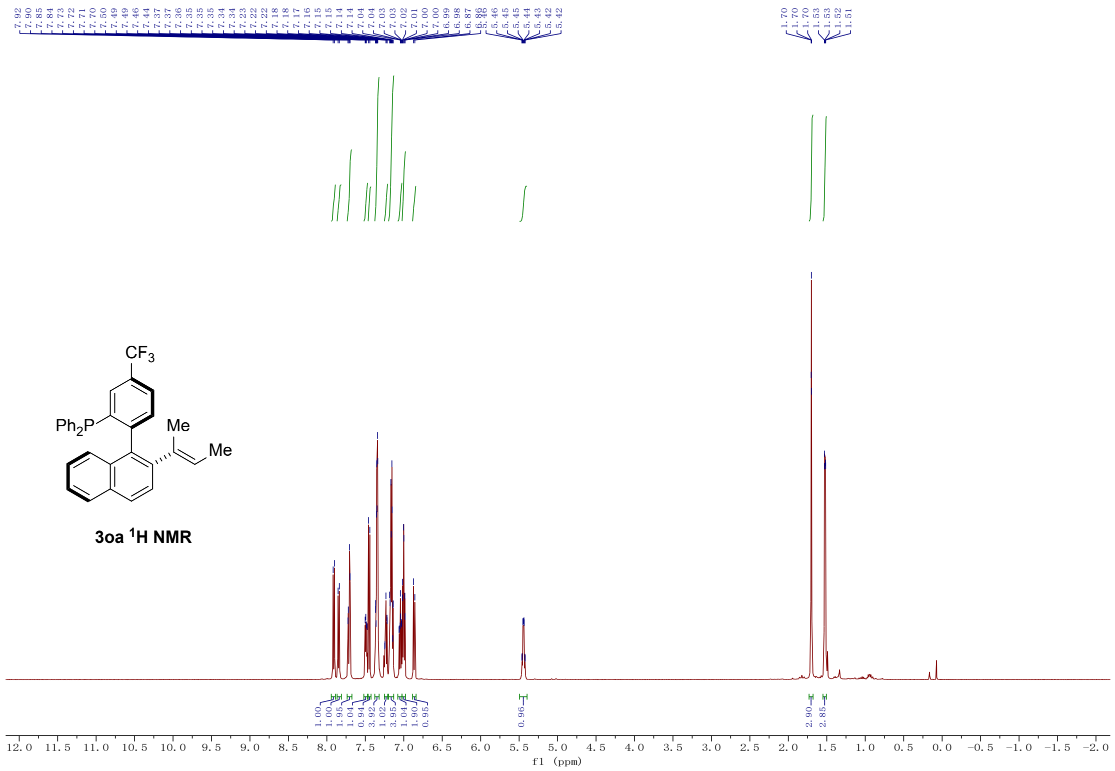

Supplementary Fig. 131. ^{1}H NMR spectra (500 MHz, $CDCl_3$, 25 °C) of **3oa**

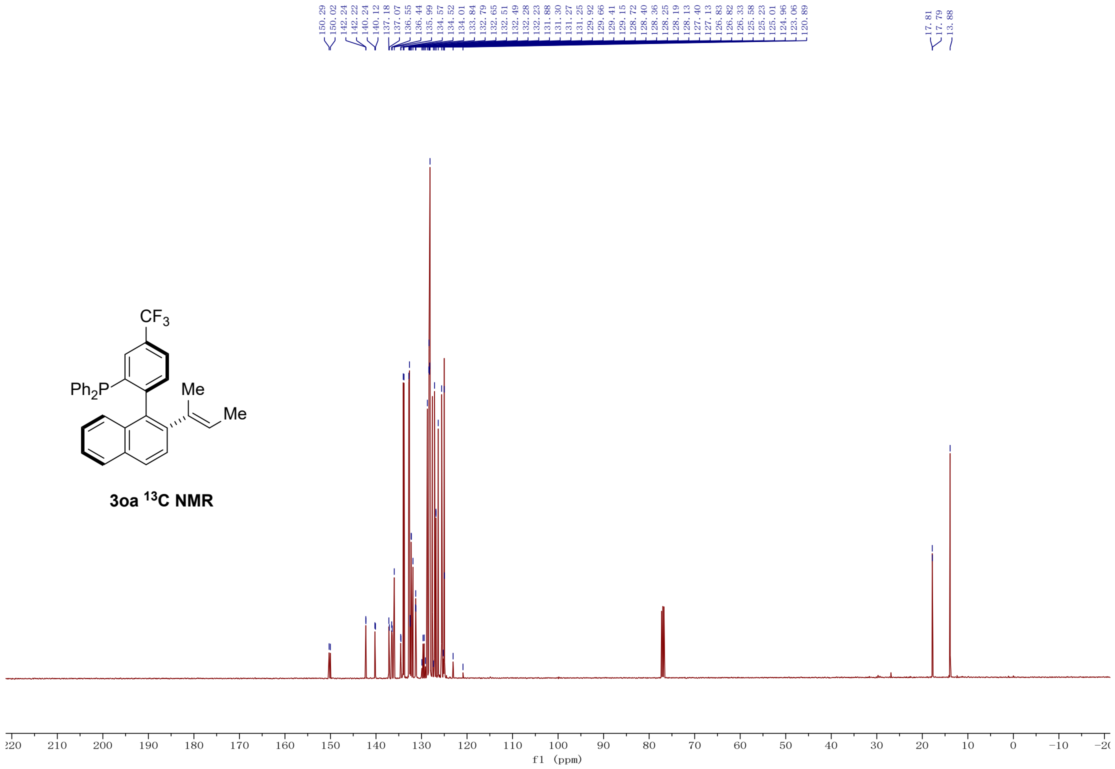

Supplementary Fig. 132. ^{13}C NMR spectra (126 MHz, $CDCl_3$, 25 °C) of **3oa**

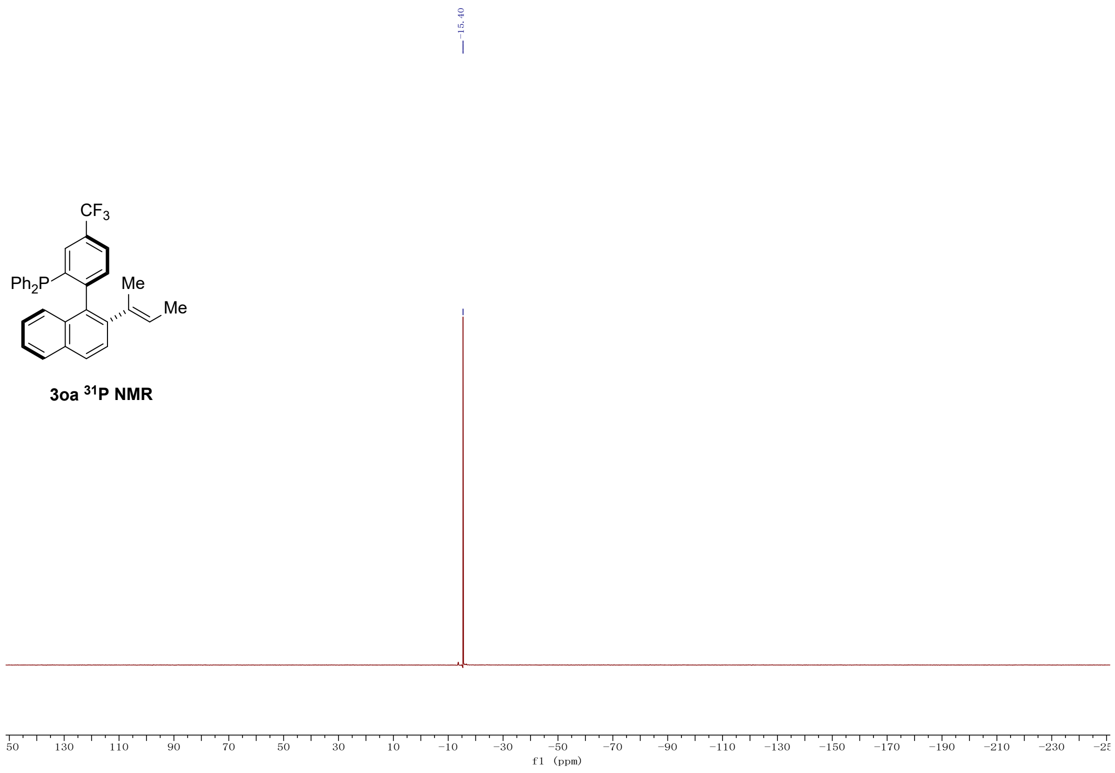

Supplementary Fig. 133. ^{31}P NMR spectra (202 MHz, $CDCl_3$, 25 °C) of **3oa**

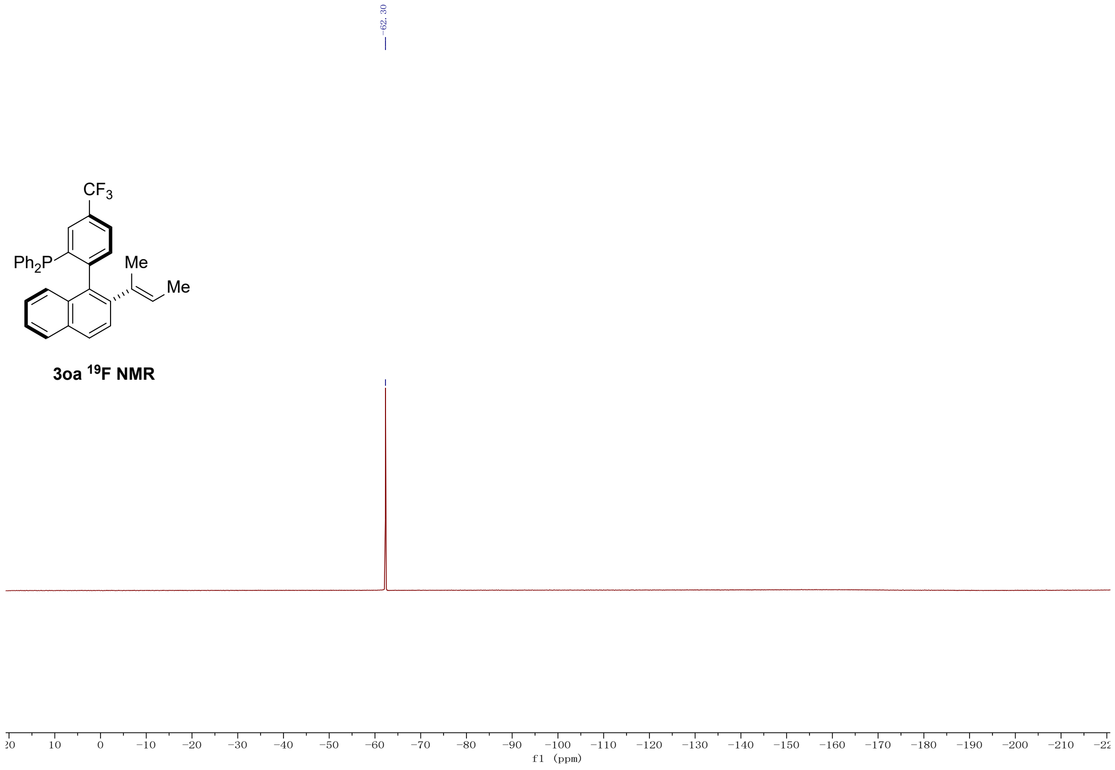

Supplementary Fig. 134. ^{19}F NMR spectra (471 MHz, $CDCl_3$, 25 °C) of **3oa**

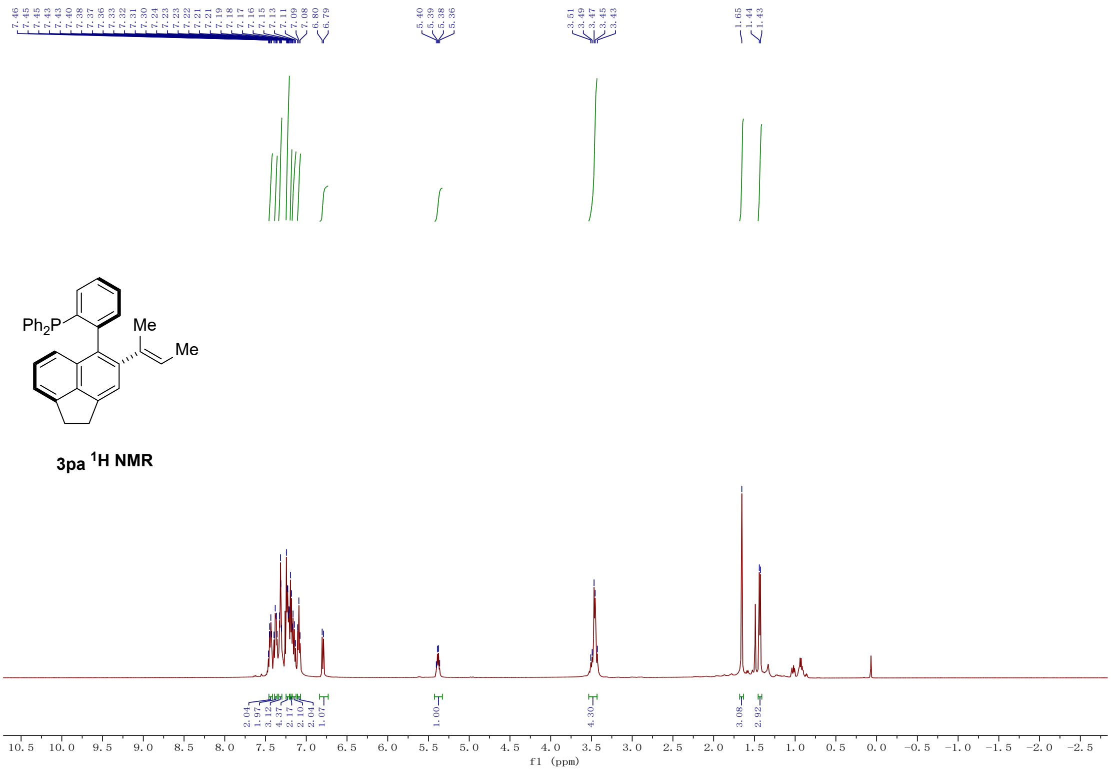

Supplementary Fig. 135. ^{1}H NMR spectra (500 MHz, $CDCl_3$, 25 °C) of **3pa**

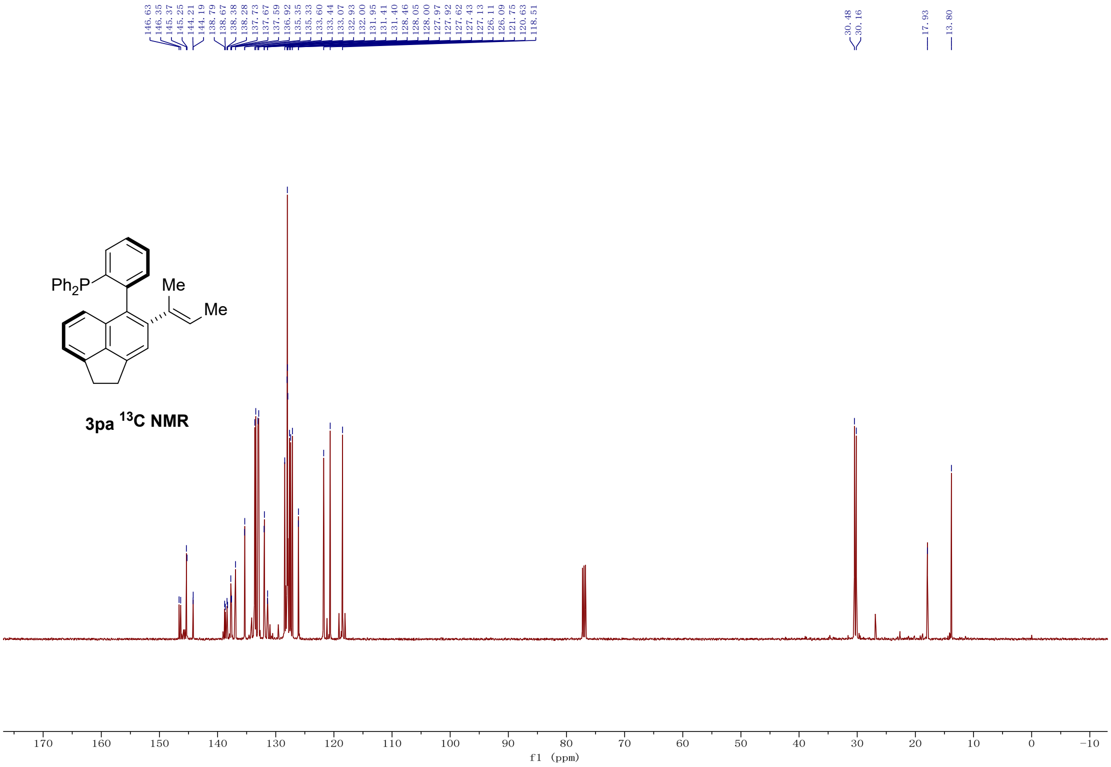

Supplementary Fig. 136. ^{13}C NMR spectra (126 MHz, $CDCl_3$, 25 °C) of **3pa**

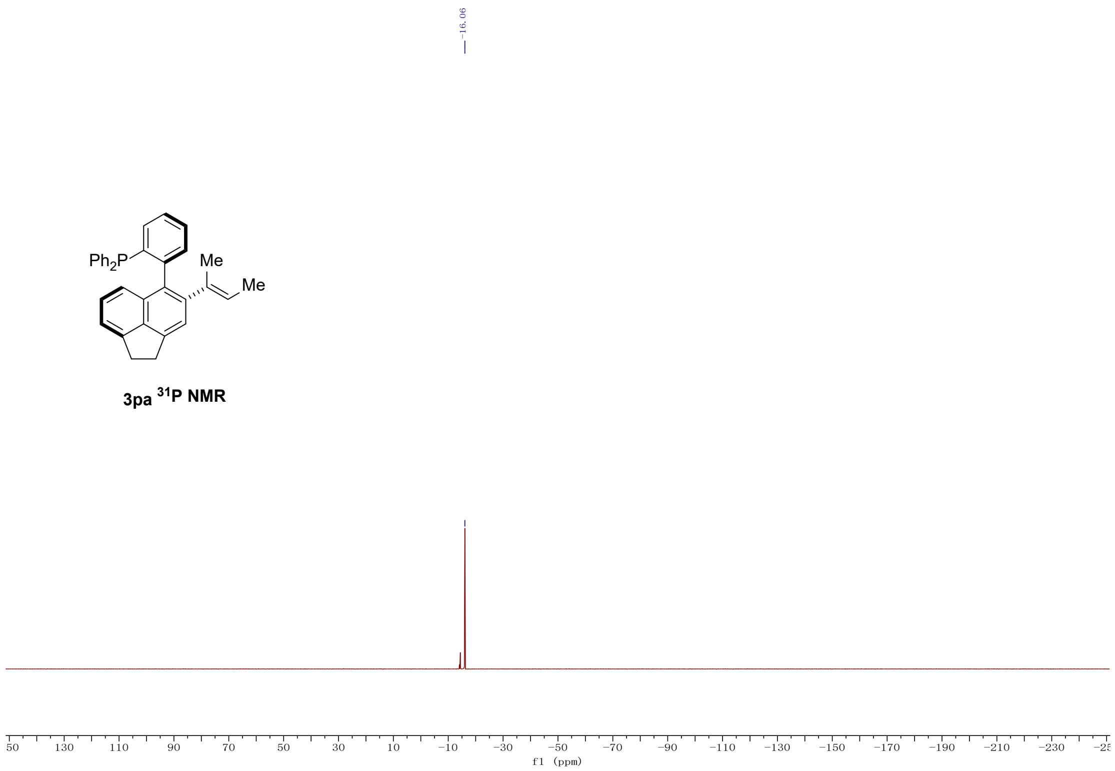

Supplementary Fig. 137. ^{31}P NMR spectra (202 MHz, $CDCl_3$, 25 °C) of **3pa**

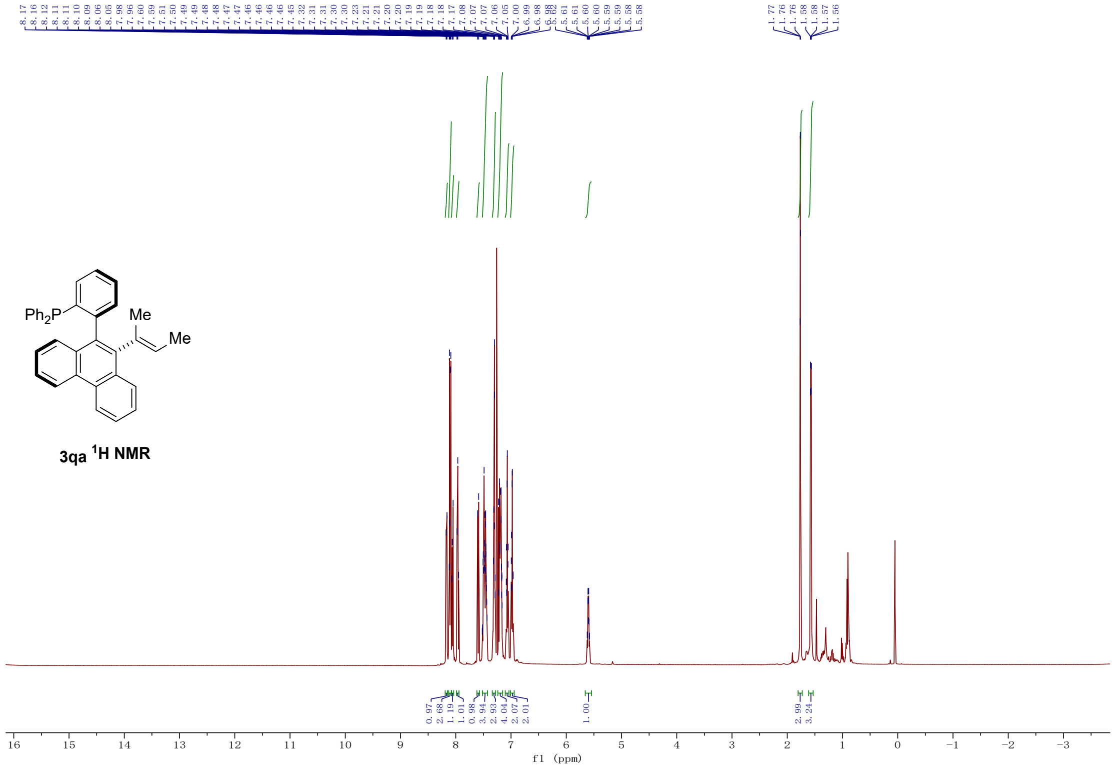

Supplementary Fig. 138. ^{1}H NMR spectra (500 MHz, $CDCl_3$, 25 °C) of **3qa**

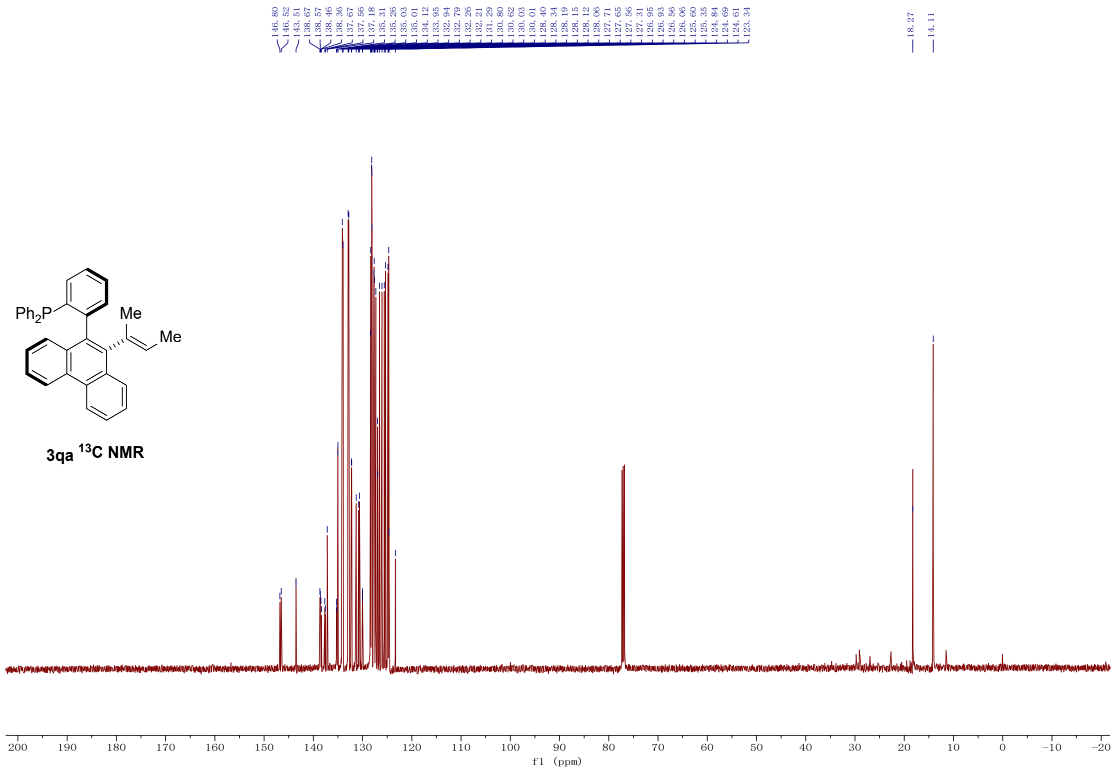

Supplementary Fig. 139. ^{13}C NMR spectra (126 MHz, $CDCl_3$, 25 °C) of **3qa**

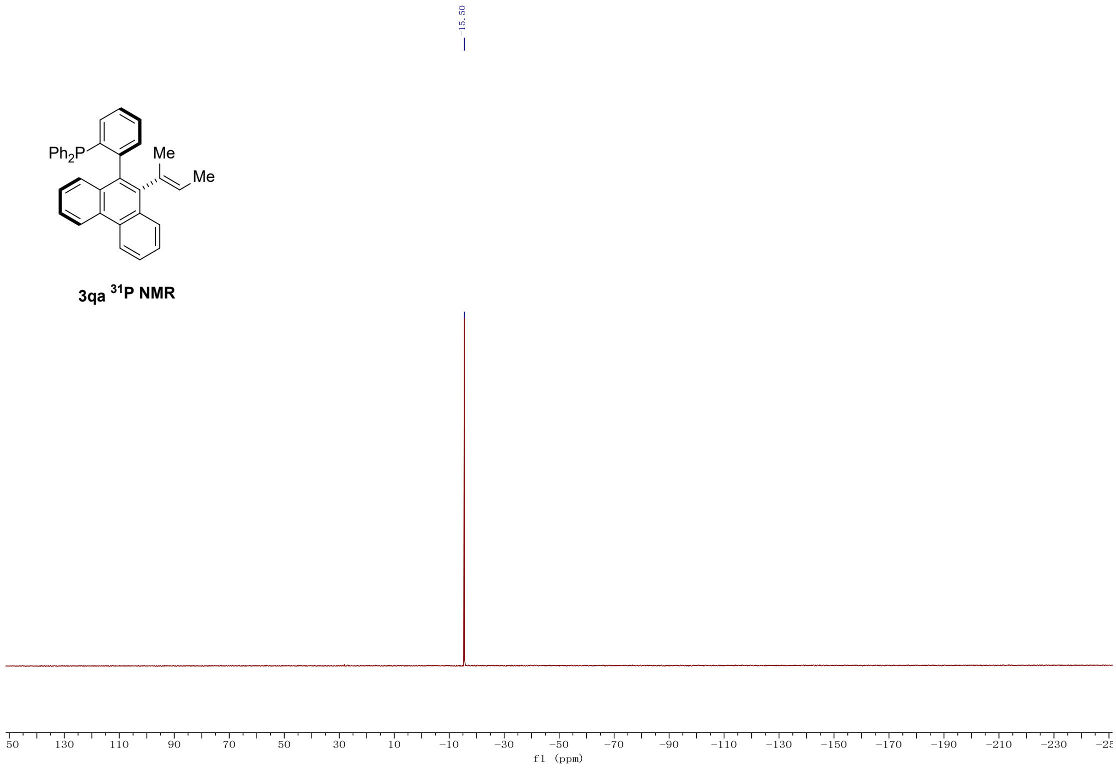

Supplementary Fig. 140. ^{31}P NMR spectra (202 MHz, $CDCl_3$, 25 °C) of **3qa**

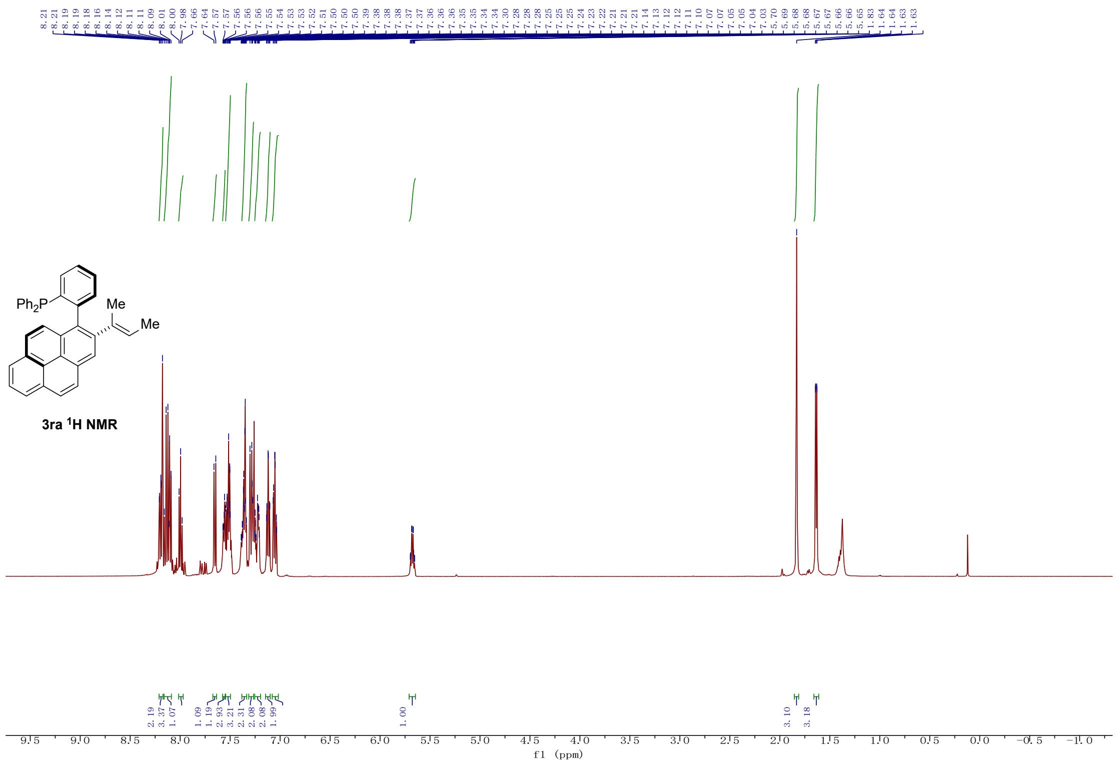

Supplementary Fig. 141. ^{1}H NMR spectra (500 MHz, $CDCl_3$, 25 °C) of **3ra**

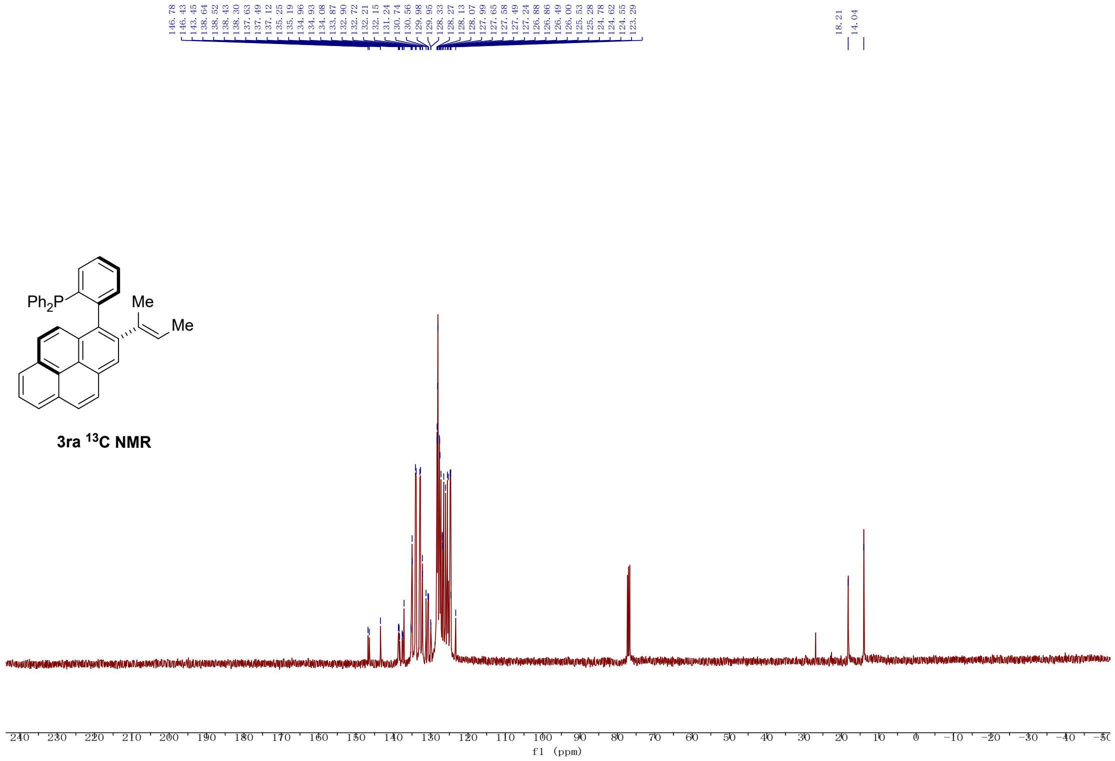

Supplementary Fig. 142. ^{13}C NMR spectra (126 MHz, $CDCl_3$, 25 °C) of **3ra**

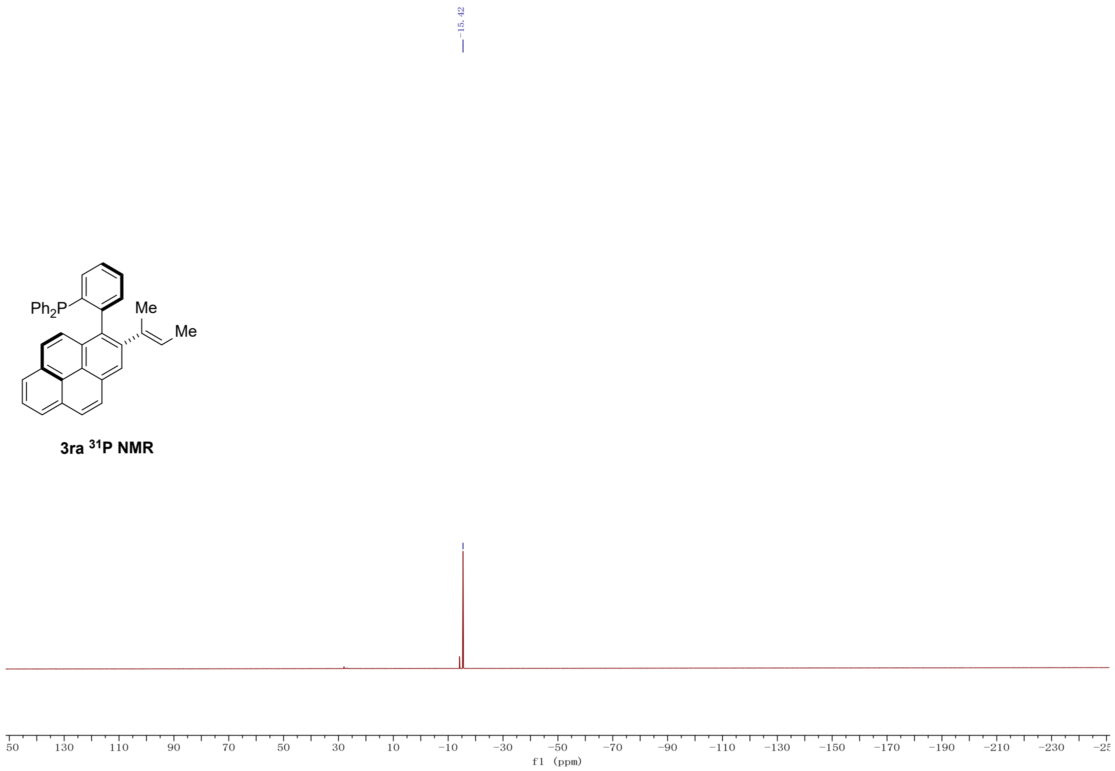

Supplementary Fig. 143. ^{31}P NMR spectra (202 MHz, $CDCl_3$, 25 °C) of **3ra**

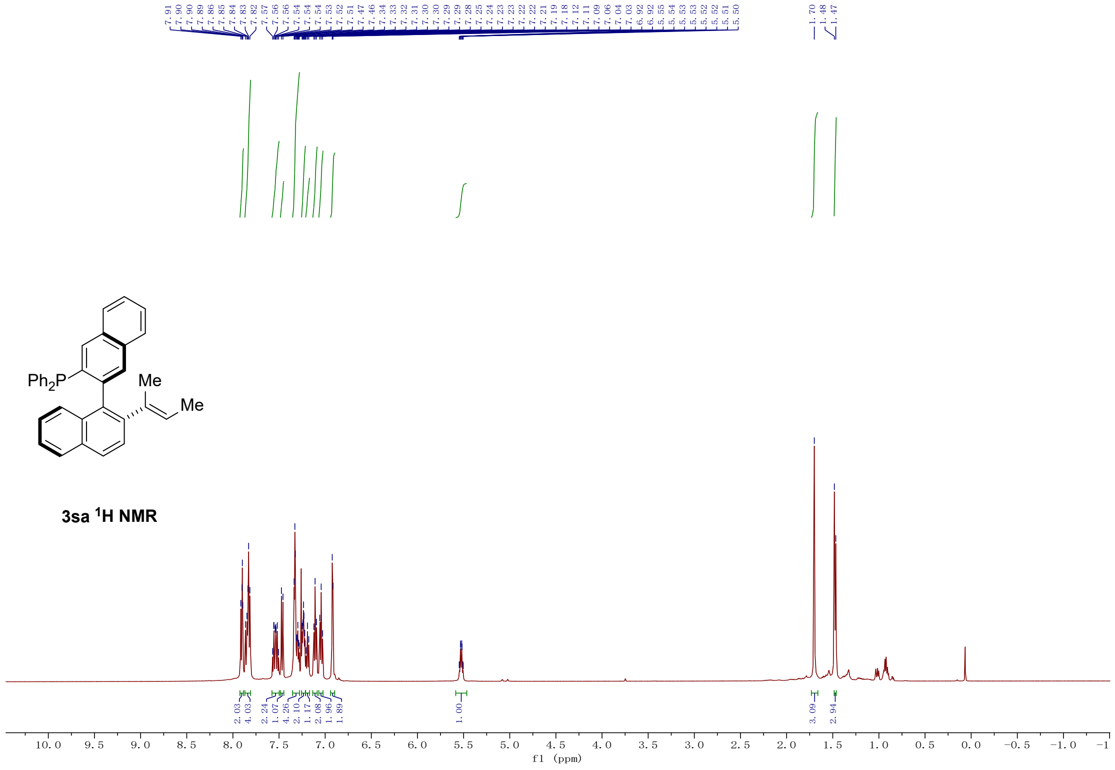

Supplementary Fig. 144. ^{1}H NMR spectra (500 MHz, $CDCl_3$, 25 °C) of **3sa**

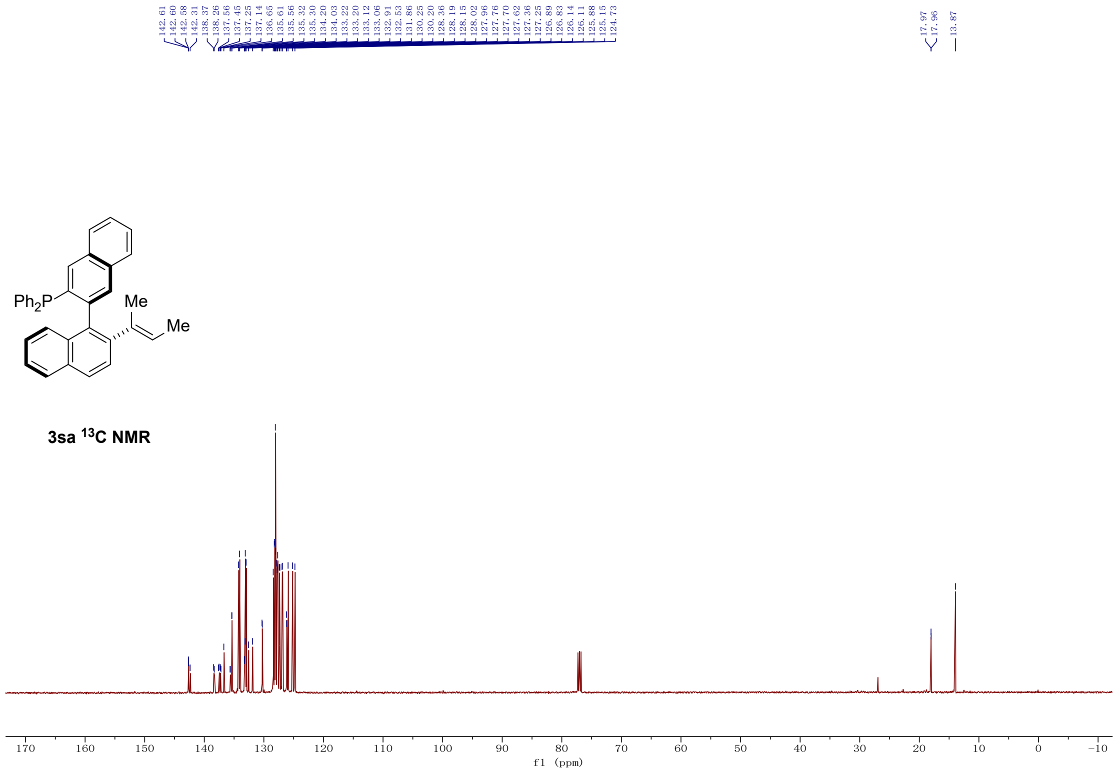

Supplementary Fig. 145. ^{13}C NMR spectra (126 MHz, $CDCl_3$, 25 °C) of **3sa**

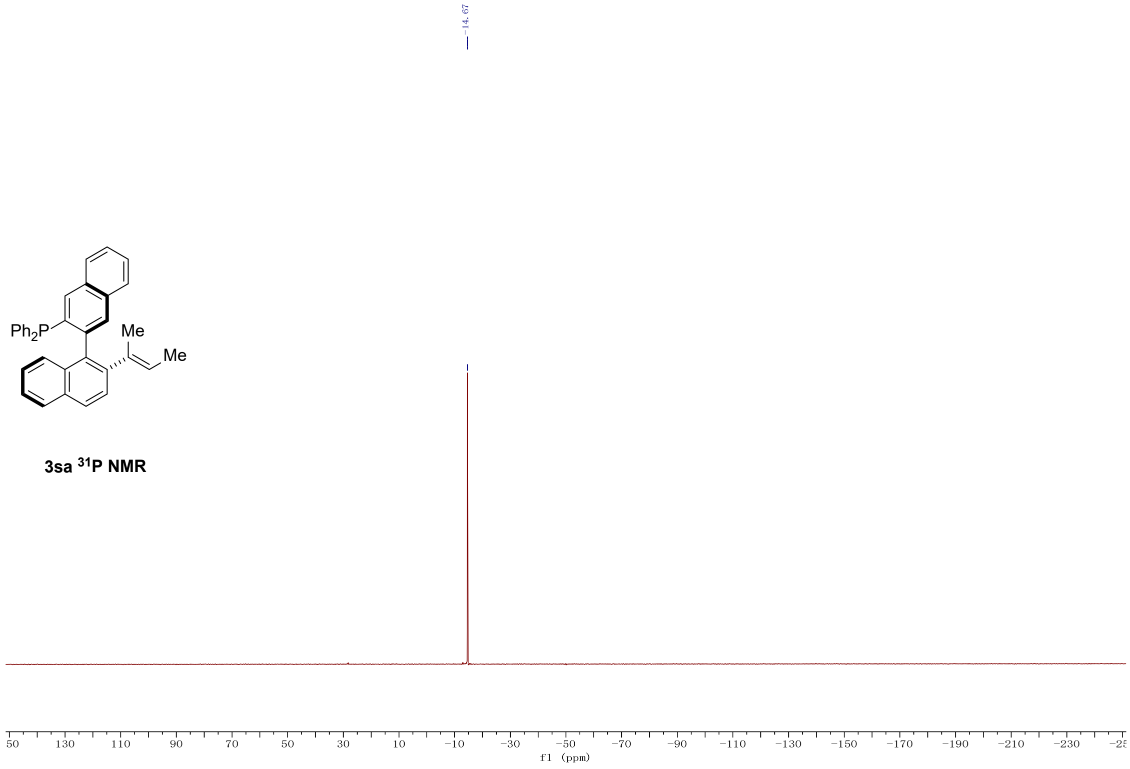

Supplementary Fig. 146. ^{31}P NMR spectra (202 MHz, $CDCl_3$, 25 °C) of **3sa**

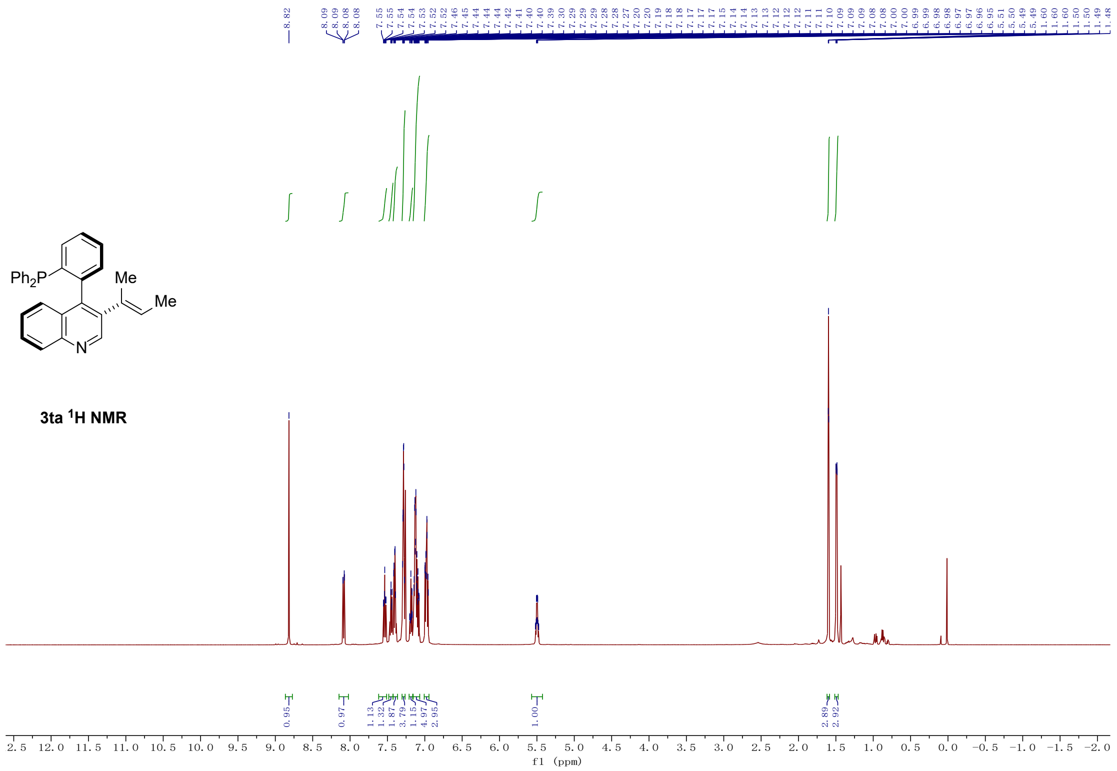

Supplementary Fig. 147. ^{1}H NMR spectra (500 MHz, CDCl$_3$, 25 °C) of **3ta**

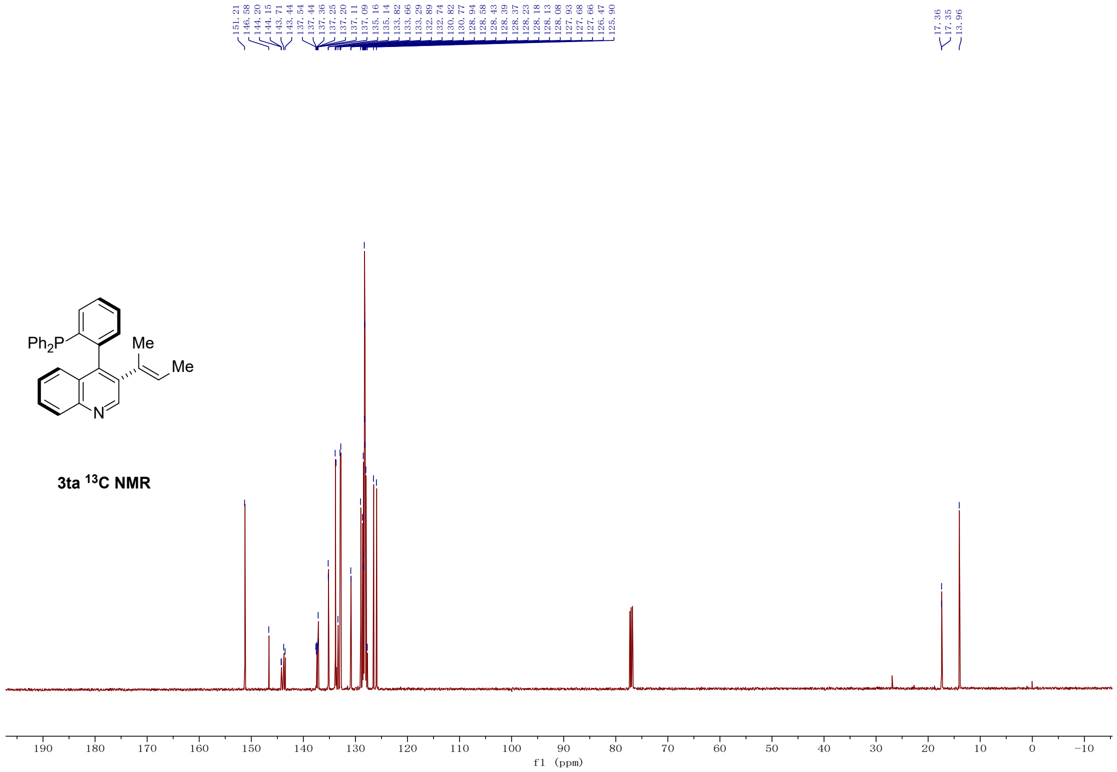

Supplementary Fig. 148. ^{13}C NMR spectra (126 MHz, CDCl$_3$, 25 °C) of **3ta**

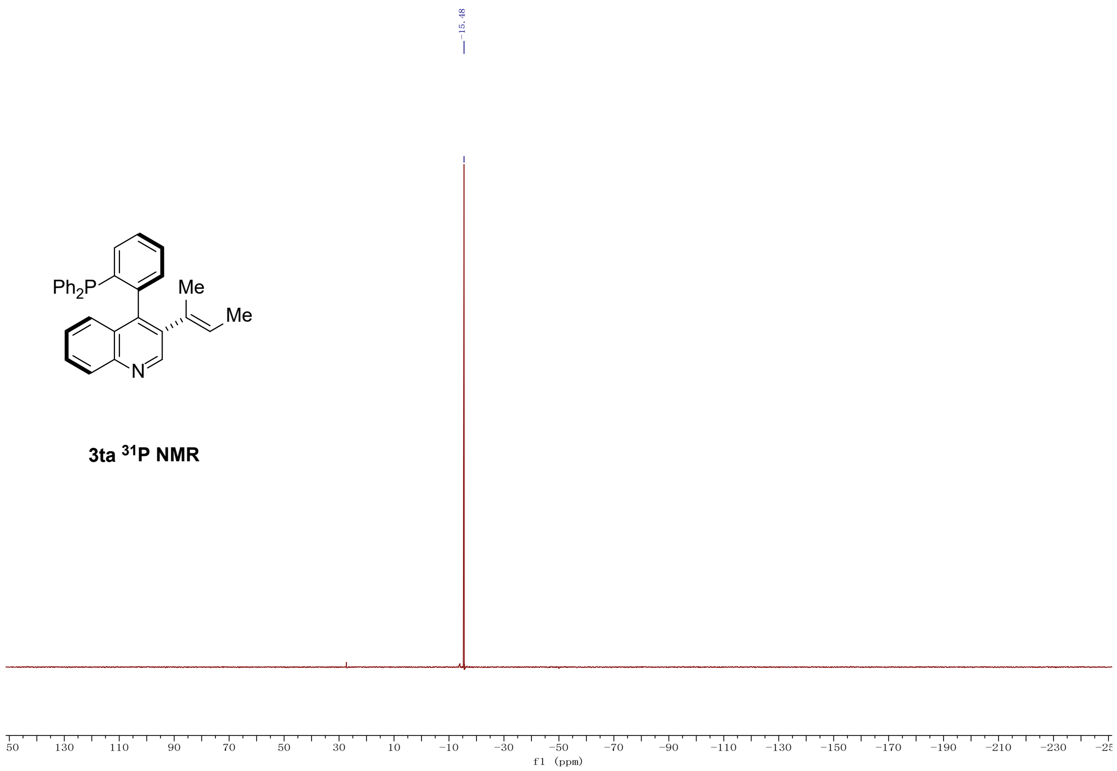

Supplementary Fig. 149. ^{31}P NMR spectra (202 MHz, $CDCl_3$, 25 °C) of **3ta**

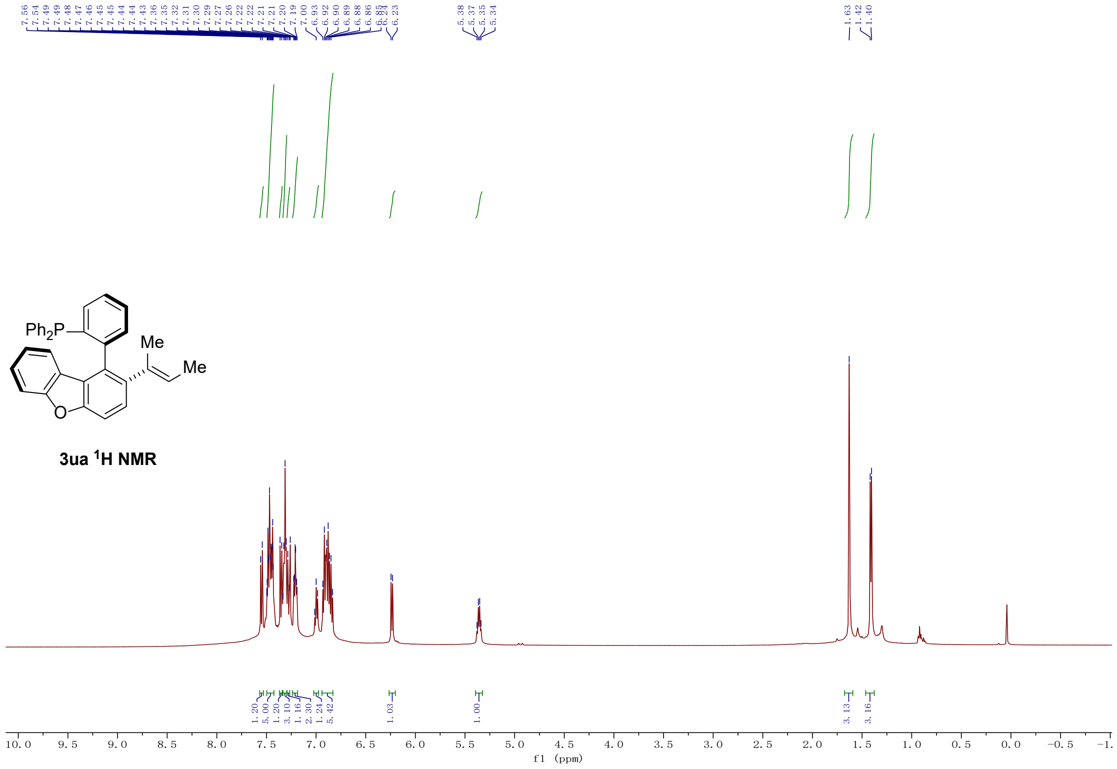

Supplementary Fig. 150. ^{1}H NMR spectra (500 MHz, $CDCl_3$, 25 °C) of **3ua**

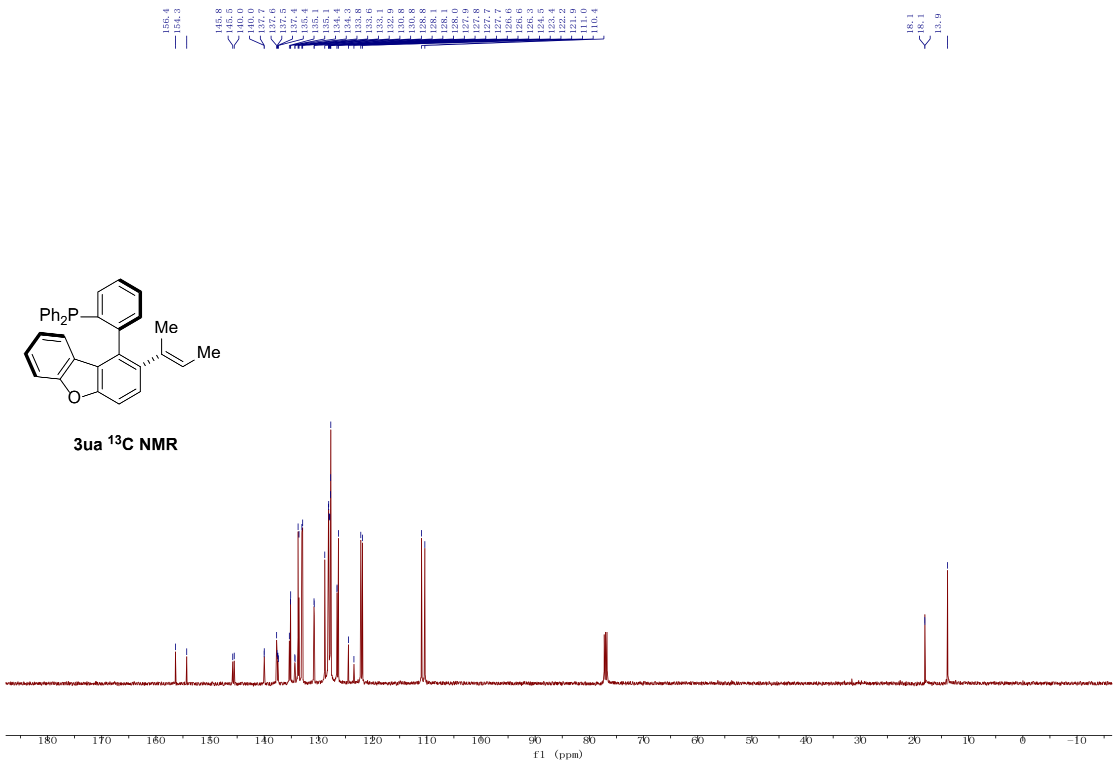

Supplementary Fig. 151. ^{13}C NMR spectra (126 MHz, $CDCl_3$, 25 °C) of **3ua**

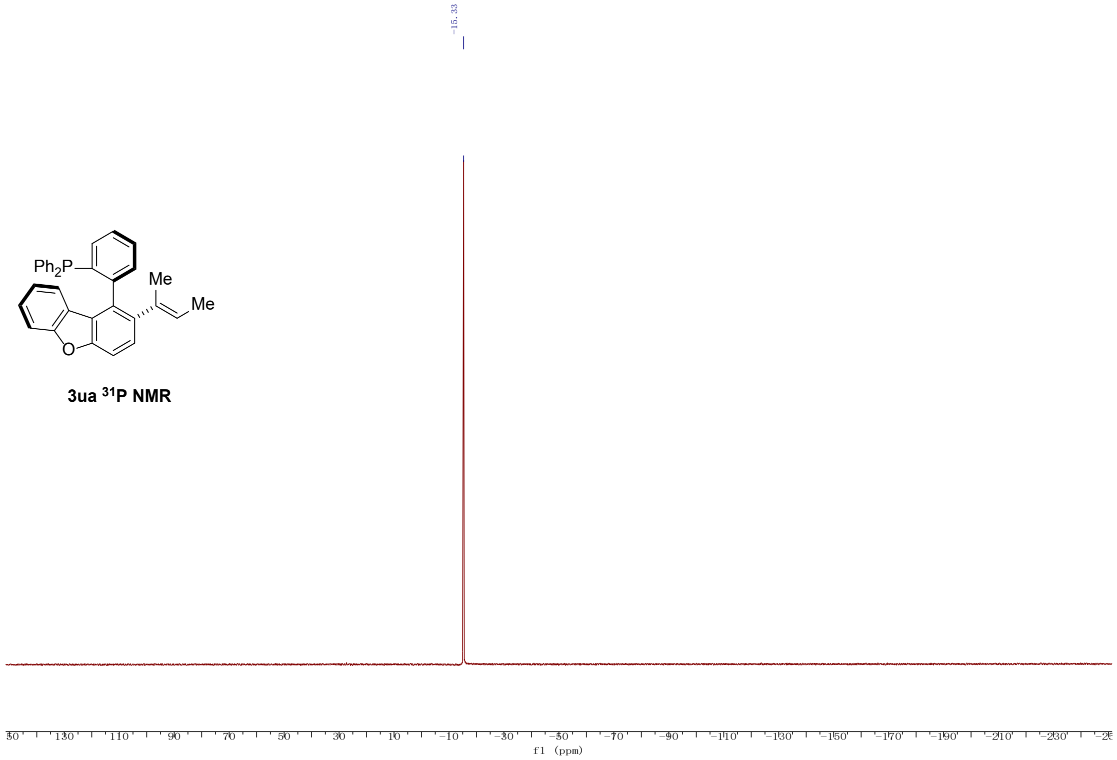

Supplementary Fig. 152. ^{31}P NMR spectra (202 MHz, $CDCl_3$, 25 °C) of **3ua**

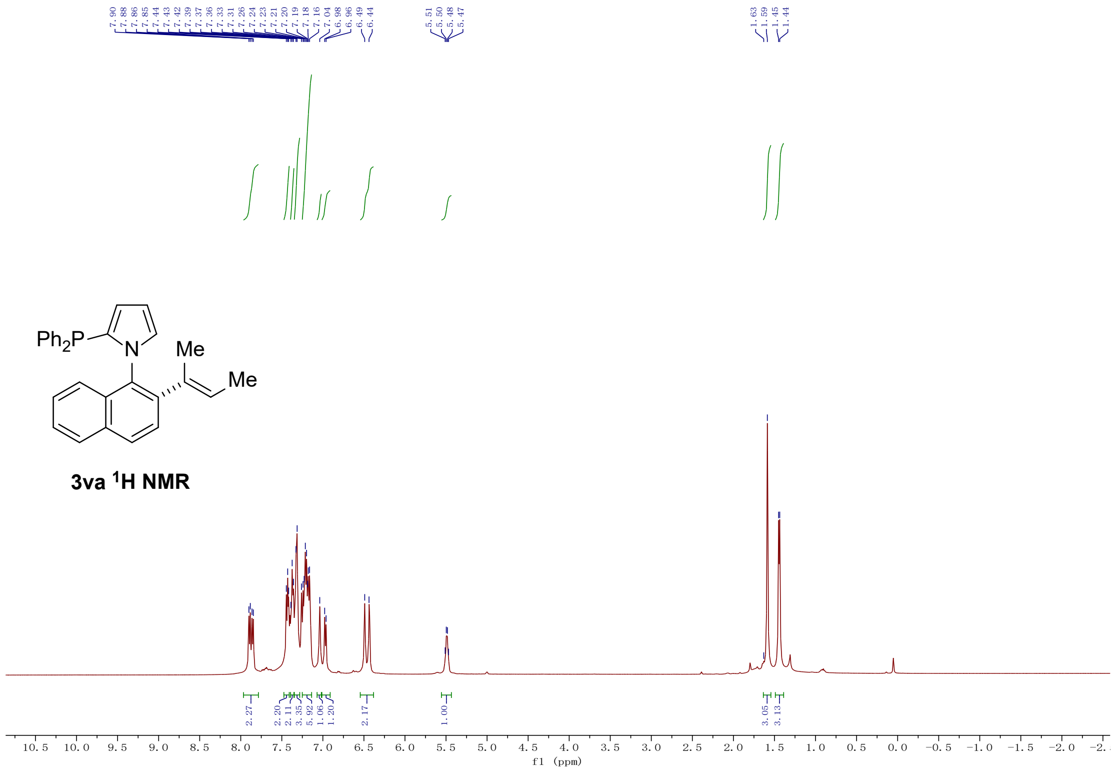

Supplementary Fig. 153. ^{1}H NMR spectra (500 MHz, CDCl₃, 25 °C) of **3va**

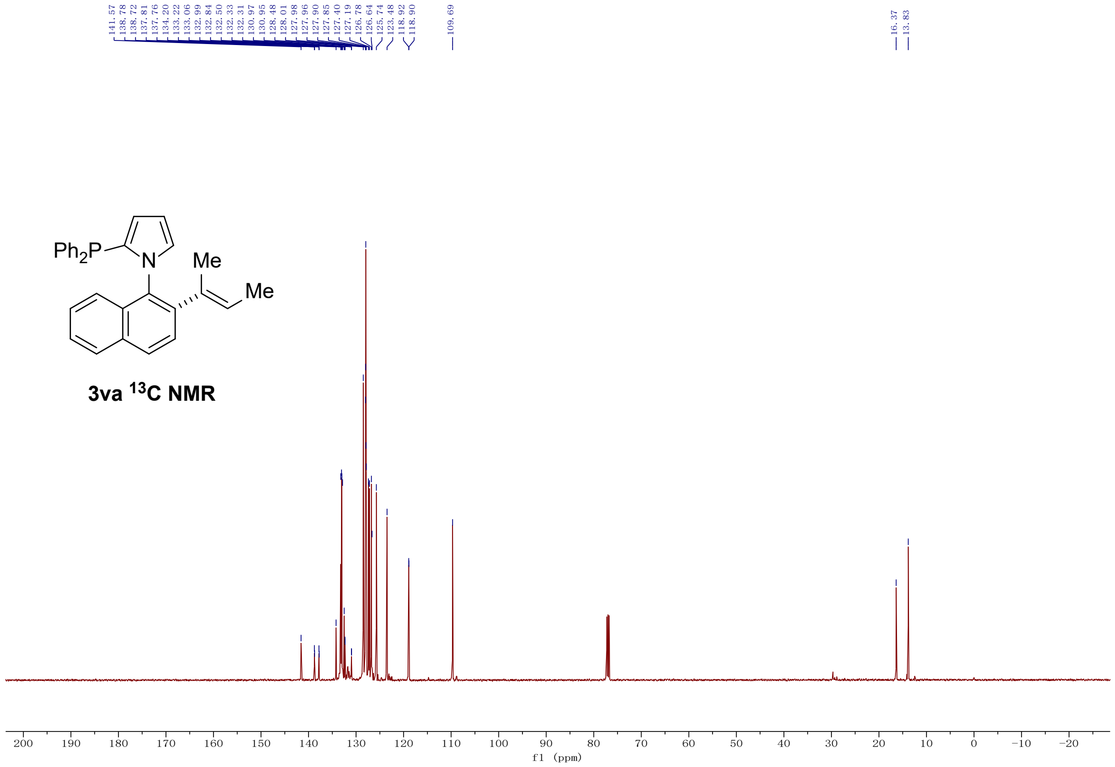

Supplementary Fig. 154. ^{13}C NMR spectra (126 MHz, CDCl₃, 25 °C) of **3va**

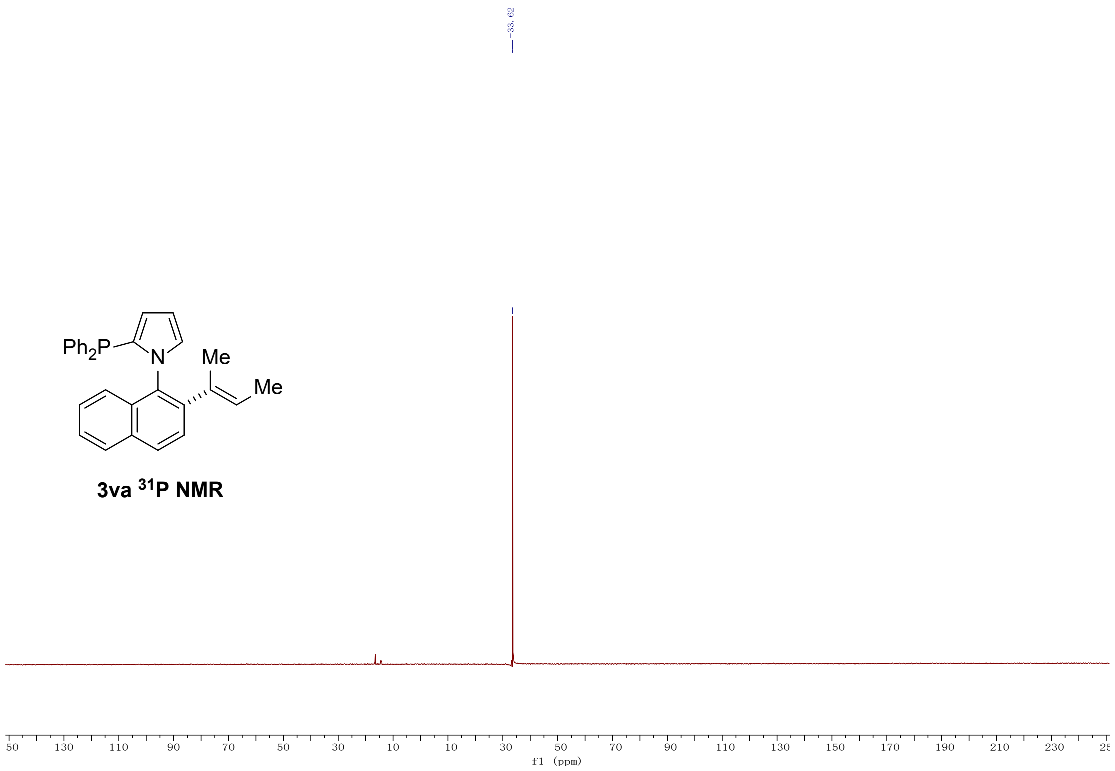

Supplementary Fig. 155. ^{31}P NMR spectra (202 MHz, CDCl₃, 25 °C) of **3va**

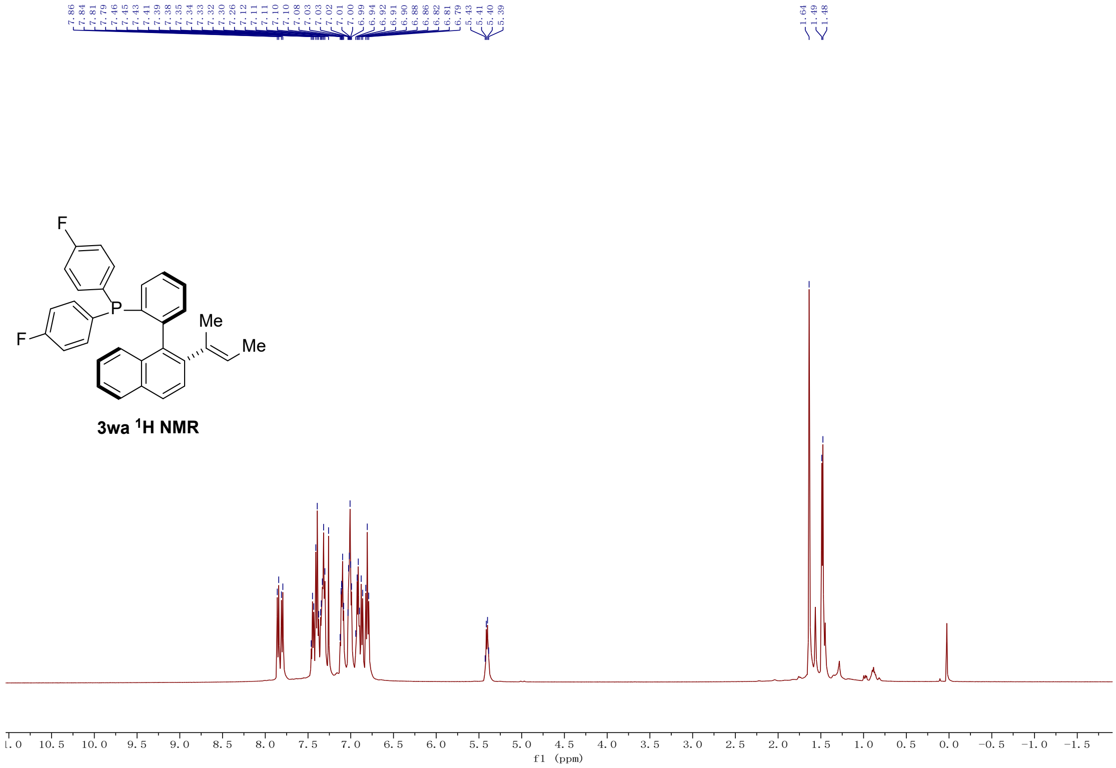

Supplementary Fig. 156. ^{1}H NMR spectra (500 MHz, CDCl₃, 25 °C) of **3wa**

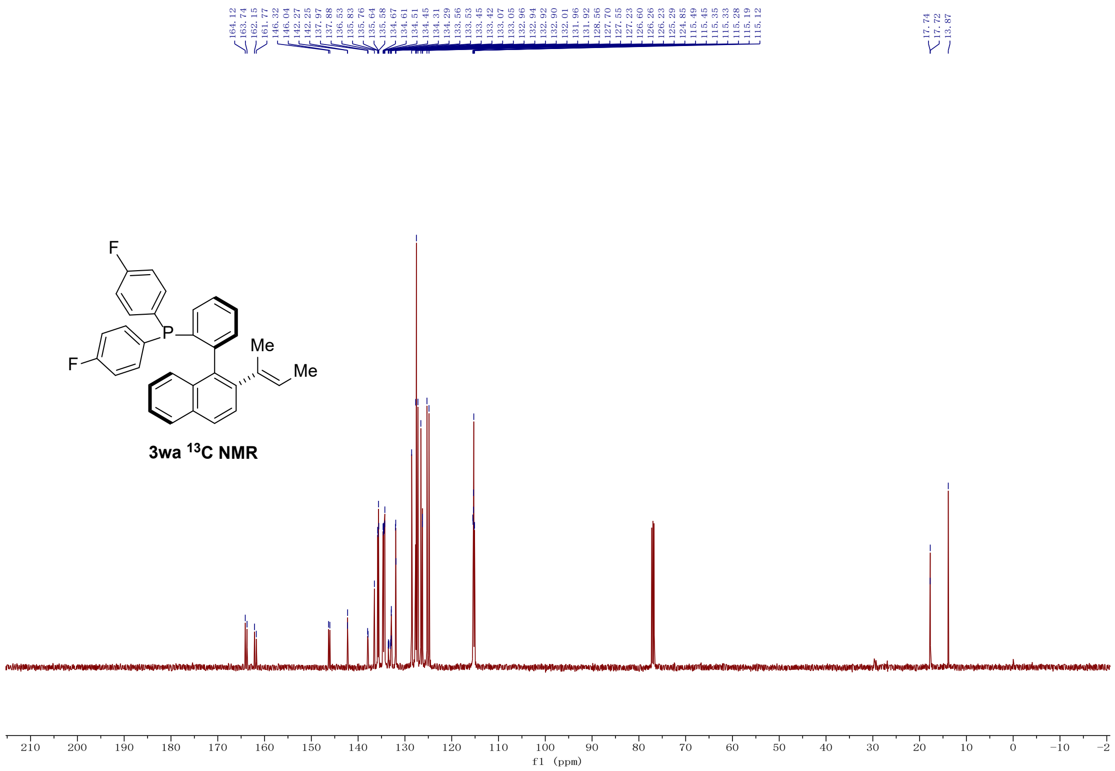

Supplementary Fig. 157. ^{13}C NMR spectra (126 MHz, $CDCl_3$, 25 ºC) of **3wa**

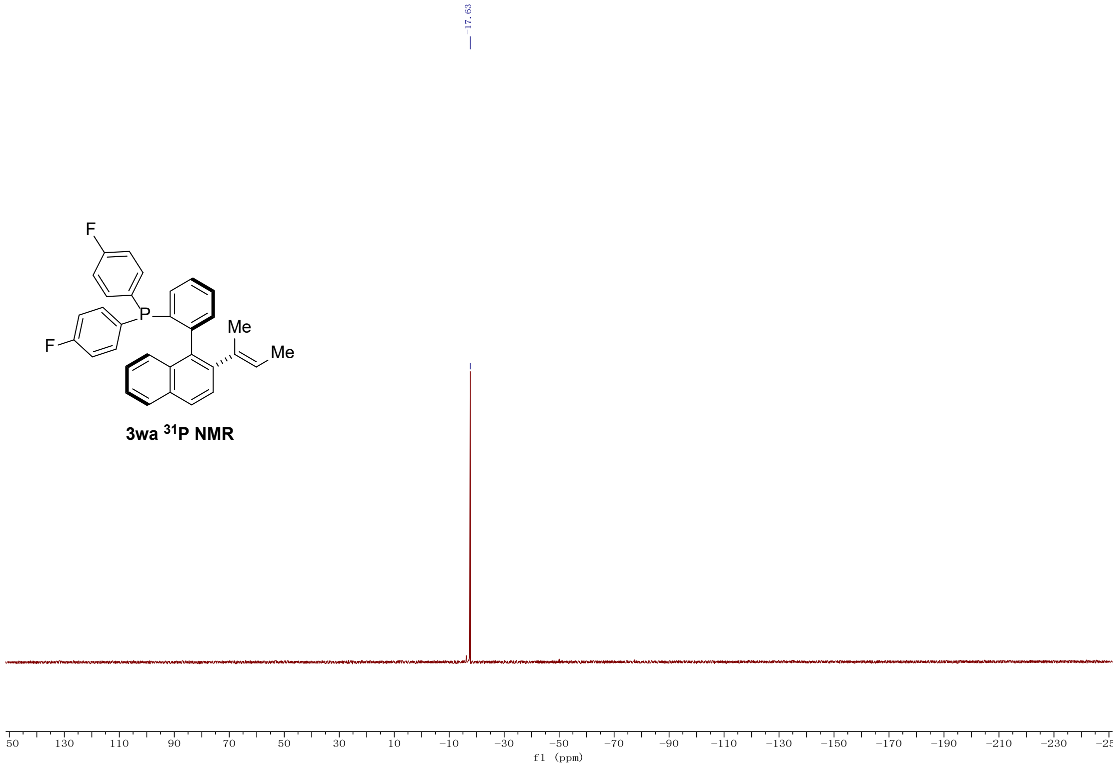

Supplementary Fig. 158. ^{31}P NMR spectra (202 MHz, $CDCl_3$, 25 ºC) of **3wa**

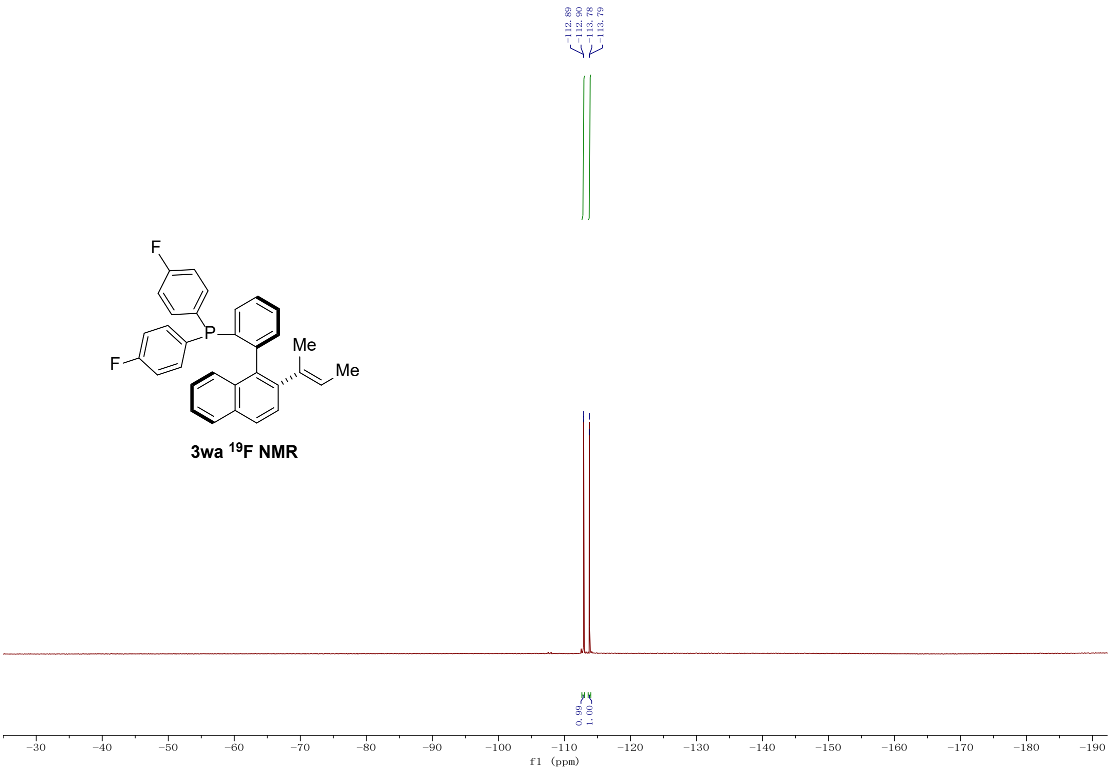

Supplementary Fig. 159. ^{19}F NMR spectra (471 MHz, $CDCl_3$, 25 ºC) of **3wa**

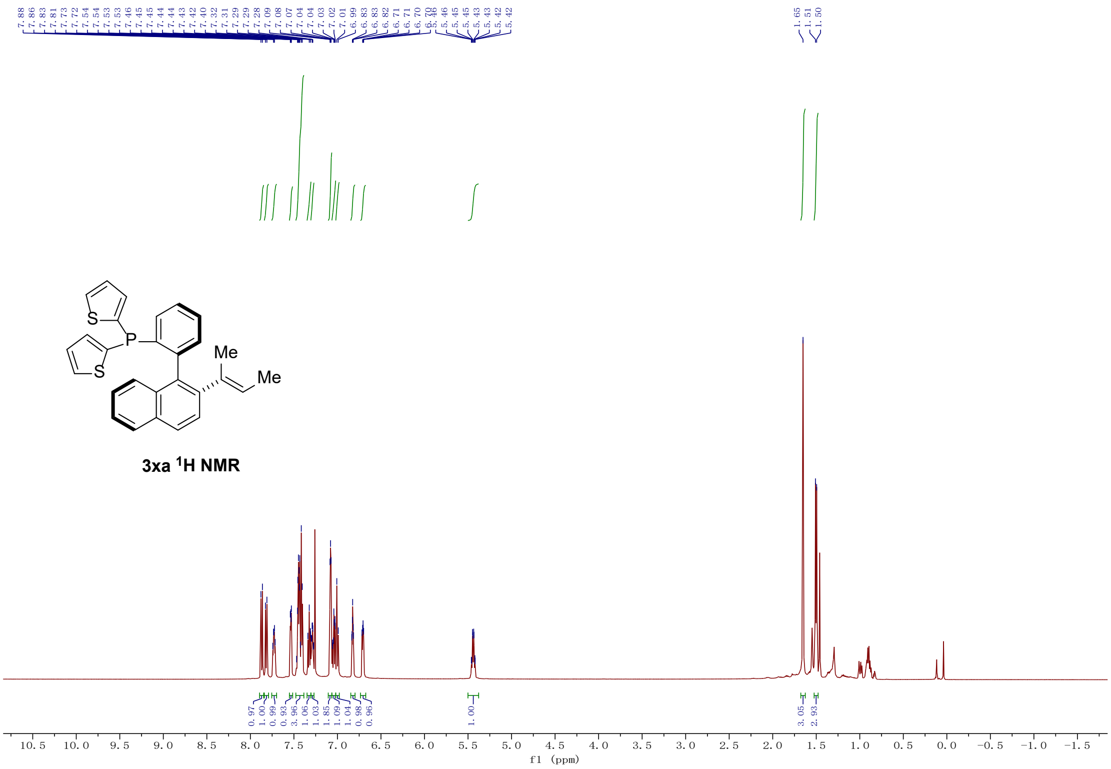

Supplementary Fig. 160. ^{1}H NMR spectra (500 MHz, $CDCl_3$, 25 ºC) of **3xa**

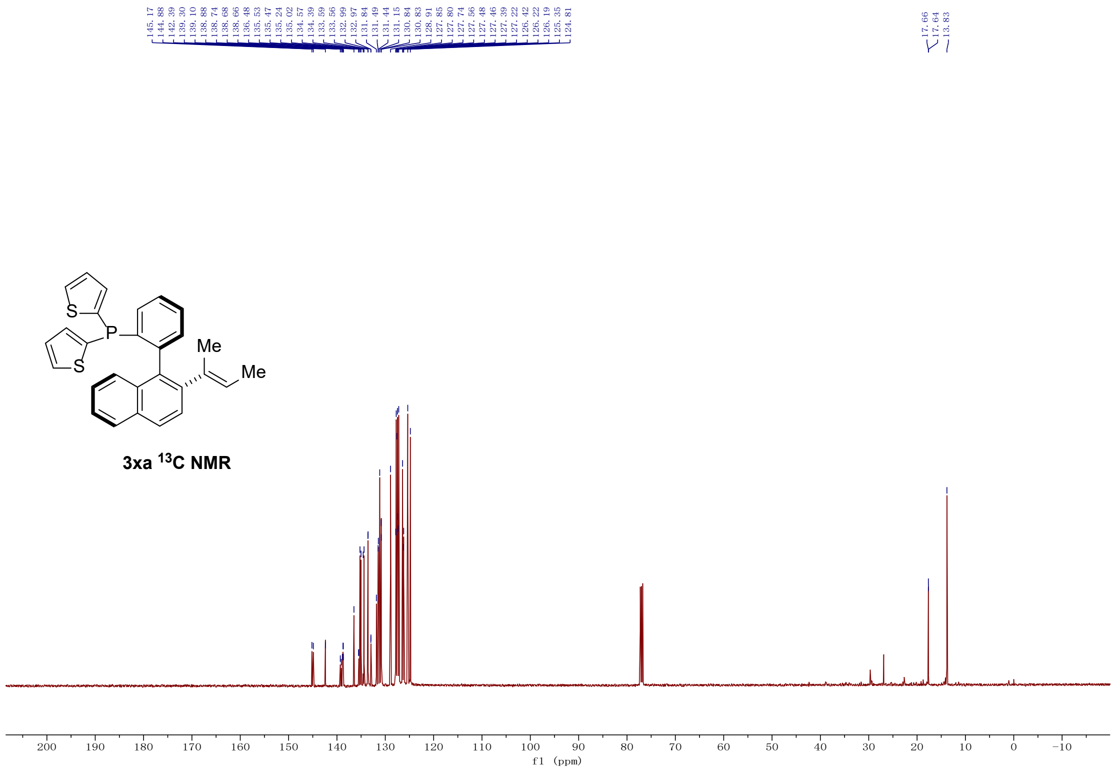

Supplementary Fig. 161. ^{13}C NMR spectra (126 MHz, $CDCl_3$, 25 °C) of **3xa**

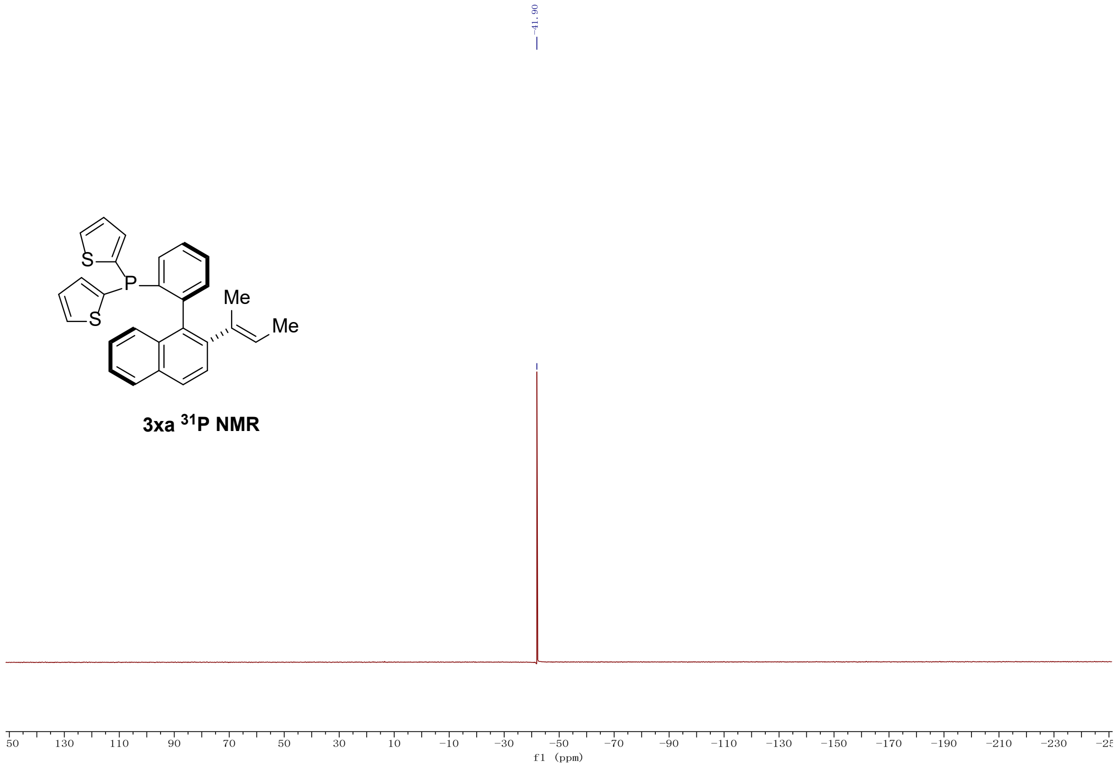

Supplementary Fig. 162. ^{31}P NMR spectra (202 MHz, $CDCl_3$, 25 °C) of **3xa**

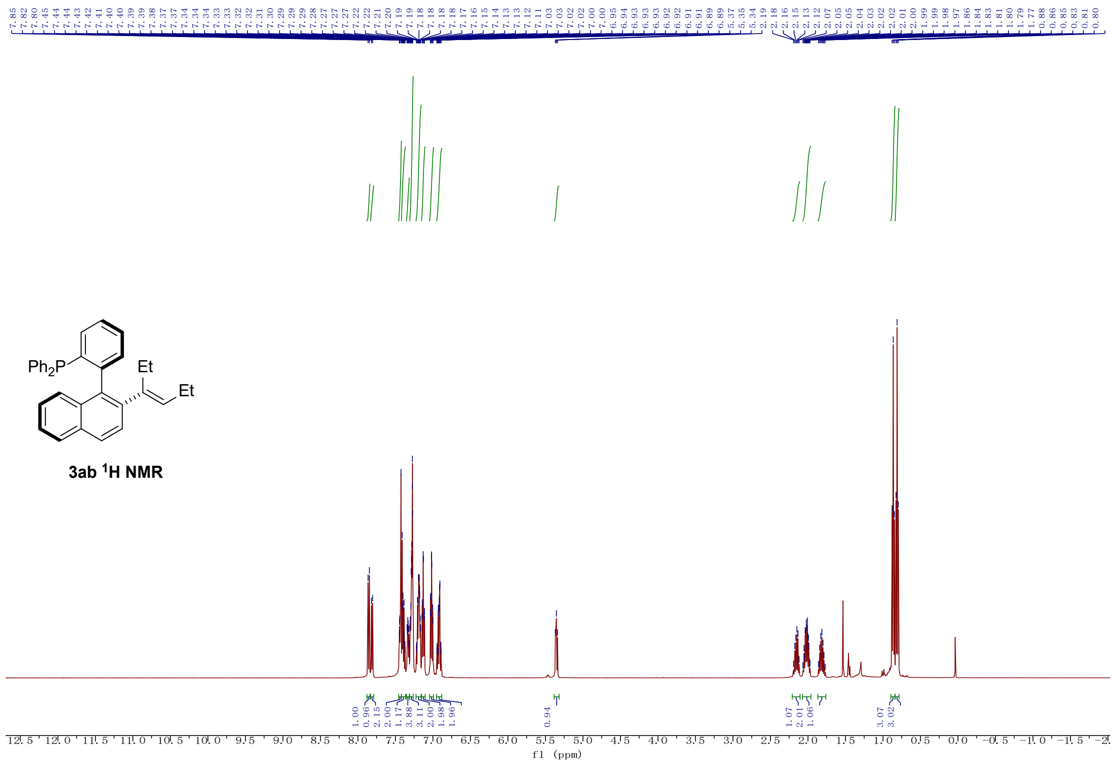

Supplementary Fig. 163. ^{1}H NMR spectra (500 MHz, CDCl$_3$, 25 °C) of **3ab**

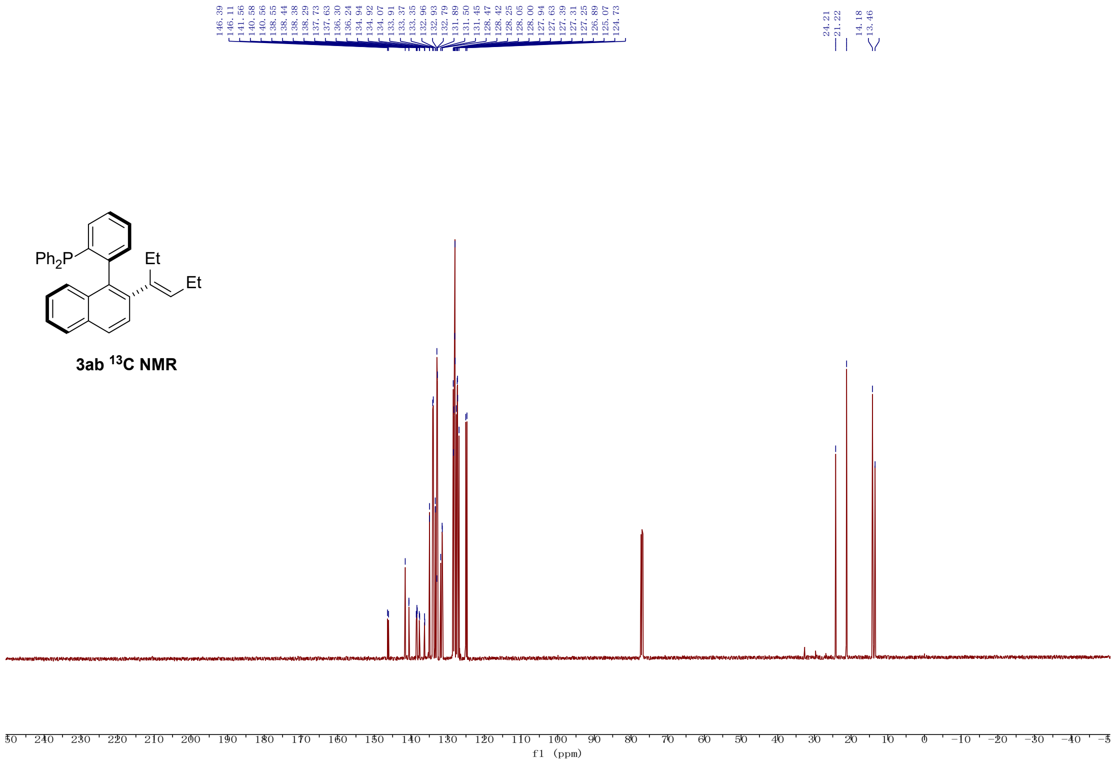

Supplementary Fig. 164. ^{13}C NMR spectra (126 MHz, CDCl$_3$, 25 °C) of **3ab**

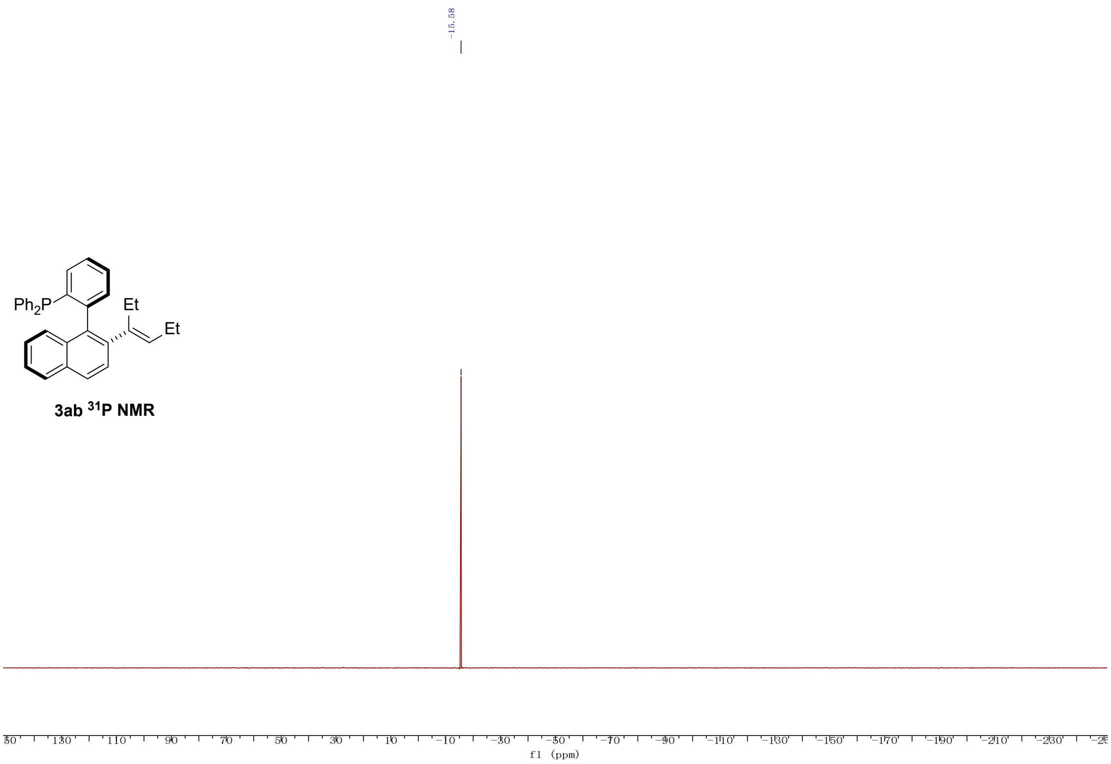

Supplementary Fig. 165. ^{31}P NMR spectra (202 MHz, $CDCl_3$, 25 °C) of **3ab**

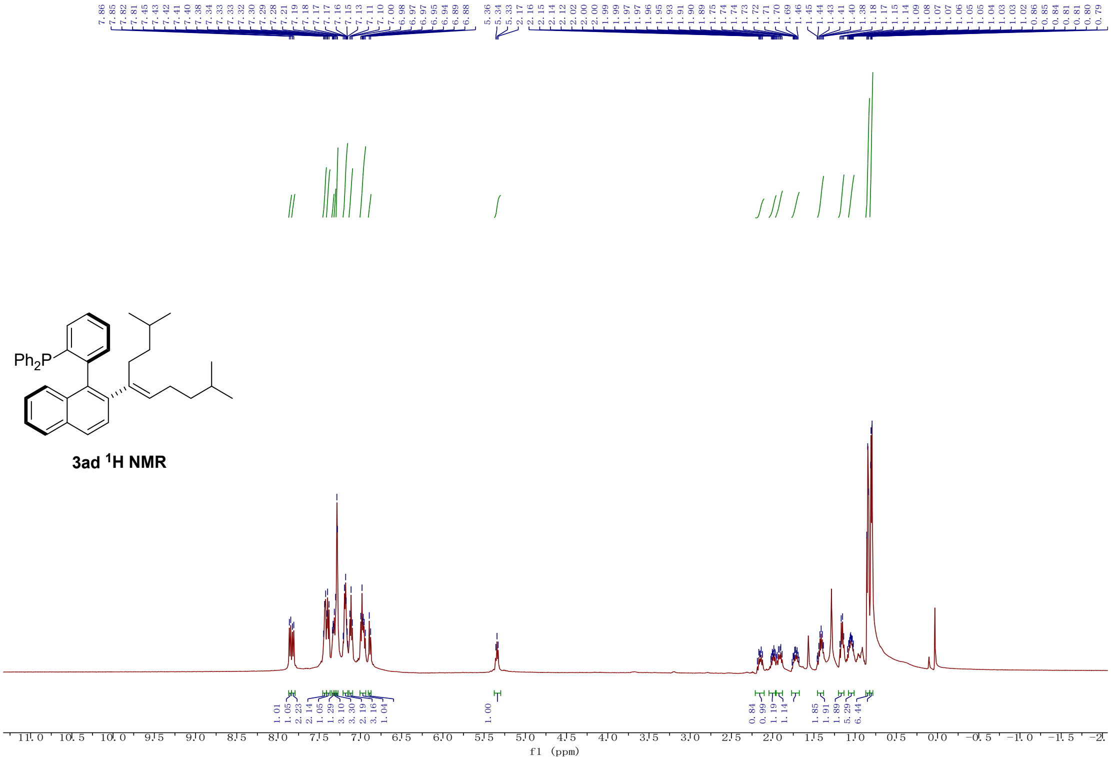

Supplementary Fig. 166. ^{1}H NMR spectra (500 MHz, $CDCl_3$, 25 °C) of **3ad**

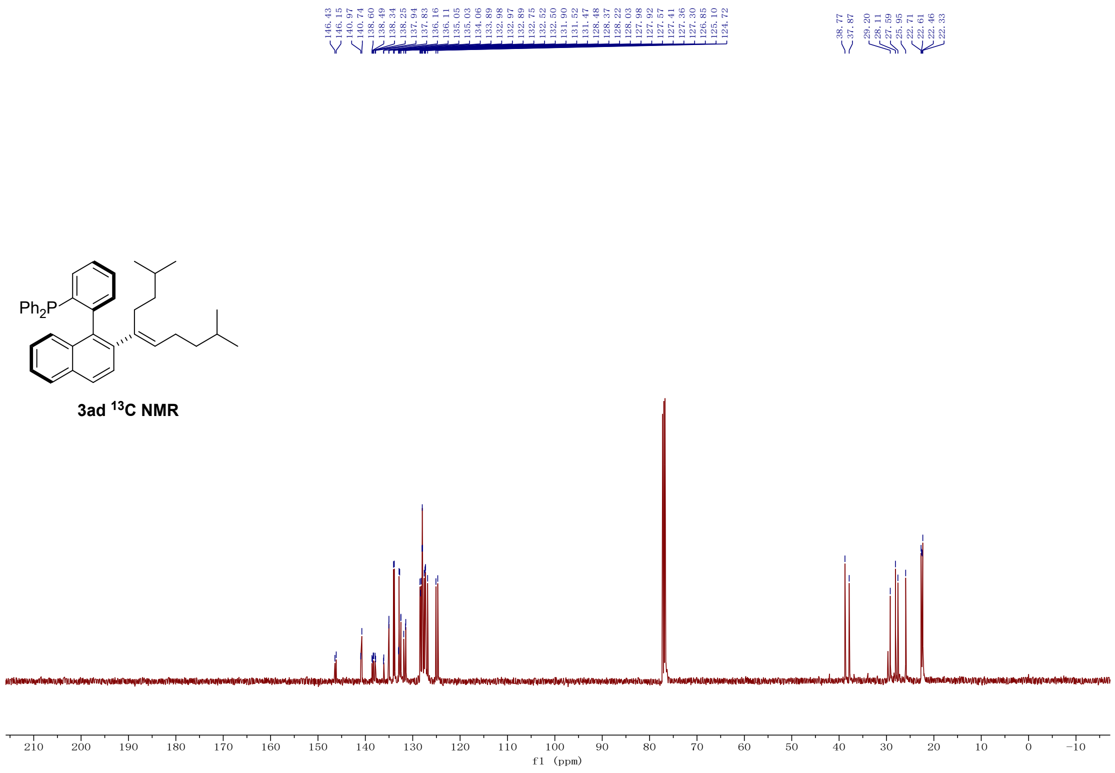

Supplementary Fig. 167. ^{13}C NMR spectra (126 MHz, $CDCl_3$, 25 °C) of **3ad**

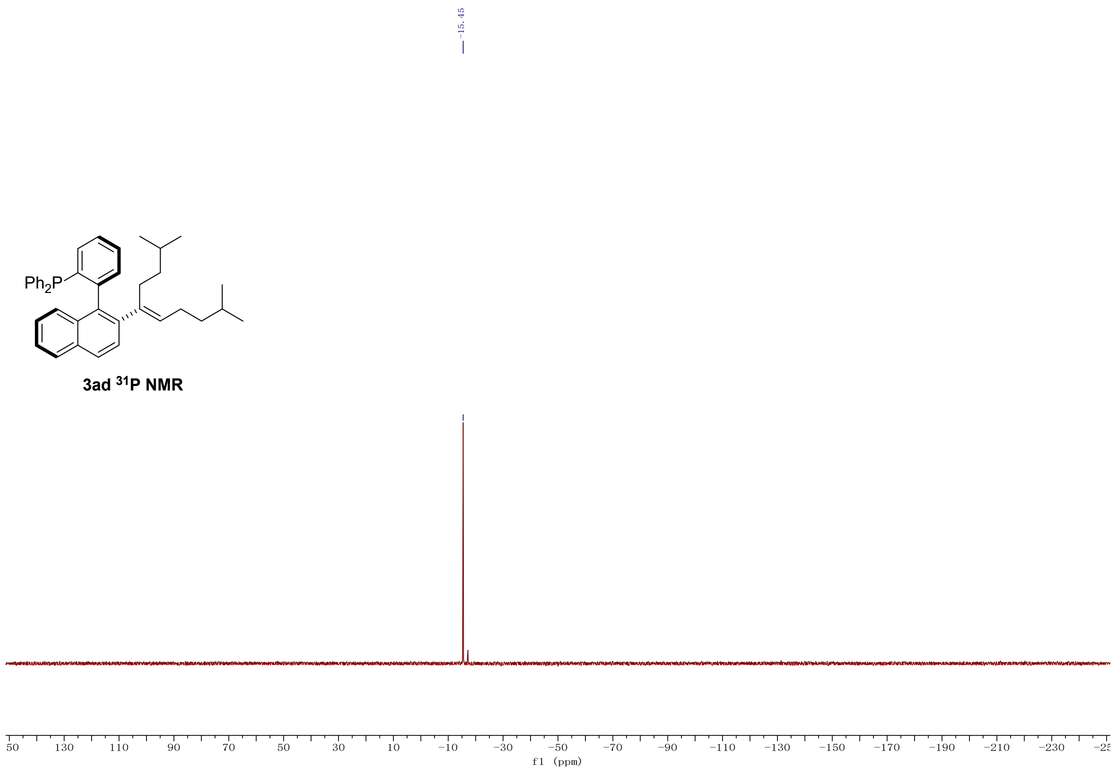

Supplementary Fig. 168. ^{31}P NMR spectra (202 MHz, $CDCl_3$, 25 °C) of **3ad**

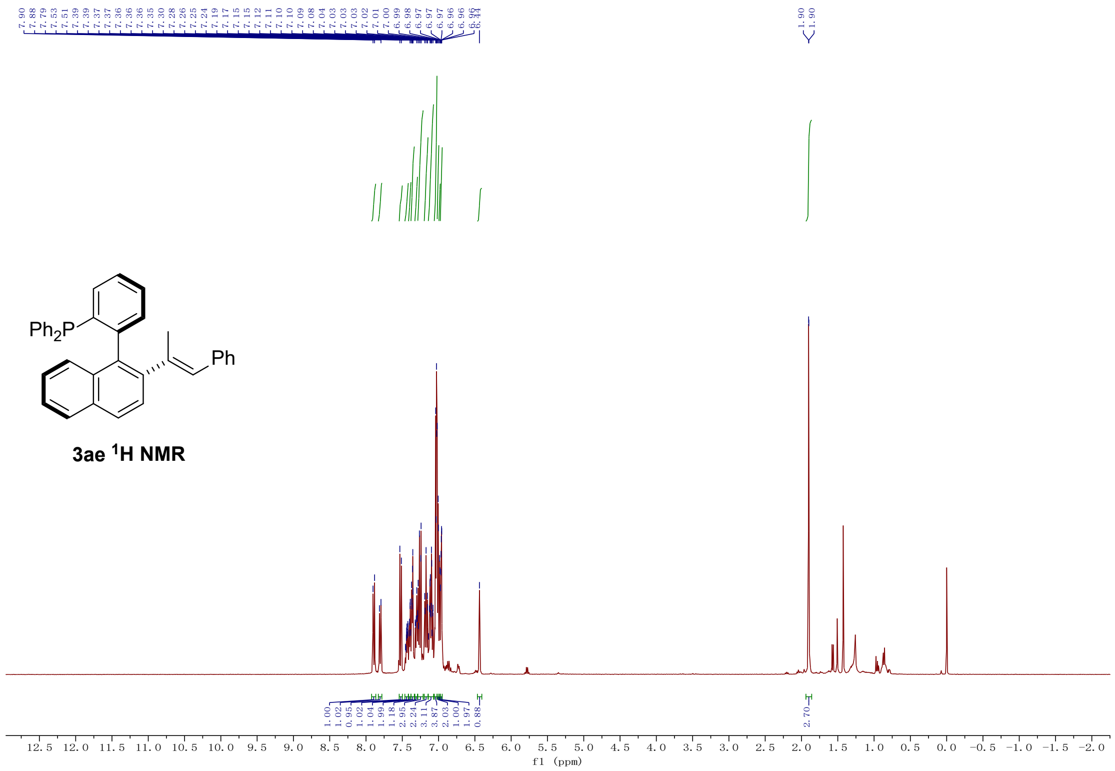

Supplementary Fig. 169. ^{1}H NMR spectra (500 MHz, CDCl$_3$, 25 °C) of **3ae**

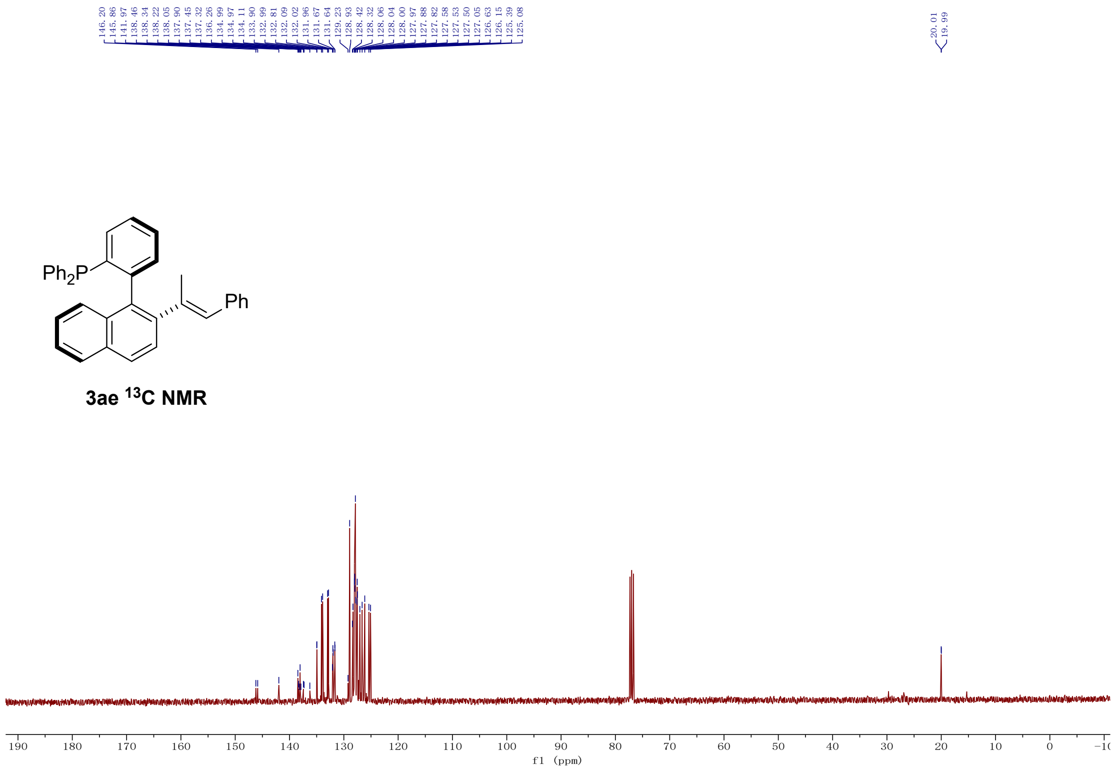

Supplementary Fig. 170. ^{13}C NMR spectra (101 MHz, CDCl$_3$, 25 °C) of **3ae**

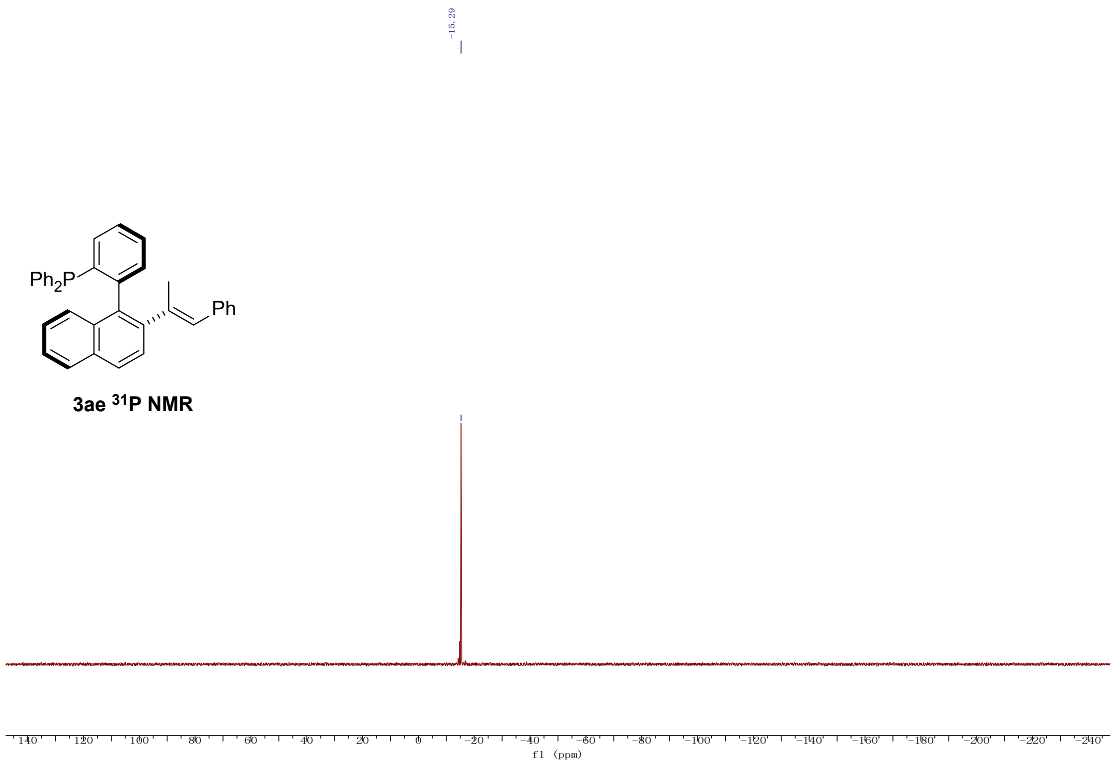

Supplementary Fig. 171. ^{31}P NMR spectra (162 MHz, $CDCl_3$, 25 °C) of **3ae**

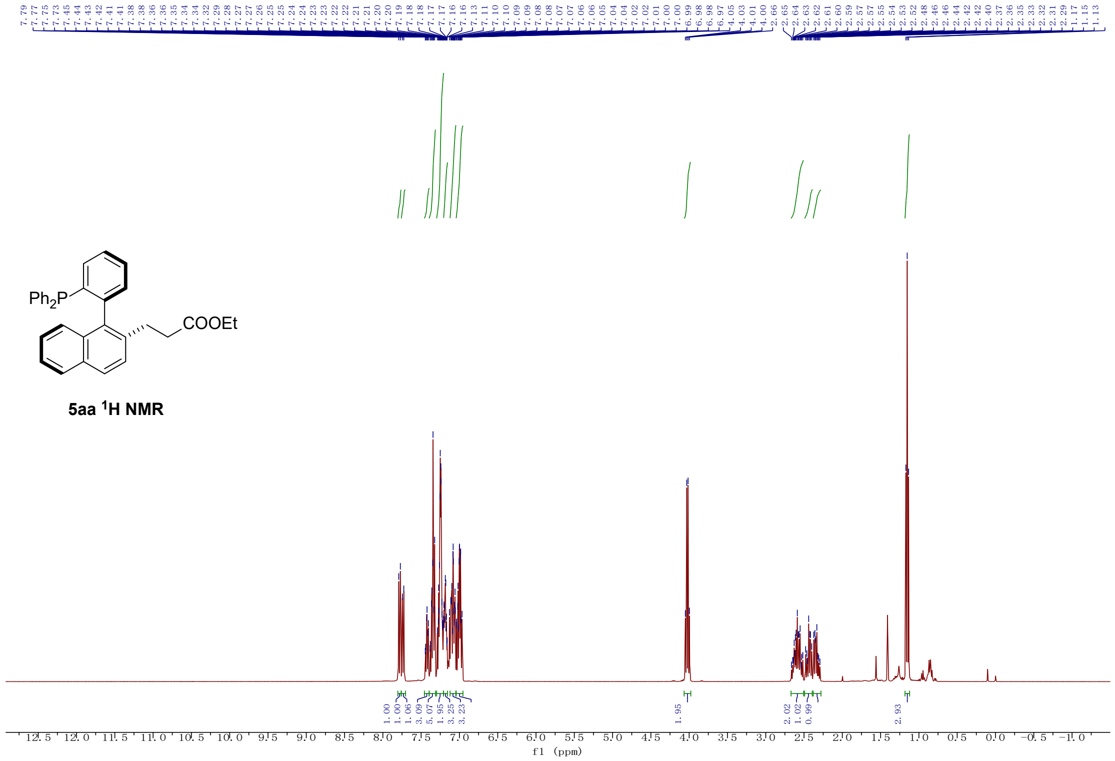

Supplementary Fig. 172 ^{1}H NMR spectra (400 MHz, $CDCl_3$, 25 °C) of **5aa**

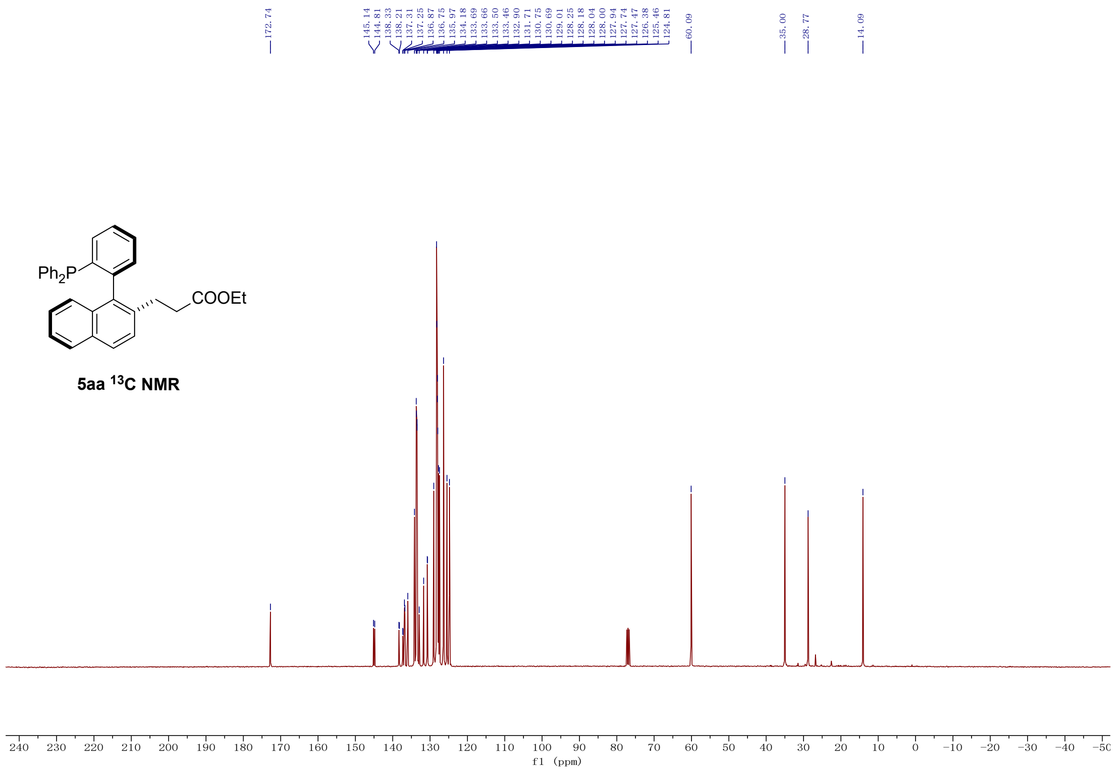

Supplementary Fig. 173. ^{13}C NMR spectra (101 MHz, $CDCl_3$, 25 ºC) of **5aa**

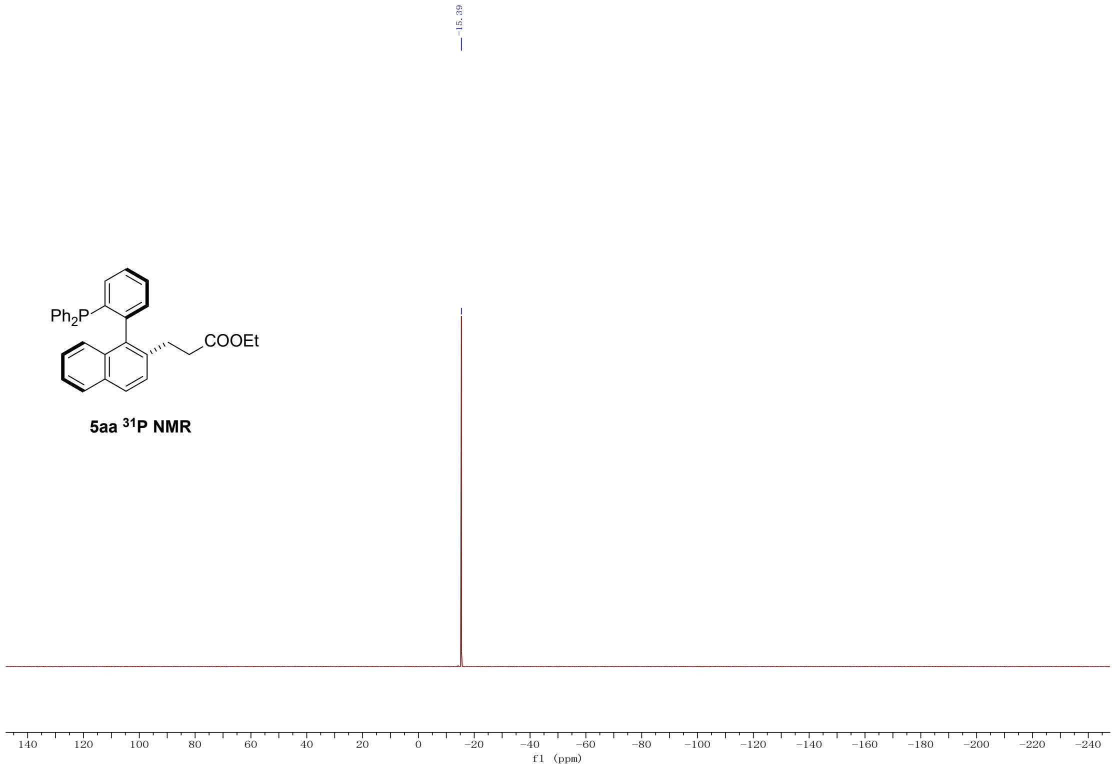

Supplementary Fig. 174. ^{31}P NMR spectra (162 MHz, $CDCl_3$, 25 ºC) of **5aa**

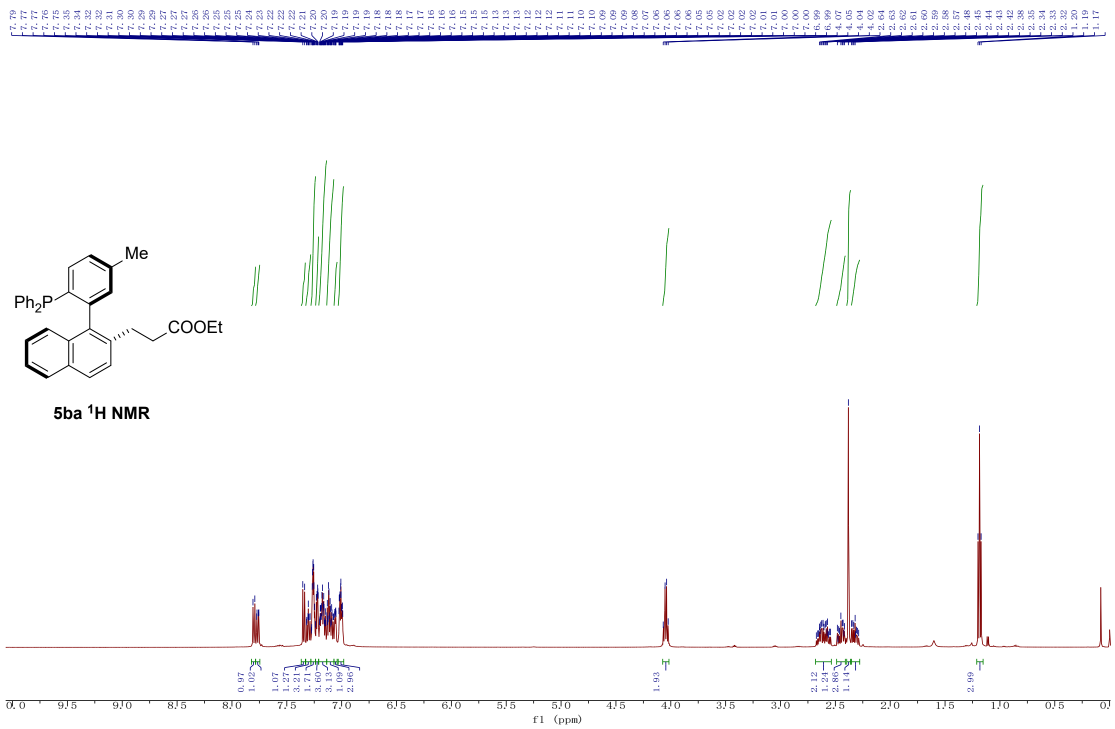

Supplementary Fig. 175 ^{1}H NMR spectra (500 MHz, $CDCl_3$, 25 °C) of **5ba**

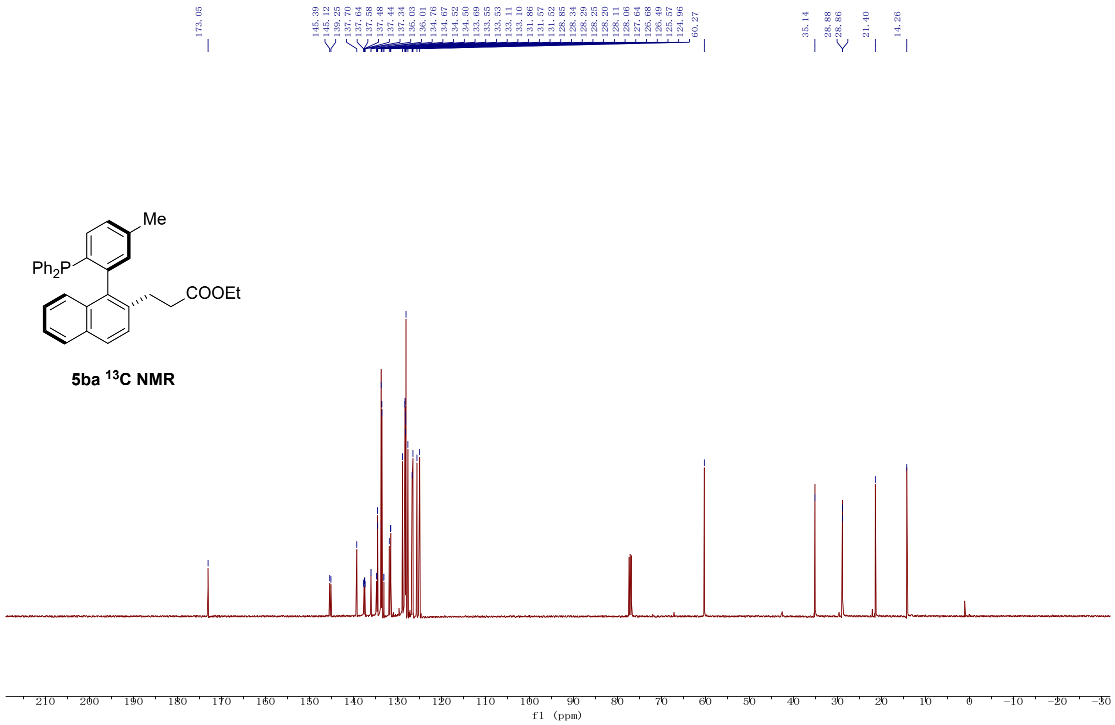

Supplementary Fig. 176. ^{13}C NMR spectra (126 MHz, $CDCl_3$, 25 °C) of **5ba**

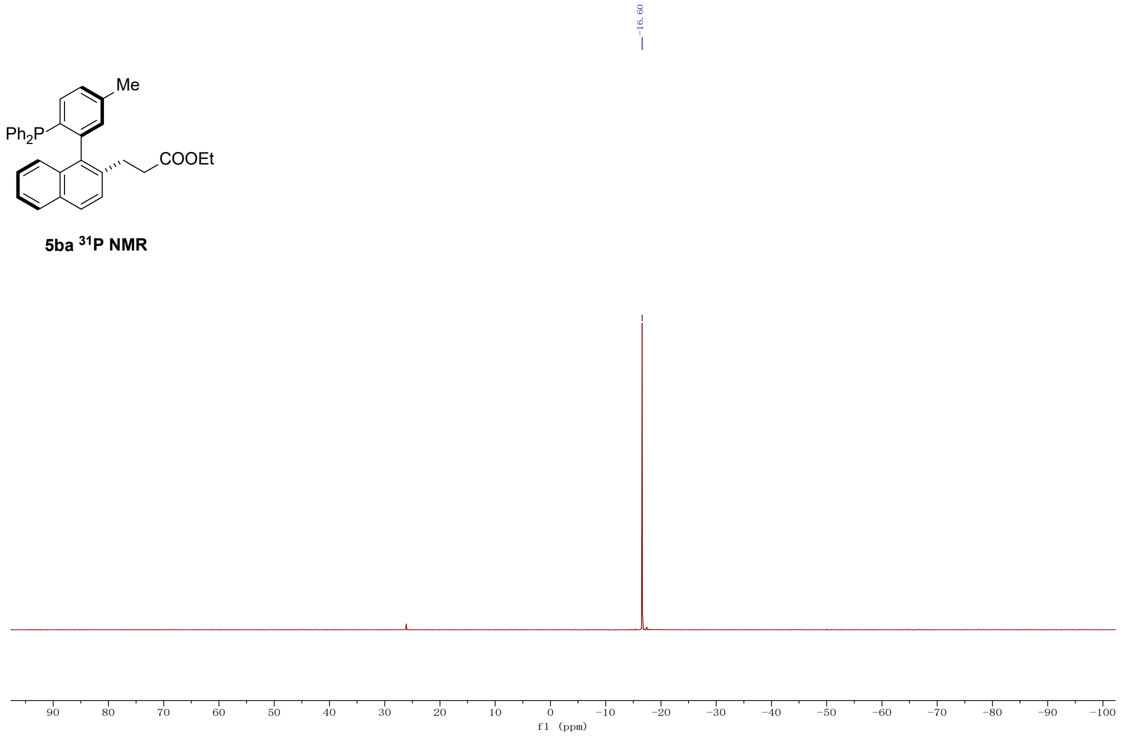

Supplementary Fig. 177. ^{31}P NMR spectra (202 MHz, $CDCl_3$, 25 °C) of **5ba**

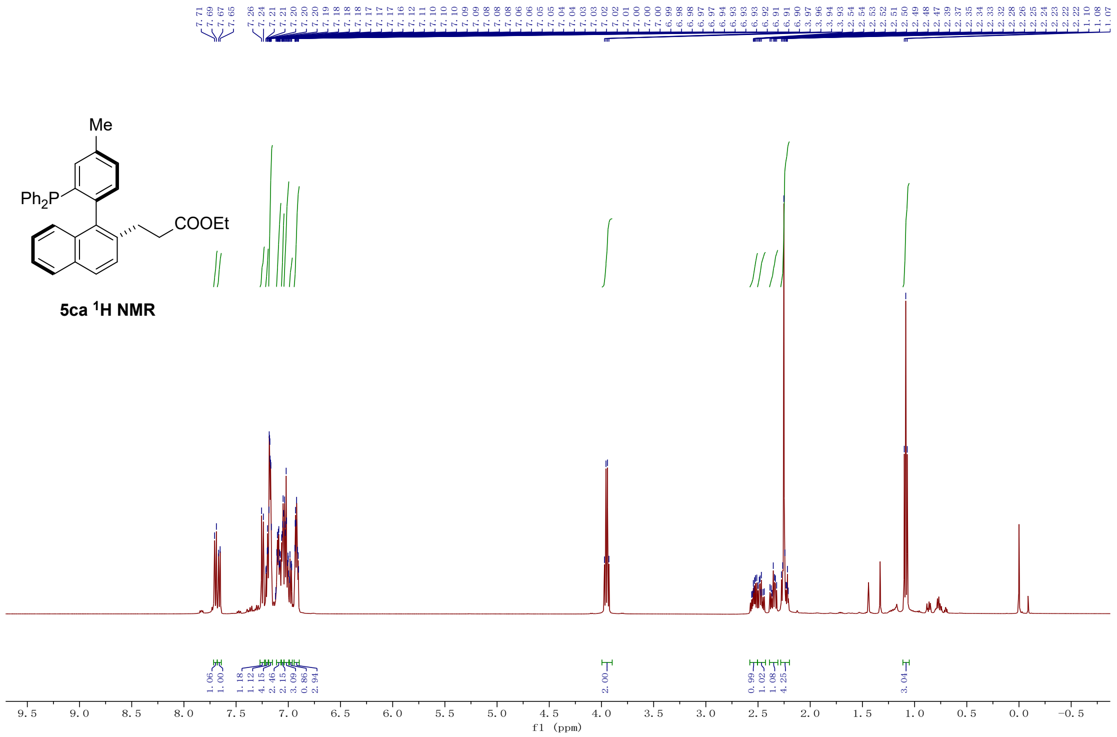

Supplementary Fig. 178 ^{1}H NMR spectra (500 MHz, $CDCl_3$, 25 °C) of **5ca**

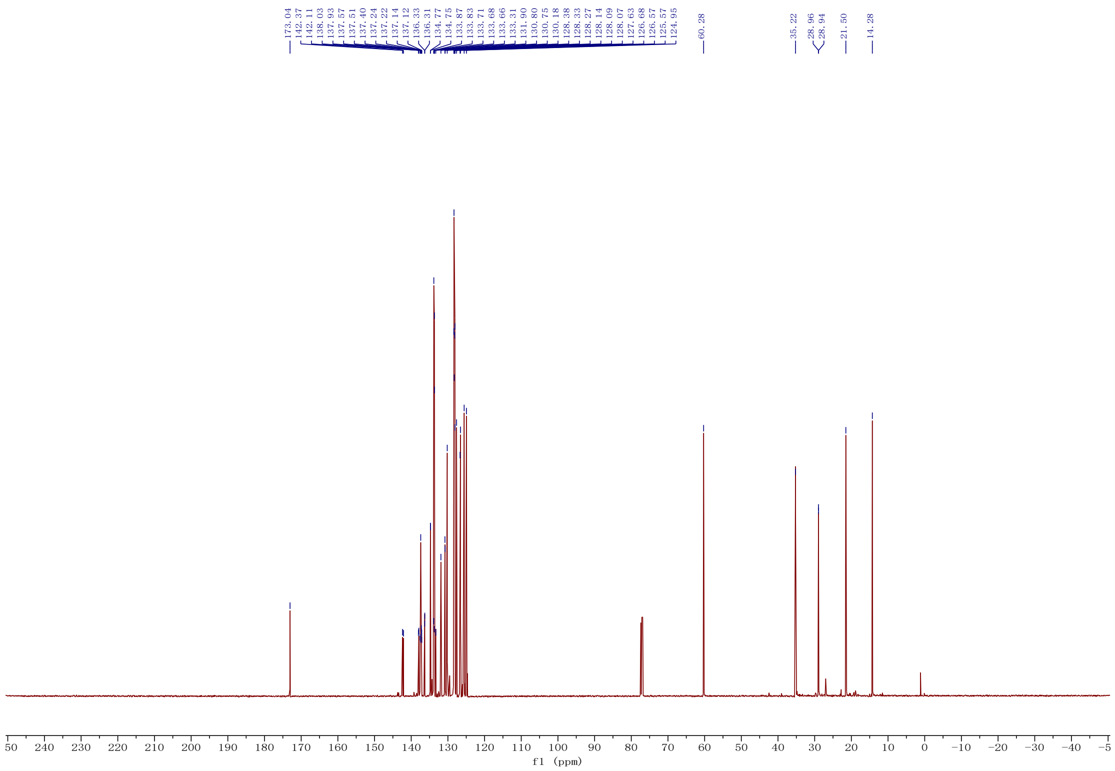

Supplementary Fig. 179. ^{13}C NMR spectra (126 MHz, $CDCl_3$, 25 °C) of **5ca**

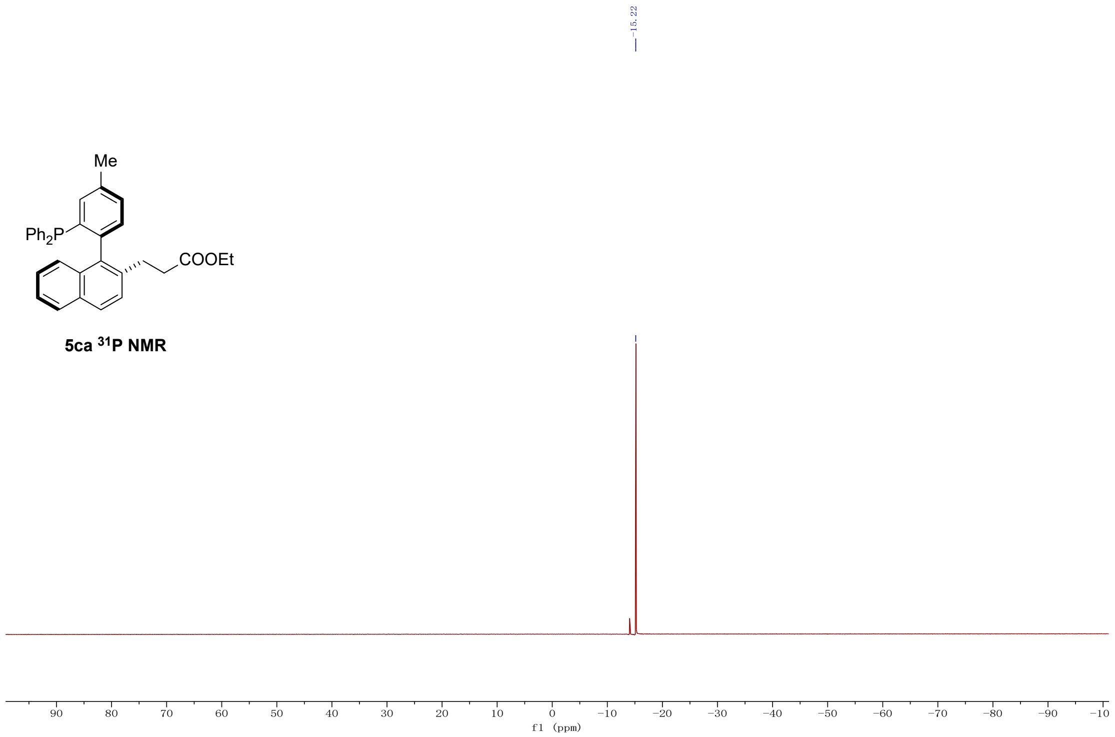

Supplementary Fig. 180. ^{31}P NMR spectra (202 MHz, $CDCl_3$, 25 °C) of **5ca**

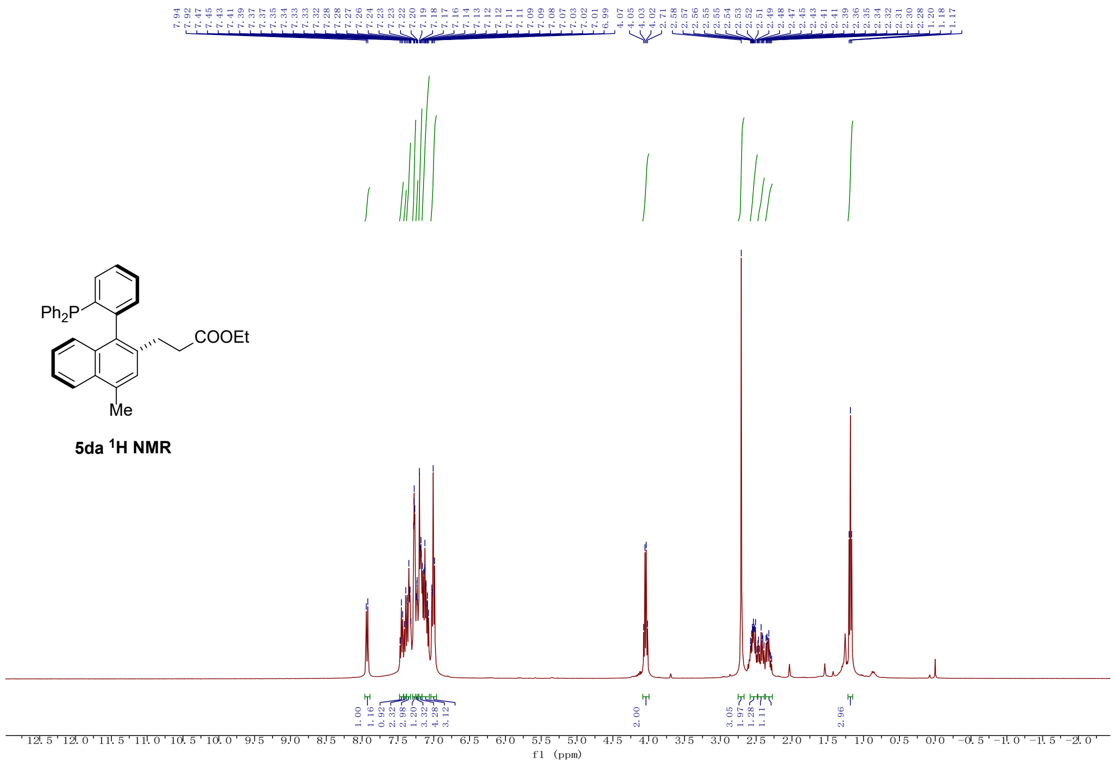

Supplementary Fig. 181 ^{1}H NMR spectra (400 MHz, $CDCl_3$, 25 ºC) of **5da**

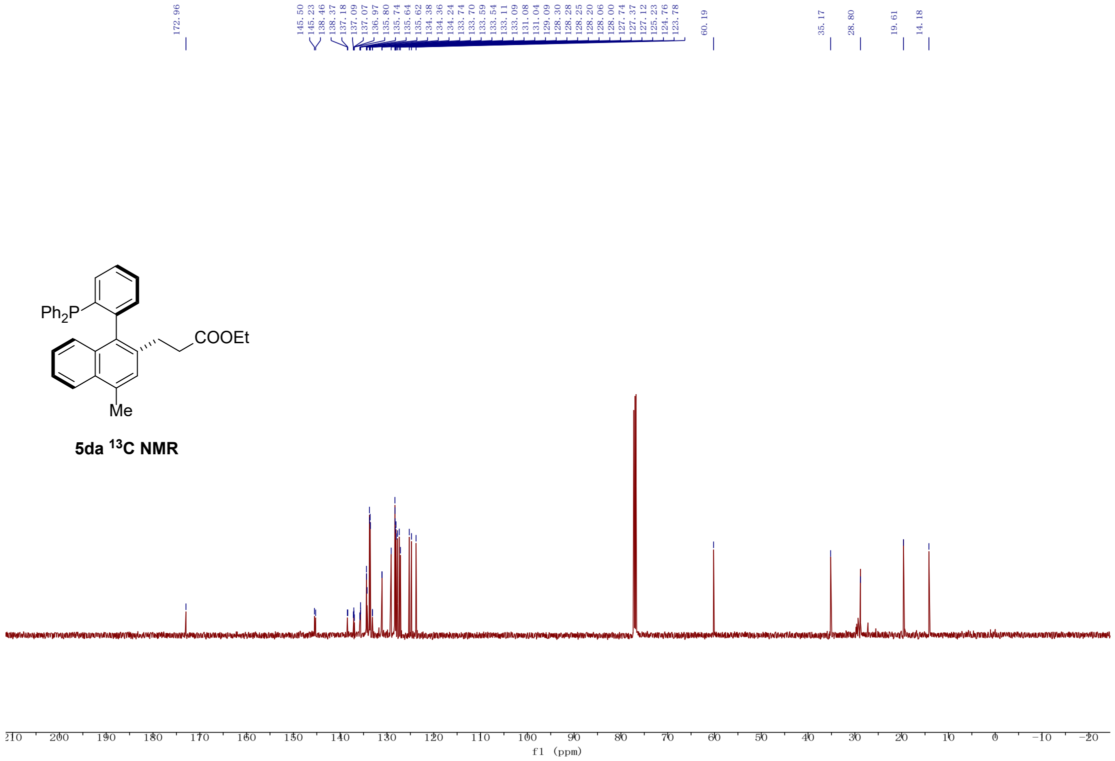

Supplementary Fig. 182. ^{13}C NMR spectra (126 MHz, $CDCl_3$, 25 ºC) of **5da**

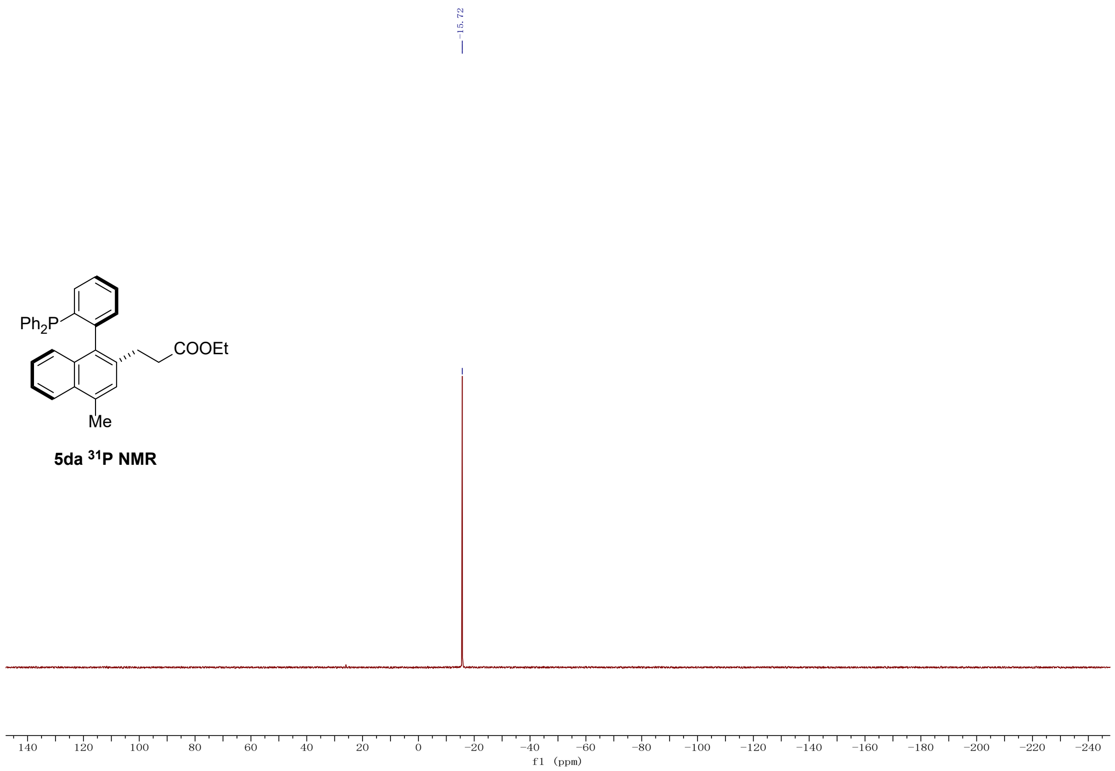

Supplementary Fig. 183. ^{31}P NMR spectra (162 MHz, $CDCl_3$, 25 °C) of **5da**

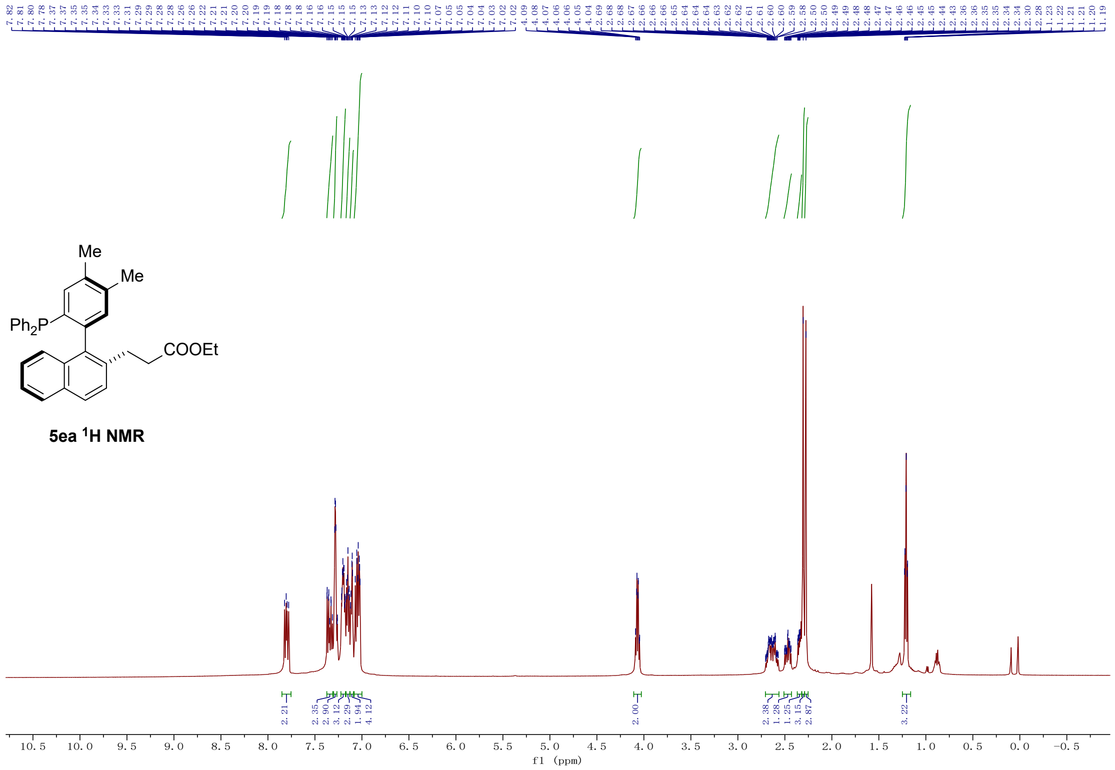

Supplementary Fig. 184 ^{1}H NMR spectra (500 MHz, $CDCl_3$, 25 °C) of **5ea**

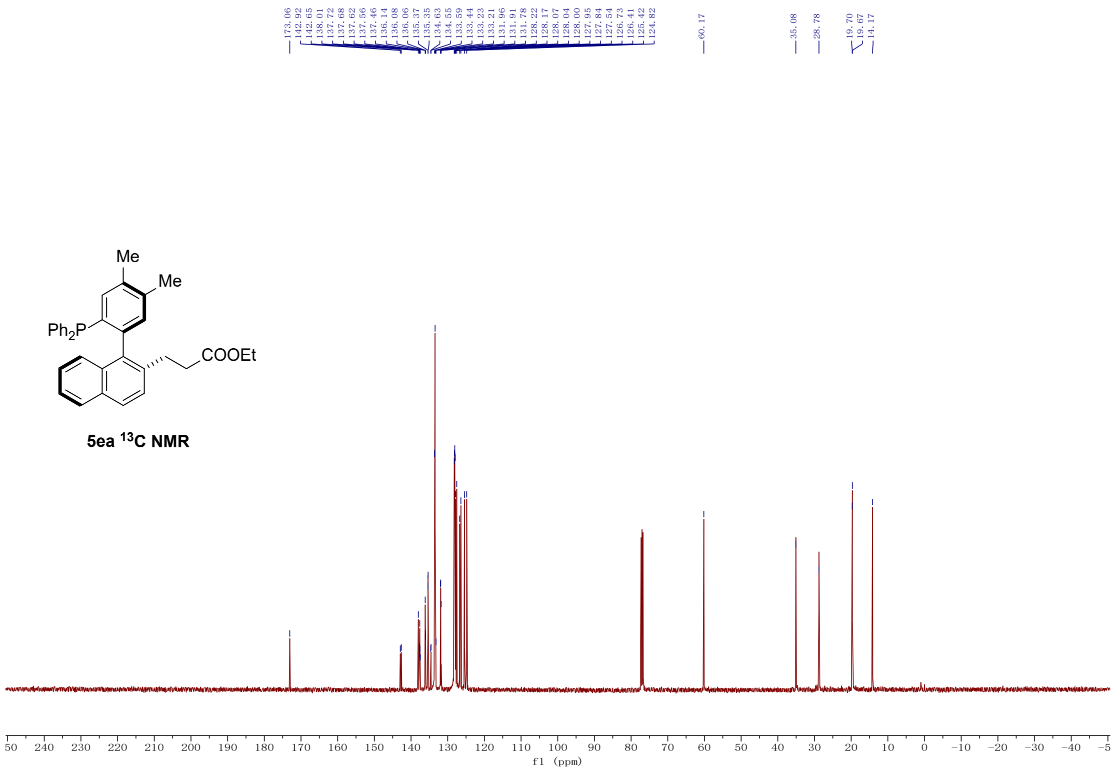

Supplementary Fig. 185. ^{13}C NMR spectra (126 MHz, $CDCl_3$, 25 °C) of **5ea**

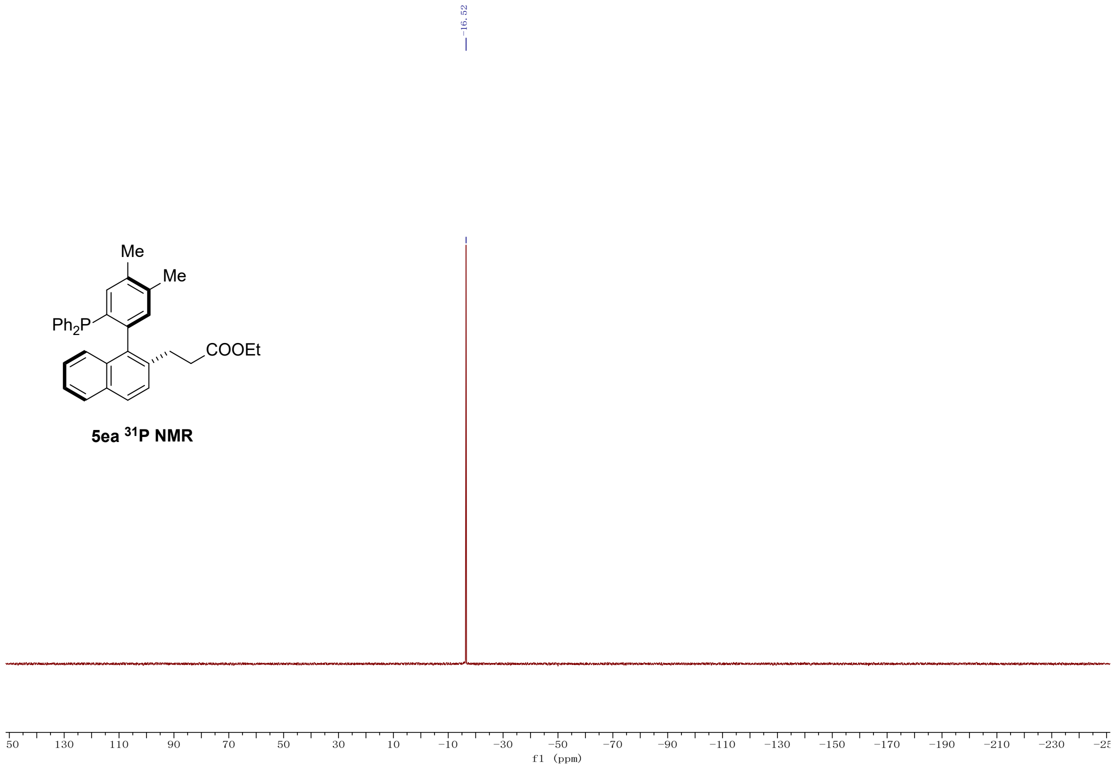

Supplementary Fig. 186. ^{31}P NMR spectra (202 MHz, $CDCl_3$, 25 °C) of **5ea**

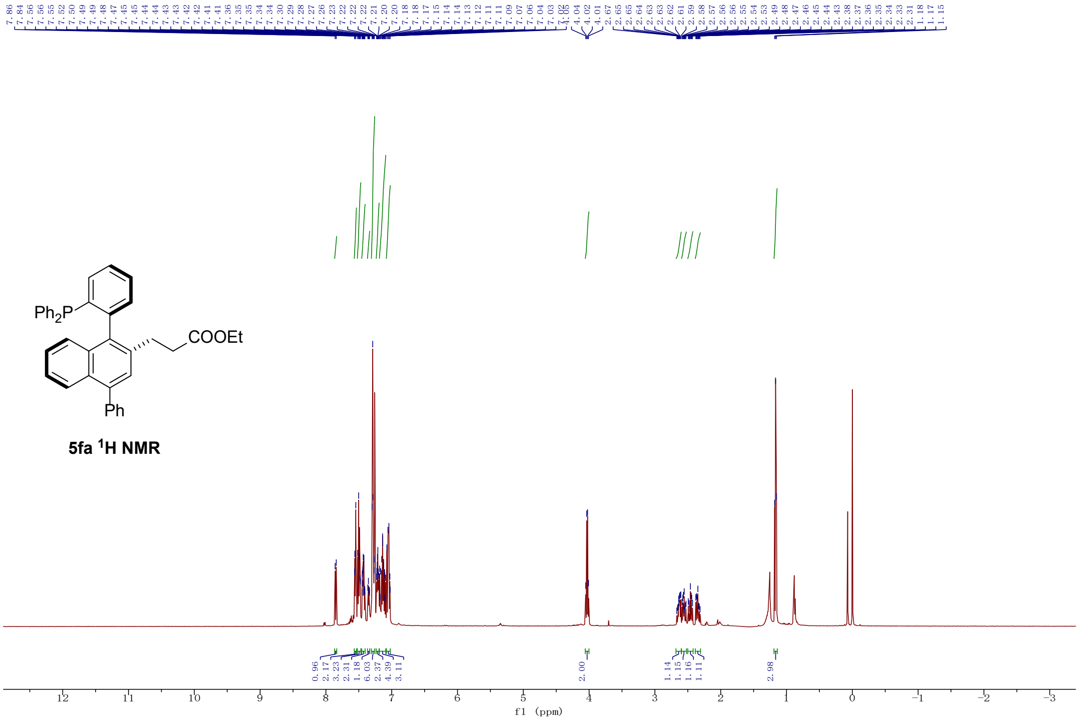

Supplementary Fig. 187 ^{1}H NMR spectra (500 MHz, CDCl$_3$, 25 ºC) of **5fa**

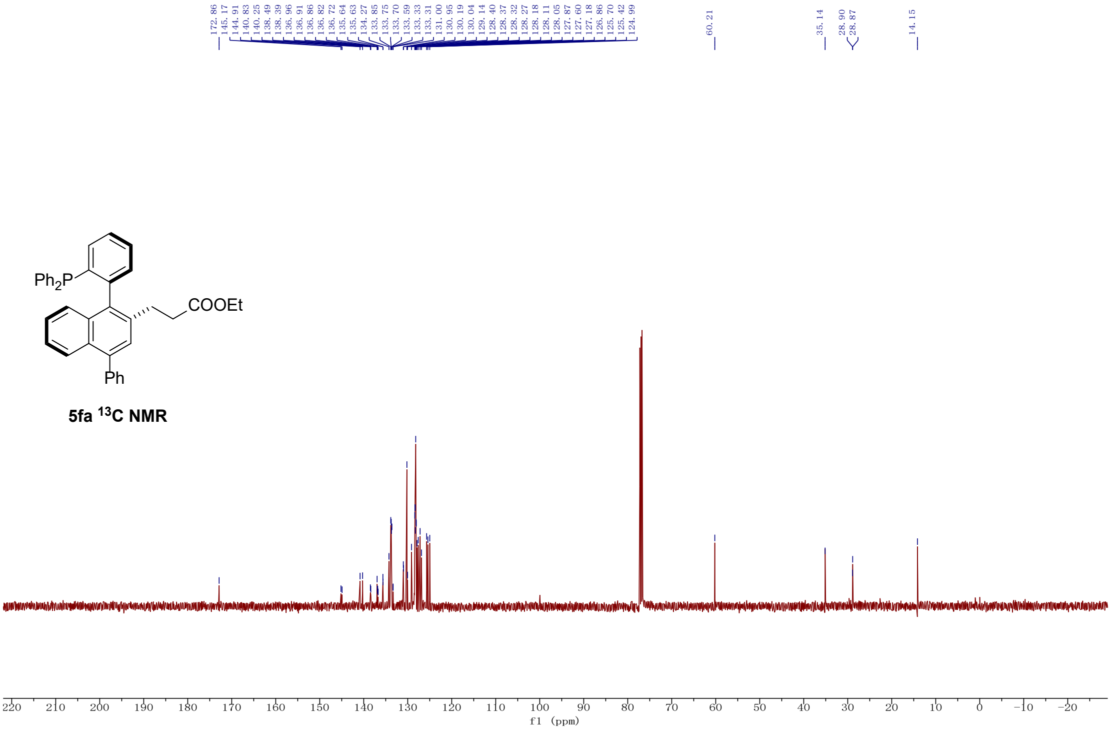

Supplementary Fig. 188. ^{13}C NMR spectra (126 MHz, CDCl$_3$, 25 ºC) of **5fa**

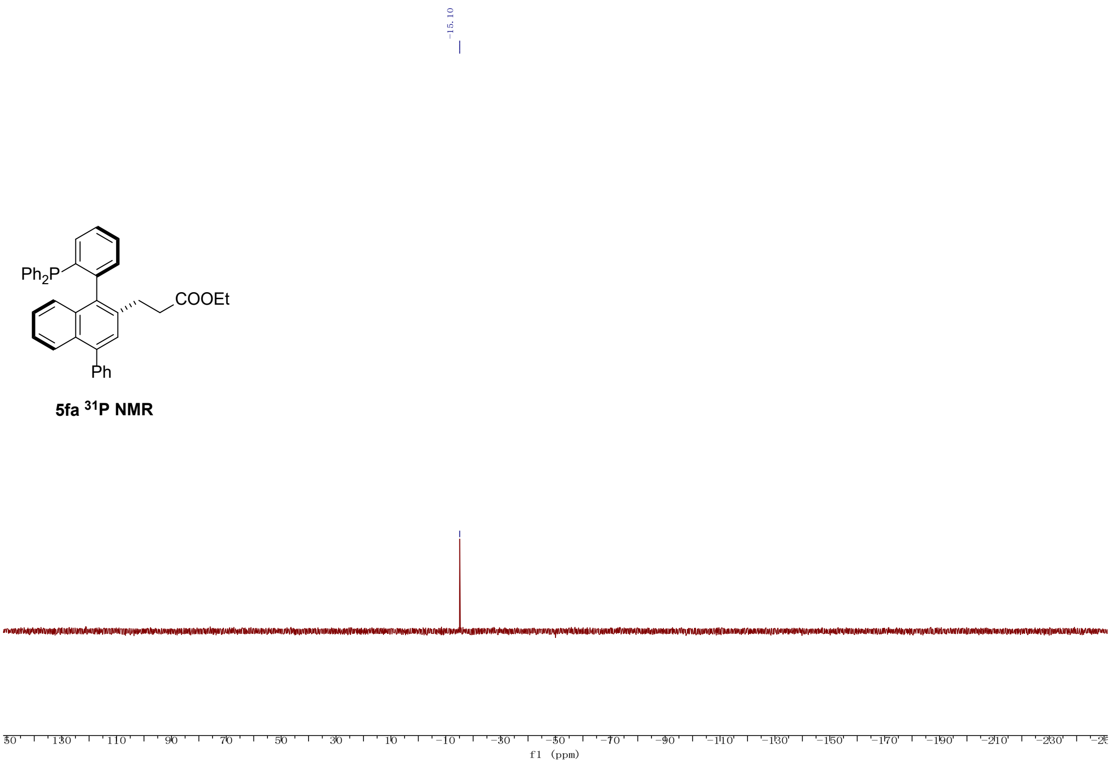

Supplementary Fig. 189. ^{31}P NMR spectra (202 MHz, CDCl$_3$, 25 °C) of **5fa**

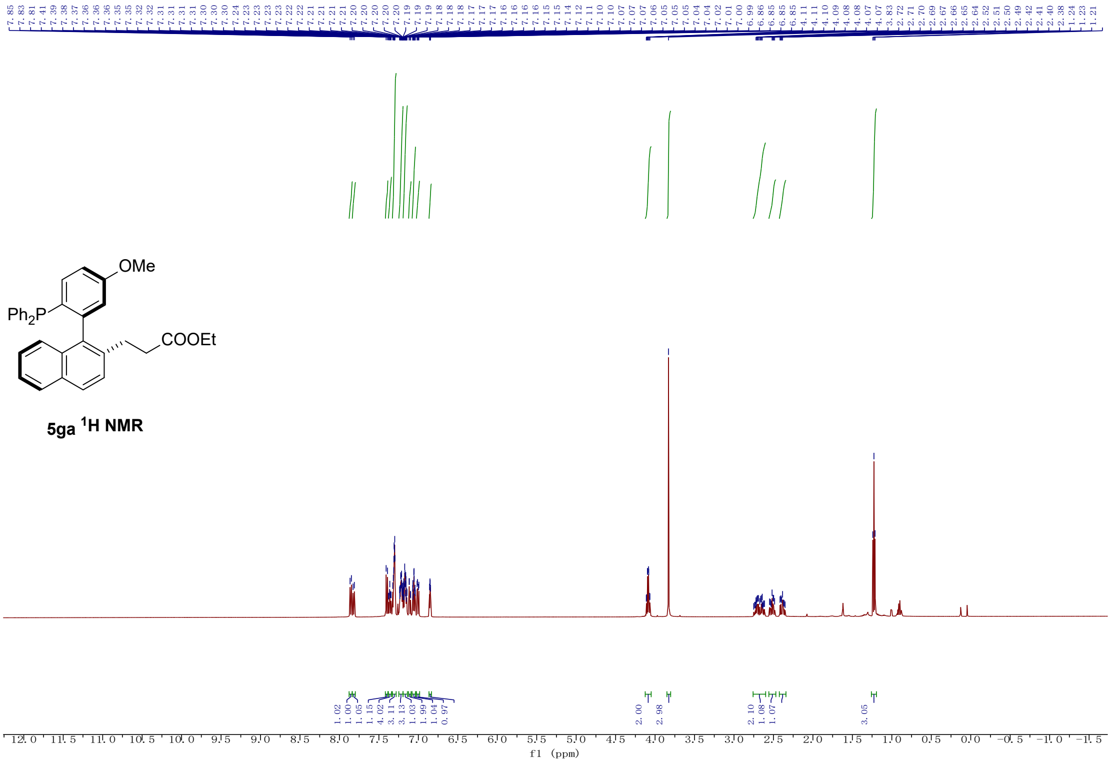

Supplementary Fig. 190 ^{1}H NMR spectra (500 MHz, CDCl$_3$, 25 °C) of **5ga**

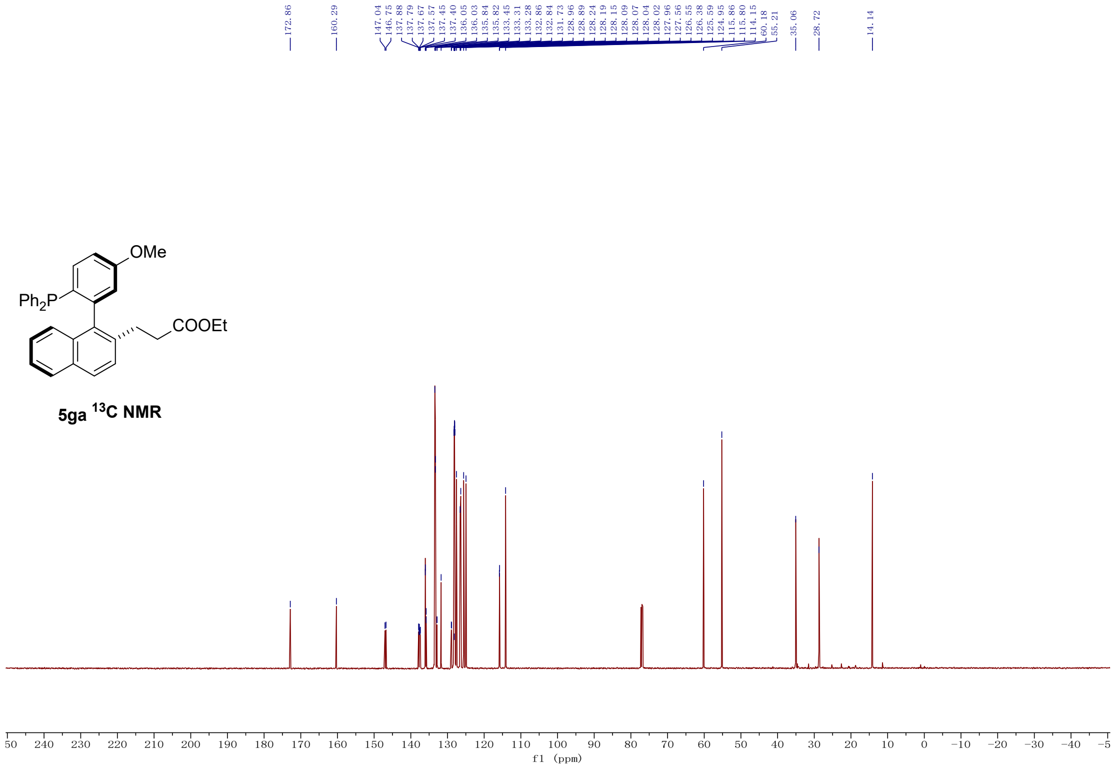

Supplementary Fig. 191. ^{13}C NMR spectra (126 MHz, $CDCl_3$, 25 °C) of **5ga**

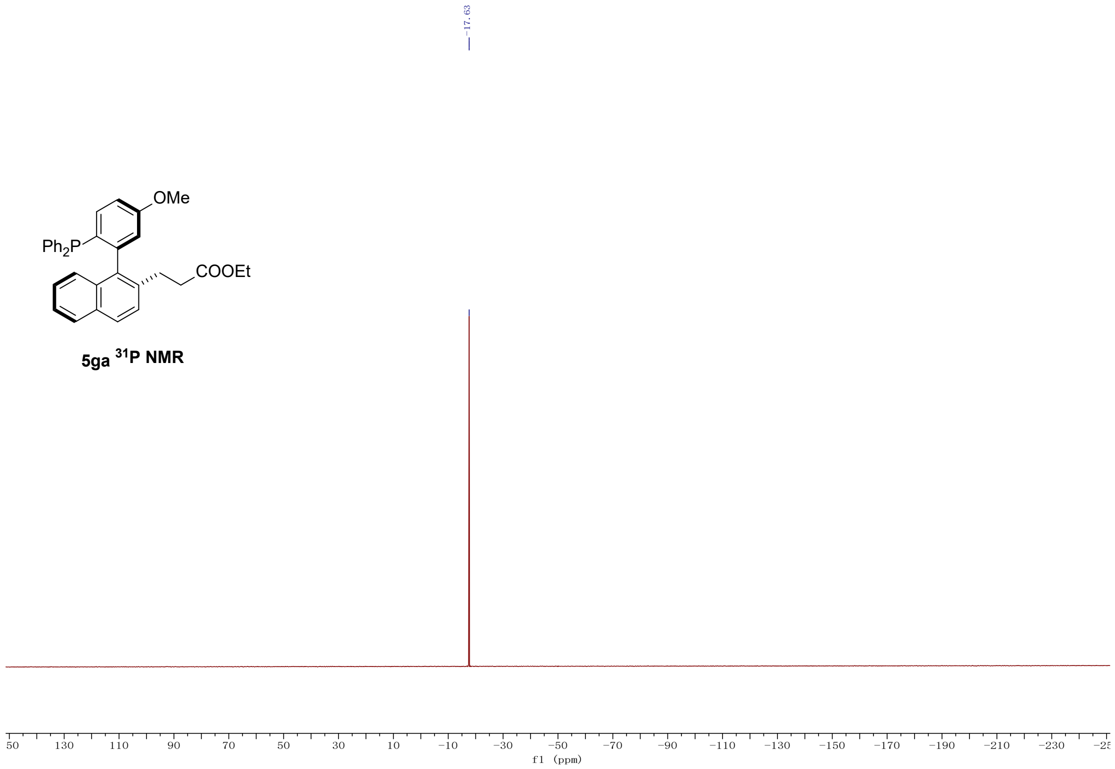

Supplementary Fig. 192. ^{31}P NMR spectra (202 MHz, $CDCl_3$, 25 °C) of **5ga**

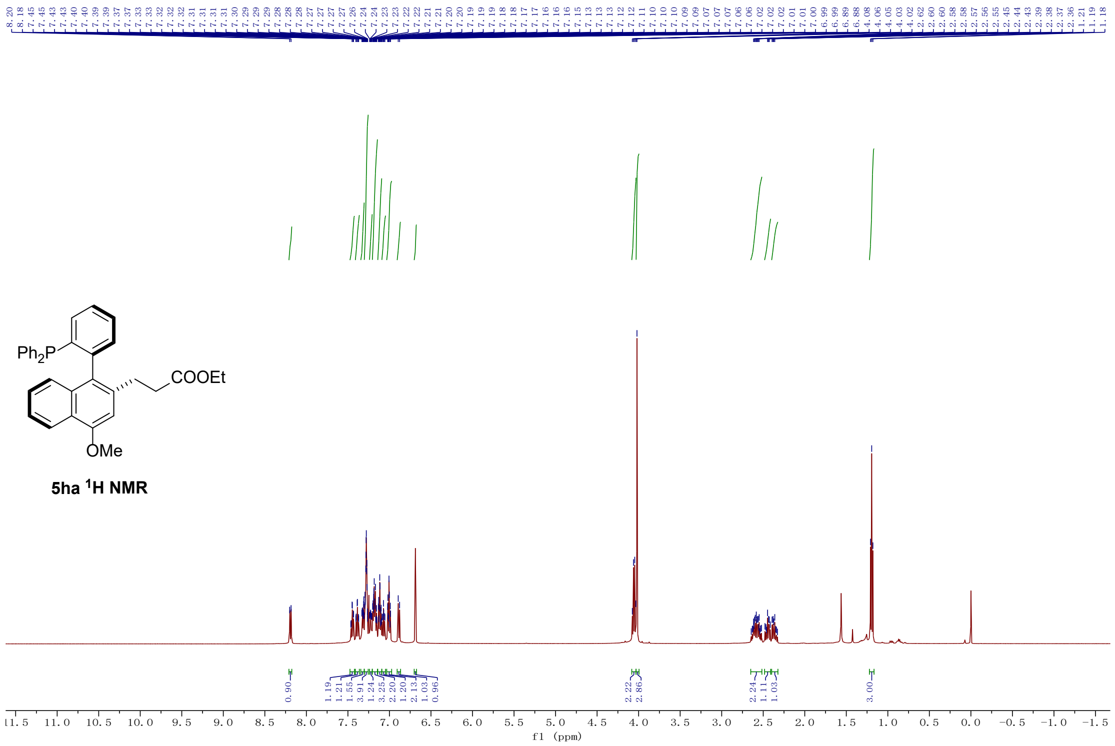

Supplementary Fig. 193 ^{1}H NMR spectra (500 MHz, $CDCl_3$, 25 °C) of **5ha**

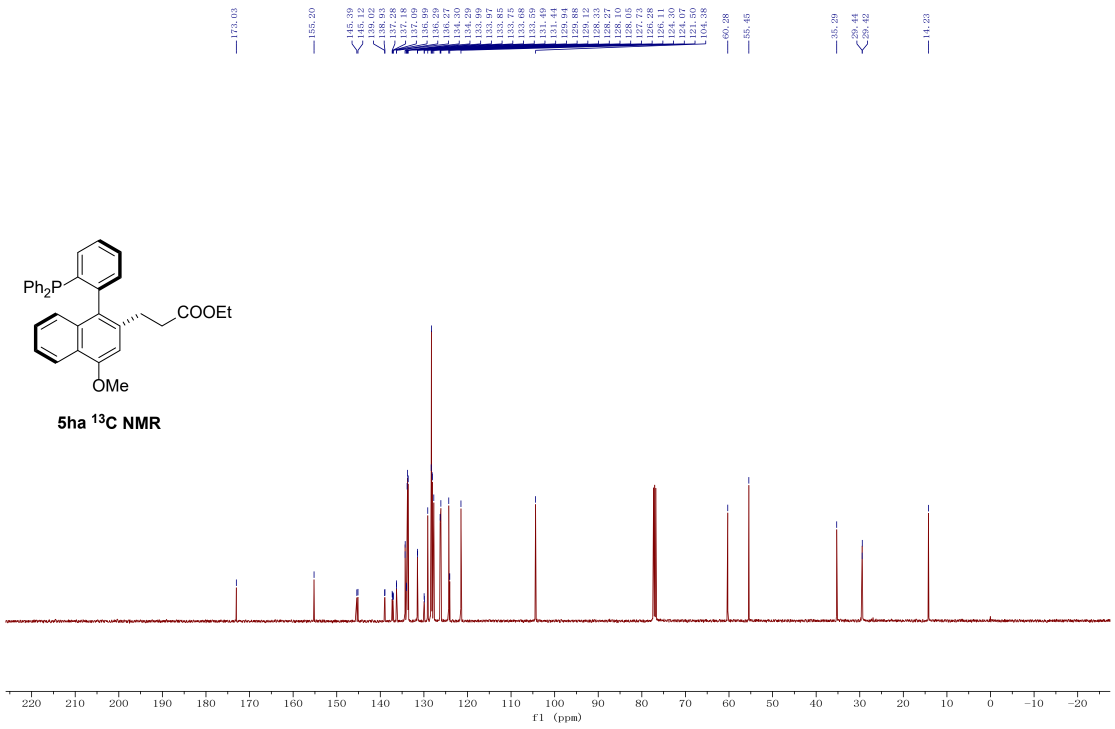

Supplementary Fig. 194. ^{13}C NMR spectra (126 MHz, $CDCl_3$, 25 °C) of **5ha**

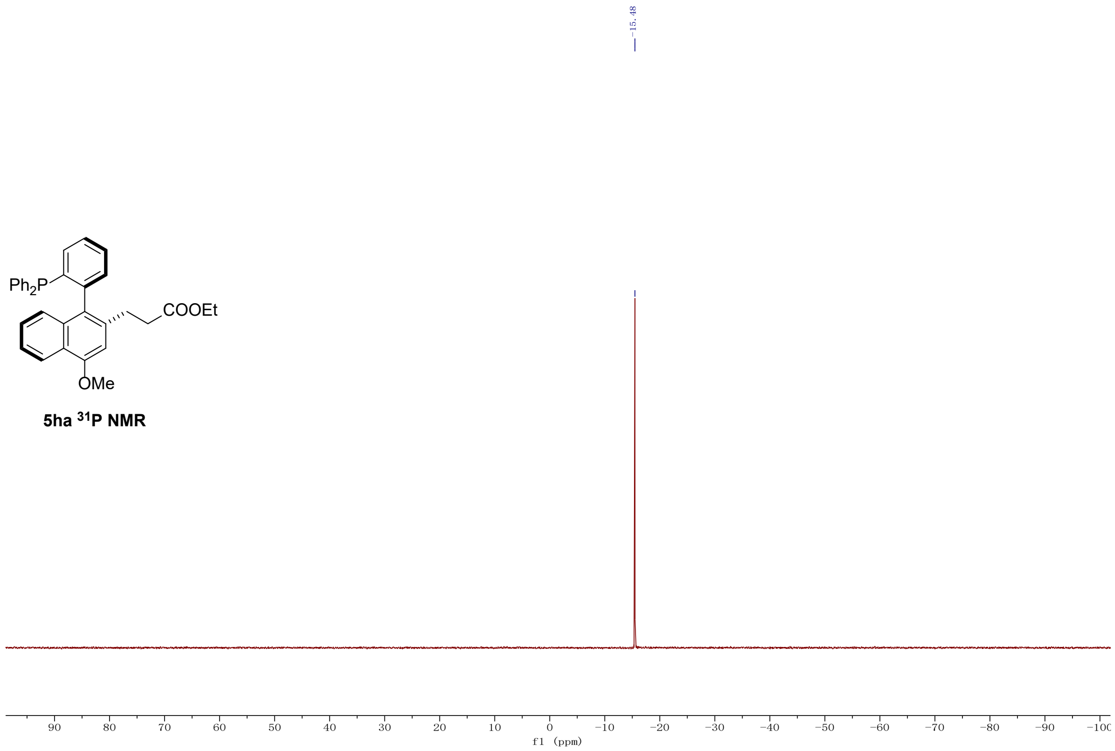

Supplementary Fig. 195. ^{31}P NMR spectra (202 MHz, CDCl$_3$, 25 °C) of **5ha**

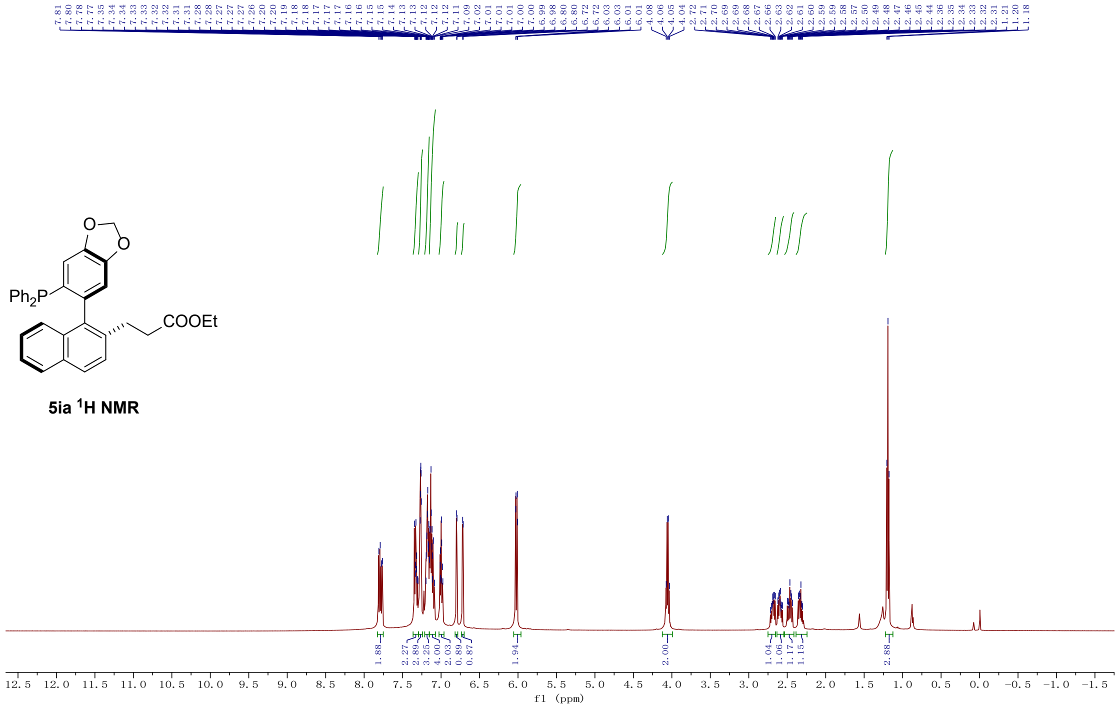

Supplementary Fig. 196 ^{1}H NMR spectra (500 MHz, CDCl$_3$, 25 °C) of **5ia**

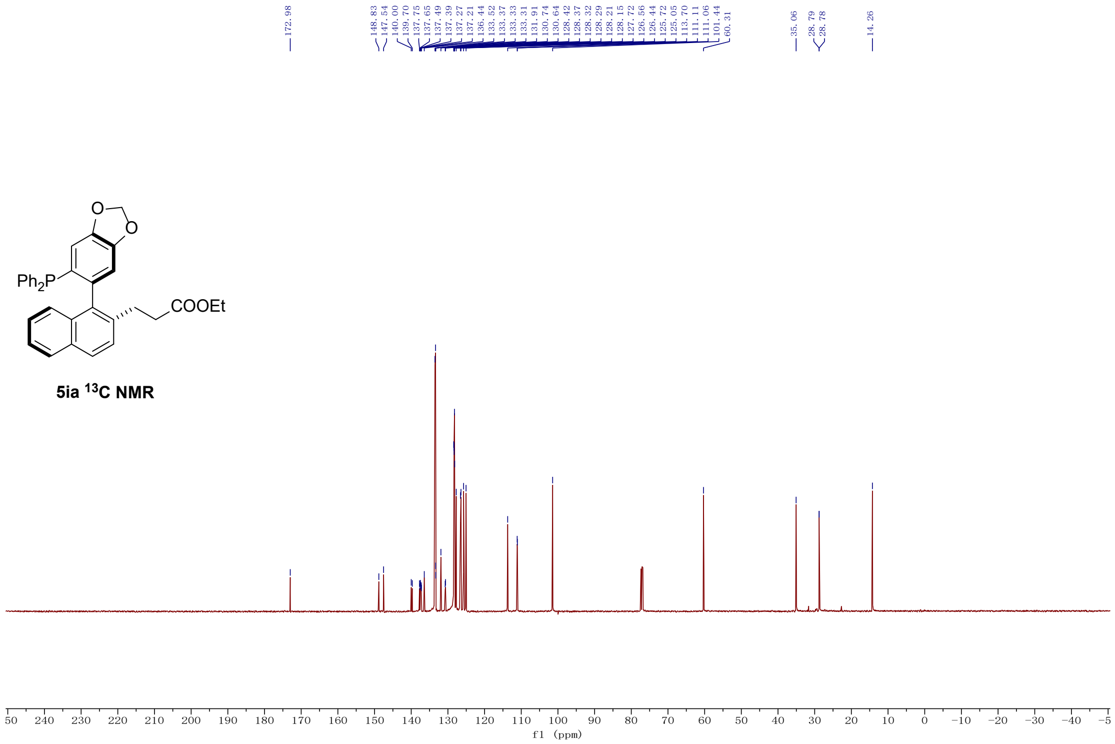

Supplementary Fig. 197. ^{13}C NMR spectra (126 MHz, $CDCl_3$, 25 °C) of **5ia**

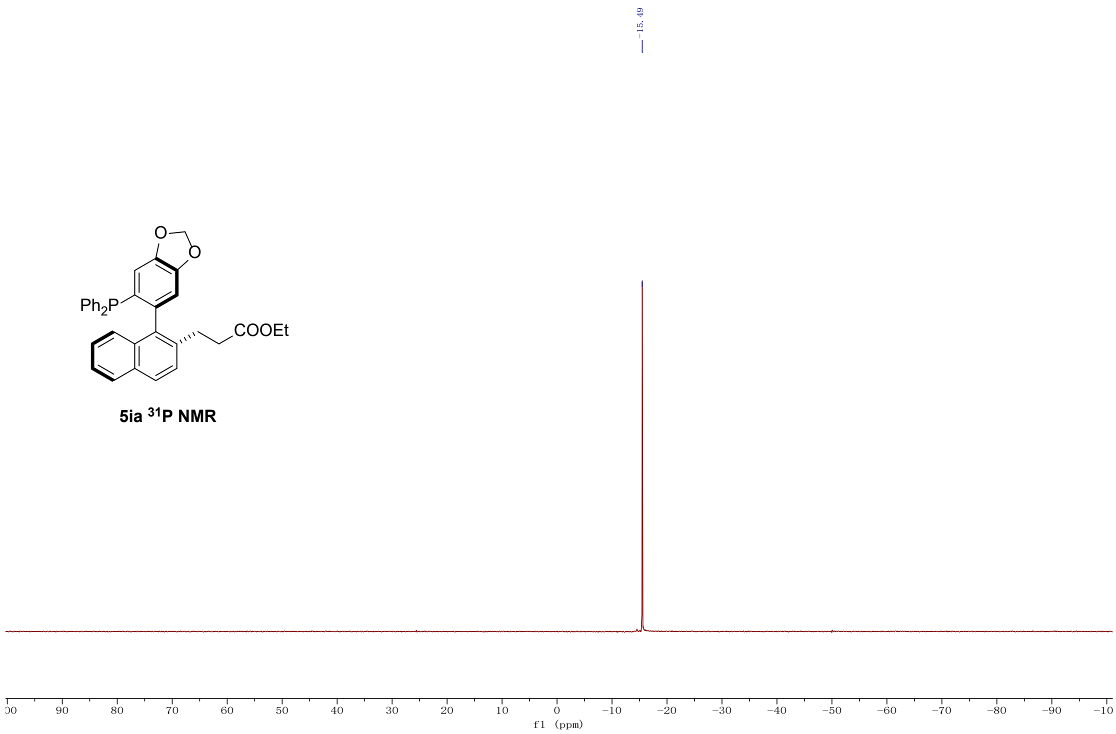

Supplementary Fig. 198. ^{31}P NMR spectra (202 MHz, $CDCl_3$, 25 °C) of **5ia**

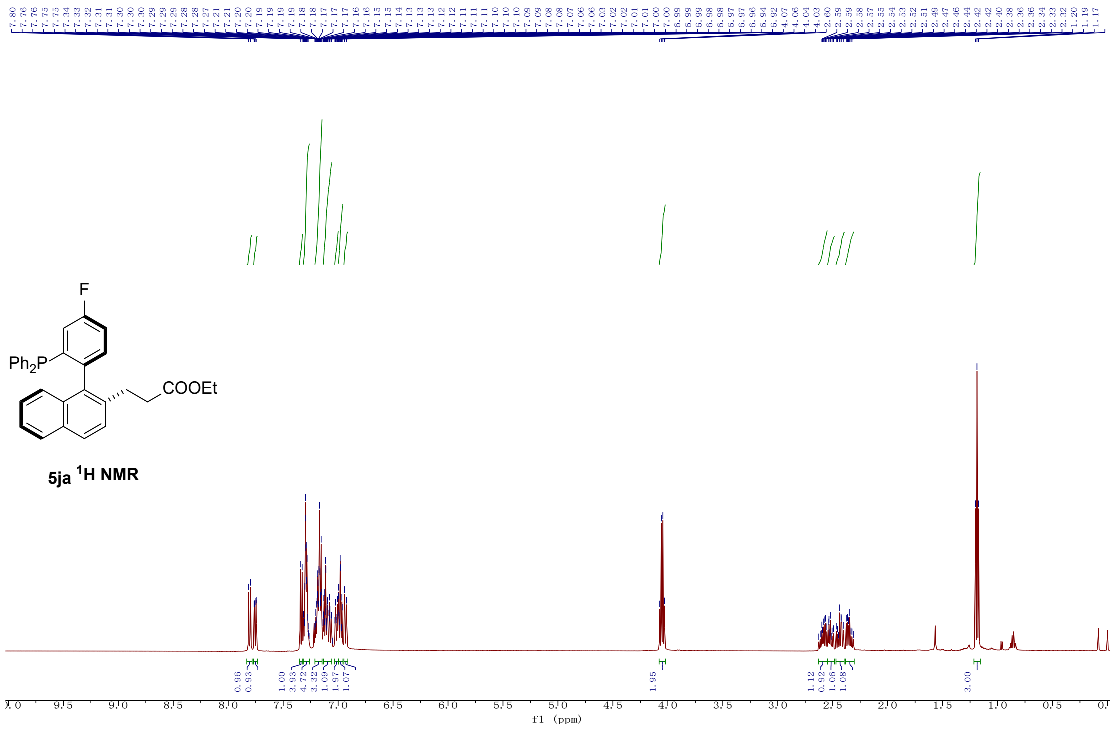

Supplementary Fig. 199 ^{1}H NMR spectra (500 MHz, $CDCl_3$, 25 ºC) of **5ja**

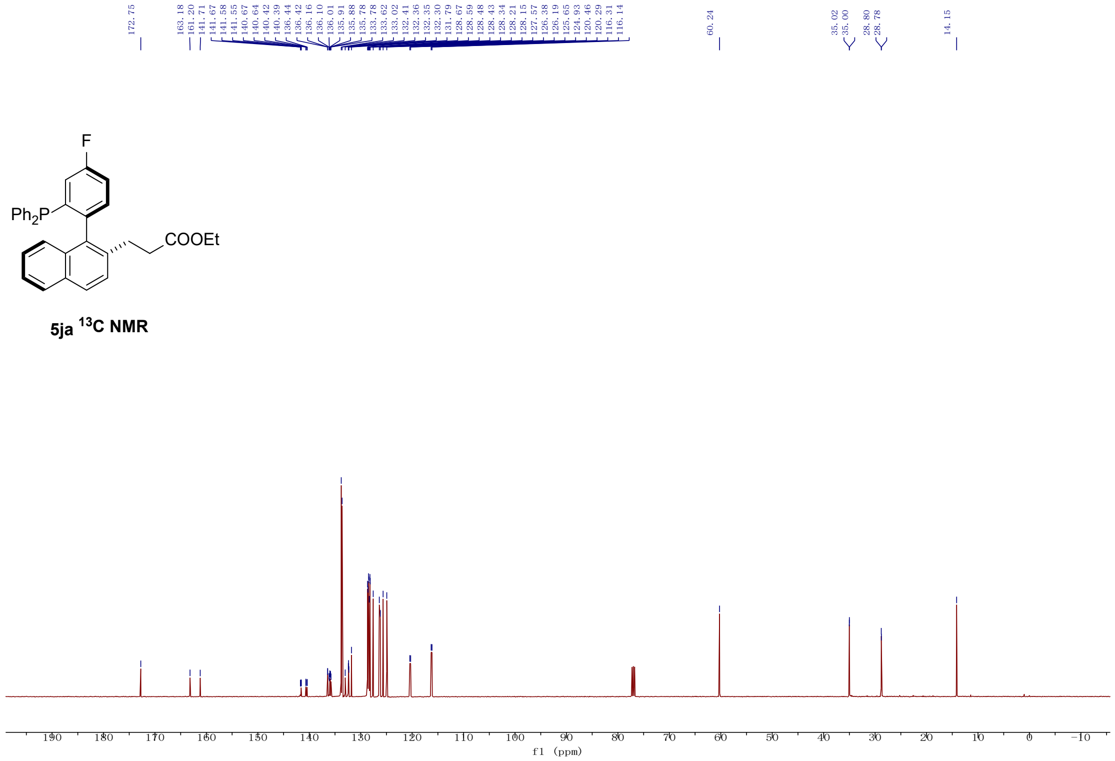

Supplementary Fig. 200. ^{13}C NMR spectra (126 MHz, $CDCl_3$, 25 ºC) of **5ja**

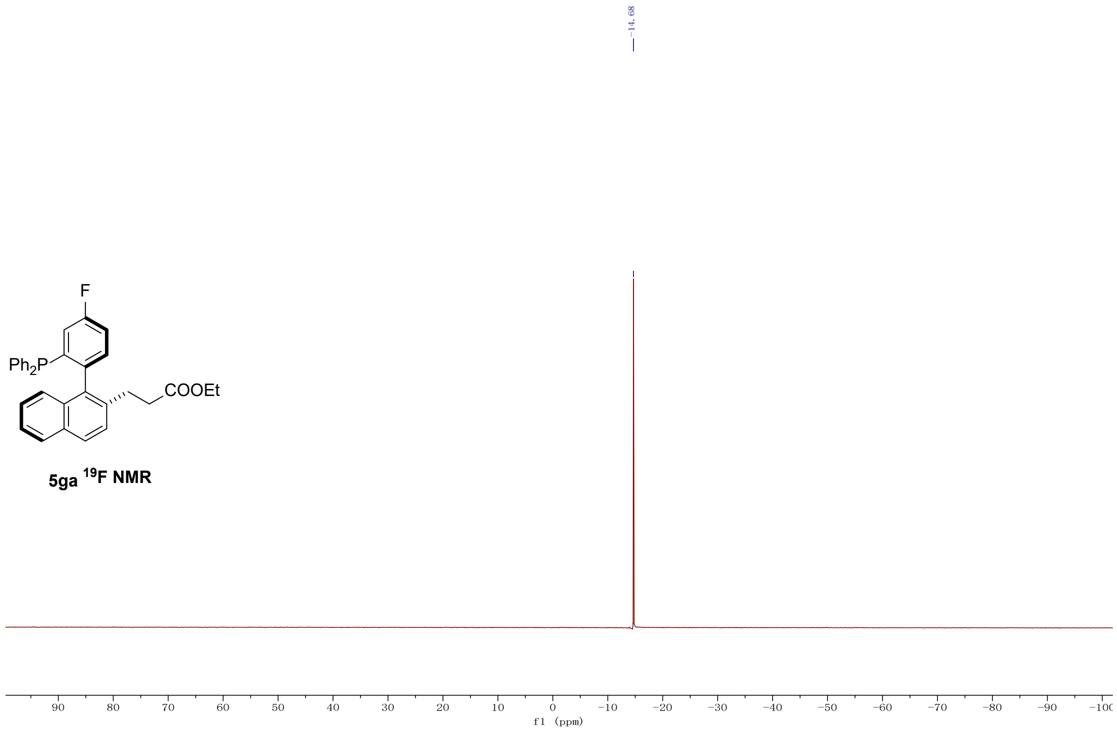

Supplementary Fig. 201. ^{31}P NMR spectra (202 MHz, $CDCl_3$, 25 ºC) of **5ja**

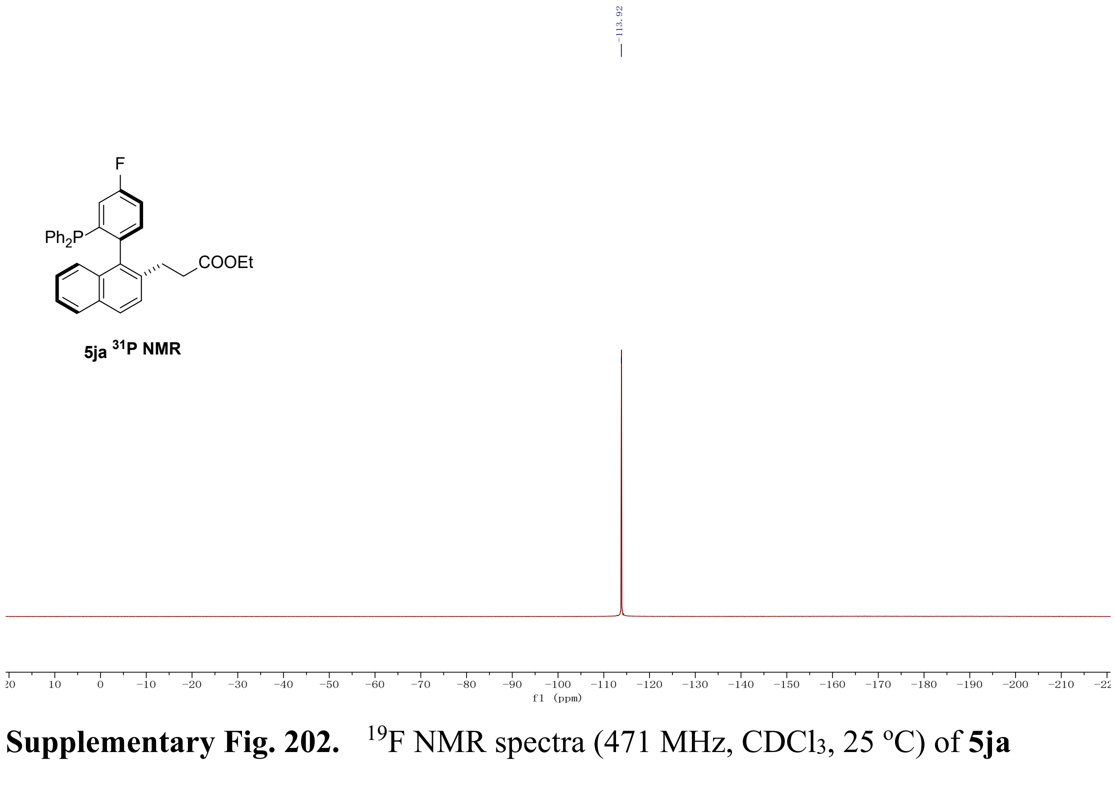

Supplementary Fig. 202. ^{19}F NMR spectra (471 MHz, $CDCl_3$, 25 ºC) of **5ja**

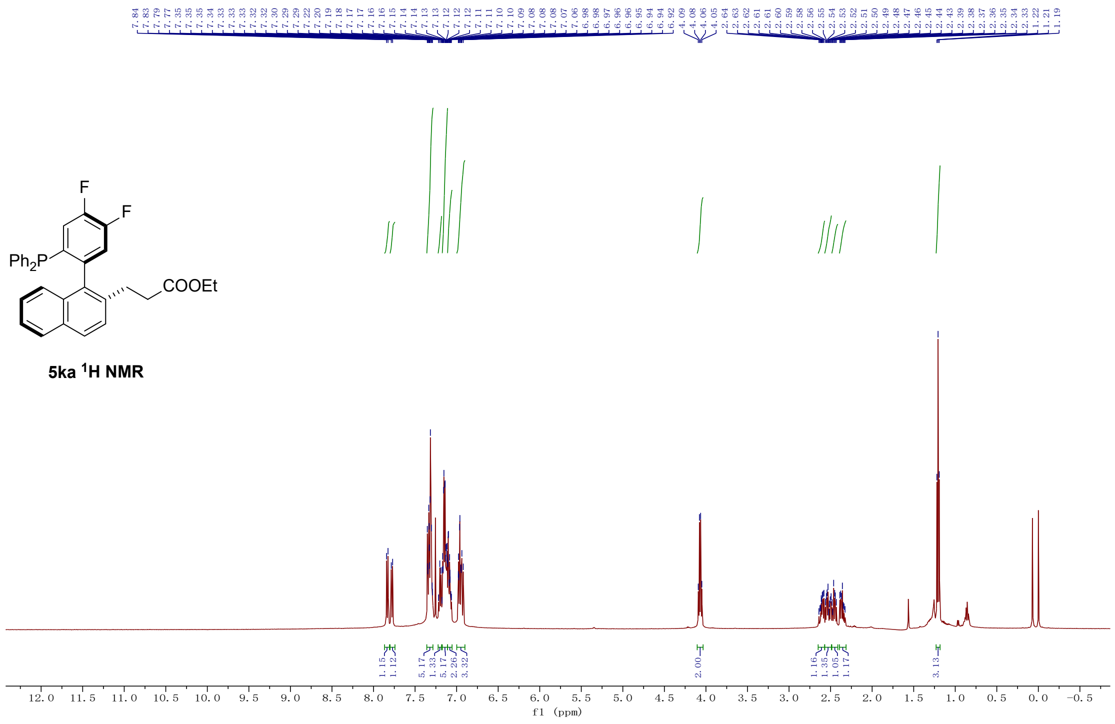

Supplementary Fig. 203 ^{1}H NMR spectra (500 MHz, $CDCl_3$, 25 ºC) of **5ka**

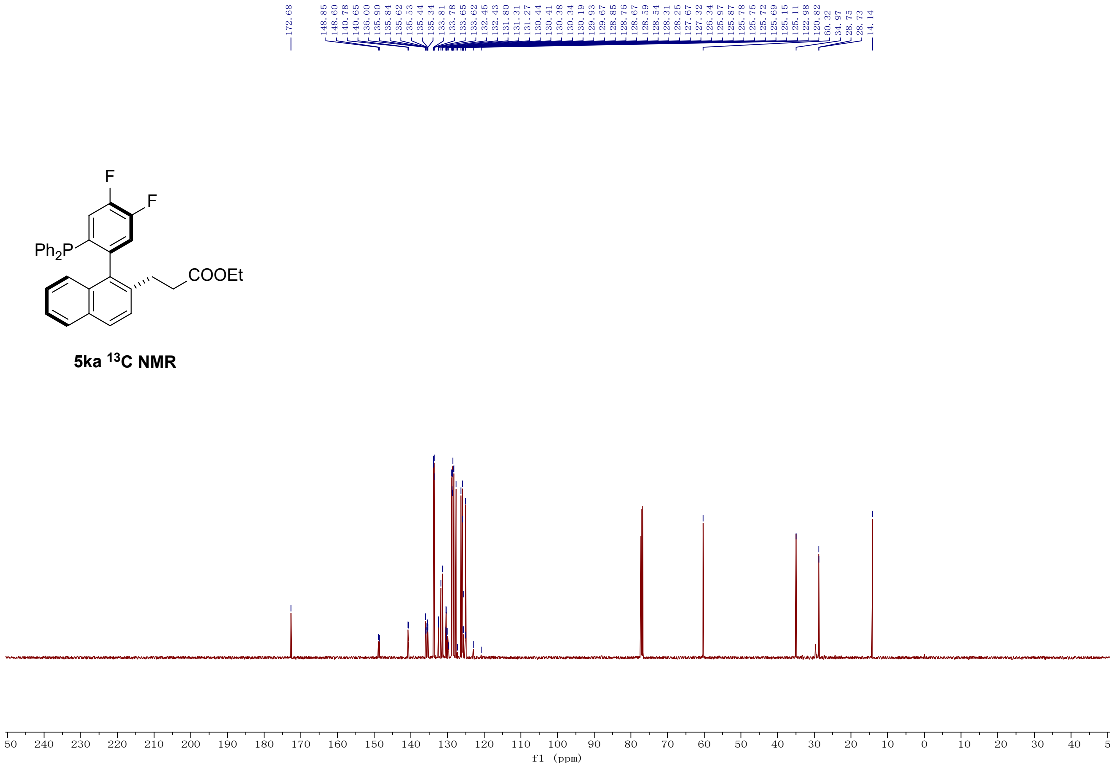

Supplementary Fig. 204. ^{13}C NMR spectra (126 MHz, $CDCl_3$, 25 ºC) of **5ka**

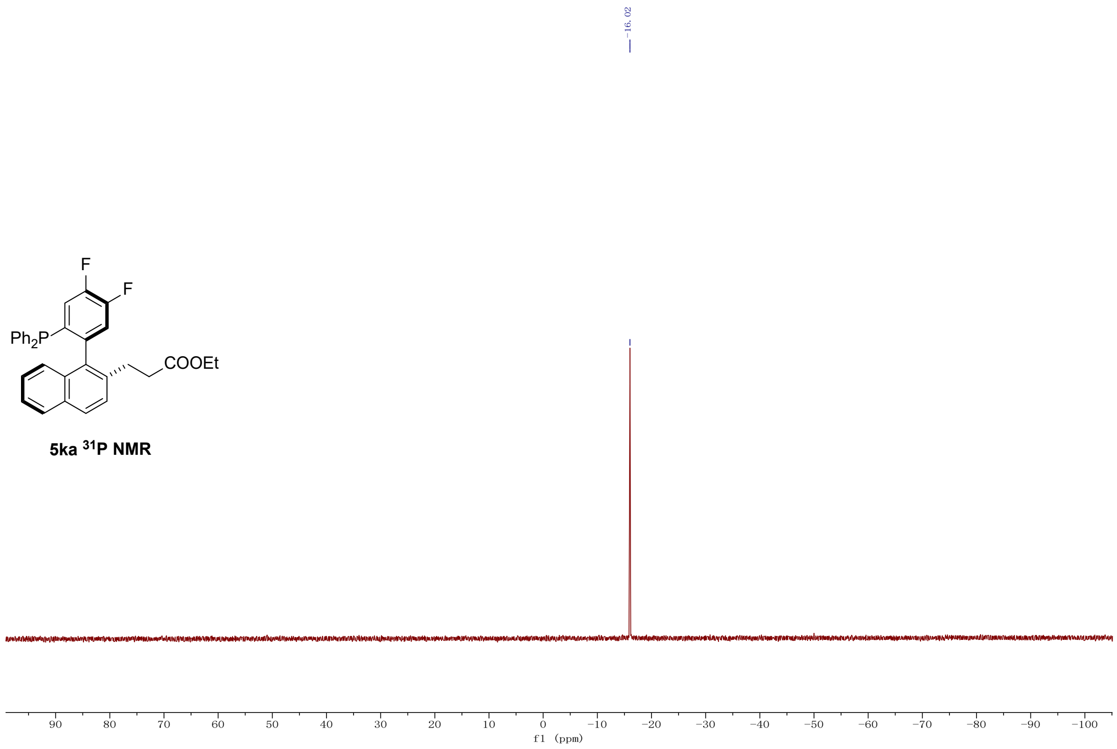

Supplementary Fig. 205. ^{31}P NMR spectra (202 MHz, $CDCl_3$, 25 °C) of **5ka**

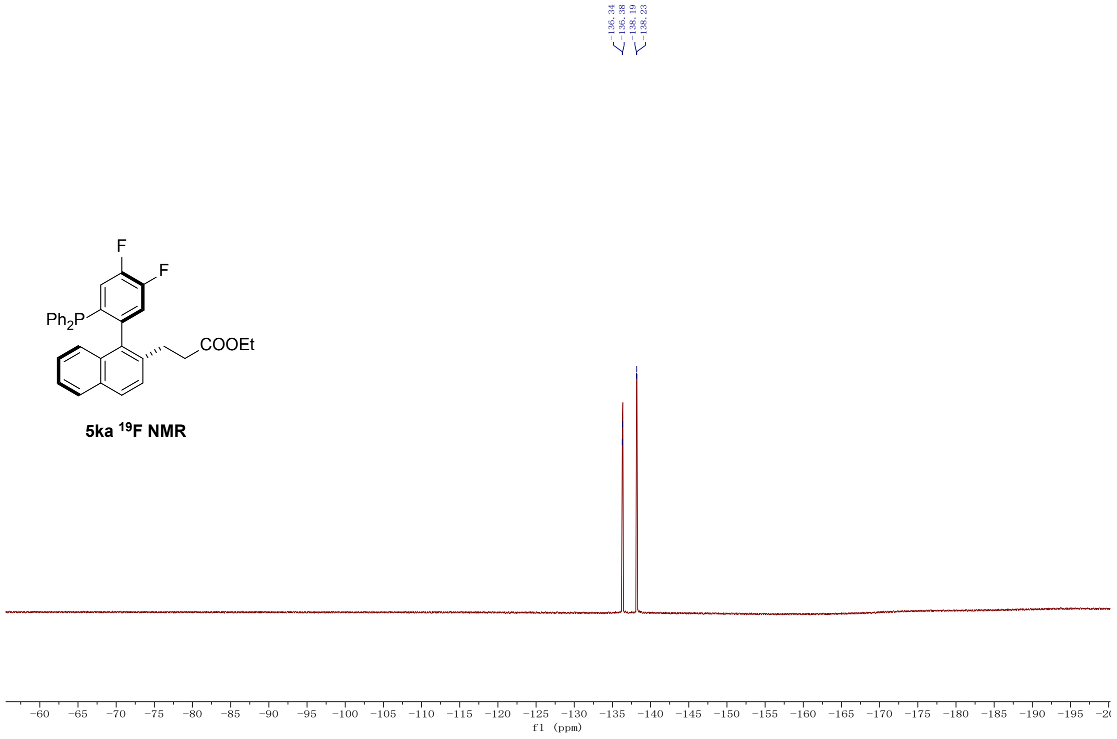

Supplementary Fig. 206. ^{19}F NMR spectra (471 MHz, $CDCl_3$, 25 °C) of **5ka**

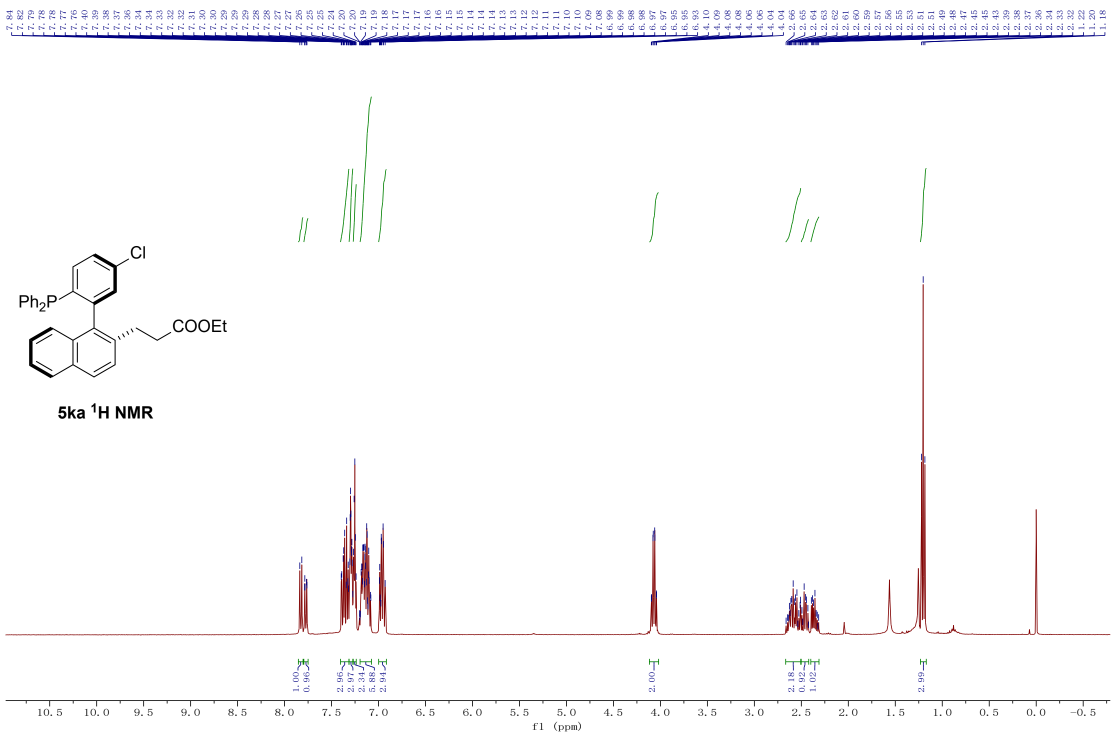

Supplementary Fig. 207 ^{1}H NMR spectra (500 MHz, CDCl$_3$, 25 ºC) of **5la**

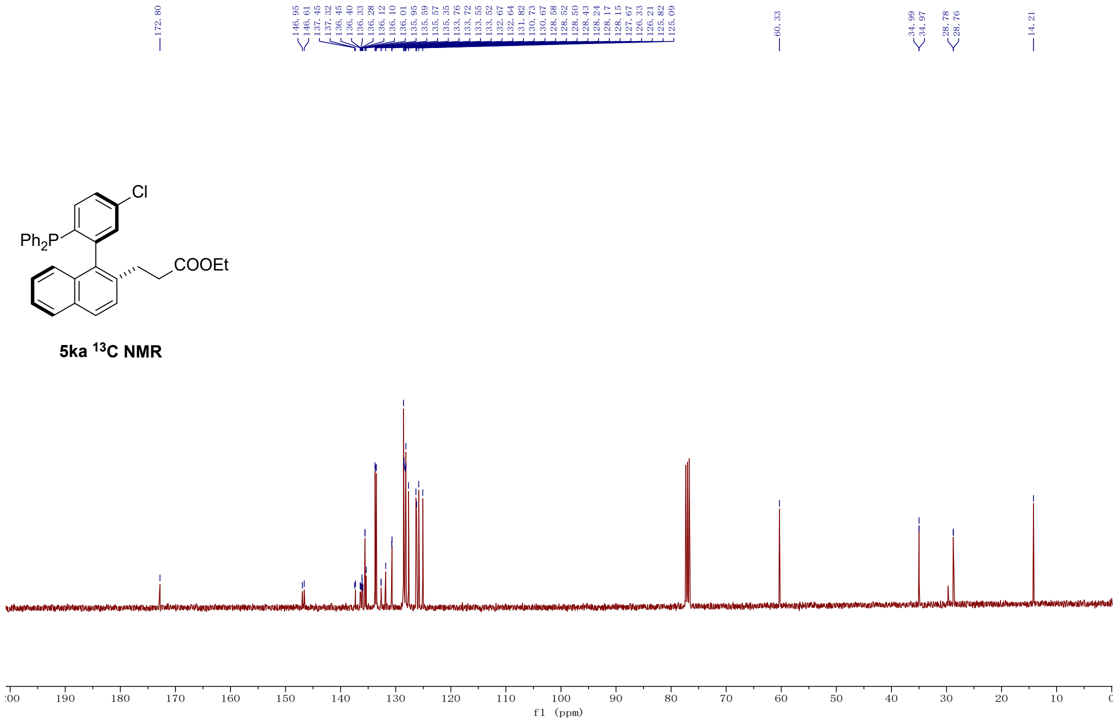

Supplementary Fig. 208. ^{13}C NMR spectra (126 MHz, CDCl$_3$, 25 ºC) of **5la**

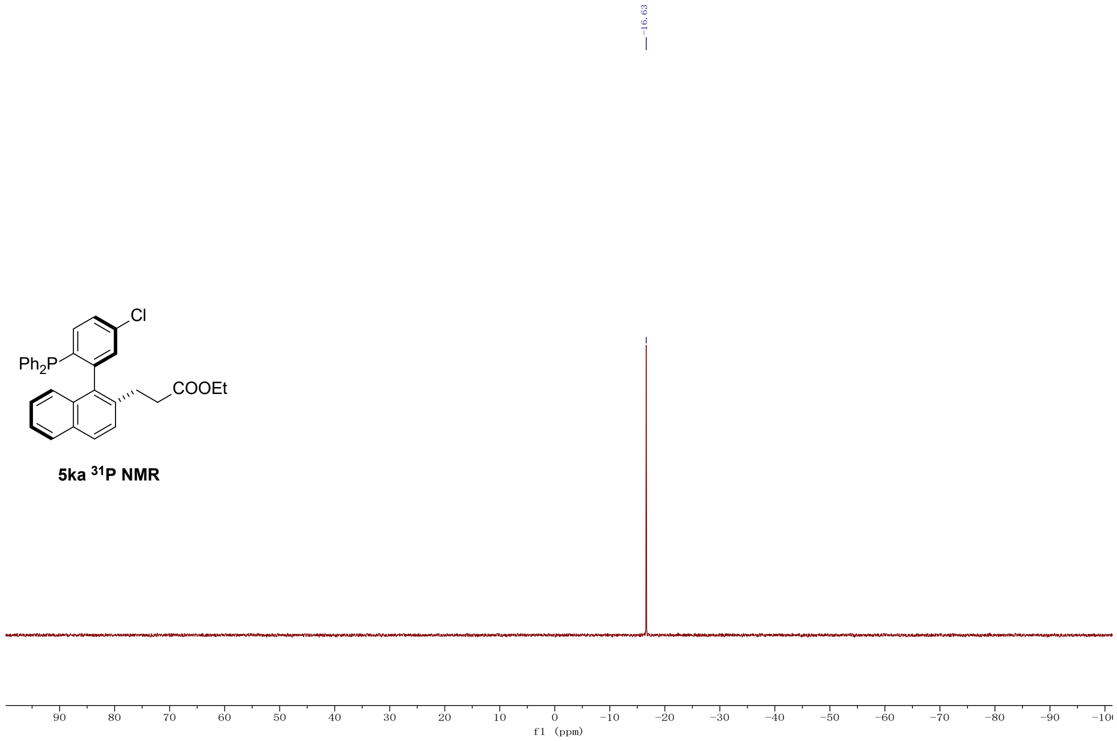

Supplementary Fig. 209. ^{31}P NMR spectra (202 MHz, $CDCl_3$, 25 ºC) of **5la**

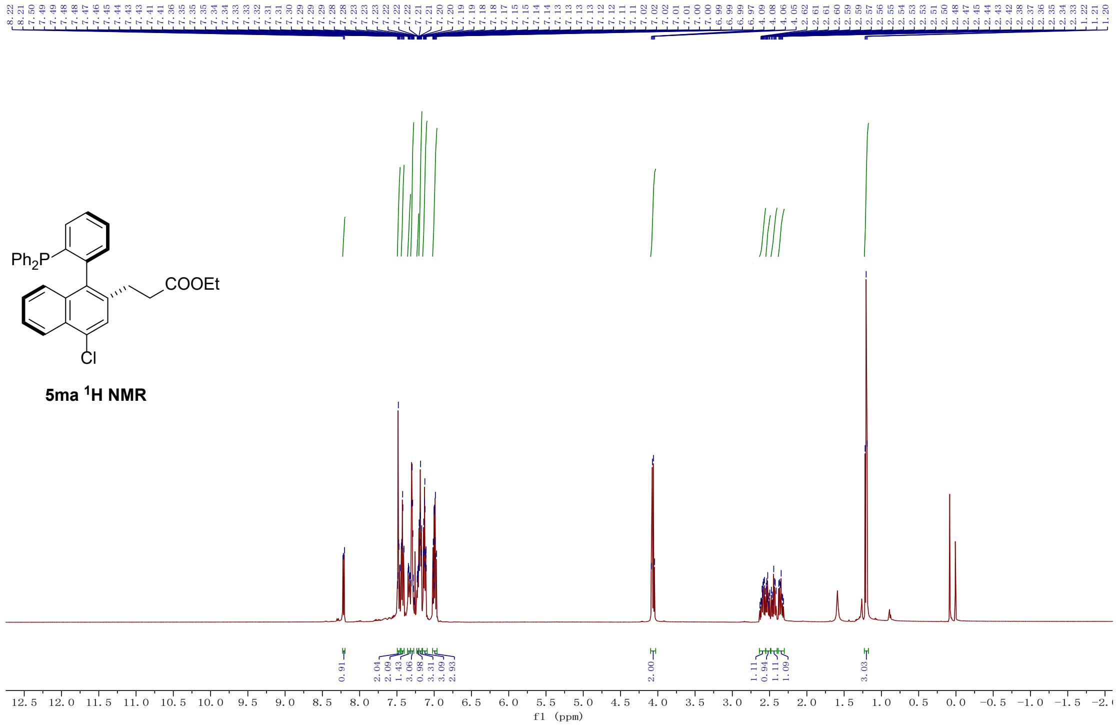

Supplementary Fig. 210 ^{1}H NMR spectra (500 MHz, $CDCl_3$, 25 ºC) of **5ma**

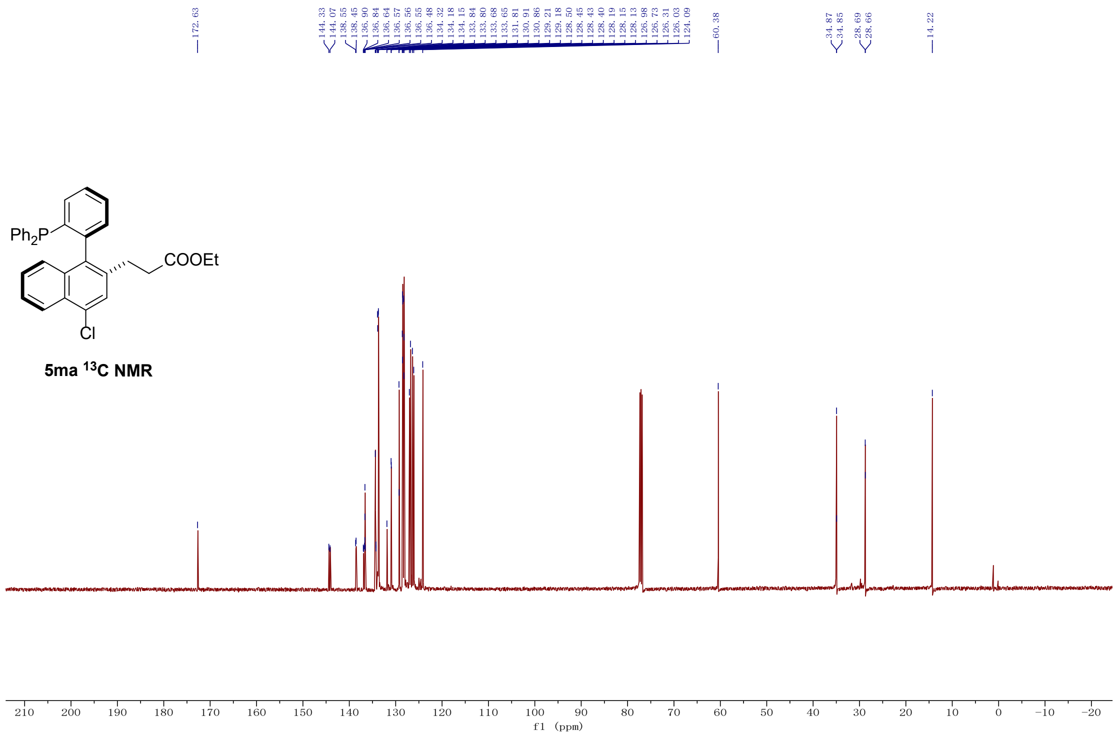

Supplementary Fig. 211. ^{13}C NMR spectra (126 MHz, $CDCl_3$, 25 °C) of **5ma**

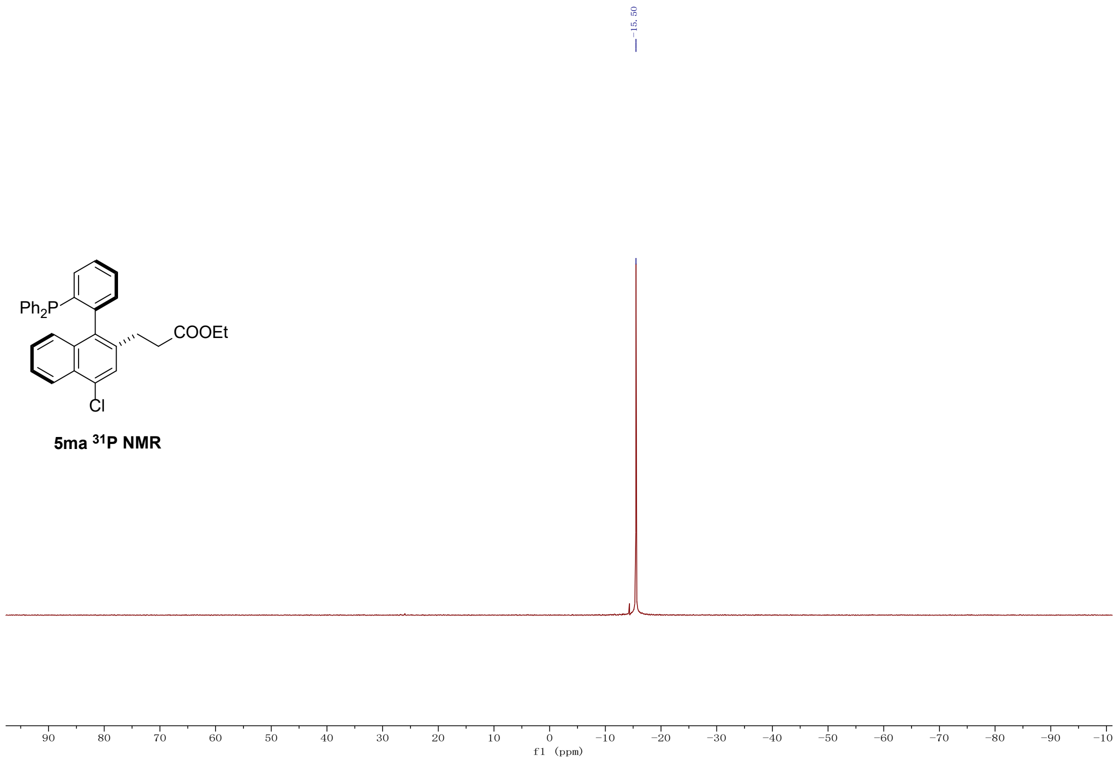

Supplementary Fig. 212. ^{31}P NMR spectra (202 MHz, $CDCl_3$, 25 °C) of **5ma**

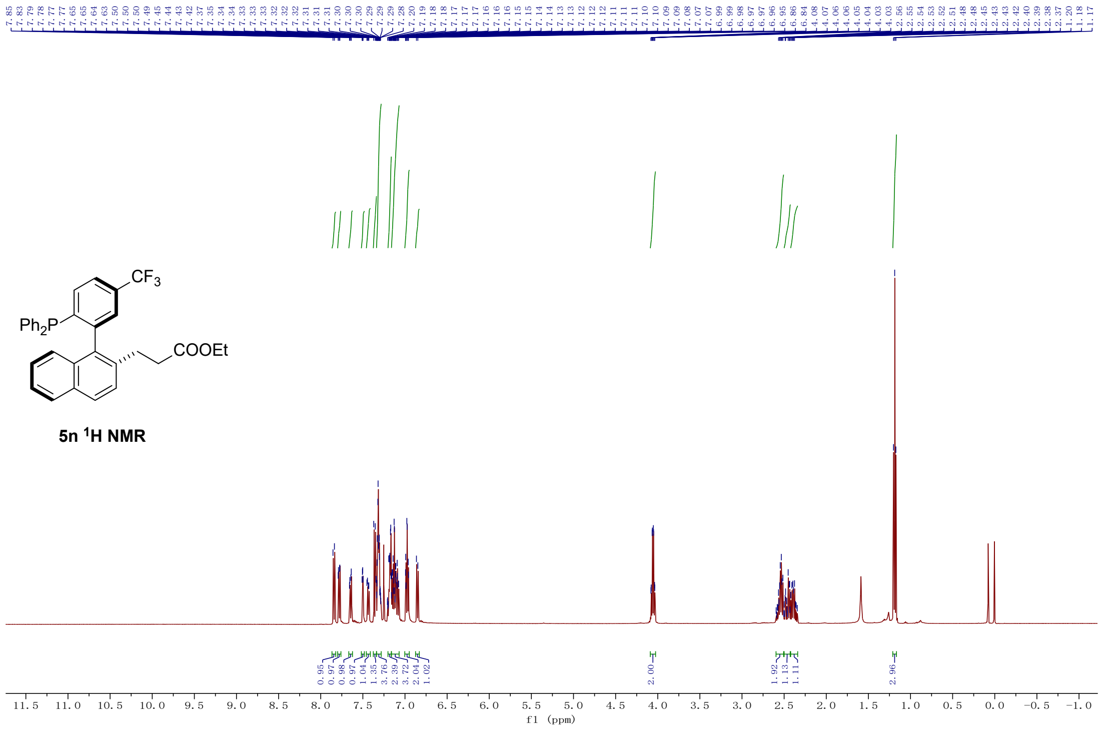

Supplementary Fig. 213 ^{1}H NMR spectra (500 MHz, $CDCl_3$, 25 °C) of **5na**

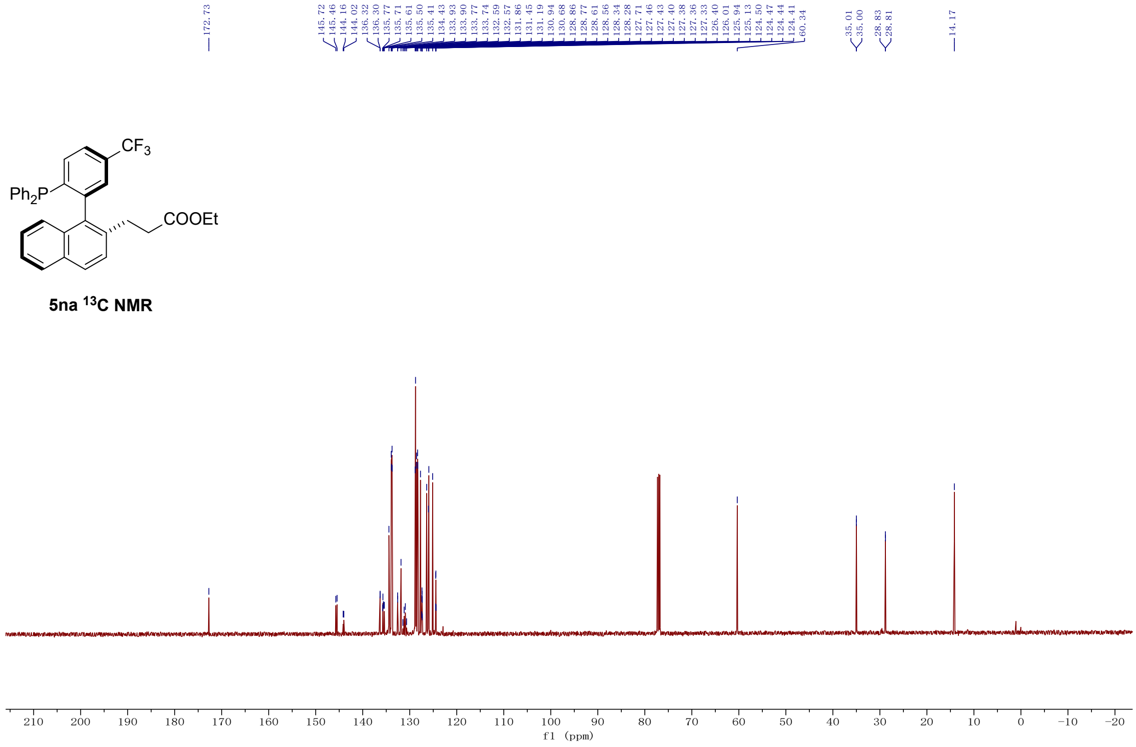

Supplementary Fig. 214. ^{13}C NMR spectra (126 MHz, $CDCl_3$, 25 °C) of **5na**

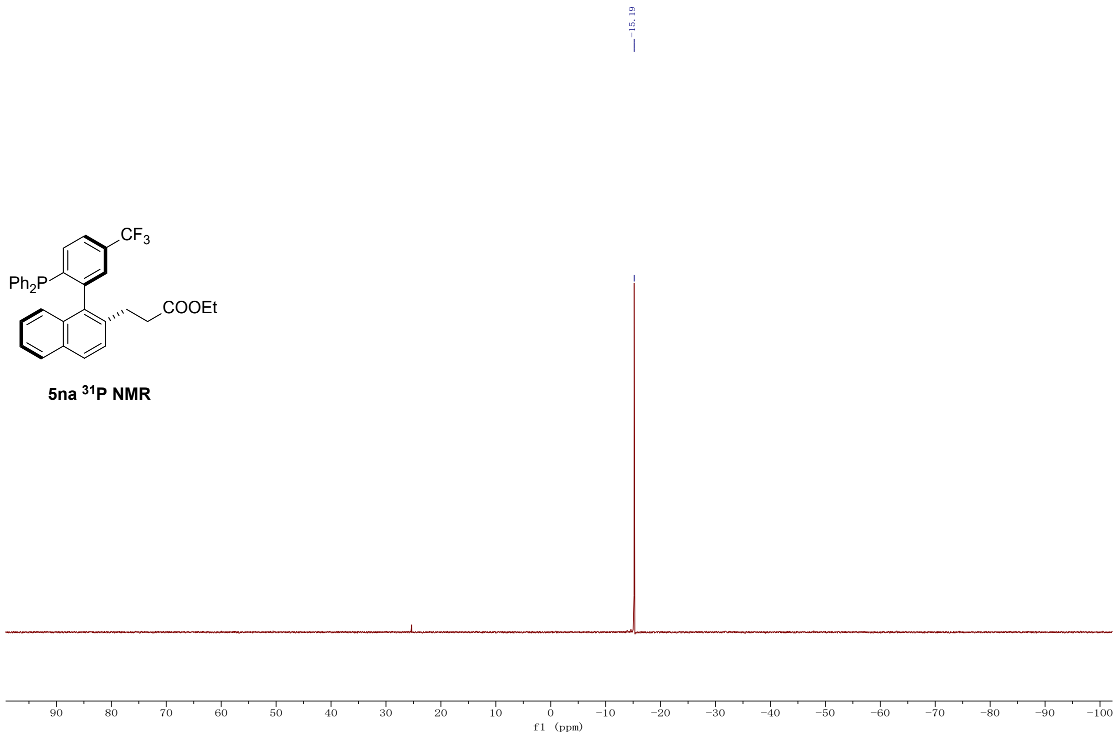

Supplementary Fig. 215. ^{31}P NMR spectra (202 MHz, $CDCl_3$, 25 °C) of **5na**

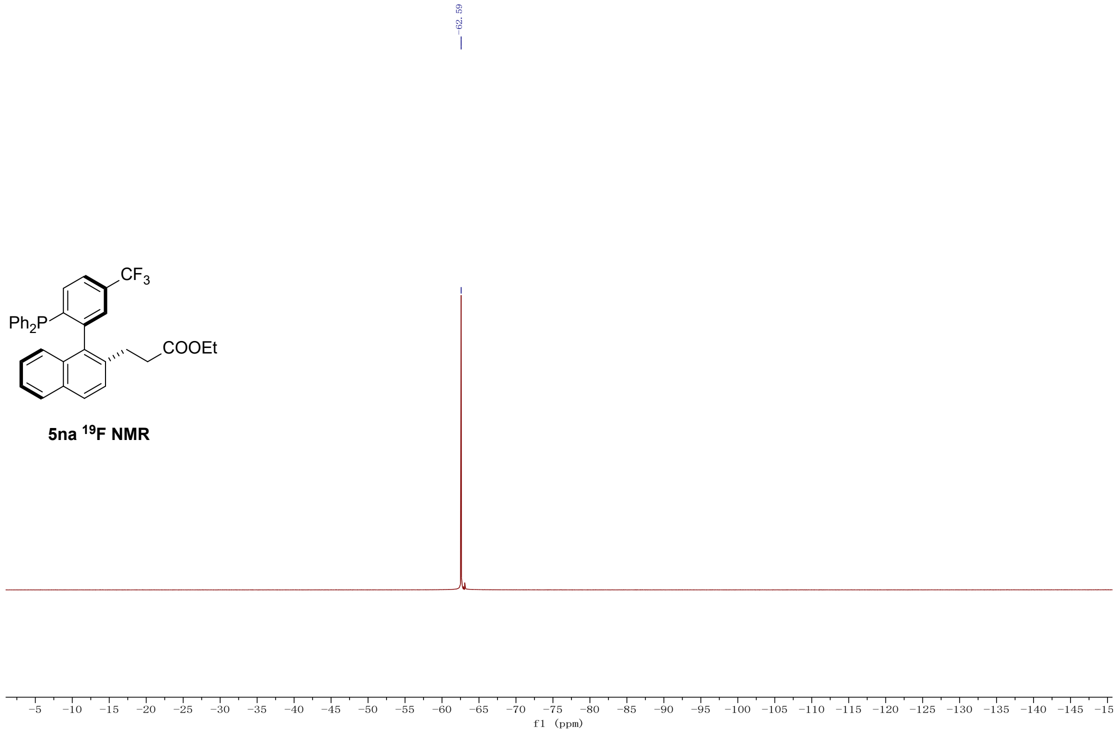

Supplementary Fig. 216 ^{19}F NMR spectra (471 MHz, $CDCl_3$, 25 °C) of **5na**

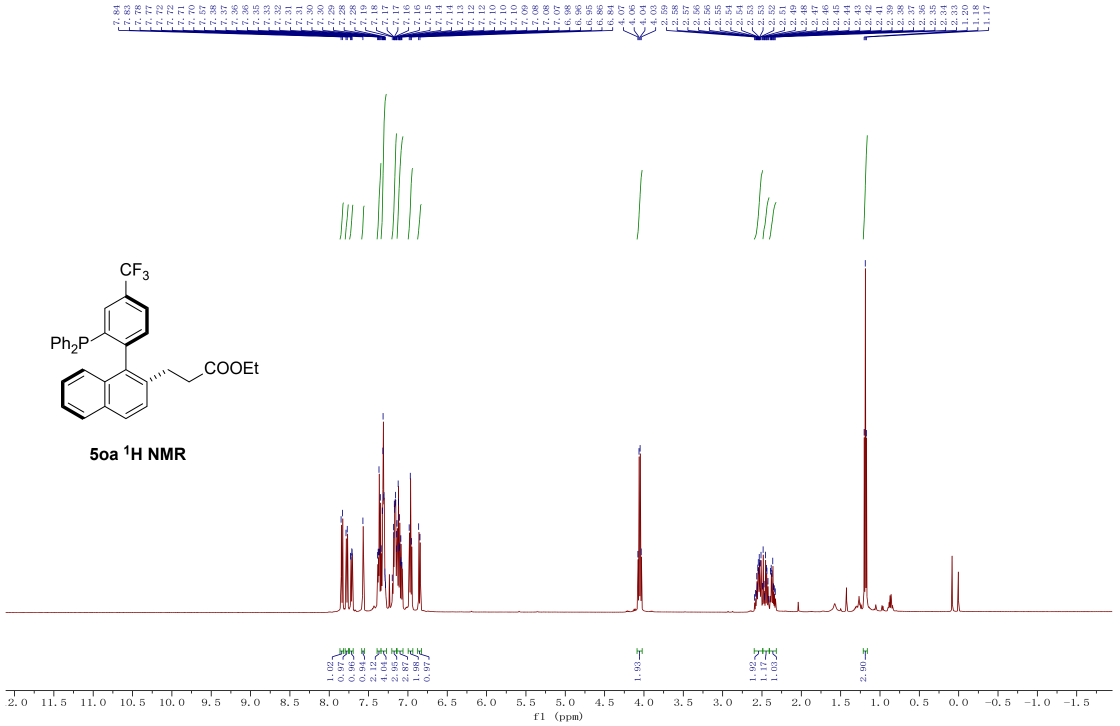

Supplementary Fig. 217 ^{1}H NMR spectra (500 MHz, $CDCl_3$, 25 ºC) of **5oa**

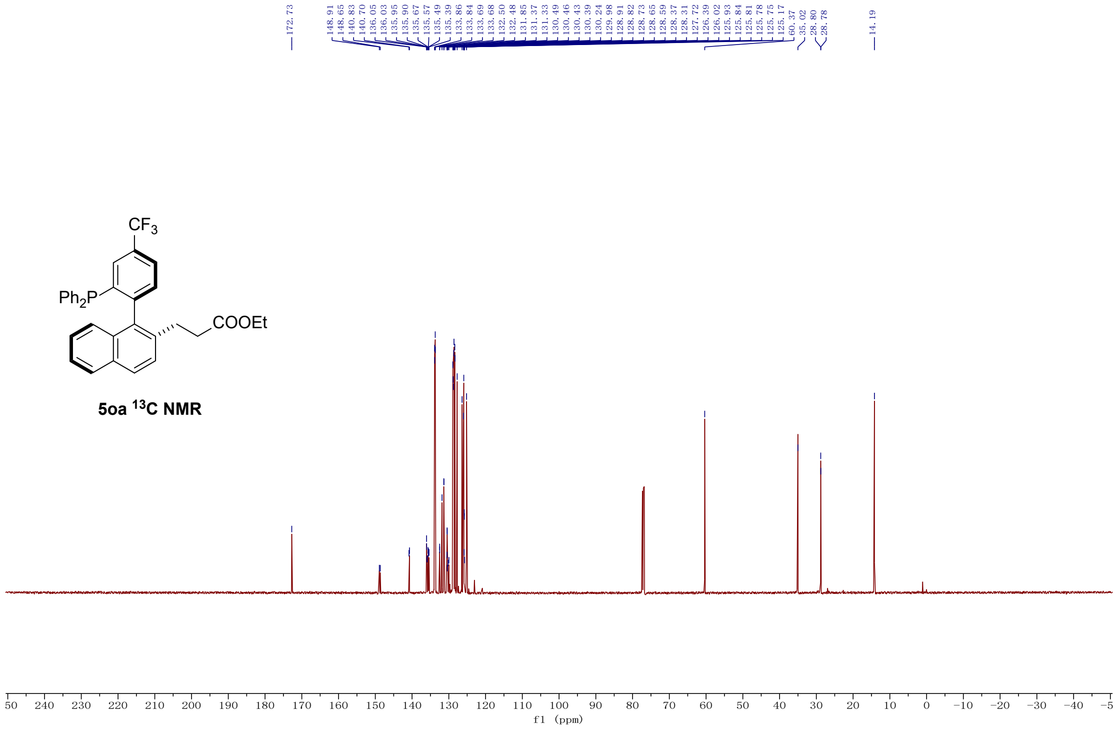

Supplementary Fig. 218. ^{13}C NMR spectra (126 MHz, $CDCl_3$, 25 ºC) of **5oa**

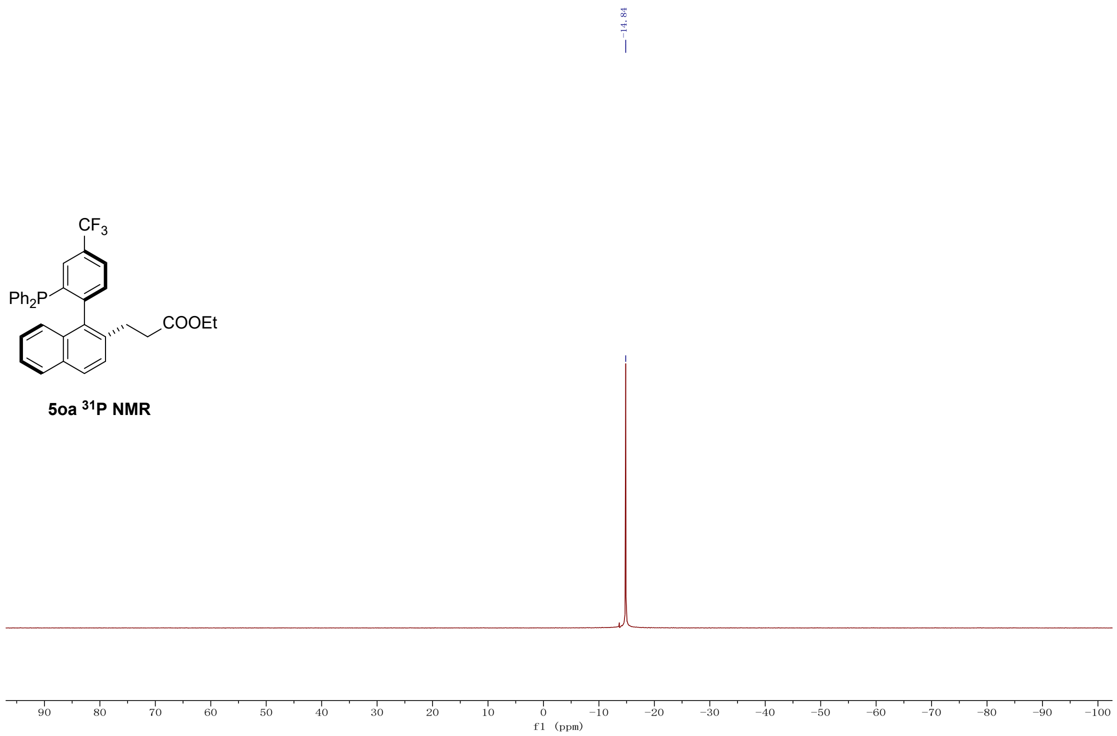

Supplementary Fig. 219 ^{31}P NMR spectra (202 MHz, $CDCl_3$, 25 ºC) of **5oa**

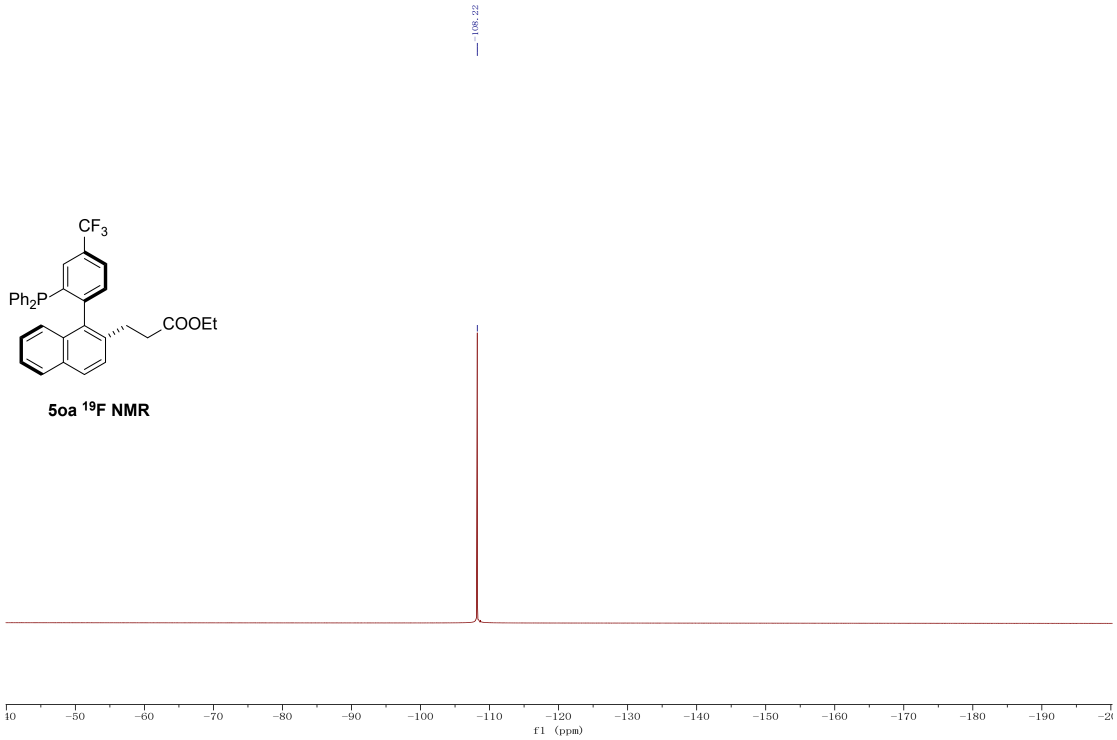

Supplementary Fig. 220 ^{19}F NMR spectra (471 MHz, $CDCl_3$, 25 ºC) of **5oa**

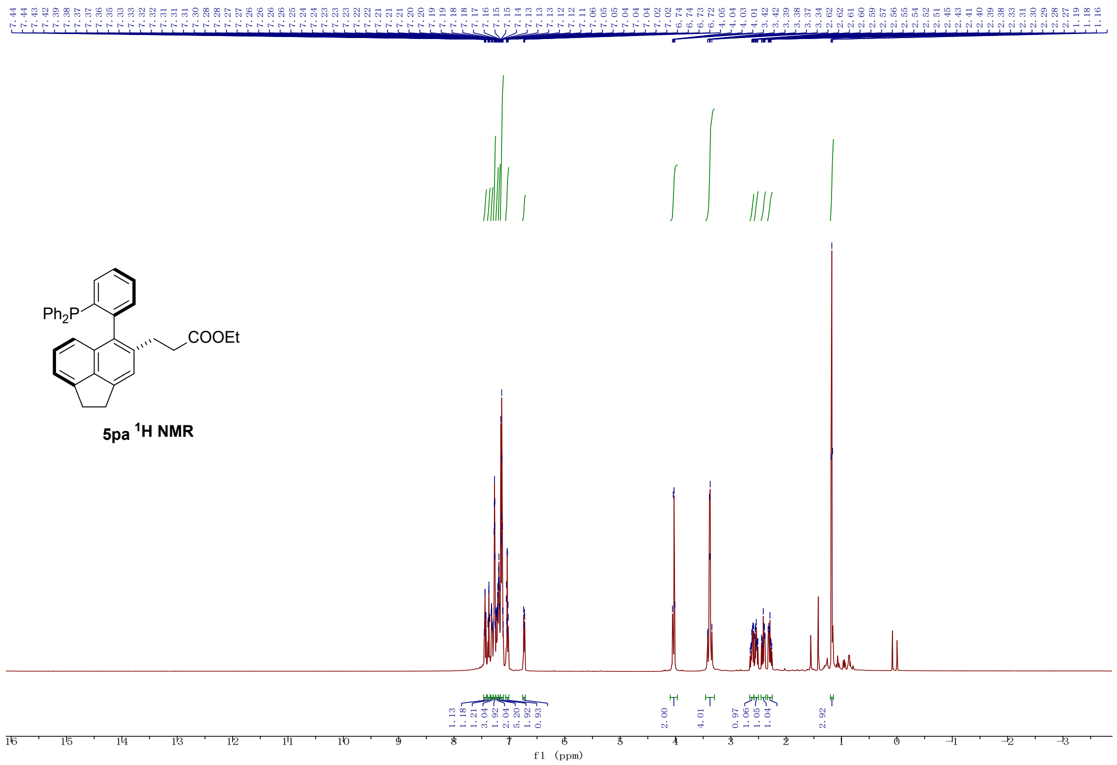

Supplementary Fig. 221 ^{1}H NMR spectra (500 MHz, CDCl3, 25 ºC) of **5pa**

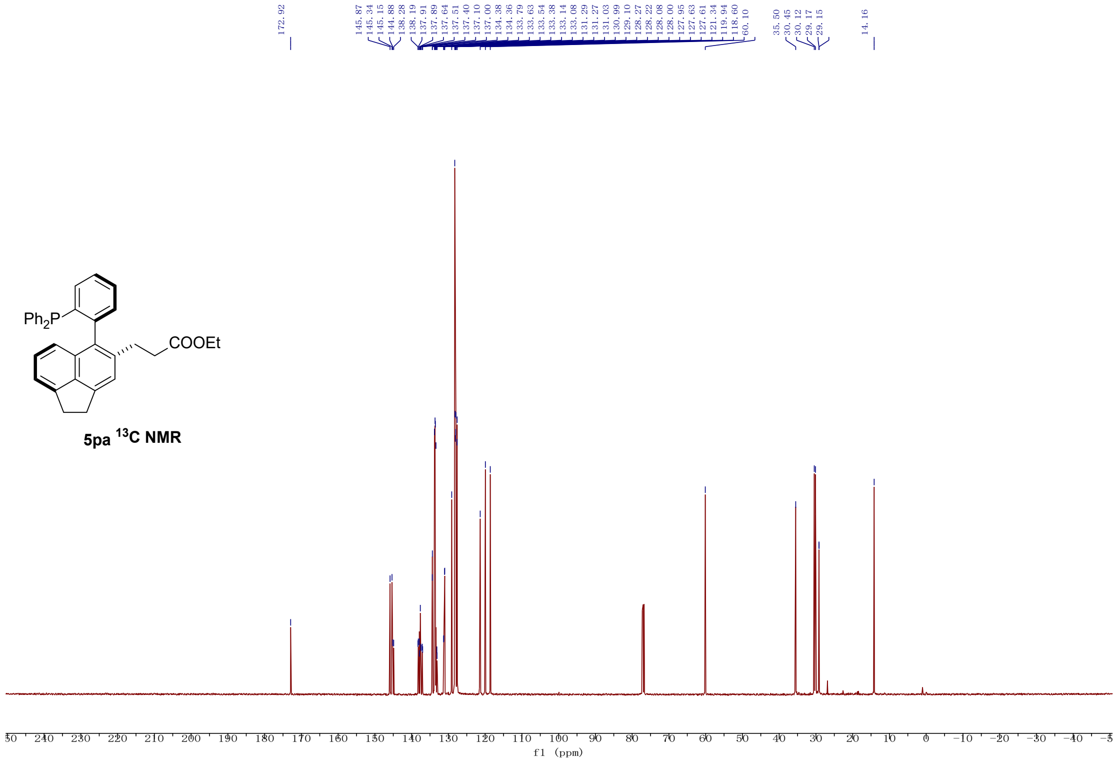

Supplementary Fig. 222. ^{13}C NMR spectra (126 MHz, CDCl3, 25 ºC) of **5pa**

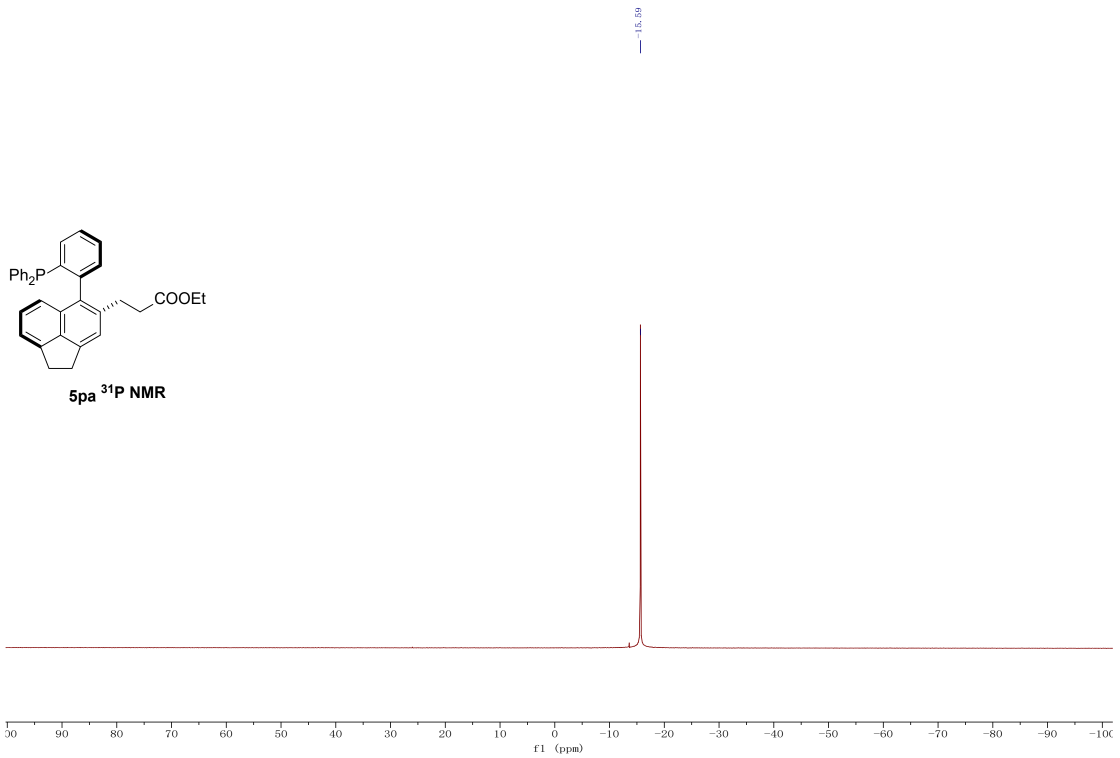

Supplementary Fig. 223 ^{31}P NMR spectra (202 MHz, $CDCl_3$, 25 ºC) of **5pa**

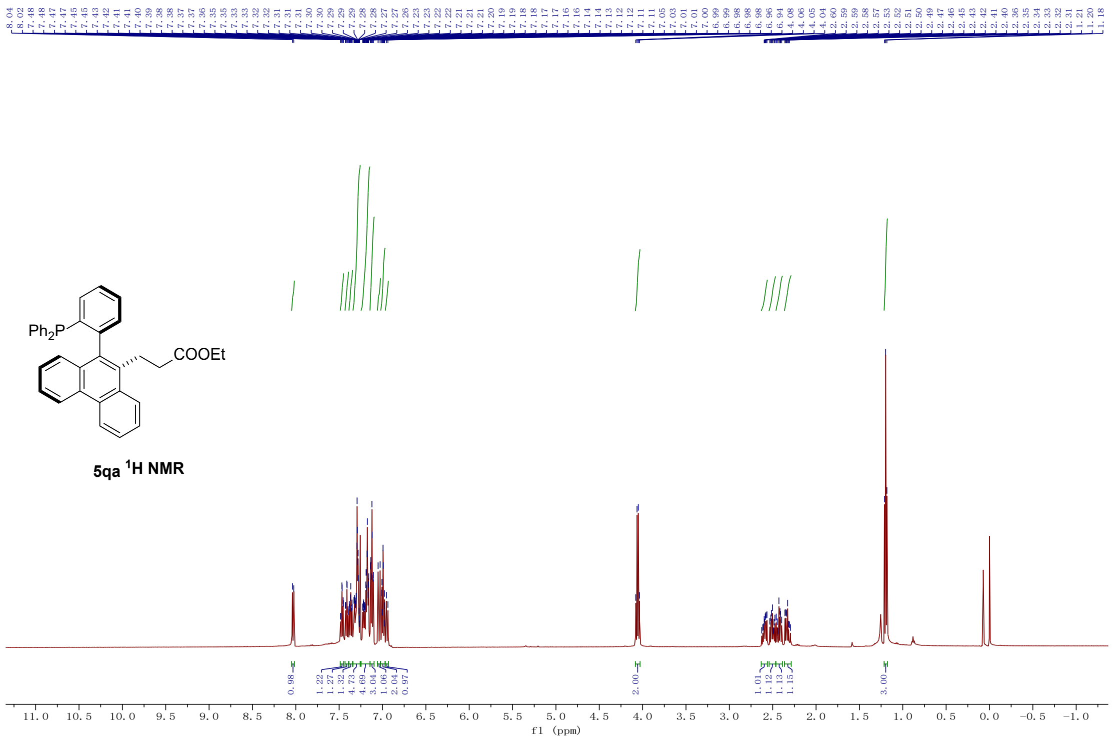

Supplementary Fig. 224 ^{1}H NMR spectra (500 MHz, $CDCl_3$, 25 ºC) of **5qa**

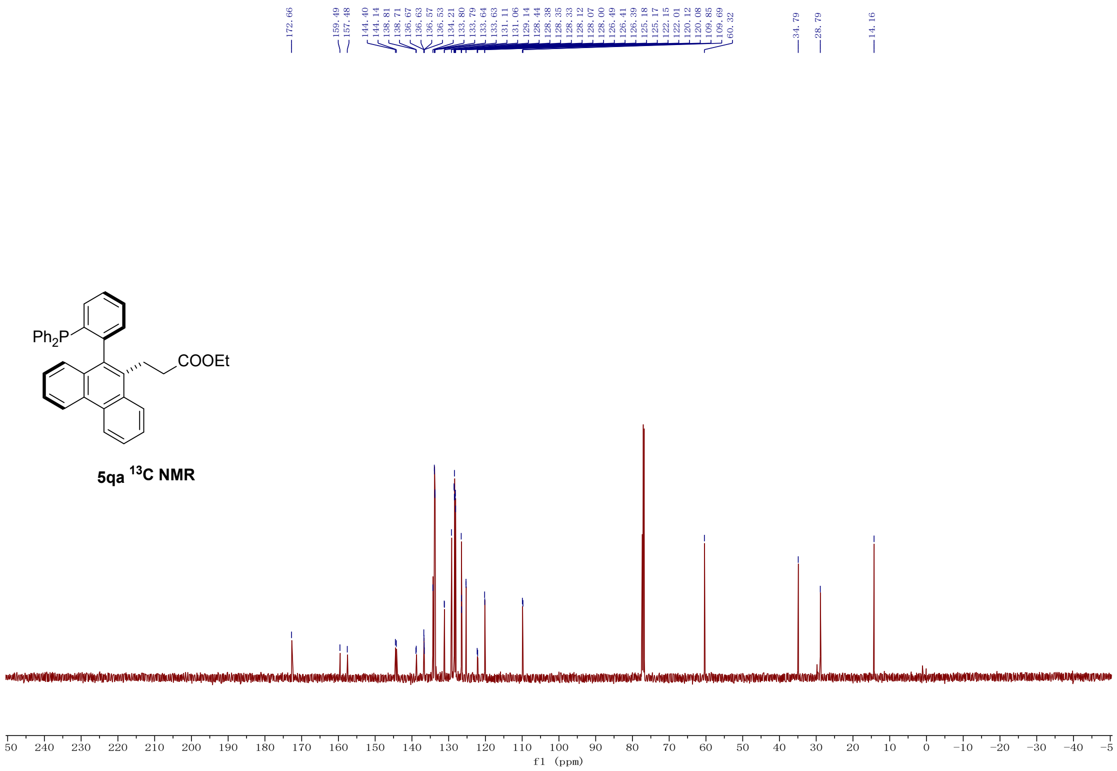

Supplementary Fig. 225. ^{13}C NMR spectra (126 MHz, $CDCl_3$, 25 °C) of **5qa**

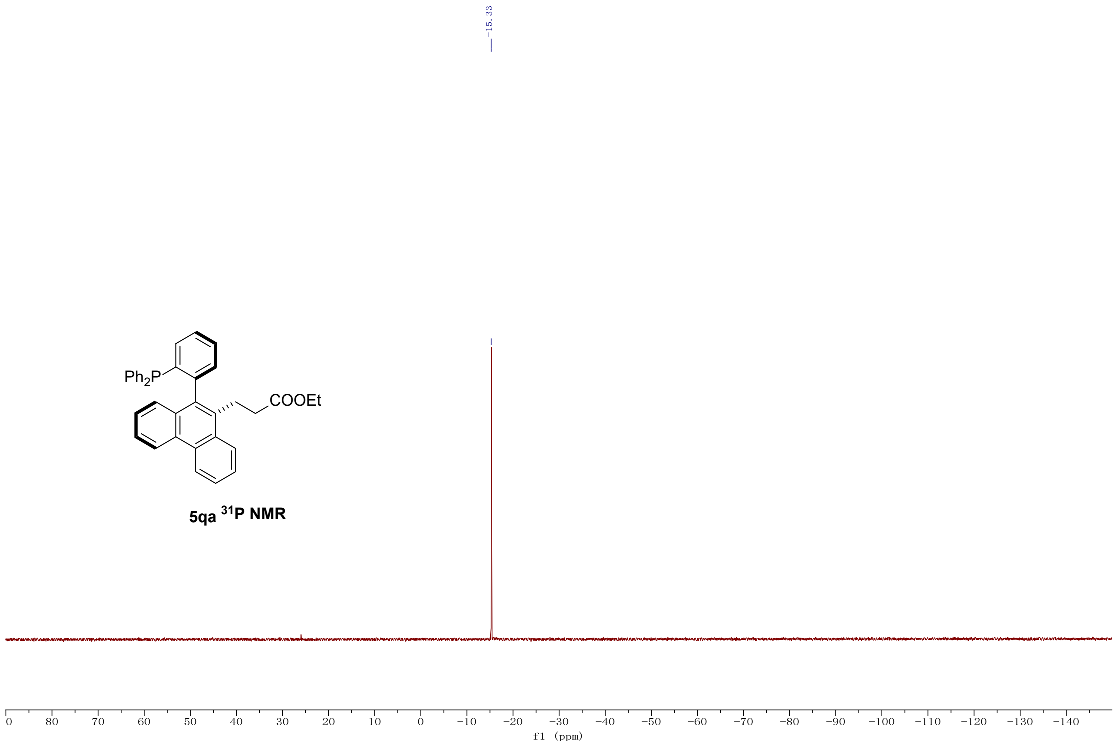

Supplementary Fig. 226 ^{31}P NMR spectra (202 MHz, $CDCl_3$, 25 °C) of **5qa**

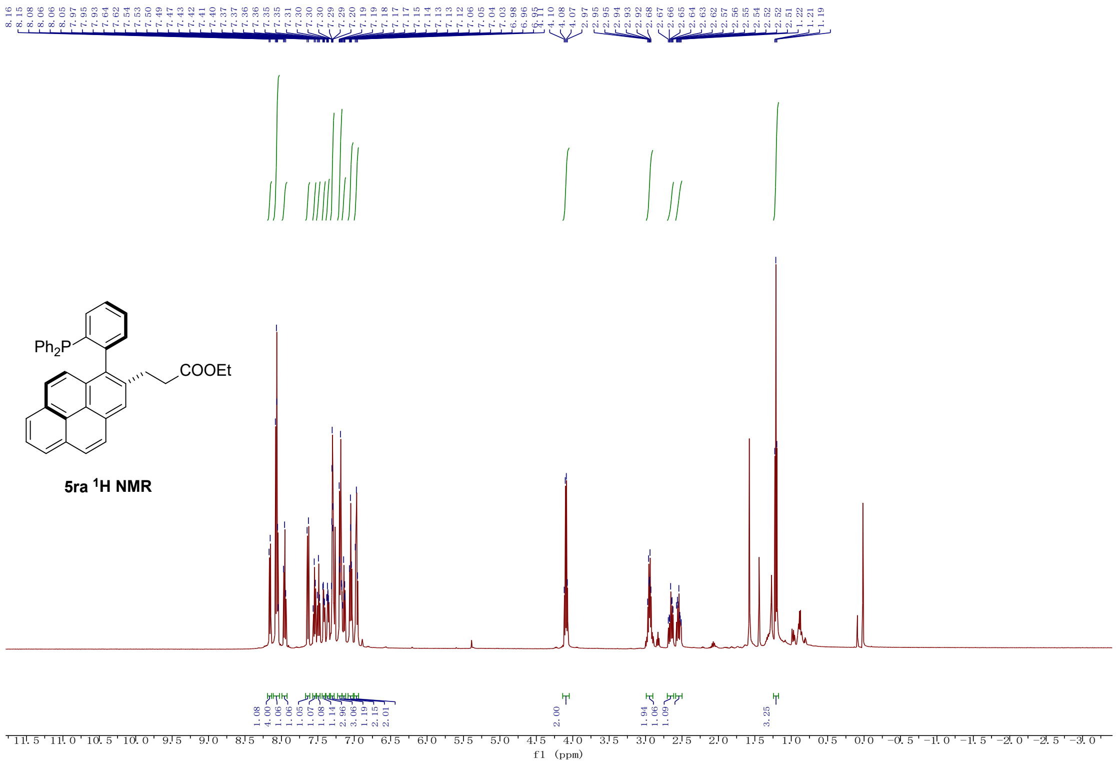

Supplementary Fig. 227 ^{1}H NMR spectra (500 MHz, $CDCl_3$, 25 ºC) of **5ra**

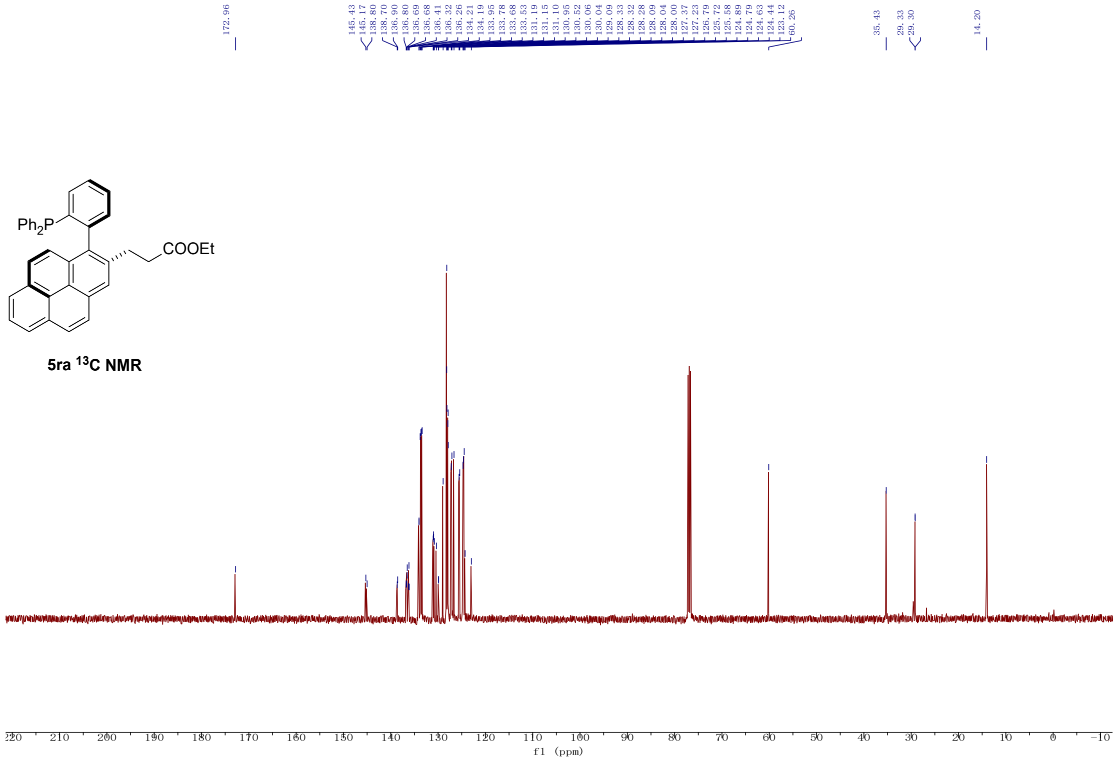

Supplementary Fig. 228. ^{13}C NMR spectra (126 MHz, $CDCl_3$, 25 ºC) of **5ra**

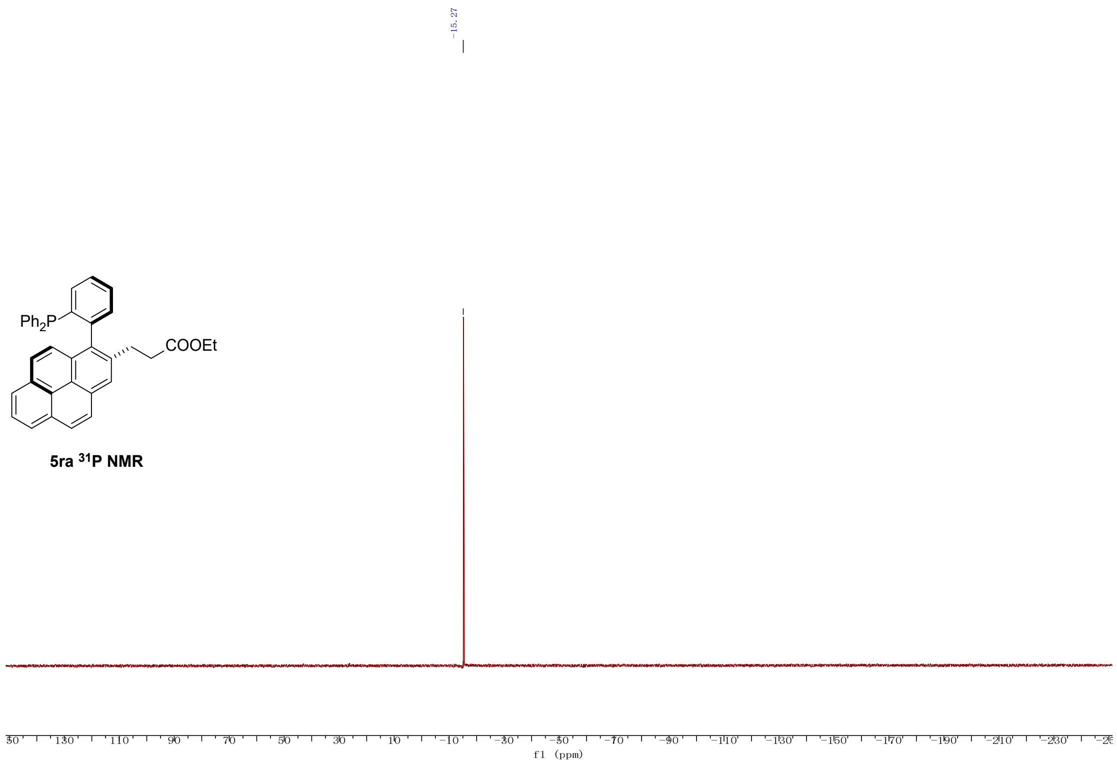

Supplementary Fig. 229 ^{31}P NMR spectra (202 MHz, $CDCl_3$, 25 ºC) of **5ra**

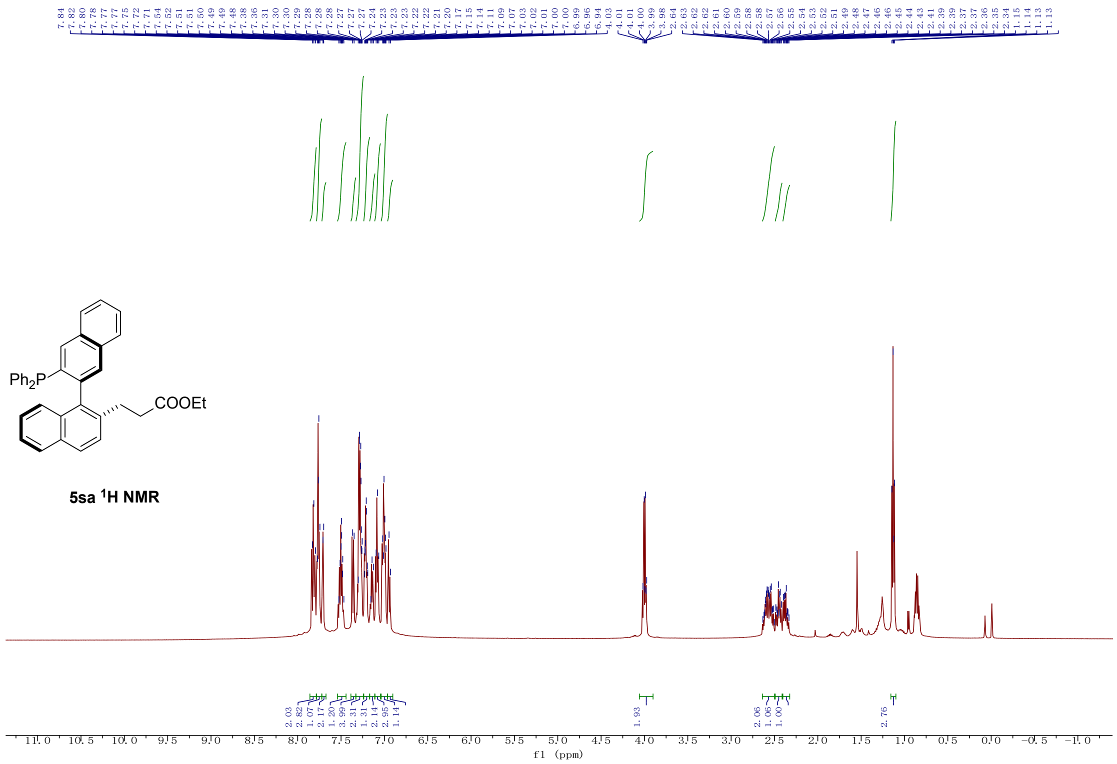

Supplementary Fig. 230 ^{1}H NMR spectra (500 MHz, $CDCl_3$, 25 ºC) of **5sa**

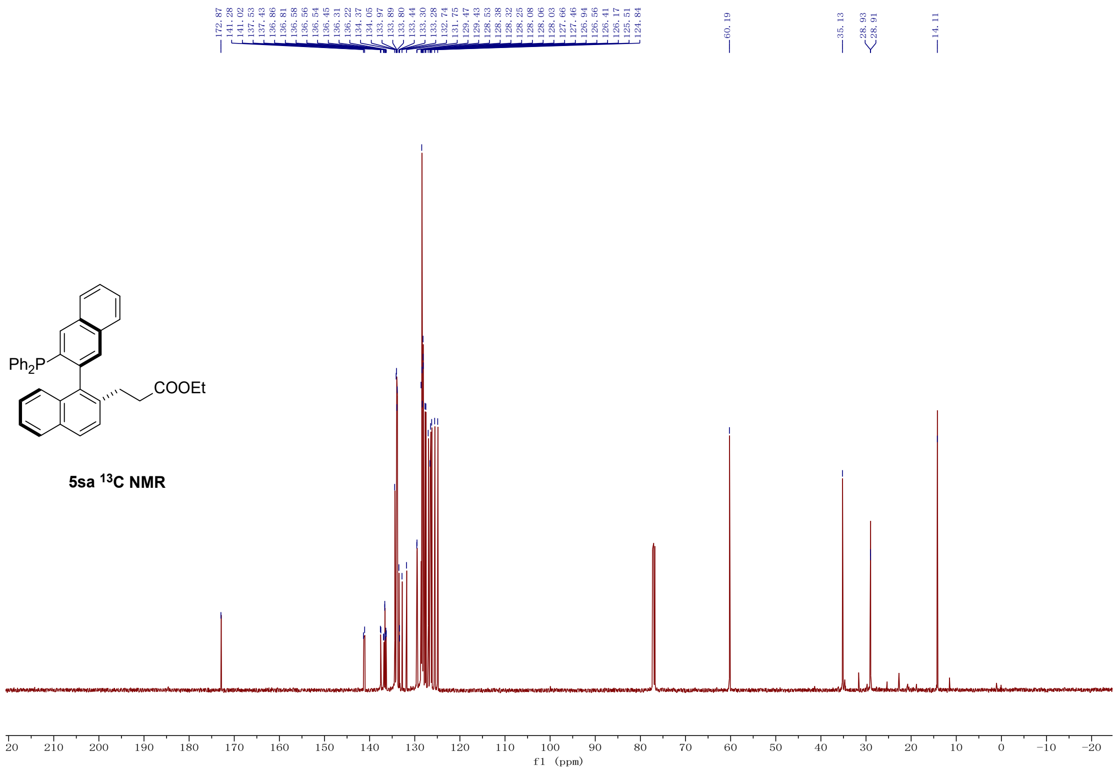

Supplementary Fig. 231. ^{13}C NMR spectra (126 MHz, $CDCl_3$, 25 ºC) of **5sa**

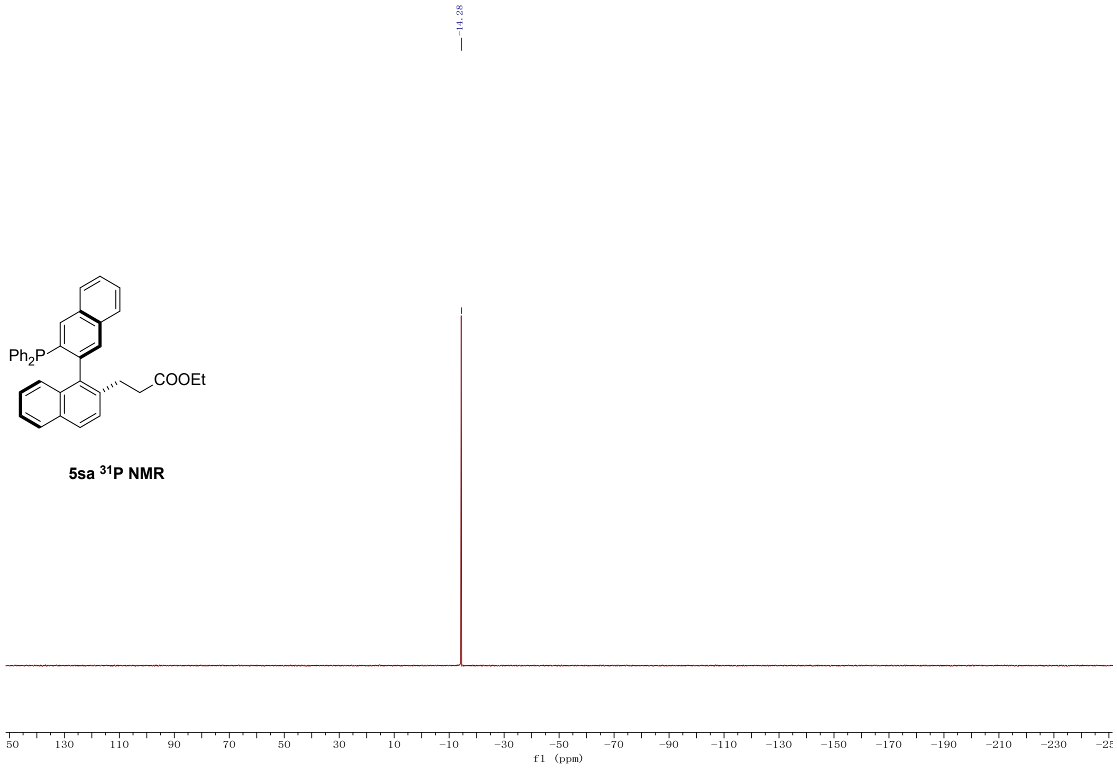

Supplementary Fig. 232 ^{31}P NMR spectra (202 MHz, $CDCl_3$, 25 ºC) of **5sa**

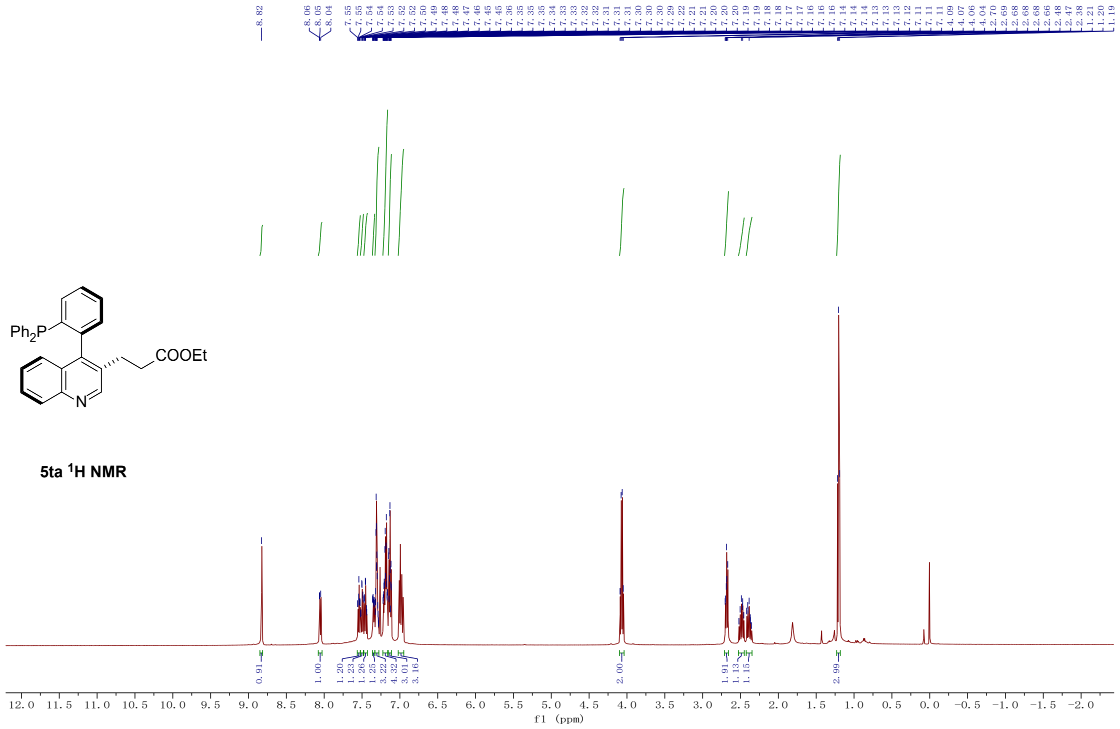

Supplementary Fig. 233 ^{1}H NMR spectra (500 MHz, $CDCl_3$, 25 °C) of **5ta**

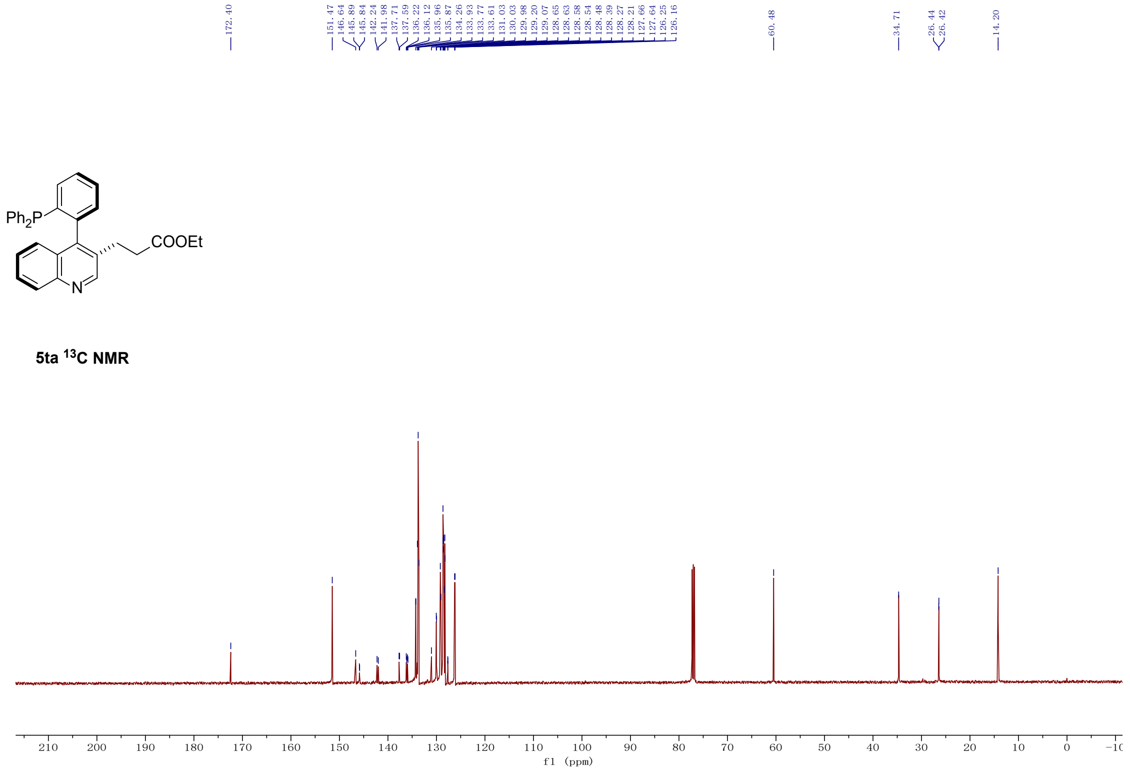

Supplementary Fig. 234. ^{13}C NMR spectra (126 MHz, $CDCl_3$, 25 °C) of **5ta**

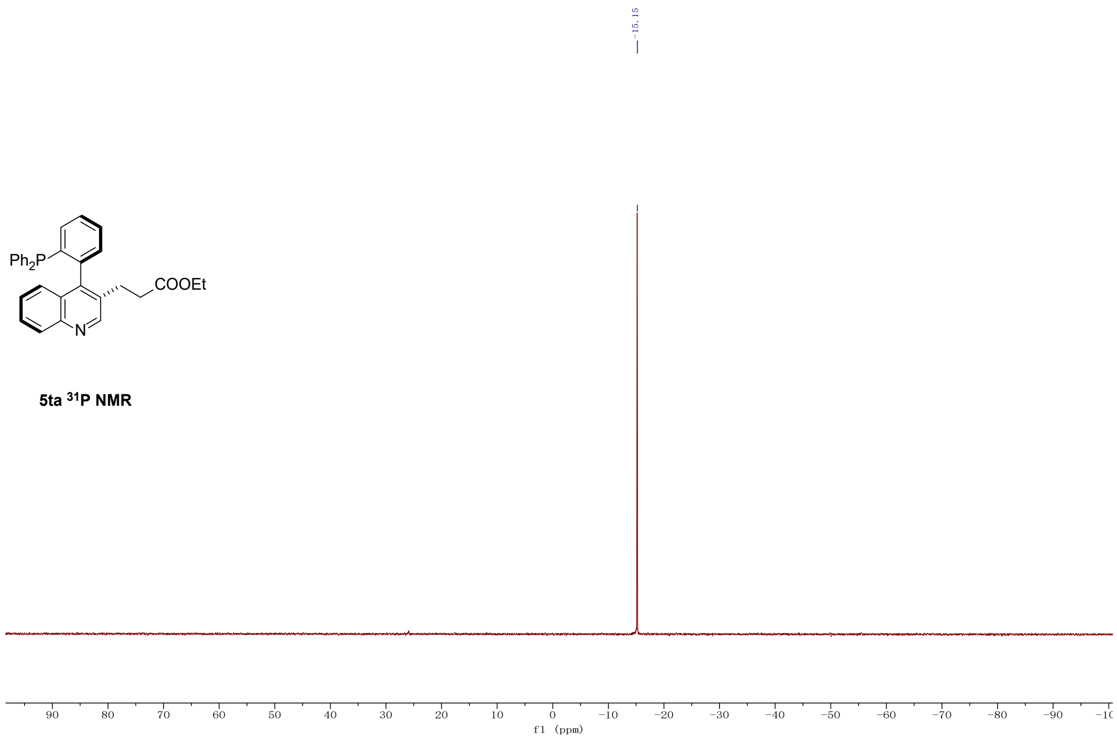

Supplementary Fig. 235 ^{31}P NMR spectra (202 MHz, $CDCl_3$, 25 °C) of **5ta**

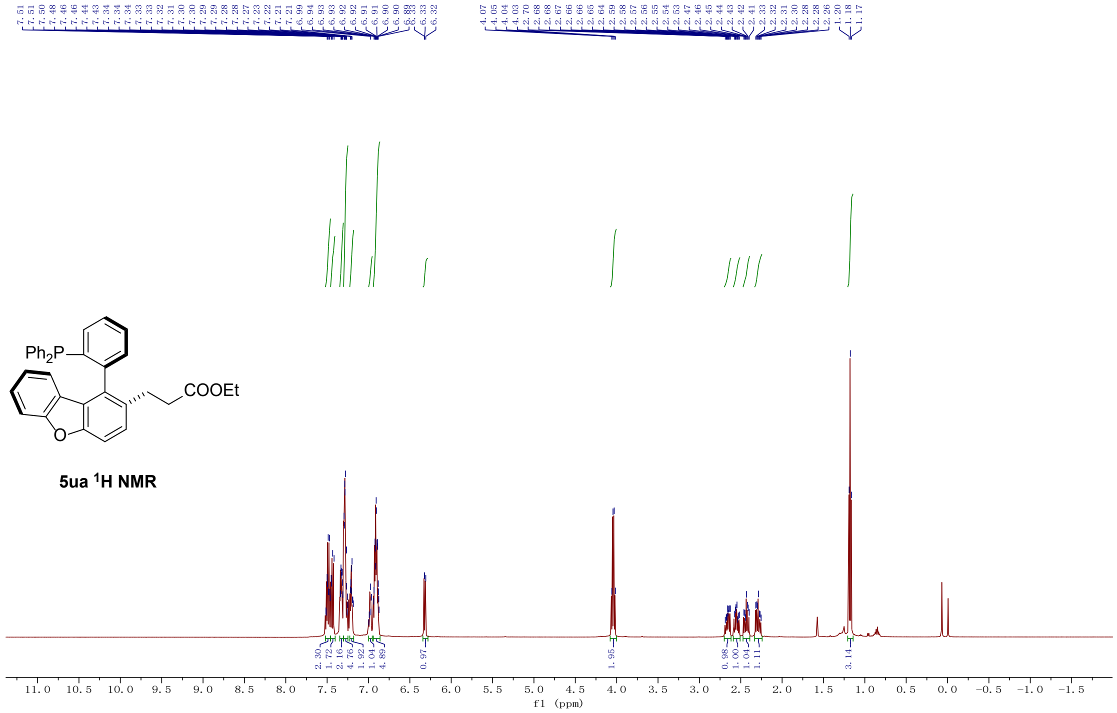

Supplementary Fig. 236 ^{1}H NMR spectra (500 MHz, $CDCl_3$, 25 °C) of **5ua**

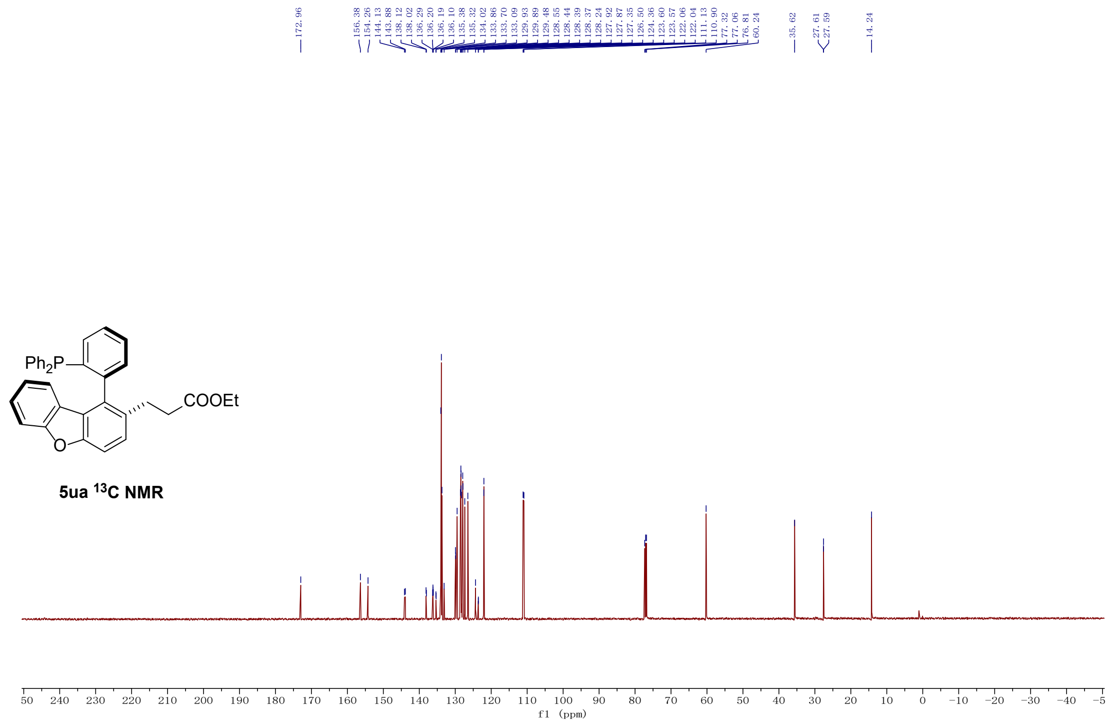

Supplementary Fig. 237. ^{13}C NMR spectra (126 MHz, $CDCl_3$, 25 °C) of **5ua**

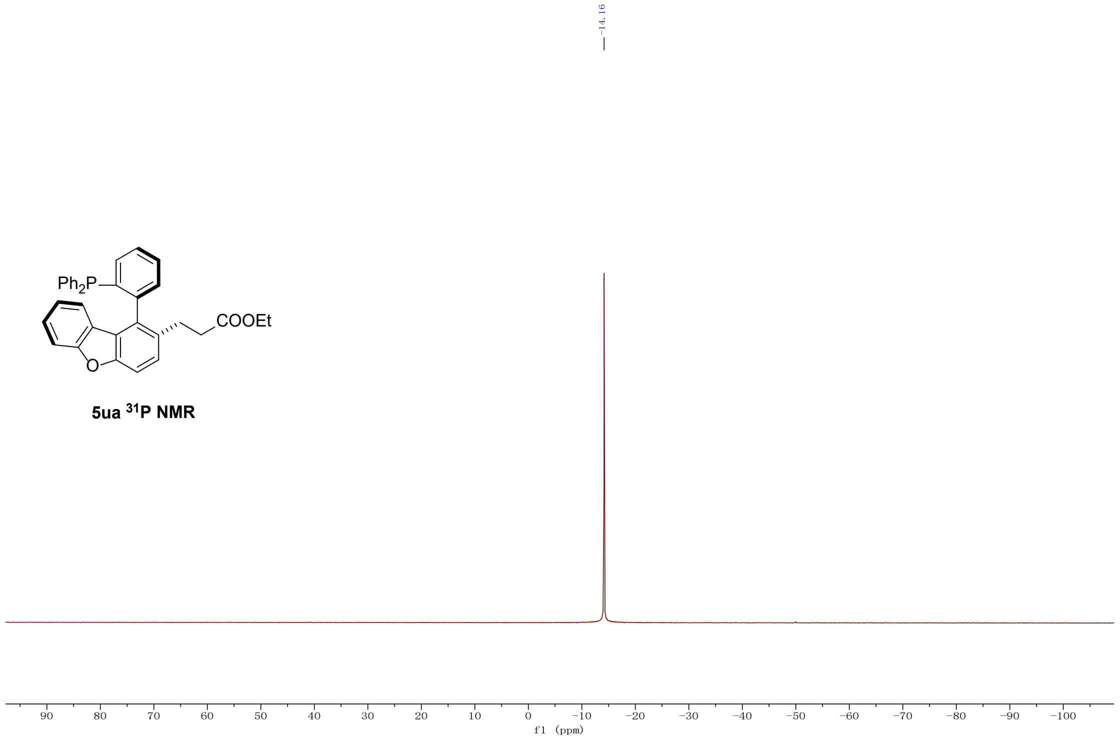

Supplementary Fig. 238 ^{31}P NMR spectra (202 MHz, $CDCl_3$, 25 °C) of **5ua**

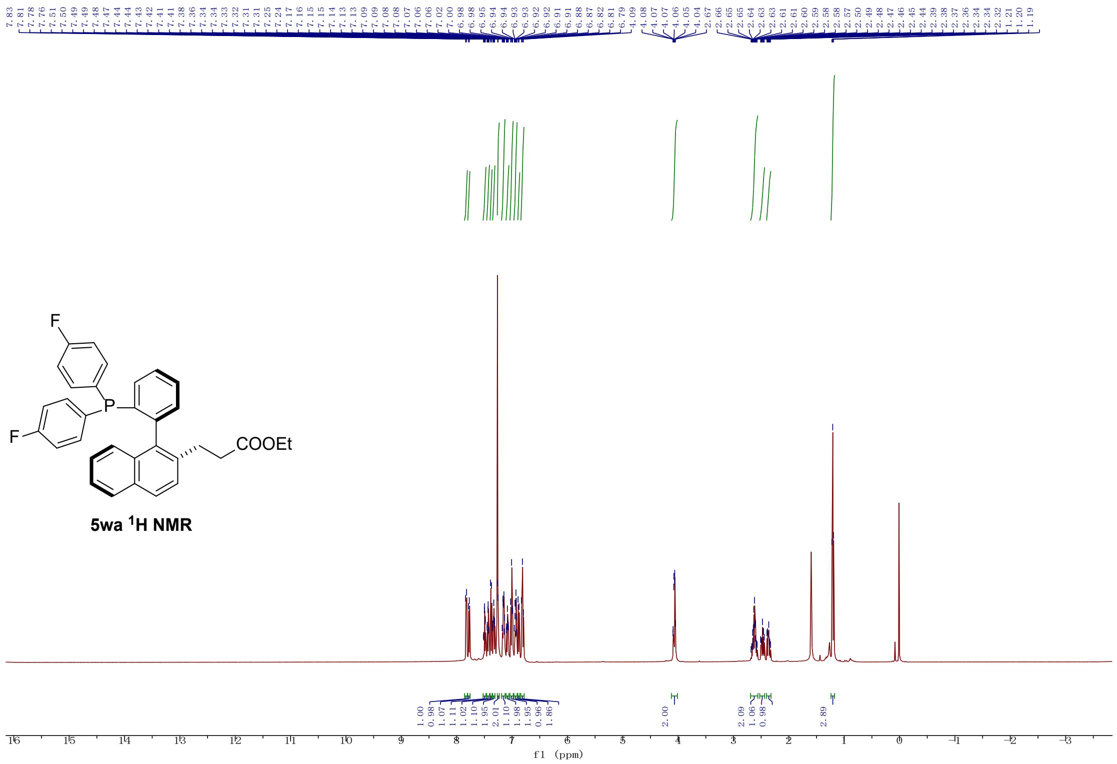

Supplementary Fig. 239 ^{1}H NMR spectra (500 MHz, $CDCl_3$, 25 ºC) of **5wa**

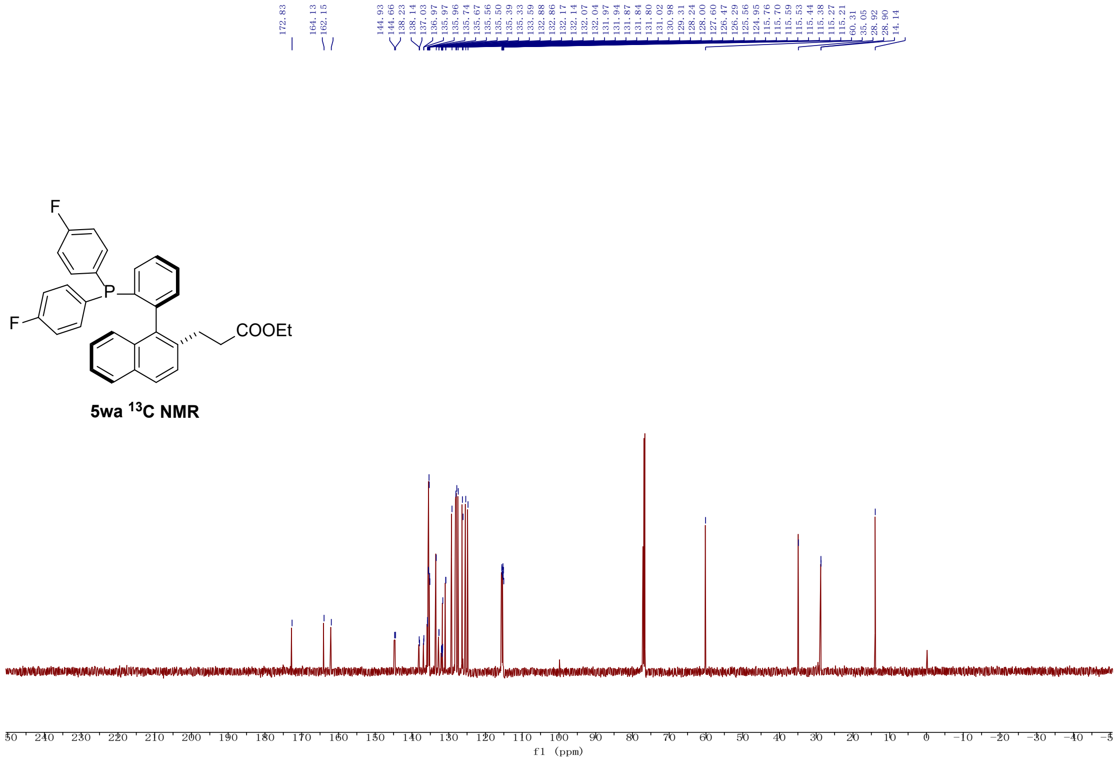

Supplementary Fig. 240. ^{13}C NMR spectra (126 MHz, $CDCl_3$, 25 ºC) of **5wa**

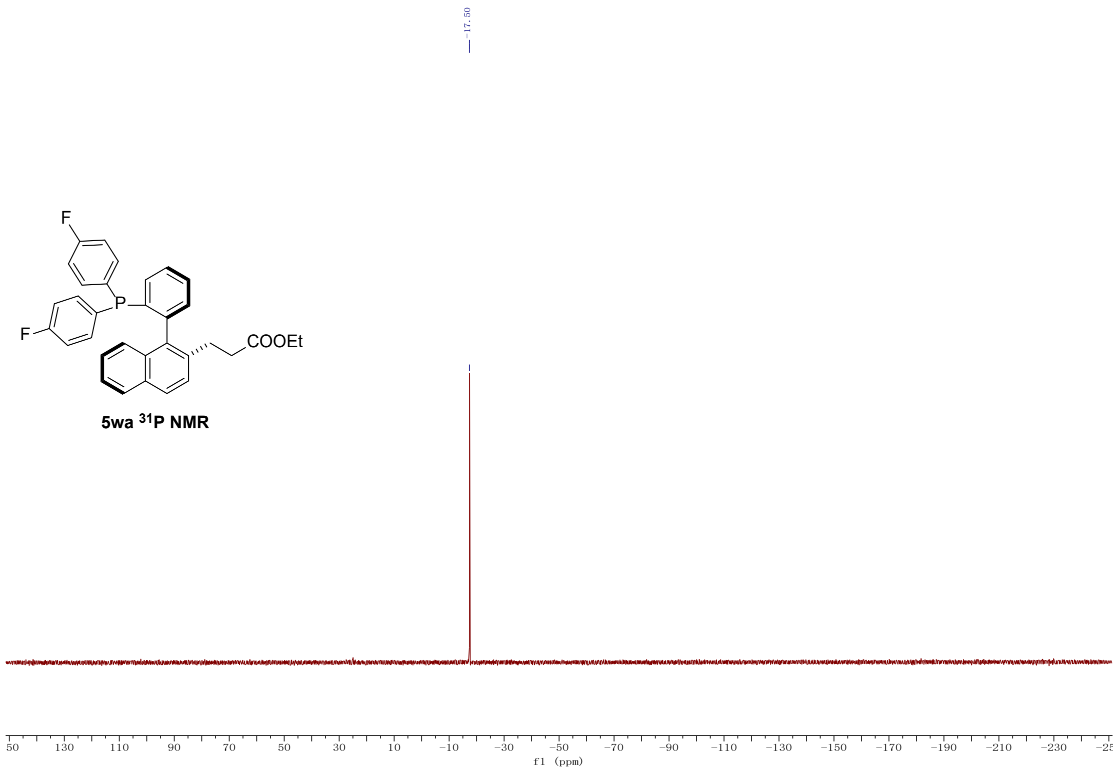

Supplementary Fig. 241 ^{31}P NMR spectra (202 MHz, $CDCl_3$, 25 ºC) of **5wa**

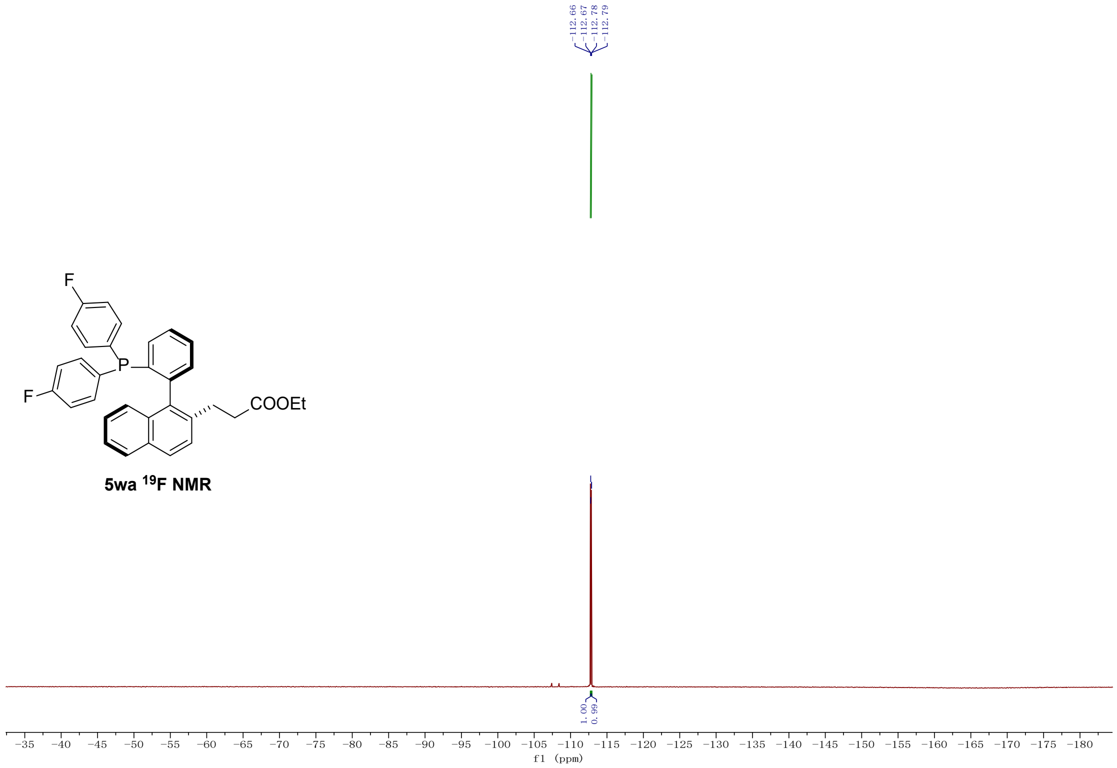

Supplementary Fig. 242 ^{19}F NMR spectra (471 MHz, $CDCl_3$, 25 ºC) of **5wa**

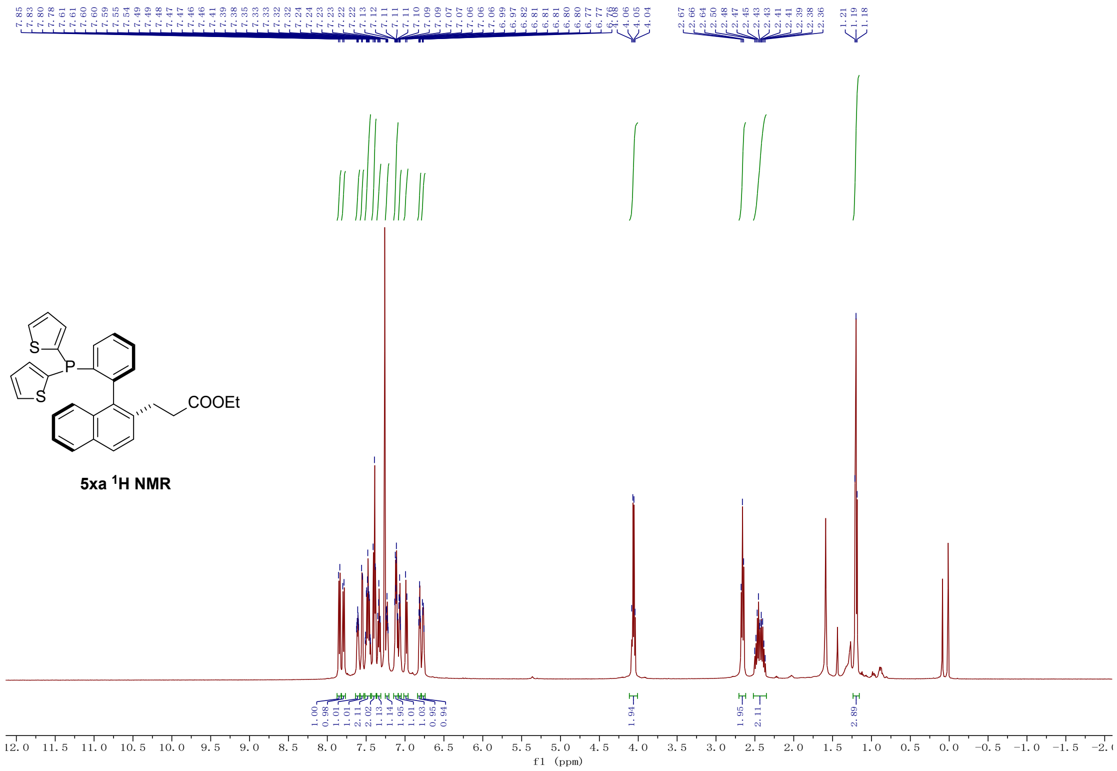

Supplementary Fig. 243 ^{1}H NMR spectra (500 MHz, $CDCl_3$, 25 °C) of **5xa**

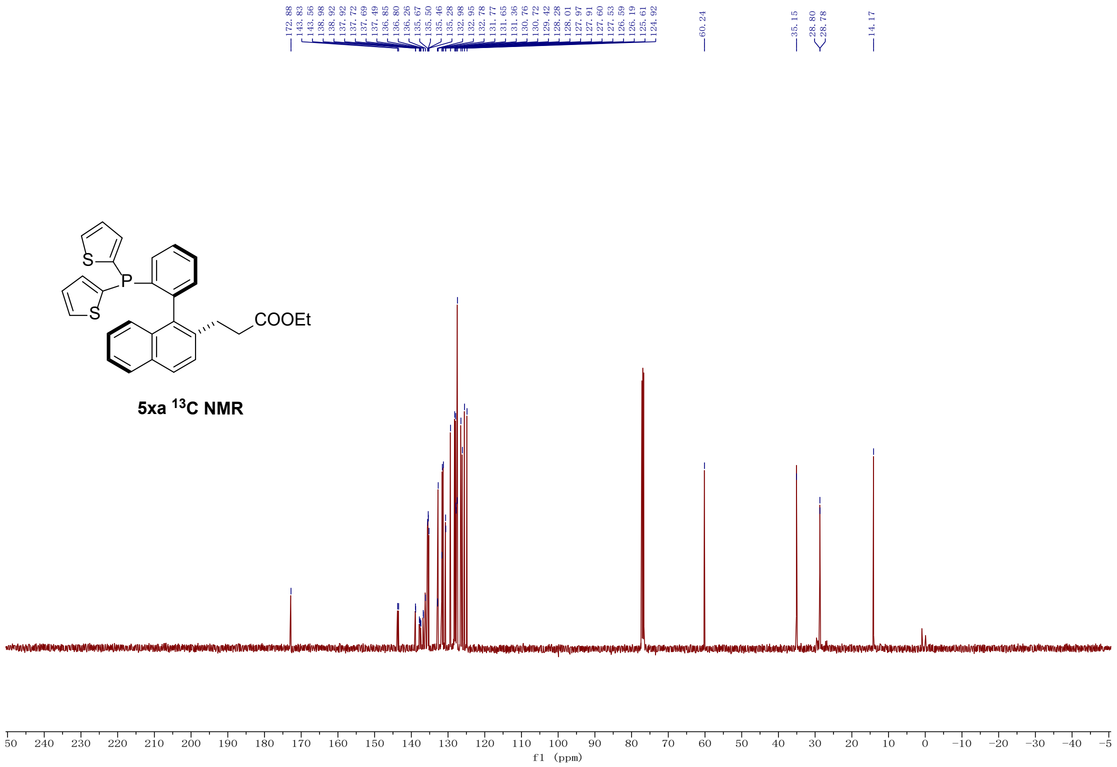

Supplementary Fig. 244. ^{13}C NMR spectra (126 MHz, $CDCl_3$, 25 °C) of **5xa**

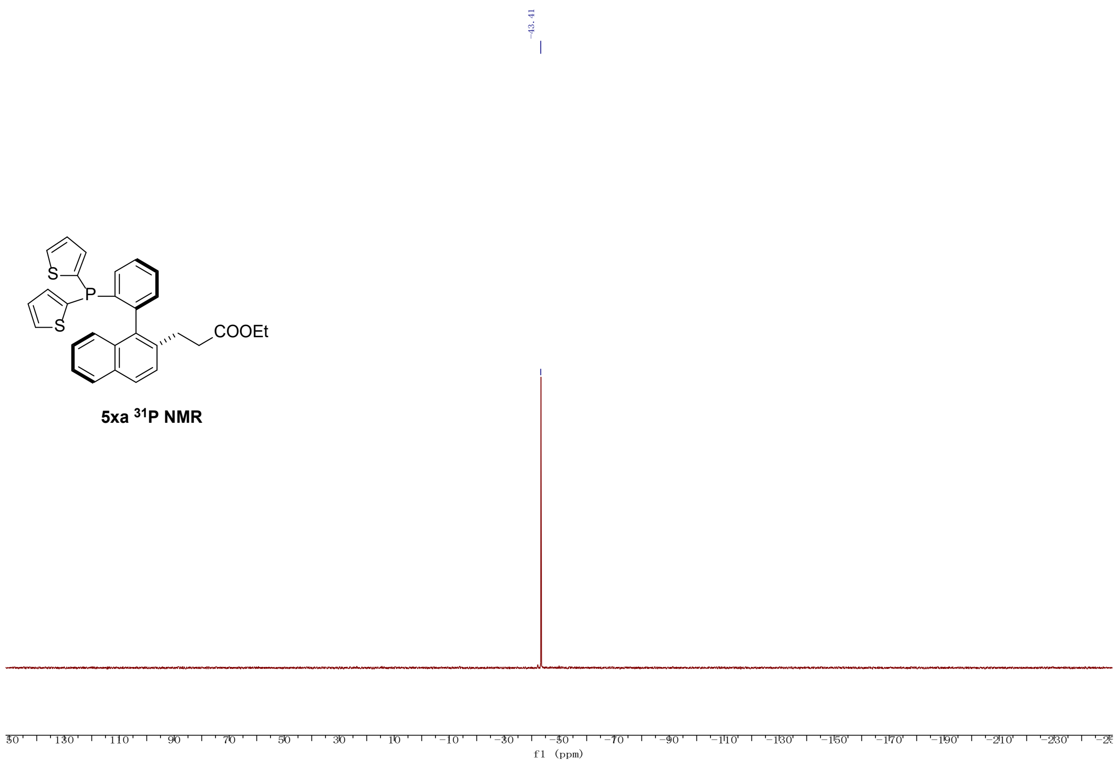

Supplementary Fig. 245 ^{31}P NMR spectra (202 MHz, $CDCl_3$, 25 °C) of **5xa**

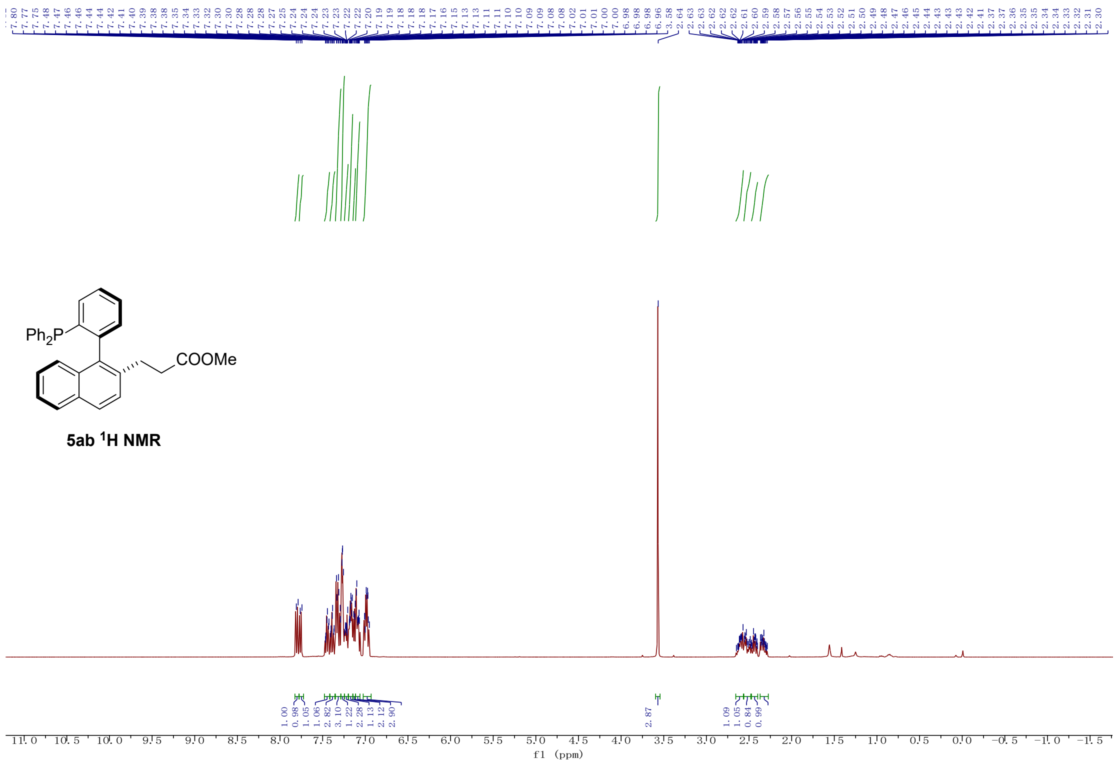

Supplementary Fig. 246 ^{1}H NMR spectra (400 MHz, $CDCl_3$, 25 °C) of **5ab**

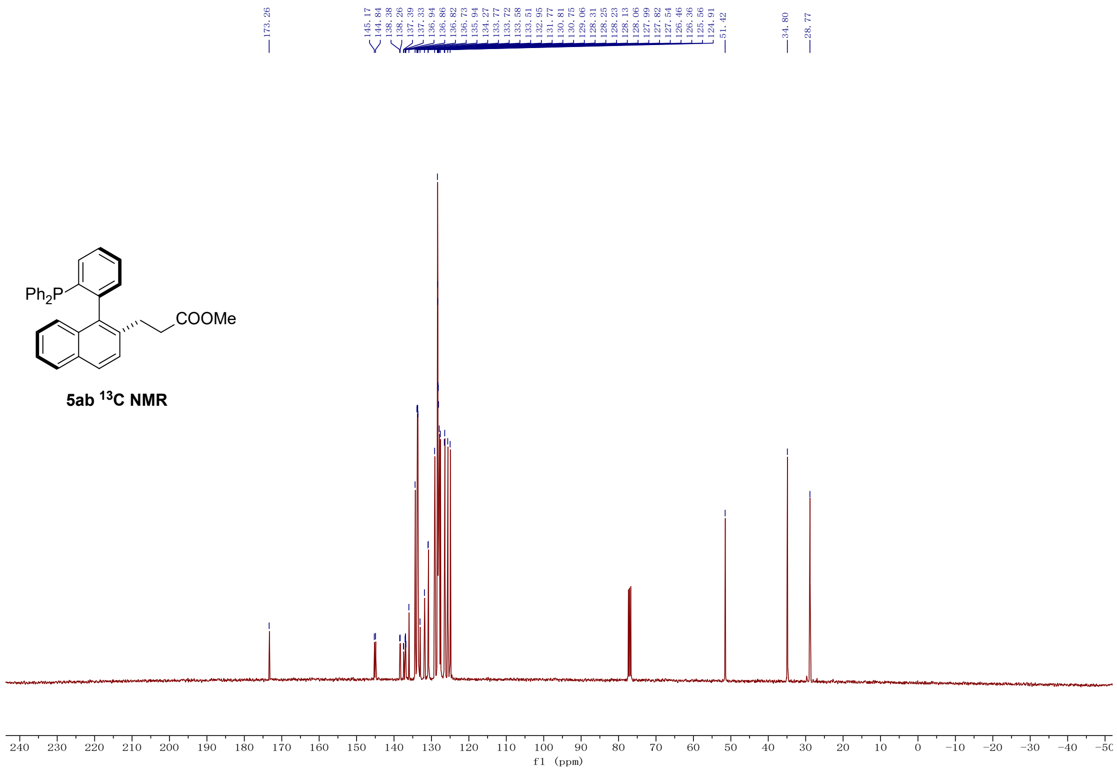

Supplementary Fig. 247. ^{13}C NMR spectra (101 MHz, $CDCl_3$, 25 ºC) of **5ab**

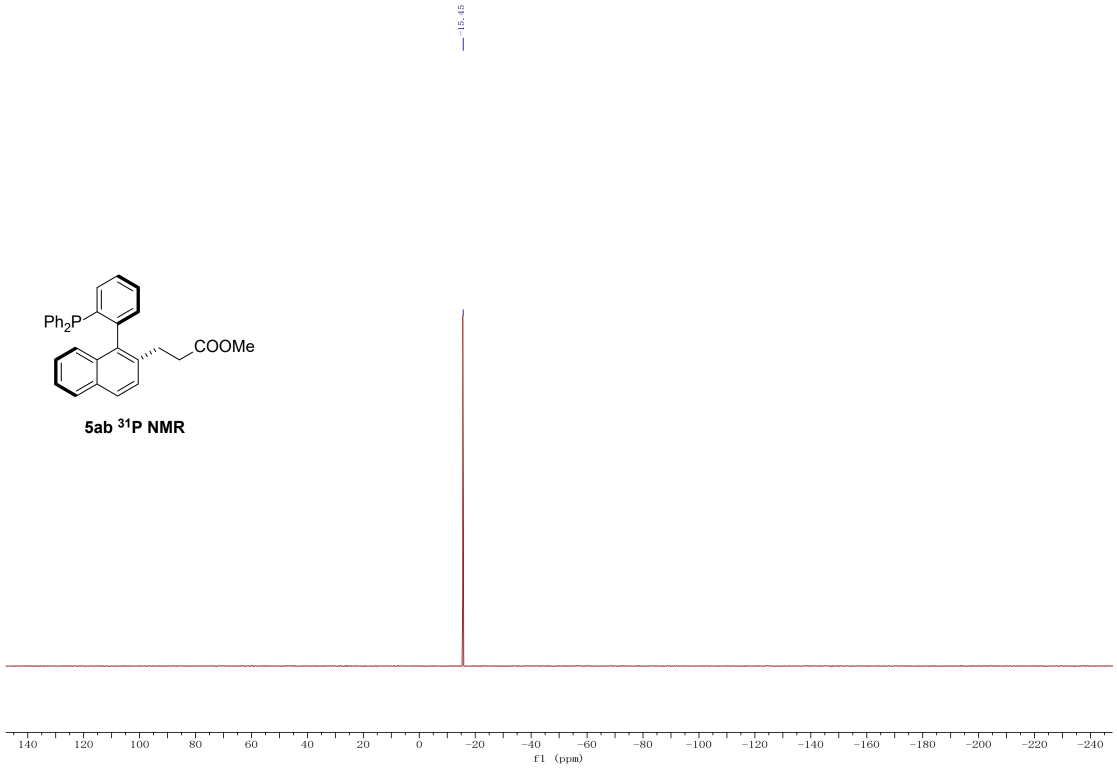

Supplementary Fig. 248 ^{31}P NMR spectra (162 MHz, $CDCl_3$, 25 ºC) of **5ab**

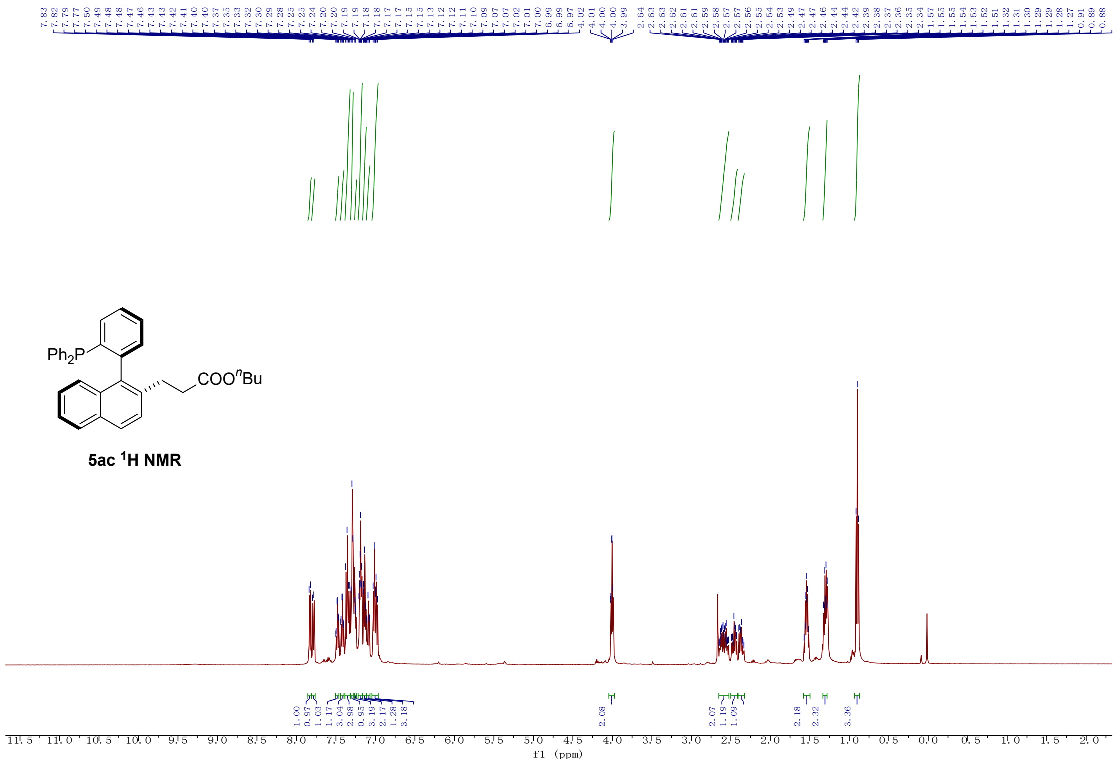

Supplementary Fig. 249 ^{1}H NMR spectra (500 MHz, $CDCl_3$, 25 ºC) of **5ac**

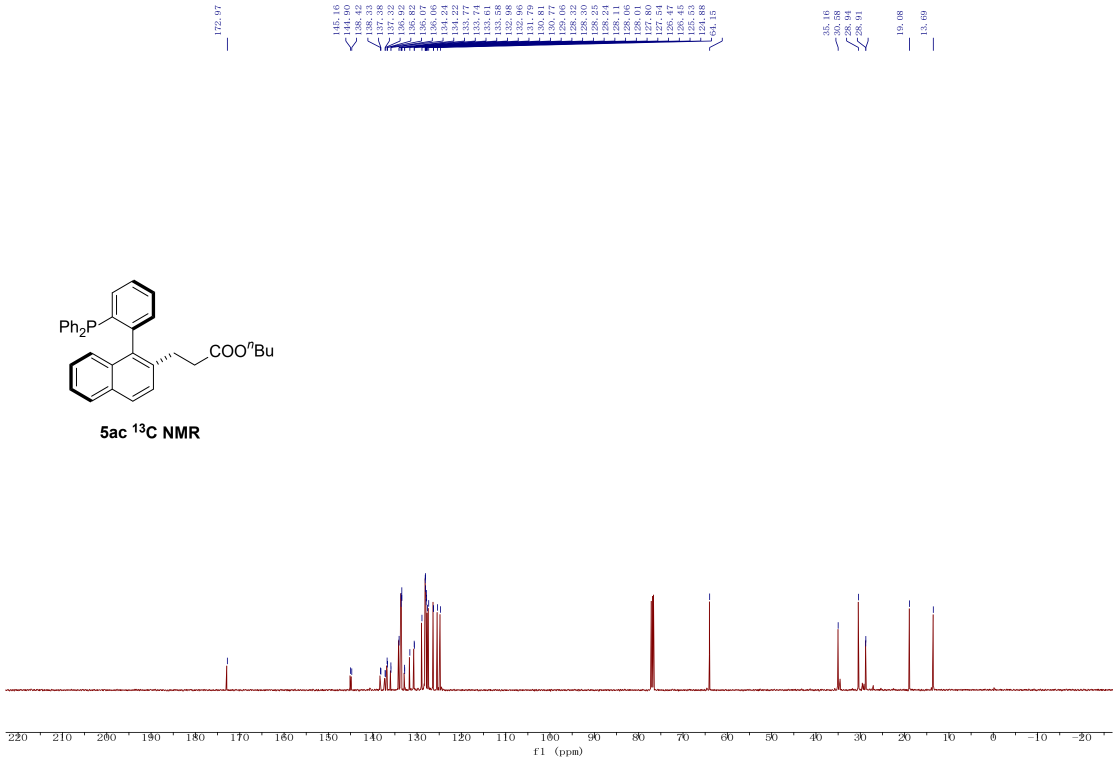

Supplementary Fig. 250. ^{13}C NMR spectra (126 MHz, $CDCl_3$, 25 ºC) of **5ac**

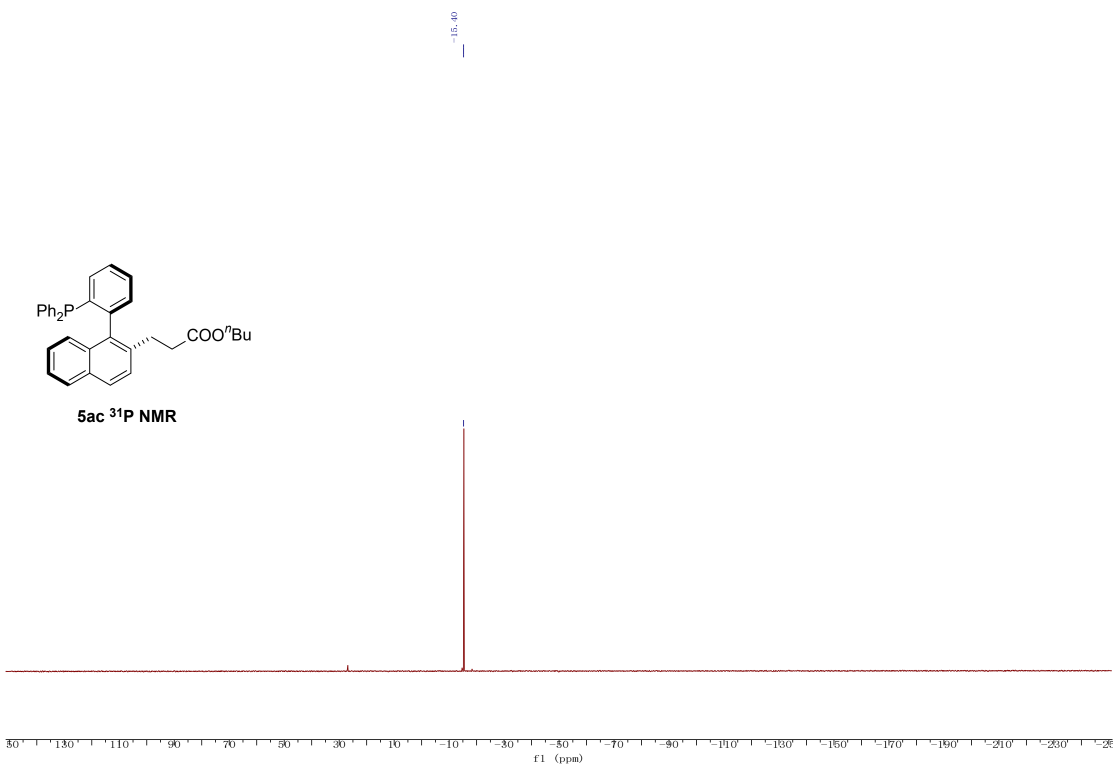

Supplementary Fig. 251. ^{31}P NMR spectra (202 MHz, CDCl$_3$, 25 °C) of **5ac**

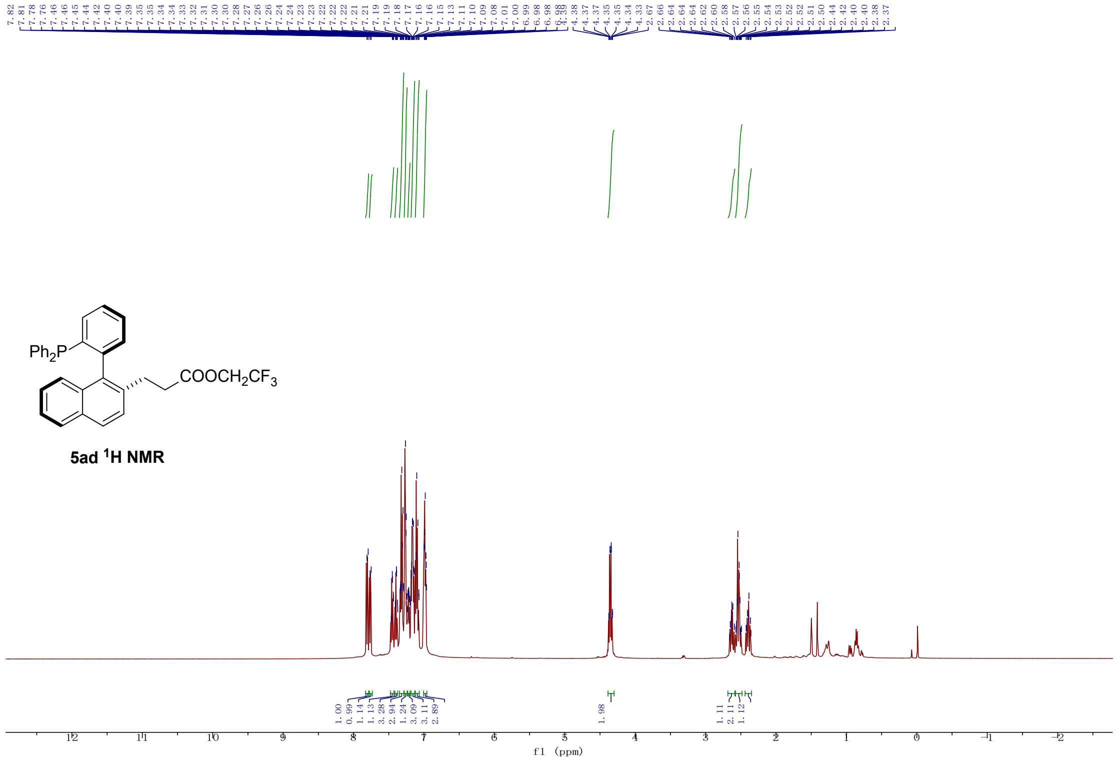

Supplementary Fig. 252 ^{1}H NMR spectra (500 MHz, CDCl$_3$, 25 °C) of **5ad**

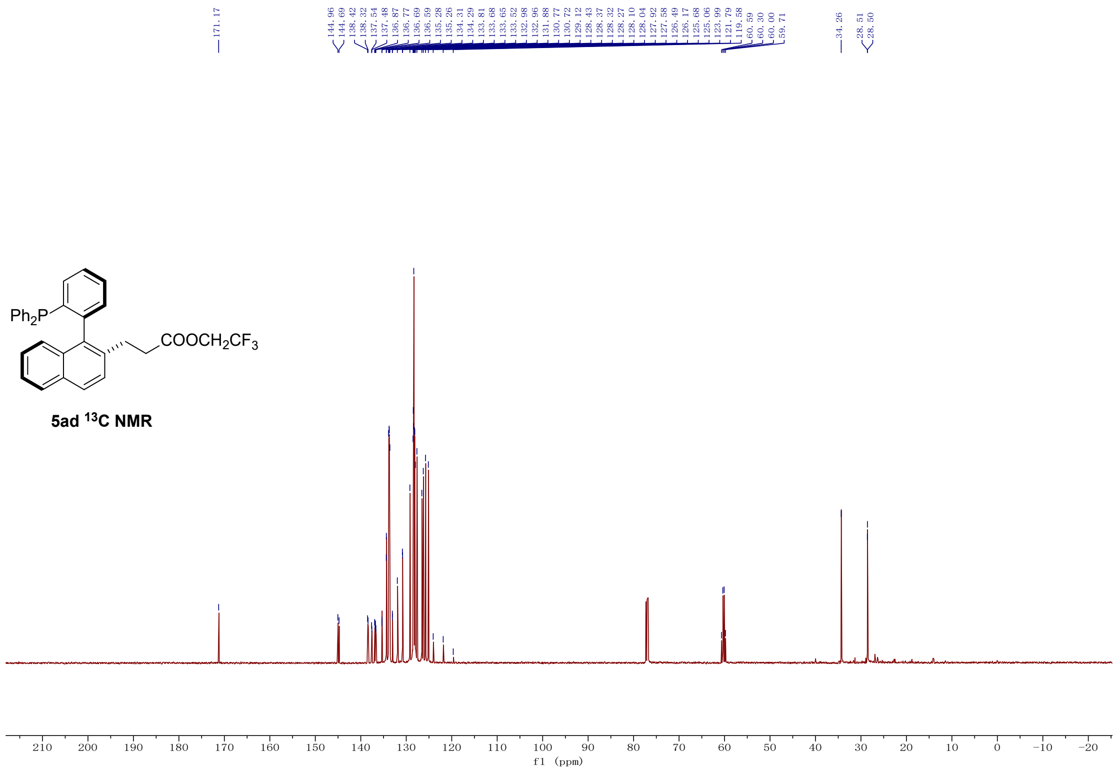

Supplementary Fig. 253. ^{13}C NMR spectra (126 MHz, $CDCl_3$, 25 °C) of **5ad**

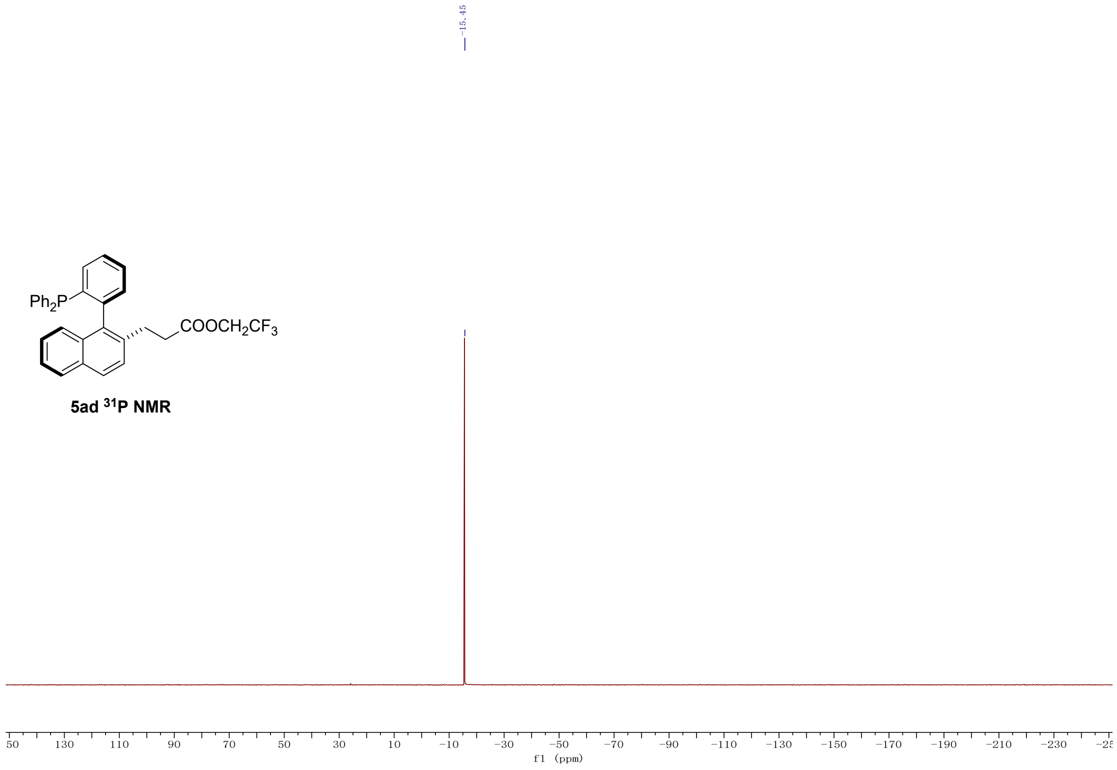

Supplementary Fig. 254. ^{31}P NMR spectra (202 MHz, $CDCl_3$, 25 °C) of **5ad**

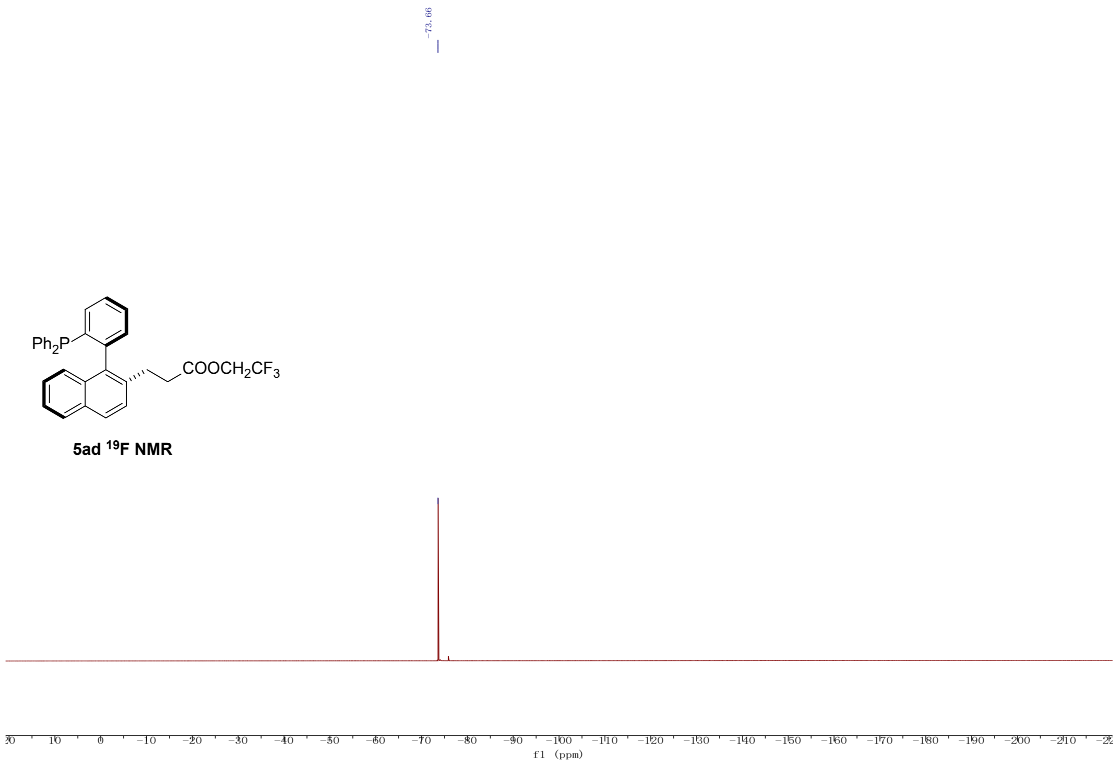

Supplementary Fig. 255. ^{19}F NMR spectra (471 MHz, CDCl$_3$, 25 °C) of **5ad**

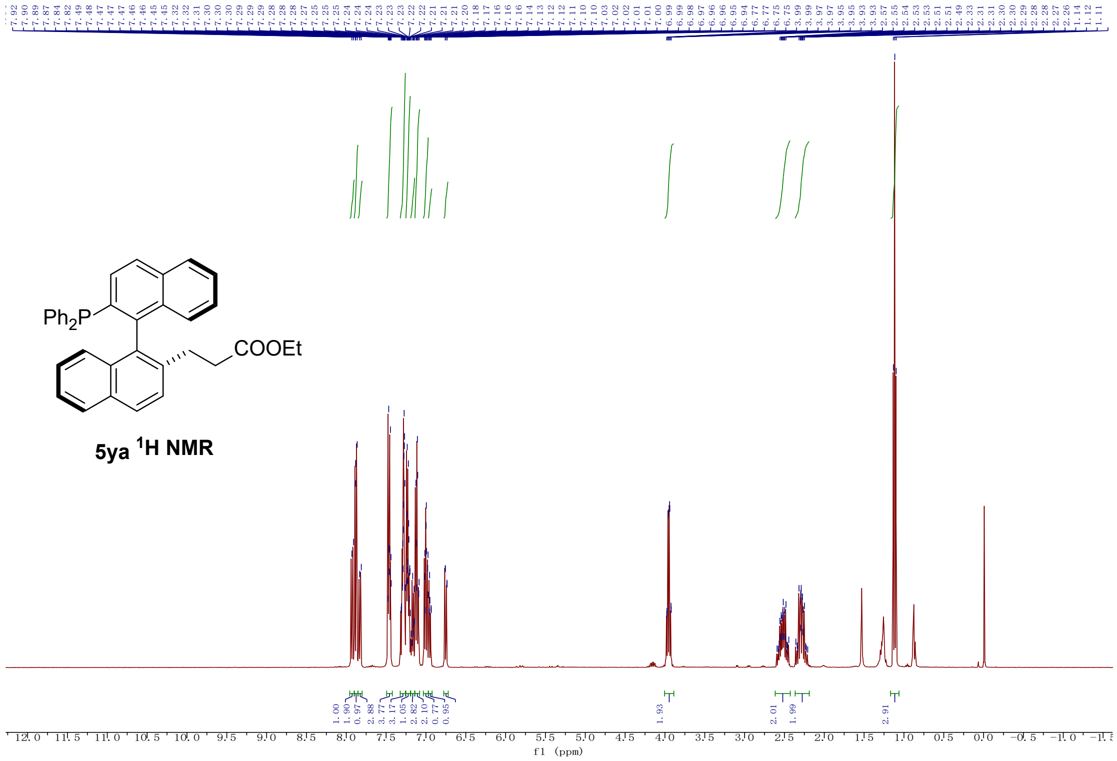

Supplementary Fig. 256 ^{1}H NMR spectra (400 MHz, CDCl$_3$, 25 °C) of **5ya**

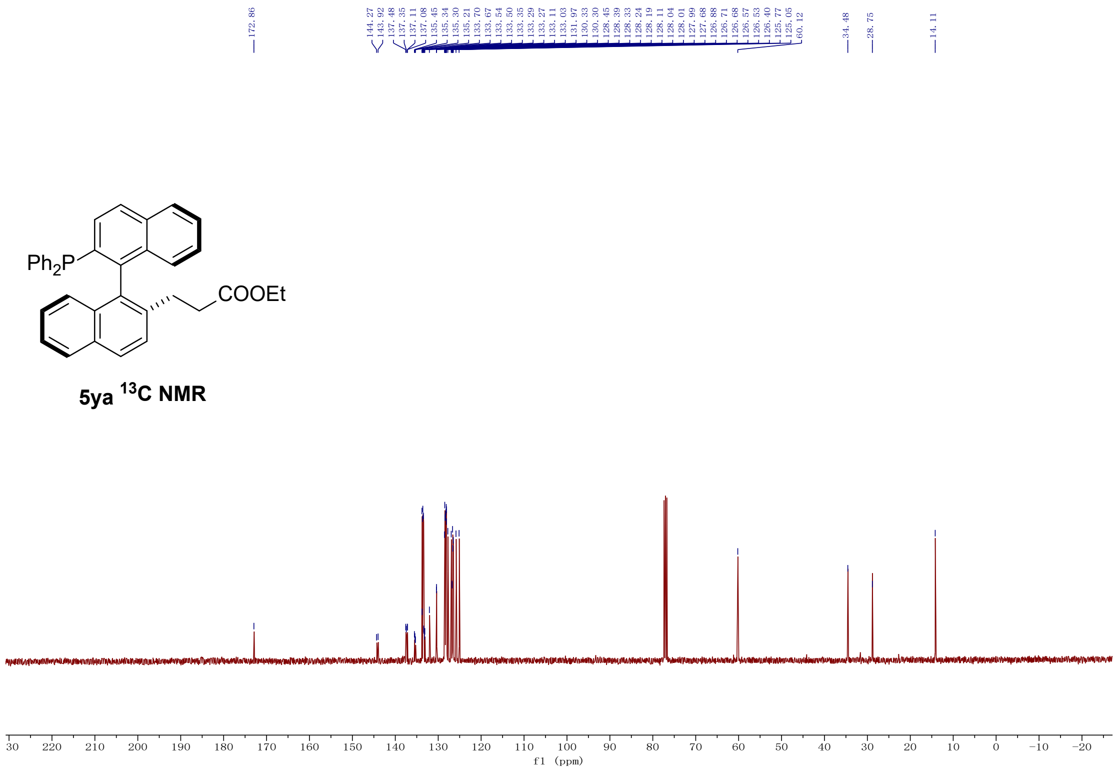

Supplementary Fig. 257. ^{13}C NMR spectra (101 MHz, $CDCl_3$, 25 °C) of **5ya**

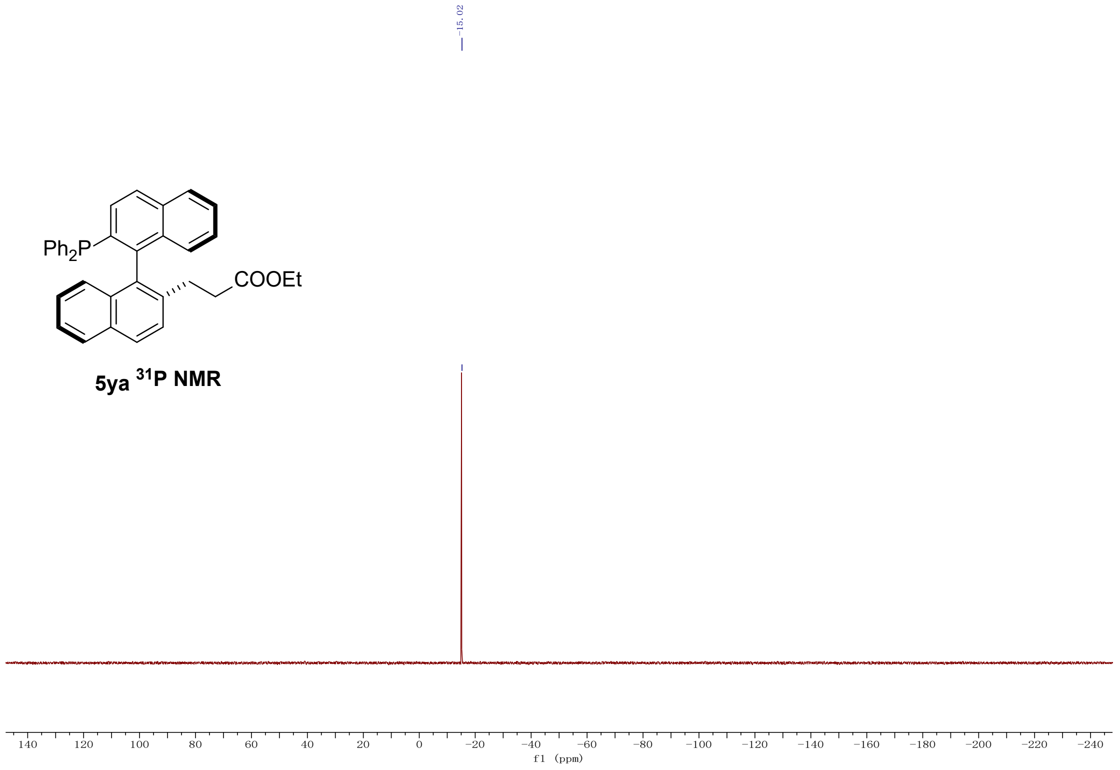

Supplementary Fig. 258. ^{31}P NMR spectra (162 MHz, $CDCl_3$, 25 °C) of **5ya**

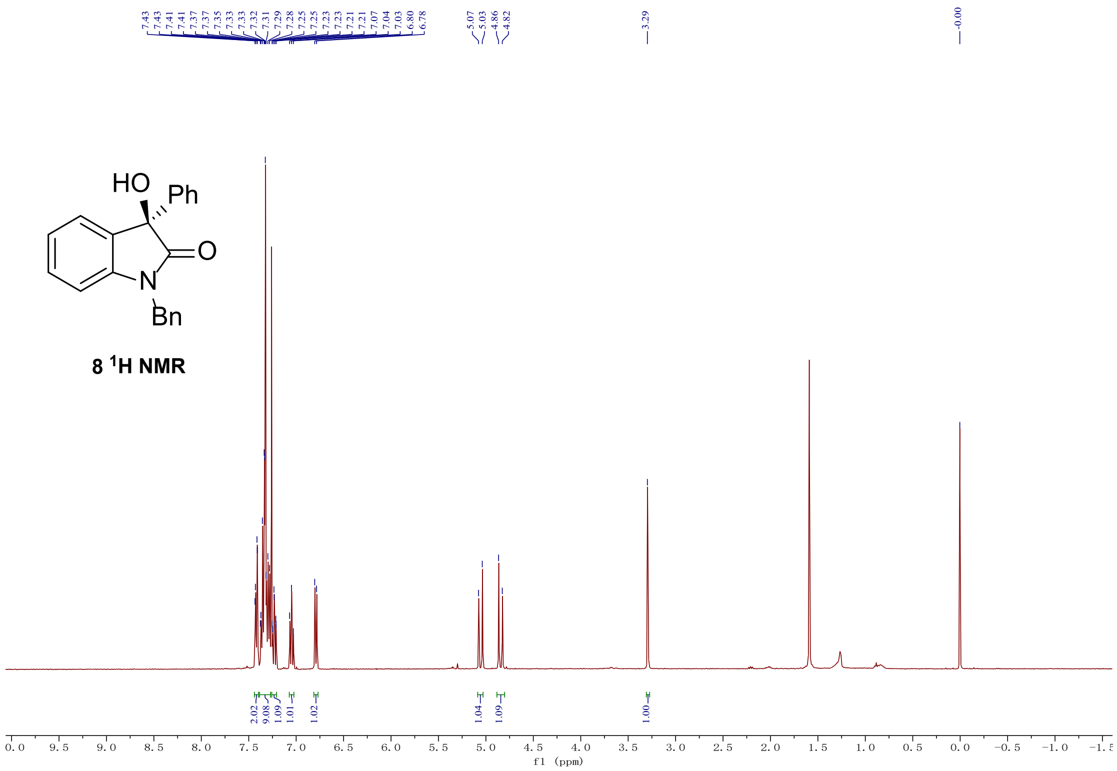

Supplementary Fig. 259 ^{1}H NMR spectra (400 MHz, $CDCl_3$, 25 ºC) of **8**

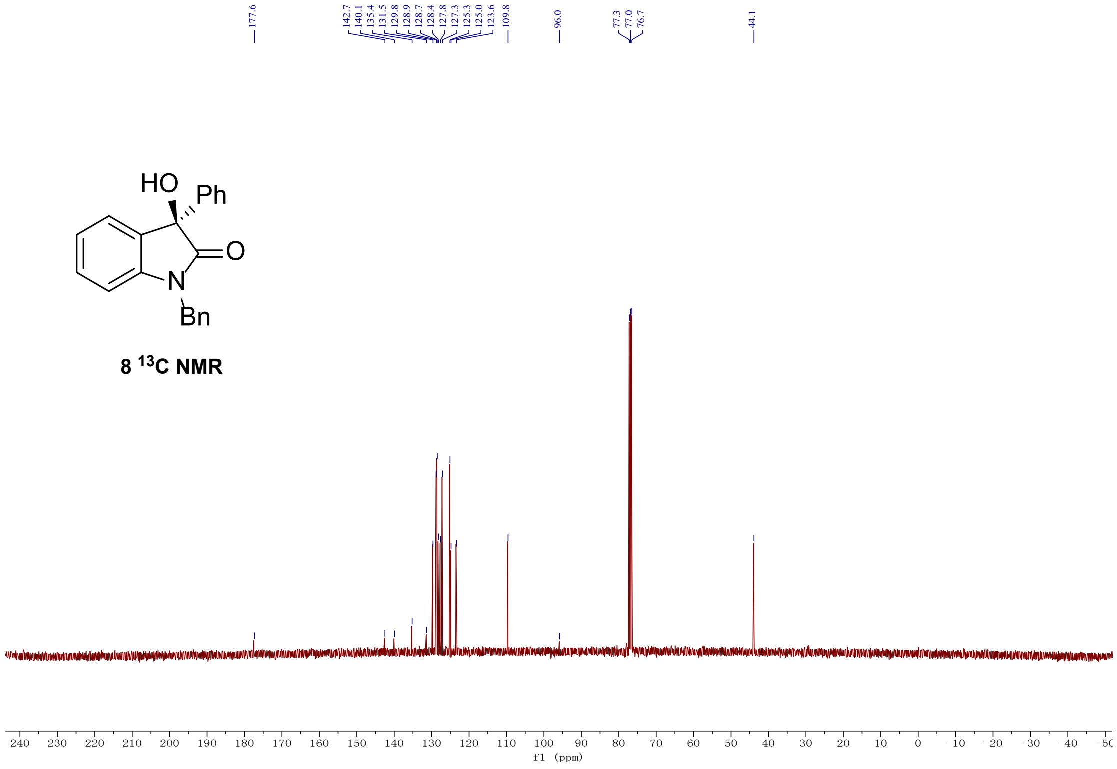

Supplementary Fig. 260. ^{13}C NMR spectra (101 MHz, $CDCl_3$, 25 ºC) of **8**

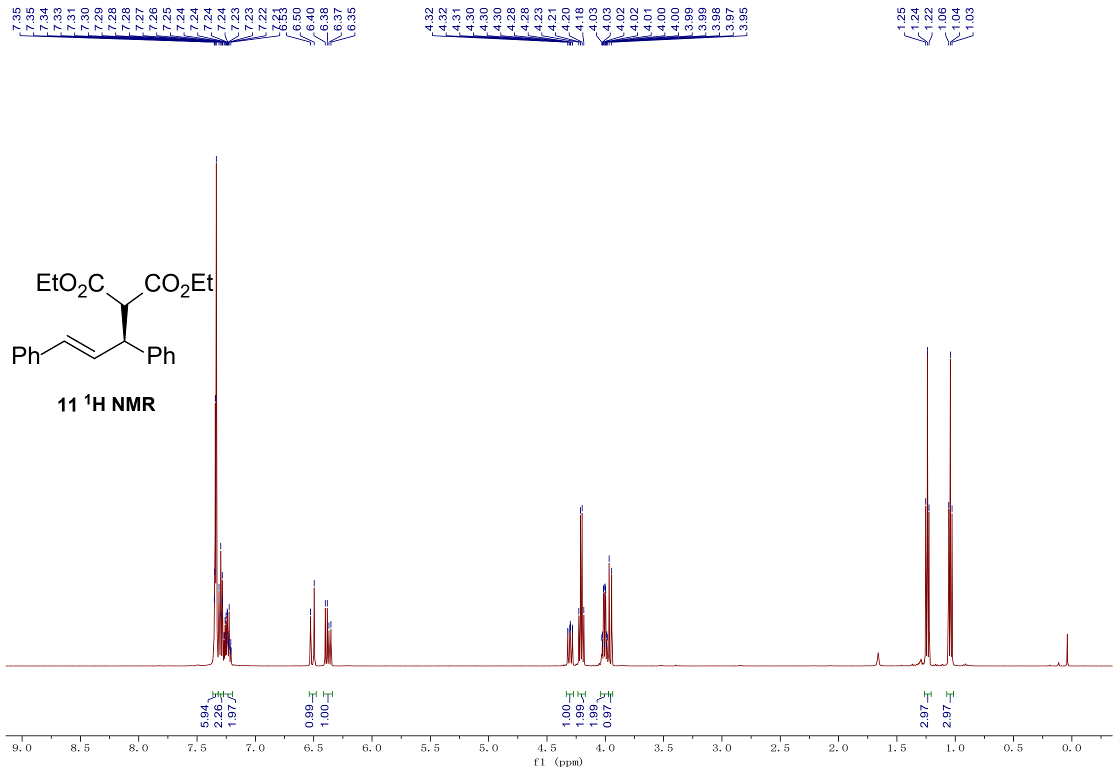

Supplementary Fig. 261 ^{1}H NMR spectra (500 MHz, CDCl3, 25 ºC) of **11**

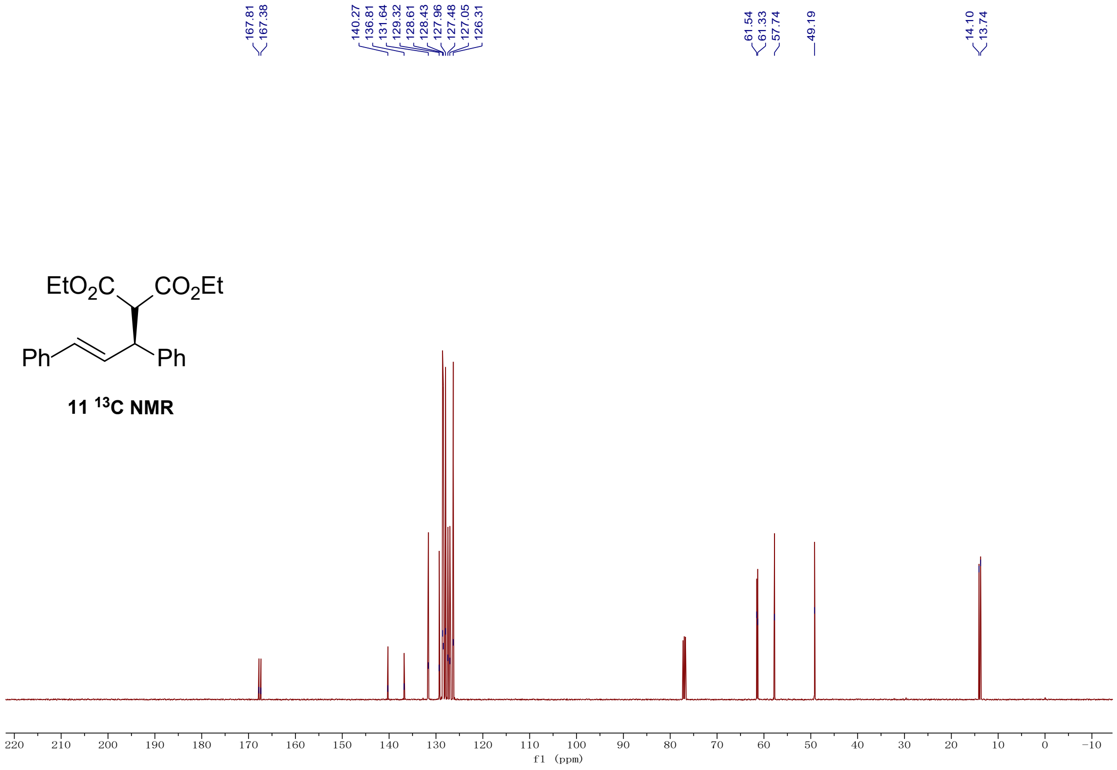

Supplementary Fig. 262. ^{13}C NMR spectra (126 MHz, CDCl3, 25 ºC) of **11**

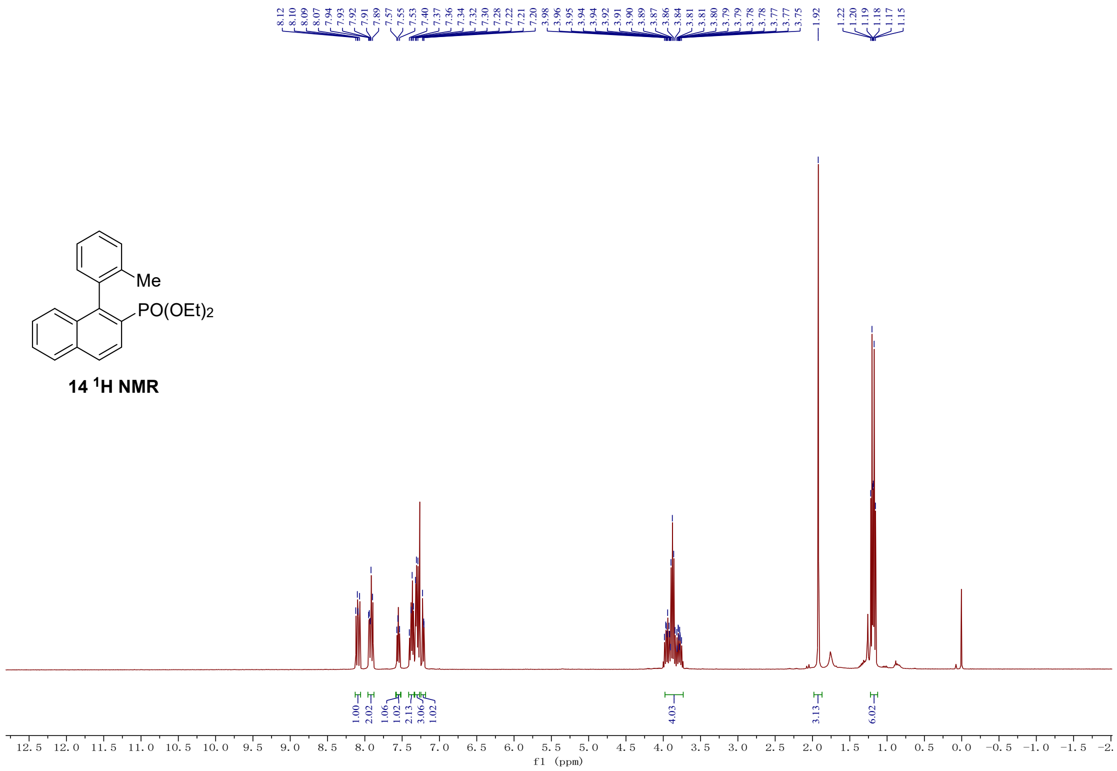

Supplementary Fig. 263 ^{1}H NMR spectra (400 MHz, $CDCl_3$, 25 °C) of **14**

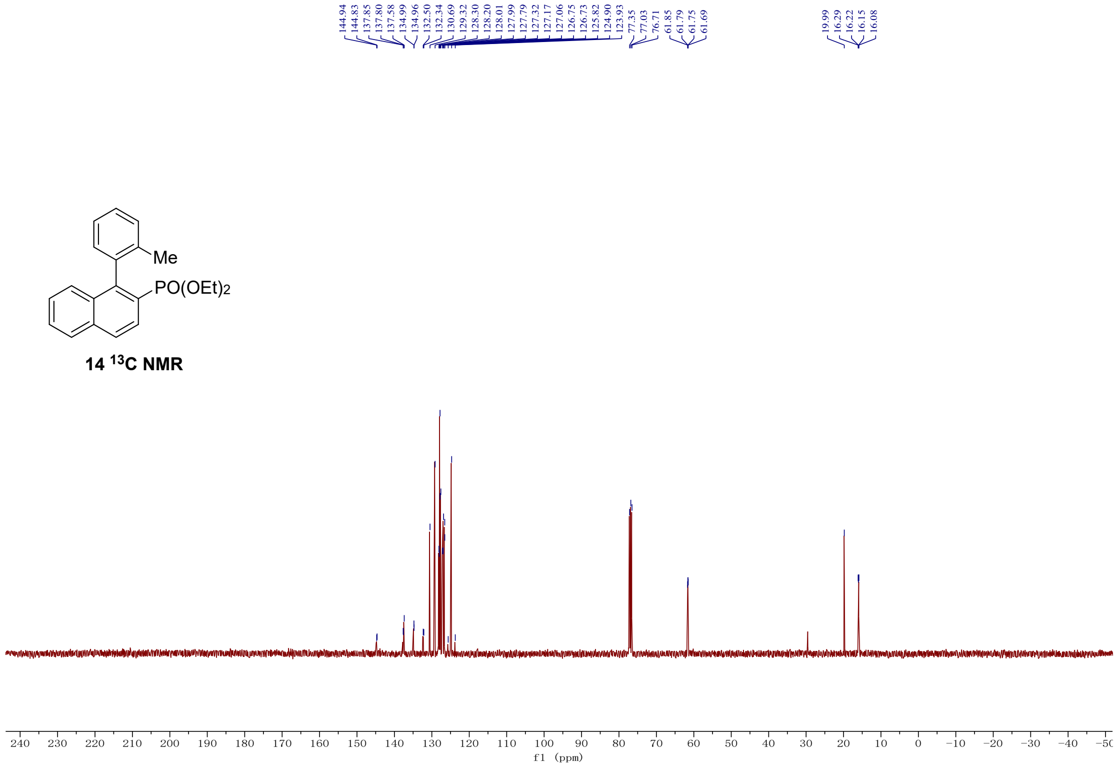

Supplementary Fig. 264. ^{13}C NMR spectra (101 MHz, $CDCl_3$, 25 °C) of **14**

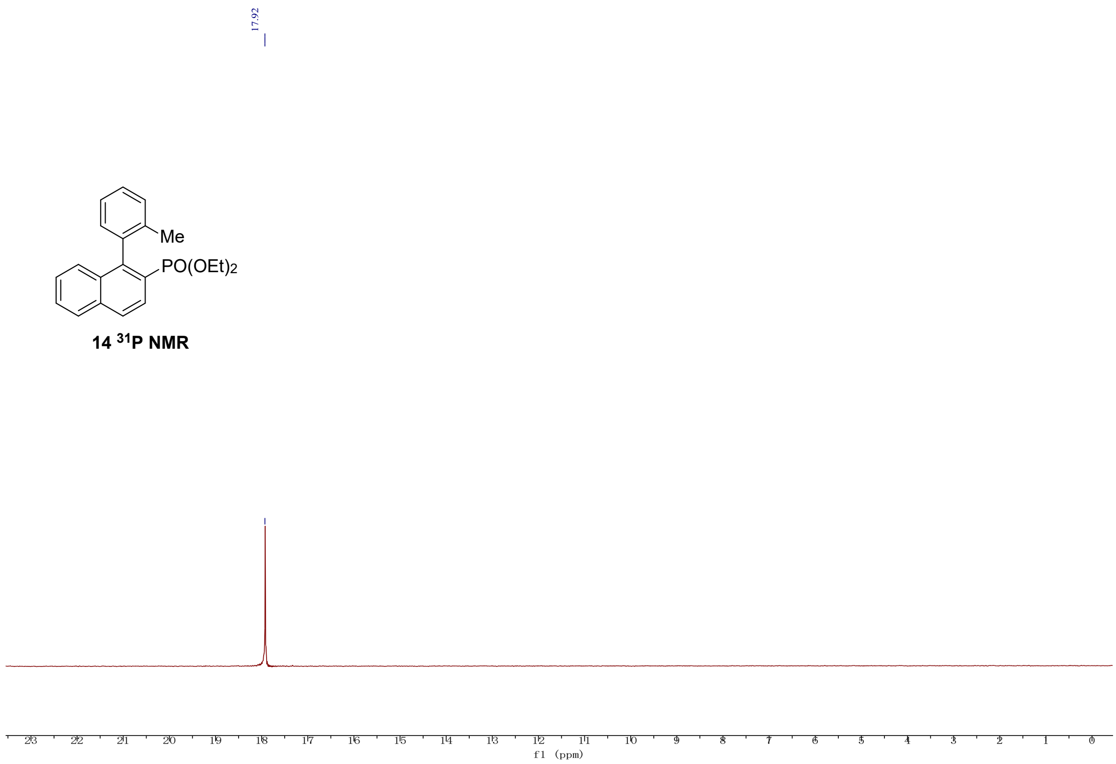

Supplementary Fig. 265. ^{31}P NMR spectra (162 MHz, CDCl$_3$, 25 °C) of **14**

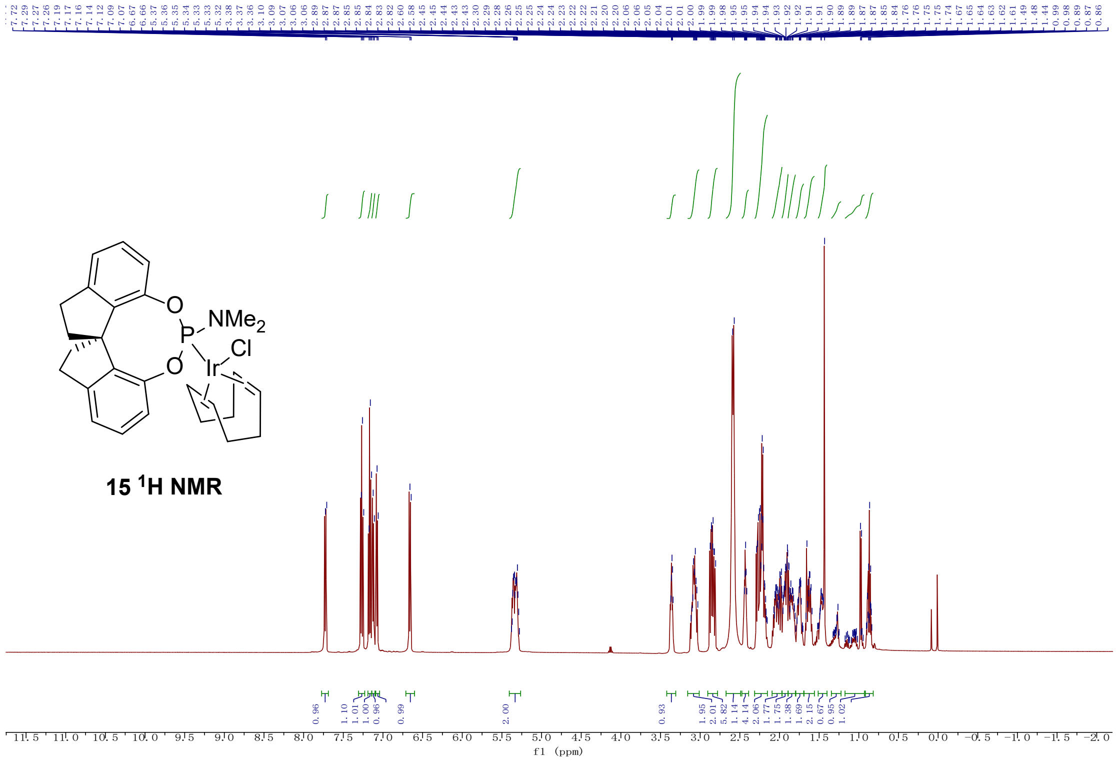

Supplementary Fig. 266 ^{1}H NMR spectra (500 MHz, CDCl$_3$, 25 °C) of **15**

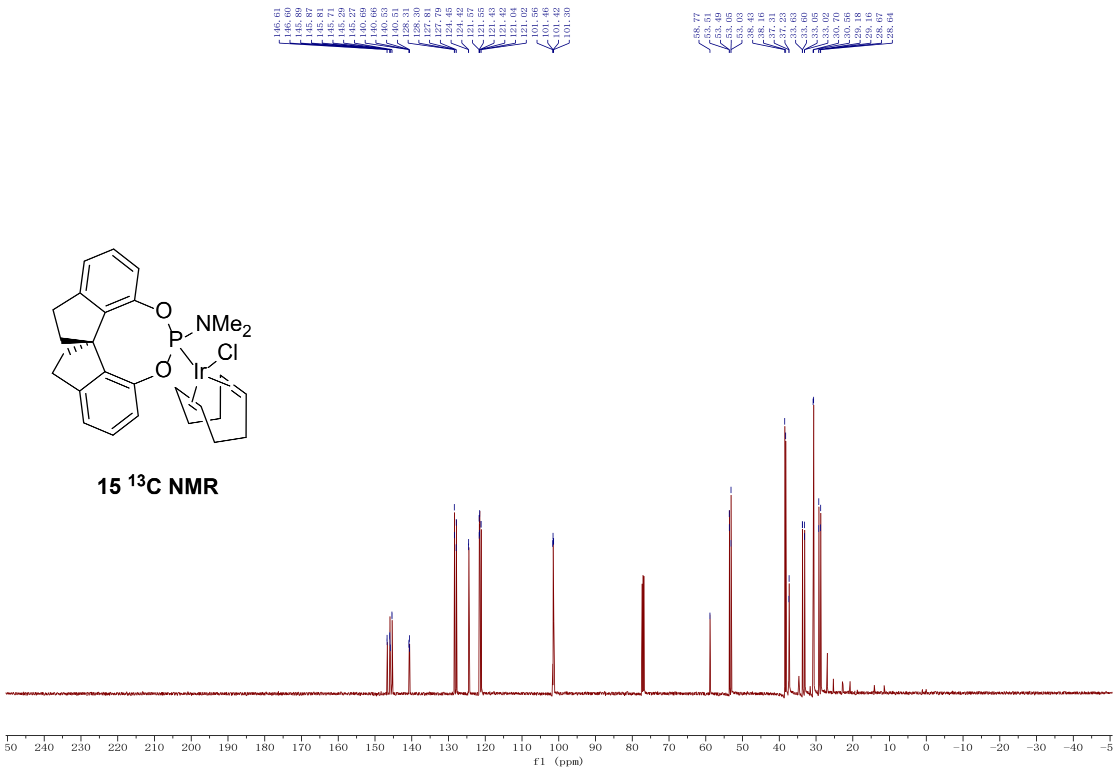

Supplementary Fig. 267. ^{13}C NMR spectra (126 MHz, $CDCl_3$, 25 ºC) of **15**

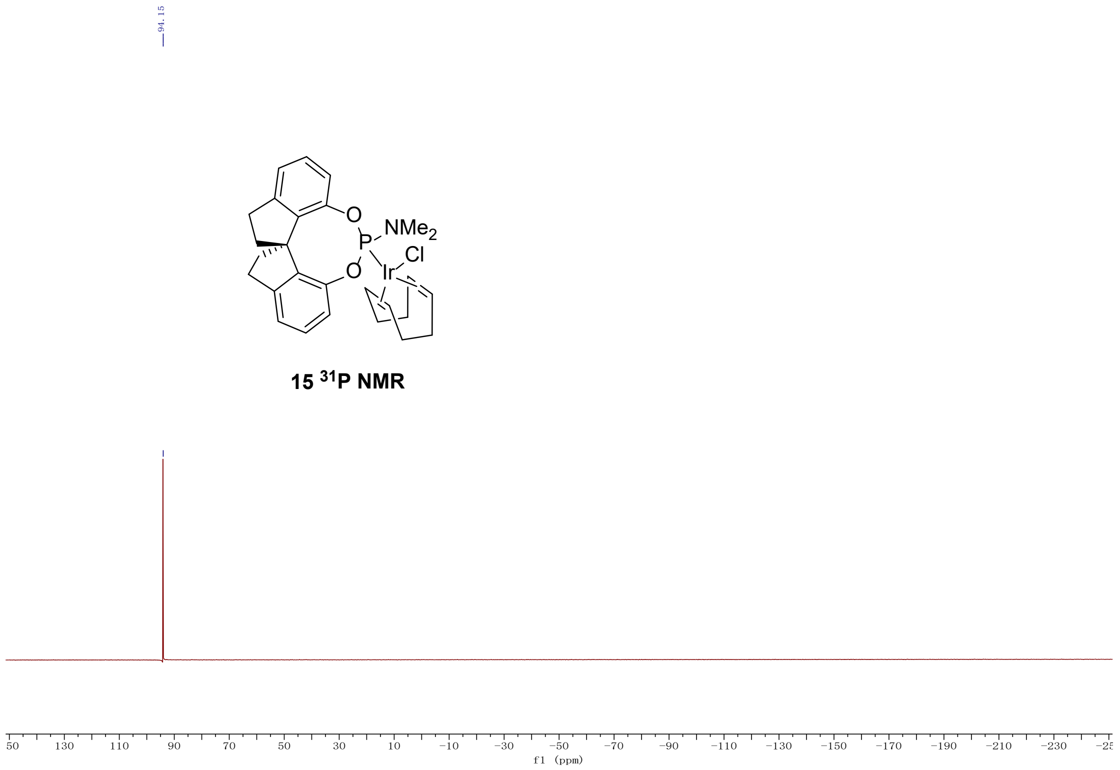

Supplementary Fig. 268. ^{31}P NMR spectra (202 MHz, $CDCl_3$, 25 ºC) of **15**

2.7 Copies HPLC Spectra

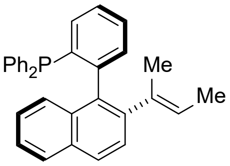

3aa

Chromatogram

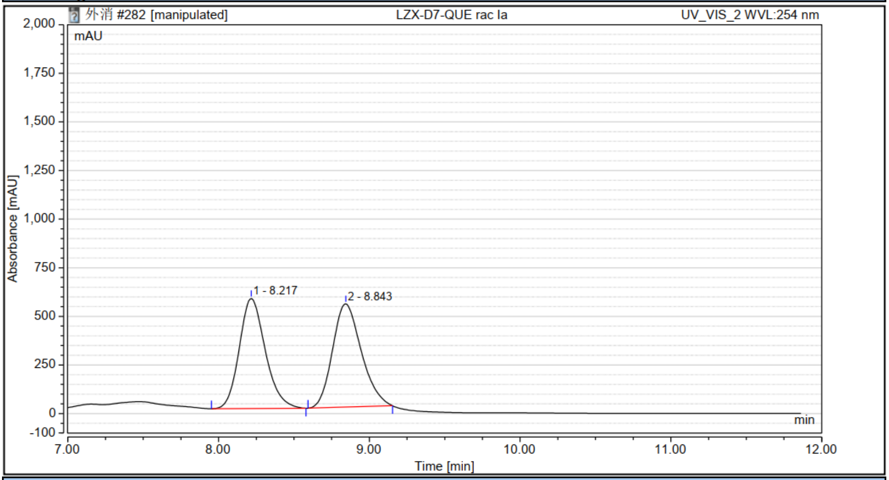

Integration Results

No.	Peak Name	Retention Time min	Area mAU*min	Height mAU	Relative Area %	Relative Height %	Amount n.a.
1		8.217	113.431	566.021	49.11	51.59	n.a.
2		8.843	117.536	531.146	50.89	48.41	n.a.
**Total:**			**230.967**	**1097.168**	**100.00**	**100.00**	

Supplementary Fig. 269 HPLC spectra of rac-**3aa**

Chromatogram

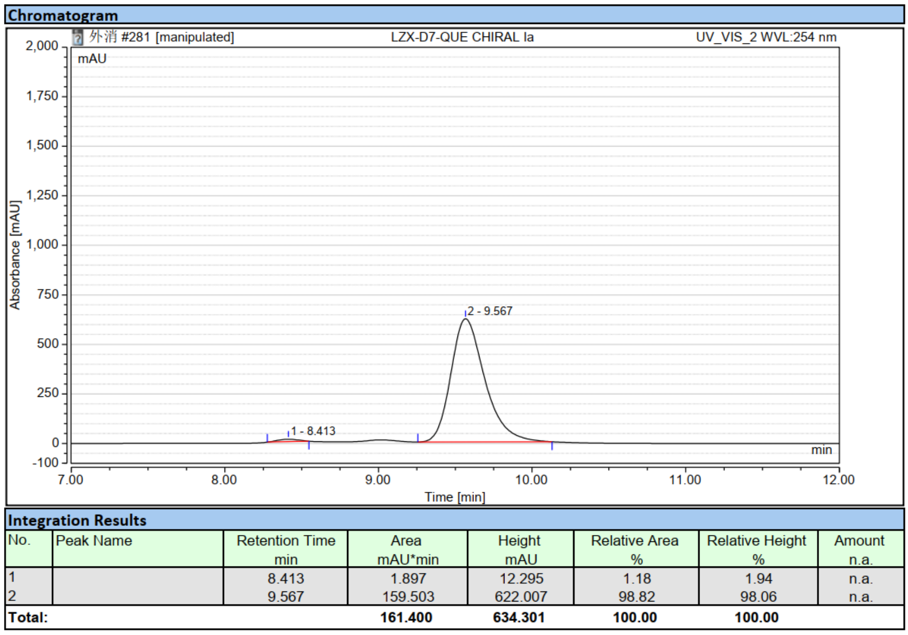

Integration Results

No.	Peak Name	Retention Time min	Area mAU*min	Height mAU	Relative Area %	Relative Height %	Amount n.a.
1		8.413	1.897	12.295	1.18	1.94	n.a.
2		9.567	159.503	622.007	98.82	98.06	n.a.
**Total:**			**161.400**	**634.301**	**100.00**	**100.00**	

Supplementary Fig. 270 HPLC spectra of **3aa**

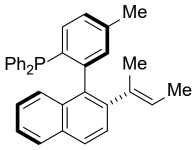

3ba

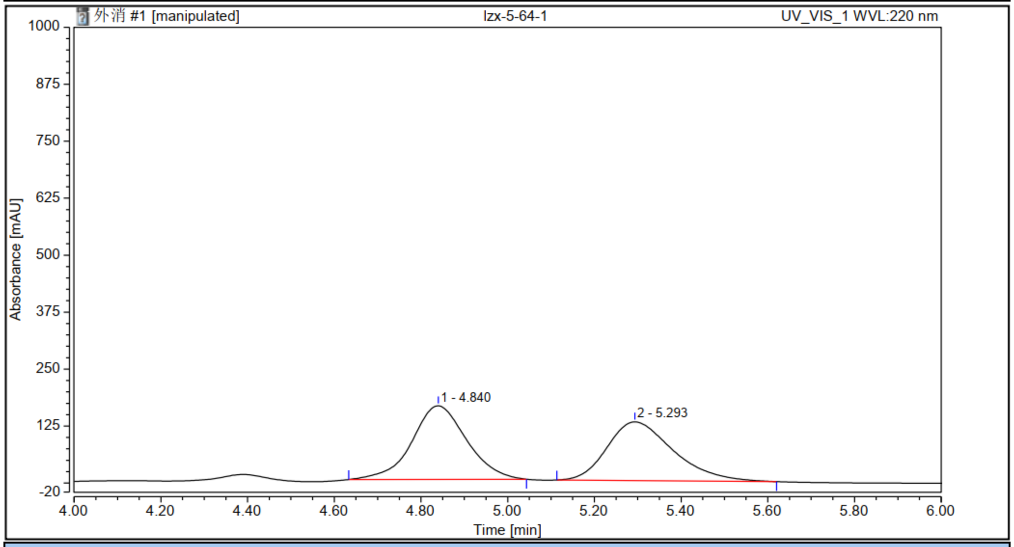

Integration Results

No.	Peak Name	Retention Time min	Area mAU*min	Height mAU	Relative Area %	Relative Height %	Amount n.a.
1		4.840	23.635	161.404	50.90	55.62	n.a.
2		5.293	22.799	128.779	49.10	44.38	n.a.
**Total:**			**46.433**	**290.183**	**100.00**	**100.00**	

Supplementary Fig. 271 HPLC spectra of rac-**3ba**

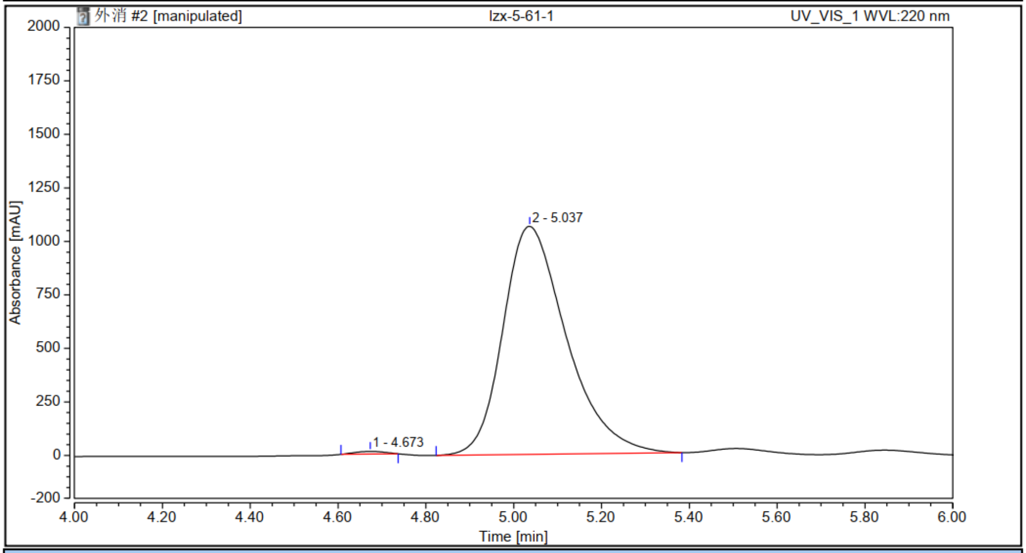

Integration Results

No.	Peak Name	Retention Time min	Area mAU*min	Height mAU	Relative Area %	Relative Height %	Amount n.a.
1		4.673	0.967	12.345	0.54	1.15	n.a.
2		5.037	178.601	1065.059	99.46	98.85	n.a.
**Total:**			**179.568**	**1077.405**	**100.00**	**100.00**	

Supplementary Fig. 272 HPLC spectra of **3ba**

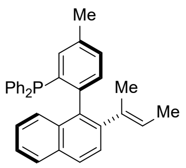

3ca

Chromatogram

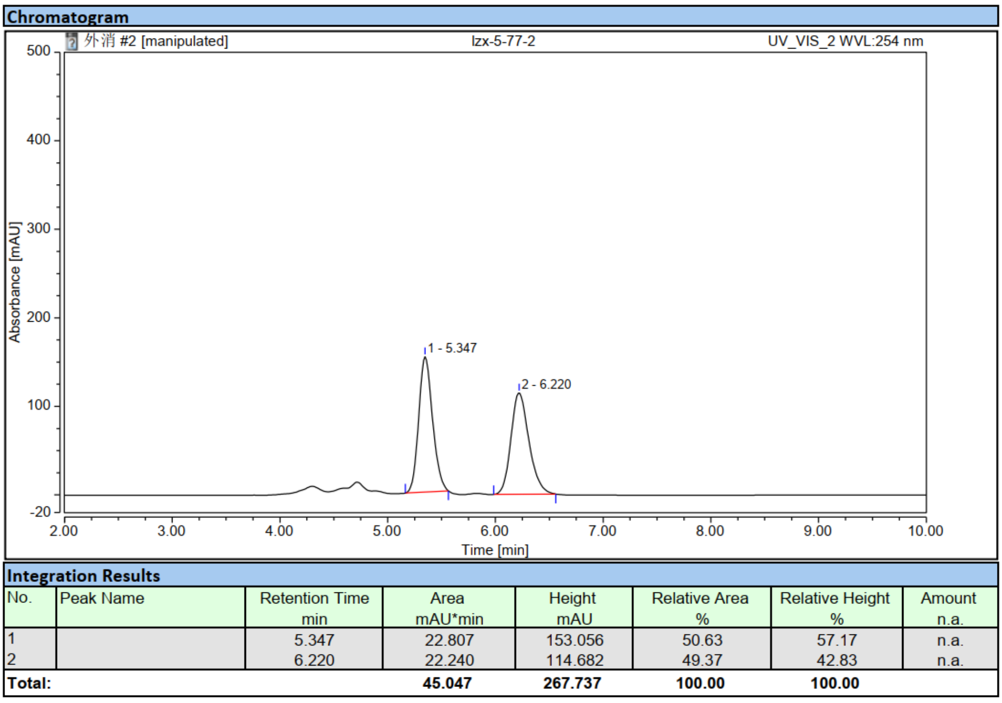

Integration Results

No.	Peak Name	Retention Time<br>min	Area<br>mAU*min	Height<br>mAU	Relative Area<br>%	Relative Height<br>%	Amount<br>n.a.
1		5.347	22.807	153.056	50.63	57.17	n.a.
2		6.220	22.240	114.682	49.37	42.83	n.a.
**Total:**			**45.047**	**267.737**	**100.00**	**100.00**	

Supplementary Fig. 273 HPLC spectra of rac-**3ca**

Chromatogram

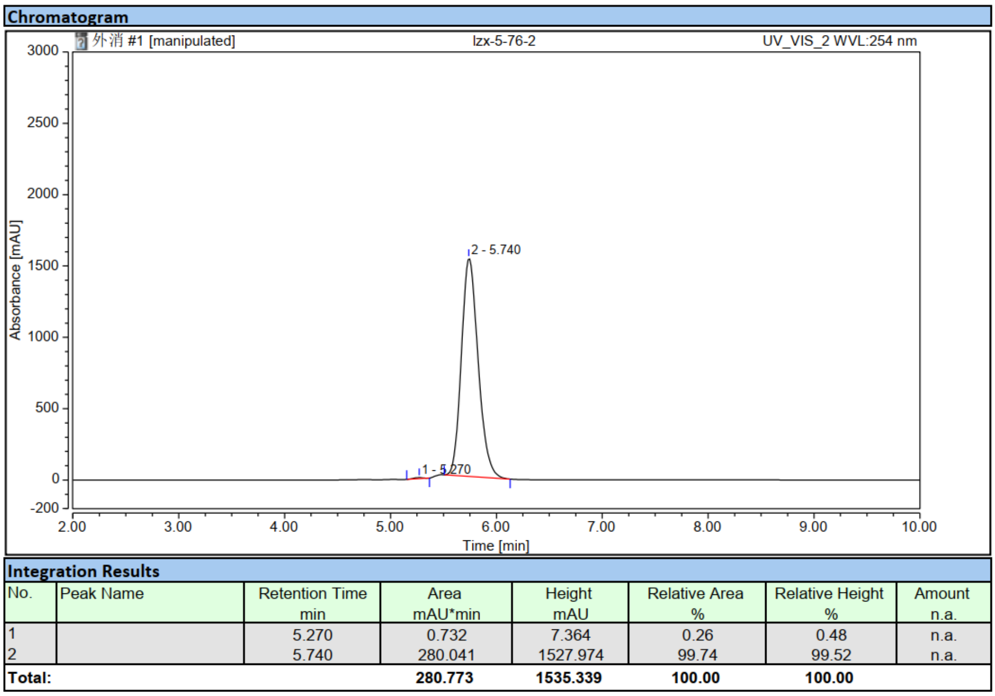

Integration Results

No.	Peak Name	Retention Time<br>min	Area<br>mAU*min	Height<br>mAU	Relative Area<br>%	Relative Height<br>%	Amount<br>n.a.
1		5.270	0.732	7.364	0.26	0.48	n.a.
2		5.740	280.041	1527.974	99.74	99.52	n.a.
**Total:**			**280.773**	**1535.339**	**100.00**	**100.00**	

Supplementary Fig. 274 HPLC spectra of **3ca**

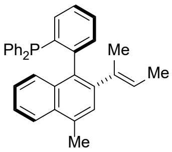

3da

Chromatogram

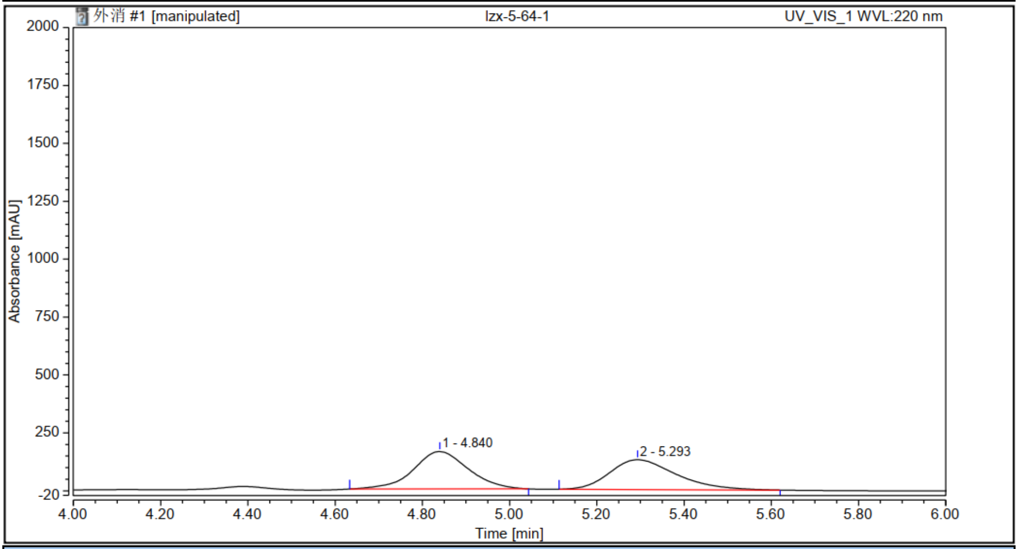

Integration Results

No.	Peak Name	Retention Time min	Area mAU*min	Height mAU	Relative Area %	Relative Height %	Amount n.a.
1		4.840	23.635	161.404	50.90	55.62	n.a.
2		5.293	22.799	128.779	49.10	44.38	n.a.
**Total:**			**46.433**	**290.183**	**100.00**	**100.00**	

Supplementary Fig. 275 HPLC spectra of rac-**3da**

Chromatogram

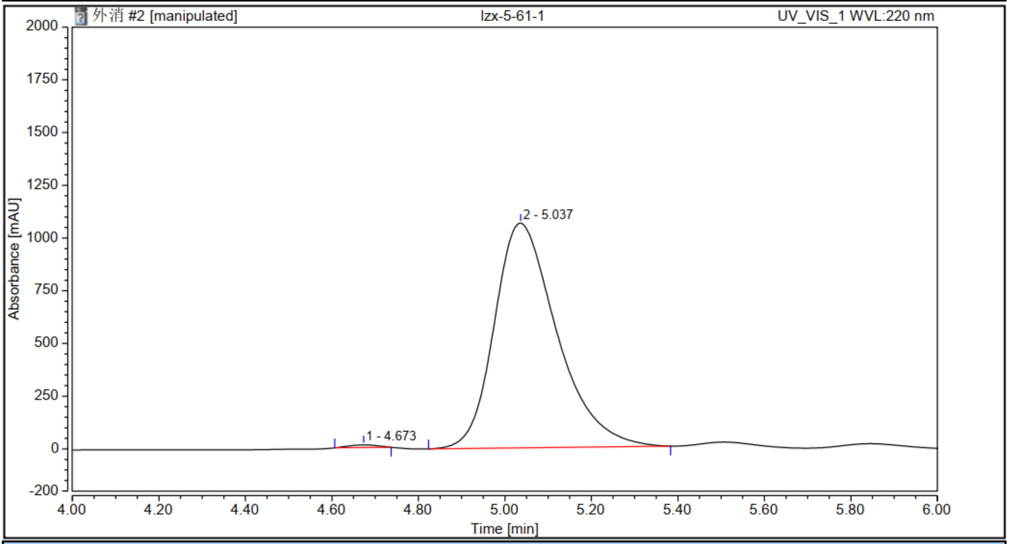

Integration Results

No.	Peak Name	Retention Time min	Area mAU*min	Height mAU	Relative Area %	Relative Height %	Amount n.a.
1		4.673	0.967	12.345	0.54	1.15	n.a.
2		5.037	178.601	1065.059	99.46	98.85	n.a.
**Total:**			**179.568**	**1077.405**	**100.00**	**100.00**	

Supplementary Fig. 276 HPLC spectra of **3da**

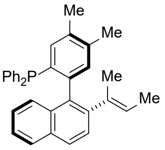

3ea

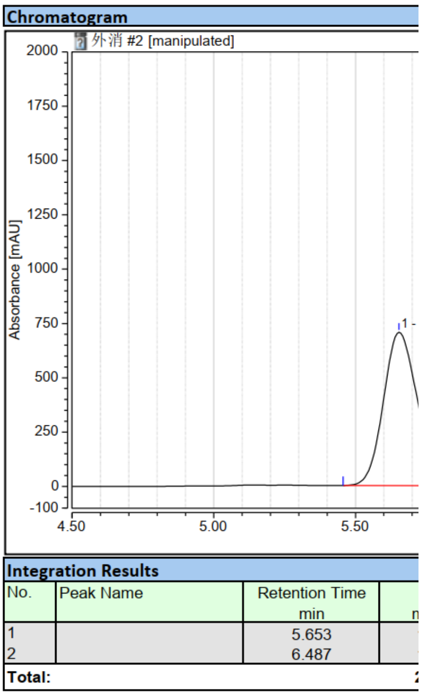

Integration Results

No.	Peak Name	Retention Time min	n[illegible]
1		5.653	
2		6.487	
**Total:**			[illegible]

Supplementary Fig. 277 HPLC spectra of rac-**3ea**

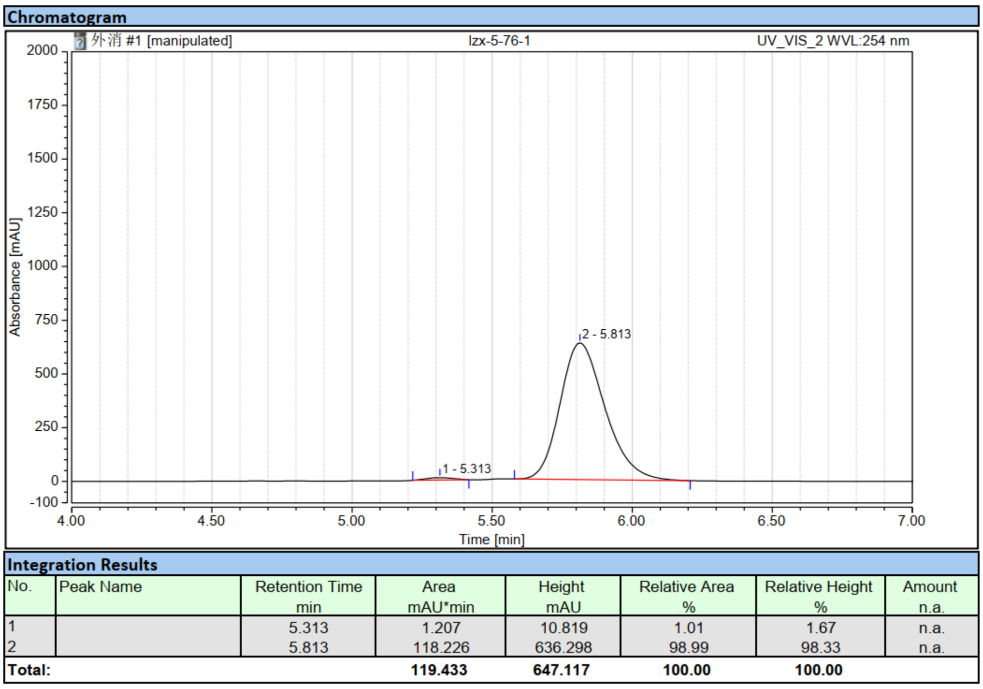

Integration Results

No.	Peak Name	Retention Time min	Area mAU*min	Height mAU	Relative Area %	Relative Height %	Amount n.a.
1		5.313	1.207	10.819	1.01	1.67	n.a.
2		5.813	118.226	636.298	98.99	98.33	n.a.
**Total:**			**119.433**	**647.117**	**100.00**	**100.00**	

Supplementary Fig. 278 HPLC spectra of **3ea**

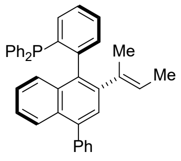

3fa

Chromatogram

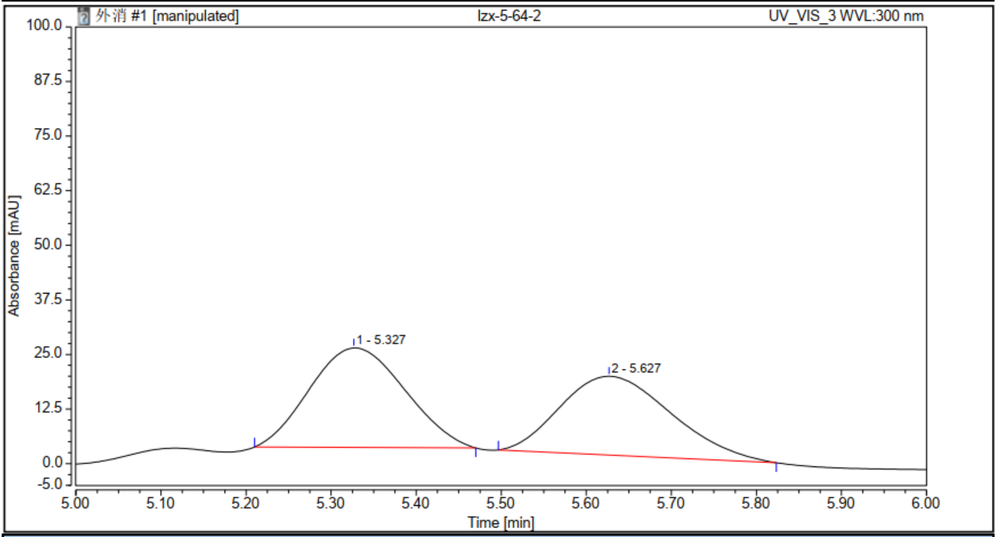

Integration Results

No.	Peak Name	Retention Time min	Area mAU*min	Height mAU	Relative Area %	Relative Height %	Amount n.a.
1		5.327	2.948	22.770	51.97	55.82	n.a.
2		5.627	2.725	18.025	48.03	44.18	n.a.
**Total:**			**5.673**	**40.795**	**100.00**	**100.00**	

Supplementary Fig. 279 HPLC spectra of rac-**3fa**

Chromatogram

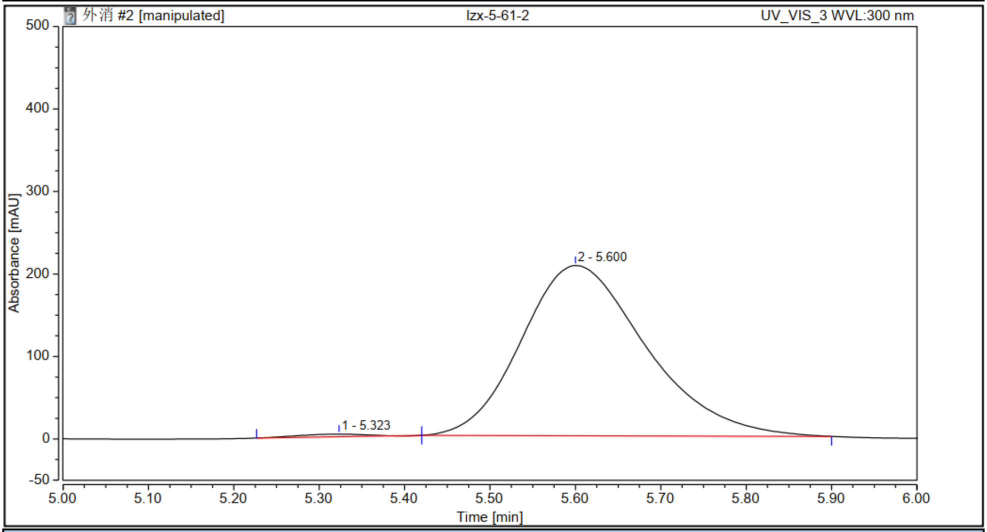

Integration Results

No.	Peak Name	Retention Time min	Area mAU*min	Height mAU	Relative Area %	Relative Height %	Amount n.a.
1		5.323	0.282	3.099	0.80	1.48	n.a.
2		5.600	34.985	206.473	99.20	98.52	n.a.
**Total:**			**35.267**	**209.572**	**100.00**	**100.00**	

Supplementary Fig. 280 HPLC spectra of **3fa**

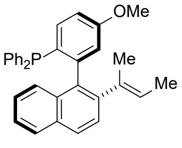

3ga

Chromatogram

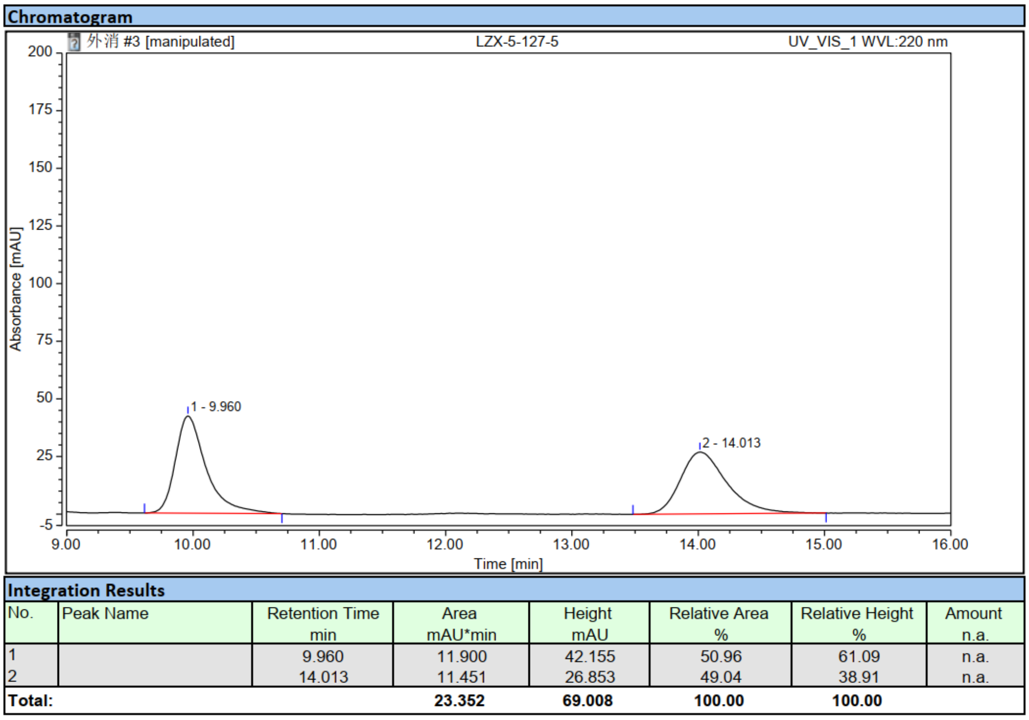

Integration Results

No.	Peak Name	Retention Time min	Area mAU*min	Height mAU	Relative Area %	Relative Height %	Amount n.a.
1		9.960	11.900	42.155	50.96	61.09	n.a.
2		14.013	11.451	26.853	49.04	38.91	n.a.
**Total:**			**23.352**	**69.008**	**100.00**	**100.00**	

Supplementary Fig. 281 HPLC spectra of rac-**3ga**

Chromatogram

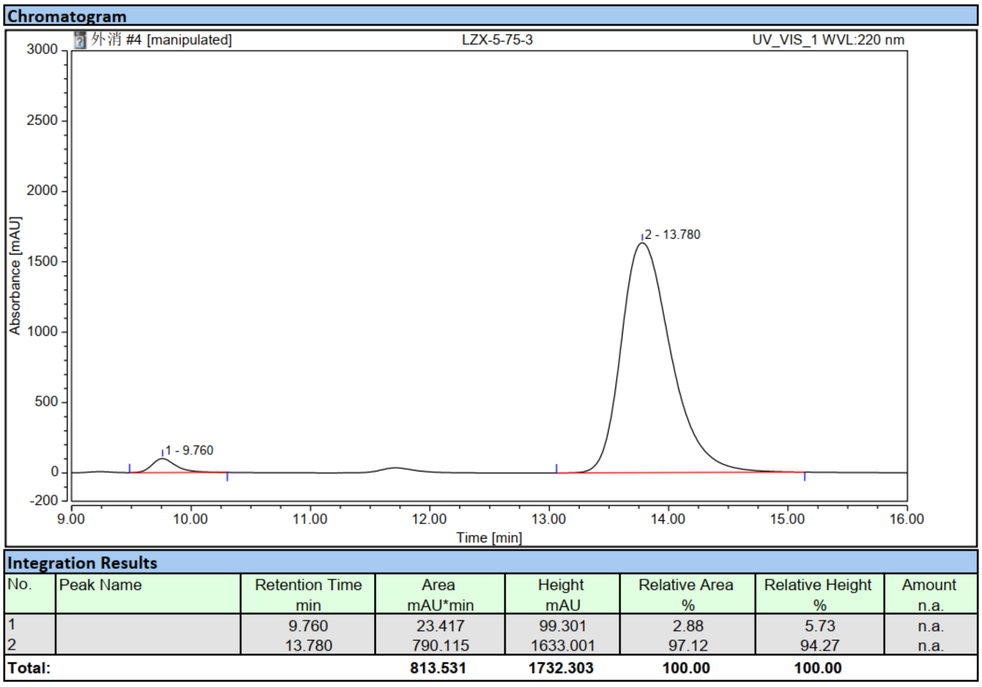

Integration Results

No.	Peak Name	Retention Time min	Area mAU*min	Height mAU	Relative Area %	Relative Height %	Amount n.a.
1		9.760	23.417	99.301	2.88	5.73	n.a.
2		13.780	790.115	1633.001	97.12	94.27	n.a.
**Total:**			**813.531**	**1732.303**	**100.00**	**100.00**	

Supplementary Fig. 282 HPLC spectra of **3ga**

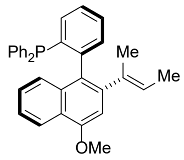

3ha

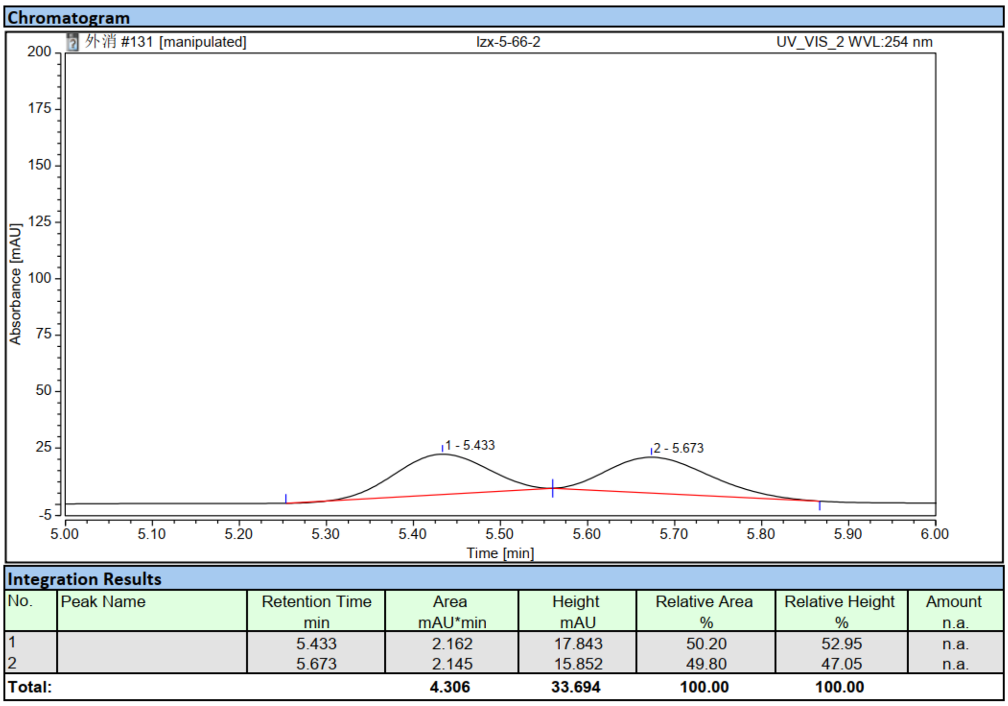

Integration Results

No.	Peak Name	Retention Time min	Area mAU*min	Height mAU	Relative Area %	Relative Height %	Amount n.a.
1		5.433	2.162	17.843	50.20	52.95	n.a.
2		5.673	2.145	15.852	49.80	47.05	n.a.
**Total:**			**4.306**	**33.694**	**100.00**	**100.00**	

Supplementary Fig. 283 HPLC spectra of rac-**3ha**

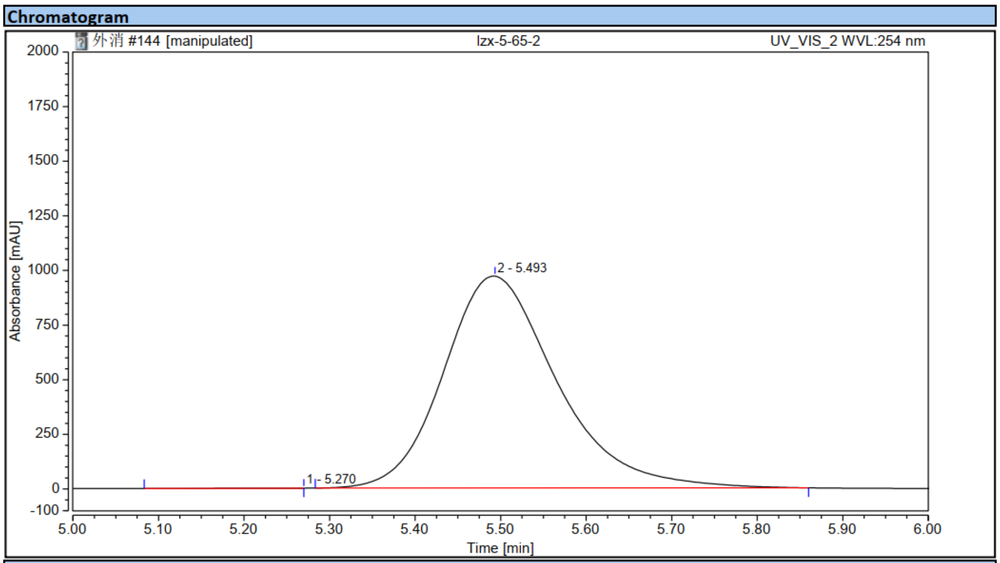

Integration Results

No.	Peak Name	Retention Time min	Area mAU*min	Height mAU	Relative Area %	Relative Height %	Amount n.a.
1		5.270	0.001	0.000	0.00	0.00	n.a.
2		5.493	148.845	969.732	100.00	100.00	n.a.
**Total:**			**148.847**	**969.732**	**100.00**	**100.00**	

Supplementary Fig. 284 HPLC spectra of **3ha**

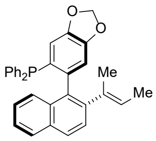

3ia

Chromatogram

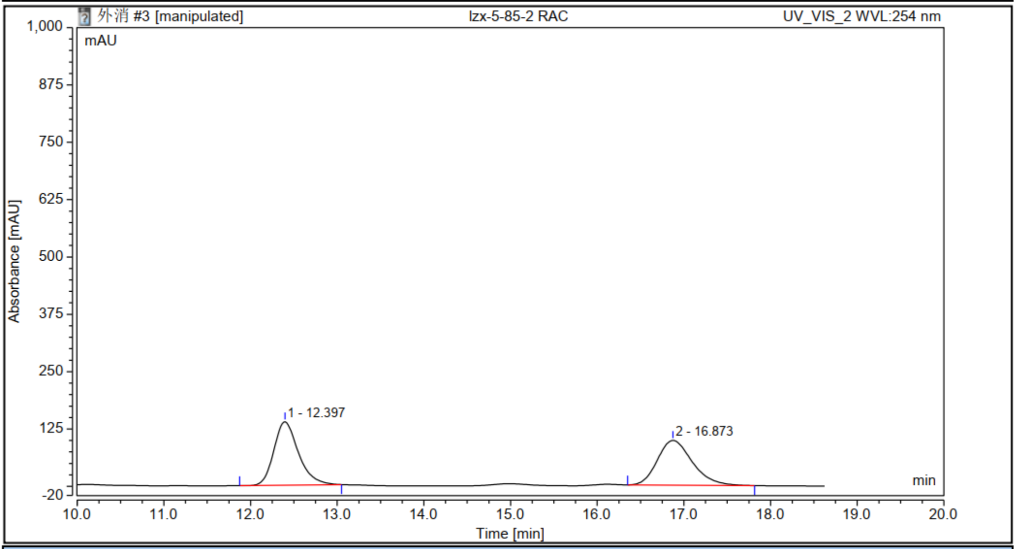

Integration Results

No.	Peak Name	Retention Time min	Area mAU*min	Height mAU	Relative Area %	Relative Height %	Amount n.a.
1		12.397	45.582	138.216	49.99	58.63	n.a.
2		16.873	45.609	97.547	50.01	41.37	n.a.
**Total:**			**91.191**	**235.763**	**100.00**	**100.00**	

Supplementary Fig. 285 HPLC spectra of rac-**3ia**

Chromatogram

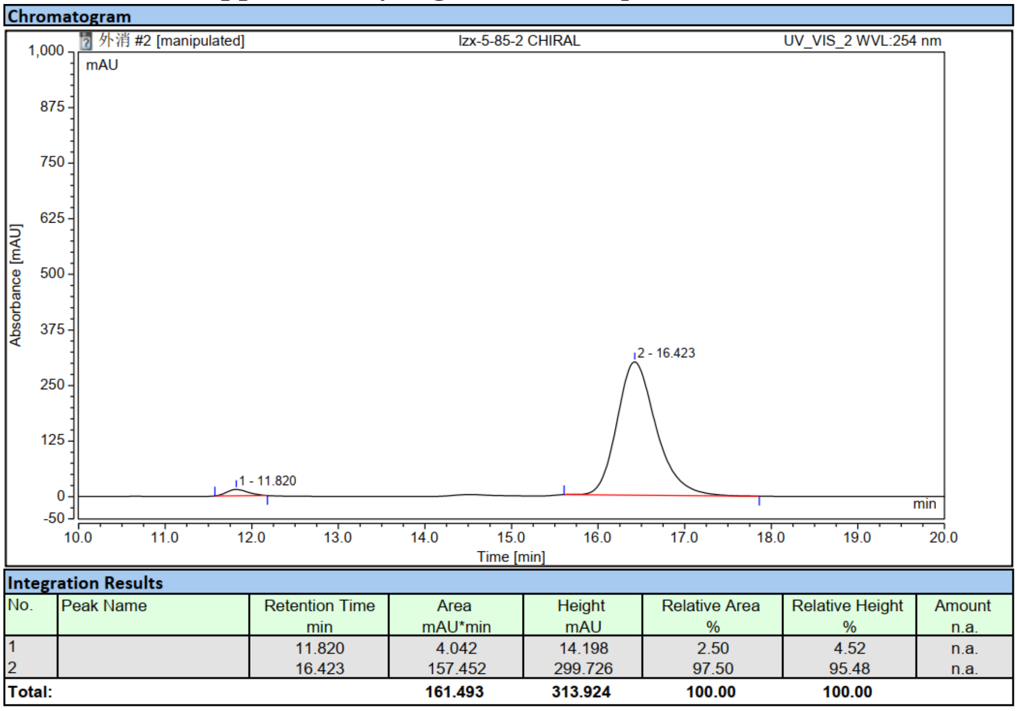

Integration Results

No.	Peak Name	Retention Time min	Area mAU*min	Height mAU	Relative Area %	Relative Height %	Amount n.a.
1		11.820	4.042	14.198	2.50	4.52	n.a.
2		16.423	157.452	299.726	97.50	95.48	n.a.
**Total:**			**161.493**	**313.924**	**100.00**	**100.00**	

Supplementary Fig. 286 HPLC spectra of **3ia**

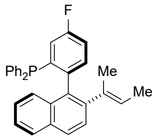

3ja

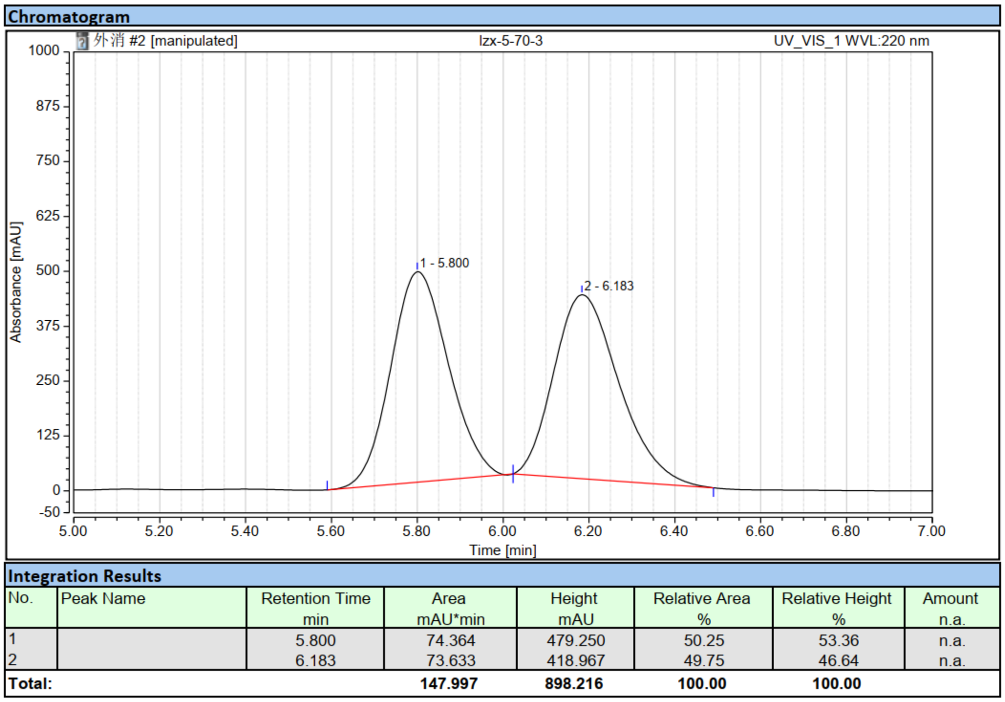

Integration Results

No.	Peak Name	Retention Time min	Area mAU*min	Height mAU	Relative Area %	Relative Height %	Amount n.a.
1		5.800	74.364	479.250	50.25	53.36	n.a.
2		6.183	73.633	418.967	49.75	46.64	n.a.
**Total:**			**147.997**	**898.216**	**100.00**	**100.00**	

Supplementary Fig. 287 HPLC spectra of rac-**3ja**

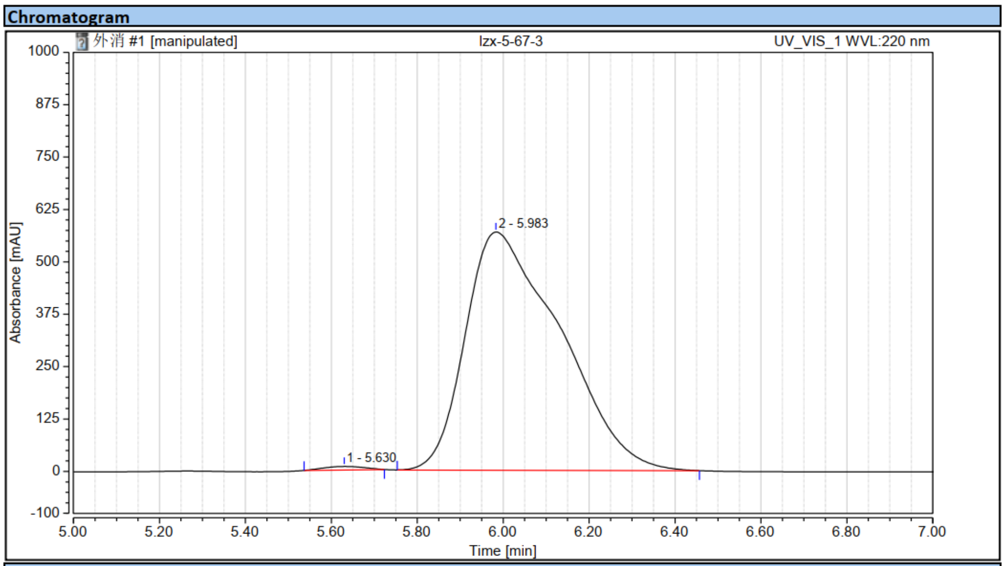

Integration Results

No.	Peak Name	Retention Time min	Area mAU*min	Height mAU	Relative Area %	Relative Height %	Amount n.a.
1		5.630	0.899	8.286	0.61	1.44	n.a.
2		5.983	145.723	568.293	99.39	98.56	n.a.
**Total:**			**146.622**	**576.578**	**100.00**	**100.00**	

Supplementary Fig. 288 HPLC spectra of **3ka**

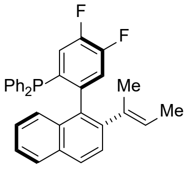

3ka

Chromatogram

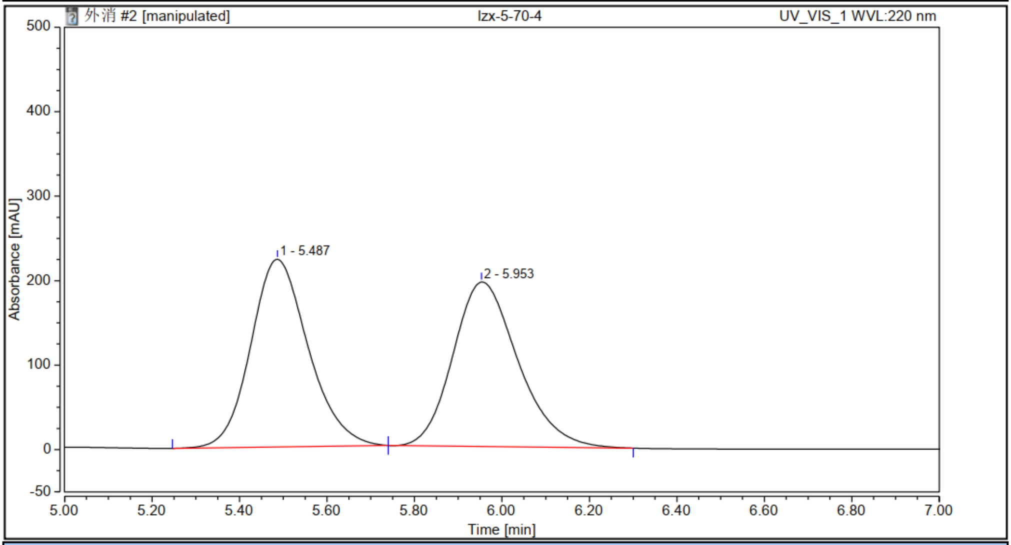

Integration Results

No.	Peak Name	Retention Time<br>min	Area<br>mAU*min	Height<br>mAU	Relative Area<br>%	Relative Height<br>%	Amount<br>n.a.
1		5.487	33.874	222.132	50.21	53.28	n.a.
2		5.953	33.594	194.769	49.79	46.72	n.a.
**Total:**			**67.468**	**416.901**	**100.00**	**100.00**	

Supplementary Fig. 289 HPLC spectra of rac-**3ka**

Chromatogram

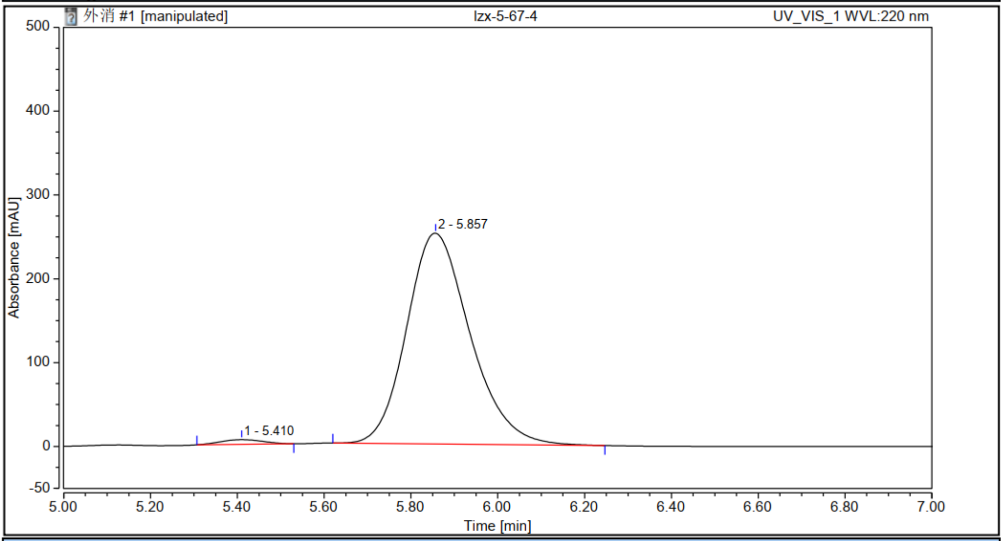

Integration Results

No.	Peak Name	Retention Time<br>min	Area<br>mAU*min	Height<br>mAU	Relative Area<br>%	Relative Height<br>%	Amount<br>n.a.
1		5.410	0.630	5.566	1.46	2.17	n.a.
2		5.857	42.597	251.527	98.54	97.83	n.a.
**Total:**			**43.227**	**257.093**	**100.00**	**100.00**	

Supplementary Fig. 290 HPLC spectra of **3ka**

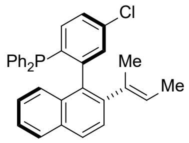

3la

Chromatogram

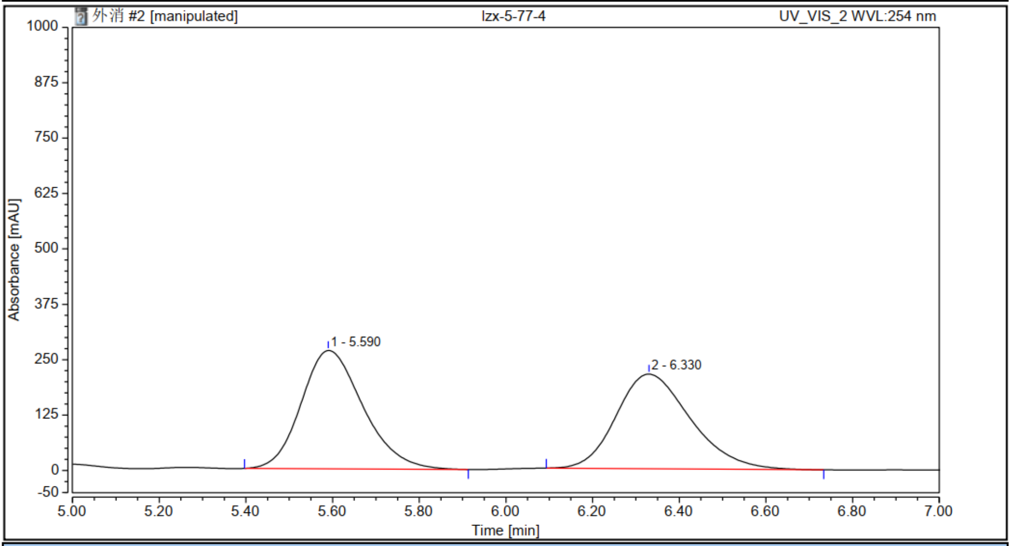

Integration Results

No.	Peak Name	Retention Time<br>min	Area<br>mAU*min	Height<br>mAU	Relative Area<br>%	Relative Height<br>%	Amount<br>n.a.
1		5.590	44.163	267.147	50.96	55.56	n.a.
2		6.330	42.507	213.666	49.04	44.44	n.a.
**Total:**			**86.670**	**480.813**	**100.00**	**100.00**	

Supplementary Fig. 291 HPLC spectra of rac-**3la**

Chromatogram

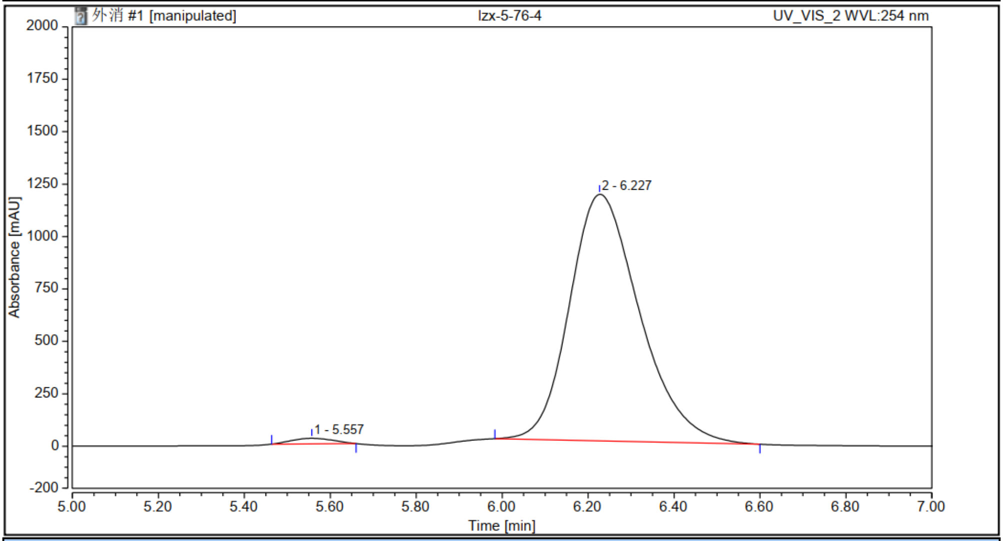

Integration Results

No.	Peak Name	Retention Time<br>min	Area<br>mAU*min	Height<br>mAU	Relative Area<br>%	Relative Height<br>%	Amount<br>n.a.
1		5.557	2.957	26.433	1.31	2.20	n.a.
2		6.227	222.296	1176.179	98.69	97.80	n.a.
**Total:**			**225.253**	**1202.612**	**100.00**	**100.00**	

Supplementary Fig. 292 HPLC spectra of **3la**

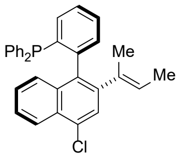

3ma

Chromatogram

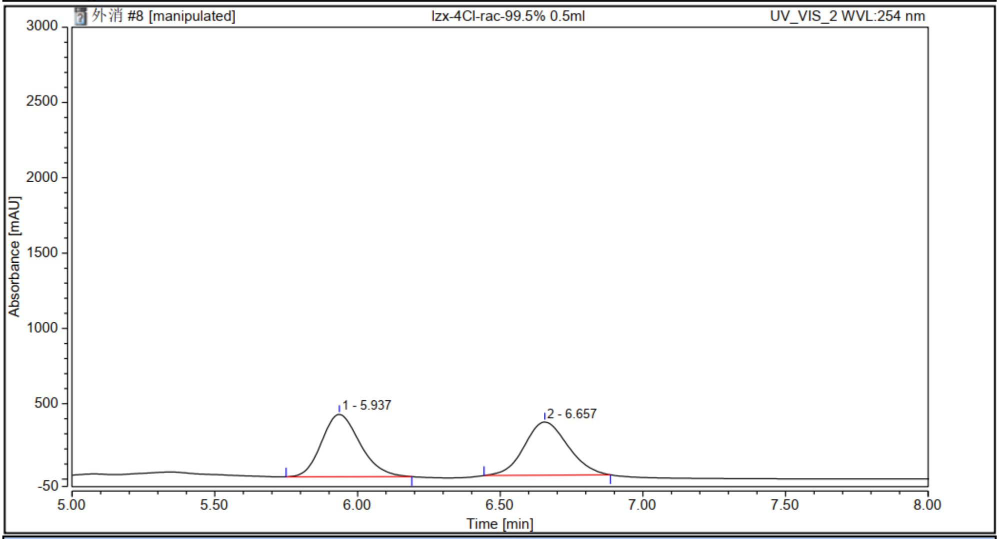

Integration Results

No.	Peak Name	Retention Time min	Area mAU*min	Height mAU	Relative Area %	Relative Height %	Amount n.a.
1		5.937	64.613	413.844	50.81	53.96	n.a.
2		6.657	62.549	353.108	49.19	46.04	n.a.
**Total:**			**127.162**	**766.952**	**100.00**	**100.00**	

Supplementary Fig. 293 HPLC spectra of rac-**3ma**

Chromatogram

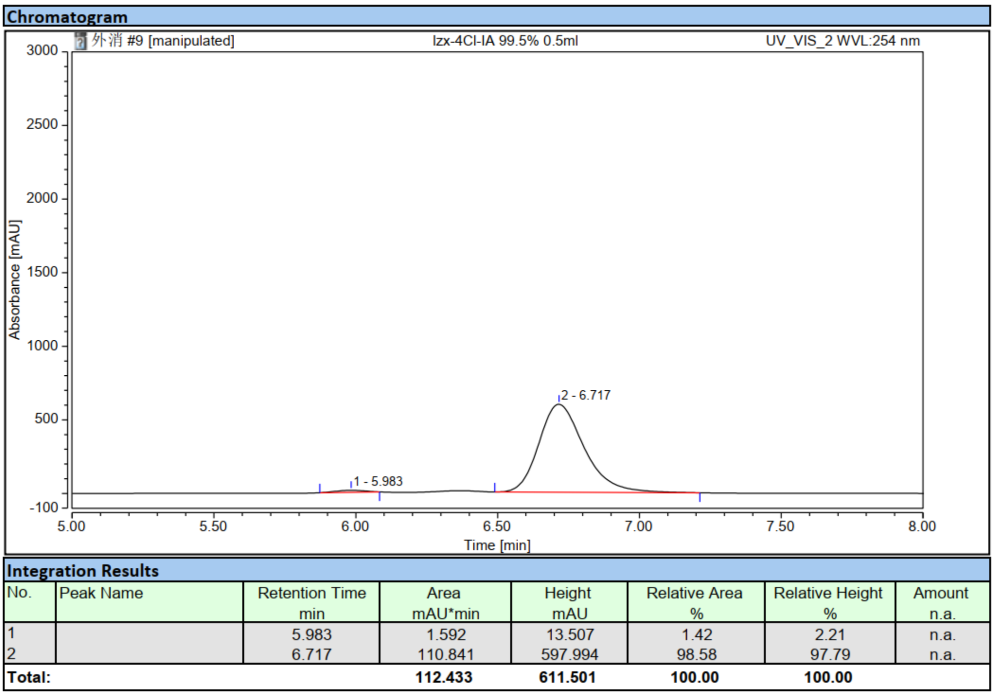

Integration Results

No.	Peak Name	Retention Time min	Area mAU*min	Height mAU	Relative Area %	Relative Height %	Amount n.a.
1		5.983	1.592	13.507	1.42	2.21	n.a.
2		6.717	110.841	597.994	98.58	97.79	n.a.
**Total:**			**112.433**	**611.501**	**100.00**	**100.00**	

Supplementary Fig. 294 HPLC spectra of **3ma**

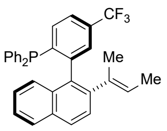

3na

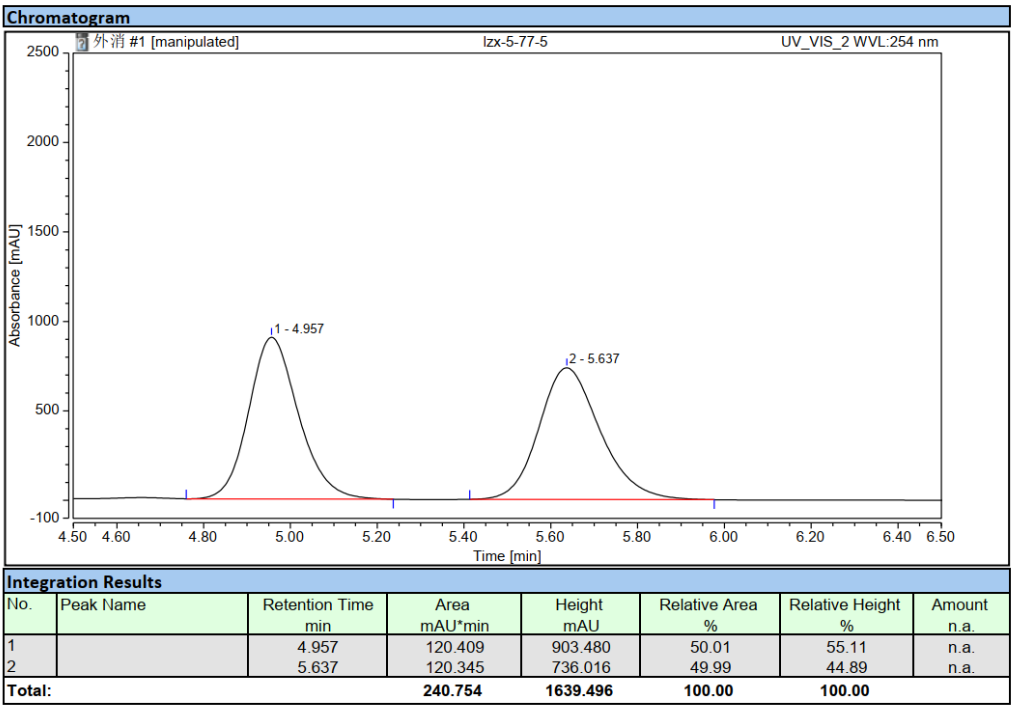

Integration Results

No.	Peak Name	Retention Time<br>min	Area<br>mAU*min	Height<br>mAU	Relative Area<br>%	Relative Height<br>%	Amount<br>n.a.
1		4.957	120.409	903.480	50.01	55.11	n.a.
2		5.637	120.345	736.016	49.99	44.89	n.a.
**Total:**			**240.754**	**1639.496**	**100.00**	**100.00**	

Supplementary Fig. 295 HPLC spectra of rac-**3na**

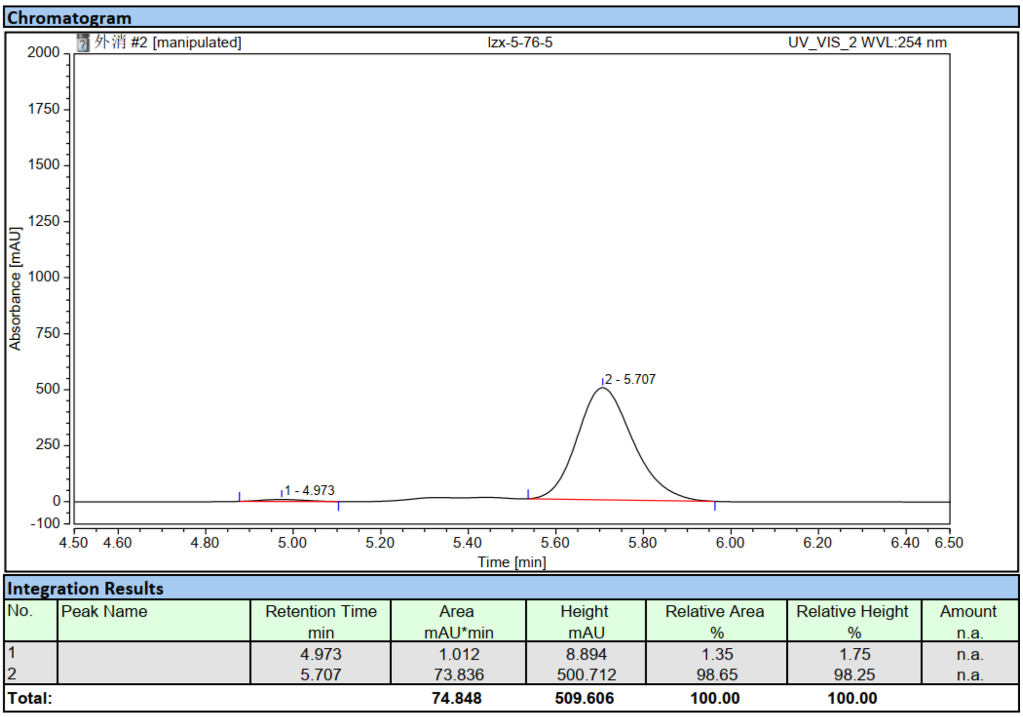

Integration Results

No.	Peak Name	Retention Time<br>min	Area<br>mAU*min	Height<br>mAU	Relative Area<br>%	Relative Height<br>%	Amount<br>n.a.
1		4.973	1.012	8.894	1.35	1.75	n.a.
2		5.707	73.836	500.712	98.65	98.25	n.a.
**Total:**			**74.848**	**509.606**	**100.00**	**100.00**	

Supplementary Fig. 296 HPLC spectra of **3na**

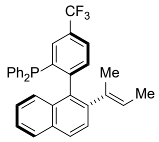

3oa

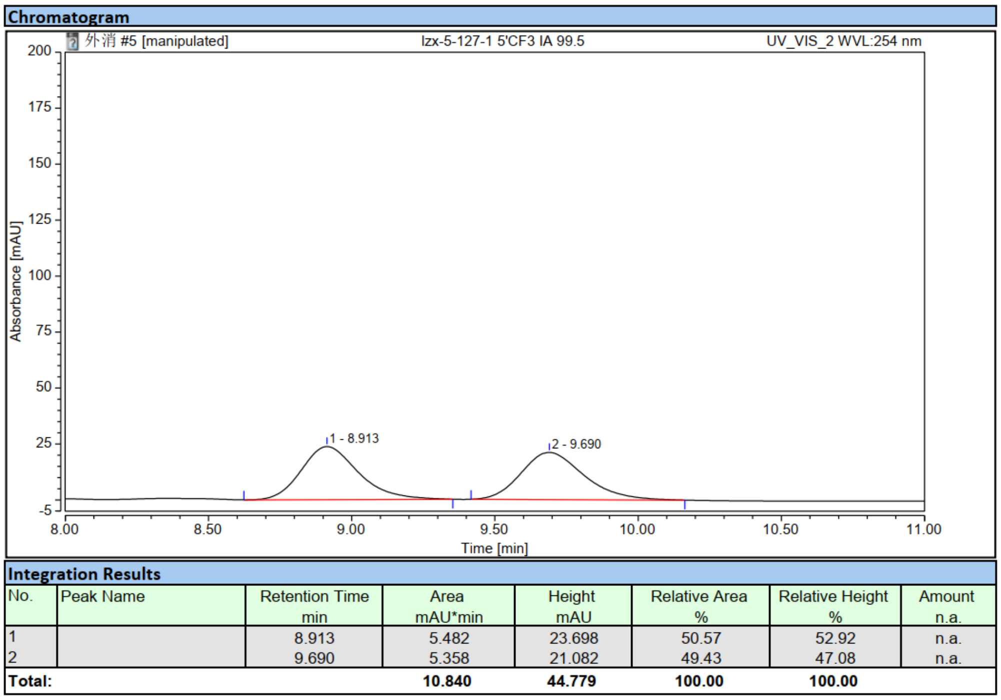

Integration Results

No.	Peak Name	Retention Time min	Area mAU*min	Height mAU	Relative Area %	Relative Height %	Amount n.a.
1		8.913	5.482	23.698	50.57	52.92	n.a.
2		9.690	5.358	21.082	49.43	47.08	n.a.
**Total:**			**10.840**	**44.779**	**100.00**	**100.00**	

Supplementary Fig. 297 HPLC spectra of rac-**3oa**

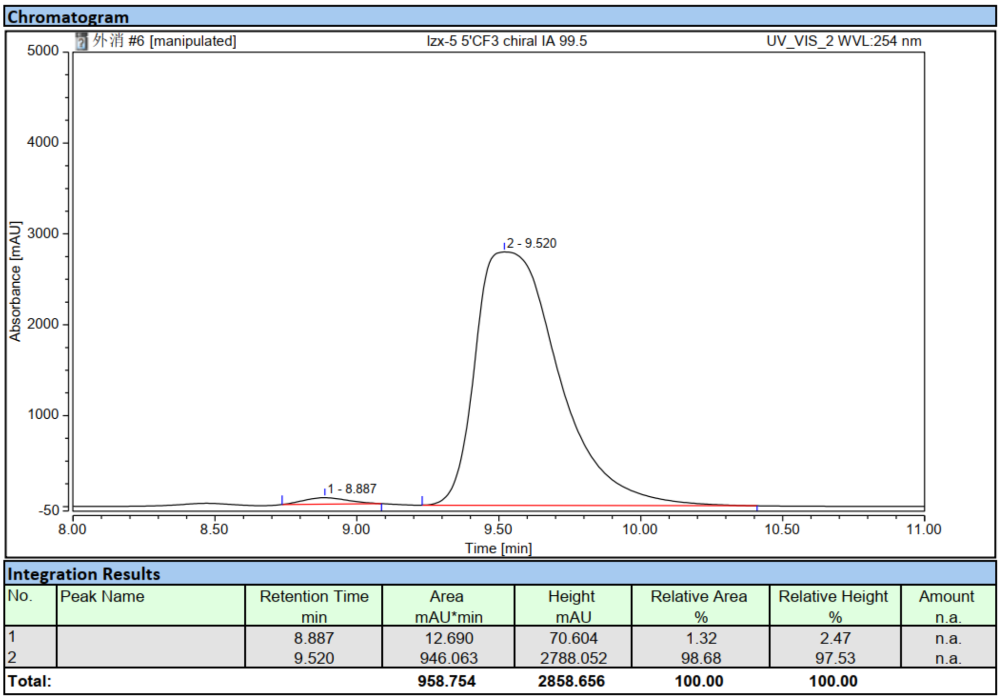

Integration Results

No.	Peak Name	Retention Time min	Area mAU*min	Height mAU	Relative Area %	Relative Height %	Amount n.a.
1		8.887	12.690	70.604	1.32	2.47	n.a.
2		9.520	946.063	2788.052	98.68	97.53	n.a.
**Total:**			**958.754**	**2858.656**	**100.00**	**100.00**	

Supplementary Fig. 298 HPLC spectra of **3oa**

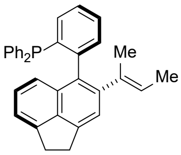

3pa

Chromatogram

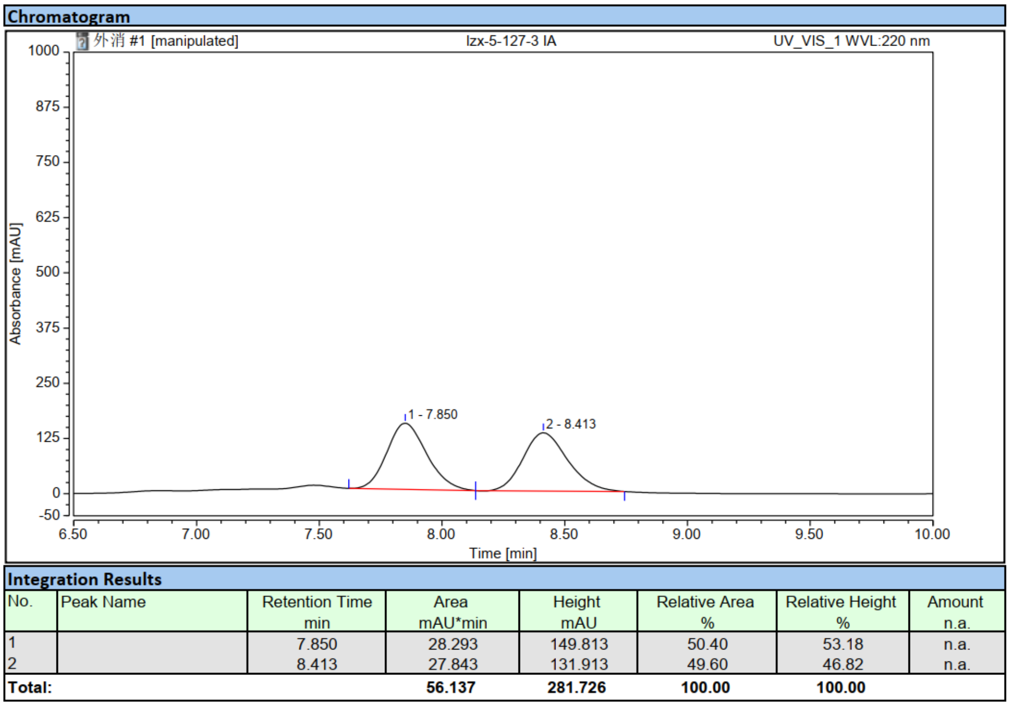

Integration Results

No.	Peak Name	Retention Time<br>min	Area<br>mAU*min	Height<br>mAU	Relative Area<br>%	Relative Height<br>%	Amount<br>n.a.
1		7.850	28.293	149.813	50.40	53.18	n.a.
2		8.413	27.843	131.913	49.60	46.82	n.a.
**Total:**			**56.137**	**281.726**	**100.00**	**100.00**	

Supplementary Fig. 299 HPLC spectra of rac-**3pa**

Chromatogram

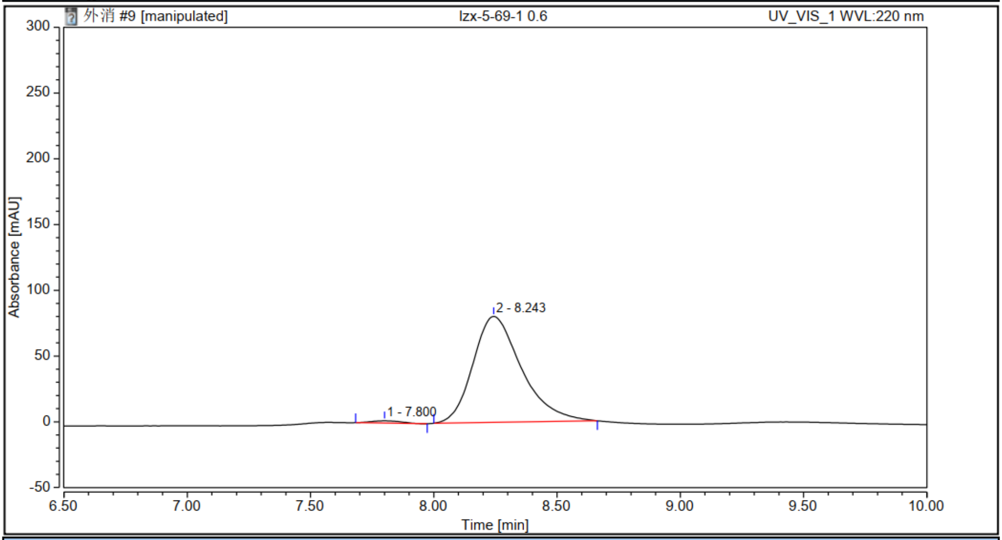

Integration Results

No.	Peak Name	Retention Time<br>min	Area<br>mAU*min	Height<br>mAU	Relative Area<br>%	Relative Height<br>%	Amount<br>n.a.
1		7.800	0.238	1.746	1.28	2.12	n.a.
2		8.243	18.396	80.545	98.72	97.88	n.a.
**Total:**			**18.634**	**82.291**	**100.00**	**100.00**	

Supplementary Fig. 300 HPLC spectra of **3pa**

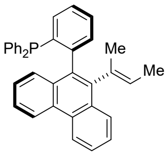

3qa

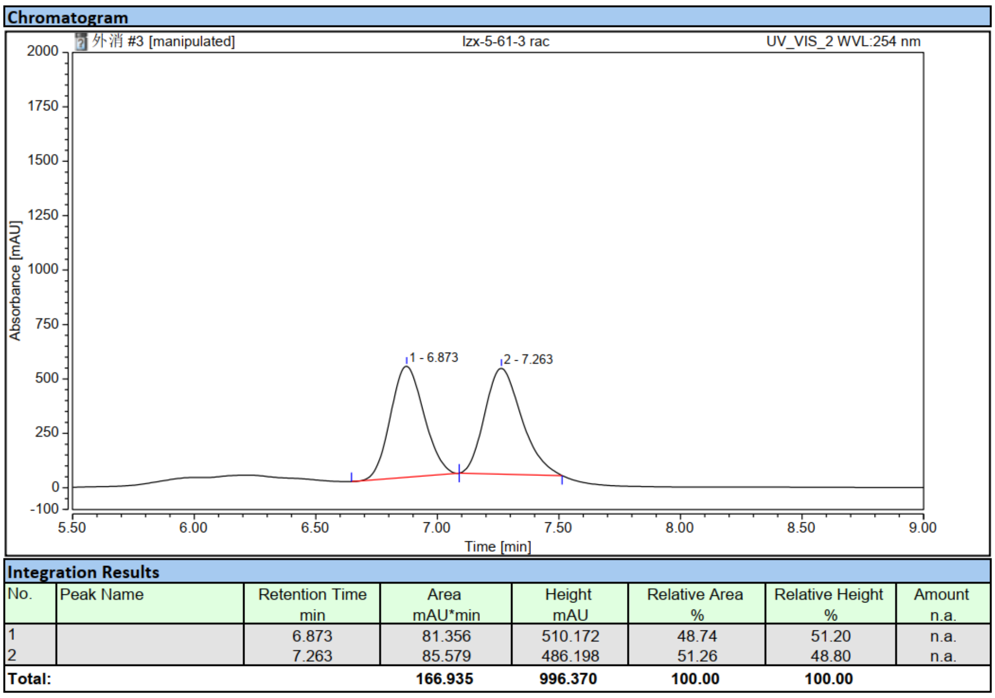

Integration Results

No.	Peak Name	Retention Time min	Area mAU*min	Height mAU	Relative Area %	Relative Height %	Amount n.a.
1		6.873	81.356	510.172	48.74	51.20	n.a.
2		7.263	85.579	486.198	51.26	48.80	n.a.
**Total:**			**166.935**	**996.370**	**100.00**	**100.00**	

Supplementary Fig. 301 HPLC spectra of rac-**3qa**

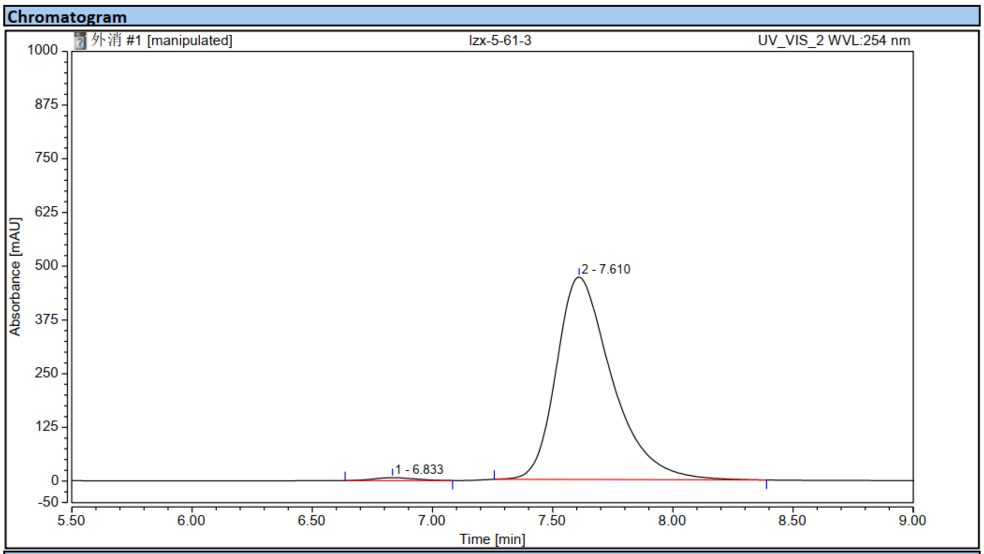

Integration Results

No.	Peak Name	Retention Time min	Area mAU*min	Height mAU	Relative Area %	Relative Height %	Amount n.a.
1		6.833	1.346	6.757	1.05	1.41	n.a.
2		7.610	126.477	470.797	98.95	98.59	n.a.
**Total:**			**127.823**	**477.555**	**100.00**	**100.00**	

Supplementary Fig. 302 HPLC spectra of **3qa**

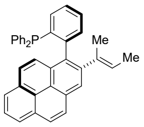

3ra

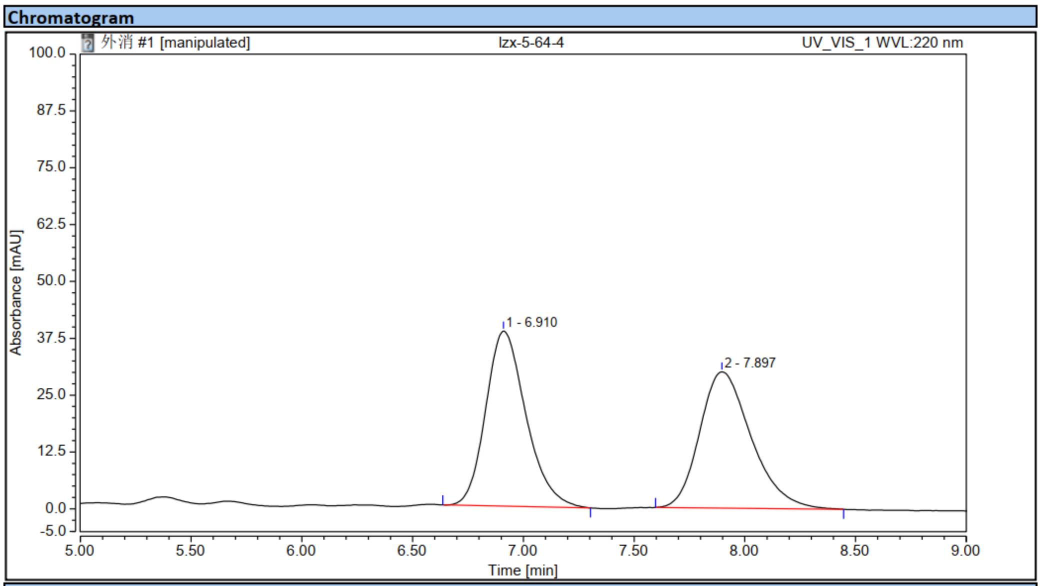

Integration Results

No.	Peak Name	Retention Time min	Area mAU*min	Height mAU	Relative Area %	Relative Height %	Amount n.a.
1		6.910	8.124	38.488	50.13	56.23	n.a.
2		7.897	8.081	29.957	49.87	43.77	n.a.
**Total:**			**16.205**	**68.446**	**100.00**	**100.00**	

Supplementary Fig. 303 HPLC spectra of rac-**3ra**

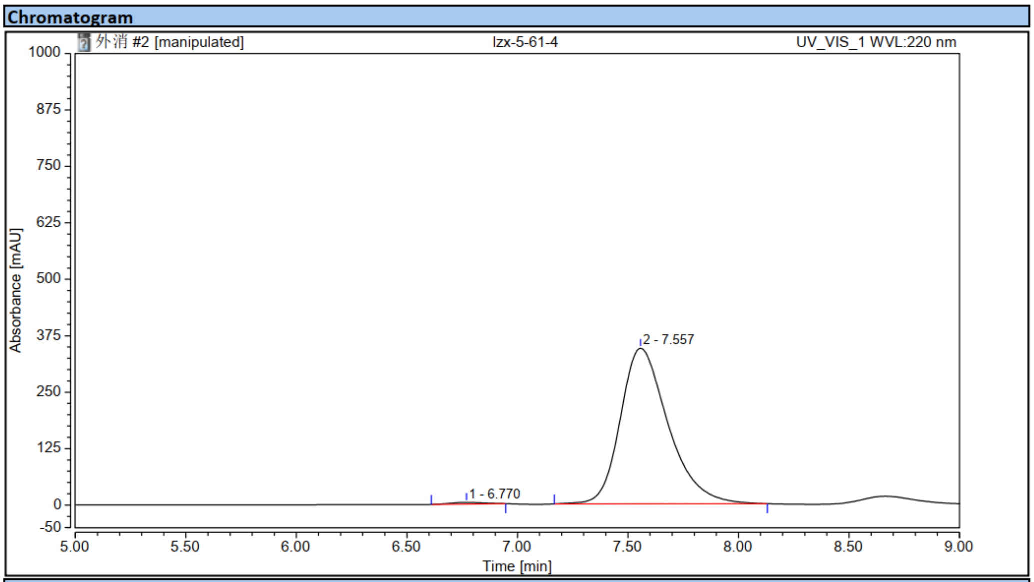

Integration Results

No.	Peak Name	Retention Time min	Area mAU*min	Height mAU	Relative Area %	Relative Height %	Amount n.a.
1		6.770	0.649	3.735	0.74	1.07	n.a.
2		7.557	86.567	344.469	99.26	98.93	n.a.
**Total:**			**87.216**	**348.204**	**100.00**	**100.00**	

Supplementary Fig. 304 HPLC spectra of **3ra**

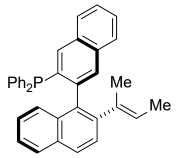

3sa

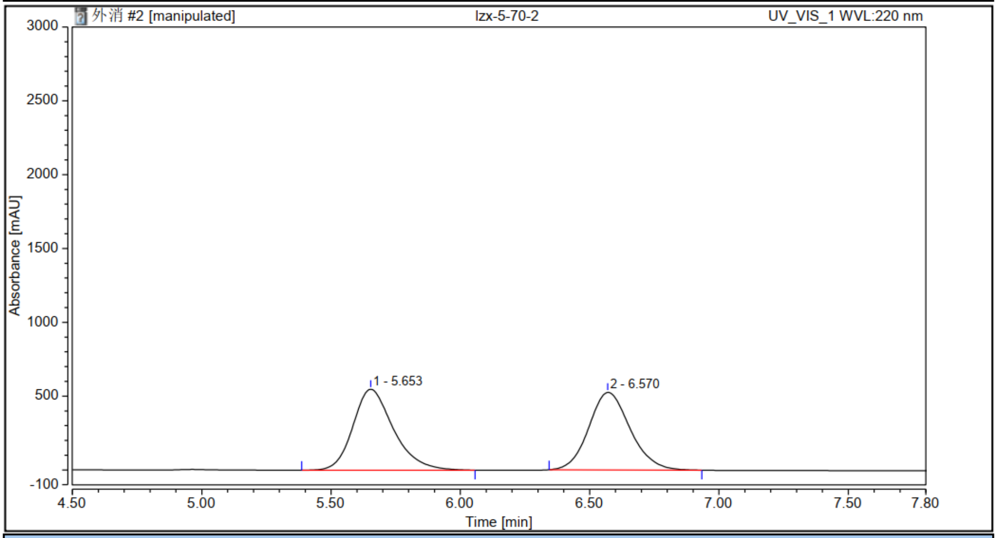

Integration Results

No.	Peak Name	Retention Time min	Area mAU*min	Height mAU	Relative Area %	Relative Height %	Amount n.a.
1		5.653	100.026	548.502	50.62	51.09	n.a.
2		6.570	97.557	525.117	49.38	48.91	n.a.
**Total:**			**197.583**	**1073.619**	**100.00**	**100.00**	

Supplementary Fig. 305 HPLC spectra of rac-**3sa**

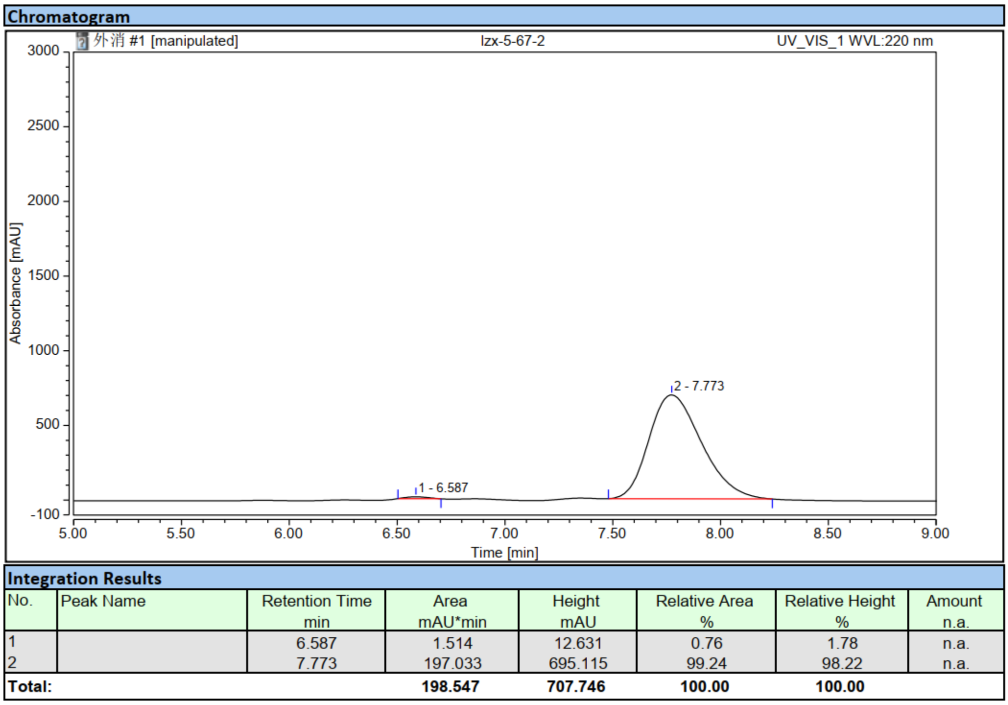

Integration Results

No.	Peak Name	Retention Time min	Area mAU*min	Height mAU	Relative Area %	Relative Height %	Amount n.a.
1		6.587	1.514	12.631	0.76	1.78	n.a.
2		7.773	197.033	695.115	99.24	98.22	n.a.
**Total:**			**198.547**	**707.746**	**100.00**	**100.00**	

Supplementary Fig. 306 HPLC spectra of **3sa**

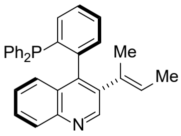

3ta

Chromatogram

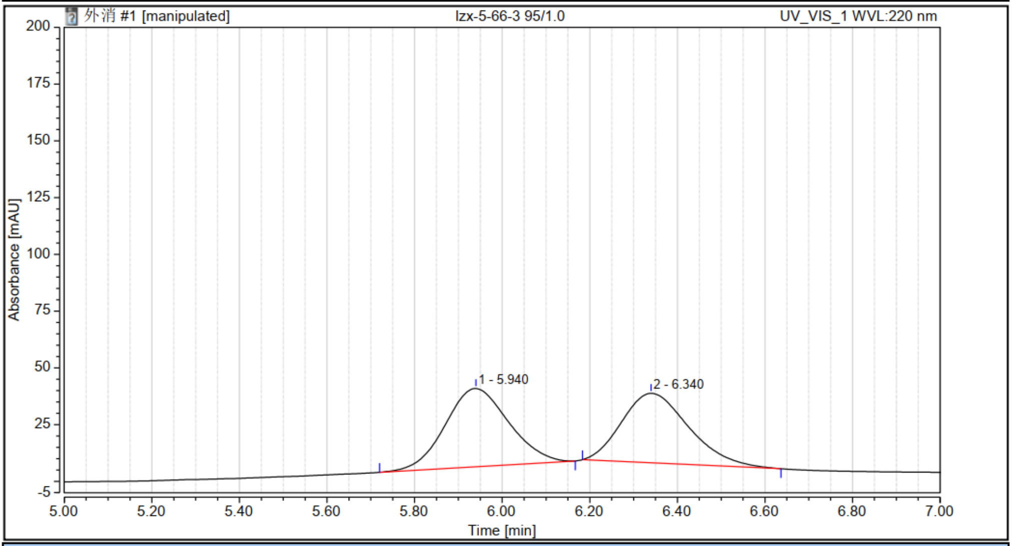

Integration Results

No.	Peak Name	Retention Time<br>min	Area<br>mAU*min	Height<br>mAU	Relative Area<br>%	Relative Height<br>%	Amount<br>n.a.
1		5.940	5.786	34.436	51.58	52.96	n.a.
2		6.340	5.433	30.592	48.42	47.04	n.a.
**Total:**			**11.219**	**65.028**	**100.00**	**100.00**	

Supplementary Fig. 307 HPLC spectra of rac-**3ta**

Chromatogram

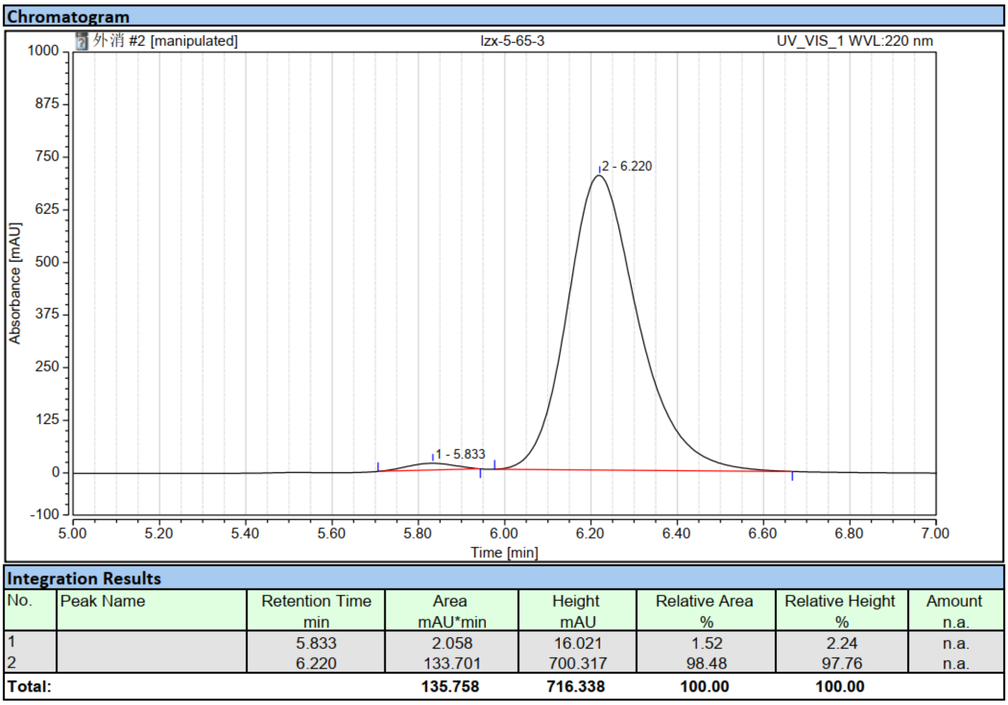

Integration Results

No.	Peak Name	Retention Time<br>min	Area<br>mAU*min	Height<br>mAU	Relative Area<br>%	Relative Height<br>%	Amount<br>n.a.
1		5.833	2.058	16.021	1.52	2.24	n.a.
2		6.220	133.701	700.317	98.48	97.76	n.a.
**Total:**			**135.758**	**716.338**	**100.00**	**100.00**	

Supplementary Fig. 308 HPLC spectra of **3ta**

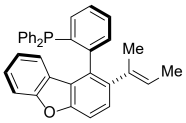

3ua

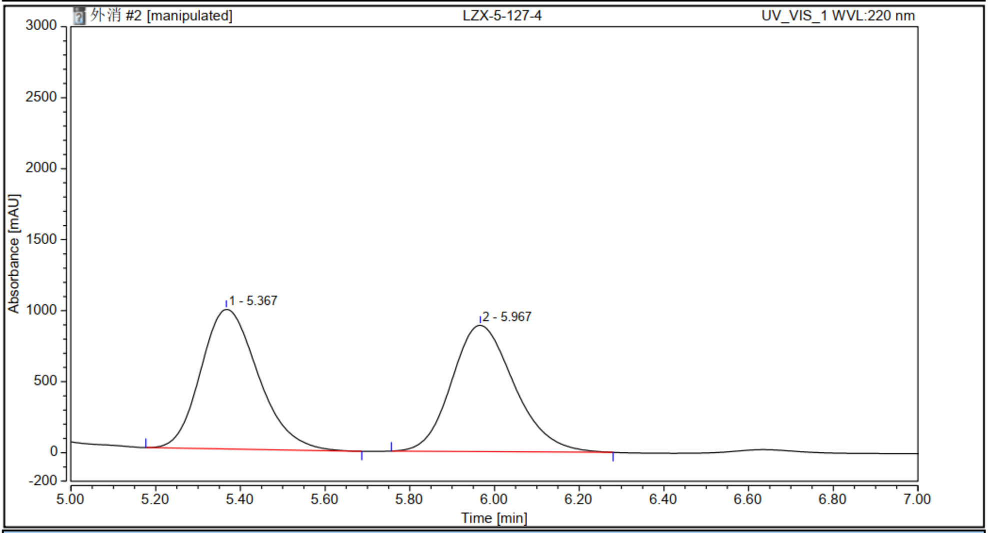

Integration Results

No.	Peak Name	Retention Time<br>min	Area<br>mAU*min	Height<br>mAU	Relative Area<br>%	Relative Height<br>%	Amount<br>n.a.
1		5.367	154.613	982.151	50.38	52.50	n.a.
2		5.967	152.297	888.676	49.62	47.50	n.a.
**Total:**			**306.910**	**1870.826**	**100.00**	**100.00**	

Supplementary Fig. 309 HPLC spectra of rac-**3ua**

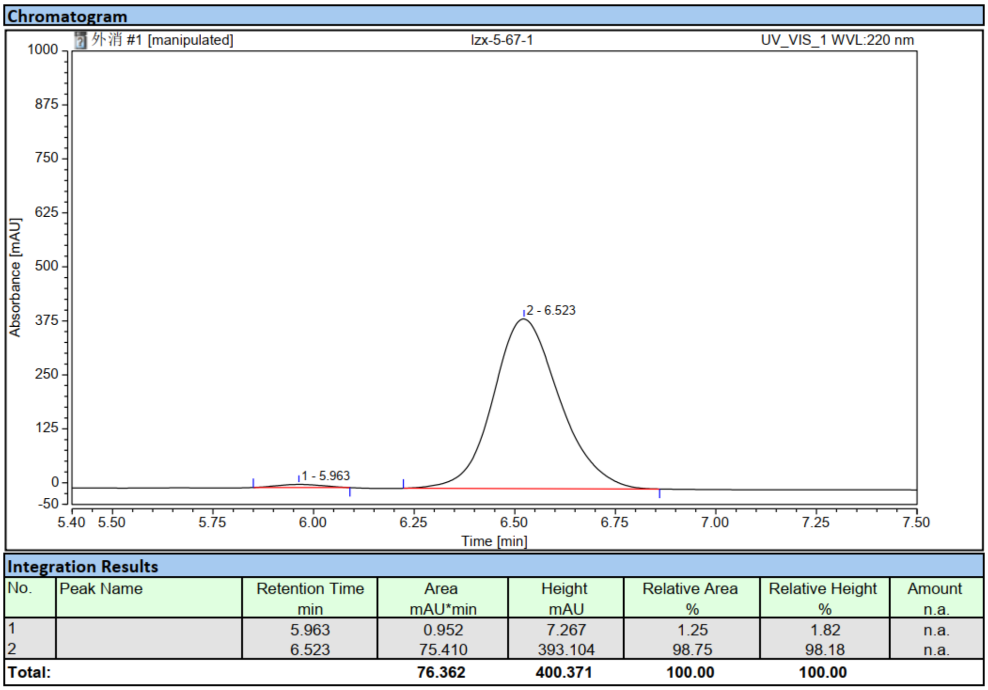

Integration Results

No.	Peak Name	Retention Time<br>min	Area<br>mAU*min	Height<br>mAU	Relative Area<br>%	Relative Height<br>%	Amount<br>n.a.
1		5.963	0.952	7.267	1.25	1.82	n.a.
2		6.523	75.410	393.104	98.75	98.18	n.a.
**Total:**			**76.362**	**400.371**	**100.00**	**100.00**	

Supplementary Fig. 310 HPLC spectra of **3ua**

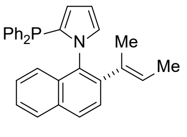

3va

Chromatogram

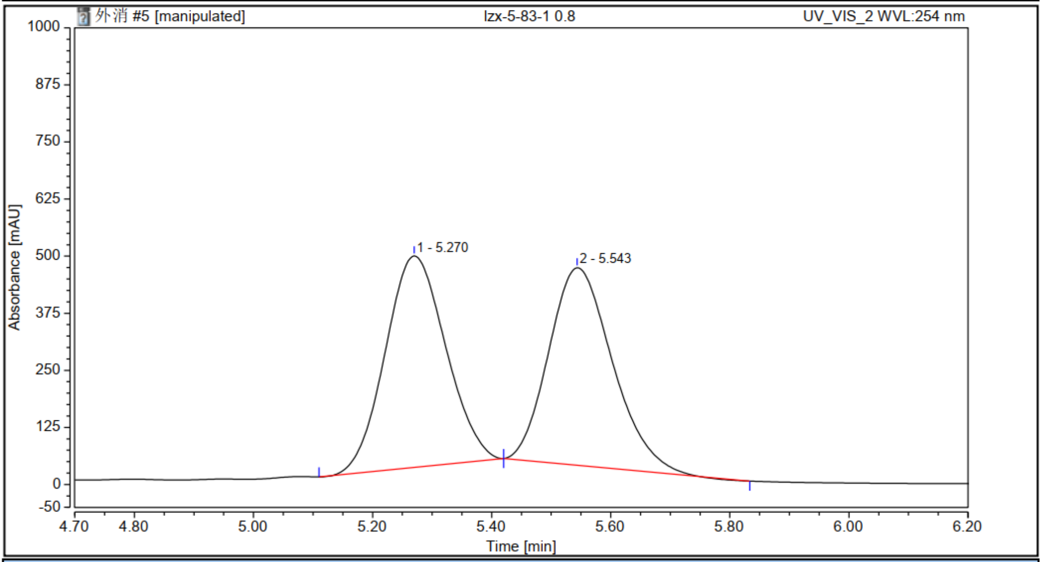

Integration Results

No.	Peak Name	Retention Time min	Area mAU*min	Height mAU	Relative Area %	Relative Height %	Amount n.a.
1		5.270	54.372	463.077	50.38	51.71	n.a.
2		5.543	53.551	432.521	49.62	48.29	n.a.
**Total:**			**107.923**	**895.597**	**100.00**	**100.00**	

Supplementary Fig. 311 HPLC spectra of rac-**3va**

Chromatogram

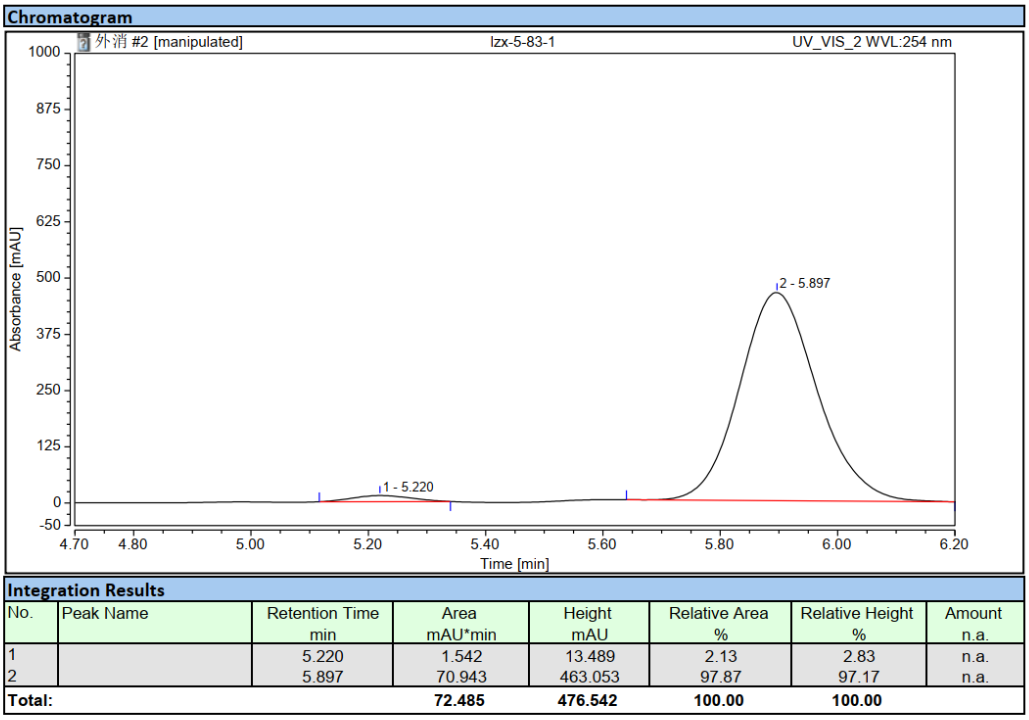

Integration Results

No.	Peak Name	Retention Time min	Area mAU*min	Height mAU	Relative Area %	Relative Height %	Amount n.a.
1		5.220	1.542	13.489	2.13	2.83	n.a.
2		5.897	70.943	463.053	97.87	97.17	n.a.
**Total:**			**72.485**	**476.542**	**100.00**	**100.00**	

Supplementary Fig. 312 HPLC spectra of **3va**

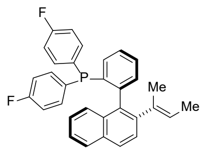

3wa

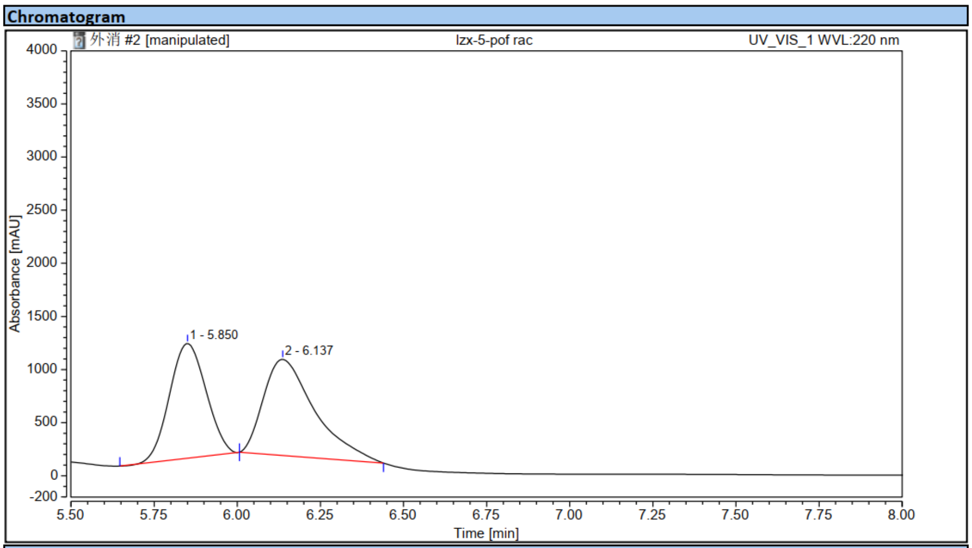

Integration Results

No.	Peak Name	Retention Time min	Area mAU*min	Height mAU	Relative Area %	Relative Height %	Amount n.a.
1		5.850	138.097	1080.081	46.13	54.42	n.a.
2		6.137	161.284	904.635	53.87	45.58	n.a.
**Total:**			**299.380**	**1984.716**	**100.00**	**100.00**	

Supplementary Fig. 313 HPLC spectra of rac-**3wa**

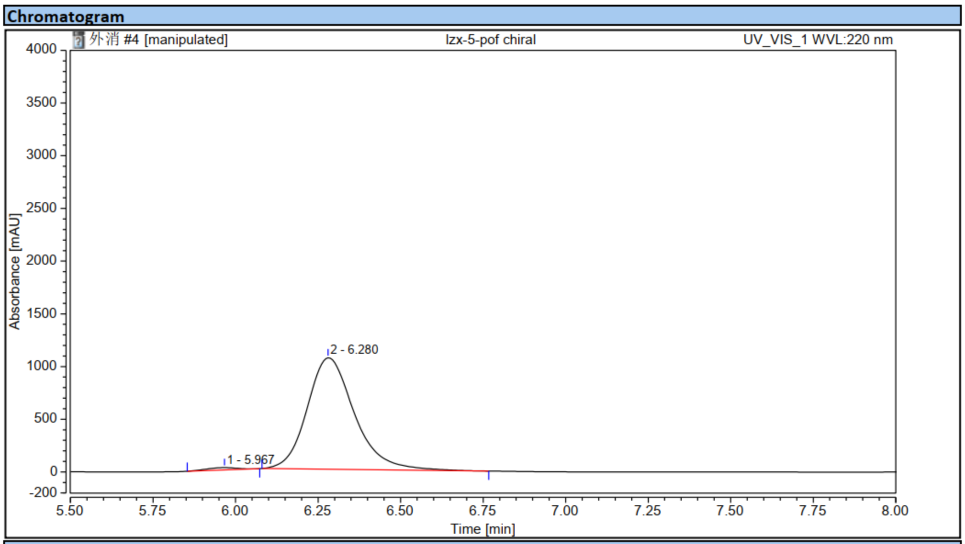

Integration Results

No.	Peak Name	Retention Time min	Area mAU*min	Height mAU	Relative Area %	Relative Height %	Amount n.a.
1		5.967	2.307	21.602	1.29	2.00	n.a.
2		6.280	175.997	1056.757	98.71	98.00	n.a.
**Total:**			**178.304**	**1078.359**	**100.00**	**100.00**	

Supplementary Fig. 314 HPLC spectra of **3wa**

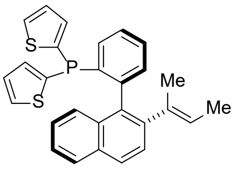

3xa

Chromatogram

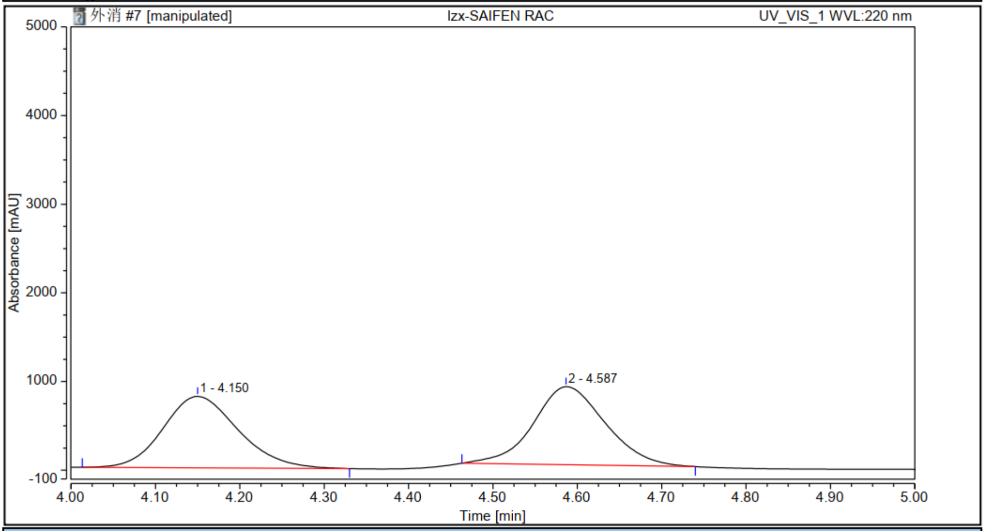

Integration Results

No.	Peak Name	Retention Time min	Area mAU*min	Height mAU	Relative Area %	Relative Height %	Amount n.a.
1		4.150	83.166	805.390	48.82	47.79	n.a.
2		4.587	87.172	880.004	51.18	52.21	n.a.
**Total:**			**170.337**	**1685.394**	**100.00**	**100.00**	

Supplementary Fig. 315 HPLC spectra of rac-**3xa**

Chromatogram

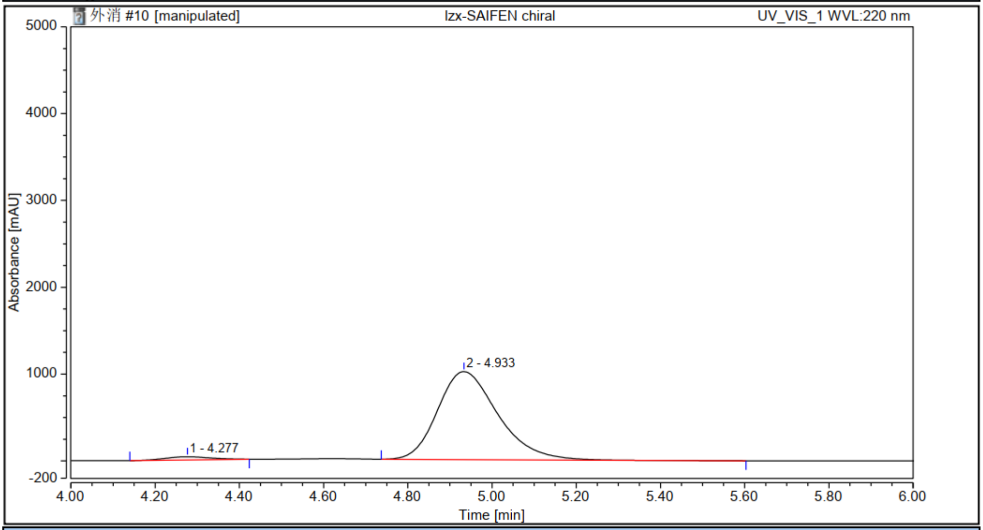

Integration Results

No.	Peak Name	Retention Time min	Area mAU*min	Height mAU	Relative Area %	Relative Height %	Amount n.a.
1		4.277	4.572	36.981	2.65	3.52	n.a.
2		4.933	168.125	1013.464	97.35	96.48	n.a.
**Total:**			**172.697**	**1050.445**	**100.00**	**100.00**	

Supplementary Fig. 316 HPLC spectra of **3xa**

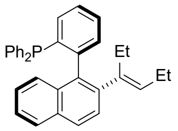

3ab

Chromatogram

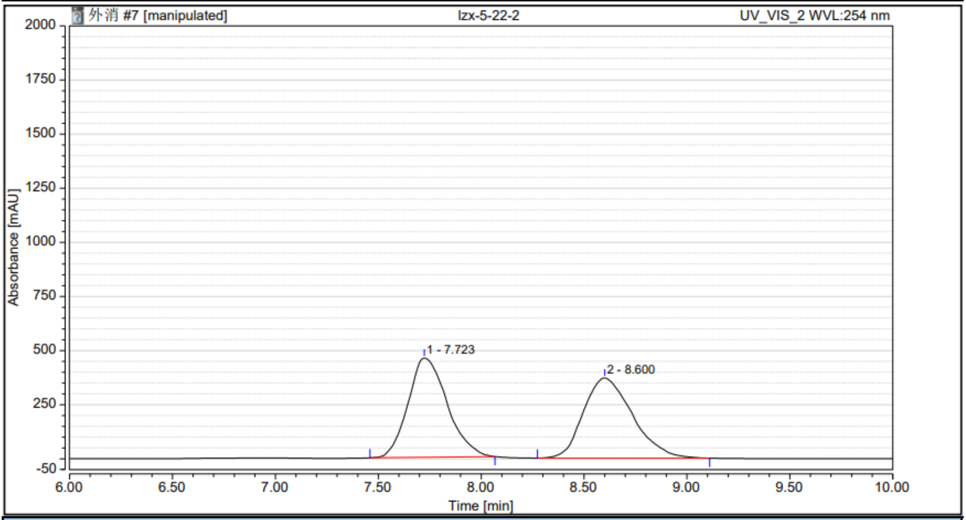

Integration Results

No.	Peak Name	Retention Time min	Area mAU*min	Height mAU	Relative Area %	Relative Height %	Amount n.a.
1		7.723	101.144	458.316	49.74	55.30	n.a.
2		8.600	102.202	370.456	50.26	44.70	n.a.
**Total:**			**203.345**	**828.772**	**100.00**	**100.00**	

Supplementary Fig. 317 HPLC spectra of rac-**3ab**

Chromatogram

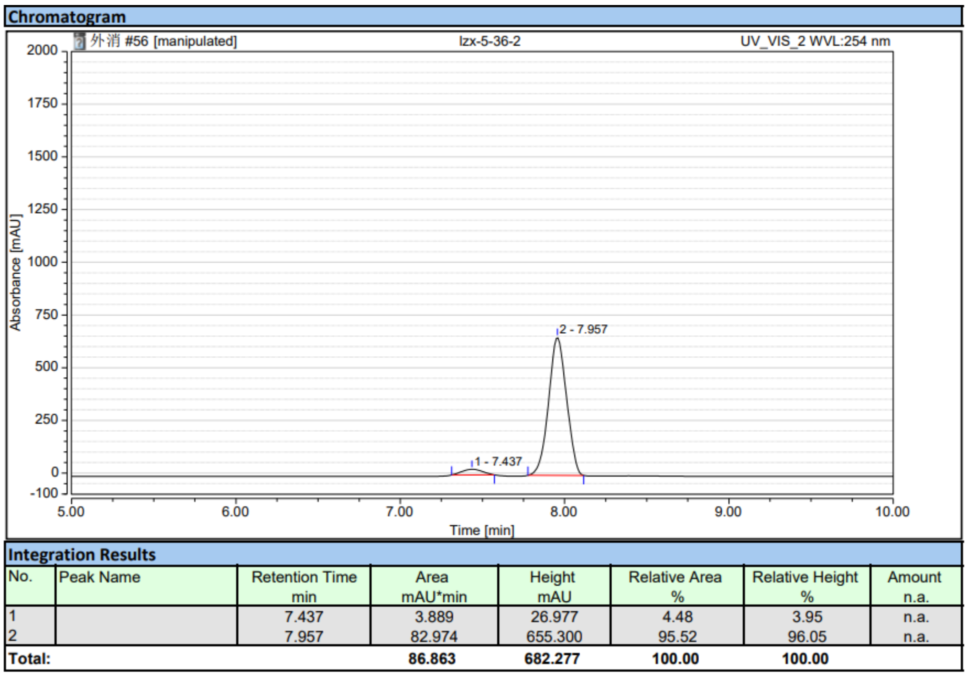

Integration Results

No.	Peak Name	Retention Time min	Area mAU*min	Height mAU	Relative Area %	Relative Height %	Amount n.a.
1		7.437	3.889	26.977	4.48	3.95	n.a.
2		7.957	82.974	655.300	95.52	96.05	n.a.
**Total:**			**86.863**	**682.277**	**100.00**	**100.00**	

Supplementary Fig. 318 HPLC spectra of **3ab**

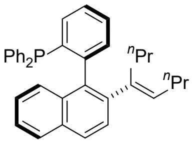

3ac

Chromatogram

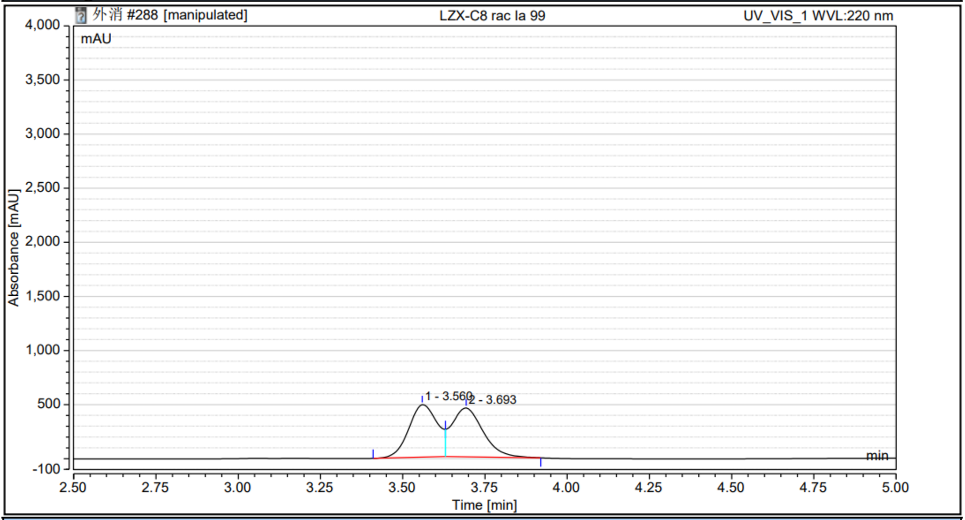

Integration Results

No.	Peak Name	Retention Time min	Area mAU*min	Height mAU	Relative Area %	Relative Height %	Amount n.a.
1		3.560	48.908	485.445	49.60	51.84	n.a.
2		3.693	49.696	450.987	50.40	48.16	n.a.
**Total:**			**98.604**	**936.432**	**100.00**	**100.00**	

Supplementary Fig. 319 HPLC spectra of rac-**3ac**

Chromatogram

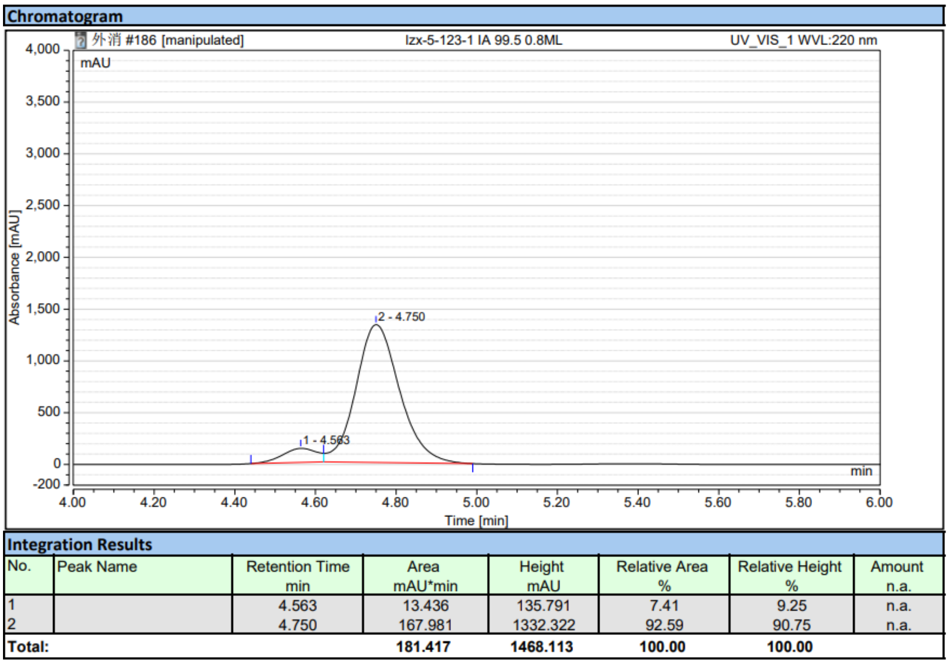

Integration Results

No.	Peak Name	Retention Time min	Area mAU*min	Height mAU	Relative Area %	Relative Height %	Amount n.a.
1		4.563	13.436	135.791	7.41	9.25	n.a.
2		4.750	167.981	1332.322	92.59	90.75	n.a.
**Total:**			**181.417**	**1468.113**	**100.00**	**100.00**	

Supplementary Fig. 320 HPLC spectra of **3ac**

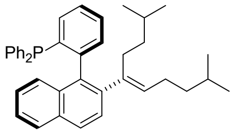

3ad

Chromatogram

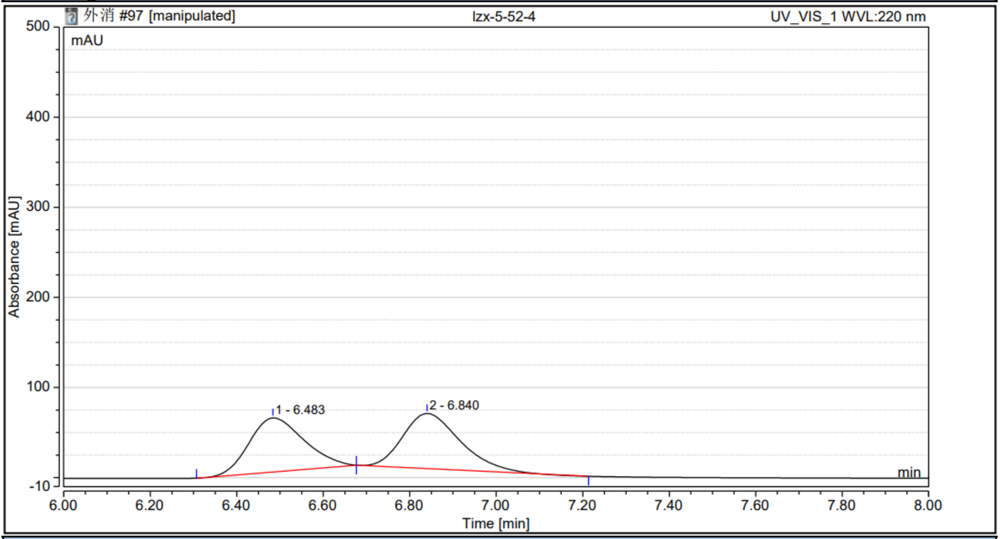

Integration Results

No.	Peak Name	Retention Time min	Area mAU*min	Height mAU	Relative Area %	Relative Height %	Amount n.a.
1		6.483	8.841	60.032	48.40	49.53	n.a.
2		6.840	9.425	61.176	51.60	50.47	n.a.
**Total:**			**18.266**	**121.208**	**100.00**	**100.00**	

Supplementary Fig. 321 HPLC spectra of rac-**3ad**

Chromatogram

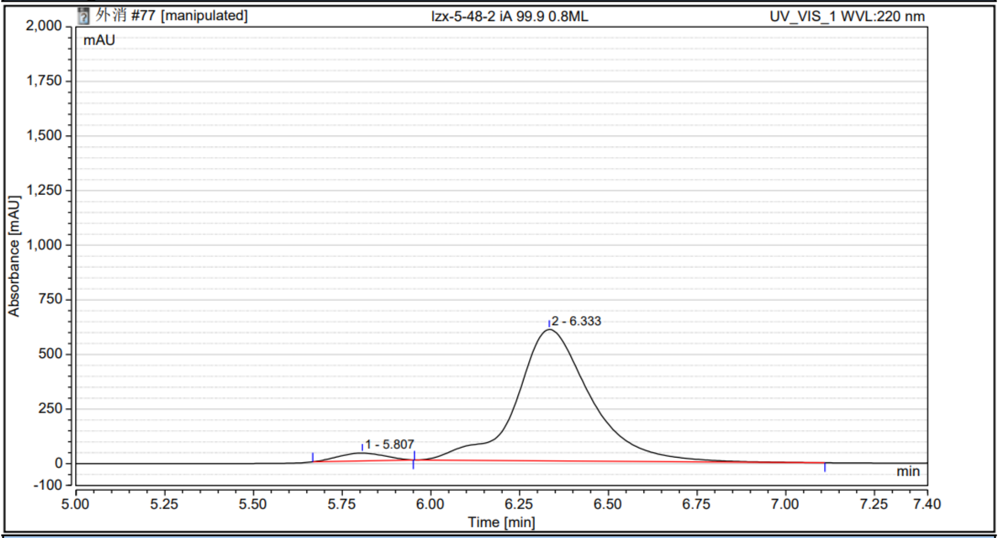

Integration Results

No.	Peak Name	Retention Time min	Area mAU*min	Height mAU	Relative Area %	Relative Height %	Amount n.a.
1		5.807	5.385	35.676	3.55	5.59	n.a.
2		6.333	146.436	602.092	96.45	94.41	n.a.
**Total:**			**151.821**	**637.768**	**100.00**	**100.00**	

Supplementary Fig. 322 HPLC spectra of **3ad**

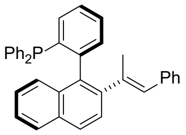

3ae

Chromatogram

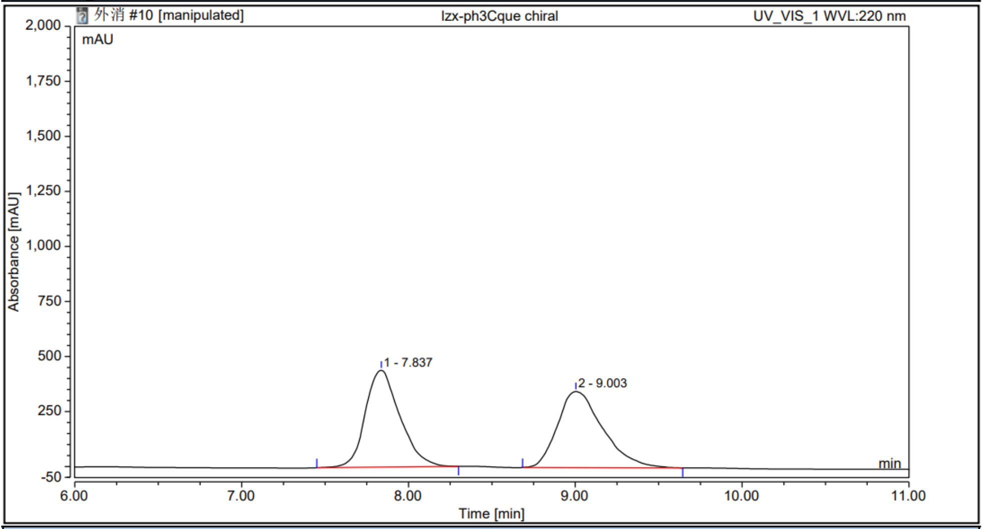

Integration Results

No.	Peak Name	Retention Time min	Area mAU*min	Height mAU	Relative Area %	Relative Height %	Amount n.a.
1		7.837	105.841	440.022	49.30	55.99	n.a.
2		9.003	108.849	345.926	50.70	44.01	n.a.
**Total:**			**214.690**	**785.947**	**100.00**	**100.00**	

Supplementary Fig. 323 HPLC spectra of rac-**3ae**

Chromatogram

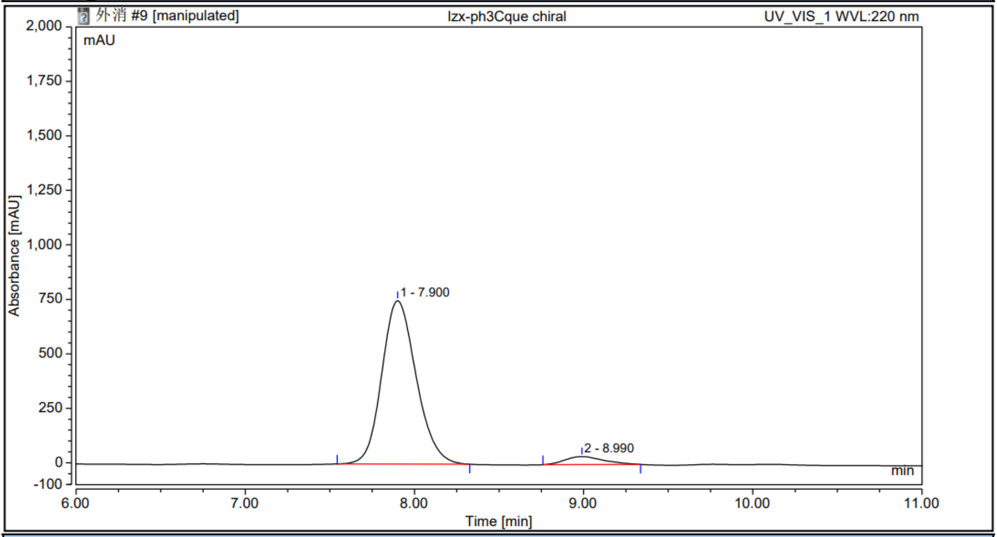

Integration Results

No.	Peak Name	Retention Time min	Area mAU*min	Height mAU	Relative Area %	Relative Height %	Amount n.a.
1		7.900	176.636	750.022	94.81	95.36	n.a.
2		8.990	9.677	36.476	5.19	4.64	n.a.
**Total:**			**186.314**	**786.498**	**100.00**	**100.00**	

Supplementary Fig. 324 HPLC spectra of **3ae**

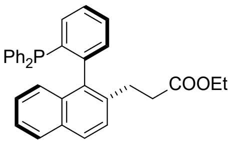

5aa

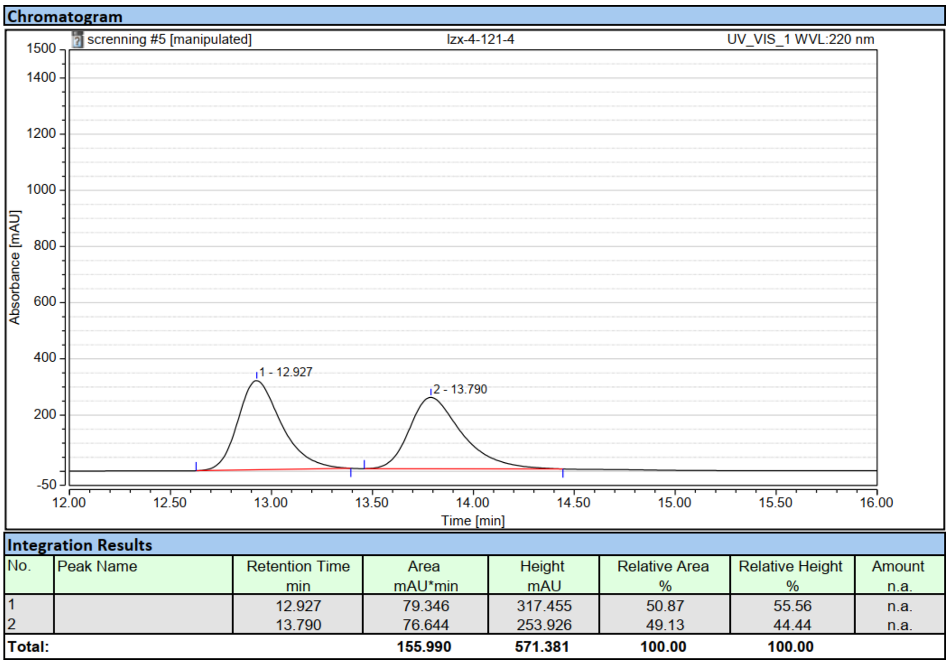

Integration Results

No.	Peak Name	Retention Time min	Area mAU*min	Height mAU	Relative Area %	Relative Height %	Amount n.a.
1		12.927	79.346	317.455	50.87	55.56	n.a.
2		13.790	76.644	253.926	49.13	44.44	n.a.
**Total:**			**155.990**	**571.381**	**100.00**	**100.00**	

Supplementary Fig. 325 HPLC spectra of rac-**5aa**

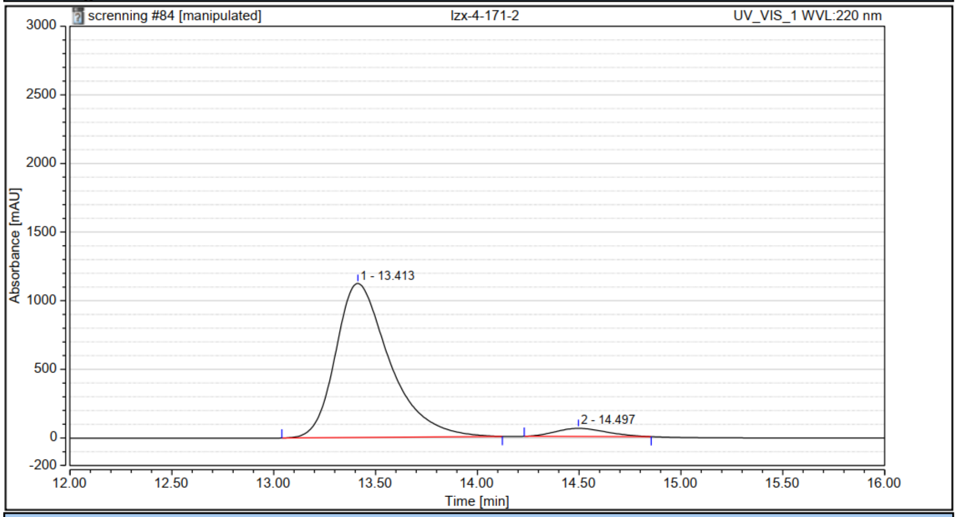

Integration Results

No.	Peak Name	Retention Time min	Area mAU*min	Height mAU	Relative Area %	Relative Height %	Amount n.a.
1		13.413	344.703	1121.997	95.26	95.00	n.a.
2		14.497	17.136	59.042	4.74	5.00	n.a.
**Total:**			**361.839**	**1181.039**	**100.00**	**100.00**	

Supplementary Fig. 326 HPLC spectra of **5ba**

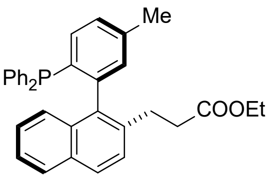

5ba

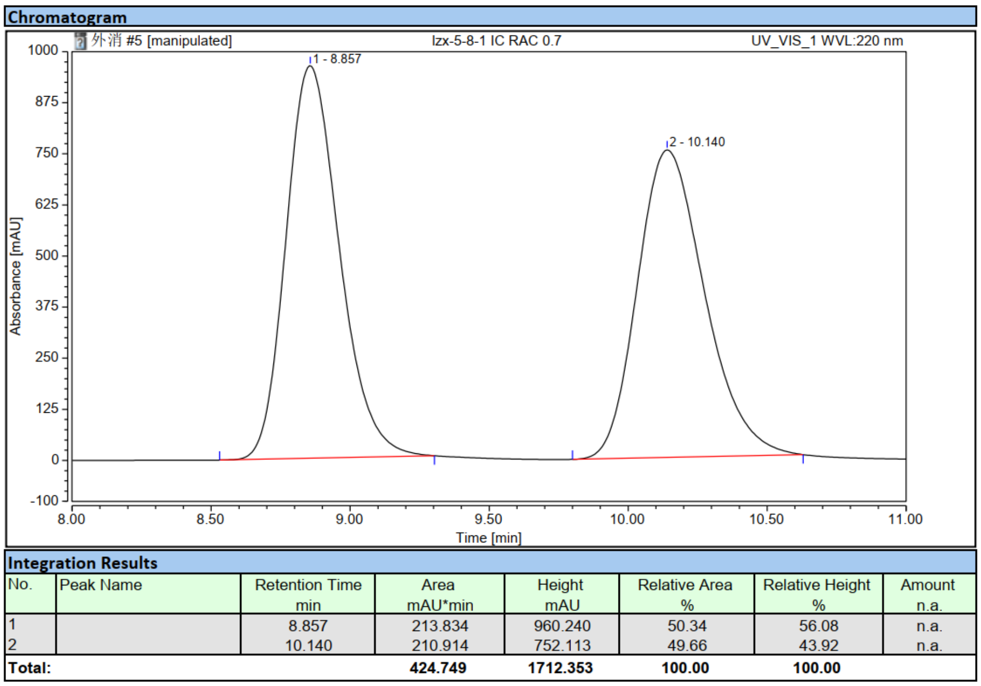

Integration Results

No.	Peak Name	Retention Time min	Area mAU*min	Height mAU	Relative Area %	Relative Height %	Amount n.a.
1		8.857	213.834	960.240	50.34	56.08	n.a.
2		10.140	210.914	752.113	49.66	43.92	n.a.
**Total:**			**424.749**	**1712.353**	**100.00**	**100.00**	

Supplementary Fig. 327 HPLC spectra of rac-**5ba**

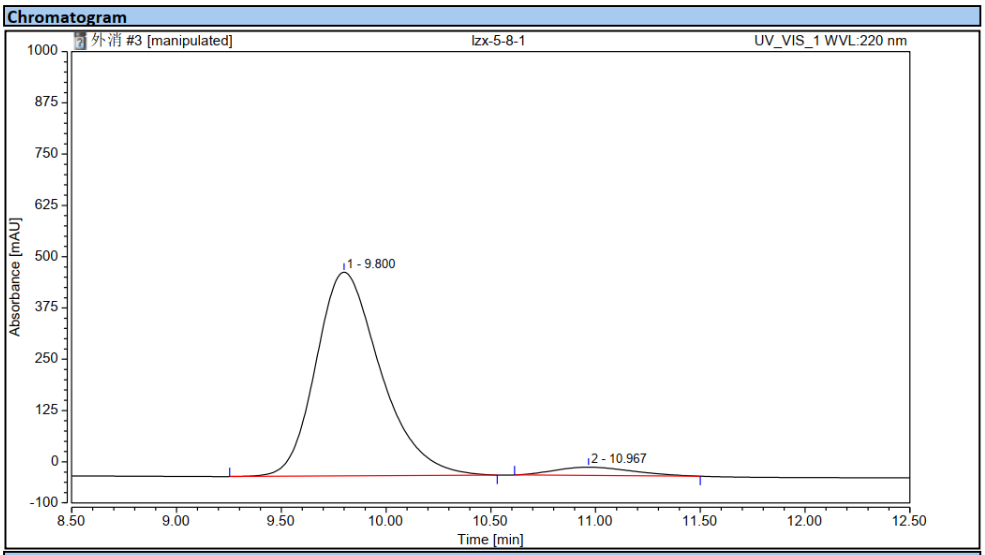

Integration Results

No.	Peak Name	Retention Time min	Area mAU*min	Height mAU	Relative Area %	Relative Height %	Amount n.a.
1		9.800	177.854	496.569	95.37	96.10	n.a.
2		10.967	8.641	20.158	4.63	3.90	n.a.
**Total:**			**186.496**	**516.727**	**100.00**	**100.00**	

Supplementary Fig. 328 HPLC spectra of **5ba**

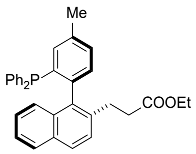

5ca

Chromatogram

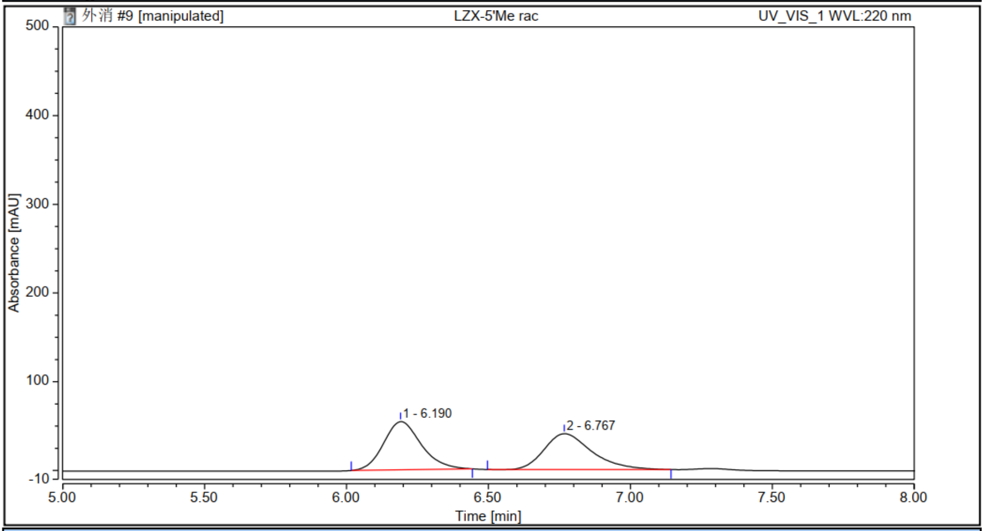

Integration Results

No.	Peak Name	Retention Time<br>min	Area<br>mAU*min	Height<br>mAU	Relative Area<br>%	Relative Height<br>%	Amount<br>n.a.
1		6.190	8.360	54.347	51.48	57.33	n.a.
2		6.767	7.878	40.446	48.52	42.67	n.a.
**Total:**			**16.238**	**94.793**	**100.00**	**100.00**	

Supplementary Fig. 329 HPLC spectra of rac-**5ca**

Chromatogram

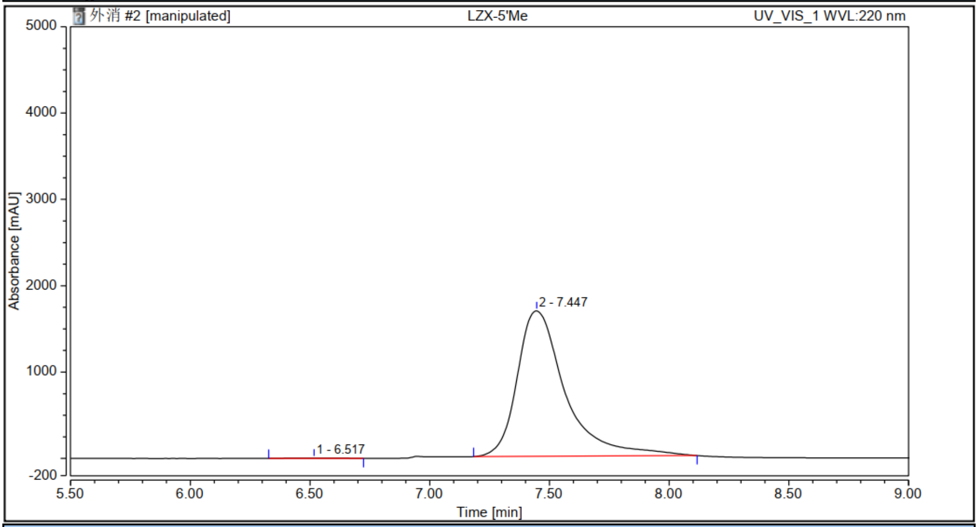

Integration Results

No.	Peak Name	Retention Time<br>min	Area<br>mAU*min	Height<br>mAU	Relative Area<br>%	Relative Height<br>%	Amount<br>n.a.
1		6.517	0.866	3.710	0.22	0.22	n.a.
2		7.447	391.741	1684.907	99.78	99.78	n.a.
**Total:**			**392.607**	**1688.616**	**100.00**	**100.00**	

Supplementary Fig. 330 HPLC spectra of **5ca**

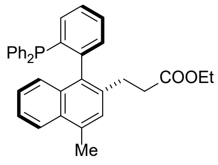

5da

Chromatogram

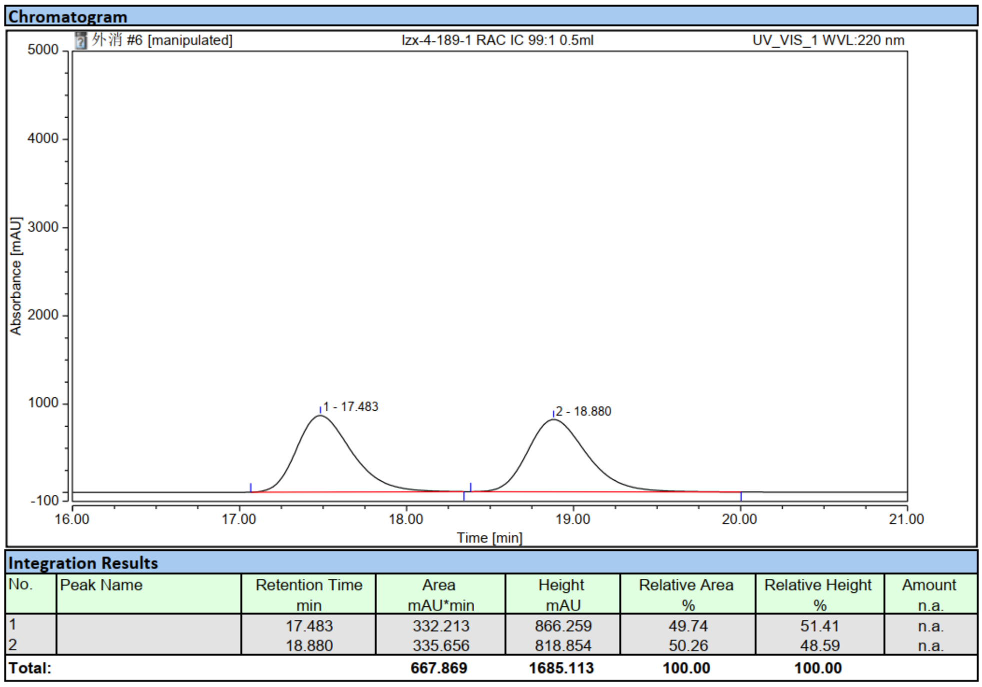

Integration Results

No.	Peak Name	Retention Time min	Area mAU*min	Height mAU	Relative Area %	Relative Height %	Amount n.a.
1		17.483	332.213	866.259	49.74	51.41	n.a.
2		18.880	335.656	818.854	50.26	48.59	n.a.
**Total:**			**667.869**	**1685.113**	**100.00**	**100.00**	

Supplementary Fig. 331 HPLC spectra of rac-**5da**

Chromatogram

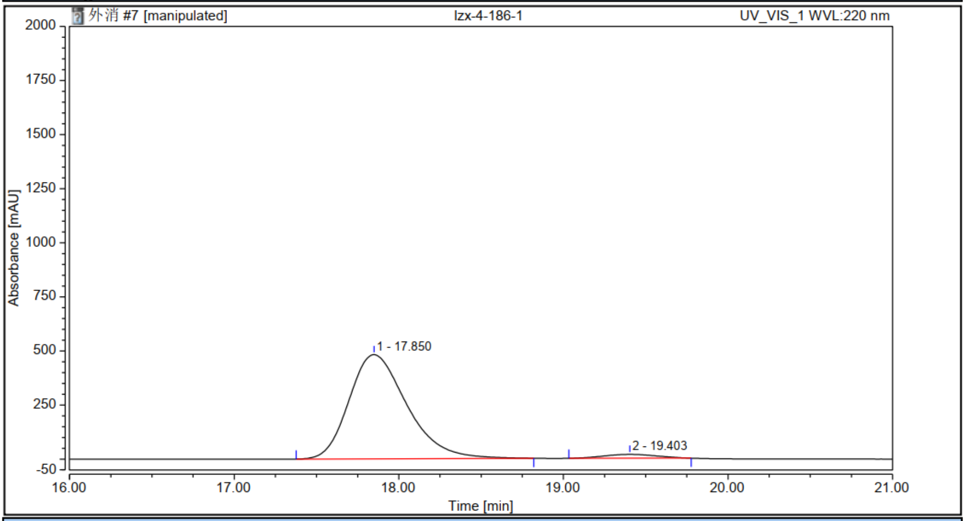

Integration Results

No.	Peak Name	Retention Time min	Area mAU*min	Height mAU	Relative Area %	Relative Height %	Amount n.a.
1		17.850	196.217	481.607	96.72	96.44	n.a.
2		19.403	6.655	17.791	3.28	3.56	n.a.
**Total:**			**202.872**	**499.398**	**100.00**	**100.00**	

Supplementary Fig. 332 HPLC spectra of **5da**

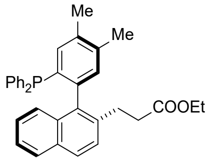

5ea

Chromatogram

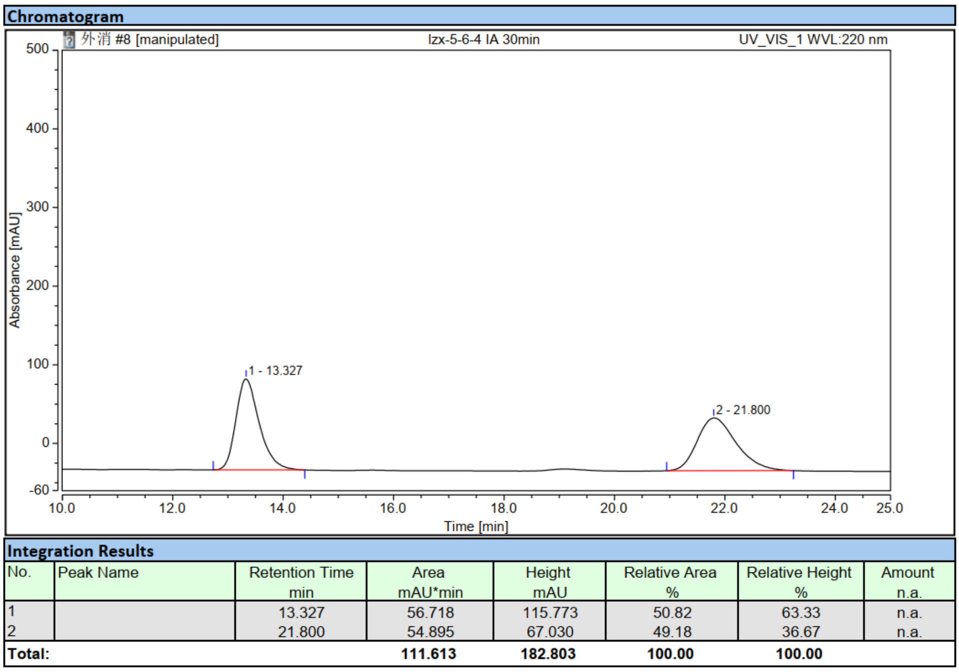

Integration Results

No.	Peak Name	Retention Time min	Area mAU*min	Height mAU	Relative Area %	Relative Height %	Amount n.a.
1		13.327	56.718	115.773	50.82	63.33	n.a.
2		21.800	54.895	67.030	49.18	36.67	n.a.
**Total:**			**111.613**	**182.803**	**100.00**	**100.00**	

Supplementary Fig. 333 HPLC spectra of rac-**5ea**

Chromatogram

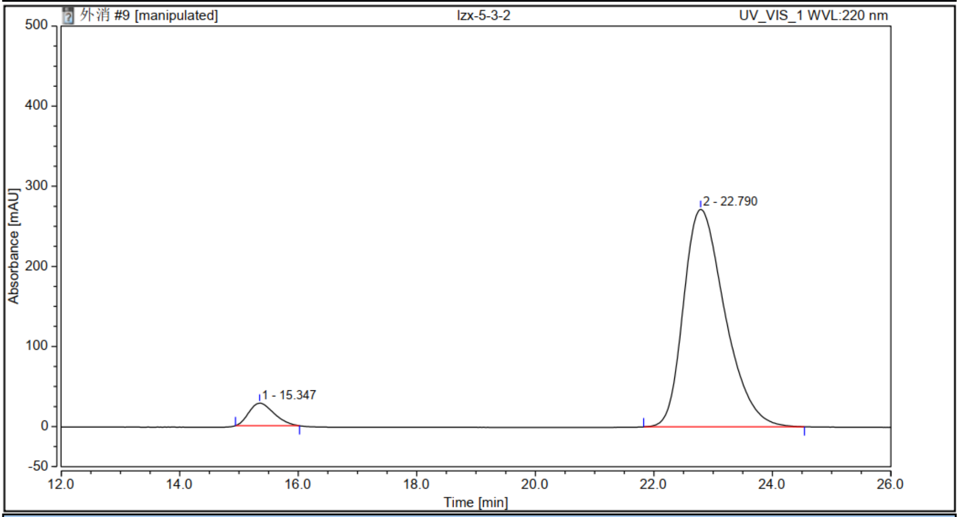

Integration Results

No.	Peak Name	Retention Time min	Area mAU*min	Height mAU	Relative Area %	Relative Height %	Amount n.a.
1		15.347	13.817	28.113	5.92	9.39	n.a.
2		22.790	219.662	271.384	94.08	90.61	n.a.
**Total:**			**233.478**	**299.497**	**100.00**	**100.00**	

Supplementary Fig. 334 HPLC spectra of **5ea**

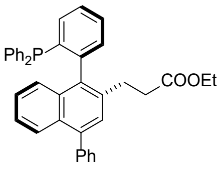

5fa

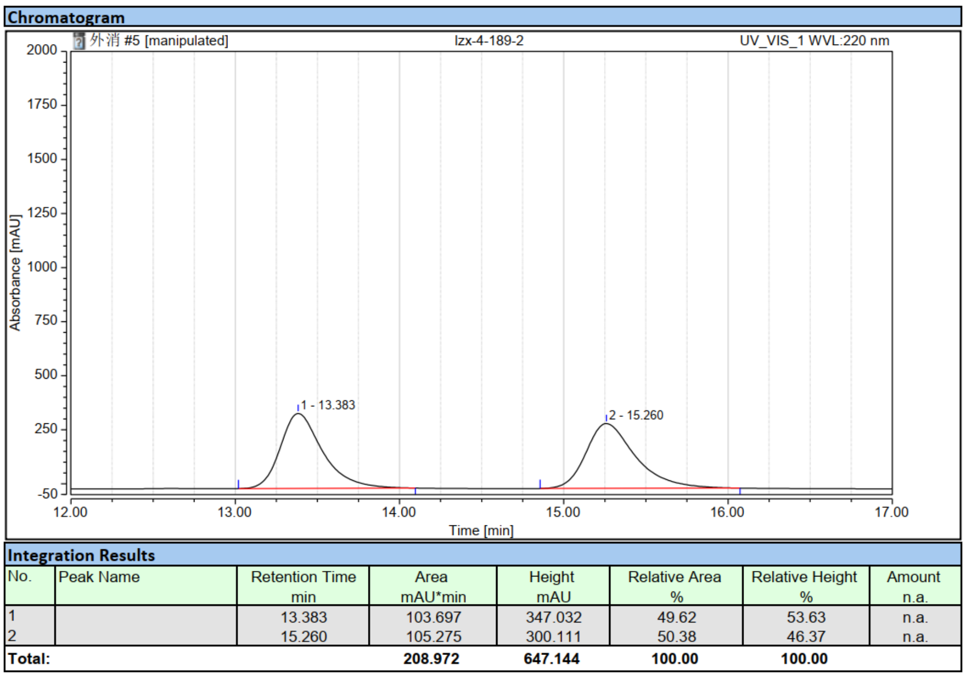

Integration Results

No.	Peak Name	Retention Time min	Area mAU*min	Height mAU	Relative Area %	Relative Height %	Amount n.a.
1		13.383	103.697	347.032	49.62	53.63	n.a.
2		15.260	105.275	300.111	50.38	46.37	n.a.
**Total:**			**208.972**	**647.144**	**100.00**	**100.00**	

Supplementary Fig. 335 HPLC spectra of rac-**5fa**

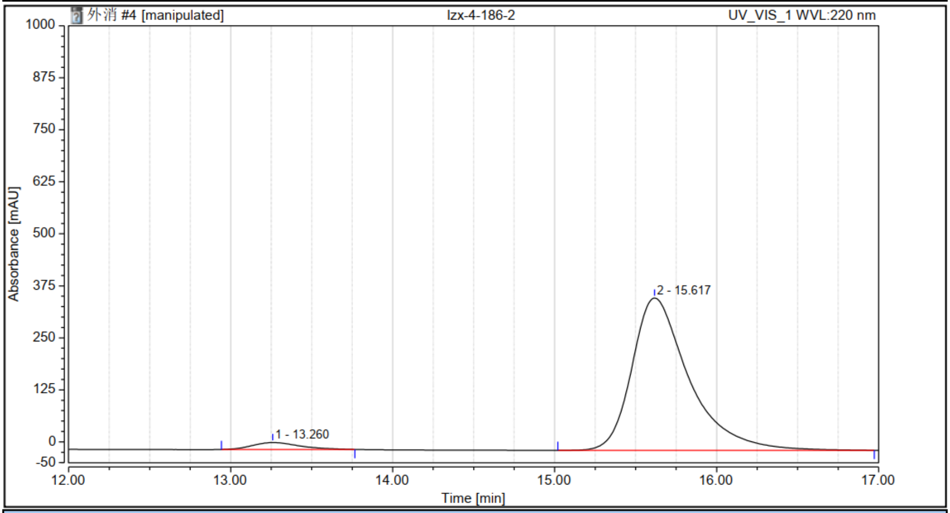

Integration Results

No.	Peak Name	Retention Time min	Area mAU*min	Height mAU	Relative Area %	Relative Height %	Amount n.a.
1		13.260	5.638	16.670	3.63	4.35	n.a.
2		15.617	149.840	366.159	96.37	95.65	n.a.
**Total:**			**155.478**	**382.828**	**100.00**	**100.00**	

Supplementary Fig. 336 HPLC spectra of **5fa**

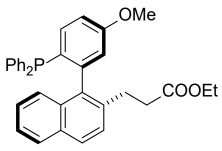

5ga

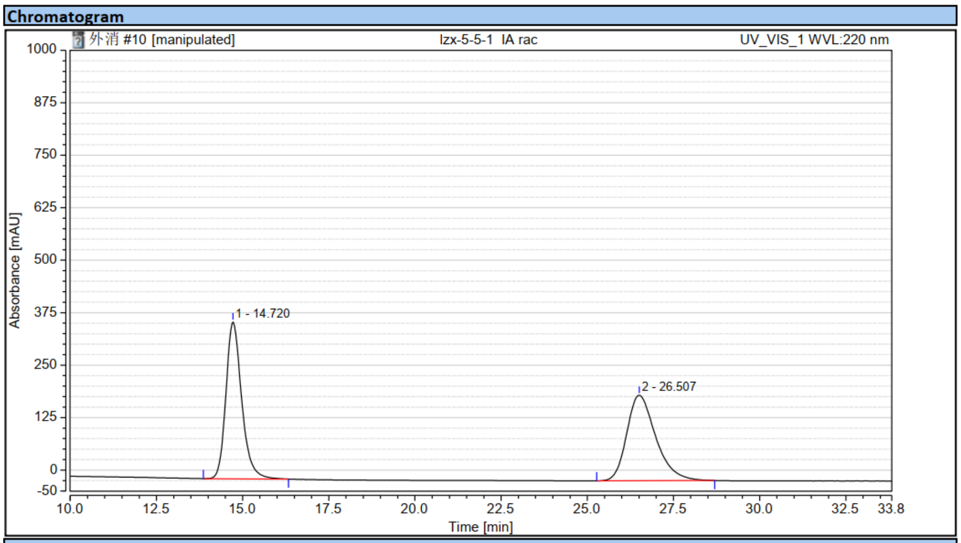

Integration Results

No.	Peak Name	Retention Time min	Area mAU*min	Height mAU	Relative Area %	Relative Height %	Amount n.a.
1		14.720	197.794	373.766	49.87	64.72	n.a.
2		26.507	198.843	203.703	50.13	35.28	n.a.
**Total:**			**396.637**	**577.470**	**100.00**	**100.00**	

Supplementary Fig. 337 HPLC spectra of rac-**5ga**

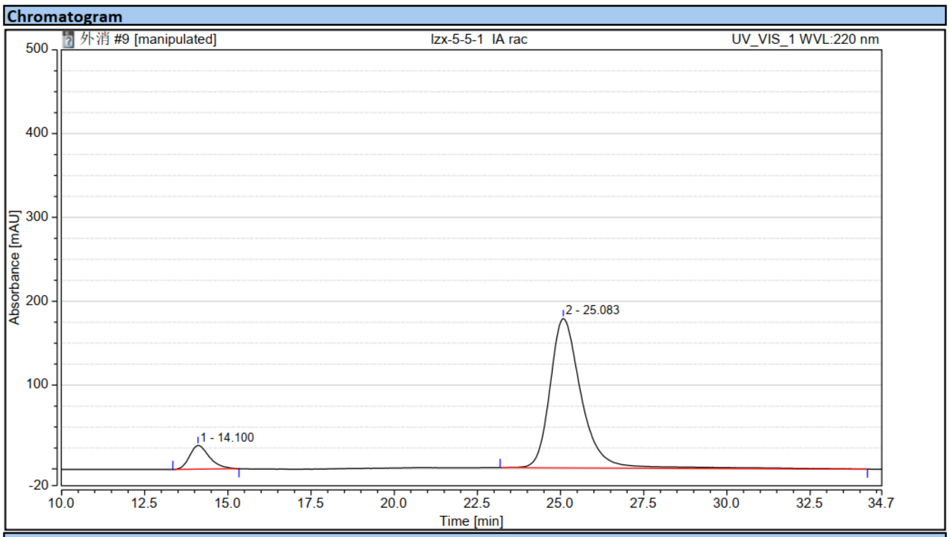

Integration Results

No.	Peak Name	Retention Time min	Area mAU*min	Height mAU	Relative Area %	Relative Height %	Amount n.a.
1		14.100	19.520	28.237	9.06	13.69	n.a.
2		25.083	195.931	178.020	90.94	86.31	n.a.
**Total:**			**215.450**	**206.257**	**100.00**	**100.00**	

Supplementary Fig. 338 HPLC spectra of **5ga**

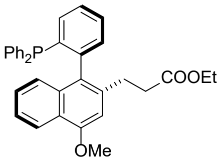

5ha

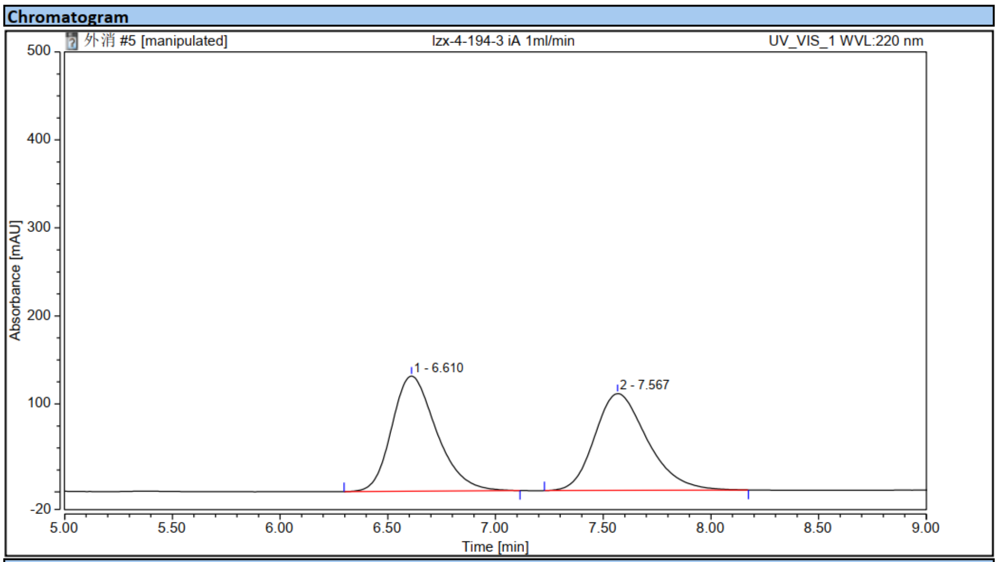

Integration Results

No.	Peak Name	Retention Time min	Area mAU*min	Height mAU	Relative Area %	Relative Height %	Amount n.a.
1		6.610	31.415	130.712	50.15	54.30	n.a.
2		7.567	31.226	109.990	49.85	45.70	n.a.
**Total:**			**62.640**	**240.702**	**100.00**	**100.00**	

Supplementary Fig. 339 HPLC spectra of rac-**5ha**

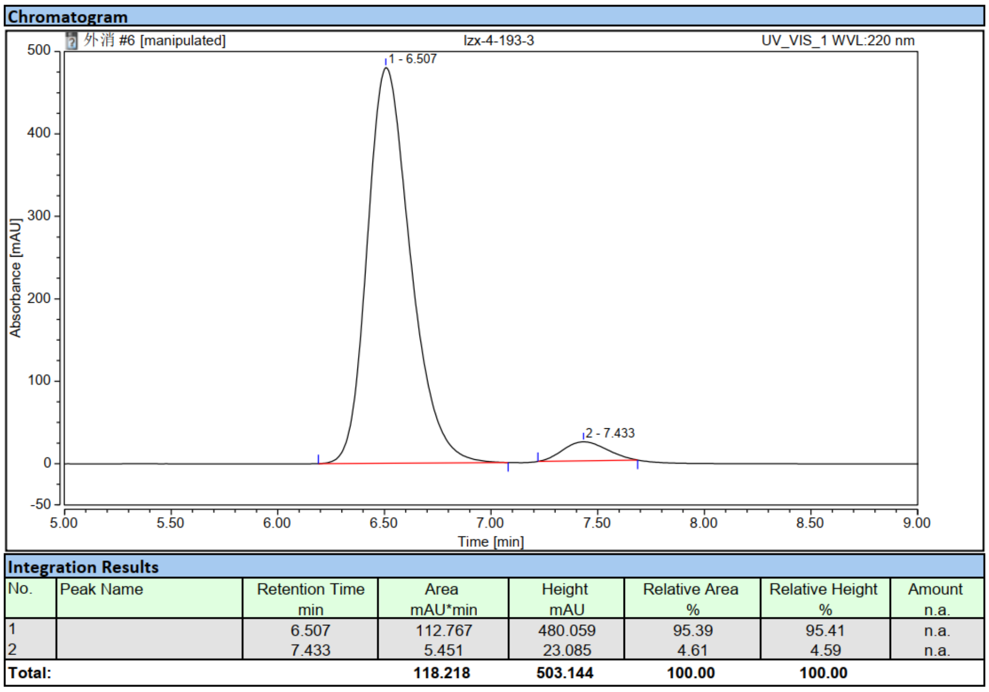

Integration Results

No.	Peak Name	Retention Time min	Area mAU*min	Height mAU	Relative Area %	Relative Height %	Amount n.a.
1		6.507	112.767	480.059	95.39	95.41	n.a.
2		7.433	5.451	23.085	4.61	4.59	n.a.
**Total:**			**118.218**	**503.144**	**100.00**	**100.00**	

Supplementary Fig. 340 HPLC spectra of **5ha**

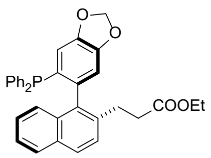

5ia

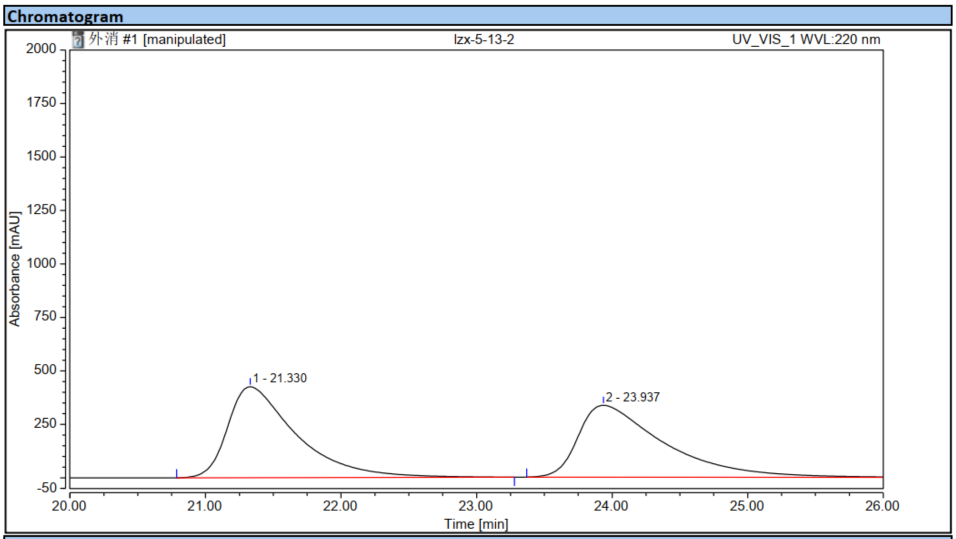

Integration Results

No.	Peak Name	Retention Time min	Area mAU*min	Height mAU	Relative Area %	Relative Height %	Amount n.a.
1		21.330	249.105	424.951	50.18	55.84	n.a.
2		23.937	247.292	336.075	49.82	44.16	n.a.
**Total:**			**496.397**	**761.026**	**100.00**	**100.00**	

Supplementary Fig. 341 HPLC spectra of rac-**5ia**

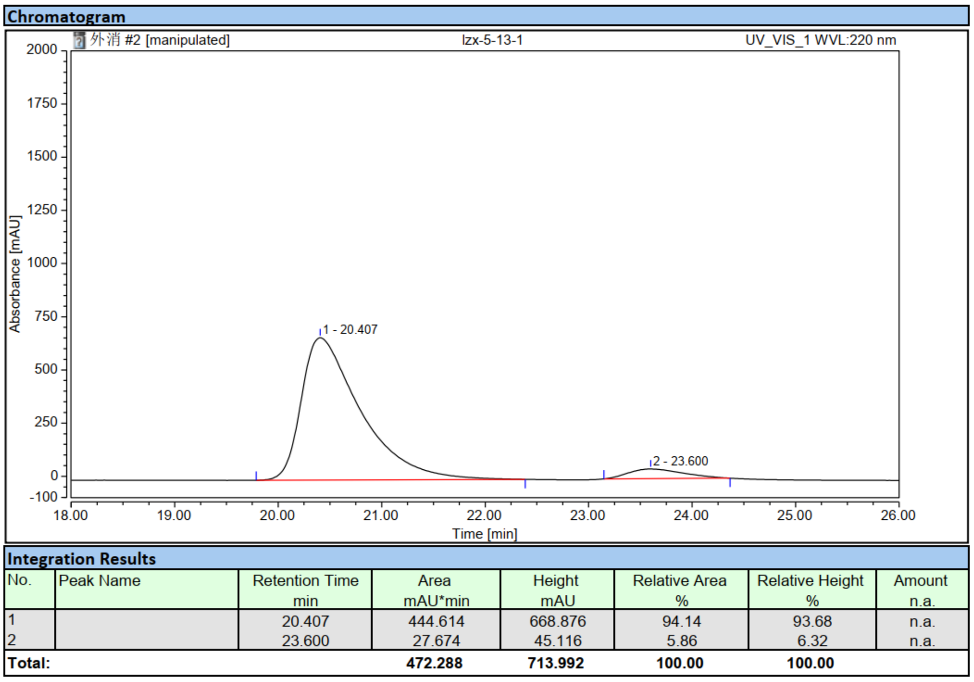

Integration Results

No.	Peak Name	Retention Time min	Area mAU*min	Height mAU	Relative Area %	Relative Height %	Amount n.a.
1		20.407	444.614	668.876	94.14	93.68	n.a.
2		23.600	27.674	45.116	5.86	6.32	n.a.
**Total:**			**472.288**	**713.992**	**100.00**	**100.00**	

Supplementary Fig. 342 HPLC spectra of **5ia**

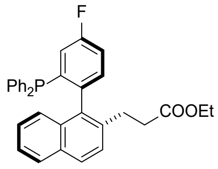

5ja

Chromatogram

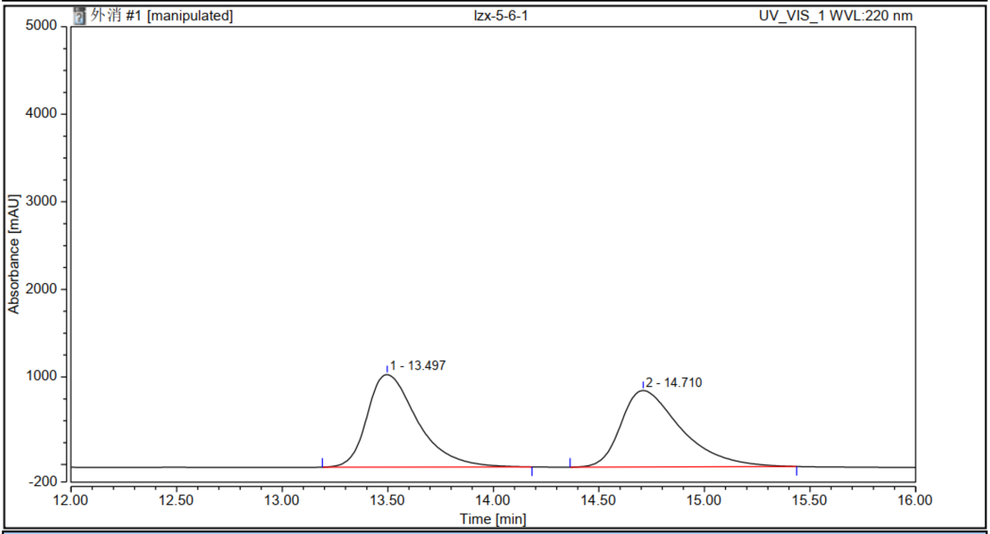

Integration Results

No.	Peak Name	Retention Time min	Area mAU*min	Height mAU	Relative Area %	Relative Height %	Amount n.a.
1		13.497	291.644	1054.875	50.29	54.76	n.a.
2		14.710	288.228	871.622	49.71	45.24	n.a.
**Total:**			**579.872**	**1926.497**	**100.00**	**100.00**	

Supplementary Fig. 343 HPLC spectra of rac-**5ja**

Chromatogram

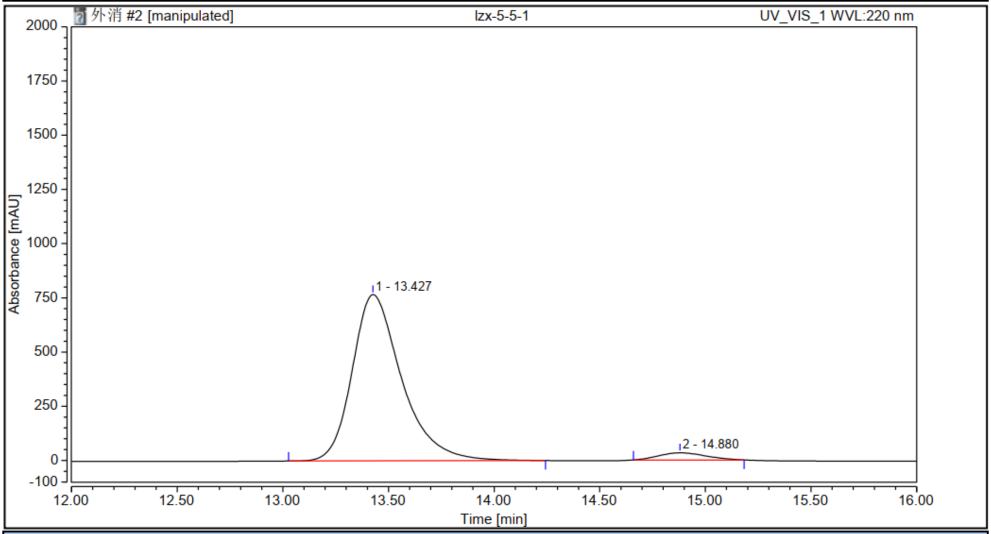

Integration Results

No.	Peak Name	Retention Time min	Area mAU*min	Height mAU	Relative Area %	Relative Height %	Amount n.a.
1		13.427	208.274	766.942	96.11	95.90	n.a.
2		14.880	8.422	32.792	3.89	4.10	n.a.
**Total:**			**216.696**	**799.734**	**100.00**	**100.00**	

Supplementary Fig. 344 HPLC spectra of **5ja**

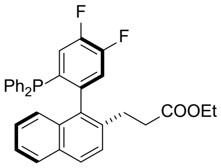

5ka

Chromatogram

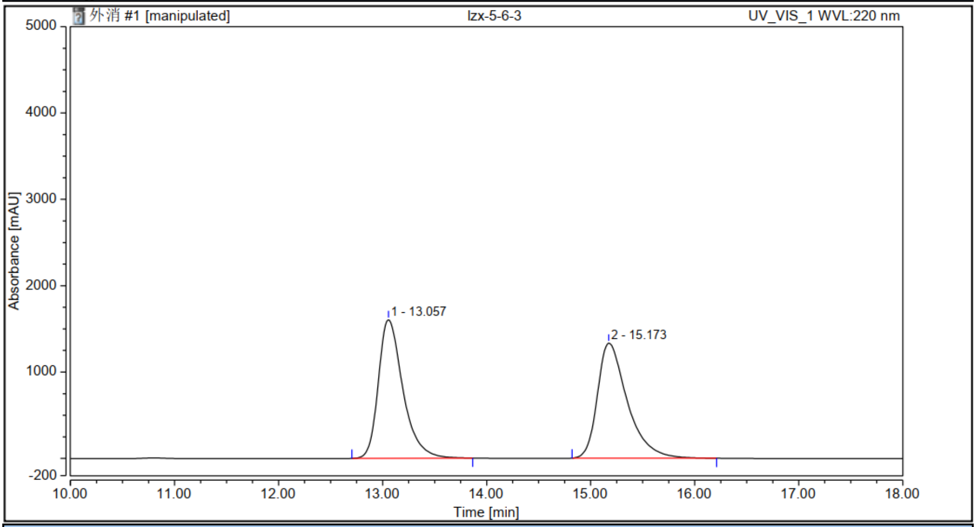

Integration Results

No.	Peak Name	Retention Time min	Area mAU*min	Height mAU	Relative Area %	Relative Height %	Amount n.a.
1		13.057	439.246	1604.837	49.70	54.65	n.a.
2		15.173	444.524	1331.924	50.30	45.35	n.a.
**Total:**			**883.770**	**2936.761**	**100.00**	**100.00**	

Supplementary Fig. 345 HPLC spectra of rac-**5ka**

Chromatogram

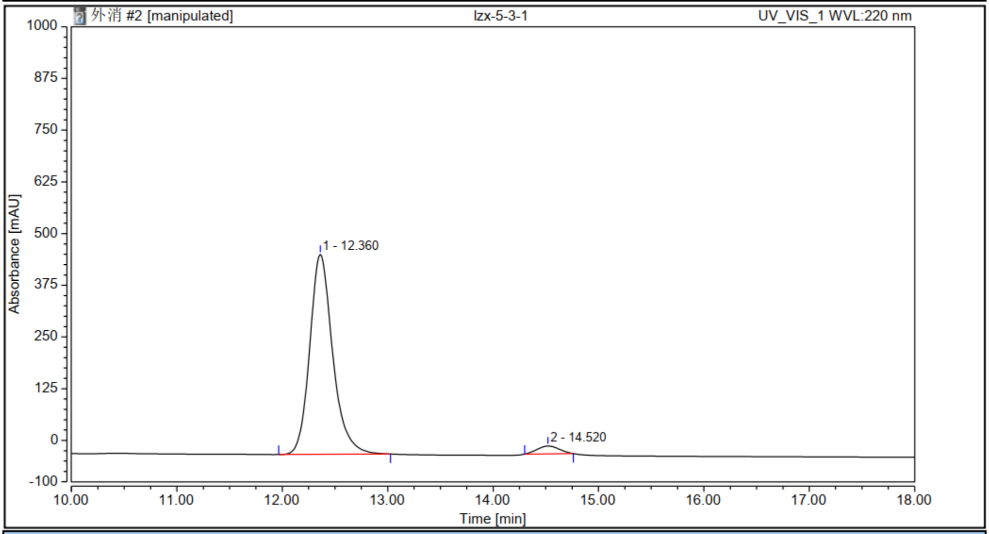

Integration Results

No.	Peak Name	Retention Time min	Area mAU*min	Height mAU	Relative Area %	Relative Height %	Amount n.a.
1		12.360	122.295	483.372	96.38	96.21	n.a.
2		14.520	4.597	19.054	3.62	3.79	n.a.
**Total:**			**126.892**	**502.426**	**100.00**	**100.00**	

Supplementary Fig. 346 HPLC spectra of **5ka**

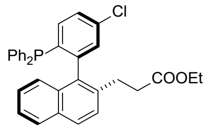

5la

Chromatogram

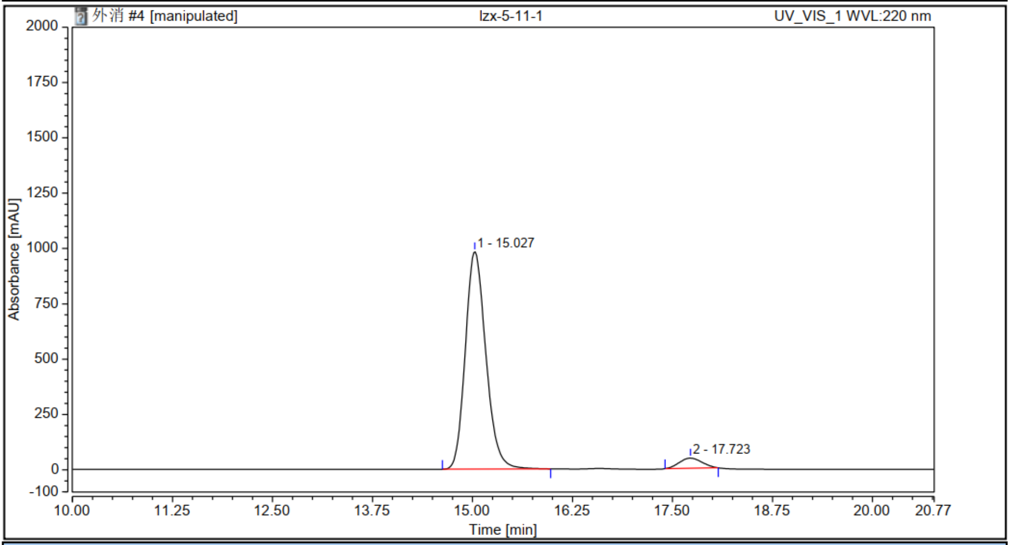

Integration Results

No.	Peak Name	Retention Time<br>min	Area<br>mAU*min	Height<br>mAU	Relative Area<br>%	Relative Height<br>%	Amount<br>n.a.
1		15.027	293.235	982.941	95.14	95.55	n.a.
2		17.723	14.995	45.786	4.86	4.45	n.a.
**Total:**			**308.230**	**1028.727**	**100.00**	**100.00**	

Supplementary Fig. 347 HPLC spectra of rac-**5la**

Chromatogram

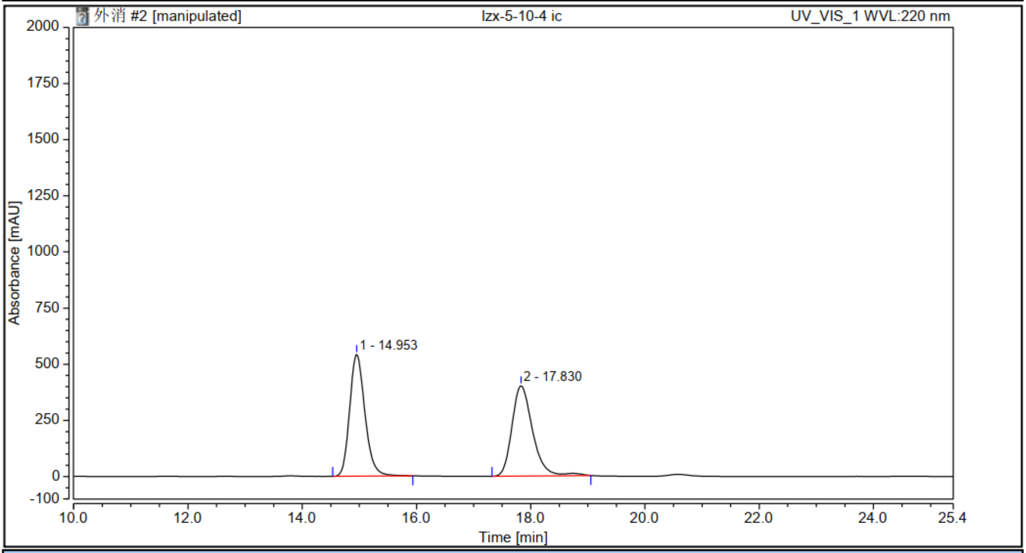

Integration Results

No.	Peak Name	Retention Time<br>min	Area<br>mAU*min	Height<br>mAU	Relative Area<br>%	Relative Height<br>%	Amount<br>n.a.
1		14.953	168.638	542.748	50.01	57.42	n.a.
2		17.830	168.554	402.539	49.99	42.58	n.a.
**Total:**			**337.191**	**945.287**	**100.00**	**100.00**	

Supplementary Fig. 348 HPLC spectra of **5la**

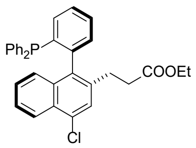

5ma

Chromatogram

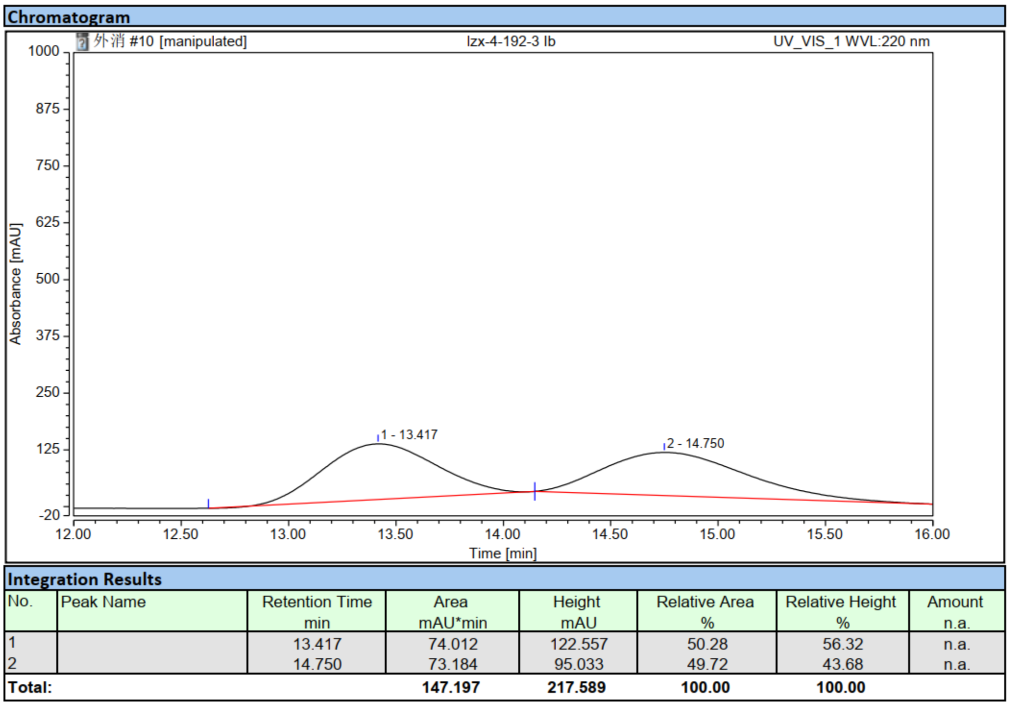

Integration Results

No.	Peak Name	Retention Time<br>min	Area<br>mAU*min	Height<br>mAU	Relative Area<br>%	Relative Height<br>%	Amount<br>n.a.
1		13.417	74.012	122.557	50.28	56.32	n.a.
2		14.750	73.184	95.033	49.72	43.68	n.a.
**Total:**			**147.197**	**217.589**	**100.00**	**100.00**	

Supplementary Fig. 349 HPLC spectra of rac-**5ma**

Chromatogram

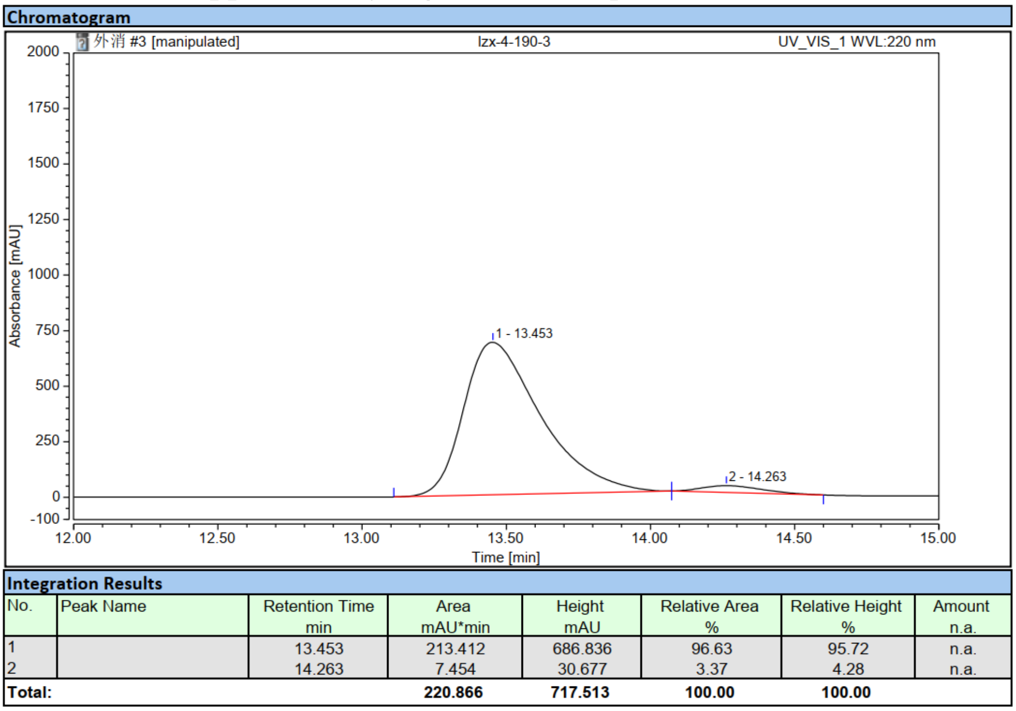

Integration Results

No.	Peak Name	Retention Time<br>min	Area<br>mAU*min	Height<br>mAU	Relative Area<br>%	Relative Height<br>%	Amount<br>n.a.
1		13.453	213.412	686.836	96.63	95.72	n.a.
2		14.263	7.454	30.677	3.37	4.28	n.a.
**Total:**			**220.866**	**717.513**	**100.00**	**100.00**	

Supplementary Fig. 350 HPLC spectra of **5ma**

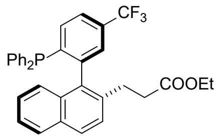

5na

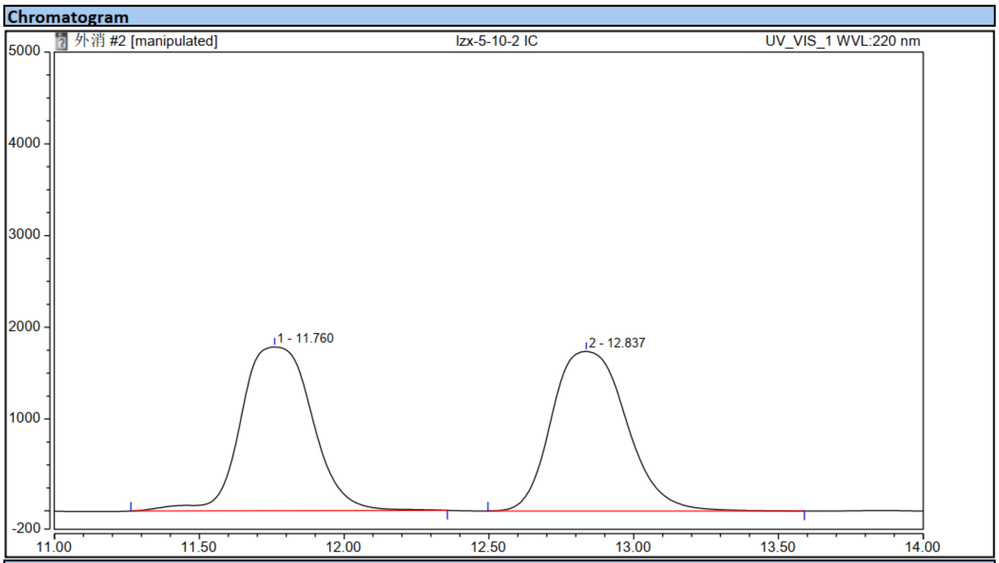

Integration Results

No.	Peak Name	Retention Time min	Area mAU*min	Height mAU	Relative Area %	Relative Height %	Amount n.a.
1		11.760	509.387	1784.461	49.54	50.65	n.a.
2		12.837	518.791	1738.615	50.46	49.35	n.a.
**Total:**			**1028.179**	**3523.076**	**100.00**	**100.00**	

Supplementary Fig. 351 HPLC spectra of rac-**5na**

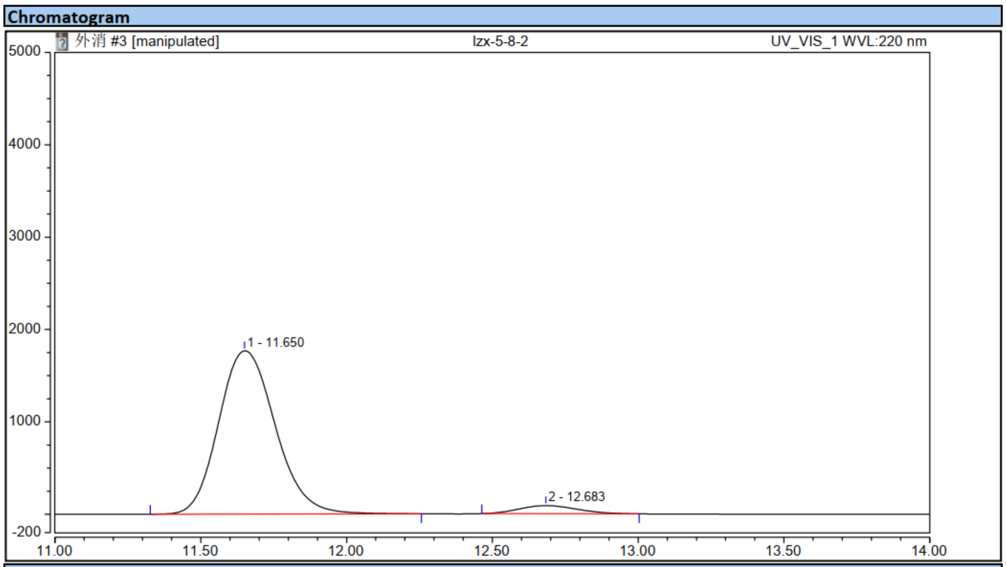

Integration Results

No.	Peak Name	Retention Time min	Area mAU*min	Height mAU	Relative Area %	Relative Height %	Amount n.a.
1		11.650	397.635	1766.202	95.15	95.28	n.a.
2		12.683	20.265	87.575	4.85	4.72	n.a.
**Total:**			**417.900**	**1853.777**	**100.00**	**100.00**	

Supplementary Fig. 352 HPLC spectra of **5na**

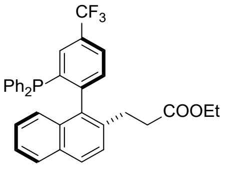

5oa

Chromatogram

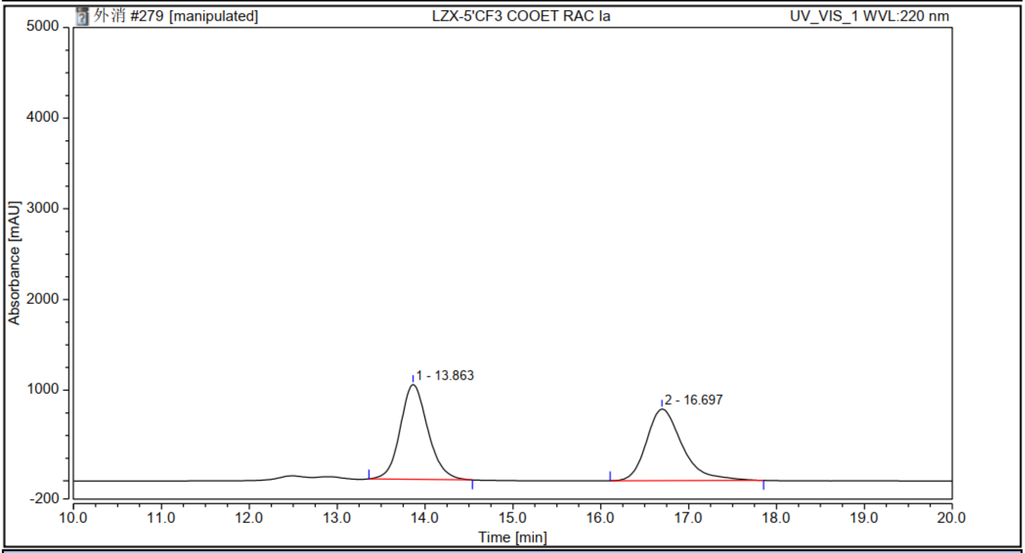

Integration Results

No.	Peak Name	Retention Time<br>min	Area<br>mAU*min	Height<br>mAU	Relative Area<br>%	Relative Height<br>%	Amount<br>n.a.
1		13.863	381.212	1046.577	50.57	57.01	n.a.
2		16.697	372.691	789.163	49.43	42.99	n.a.
**Total:**			**753.904**	**1835.739**	**100.00**	**100.00**	

Supplementary Fig. 353 HPLC spectra of rac-**5oa**

Chromatogram

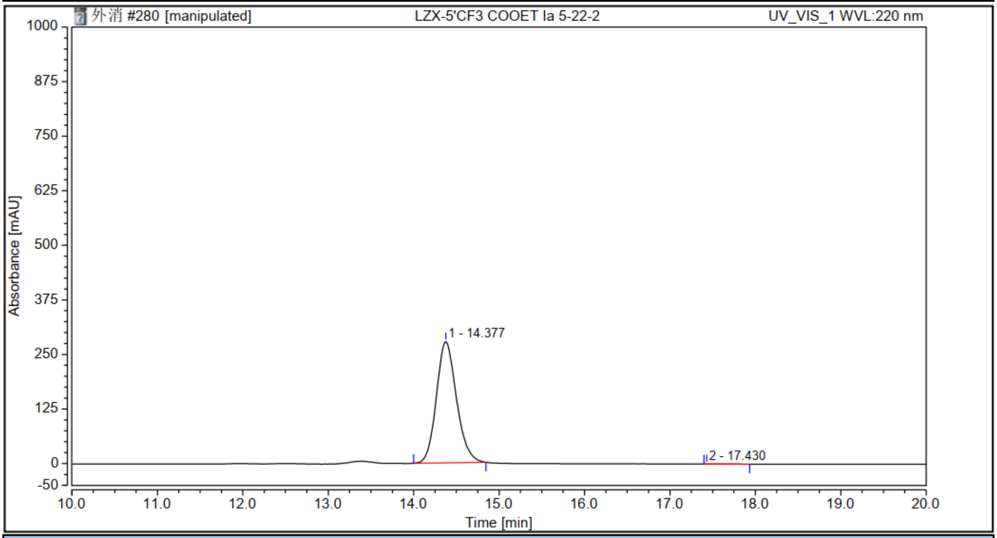

Integration Results

No.	Peak Name	Retention Time<br>min	Area<br>mAU*min	Height<br>mAU	Relative Area<br>%	Relative Height<br>%	Amount<br>n.a.
1		14.377	75.879	277.758	99.98	99.97	n.a.
2		17.430	0.015	0.072	0.02	0.03	n.a.
**Total:**			**75.894**	**277.830**	**100.00**	**100.00**	

Supplementary Fig. 354 HPLC spectra of **5oa**

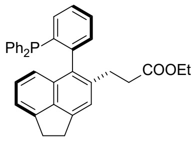

5pa

Chromatogram

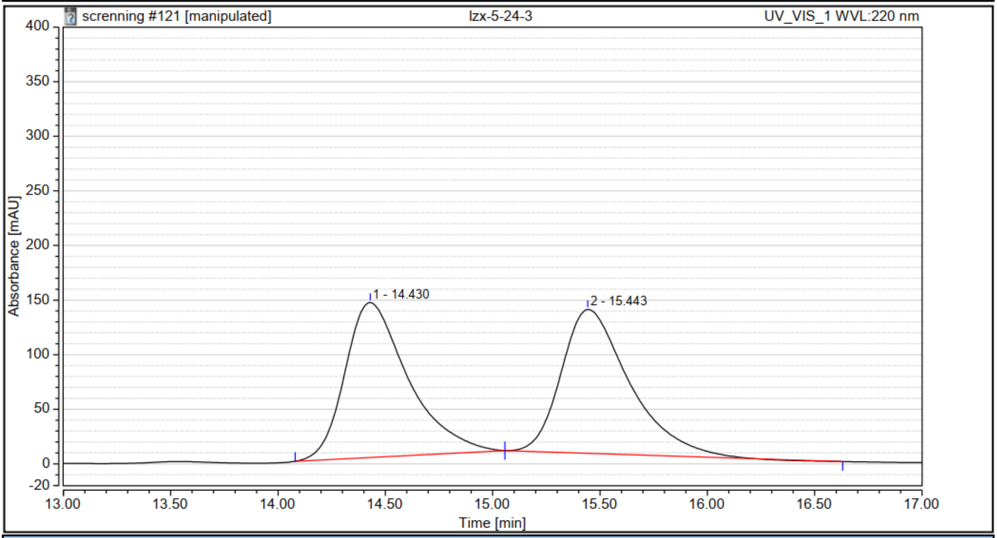

Integration Results

No.	Peak Name	Retention Time<br>min	Area<br>mAU*min	Height<br>mAU	Relative Area<br>%	Relative Height<br>%	Amount<br>n.a.
1		14.430	48.467	141.857	49.98	51.85	n.a.
2		15.443	48.513	131.708	50.02	48.15	n.a.
**Total:**			**96.980**	**273.565**	**100.00**	**100.00**	

Supplementary Fig. 355 HPLC spectra of rac-**5pa**

Chromatogram

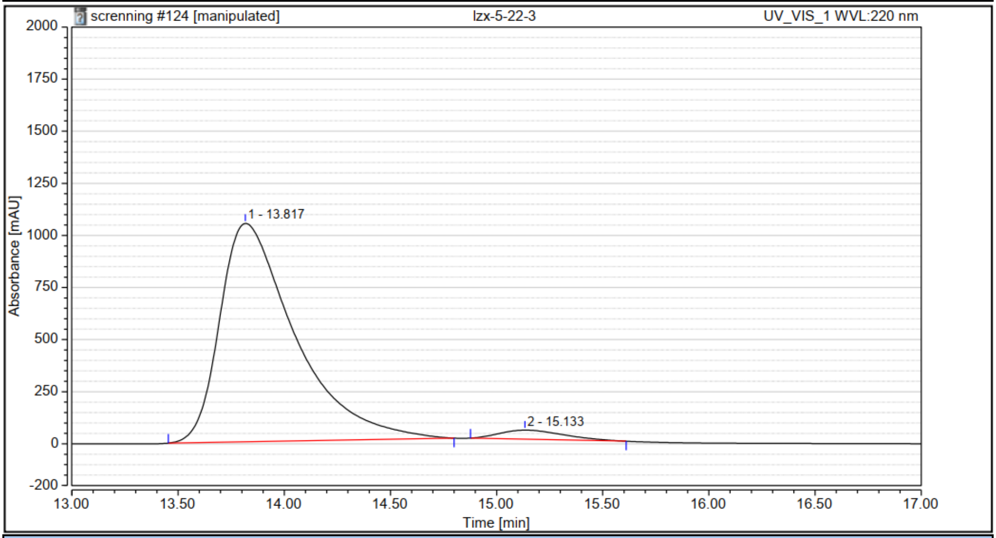

Integration Results

No.	Peak Name	Retention Time<br>min	Area<br>mAU*min	Height<br>mAU	Relative Area<br>%	Relative Height<br>%	Amount<br>n.a.
1		13.817	431.075	1048.125	96.59	96.02	n.a.
2		15.133	15.239	43.438	3.41	3.98	n.a.
**Total:**			**446.313**	**1091.563**	**100.00**	**100.00**	

Supplementary Fig. 356 HPLC spectra of **5pa**

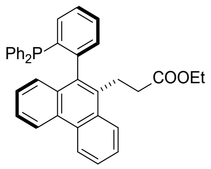

5qa

Chromatogram

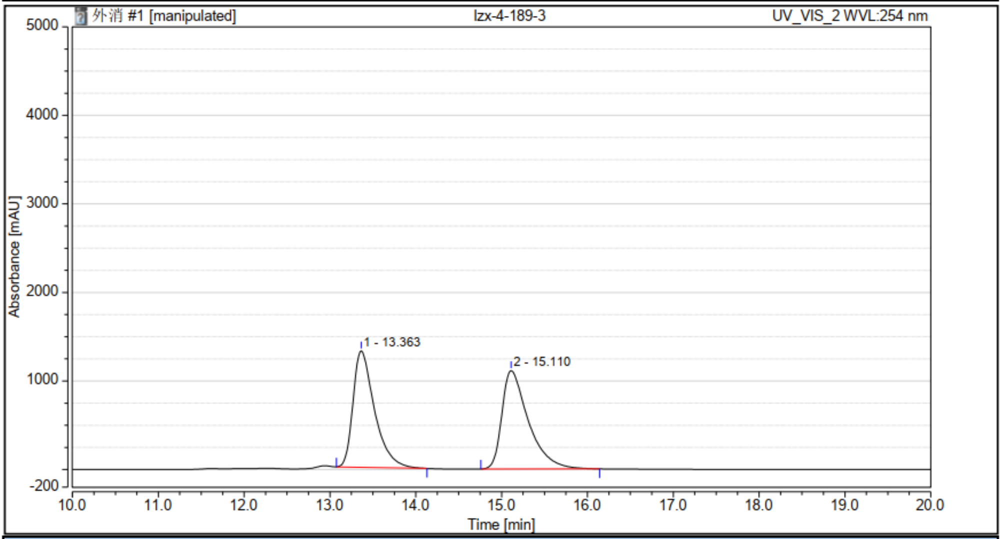

Integration Results

No.	Peak Name	Retention Time min	Area mAU*min	Height mAU	Relative Area %	Relative Height %	Amount n.a.
1		13.363	382.748	1317.777	49.05	54.22	n.a.
2		15.110	397.620	1112.461	50.95	45.78	n.a.
**Total:**			**780.368**	**2430.238**	**100.00**	**100.00**	

Supplementary Fig. 357 HPLC spectra of rac-**5qa**

Chromatogram

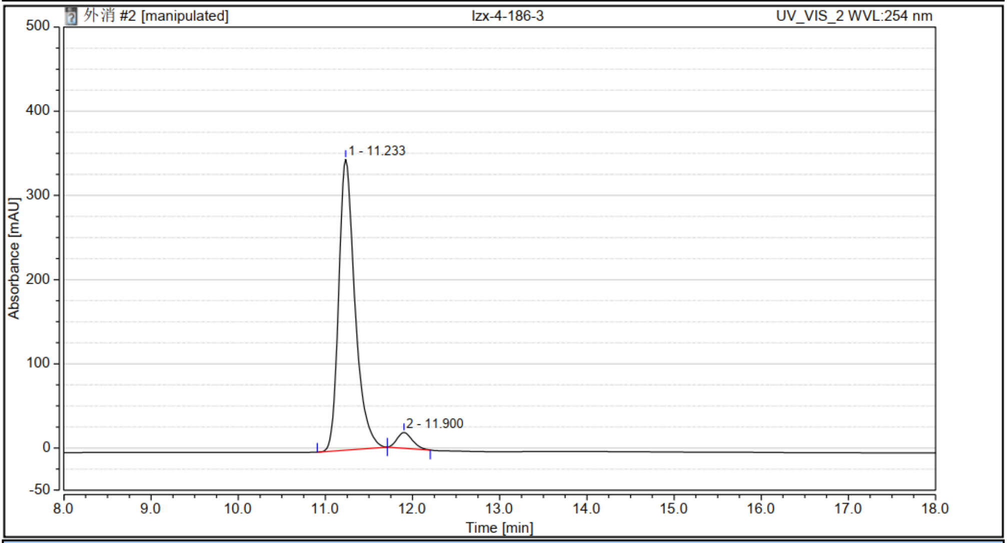

Integration Results

No.	Peak Name	Retention Time min	Area mAU*min	Height mAU	Relative Area %	Relative Height %	Amount n.a.
1		11.233	74.492	345.258	95.16	94.90	n.a.
2		11.900	3.788	18.550	4.84	5.10	n.a.
**Total:**			**78.281**	**363.808**	**100.00**	**100.00**	

Supplementary Fig. 358 HPLC spectra of **5qa**

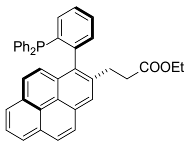

5ra

Chromatogram

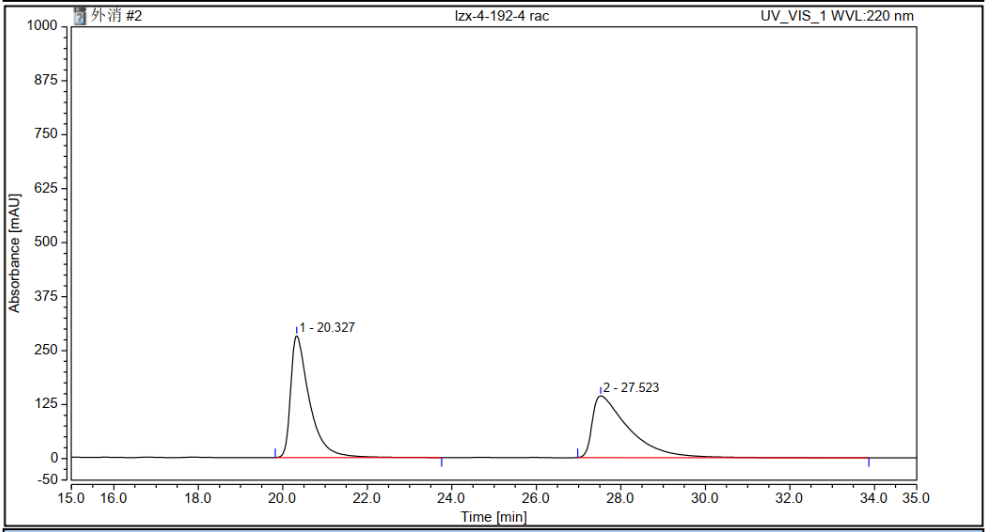

Integration Results

No.	Peak Name	Retention Time<br>min	Area<br>mAU*min	Height<br>mAU	Relative Area<br>%	Relative Height<br>%	Amount<br>n.a.
1		20.327	146.066	282.180	50.62	66.41	n.a.
2		27.523	142.488	142.710	49.38	33.59	n.a.
**Total:**			**288.554**	**424.890**	**100.00**	**100.00**	

Supplementary Fig. 359 HPLC spectra of rac-**5ra**

Chromatogram

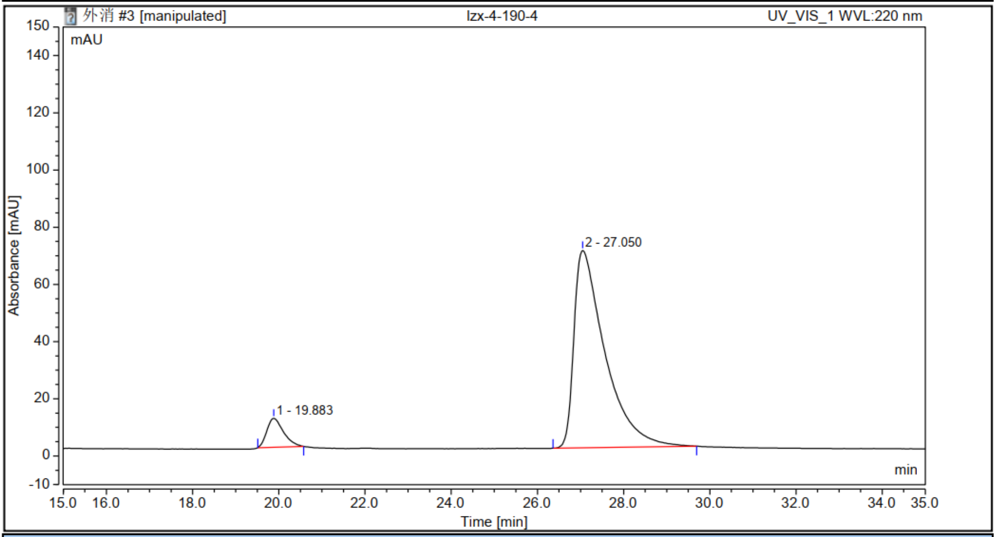

Integration Results

No.	Peak Name	Retention Time<br>min	Area<br>mAU*min	Height<br>mAU	Relative Area<br>%	Relative Height<br>%	Amount<br>n.a.
1		19.883	4.500	10.063	7.24	12.72	n.a.
2		27.050	57.653	69.035	92.76	87.28	n.a.
**Total:**			**62.153**	**79.098**	**100.00**	**100.00**	

Supplementary Fig. 360 HPLC spectra of **5ra**

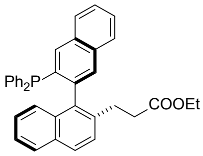

5sa

Chromatogram

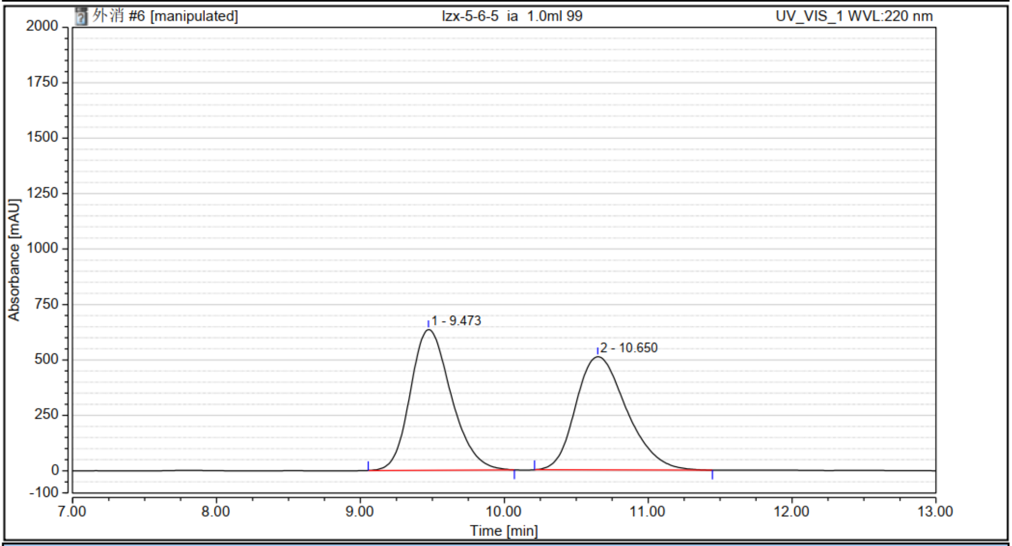

Integration Results

No.	Peak Name	Retention Time<br>min	Area<br>mAU*min	Height<br>mAU	Relative Area<br>%	Relative Height<br>%	Amount<br>n.a.
1		9.473	208.496	634.238	49.92	55.42	n.a.
2		10.650	209.123	510.284	50.08	44.58	n.a.
**Total:**			**417.619**	**1144.523**	**100.00**	**100.00**	

Supplementary Fig. 361 HPLC spectra of rac-**5sa**

Chromatogram

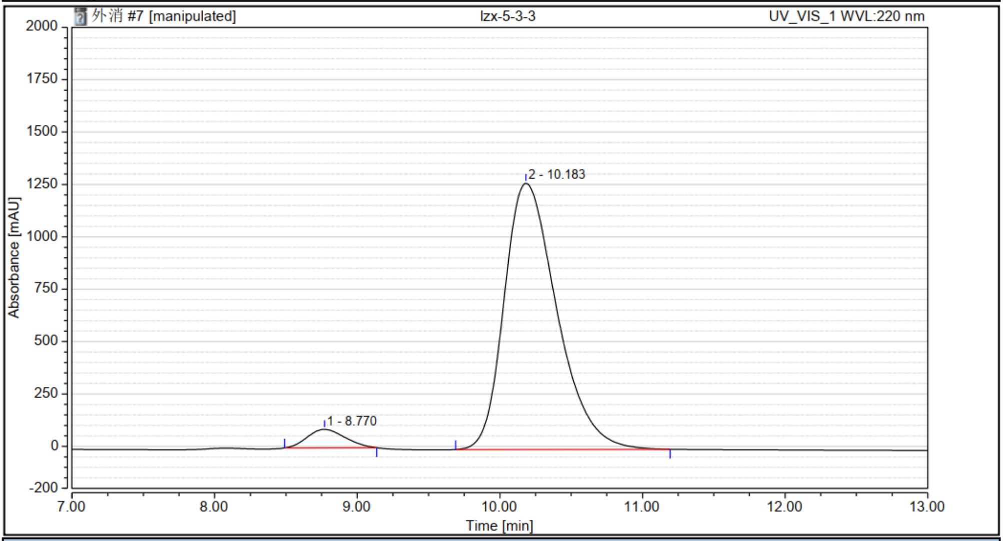

Integration Results

No.	Peak Name	Retention Time<br>min	Area<br>mAU*min	Height<br>mAU	Relative Area<br>%	Relative Height<br>%	Amount<br>n.a.
1		8.770	27.136	88.408	4.75	6.51	n.a.
2		10.183	543.637	1270.502	95.25	93.49	n.a.
**Total:**			**570.773**	**1358.910**	**100.00**	**100.00**	

Supplementary Fig. 362 HPLC spectra of **5sa**

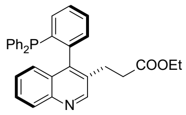

5ta

Chromatogram

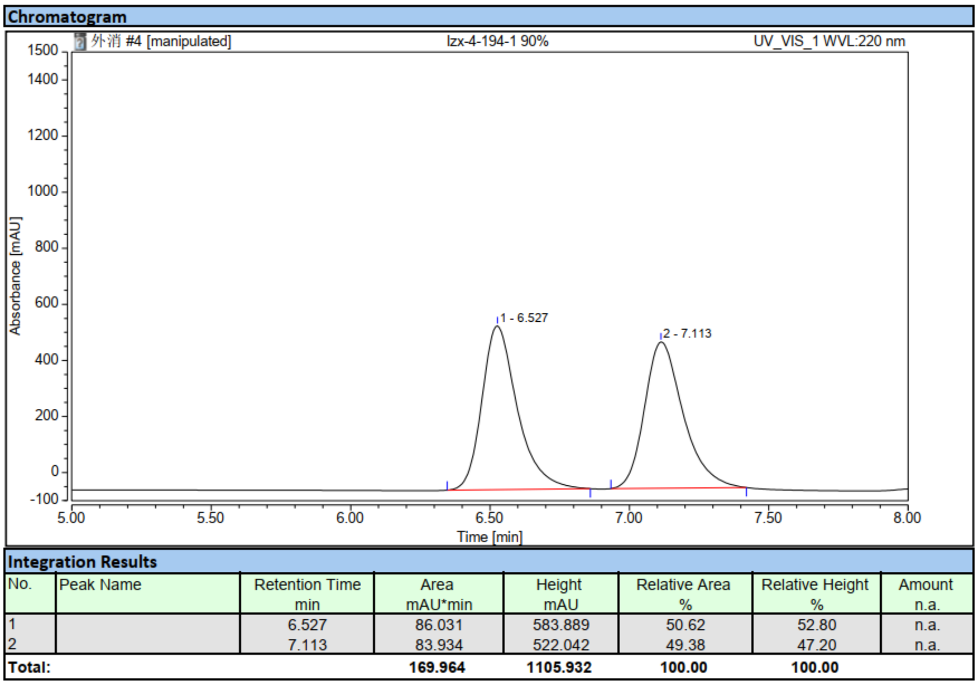

Integration Results

No.	Peak Name	Retention Time<br>min	Area<br>mAU*min	Height<br>mAU	Relative Area<br>%	Relative Height<br>%	Amount<br>n.a.
1		6.527	86.031	583.889	50.62	52.80	n.a.
2		7.113	83.934	522.042	49.38	47.20	n.a.
**Total:**			**169.964**	**1105.932**	**100.00**	**100.00**	

Supplementary Fig. 363 HPLC spectra of rac-**5ta**

Chromatogram

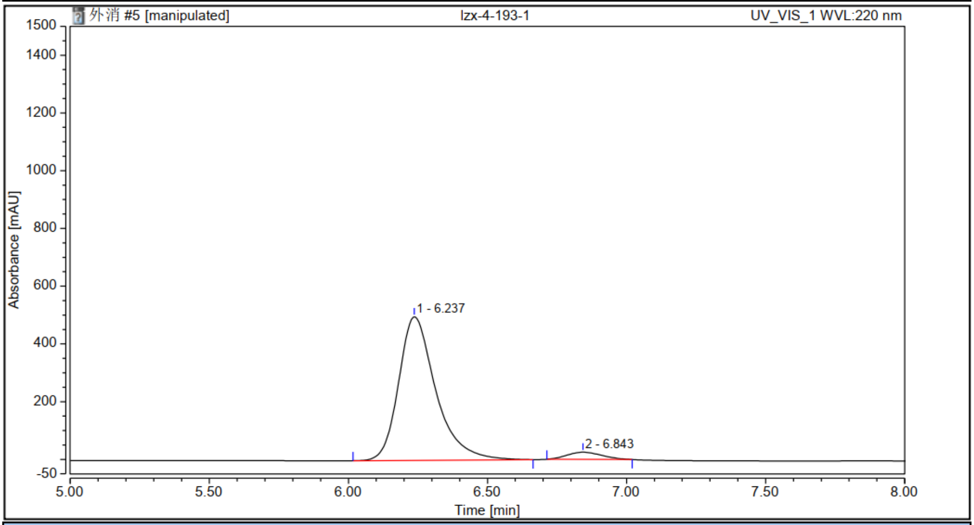

Integration Results

No.	Peak Name	Retention Time<br>min	Area<br>mAU*min	Height<br>mAU	Relative Area<br>%	Relative Height<br>%	Amount<br>n.a.
1		6.237	77.093	497.273	95.51	95.24	n.a.
2		6.843	3.623	24.865	4.49	4.76	n.a.
**Total:**			**80.716**	**522.137**	**100.00**	**100.00**	

Supplementary Fig. 364 HPLC spectra of **5ta**

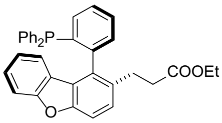

5ua

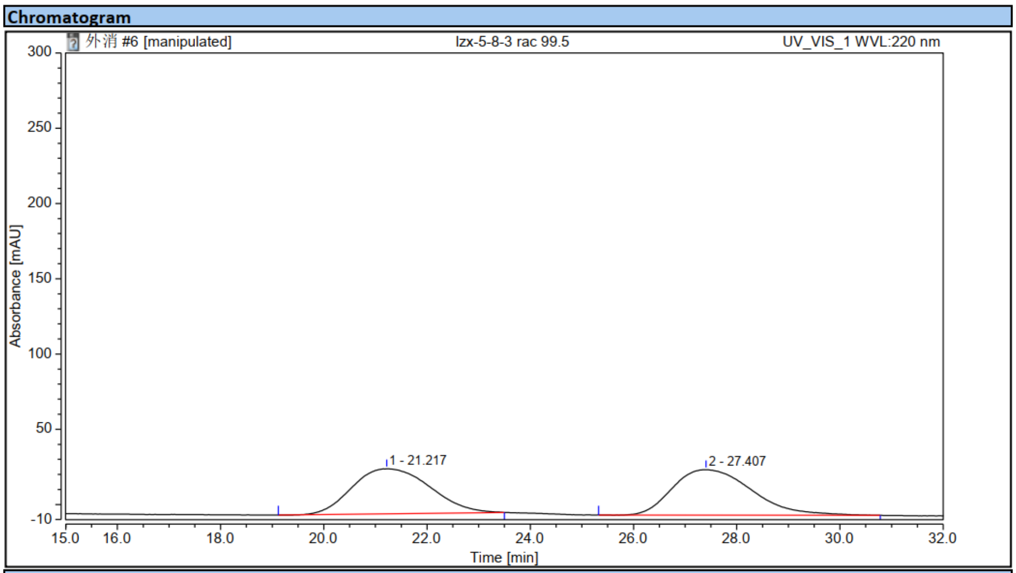

Integration Results

No.	Peak Name	Retention Time min	Area mAU*min	Height mAU	Relative Area %	Relative Height %	Amount n.a.
1		21.217	52.302	29.874	49.32	49.75	n.a.
2		27.407	53.736	30.169	50.68	50.25	n.a.
**Total:**			**106.037**	**60.043**	**100.00**	**100.00**	

Supplementary Fig. 365 HPLC spectra of rac-**5ua**

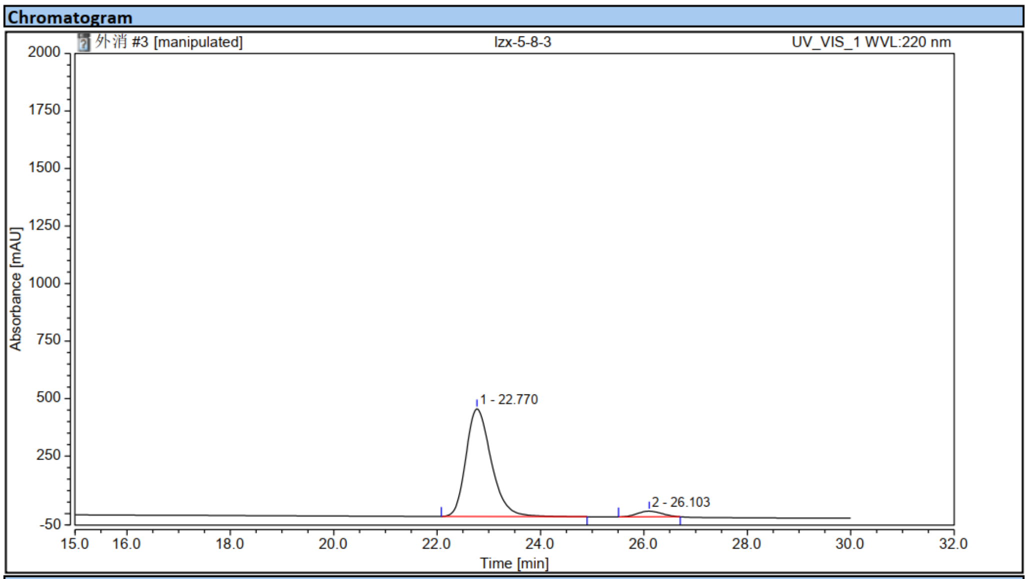

Integration Results

No.	Peak Name	Retention Time min	Area mAU*min	Height mAU	Relative Area %	Relative Height %	Amount n.a.
1		22.770	265.789	468.039	95.09	94.95	n.a.
2		26.103	13.738	24.915	4.91	5.05	n.a.
**Total:**			**279.527**	**492.954**	**100.00**	**100.00**	

Supplementary Fig. 366 HPLC spectra of **5ua**

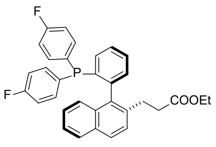

5wa

Chromatogram

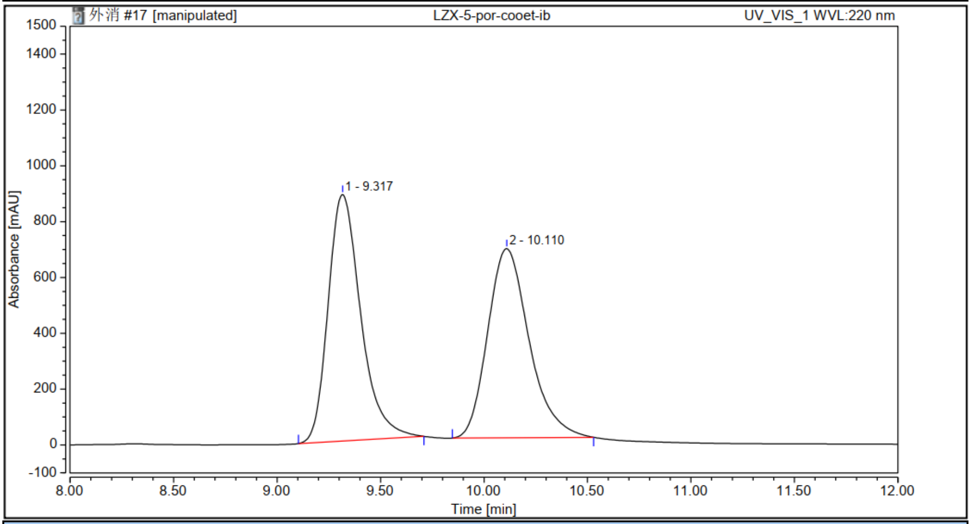

Integration Results

No.	Peak Name	Retention Time min	Area mAU*min	Height mAU	Relative Area %	Relative Height %	Amount n.a.
1		9.317	161.456	883.467	50.65	56.60	n.a.
2		10.110	157.320	677.493	49.35	43.40	n.a.
**Total:**			**318.776**	**1560.960**	**100.00**	**100.00**	

Supplementary Fig. 367 HPLC spectra of rac-**5wa**

Chromatogram

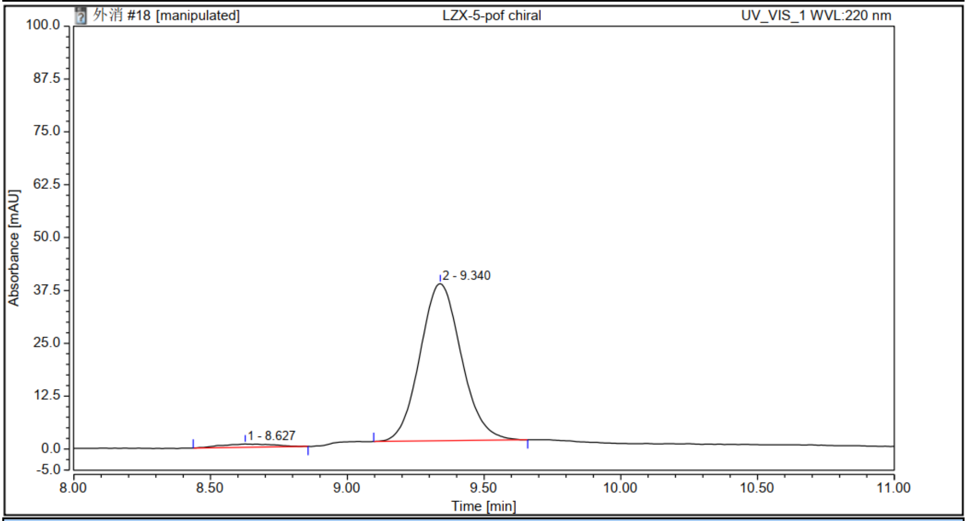

Integration Results

No.	Peak Name	Retention Time min	Area mAU*min	Height mAU	Relative Area %	Relative Height %	Amount n.a.
1		8.627	0.170	0.770	2.56	2.03	n.a.
2		9.340	6.491	37.145	97.44	97.97	n.a.
**Total:**			**6.662**	**37.915**	**100.00**	**100.00**	

Supplementary Fig. 368 HPLC spectra of **5wa**

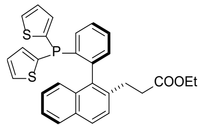

5xa

Chromatogram

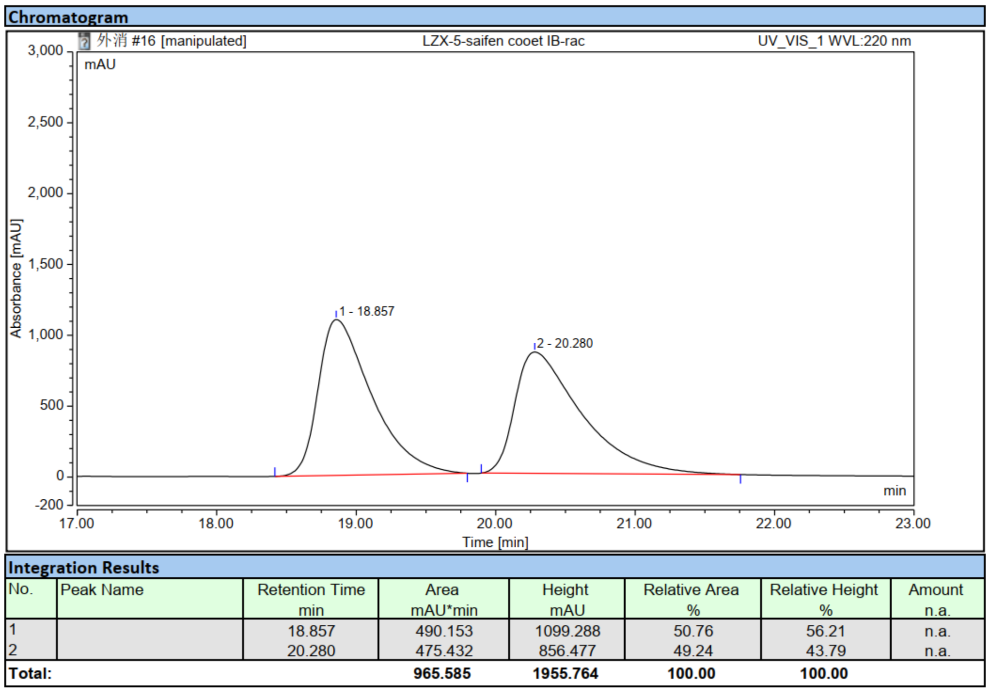

Integration Results

No.	Peak Name	Retention Time min	Area mAU*min	Height mAU	Relative Area %	Relative Height %	Amount n.a.
1		18.857	490.153	1099.288	50.76	56.21	n.a.
2		20.280	475.432	856.477	49.24	43.79	n.a.
**Total:**			**965.585**	**1955.764**	**100.00**	**100.00**	

Supplementary Fig. 369 HPLC spectra of rac-**5xa**

Chromatogram

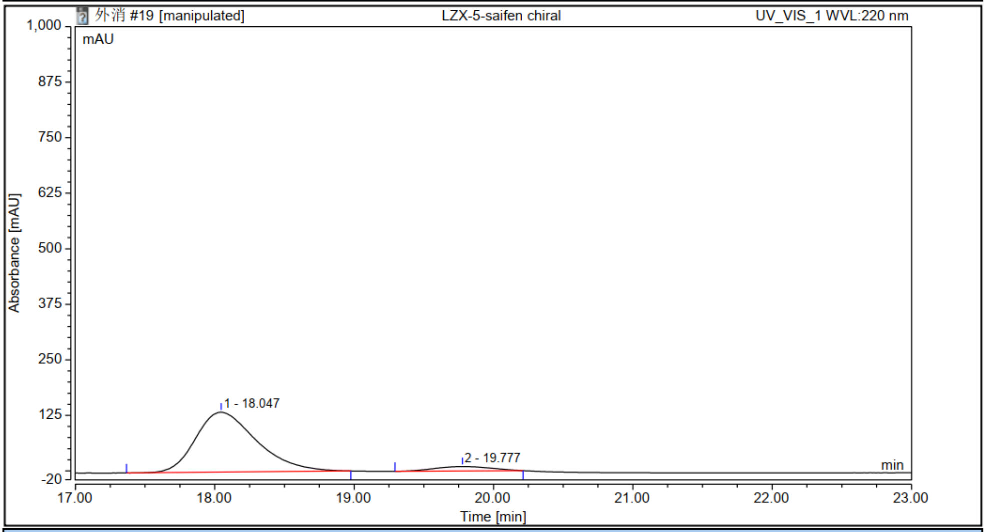

Integration Results

No.	Peak Name	Retention Time min	Area mAU*min	Height mAU	Relative Area %	Relative Height %	Amount n.a.
1		18.047	68.362	134.652	93.57	93.08	n.a.
2		19.777	4.702	10.014	6.43	6.92	n.a.
**Total:**			**73.064**	**144.666**	**100.00**	**100.00**	

Supplementary Fig. 370 HPLC spectra of **5xa**

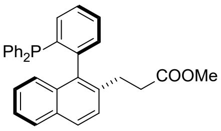

5ab

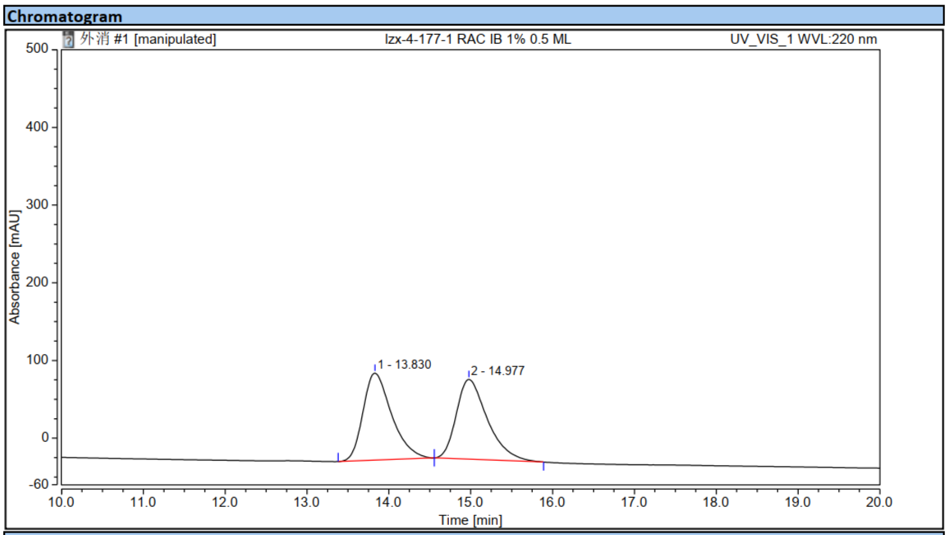

Integration Results

No.	Peak Name	Retention Time min	Area mAU*min	Height mAU	Relative Area %	Relative Height %	Amount n.a.
1		13.830	45.392	112.105	50.08	52.19	n.a.
2		14.977	45.252	102.704	49.92	47.81	n.a.
**Total:**			**90.645**	**214.809**	**100.00**	**100.00**	

Supplementary Fig. 371 HPLC spectra of rac-**5ab**

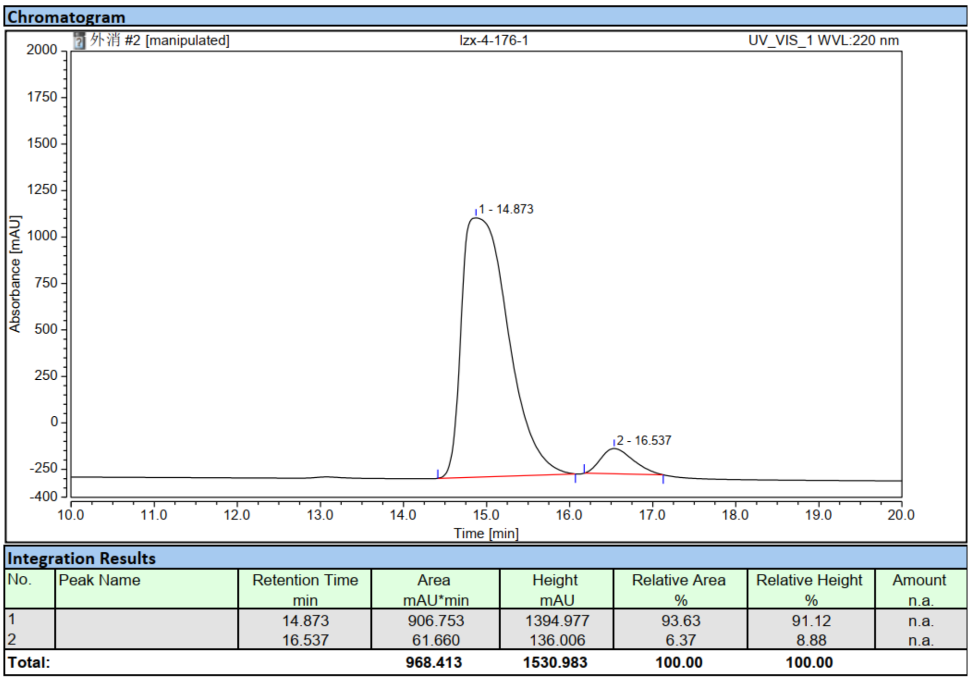

Integration Results

No.	Peak Name	Retention Time min	Area mAU*min	Height mAU	Relative Area %	Relative Height %	Amount n.a.
1		14.873	906.753	1394.977	93.63	91.12	n.a.
2		16.537	61.660	136.006	6.37	8.88	n.a.
**Total:**			**968.413**	**1530.983**	**100.00**	**100.00**	

Supplementary Fig. 372 HPLC spectra of **5ab**

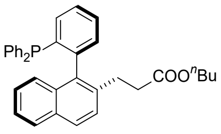

5ac

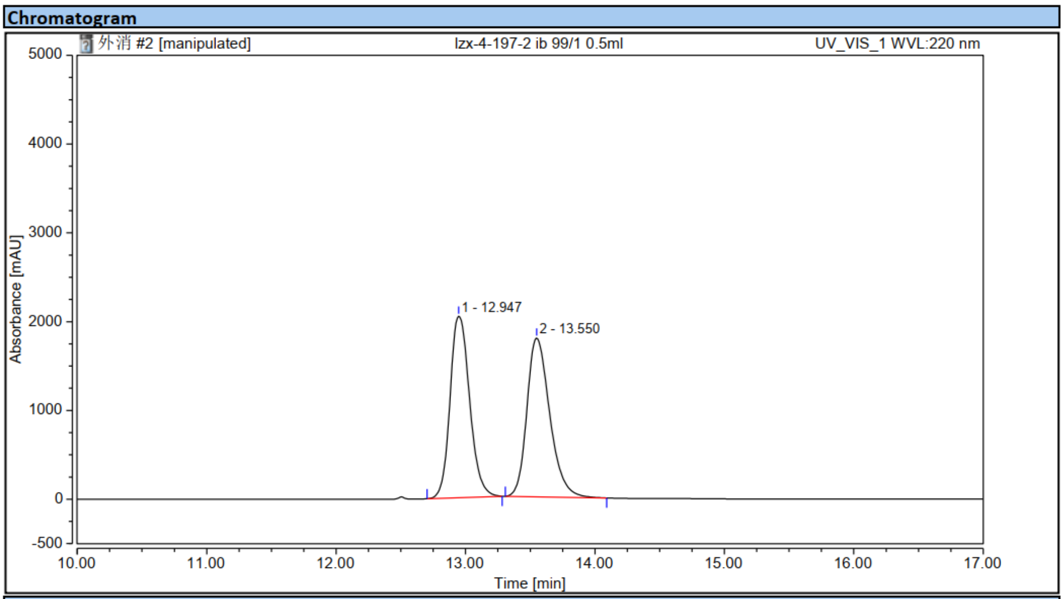

Integration Results

No.	Peak Name	Retention Time min	Area mAU*min	Height mAU	Relative Area %	Relative Height %	Amount n.a.
1		12.947	357.925	2045.172	49.47	53.36	n.a.
2		13.550	365.555	1787.672	50.53	46.64	n.a.
**Total:**			**723.481**	**3832.844**	**100.00**	**100.00**	

Supplementary Fig. 373 HPLC spectra of rac-**5ac**

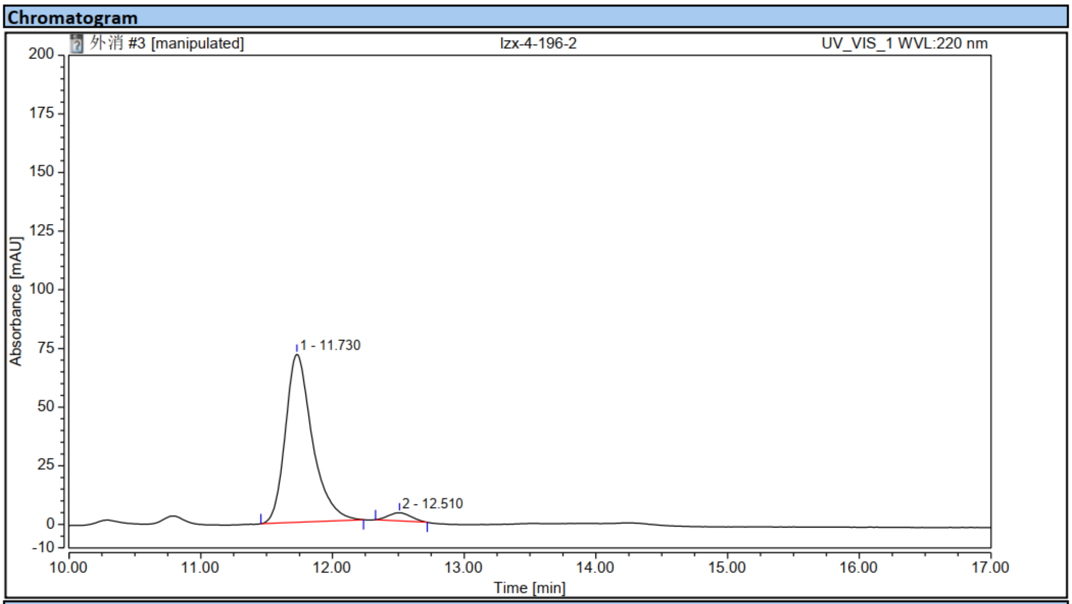

Integration Results

No.	Peak Name	Retention Time min	Area mAU*min	Height mAU	Relative Area %	Relative Height %	Amount n.a.
1		11.730	16.949	71.665	96.13	95.41	n.a.
2		12.510	0.683	3.448	3.87	4.59	n.a.
**Total:**			**17.632**	**75.113**	**100.00**	**100.00**	

Supplementary Fig. 374 HPLC spectra of **5ac**

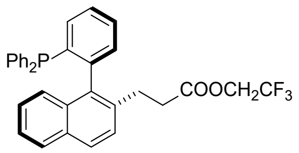

5ad

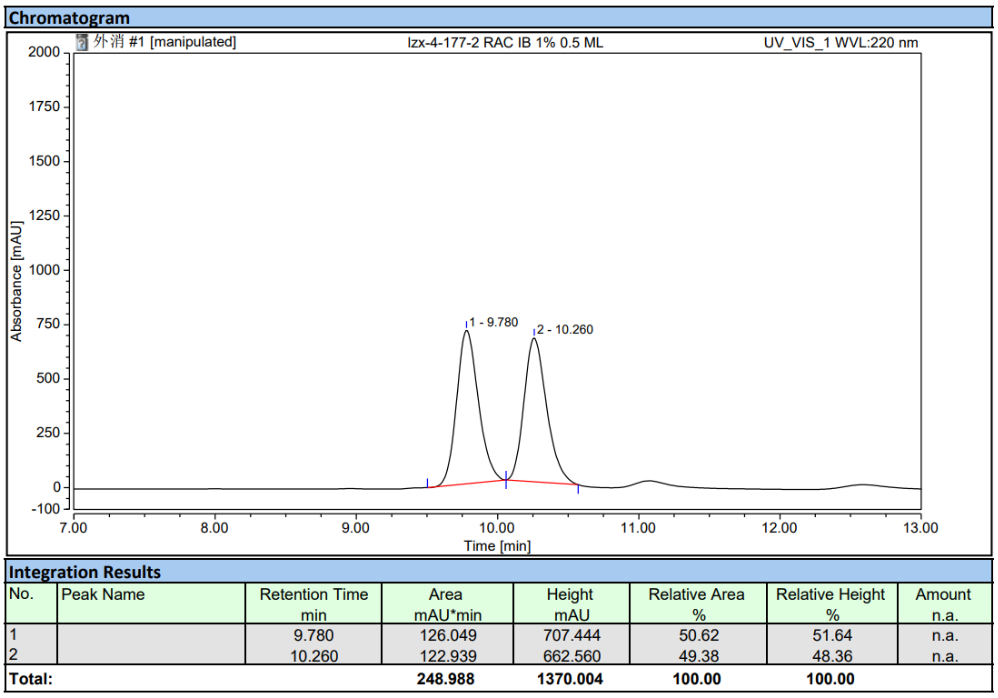

Integration Results

No.	Peak Name	Retention Time min	Area mAU*min	Height mAU	Relative Area %	Relative Height %	Amount n.a.
1		9.780	126.049	707.444	50.62	51.64	n.a.
2		10.260	122.939	662.560	49.38	48.36	n.a.
**Total:**			**248.988**	**1370.004**	**100.00**	**100.00**	

Supplementary Fig. 375 HPLC spectra of rac-**5ad**

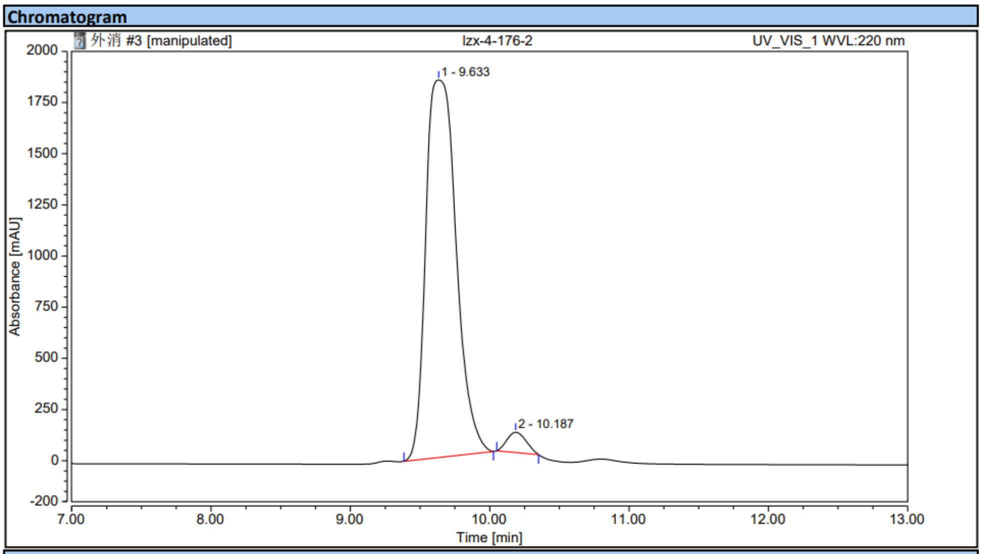

Integration Results

No.	Peak Name	Retention Time min	Area mAU*min	Height mAU	Relative Area %	Relative Height %	Amount n.a.
1		9.633	466.545	1844.480	96.71	94.91	n.a.
2		10.187	15.868	98.909	3.29	5.09	n.a.
**Total:**			**482.413**	**1943.389**	**100.00**	**100.00**	

Supplementary Fig. 376HPLC spectra of **5ad**

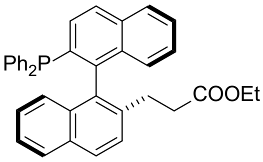

5ya

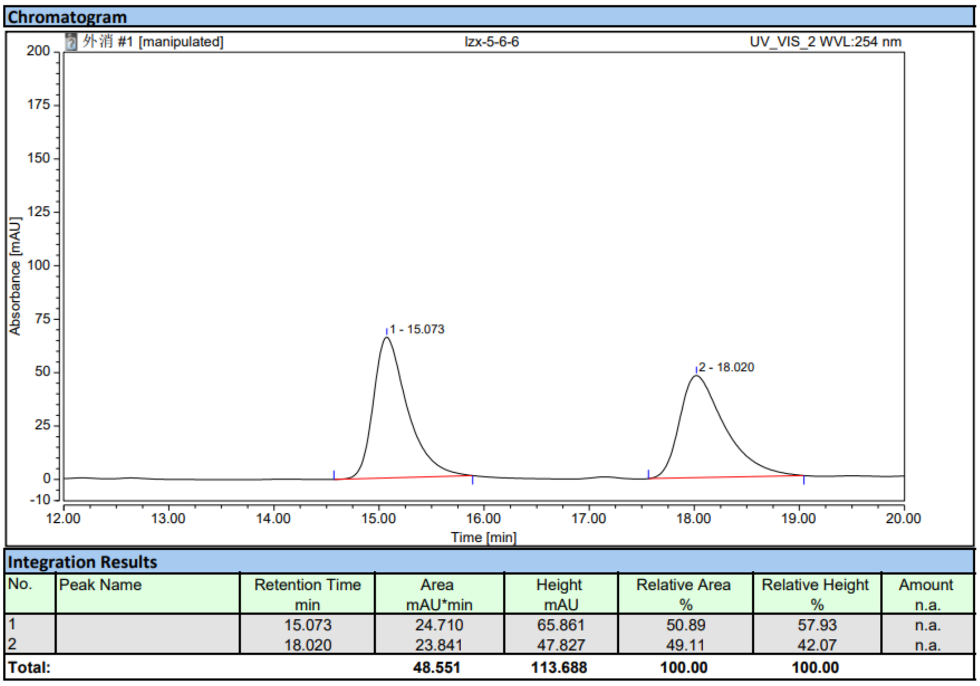

Integration Results

No.	Peak Name	Retention Time min	Area mAU*min	Height mAU	Relative Area %	Relative Height %	Amount n.a.
1		15.073	24.710	65.861	50.89	57.93	n.a.
2		18.020	23.841	47.827	49.11	42.07	n.a.
**Total:**			**48.551**	**113.688**	**100.00**	**100.00**	

Supplementary Fig. 377 HPLC spectra of rac-**5ya**

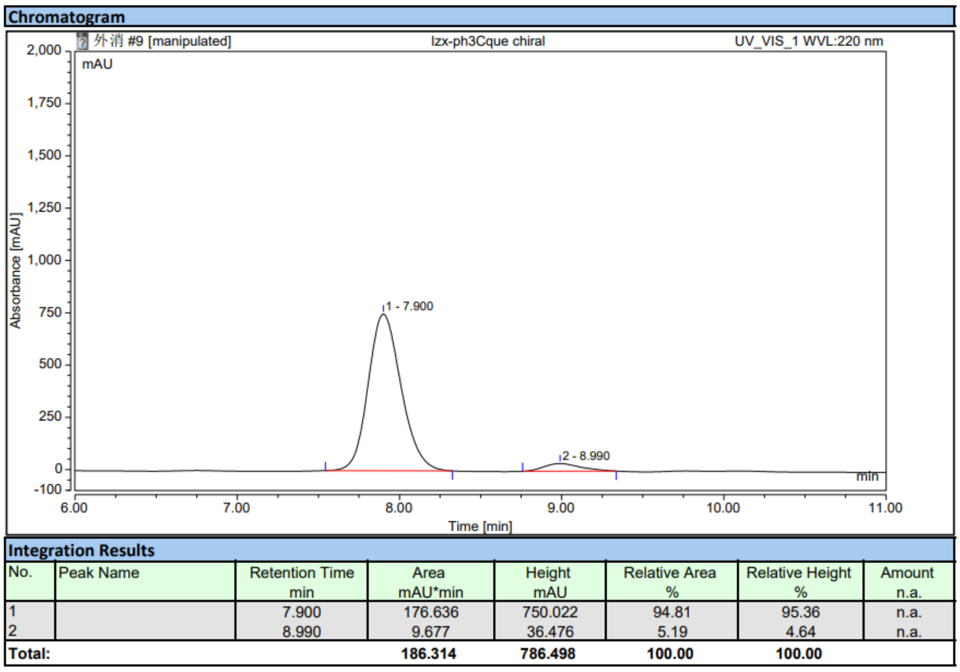

Integration Results

No.	Peak Name	Retention Time min	Area mAU*min	Height mAU	Relative Area %	Relative Height %	Amount n.a.
1		7.900	176.636	750.022	94.81	95.36	n.a.
2		8.990	9.677	36.476	5.19	4.64	n.a.
**Total:**			**186.314**	**786.498**	**100.00**	**100.00**	

Supplementary Fig. 378 HPLC spectra of **5ya**

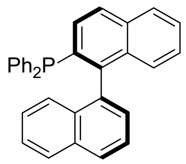

1y

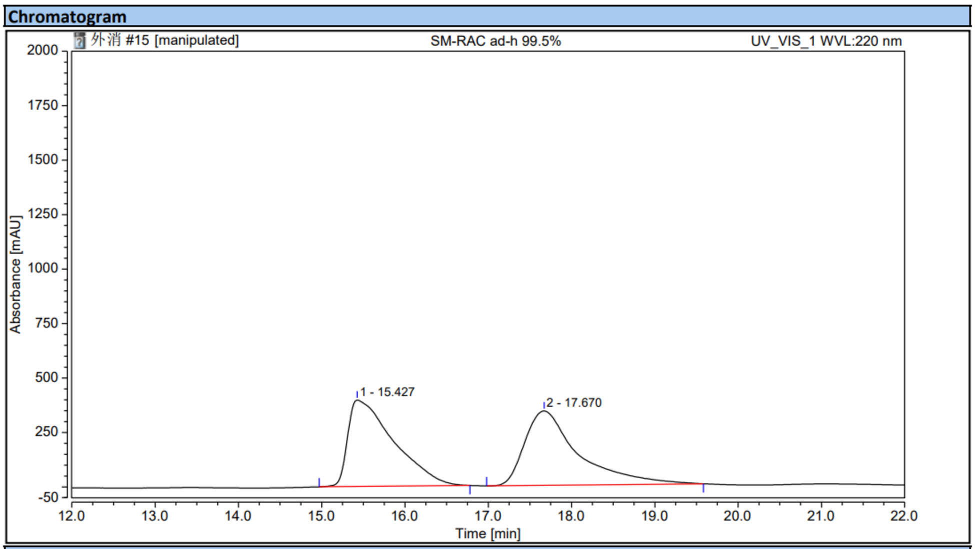

Integration Results

No.	Peak Name	Retention Time min	Area mAU*min	Height mAU	Relative Area %	Relative Height %	Amount n.a.
1		15.427	252.959	397.324	49.88	53.81	n.a.
2		17.670	254.166	341.007	50.12	46.19	n.a.
**Total:**			**507.126**	**738.331**	**100.00**	**100.00**	

Supplementary Fig. 379 HPLC spectra of rac-**1y**

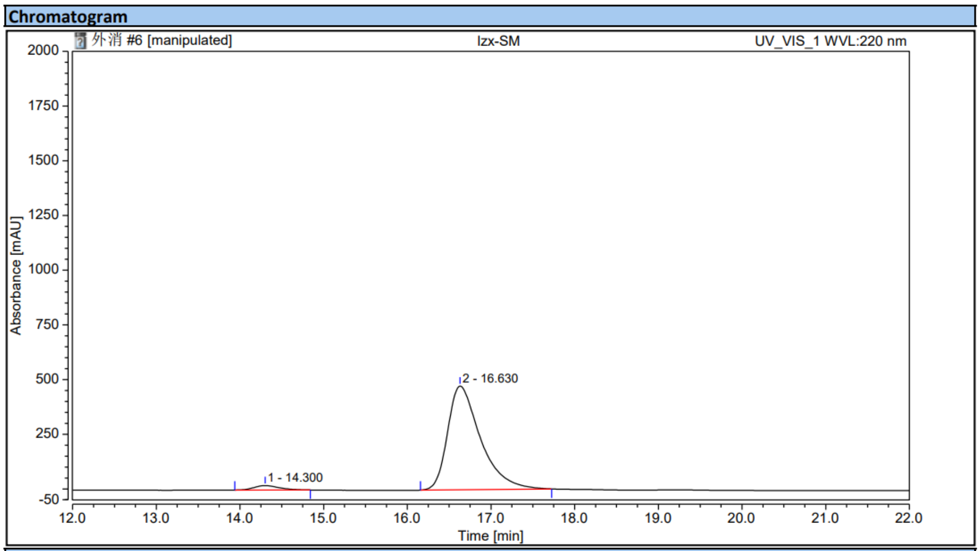

Integration Results

No.	Peak Name	Retention Time min	Area mAU*min	Height mAU	Relative Area %	Relative Height %	Amount n.a.
1		14.300	6.990	20.118	3.13	4.07	n.a.
2		16.630	216.315	473.921	96.87	95.93	n.a.
**Total:**			**223.305**	**494.039**	**100.00**	**100.00**	

Supplementary Fig. 380 HPLC spectra of **1y**

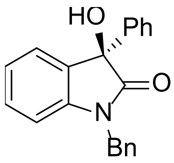

8

Chromatogram

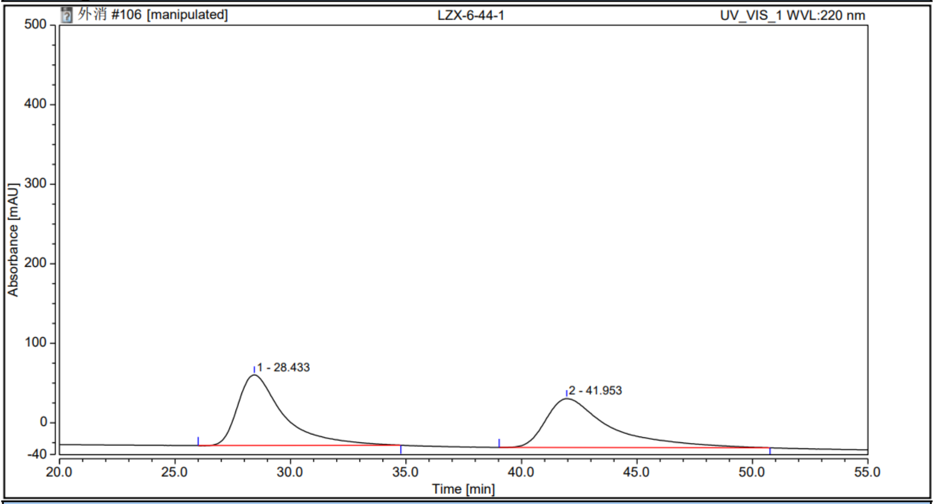

Integration Results

No.	Peak Name	Retention Time<br>min	Area<br>mAU*min	Height<br>mAU	Relative Area<br>%	Relative Height<br>%	Amount<br>n.a.
1		28.433	203.328	88.742	50.64	59.07	n.a.
2		41.953	198.161	61.479	49.36	40.93	n.a.
**Total:**			**401.489**	**150.221**	**100.00**	**100.00**	

Supplementary Fig. 381 HPLC spectra of rac-**8**

Chromatogram

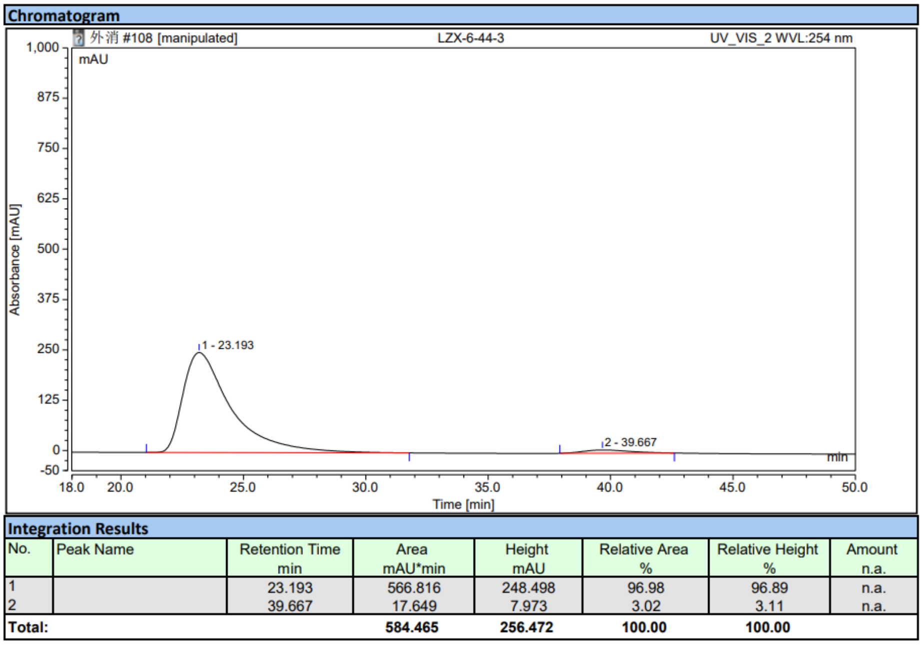

Integration Results

No.	Peak Name	Retention Time<br>min	Area<br>mAU*min	Height<br>mAU	Relative Area<br>%	Relative Height<br>%	Amount<br>n.a.
1		23.193	566.816	248.498	96.98	96.89	n.a.
2		39.667	17.649	7.973	3.02	3.11	n.a.
**Total:**			**584.465**	**256.472**	**100.00**	**100.00**	

Supplementary Fig. 382 HPLC spectra of **8**

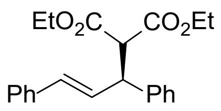

11

Chromatogram

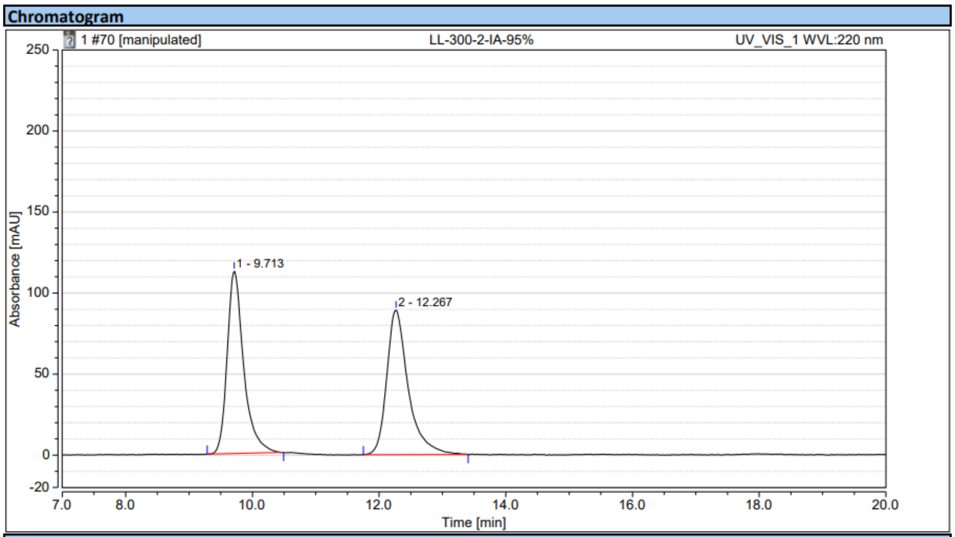

Integration Results

No.	Peak Name	Retention Time min	Area mAU*min	Height mAU	Relative Area %	Relative Height %	Amount n.a.
1		9.713	34.013	112.644	49.26	55.78	n.a.
2		12.267	35.039	89.288	50.74	44.22	n.a.
**Total:**			**69.052**	**201.932**	**100.00**	**100.00**	

Supplementary Fig. 383 HPLC spectra of rac-**11**

Chromatogram

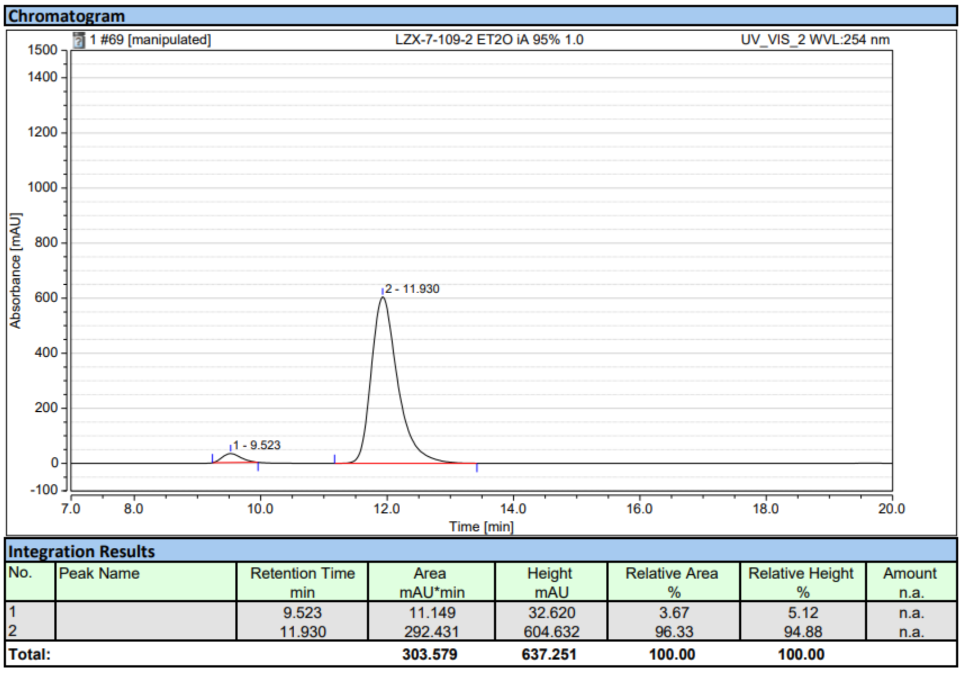

Integration Results

No.	Peak Name	Retention Time min	Area mAU*min	Height mAU	Relative Area %	Relative Height %	Amount n.a.
1		9.523	11.149	32.620	3.67	5.12	n.a.
2		11.930	292.431	604.632	96.33	94.88	n.a.
**Total:**			**303.579**	**637.251**	**100.00**	**100.00**	

Supplementary Fig. 384 HPLC spectra of **11**

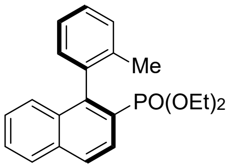

14

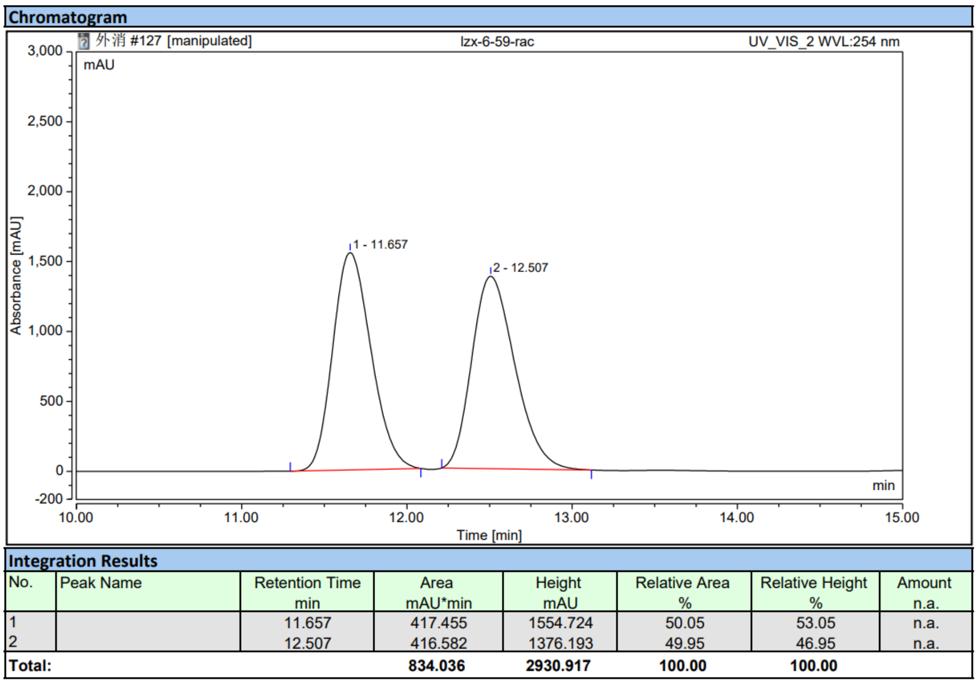

Integration Results

No.	Peak Name	Retention Time min	Area mAU*min	Height mAU	Relative Area %	Relative Height %	Amount n.a.
1		11.657	417.455	1554.724	50.05	53.05	n.a.
2		12.507	416.582	1376.193	49.95	46.95	n.a.
**Total:**			**834.036**	**2930.917**	**100.00**	**100.00**	

Supplementary Fig. 385 HPLC spectra of rac-**14**

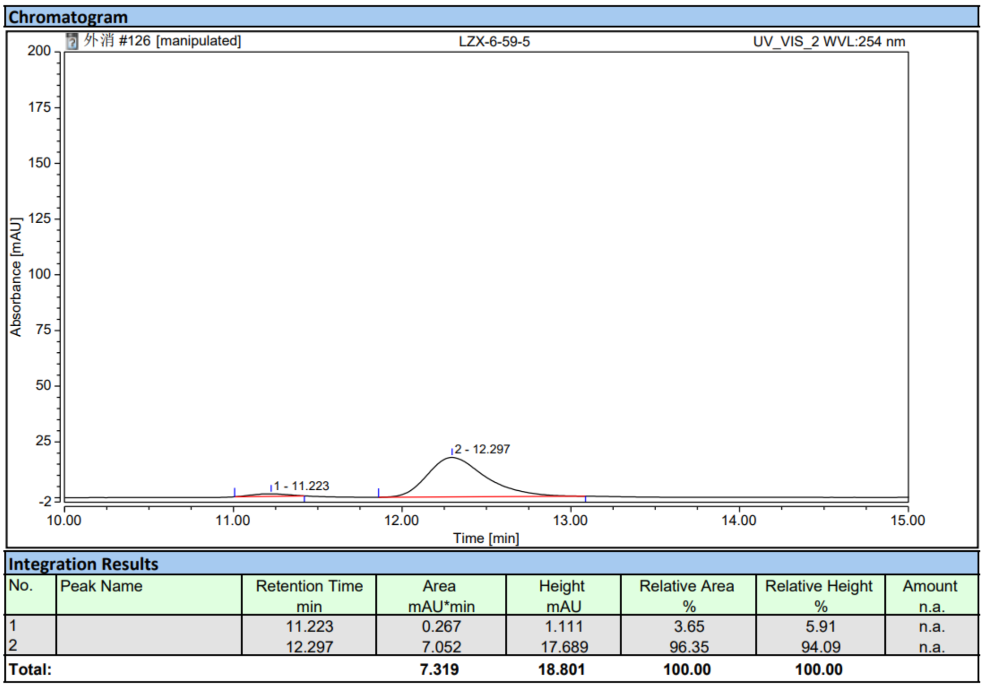

Integration Results

No.	Peak Name	Retention Time min	Area mAU*min	Height mAU	Relative Area %	Relative Height %	Amount n.a.
1		11.223	0.267	1.111	3.65	5.91	n.a.
2		12.297	7.052	17.689	96.35	94.09	n.a.
**Total:**			**7.319**	**18.801**	**100.00**	**100.00**	

Supplementary Fig. 386 HPLC spectra of **14**

3. Supplementary References

[1] Luo, X.; Zhang, H.; Duan, H.; Liu, Q.; Zhu, L.; Zhang, T.; Lei, A. *Org. Lett.* **2007**, *9*, 4571.

[2] Baba, K.; Tobisu, M.; Chatani, N. *Angew. Chem. Int. Ed.* **2013**, *52*, 11892.

[3] Tan, X.; Zeng, W.; Zhang, X.; Chung, L. W.; Zhang, X. *Chem. Commun.* **2018**, *54*, 535.

[4] Ma, H.-C.; Jiang, X.-Z. *J. Org. Chem.* **2007**, *72*, 8943.

[5] Liu, Z.-S.; Hua, Y.; Gao, Q.; Ma, Y.; Tang, H.; Shang, Y.; Cheng, H.-G.; Zhou, Q. *Nat. Catal*. **2020**, *3*, 727.

[6] Uozumi, Y.; Suzuki, N.; Ogiwara, A.; Hayashi, T. *Tetrahedron* **1994**, *50*, 4293.

[7] Kagan, H. B.; Fiaud, J. C. *Top. Stereochem*. **1988**, *18*, 249.

[8] Wang, D., Dong, B., Wang, Y., Qian, J., Zhu, J., Zhao, Y. and Shi, Z. *Nat. Commun.* **2019**, *10*, 3539.

[9] Li, Z.; Chen, Y.; Wang, C.; Xu, G.; Shao, Y.; Zhang, X.; Tang, S.; Sun, J. *Angew. Chem. Int. Ed.* **2021**, *60*, 25714-25718.

[10] Wu, W., Wang, S., Zhou, Y., He, Y., Zhuang, Y., Li, L., Wan, P., Wang, L., Zhou, Z. and Qiu, L. *Adv. Synth. Catal.* **2012**, *354*, 2395-2402.

[11] Gaussian 09, Revision E.01, Frisch, M. J.; Trucks, G. W.; Schlegel, H. B.; Scuseria, G. E.; Robb, M. A.; Cheeseman, J. R.; Scalmani, G.; Barone, V.; Mennucci, B.; Petersson, G. A.; Nakatsuji, H.; Caricato, M.; Li, X.; Hratchian, H. P.; Izmaylov, A. F.; Bloino, J.; Zheng, G.; Sonnenberg, J. L.; Hada, M.; Ehara, M.; Toyota, K.; Fukuda, R.; Hasegawa, J.; Ishida, M.; Nakajima, T.; Honda, Y.; Kitao, O.; Nakai, H.; Vreven, T.; Montgomery, J. A.; Peralta, Jr., J. E.; Ogliaro, F.; Bearpark, M.; Heyd, J. J.; Brothers, E.; Kudin, K. N.; Staroverov, V. N.; Keith, T.; Kobayashi, R.; Normand, J.; Raghavachari, K.; Rendell, A.; Burant, J.

C.; Iyengar, S. S.; Tomasi, J.; Cossi, M.; Rega, N.; Millam, J. M.; Klene, M.; Knox, J. E.; Cross, J. B.; Bakken, V.; Adamo, C.; Jaramillo, J.; Gomperts, R.; Stratmann, R. E.; Yazyev, O.; Austin, A. J.; Cammi, R.; Pomelli, C.; Ochterski, J. W.; Martin, R. L.; Morokuma, K.; Zakrzewski, V. G.; Voth, G. A.; Salvador, P.; Dannenberg, J. J.; Dapprich, S.; Daniels, A. D.; Farkas, O.; Foresman, J. B.; Ortiz, J. V.; Cioslowski, J.; and Fox, D. J. Gaussian, Inc., Wallingford CT, **2013**.

[12] a) Becke, A. D. *J. Chem. Phys.* **1993**, 98, 5648. b) Becke, A. D.; Johnson, E. R. *J. Chem. Phys.* **2005** 123, 154101. c) Becke, A. D. *Phys. Rev. A.* **1988**, *38*, 3098. d) Grimme, S.; Ehrlich, S.; Goerigk, L. *J. Comput. Chem.* **2011**, *32*, 1456.

[13] Hay, P. J.; Wadt, W. R. *J. Chem. Phys.*, **1985**, *82*, 299.

[14] a) Ditchfield, R.; Hehre, W. J.; Pople, J. A. *J. Chem. Phys.* **1971**, *54*, 724. b) Hehre, W. J.; Ditchfield, R.; Pople, J. A. *J. Chem. Phys.* **1972**, *56*, 2257. c) Hariharan, P. C.; Pople, J. A. *Theor. Chem. Acc.* **1973**, *28*, 213.

[15] a) Barone, V.; Cossi, M. *J. Phys. Chem. A* **1998**, *102*, 1995. b) Cossi, M.; Rega, N.; Scalmani, G.; Barone, V. *J. Comput. Chem.* **2003**, *24*, 669.

[16] a) Clark, T.; Chandrasekhar, J.; Spitznagel, G. W.; Schleyer, P. Von R. *J. Comput. Chem.* **1983**, *4*, 294. b) Krishnan, R.; Binkley, J. S.; Seeger, R.; Pople, J. A. *J. Chem. Phys.* **1980**, *72*, 650.

[17] a) Dolg, M.; Wedig, U.; Stoll, H.; Preuss, H. *J. Chem. Phys.* **1987**, *86*, 866. b) Nicklass, A.; Dolg, M.; Stoll, H.; Preuss, H. *J. Chem. Phys.* **1995**, *102*, 8942.

[18] Legault, C. Y. CYL View, version 1.0 b; Universite de Sherbrooke, Sherbrooke, Quebec, Canada, **2009**; http://www.cylview.org.
